# Independent validation of induced overexpression efficiency across 242 experiments shows a success rate of 39%

**DOI:** 10.1038/s41598-018-36122-8

**Published:** 2019-01-23

**Authors:** Gyöngyi Munkácsy, Péter Herman, Balázs Győrffy

**Affiliations:** 1 Semmelweis University 2nd Dept. of Pediatrics, Budapest, Hungary; 2 MTA TTK Lendület Cancer Biomarker Research Group, Institute of Enzymology, Budapest, Hungary

## Abstract

Although numerous studies containing induced gene expression have already been published, independent authentication of their results has not yet been performed. Here, we utilized available transcriptomic data to validate the achieved efficiency in overexpression studies. Microarray data of experiments containing cell lines with induced overexpression in one or more genes were analyzed. All together 342 studies were processed, these include 242 different genes overexpressed in 184 cell lines. The final database includes 4,755 treatment-control sample pairs. Successful gene induction (fold change induction over 1.44) was validated in 39.3% of all genes at p < 0.05. Number of repetitions within a study (p < 0.0001) and type of used vector (p = 0.023) had significant impact on successful overexpression efficacy. In summary, over 60% of studies failed to deliver a reproducible overexpression. To achieve higher efficiency, robust and strict study design with multi-level quality control will be necessary.

## Introduction

Reproducibility and reliability of findings identified in experimental studies was repeatedly questioned in recent years^1^. For example, when we compared 24 studies set-up to recognize RAS-responsive genes previously, only 8% of the targets were identified in more than one study^2^. As many discoveries in cancer biology of the last two decade failed to translate into clinically useful new therapies, reproducibility got into the spotlight^3–5^. For studies containing genomic data, like transcriptomic analyses employing gene arrays, current guidelines require transparent design and the publication of raw data. On the contrary, it is either impossible or with significant inconsistencies to reproduce more than half of published projects in independent repetition studies^6,7^. Overall, these discrepancies result in loss of confidence in the experimental results and also emphasize the necessity of independent validation studies.

Increased gene expression can cause intense phenotypic changes in a variety fields of biology including human cancers^8,9^. Since the first reported description confirming the correlation between gene dosage and function^10^, experimental overexpression methods became widely utilized. At the same time, technological difficulties are reported in many instances. Little efficacy and short time of gene expression induction remain an open issue^11^. Metabolic stress and instability of the plasmids are similar difficulties yet to solve^12^. Correct design can help to improve efficacy, safety and production of DNA vectors^13,14^. Therefore, vector design^15,16^ and other DNA vector topology strategies^17^ were proposed to enhance plasmid performance. Despite of all the progress, multiple overexpression studies^18–20^ had to be withdrawn because of unreliable data.

Gene arrays were designed to simultaneously measure the expression of almost all human genes. When analysed in patients with the same clinical characteristics, they can be used to compare and rank regarding clinical relevance a large number of mRNAs and miRNAs^21,22^. Expression changes can be measured before and after any treatment including effects of induced gene overexpression by comparing the treated and untreated cell line samples. In this, such a genome-wide analysis enables to identify both target and off target effects.

Until today, hundreds of studies employed gene arrays in overexpression studies utilizing different cell lines from diverse tissue types. Here, our aim was to identify these projects, re-process the raw gene expression data, and to measure the efficacy of induced overexpression. By running this analysis across a large number of independent studies, we aimed to identify factors determining efficacy of gene induction.

## Results

### Database construction

**Figure 1:**
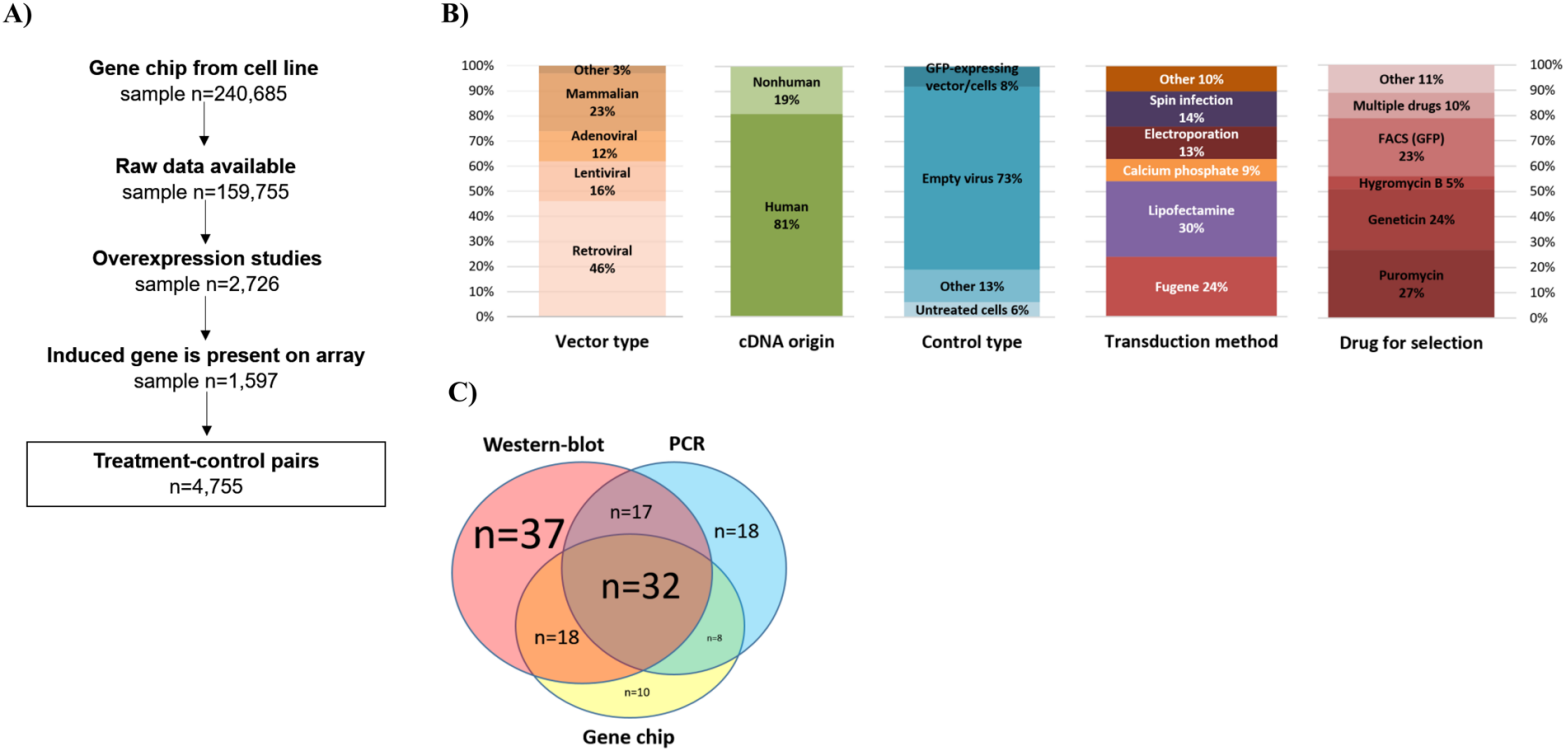
Summary of the database setup (**A**) and properties of the included studies including vector type, origin of cDNA, type of control used, transduction method, and drug used for selection (**B**), and validation techniques utilized (**C**).

The search identified 240,685 cell culture samples in 5,066 datasets in GEO. Of these, 342 datasets including 2,726 samples described a study with induced gene expression. As the gene arrays do not include every single gene, we had to exclude studies where the investigated gene was absent in the particular array platform. This reduced to the total number of eligible samples with an expression induction to 1,597. In the covered studies 242 unique genes were overexpressed in 184 distinct cell lines originating in twenty different tissue types, with a total number of 4,755 treated-control pairs (Fig. 1A).

We collected descriptive characteristics of the methods used in the studies included in the analysis. Retroviral vectors were utilized in almost half of the studies (46%), followed by mammalian constructs (23%), lentiviral (16%), and adenoviral vectors (12%). Human or mutated human cDNA construct were built in 81% the plasmids. Empty virus was used as a control in 73% of the studies. There was no dominant method across all studies for transduction, however, Lipofectamine and Fugene transfection reagents were used in more than half of the studies. Methods for the selection of stable transfectant include puromycin (27%), geneticin (24%), and GFP-based FACS (23%) (Fig. 1B). Two-third of the studies used cancer cell lines. When checking the tissue type, breast cell lines were utilized in 21% of the studies (n = 51), followed by blood (n = 39) and lung (n = 25) cell lines.

Surprisingly, in 43 overexpression studies (22.6%) no validation method to test the efficacy of the induced gene expression was mentioned at all. Western blot alone was the most popular validation method (19%), followed by a combination with PCR and microarrays (see in Fig. 1C). A complete list of all datasets with the investigated gene is presented in Supplemental Table 1.

### Percentage of successful overexpression

**Figure 2:**
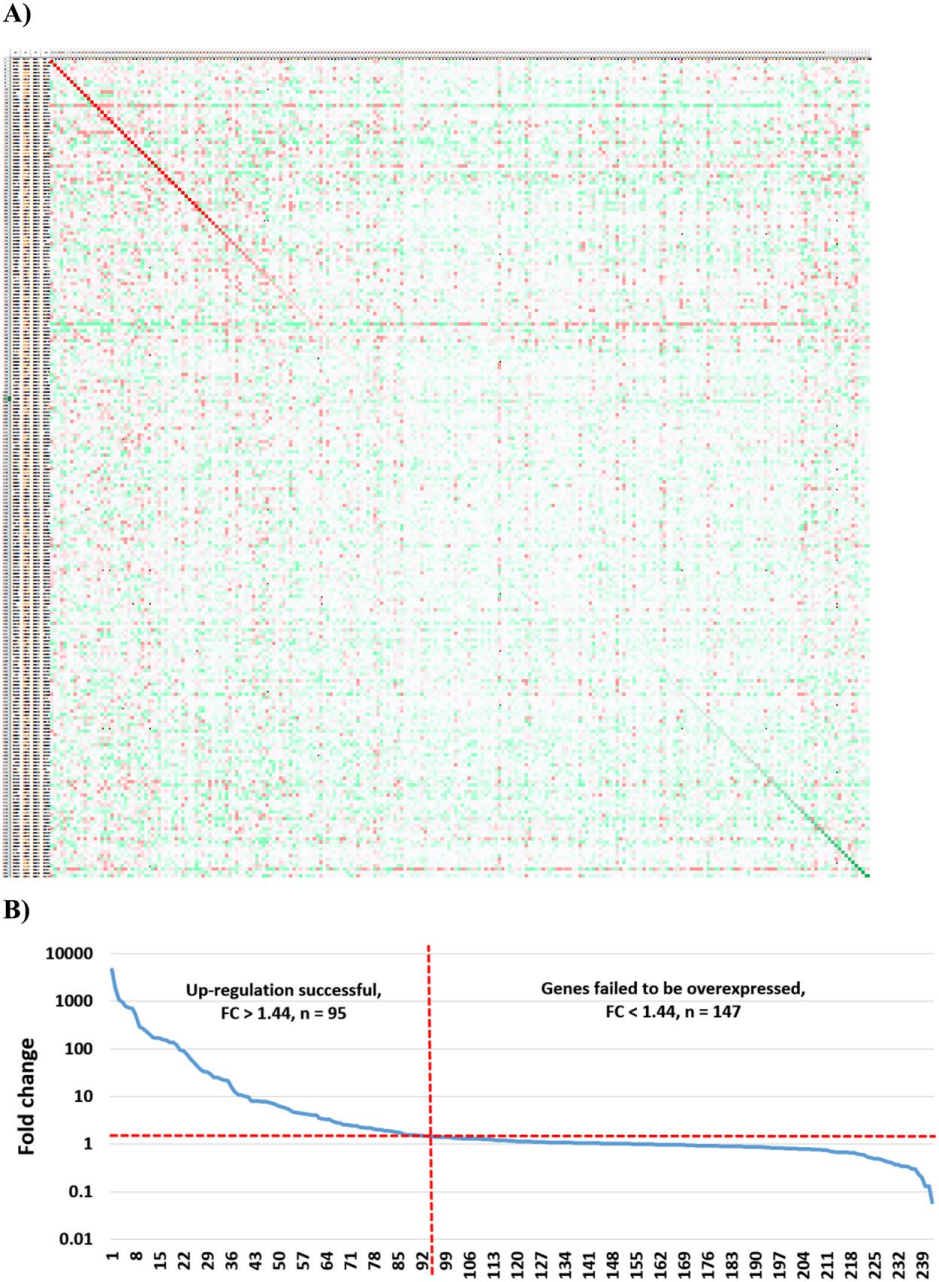
Efficacy of overexpression across all genes. Heat map demonstrating the efficacy of gene overexpression across all experiments (**A**). Columns are overexpressed genes and the rows are experiments. Green color refers to lower, while red means higher expression in the treated sample compared to control. Genes and experiments are ranked so that the diagonal (red) line corresponds to the overexpression efficacy of the targeted gene in the particular experiment. A ranked order of all genes based on fold change (**B**). Values below one indicate down-regulation, values over one indicate up-regulation. The dotted red line shows 1.44x fold change. Robust gene induction was confirmed in 39% of all genes.

Gene expression in relation to the expression in the control samples for each investigated gene in each study is displayed as a heat map in Fig. 2A. (Full resolution image of Fig. 2 is available as Supplemental Fig. 1).

When looking only on the genes subject of overexpression across all studies, a fold change (FC) of up-regulation over two-fold was reached by 80 genes (32.9%) and over 1.44 by 95 genes (39.3%). The highest fold change was realized in GSE12513 - here, a MiaPaCa2 cell line was induced with AGR2 overexpression in 9 replicates (p = 0.009152; FC = 4612). The highest significance was achieved in GSE9936 using repeated experiments at different time points for ESR2 induction in the MCF7 cell line (p = 1.73E-38; FC = 966.7). On the other hand, the fold change did not reached 1.44 in 147 experiments. Opposite outcome (decreased expression instead of gene induction, FC ≤ 1.0) was delivered in 35% of the studies. The ranked fold change for all genes is displayed in Fig. 2B.

### Effect of experimental parameters on achieved overexpression

Efficacy of the overexpression was examined across all cell lines, tissue types, number of replicates, vector types, control types, drugs for selection, transduction methods, methods for validation of expression changes and publication year. When comparing the origin of the used cell lines (cancer, noncancer or stem cells) and utilization of different tissue types, there was no significant effect on overexpression efficiency (p = 0.8, n = 242; and p = 0.16, n = 242, respectively).

**Figure 3:**
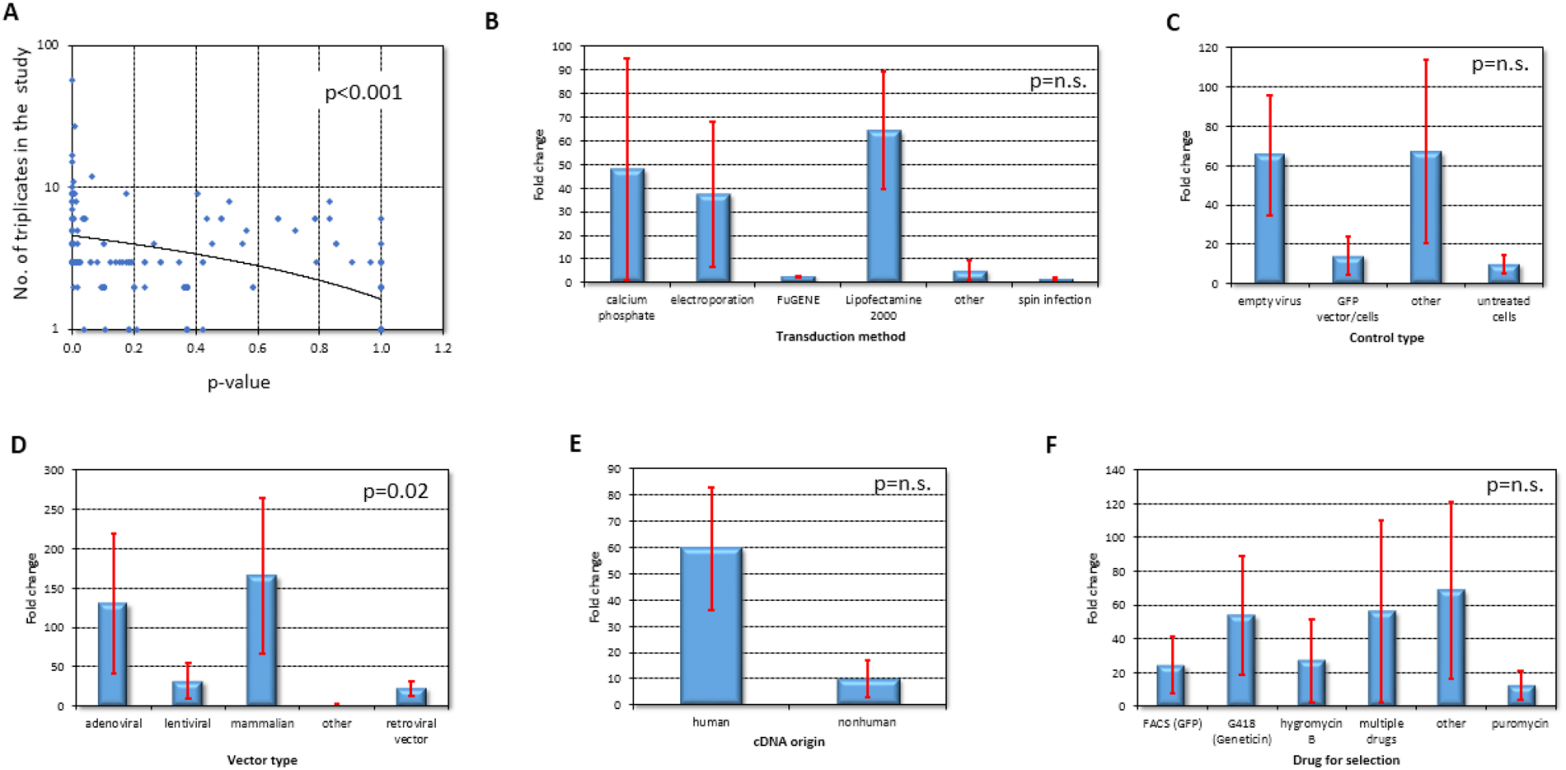
Overexpression efficacy in the examined models and techniques. More replicates in a study lead to increased overexpression efficacy (**A**), while selection of transduction method and control type had no effect on it (**B**,**C**, respectively). Vector type and origin of cDNA had significant correlation (**D**,**E**, respectively), but selection reagents were again insignificant (**F**).

As expected, there was a significant correlation between number of replicates used and achieved significance of the overexpression. (p < 0.001, n = 242, Fig. 3A).

The employed transduction method and the type of control utilized did not had a significant effect on overexpression efficiency (p = 0.13, n = 130, Fig. 3B; and p = 0.09, n = 216, Fig. 3C, respectively and Supplemental Table 1). Similarly, the year of publication and the method used to validate the expression changes had no influence on overexpression significance (p = 0.36, n = 242; and p = 0.22, n = 190, respectively).

The type of vector employed had a significant effect on overexpression efficiency by Kruskal-Wallis test (p = 0.022, n = 206, Fig. 3D). Best results were obtained in the mammalian origin (mean FC = 165, n = 48), followed by adenoviral (mean FC = 130, n = 24), and then lentiviral vectors (mean FC = 32, n = 34).

Another correlation was uncovered when the origin of the cDNA was considered, with a lower FC in experiments using nonhuman cDNA (n = 64, Fig. 3E). Finally, the drugs used for the selection of stable transfectant had no significant effect on overexpression efficacy (p = 0.97, n = 131, Fig. 3F, see also Supplemental Table 1).

## Discussion

Here, we performed a large-scale validation of experimental gene induction initiated by DNA vectors or plasmids. We processed all together 342 autonomous experiments where published raw gene array data enabled independent re-validation of the results. Surprisingly, the overall success rate was merely at 39%. Alarmingly, this is only minimally higher than the 35% proportion of studies where the outcome was actually opposite (decreased expression instead of gene induction).

Low reproducibility of academic science is an important real issue and its origins have to be promptly uncovered^1,3,23,24^. The recently launched “Reproducibility Project: Cancer Biology” has set the goal to independently validate fifty high-impact cancer studies. For this goal, studies published between 2010 and 2012 are objectively evaluated^4^. The first reports of the project were published earlier this year with contradictory results. Out of five studies, one was not reproducible at all, two reproduced the original results – but statistical significance was lost in some cases, and two papers were substantially different and did not enable clear interpretation^25^.

We analyzed variables potentially influencing overexpression efficacy including the utilized transduction method, the type of control used, the vector type, the origin of the cDNA, the drug used for selection, and the number of repetitions within a given study. Of these, only the type of vector, the origin of the cDNA, and the number of repetitions delivered significant correlation with better gene induction. Of note, there was also no correlation between publication date and gene induction efficiency proposing that current techniques are not superior compared to methods available a decade ago. However, we have to note that many of the studies published during the investigated time span used similar techniques.

Today, different vector types available include those with a viral origin (retroviral, adenoviral, and lentiviral) and mammalian plasmids. In our analysis, mammalian types gave the highest overexpression. These results are in line with previous experimental observations^26,27^. Secondly, origin of cDNA also gave significant correlation. We have to note that this result was derived from only 62 overexpression experiments as the origin of cDNA was not disclosed in most of the studies. Similarly, in more than in half of the studies (58%) the transfection method was also not available at all.

We observed the strongest correlation between higher number of repetitions and efficacy of overexpression. This is not surprising as in a reliable experimental setup additional samples with low standard deviations increase the statistical power due to the concept of current tests. Overall, almost all studies with more than three untreated and three treated samples (in other word at least ten pairs available for relation analysis) achieved a significant overexpression.

Once the vector construction and experiment is completed, the efficacy of gene induction has to be verified by an independent method. Western blot, qRT-PCR, and gene chips are used in most cases for validation at the protein or mRNA level. Other techniques were mentioned in only seven studies (3.5%). Surprisingly, 22.6% of all studies did not employed any validation method. This negligence might be one of the reasons for the high failure rate detected in our analysis.

Despite our expectations, the success rate in our validation analysis was extremely low. We have to emphasize another even more dramatic issue in this regard – we performed an analysis which was already feasible for the original authors. In other words, we merely re-computed the actual expression changes using data they provided. Therefore, methodological and biological deviations like the ones disclosed in the “Reproducibility Project: Cancer Biology” studies should not have any effect on our analysis results.

We must list some important limitations of our study. First, we utilized data downloaded from GEO – however, a publication within GEO does not guarantee that the data meets any particular standard of quality as a dataset can be uploaded even without a peer reviewed manuscript. To ease this potential bias, we looked up corresponding publications for each dataset. The results of all together 201 (83%) studies were also published in a peer-reviewed paper (Supplemental Table 1). The high percentage supports the overall reliability of the datasets used in our analysis. In addition, one should note that the quality of the data in GEO can also be easily visualized through the integrated GEO2R platform.

Second, we have to mention potential cross-species issues. It is possible that in some cases mouse cDNAs were over-expressed in human cells, or vice versa. If cDNA from a different species was used, it is likely that the probe set on the gene chip was not sufficiently homologous to recognize that cDNA, or at least it recognized it poorly. We did not had the exact sequences of the over-expressed genes; thus we were not able to blast these to validate their specificity.

Deep sequencing studies might do a much better job at estimating the true abundance of RNAs than microarrays. In case the overexpressed gene is substantially mutated or modified, it might not bind on the GeneChip probes. However, we were not able to utilize RNAseq data, because there is still limited data available (the 117datasets currently in SRA dealing with over-expression were generated using fifteen different sequencing platforms and these can have different sensitivity, specificity and dynamic range for the same genes).

Finally, we have to note that the overexpression of a gene will not necessarily lead to higher protein levels because these are influenced by additional factors including translation factors, folding, post-translational modifications, etc. A balance in the different steps including copy number, transcription, translation, folding, and secretion is important for better protein overexpression.

In summary, we performed a large-scale validation of overexpression efficiency by taking advantage of available transcriptomic profiles published in many of these studies. Surprisingly, the magnitude of the achieved overexpression was insufficient in the bulk of all experiments. Therefore, cautious study design is necessary – this should include selection of a proper validation method, utilizations of more than three replicates, and the careful selection of vector types as these had the most significant influence on overexpression proficiency.

## Methods

### Database construction for overexpression studies

A GEO search was performed in the NCBI GEO database. The search was set to include all studies published 2005–2017 using following platforms: Affymetrix HGU133A, Affymetrix HGU133A plus2 and Affymetrix HGU133A v2 microarrays. The R-based GEOsql package was used to perform a text based search in the database to identify cell line based studies among the hits as described earlier^28^. Hereafter, each identified study was manually evaluated to isolate those with an overexpression study. Only studies fulfilling following criteria were included in the final set: A) the study design included at least one untreated control, and B) overexpression of only one gene was induced in the treatment-control pair. The analysis was not restricted to studies examining solely wild-type genes – in other words experiments using a mutated form of a gene were designated as eligible. Finally, experimental data describing the performed methods including method of validation, vector type, selection method, utilized cell line, method of transfection, year of publication, type of control samples, and origin of cDNA were extracted for each study.

### Re-processing of microarrays

The raw CEL files for each gene array re-normalized using MAS5 in R. We selected MAS5 because it enabled to independently process thousands of arrays and it also ranked among the best method when compared to RT-PCR expression in our earlier study^29^. Array quality was gauged by evaluating the parameters of percentage of present calls, background intensity, noise, presence of the spike-in controls and the RNA degradation profile as described previously^30^.

### Statistical analyses

The overexpressed and the corresponding control experiments were paired in each experiment. The mean fold change was calculated across all possible pairs for each gene in each study to assess the extent of expression intensification. Non-parametric Wilcoxon signed-rank test was computed to compare control samples to those with an induced overexpression. Kruskall-Wallis H-test was used for comparisons involving multiple cohorts. Continuous variables were compared using Mann-Whitney U tests. Spearman rank correlation was calculated to measure the strength of association between variables. Cutoff for statistical significance was set at p < 0.05.

## Electronic supplementary material

Supplementary Information
