# Supplementary material for: Independent validation of induced overexpression efficiency across 242 experiments shows a success rate of 39%
Source: Sci Rep. 2019 Jan 23;9:343. doi: 10.1038/s41598-018-36122-8 (PMC6344512; doi:10.1038/s41598-018-36122-8)
Supplement: Supplementary file 1 — Supplementary Information [file 41598_2018_36122_MOESM1_ESM.pdf]

# Independent validation of induced overexpression efficiency across 242 experiments shows a success rate of 39%

Gyöngyi Munkácsy[1,2*], Péter Herman[2], Balázs Győrffy[1,2]

[1] Semmelweis University 2nd Dept. of Pediatrics, Budapest, Hungary,

[2] MTA TTK Lendület Cancer Biomarker Research Group, Institute of Enzymology, Budapest, Hungary

**Supplemental Table 1:** GEO datasets used in the study as well as detailed parameters and results for each gene.

| Gene symbol | Affymetrix probe set | Dataset | PMID | Calculated fold change | Fold change in the original study | Vector type (1= retroviral vector 2=lentiviral 3= adenoviral 4=mammalian 5= other) | cDNA origin (human=1 nonhuman=2) | Control type (1=empty/control/virus 2=untreated cells 3=GFP-expressing vector/cells 4=other) | Transduction method (1=FuGENE 2=Lipofectamine (2000) 3=calcium phosfate 4=electroporation 5=spin infection 6=other) | Drug for selection (1=puromycin, 2=G418 (Geneticin) , 3=hygromycin B 4=FACS (GFP) 5=multiple drugs 6=other) |
|---|---|---|---|---|---|---|---|---|---|---|
| YAP1 | 213342_at | GSE10196 | 18413746 | 0.78 | NA | 1 | 1 | 1 | 1 | NA |
| CLDN1 | 218182_s_at | GSE10309 | 18787218 | 37.13 | NA | NA | 1 | 1 | NA | 2 |
| RUNX1 | 209360_s_at | GSE10520 | 19043539 | 0.59 | NA | NA | 1 | 1 | 2 | NA |
| PRDM5 | 220792_at | GSE10580 | 19169355 | 293.65 | NA | 4 | 1 | 1 | NA | 1 |
| CDHR2 | 220186_s_at | GSE10650 | 19383367 | 169.81 | NA | NA | NA | 3 | NA | NA |
| SOX17 | 219993_at | GSE10809 | 18682240 | 3.45 | NA | 4 | NA | 2 | 4 | 6 |
| SMARCA4 | 213720_s_at | GSE10847 | NA | 1.75 | NA | NA | NA | NA | NA | NA |
| TGFBR2 | 208944_at | GSE10847 | NA | 0.44 | NA | NA | NA | NA | NA | NA |
| ESR2 | 211120_x_at | GSE11115 | 18097065 | 33.54 | NA | 2 | NA | 2 | NA | 1 |
| GLB1 | 201576_s_at | GSE11266 | 18974123 | 1.34 | NA | 3 | NA | 4 | NA | NA |
| SOX2 | 213721_at | GSE11330 | 18456656 | 0.37 | NA | 3 | NA | 4 | NA | NA |
| ITGB4 | 204990_s_at | GSE11466 | 19011242 | 2.52 | NA | 4 | NA | 1 | 2 | 2 |
| XIAP | 206536_s_at | GSE11618 | 19034449 | 0.33 | NA | NA | NA | NA | NA | NA |
| MYC | 202431_s_at | GSE11791 | 18714337 | 5.45 | 4 | 5 | 2 | 1 | NA | 1 |
| KLF9 | 203543_s_at | GSE11855 | 18783612 | 91.59 | 2.4 | 4 | 2 | NA | 2 | 2 |
| IL6 | 205207_at | GSE11883 | 17079474 | 0.9 | NA | 5 | NA | 1 | 6 | 2 |
| SOX4 | 201417_at | GSE11914 | 19147588 | 2.26 | 2.8 | 2 | NA | 1 | 2 | NA |
| CTNNB1 | 201533_at | GSE11916 | NA | 1.08 | NA | NA | NA | 1 | NA | NA |
| PA2G4 | 214794_at | GSE12200 | 18852121 | 0.96 | 3 | 5 | NA | NA | 1 | 2 |
| MEX3D | 91816_f_at | GSE12239 | NA | 0.13 | NA | NA | NA | 4 | 2 | NA |
| NOTCH1 | 218902_at | GSE12355 | 19339697 | 1.03 | NA | 4 | 1 | 4 | 4 | 3 |
| CAT | 201432_at | GSE12355 | 19339697 | 0.91 | NA | 4 | 2 | NA | 4 | 3 |

| | | | | | | | | | | |
|---|---|---|---|---|---|---|---|---|---|---|
| NOTCH2 | 212377_s_at | GSE12355 | 19339697 | 0.82 | NA | 4 | 1 | 4 | 4 | 3 |
| AGR2 | 209173_at | GSE12513 | NA | 4612.35 | NA | 4 | NA | 1 | NA | NA |
| HRAS | 212983_at | GSE12790 | 19567590 | 21.77 | NA | 3 | 1 | 1 | NA | NA |
| MAP2K1 | 202670_at | GSE12790 | 19567590 | 1.26 | NA | 3 | 1 | 1 | NA | NA |
| PRKAR2B | 203680_at | GSE12854 | 18822129 | 32.61 | NA | 1 | 1 | 2 | NA | NA |
| PRKAR1A | 200603_at | GSE12854 | 18822129 | 0.89 | NA | 1 | 1 | 2 | NA | NA |
| HLX | 214438_at | GSE13054 | 21224470 | 0.34 | NA | 3 | 1 | 1 | 3 | NA |
| OLR1 | 210004_at | GSE13139 | 19279231 | 0.96 | NA | 3 | 1 | 3 | NA | NA |
| NR0B1 | 206645_s_at | GSE1318 | 15105423 | 2.42 | NA | 3 | 2 | 1 | 1 | NA |
| TCFL5 | 204849_at | GSE1318 | 15105423 | 1.36 | NA | 3 | 2 | 1 | 1 | NA |
| MYB | 204798_at | GSE1318 | 15105423 | 0.97 | NA | 3 | 2 | 1 | 2 | NA |
| MYBL1 | 213906_at | GSE1318 | 15105423 | 0.78 | NA | 3 | 2 | 1 | 2 | NA |
| CD9 | 201005_at | GSE13210 | NA | 1066.88 | NA | 4 | NA | 4 | NA | 2 |
| YAP1 | 213342_at | GSE13218 | NA | 1.12 | NA | 1 | NA | 1 | NA | NA |
| TP73 | 220804_s_at | GSE13504 | 19001418 | 136 | 21 | 3 | NA | 1 | 2 | NA |
| MKL1 | 212748_at | GSE13710 | 19136660 | 1.03 | 10 | 2 | NA | 1 | 4 | 3 |
| BRAF | 206044_s_at | GSE13827 | NA | 12.81 | NA | 2 | NA | 1 | NA | NA |
| PURA | 204020_at | GSE14464 | 19267365 | 1.19 | 50 | 1 | 1 | 4 | 2 | 1 |
| PURA | 204020_at | GSE14464 | 19267365 | 1.03 | NA | 1 | 1 | 4 | 2 | 1 |
| SLAMF7 | 219159_s_at | GSE14680 | 19196658 | 7.92 | NA | NA | NA | NA | NA | NA |
| MYOD1 | 206657_s_at | GSE14825 | 19299559 | 1.4 | NA | 1 | 1 | 1 | 6 | 1 |
| ERBB2 | 216836_s_at | GSE14987 | 20133671 | 0.65 | NA | 1 | NA | 1 | 3 | NA |
| AR | 211110_s_at | GSE15091 | 19668381 | 268.62 | NA | 4 | NA | 1 | 2 | NA |
| BCR | 202315_s_at | GSE15811 | 20554971 | 25.57 | NA | 1 | NA | 3 | NA | 4 |
| FGFR1 | 210973_s_at | GSE15811 | 20554971 | 25.57 | NA | 1 | NA | 3 | NA | 4 |
| ZMYM2 | 202778_s_at | GSE15811 | 20554971 | 0.95 | NA | 1 | NA | 3 | NA | 4 |
| ABL1 | 202123_s_at | GSE15811 | 20554971 | 0.93 | NA | 1 | NA | 3 | NA | 4 |
| CXCR4 | 217028_at | GSE15893 | 20603605 | 4.8 | 6.81 | 1 | NA | NA | NA | 2 |
| SNAI1 | 219480_at | GSE16194 | 19887480 | 9.96 | NA | 1 | NA | 1 | NA | 3 |

| | | | | | | | | | | |
|---|---|---|---|---|---|---|---|---|---|---|
| NOTCH3 | 203238_s_at | GSE16254 | 23649002 | 3.33 | 170 | 4 | NA | NA | NA | NA |
| MSX1 | 205932_s_at | GSE16254 | 23649002 | 1.26 | NA | 4 | NA | NA | NA | NA |
| JAG1 | 216268_s_at | GSE16547 | 19816565 | 1.06 | NA | 2 | 1 | 1 | NA | NA |
| PRAME | 204086_at | GSE17100 | 19625708 | 1.8 | NA | 2 | NA | 3 | 3 | 4 |
| IKBKB | 209341_s_at | GSE17129 | 19628709 | 1.2 | NA | 1 | NA | 1 | 4 | 6 |
| USP2 | 207213_s_at | GSE17466 | 22585994 | 7.52 | NA | 1 | NA | 1 | 2 | 1 |
| NFIC | 206929_s_at | GSE17636 | 20145151 | 1.06 | NA | 4 | NA | 1 | 2 | NA |
| PIK3CA | 204369_at | GSE17785 | NA | 0.65 | NA | NA | NA | NA | NA | NA |
| MAP2K5 | 211370_s_at | GSE17939 | 20551324 | 2.43 | NA | 1 | NA | 1 | 3 | 1 |
| MAP3K14 | 205192_at | GSE18047 | 20053756 | 1.47 | NA | 1 | NA | 1 | 5 | 1 |
| MAP3K14 | 205192_at | GSE18047 | 20053756 | 1.08 | NA | 1 | NA | 1 | 5 | 1 |
| IKBKB | 209341_s_at | GSE18047 | 20053756 | 0.94 | NA | 1 | NA | 1 | 5 | 1 |
| IKBKB | 209341_s_at | GSE18047 | 20053756 | 0.92 | NA | 1 | NA | 1 | 5 | 1 |
| SMAD7 | 204790_at | GSE18070 | 20086175 | 0.3 | NA | 1 | NA | 1 | 1 | 1 |
| ZBTB16 | 205883_at | GSE18476 | 19855079 | 0.48 | NA | 1 | NA | 1 | 1 | NA |
| RALA | 214435_x_at | GSE19295 | NA | 2.55 | NA | 1 | NA | 1 | NA | NA |
| PPARGC1A | 219195_at | GSE19643 | NA | 0.83 | NA | 3 | NA | NA | NA | NA |
| HSF1 | 202344_at | GSE19797 | 20834230 | 0.42 | NA | 4 | 1 | 2 | 3 | 6 |
| IKBKB | 209341_s_at | GSE20115 | 20436485 | 1.39 | NA | 4 | NA | 1 | 4 | 4 |
| KAT8 | 221820_s_at | GSE20193 | 20479123 | 1.46 | NA | 4 | 1 | 1 | 6 | 2 |
| NOTCH1 | 218902_at | GSE20285 | 20194747 | 1.12 | NA | 1 | NA | 1 | 3 | NA |
| TP63 | 209863_s_at | GSE20286 | 16715076 | 0.52 | NA | 1 | NA | 1 | 3 | NA |
| YWHAZ | 200639_s_at | GSE20318 | 22912335 | 1.09 | NA | 4 | NA | 4 | 2 | 6 |
| CA9 | 205199_at | GSE20569 | 21363891 | 8.1 | NA | 4 | 1 | 1 | 2 | 2 |
| TP53 | 201746_at | GSE21105 | 20833636 | 0.5 | NA | 4 | NA | 2 | 2 | 2 |
| BCL11B | 219528_s_at | GSE21382 | 20824091 | 0.49 | 20 | 1 | 1 | 1 | NA | 4 |
| MYC | 202431_s_at | GSE22139 | 21317922 | 6.01 | NA | 4 | 1 | 1 | 1 | 2 |
| ESR1 | 205225_at | GSE22593 | 20547749 | 1.39 | NA | 4 | 1 | NA | NA | NA |
| EHMT2 | 202326_at | GSE22810 | NA | 2.18 | NA | NA | NA | 1 | NA | 1 |

| | | | | | | | | | | |
|---|---|---|---|---|---|---|---|---|---|---|
| EHMT2 | 202326_at | GSE22810 | NA | 0.06 | NA | NA | NA | 1 | NA | 1 |
| CDH3 | 203256_at | GSE23360 | NA | 205.96 | NA | 1 | NA | 1 | NA | NA |
| SETD8 | 220200_s_at | GSE24170 | 20932471 | 2.16 | NA | 1 | 1 | 1 | 2 | 1 |
| GATA3 | 209602_s_at | GSE24249 | 21892208 | 0.34 | NA | 2 | NA | 1 | NA | 2 |
| RASSF1 | 204346_s_at | GSE24473 | 20955597 | 0.78 | NA | NA | NA | NA | NA | NA |
| NDRG1 | 200632_s_at | GSE25086 | NA | 0.67 | NA | NA | NA | 1 | NA | NA |
| MAP2K5 | 211370_s_at | GSE25145 | 21166929 | 1.71 | 8.5-48 | 1 | NA | 4 | NA | 1 |
| EPCAM | 201839_s_at | GSE25743 | NA | 10.58 | NA | 4 | NA | 1 | NA | 1 |
| ZBTB16 | 205883_at | GSE26272 | 21547890 | 153.83 | NA | 2 | NA | 1 | NA | 6 |
| WNT1 | 208570_at | GSE26656 | 22572818 | 156.88 | NA | 1 | NA | 1 | 2 | NA |
| WNT1 | 208570_at | GSE26656 | 22572818 | 138.69 | NA | 1 | NA | 1 | 2 | NA |
| CFAP45 | 220308_at | GSE27318 | 20715168 | 43.71 | NA | 2 | 1 | 1 | 2 | NA |
| ZNF83 | 221645_s_at | GSE27444 | 21818254 | 1.93 | NA | 1 | NA | 2 | 1 | 5 |
| STIP1 | 213330_s_at | GSE27654 | 22884369 | 1.05 | NA | 5 | NA | 4 | 4 | NA |
| PRDM1 | 217192_s_at | GSE27670 | 21411757 | 94.18 | NA | 4 | NA | 1 | 4 | 4 |
| MYB | 204798_at | GSE2815 | 16205643 | 0.88 | NA | 3 | 2 | 1 | NA | NA |
| MYB | 204798_at | GSE2816 | 16205643 | 2.23 | NA | 3 | 2 | 1 | NA | NA |
| RUNX1 | 209360_s_at | GSE28317 | 21764752 | 1.03 | NA | 1 | NA | 1 | 3 | NA |
| NUMB | 207545_s_at | GSE29137 | NA | 2.07 | NA | 4 | NA | 1 | NA | 2 |
| NUMB | 207545_s_at | GSE29137 | NA | 1.09 | NA | 4 | NA | 1 | NA | 2 |
| CYR61 | 201289_at | GSE29384 | 22282654 | 1.34 | NA | NA | NA | NA | NA | NA |
| SNAI2 | 213139_at | GSE29672 | 22028892 | 1.29 | NA | 3 | NA | 2 | NA | NA |
| RB1 | 203132_at | GSE29783 | 23212373 | 0.74 | NA | 2 | NA | NA | NA | NA |
| SFTPC | 38691_s_at | GSE2980 | 16449190 | 173.51 | NA | 4 | 1 | 1 | 2 | 3 |
| NOTCH1 | 218902_at | GSE29850 | 21839921 | 1.05 | NA | 1 | NA | 1 | 3 | 2 |
| RAF1 | 201244_s_at | GSE29884 | NA | 1.15 | NA | 1 | NA | 2 | NA | NA |
| NOTCH1 | 218902_at | GSE29959 | 21807868 | 1.51 | NA | 1 | NA | 4 | 5 | NA |
| NOTCH1 | 218902_at | GSE29959 | 21807868 | 1.24 | NA | 1 | NA | 4 | 5 | NA |
| NOTCH1 | 218902_at | GSE29959 | 21807868 | 1.18 | NA | 1 | NA | 4 | 5 | NA |

| | | | | | | | | | | |
|---|---|---|---|---|---|---|---|---|---|---|
| NOTCH1 | 218902_at | GSE29959 | 21807868 | 1.12 | NA | 1 | NA | 4 | 5 | NA |
| MYC | 202431_s_at | GSE29959 | 21807868 | 0.92 | NA | 1 | NA | 4 | 5 | NA |
| NOTCH1 | 218902_at | GSE29959 | 21807868 | 0.89 | NA | 1 | NA | 4 | 5 | NA |
| MYC | 202431_s_at | GSE29959 | 21807868 | 0.86 | NA | 1 | NA | 4 | 5 | NA |
| MYC | 202431_s_at | GSE29959 | 21807868 | 0.78 | NA | 1 | NA | 4 | 5 | NA |
| MYC | 202431_s_at | GSE29959 | 21807868 | 0.75 | NA | 1 | NA | 4 | 5 | NA |
| MYC | 202431_s_at | GSE29959 | 21807868 | 0.68 | NA | 1 | NA | 4 | 5 | NA |
| BRCA1 | 204531_s_at | GSE30296 | 22685544 | 0.88 | NA | 4 | NA | 1 | 2 | 2 |
| NEUROG3 | 207965_at | GSE30802 | 22606327 | 22 | NA | 3 | 2 | 1 | NA | 4 |
| FLI1 | 204236_at | GSE31215 | 20382729 | 2.95 | NA | 1 | 1 | 1 | 1 | 6 |
| EWSR1 | 209214_s_at | GSE31215 | 20382729 | 1.1 | NA | 1 | 1 | 1 | 1 | 6 |
| MYC | 202431_s_at | GSE31311 | 21913186 | 735.49 | NA | 4 | NA | 1 | NA | NA |
| PRSS2 | 205402_x_at | GSE32056 | 22909050 | 0.41 | NA | 5 | 1 | 1 | NA | 2 |
| CDH1 | 201131_s_at | GSE32540 | 22752307 | 1.04 | NA | 2 | NA | 1 | NA | 4 |
| HRAS | 212983_at | GSE32975 | 22549044 | 10.21 | NA | NA | NA | 1 | NA | NA |
| RELA | 201783_s_at | GSE32975 | 22549044 | 1.02 | NA | NA | NA | 1 | NA | NA |
| EGFR | 201984_s_at | GSE32975 | 22549044 | 0.82 | NA | NA | NA | 1 | NA | NA |
| FTO | 209702_at | GSE33870 | 22872099 | 6.35 | 8-10 | 4 | NA | 1 | 1 | 5 |
| BHLHE41 | 221530_s_at | GSE33950 | 22801492 | 0.2 | NA | 1 | NA | 1 | 6 | 5 |
| MYC | 202431_s_at | GSE34055 | 22157079 | 0.96 | NA | 1 | NA | 1 | 6 | NA |
| ARHGDIB | 201288_at | GSE35014 | 22406535 | 3.28 | NA | 4 | 1 | 3 | NA | 1 |
| ZNF217 | 203739_at | GSE35511 | 22593193 | 0.82 | 2-3.5 | 4 | NA | 1 | NA | 6 |
| PPARGC1A | 219195_at | GSE36879 | 23416000 | 0.23 | NA | NA | NA | NA | NA | NA |
| LTF | 202018_s_at | GSE36972 | 23069661 | 169 | NA | 4 | NA | 1 | 2 | 2 |
| HOXA1 | 214639_s_at | GSE37136 | 23435427 | 4.27 | NA | 2 | 1 | 1 | NA | NA |
| FOXP3 | 221334_s_at | GSE37253 | 23244505 | 1.39 | NA | 1 | 1 | 1 | 3 | 4 |
| MITF | 207233_s_at | GSE38007 | 23477830 | 10.83 | NA | 4 | NA | 1 | NA | NA |
| WNT4 | 208606_s_at | GSE39949 | 23142810 | 4.39 | NA | 3 | NA | 1 | NA | NA |
| BMP2 | 205289_at | GSE39949 | 23142810 | 1.93 | NA | 3 | NA | 1 | NA | NA |

| | | | | | | | | | | |
|---|---|---|---|---|---|---|---|---|---|---|
| FOXO1 | 202724_s_at | GSE40543 | 24334454 | 0.37 | NA | 1 | 1 | 1 | NA | 1 |
| MED1 | 203497_at | GSE41150 | 23538858 | 1.07 | NA | 4 | 1 | 1 | 2 | 2 |
| PIAS3 | 203035_s_at | GSE42979 | 23959540 | 1.04 | NA | 1 | 2 | 1 | 1 | 2 |
| FOXC2 | 214520_at | GSE44335 | 23378344 | 62.74 | NA | 1 | 1 | 1 | NA | 6 |
| NFKBIA | 201502_s_at | GSE44619 | 23635779 | 7.24 | 1.4 or more | 1 | NA | 1 | 5 | 4 |
| NFKBIA | 201502_s_at | GSE44619 | 23635779 | 5.73 | 1.4 or more | 1 | NA | 1 | 5 | 4 |
| NFKBIA | 201502_s_at | GSE44619 | 23635779 | 4.1 | 1.4 or more | 1 | NA | 1 | 5 | 4 |
| NFKBIA | 201502_s_at | GSE44619 | 23635779 | 1.55 | 1.4 or more | 1 | NA | 1 | 5 | 4 |
| IKBKB | 209341_s_at | GSE44619 | 23635779 | 1.09 | 1.4 or more | 1 | NA | 1 | 5 | 4 |
| IKBKB | 209341_s_at | GSE44619 | 23635779 | 0.87 | 1.4 or more | 1 | NA | 1 | 5 | 4 |
| IKBKB | 209341_s_at | GSE44619 | 23635779 | 0.84 | 1.4 or more | 1 | NA | 1 | 5 | 4 |
| IKBKB | 209341_s_at | GSE44619 | 23635779 | 0.77 | 1.4 or more | 1 | NA | 1 | 5 | 4 |
| NFKBIA | 201502_s_at | GSE44619 | 23635779 | 0.77 | 1.4 or more | 1 | NA | 1 | 5 | 4 |
| IKBKB | 209341_s_at | GSE44619 | 23635779 | 0.76 | 1.4 or more | 1 | NA | 1 | 5 | 4 |
| GRHL2 | 219388_at | GSE44807 | 23690579 | 0.3 | NA | 2 | NA | 3 | 1 | 1 |
| GRHL2 | 219388_at | GSE44808 | 23690579 | 0.63 | NA | 2 | NA | 3 | 1 | 1 |
| AR | 211110_s_at | GSE44924 | 23842682 | 0.98 | NA | 2 | NA | 1 | NA | 6 |
| HRAS | 212983_at | GSE45276 | 21474066 | 4.5 | NA | 1 | NA | 1 | NA | 5 |
| AKT1 | 207163_s_at | GSE45276 | 21474066 | 0.71 | NA | 1 | NA | 1 | NA | 5 |
| SOX2 | 213721_at | GSE45391 | NA | 16.17 | NA | 2 | NA | 1 | NA | 1 |
| DLC1 | 210762_s_at | GSE46214 | 24590291 | 0.93 | NA | NA | 1 | 2 | 2 | NA |
| HOXD13 | 207397_s_at | GSE46542 | NA | 29.34 | NA | 4 | NA | 1 | NA | 2 |
| HOXD9 | 205604_at | GSE46542 | NA | 1.53 | NA | 4 | NA | 1 | NA | 2 |
| PEBP1 | 210825_s_at | GSE47378 | 23975428 | 0.91 | NA | 2 | NA | 1 | 6 | 1 |

| | | | | | | | | | | |
|---|---|---|---|---|---|---|---|---|---|---|
| RAD21 | 200608_s_at | GSE47641 | 23955599 | 0.87 | NA | 1 | NA | 4 | NA | 4 |
| TAL1 | 206283_s_at | GSE48557 | 23836559 | 1.14 | NA | 1 | 1 | 1 | NA | 2 |
| FGF18 | 211485_s_at | GSE48567 | 24018557 | 786.95 | NA | 2 | 1 | 4 | 2 | 6 |
| ELK1 | 203617_x_at | GSE48792 | 23871667 | 0.92 | NA | 5 | NA | NA | 6 | 2 |
| RCAN1 | 208370_s_at | GSE48841 | 23954784 | 1.01 | NA | 3 | 1 | 4 | NA | NA |
| PML | 211012_s_at | GSE48842 | NA | 2.02 | NA | 1 | NA | 1 | NA | 2 |
| TAGLN | 205547_s_at | GSE48998 | 26847345 | 6.89 | NA | 4 | NA | 1 | 2 | 6 |
| E2F1 | 204947_at | GSE50051 | 24244455 | 1.56 | NA | 3 | NA | 3 | NA | NA |
| WHSC1 | 209054_s_at | GSE50072 | NA | 0.96 | NA | NA | NA | NA | NA | NA |
| HSPA4 | 208815_x_at | GSE50547 | NA | 1.28 | NA | NA | NA | 1 | NA | NA |
| HOXA5 | 213844_at | GSE50659 | 25875824 | 1.31 | NA | NA | NA | 4 | NA | NA |
| BAG1 | 202387_at | GSE51524 | 24523409 | 1.14 | NA | 1 | NA | 1 | 1 | 2 |
| IRF5 | 205469_s_at | GSE51717 | 25288773 | 2.8 | NA | 1 | 1 | 1 | 4 | 4 |
| IKBKB | 209341_s_at | GSE51717 | 25288773 | 1.06 | NA | 1 | 1 | 1 | 4 | 4 |
| RNF2 | 205215_at | GSE51928 | 26450788 | 1.28 | NA | 2 | NA | 3 | 4 | 2 |
| YWHAZ | 200639_s_at | GSE52032 | 25670079 | 0.67 | NA | 2 | NA | 1 | 4 | 6 |
| IGF1R | 203628_at | GSE5225 | 16611727 | 1.39 | NA | NA | NA | 2 | 2 | NA |
| NFKB1 | 209239_at | GSE52707 | 24319068 | 1.29 | NA | 1 | NA | 1 | 2 | 1 |
| H2AFY | 207168_s_at | GSE53103 | 25959814 | 1 | NA | 2 | NA | 1 | NA | 4 |
| H2AFY | 207168_s_at | GSE53103 | 25959814 | 0.93 | NA | 2 | NA | 1 | NA | 4 |
| ZFP36 | 201531_at | GSE53183 | 24401661 | 4.16 | NA | 4 | 1 | 1 | NA | NA |
| PRDM1 | 217192_s_at | GSE53283 | 24527393 | 4.69 | NA | NA | NA | 3 | NA | NA |
| PNP | 201695_s_at | GSE53604 | 24729470 | 0.99 | NA | 2 | NA | NA | 2 | 1 |
| ZC3HAV1 | 220104_at | GSE54342 | 25288727 | 2 | NA | 4 | NA | 3 | 1 | NA |
| SNAI2 | 213139_at | GSE55269 | 25100569 | 0.53 | NA | 1 | NA | 4 | 1 | 1 |
| CDH1 | 201131_s_at | GSE55529 | NA | 1.49 | NA | NA | NA | 3 | NA | NA |
| CDH1 | 201131_s_at | GSE55529 | NA | 0.99 | NA | NA | NA | 3 | NA | NA |
| ZEB1 | 212764_at | GSE55688 | 28394329 | 705 | NA | 1 | 1 | 1 | 3 | 5 |
| HRAS | 212983_at | GSE55688 | 28394329 | 1.6 | NA | 1 | 1 | 1 | 3 | 5 |

| | | | | | | | | | | |
|---|---|---|---|---|---|---|---|---|---|---|
| ERCC6 | 207347_at | GSE56049 | 22483866 | 1.86 | NA | 4 | NA | 1 | 6 | 5 |
| RECK | 205407_at | GSE56898 | 24931164 | 0.88 | NA | 2 | NA | 1 | 1 | 1 |
| RECK | 205407_at | GSE56898 | 24931164 | 0.69 | NA | 2 | NA | 1 | 1 | 1 |
| KDM8 | 220070_at | GSE56908 | NA | 0.83 | NA | NA | NA | 1 | NA | NA |
| NUP98 | 203195_s_at | GSE57194 | 20805992 | 1.02 | NA | 1 | NA | 1 | 4 | NA |
| RUNX1 | 209360_s_at | GSE57194 | 20805992 | 0.98 | NA | 1 | NA | 1 | 4 | NA |
| PML | 211012_s_at | GSE57194 | 20805992 | 0.97 | NA | 1 | NA | 1 | 4 | NA |
| SDCBP | 200958_s_at | GSE57760 | 25593999 | 0.61 | NA | 4 | NA | 1 | 1 | 2 |
| ARL2BP | 202092_s_at | GSE58224 | 25012295 | 0.98 | NA | 2 | 2 | 1 | 2 | NA |
| ARL2BP | 202092_s_at | GSE58224 | 25012295 | 0.87 | NA | 2 | 2 | 1 | 2 | NA |
| NR4A2 | 216248_s_at | GSE58475 | NA | 76.84 | NA | 1 | NA | 1 | NA | NA |
| MECOM | 221884_at | GSE60100 | 25886616 | 521.37 | NA | 1 | 1 | 1 | 4 | 4 |
| BCL3 | 204908_s_at | GSE60551 | 25366117 | 0.13 | NA | 2 | 1 | 4 | 6 | NA |
| FGFR3 | 204379_s_at | GSE61352 | 25223521 | 0.91 | NA | 1 | NA | 1 | 6 | 3 |
| YAP1 | 213342_at | GSE61764 | 26173433 | 1.02 | NA | 2 | 1 | 1 | NA | NA |
| CD99 | 201029_s_at | GSE61928 | 26123714 | 1.03 | NA | NA | NA | 2 | 3 | 6 |
| CD44 | 212063_at | GSE63862 | 25762343 | 52.06 | NA | 4 | 1 | 2 | 6 | NA |
| CAT | 201432_at | GSE63955 | NA | 1.13 | NA | NA | NA | 4 | NA | NA |
| DPP3 | 218567_x_at | GSE6451 | 17360324 | 8.09 | NA | 4 | 1 | 1 | 1 | NA |
| ID3 | 207826_s_at | GSE64535 | 25693514 | 3.43 | 7-8 | 1 | NA | 1 | 2 | 6 |
| TP53 | 201746_at | GSE64738 | NA | 1.02 | NA | NA | NA | 1 | NA | NA |
| TP53 | 201746_at | GSE64738 | NA | 1 | NA | NA | NA | 1 | NA | NA |
| CDH13 | 204726_at | GSE65363 | NA | 1.08 | NA | NA | NA | NA | NA | NA |
| HRAS | 212983_at | GSE6559 | 17440097 | 8.05 | NA | 1 | NA | 1 | 1 | 1 |
| MAP2K7 | 209952_s_at | GSE6559 | 17440097 | 7.75 | NA | 1 | NA | 1 | 1 | 1 |
| JUN | 201466_s_at | GSE6559 | 17440097 | 4.1 | NA | 1 | NA | 1 | 1 | 1 |
| KEAP1 | 202417_at | GSE66473 | 26078391 | 0.99 | NA | 4 | NA | 1 | NA | 2 |
| MTDH | 212248_at | GSE67249 | NA | 1.14 | NA | NA | NA | 1 | NA | NA |
| MFI2 | 220043_s_at | GSE6816 | 17449903 | 235 | 18 | 4 | 1 | 1 | 2 | 2 |

| | | | | | | | | | | |
|---|---|---|---|---|---|---|---|---|---|---|
| YAP1 | 213342_at | GSE69655 | 26173433 | 1.55 | NA | 2 | NA | 1 | NA | NA |
| TCF12 | 208986_at | GSE70186 | 27221705 | 1.19 | NA | 1 | NA | 1 | NA | 1 |
| RBM14 | 204178_s_at | GSE70540 | NA | 0.93 | NA | 2 | NA | 1 | NA | 4 |
| RBM14 | 204178_s_at | GSE70540 | NA | 0.82 | NA | 2 | NA | 1 | NA | 4 |
| SMC1A | 217555_at | GSE73224 | 26607380 | 1.26 | NA | 2 | NA | 1 | NA | 4 |
| LDB1 | 203451_at | GSE7382 | 17452977 | 1.28 | NA | 1 | NA | 1 | 3 | NA |
| LMO4 | 209205_s_at | GSE7382 | 17452977 | 1.17 | NA | 1 | NA | 1 | 3 | NA |
| HOXA5 | 213844_at | GSE74862 | 26678341 | 23.15 | NA | 2 | NA | 1 | NA | 4 |
| DKK1 | 204602_at | GSE8066 | 17643814 | 0.95 | NA | 4 | NA | 1 | NA | NA |
| REPIN1 | 219041_s_at | GSE83140 | NA | 0.9 | NA | 3 | NA | 1 | NA | NA |
| KHSRP | 204372_s_at | GSE83388 | NA | 1.08 | NA | NA | NA | 1 | NA | 5 |
| NCOR2 | 207760_s_at | GSE8346 | NA | 1 | NA | 1 | NA | 1 | NA | NA |
| XBP1 | 200670_at | GSE8562 | 17660348 | 0.86 | NA | 4 | 1 | 1 | 1 | 2 |
| TP53 | 201746_at | GSE8660 | 18267967 | 0.83 | NA | 3 | 1 | 3 | 6 | NA |
| TGFBR1 | 206943_at | GSE9093 | 18316594 | 2.49 | NA | 1 | NA | 1 | 1 | 1 |
| HOXC6 | 206858_s_at | GSE9182 | NA | 120.41 | NA | NA | NA | 1 | NA | NA |
| PAX9 | 207059_at | GSE9212 | 19279207 | 2.77 | NA | 1 | NA | NA | NA | 5 |
| TTF1 | 204772_s_at | GSE9212 | 19279207 | 1.21 | NA | 1 | NA | NA | NA | 5 |
| NKX2-8 | 207451_at | GSE9212 | 19279207 | 1.04 | NA | 1 | NA | NA | NA | 5 |
| MSX1 | 205932_s_at | GSE9339 | 18201699 | 0.68 | NA | 4 | NA | NA | 2 | 5 |
| HIC1 | 208461_at | GSE9854 | NA | 1960.12 | NA | 3 | NA | NA | NA | NA |
| ESR2 | 211120_x_at | GSE9936 | 18258689 | 966.77 | 10 | 3 | NA | 1 | NA | NA |

**Supplemental Figure 1**: The heat map of **Figure 2** at full resolution.

| gene | AffyID | GSE | line | 209173_at | 208461_at | 201005_at | 211120_x_at | 211485_s_at | 202431_s_at | 212764_at |
|---|---|---|---|---|---|---|---|---|---|---|
| AGR2 | 209173_at | GSE12513 | MIAPACA2 | 4612.35 | 0.43 | 1.95 | 1.9 | 0.81 | 1.04 | 1.08 |
| HIC1 | 208461_at | GSE9854 | U2OS | 0.71 | 1960.12 | 1.03 | 2.78 | 0.81 | 0.41 | 1.43 |
| CD9 | 201005_at | GSE13210 | RAJI | 0.96 | 0.98 | 1066.88 | 1.92 | 0.34 | 1.13 | 6.18 |
| ESR2 | 211120_x_at | GSE9936 | MCF7 | 1.07 | 3.47 | 1.12 | 966.77 | 2.67 | 1.17 | 3 |
| FGF18 | 211485_s_at | GSE48567 | A224 | 1.03 | 1.5 | 3.11 | 0.83 | 786.95 | 1.22 | 1.07 |
| MYC | 202431_s_at | GSE31311 | B cells | 1.36 | 1.72 | 1.62 | 0.93 | 1.14 | 735.49 | 1.27 |
| ZEB1 | 212764_at | GSE55688 | HMEC | 0.36 | 1.5 | 0.62 | 0.93 | 1.5 | 0.85 | 705 |
| MECOM | 221884_at | GSE60100 | U937 | 0.33 | 1.97 | 0.87 | 0.62 | 0.69 | 1.08 | 0.67 |
| PRDM5 | 220792_at | GSE10580 | U2OS | 0.33 | 3.27 | 0.87 | 1.49 | 2.11 | 0.82 | 1.01 |
| AR | 211110_s_at | GSE15091 | PC3 | 0.88 | 1.17 | 0.74 | 6.09 | 2.53 | 1.95 | 1.49 |
| MFI2 | 220043_s_at | GSE6816 | SKNMC | 1 | 0.33 | 0.86 | 0.76 | 0.05 | 0.89 | 1.05 |
| CDH3 | 203256_at | GSE23360 | BLM | 0.73 | 2.93 | 1.09 | 1.4 | 1.1 | 0.97 | 1.06 |
| SFTPC | 38691_s_at | GSE2980 | HEK293 | 1.43 | 0.53 | 1.33 | 1.19 | 2.09 | 1.03 | 0.93 |
| CDHR2 | 220186_s_at | GSE10650 | HCT116 | 290.24 | 63.56 | 0.12 | 58.07 | 5.49 | 0.15 | 0.44 |
| LTF | 202018_s_at | GSE36972 | 58F | 0.97 | 1.14 | 0.99 | 0.38 | 1.46 | 1.71 | 1.36 |
| WNT1 | 208570_at | GSE26656 | A375 | 1.83 | 4.34 | 0.89 | 10.1 | 1.68 | 0.92 | 1.4 |
| ZBTB16 | 205883_at | GSE26272 | MSC | 1.03 | 1.27 | 0.93 | 2.05 | 1.61 | 0.83 | 1.01 |
| WNT1 | 208570_at | GSE26656 | M24 | 2.98 | 4.14 | 0.73 | 3.36 | 3.41 | 0.74 | 1.83 |
| TP73 | 220804_s_at | GSE13504 | SAOS2 | 1.2 | 0.21 | 2.76 | 1 | 1.5 | 0.22 | 0.65 |
| HOXC6 | 206858_s_at | GSE9182 | LNCAP | 1.36 | 16.06 | 0.25 | 1.58 | 1.57 | 0.74 | 5.88 |
| PRDM1 | 217192_s_at | GSE27670 | B cells | 0.98 | 2.25 | 0.17 | 9.56 | 2.27 | 0.49 | 0.71 |
| KLF9 | 203543_s_at | GSE11855 | HEC1A | 6.75 | 2.5 | 0.73 | 50.44 | 0.03 | 0.88 | 1.76 |
| NR4A2 | 216248_s_at | GSE58475 | HB1 | 1.17 | 0.49 | 1.34 | 0.85 | 1.76 | 1.41 | 0.87 |
| FOXC2 | 214520_at | GSE44335 | HMLER | 1.2 | 1.33 | 0.73 | 1.27 | 2.58 | 0.75 | 227.91 |
| CD44 | 212063_at | GSE63862 | HEK293 | 1.56 | 1.83 | 3.26 | 1.23 | 2.16 | 6.79 | 0.82 |
| CFAP45 | 220308_at | GSE27318 | NPC58F | 0.67 | 1.43 | 1.3 | 0.72 | 1.02 | 0.76 | 0.19 |
| CLDN1 | 218182_s_at | GSE10309 | CL15 | 15.19 | 0.33 | 1.05 | 2.05 | 1.47 | 0.76 | 0.45 |
| ESR2 | 211120_x_at | GSE11115 | U2OS | 0.33 | 2 | 0.82 | 33.54 | 0.98 | 1.03 | 1.1 |
| PRKAR2B | 203680_at | GSE12854 | OVCAR8 | 85.24 | 0.67 | 0.88 | 1.73 | 2.1 | 3.43 | 0.99 |
| HOXD13 | 207397_s_at | GSE46542 | HEK293 | 1.53 | 1.53 | 1 | 2.41 | 2.13 | 3.16 | 1.42 |

| | | | | | | | | | | |
|---|---|---|---|---|---|---|---|---|---|---|
| FGFR1 | 210973_s_at | GSE15811 | CD34+ cells | 1.21 | 1.57 | 1.22 | 0.73 | 0.91 | 1.37 | 0.71 |
| HOXA5 | 213844_at | GSE74862 | SW480 | 14.18 | 0.57 | 31.43 | 5.13 | 4.3 | 4.73 | 10.25 |
| NEUROG3 | 207965_at | GSE30802 | duct cells poo | 0.79 | 0.84 | 0.91 | 1.04 | 2.43 | 0.71 | 1.36 |
| HRAS | 212983_at | GSE12790 | MCF10A | 1.57 | 4.12 | 0.5 | 1.56 | 4.8 | 0.55 | 0.88 |
| SOX2 | 213721_at | GSE45391 | MKN28 | 1.33 | 1.93 | 0.2 | 0.2 | 0.63 | 1.69 | 0.7 |
| BRAF | 206044_s_at | GSE13827 | melanocytes | 1.25 | 2.8 | 0.74 | 1.11 | 8.7 | 1.53 | 10.98 |
| MITF | 207233_s_at | GSE38007 | mammary epi | 1.98 | 4.64 | 2.11 | 1.41 | 1 | 0.97 | 6.31 |
| EPCAM | 201839_s_at | GSE25743 | HS578T | 0.33 | 0.5 | 0.99 | 6.6 | 0.17 | 1.17 | 0.74 |
| HRAS | 212983_at | GSE32975 | HACAT | 1332.02 | 1.39 | 0.96 | 3.13 | 4.26 | 0.55 | 1.15 |
| SNAI1 | 219480_at | GSE16194 | NCIH1437 | 0.02 | 0.67 | 0.58 | 1.27 | 0.56 | 0.48 | 54.75 |
| CA9 | 205199_at | GSE20569 | C33A | 0.75 | 3.25 | 1.08 | 1.35 | 1.51 | 0.71 | 0.82 |
| DPP3 | 218567_x_at | GSE6451 | IMR32 | 0.67 | 3.75 | 1.16 | 1.23 | 1.97 | 0.47 | 1.1 |
| HRAS | 212983_at | GSE6559 | keratinocytes | 2.15 | 0.75 | 0.9 | 0.22 | 0.74 | 0.44 | 3.88 |
| SLAMF7 | 219159_s_at | GSE14680 | U266 | 1.31 | 0.53 | 1.46 | 2.55 | 2.06 | 2.84 | 1.57 |
| MAP2K7 | 209952_s_at | GSE6559 | keratinocytes | 2.27 | 1 | 0.88 | 0.22 | 1.04 | 0.6 | 2.67 |
| USP2 | 207213_s_at | GSE17466 | IPREC | 23.09 | 2.77 | 1.37 | 3.12 | 1.41 | 1.23 | 7 |
| NFKBIA | 201502_s_at | GSE44619 | HCC827 | 1.52 | 1 | 1.09 | 1.9 | 2 | 0.86 | 7.33 |
| TAGLN | 205547_s_at | GSE48998 | RKO | 0.82 | 5.83 | 1.08 | 1.46 | 1.08 | 1.38 | 0.78 |
| FTO | 209702_at | GSE33870 | HEK293 | 1.05 | 0.67 | 0.97 | 3.19 | 0.62 | 0.9 | 1.02 |
| MYC | 202431_s_at | GSE22139 | DAOY | 2.08 | 2.44 | 2.07 | 1.22 | 4.94 | 6.01 | 2.06 |
| NFKBIA | 201502_s_at | GSE44619 | PC9 | 0.77 | 2 | 1.02 | 0.29 | 0.72 | 0.74 | 0.87 |
| MYC | 202431_s_at | GSE11791 | MCF7 | 0.72 | 2.22 | 0.83 | 4.75 | 2.39 | 5.45 | 8.5 |
| CXCR4 | 217028_at | GSE15893 | MDAMB231 | 0.33 | 0.68 | 0.84 | 5.99 | 1.56 | 1.2 | 1 |
| PRDM1 | 217192_s_at | GSE53283 | H9 | 1.65 | 2.31 | 0.78 | 2.04 | 12.14 | 0.64 | 3.1 |
| HRAS | 212983_at | GSE45276 | IMR90 | 11.01 | 0.53 | 2.36 | 3.73 | 1.45 | 1.96 | 0.86 |
| WNT4 | 208606_s_at | GSE39949 | endometrial s | 0.4 | 2.76 | 1.32 | 1 | 0.5 | 0.62 | 0.83 |
| HOXA1 | 214639_s_at | GSE37136 | WM115 | 0.78 | 0.71 | 2.13 | 1.89 | 29.66 | 0.59 | 0.78 |
| ZFP36 | 201531_at | GSE53183 | HEK293 | 1.18 | 1.13 | 1.37 | 0.9 | 1.81 | 0.5 | 1.45 |
| NFKBIA | 201502_s_at | GSE44619 | H358 | 1.09 | 1 | 1.1 | 1.88 | 1.4 | 0.82 | 0.91 |
| JUN | 201466_s_at | GSE6559 | keratinocytes | 1.34 | 0.62 | 0.82 | 0.3 | 1.17 | 0.46 | 3.45 |
| SOX17 | 219993_at | GSE10809 | CA2 | 0.87 | 1.31 | 0.69 | 0.16 | 1.04 | 0.67 | 1.8 |
| ID3 | 207826_s_at | GSE64535 | A431 | 1.04 | 1.25 | 1.8 | 0.31 | 2.77 | 1.04 | 3.01 |
| NOTCH3 | 203238_s_at | GSE16254 | IMR32 | 3.03 | 2 | 1.48 | 0.73 | 1.01 | 142.34 | 0.8 |

| | | | | | | | | | | |
|---|---|---|---|---|---|---|---|---|---|---|
| ARHGDIB | 201288_at | GSE35014 | UMUC3 | 0.92 | 0.42 | 1.22 | 0.75 | 0.94 | 0.91 | 1.17 |
| FLI1 | 204236_at | GSE31215 | MSC | 1.32 | 1.75 | 0.85 | 2.1 | 4.9 | 1.85 | 0.65 |
| IRF5 | 205469_s_at | GSE51717 | REH | 0.53 | 1.25 | 1.08 | 0.83 | 0.84 | 1 | 1.62 |
| PAX9 | 207059_at | GSE9212 | BEAS2B | 127.64 | 1.21 | 1.3 | 3.25 | 2.15 | 1.37 | 5.1 |
| RALA | 214435_x_at | GSE19295 | HEKHT | 4.36 | 1.12 | 0.85 | 1.91 | 1.53 | 1.04 | 1.37 |
| ITGB4 | 204990_s_at | GSE11466 | MDAMB435 | 0.53 | 6.21 | 1.02 | 4.6 | 0.48 | 1.04 | 1.76 |
| TGFBR1 | 206943_at | GSE9093 | MCF7 | 0.18 | 0.78 | 0.74 | 1.64 | 2.38 | 1.83 | 0.9 |
| MAP2K5 | 211370_s_at | GSE17939 | HUVEC | 1.44 | 0.5 | 1.18 | 1.94 | 6.75 | 1.1 | 0.99 |
| NR0B1 | 206645_s_at | GSE1318 | MCF7 | 0.55 | 0.71 | 0.8 | 1.16 | 1.96 | 1.08 | 2.25 |
| SOX4 | 201417_at | GSE11914 | LNCAP | 0.69 | 1.76 | 0.71 | 1.49 | 1.48 | 0.8 | 1.93 |
| MYB | 204798_at | GSE2816 | monocytes | 2.93 | 1.94 | 2.19 | 5.33 | 0.44 | 2.8 | 0.81 |
| EHMT2 | 202326_at | GSE22810 | CL10 | 1.84 | 0.17 | 1.09 | 1.21 | 1.17 | 0.74 | 1.01 |
| SETD8 | 220200_s_at | GSE24170 | U2OS | 4.25 | 2.25 | 0.87 | 1.3 | 3.5 | 1.33 | 1.4 |
| NUMB | 207545_s_at | GSE29137 | MDAMB231 | 70.62 | 1.36 | 2.9 | 0.78 | 0.29 | 2 | 1.49 |
| PML | 211012_s_at | GSE48842 | K562 | 1.67 | 1 | 0.54 | 0.14 | 2.57 | 1 | 0.8 |
| ZC3HAV1 | 220104_at | GSE54342 | L02 | 0.09 | 0.06 | 43.1 | 0.05 | 0.1 | 52.03 | 15.91 |
| ZNF83 | 221645_s_at | GSE27444 | MCF7RES | 1.58 | 1.06 | 1.59 | 0.63 | 0.32 | 0.53 | 0.71 |
| BMP2 | 205289_at | GSE39949 | endometrial s | 0.6 | 0.03 | 0.67 | 0.29 | 0.4 | 0.13 | 0.85 |
| ERCC6 | 207347_at | GSE56049 | UVSS1KO | 1.67 | 3.77 | 0.58 | 1.78 | 2.76 | 1.22 | 1.85 |
| PRAME | 204086_at | GSE17100 | CD34+ cells | 2.77 | 10.36 | 1.36 | 3.14 | 2.38 | 1.02 | 1.47 |
| SMARCA4 | 213720_s_at | GSE10847 | NCIH522 | 398.85 | 0.66 | 2.96 | 0.95 | 10.41 | 0.33 | 0.24 |
| MAP2K5 | 211370_s_at | GSE25145 | endothelial ce | 1.4 | 1.44 | 1.18 | 1.85 | 1.15 | 1.17 | 1.26 |
| HRAS | 212983_at | GSE55688 | HMEC | 0.55 | 1.5 | 0.82 | 0.7 | 1.75 | 0.87 | 358.5 |
| E2F1 | 204947_at | GSE50051 | mammary epi | 2.62 | 0.48 | 1.03 | 1.19 | 6.19 | 0.51 | 1.48 |
| YAP1 | 213342_at | GSE69655 | HUCCT1 | 0.69 | 1.26 | 1.27 | 0.71 | 1.41 | 1.04 | 0.43 |
| NFKBIA | 201502_s_at | GSE44619 | A549 | 1.06 | 1 | 1.09 | 0.35 | 3.71 | 1.07 | 0.87 |
| HOXD9 | 205604_at | GSE46542 | HEK293 | 1.22 | 0.78 | 1.01 | 3.03 | 1.64 | 1.28 | 0.76 |
| NOTCH1 | 218902_at | GSE29959 | TALL1 | 0.27 | 0.68 | 2.1 | 2.89 | 0.39 | 1.31 | 0.95 |
| CDH1 | 201131_s_at | GSE55529 | MDAMB134 | 0.95 | 0.69 | 1.11 | 2.05 | 1.22 | 1.07 | 1.61 |
| MAP3K14 | 205192_at | GSE18047 | NCIH929 | 1.25 | 1.67 | 1.32 | 0.38 | 1.11 | 1.03 | 0.89 |
| KAT8 | 221820_s_at | GSE20193 | HEK293 | 0.2 | 3.33 | 1.22 | 0.77 | 3.78 | 0.83 | 0.97 |
| MYOD1 | 206657_s_at | GSE14825 | RD | 0.86 | 0.09 | 5.24 | 1.21 | 2.02 | 0.62 | 1.33 |
| IKBKB | 209341_s_at | GSE20115 | REH | 0.07 | 3 | 0.86 | 0.9 | 1.53 | 0.77 | 1.23 |

| | | | | | | | | | | |
|---|---|---|---|---|---|---|---|---|---|---|
| ESR1 | 205225_at | GSE22593 | MDAMB231 | 1.85 | 1.32 | 0.41 | 1.22 | 2.07 | 2.26 | 1.13 |
| FOXP3 | 221334_s_at | GSE37253 | JURKAT | 1.34 | 0.7 | 0.23 | 5.85 | 0.18 | 0.92 | 0.9 |
| IGF1R | 203628_at | GSE5225 | OCUBM | 2.59 | 0.79 | 0.86 | 0.97 | 1.47 | 1.59 | 1.74 |
| TCFL5 | 204849_at | GSE1318 | MCF7 | 0.78 | 0.52 | 0.78 | 0.38 | 1.65 | 0.6 | 0.22 |
| ITGB5 | 201124_at | GSE11466 | MDAMB435 | 1.58 | 0.67 | 1.18 | 1.07 | 0.52 | 1.17 | 1.57 |
| GLB1 | 201576_s_at | GSE11266 | MCF7 | 3.36 | 1.98 | 0.92 | 2.29 | 4.53 | 0.62 | 1.72 |
| CYR61 | 201289_at | GSE29384 | LN229 | 1.19 | 1.5 | 0.82 | 1.86 | 5.06 | 1.52 | 1.15 |
| HOXA5 | 213844_at | GSE50659 | CL15 | 7.46 | 0.06 | 0.85 | 0.84 | 1.8 | 0.71 | 1.18 |
| SNAI2 | 213139_at | GSE29672 | MCF7 | 0.45 | 4.52 | 0.54 | 1.73 | 2.12 | 1.65 | 8.41 |
| NFKB1 | 209239_at | GSE52707 | SKR6 | 1 | 1.06 | 1.08 | 0.51 | 2.4 | 1.39 | 1.63 |
| HSPA4 | 208815_x_at | GSE50547 | HEK293 | 0.39 | 0.82 | 0.96 | 0.56 | 3.3 | 0.65 | 0.96 |
| LDB1 | 203451_at | GSE7382 | MCF7 | 1.4 | 0.95 | 0.98 | 0.57 | 2.83 | 1.26 | 9.57 |
| RNF2 | 205215_at | GSE51928 | HMELBRAFV6 | 1.25 | 0.92 | 0.96 | 1.88 | 0.85 | 1.03 | 1.26 |
| MAP2K1 | 202670_at | GSE12790 | MCF10A | 1.05 | 1.94 | 0.7 | 0.74 | 2.38 | 0.84 | 0.99 |
| MSX1 | 205932_s_at | GSE16254 | IMR32 | 1.05 | 0.78 | 1.1 | 1 | 1.69 | 2.56 | 1.05 |
| SMC1A | 217555_at | GSE73224 | CD34+ cells | 1.85 | 1.35 | 1.51 | 4.5 | 2.93 | 1.01 | 2.63 |
| NOTCH1 | 218902_at | GSE29959 | HPBALL | 0.65 | 3 | 0.88 | 0.79 | 0.73 | 1.32 | 0.96 |
| TTF1 | 204772_s_at | GSE9212 | BEAS2B | 90.85 | 1.01 | 1.29 | 2.35 | 0.97 | 1.75 | 3.39 |
| IKBKB | 209341_s_at | GSE17129 | RAMOS | 0.75 | 3.33 | 0.87 | 1.15 | 4.08 | 0.97 | 0.87 |
| PURA | 204020_at | GSE14464 | PC3 | 1.51 | 3 | 0.99 | 1.65 | 0.52 | 0.81 | 2.31 |
| TCF12 | 208986_at | GSE70186 | HSC3 | 2.17 | 1 | 0.67 | 1.09 | 0.17 | 0.85 | 6.25 |
| NOTCH1 | 218902_at | GSE29959 | DND41 | 1.33 | 0.52 | 1.05 | 2.5 | 1.59 | 1.09 | 0.99 |
| LMO4 | 209205_s_at | GSE7382 | MCF7 | 1.9 | 1.32 | 1.17 | 0.94 | 0.86 | 1.21 | 1.58 |
| RAF1 | 201244_s_at | GSE29884 | MCF7 | 0.02 | 1 | 0.79 | 0.59 | 0.36 | 1.73 | 11.88 |
| TAL1 | 206283_s_at | GSE48557 | TF1 | 0.76 | 1.43 | 0.56 | 1.41 | 0.71 | 0.87 | 0.99 |
| BAG1 | 202387_at | GSE51524 | LNCAP | 10.32 | 0.71 | 0.95 | 3.28 | 1.19 | 1.76 | 2.39 |
| MTDH | 212248_at | GSE67249 | MCF7 | 0.96 | 1.5 | 0.99 | 0.33 | 0.32 | 1.23 | 0.45 |
| CAT | 201432_at | GSE63955 | HEK293 - TRE) | 0.92 | 1.5 | 0.97 | 3.83 | 0.25 | 2.06 | 1.35 |
| YAP1 | 213342_at | GSE13218 | MCF10A | 1.26 | 1.17 | 0.82 | 0.67 | 1.59 | 1.32 | 1.48 |
| NOTCH1 | 218902_at | GSE20285 | MCF10A | 0.95 | 0.73 | 1.11 | 1.15 | 2.05 | 0.9 | 1.44 |
| NOTCH1 | 218902_at | GSE29959 | ALLSIL | 2.23 | 1.34 | 0.43 | 0.7 | 0.6 | 1.04 | 0.92 |
| EWSR1 | 209214_s_at | GSE31215 | MSC | 1.32 | 1.75 | 0.85 | 2.1 | 4.9 | 1.85 | 0.65 |
| IKBKB | 209341_s_at | GSE44619 | HCC827 | 2.12 | 1 | 0.94 | 0.4 | 0.5 | 1.59 | 14.33 |

| | | | | | | | | | | |
|---|---|---|---|---|---|---|---|---|---|---|
| YWHAZ | 200639_s_at | GSE20318 | CL10 | 0.07 | 1.83 | 1.25 | 1.88 | 1.14 | 1.3 | 0.79 |
| NUMB | 207545_s_at | GSE29137 | MCF7 | 0.97 | 1.02 | 1.03 | 2.78 | 1.52 | 1.04 | 1.91 |
| CTNNB1 | 201533_at | GSE11916 | HUH7 | 1.35 | 1.25 | 1.11 | 2.87 | 4.15 | 1.12 | 0.79 |
| MAP3K14 | 205192_at | GSE18047 | SACHI | 2.47 | 1.33 | 1.05 | 6.7 | 3.33 | 1.08 | 1.02 |
| CDH13 | 204726_at | GSE65363 | U87 | 1.56 | 0.59 | 0.77 | 3.78 | 1.27 | 0.97 | 1 |
| KHSRP | 204372_s_at | GSE83388 | CL15 | 1 | 2 | 1 | 0.5 | 1.25 | 1.1 | 0.9 |
| MED1 | 203497_at | GSE41150 | LNCAP | 1.19 | 3 | 1.02 | 1.38 | 0.88 | 1.19 | 0.49 |
| JAG1 | 216268_s_at | GSE16547 | LEC | 1.46 | 0.27 | 1.01 | 0.51 | 0.59 | 0.84 | 0.94 |
| NFIC | 206929_s_at | GSE17636 | MDAMB436 | 1.05 | 1.97 | 0.47 | 7.61 | 3.45 | 0.65 | 0.79 |
| IKBKB | 209341_s_at | GSE51717 | REH | 0.6 | 0.75 | 0.75 | 0.42 | 0.64 | 0.84 | 1.13 |
| NOTCH1 | 218902_at | GSE29850 | HUVEC | 0.89 | 2.08 | 1.01 | 4.41 | 2.18 | 1.09 | 1.42 |
| STIP1 | 213330_s_at | GSE27654 | MDAH2774 | 0.5 | 1.7 | 1.29 | 0.89 | 2.35 | 1.15 | 0.88 |
| NKX2-8 | 207451_at | GSE9212 | BEAS2B | 117.76 | 0.78 | 0.95 | 2.37 | 0.84 | 1.6 | 4.1 |
| PIAS3 | 203035_s_at | GSE42979 | A549 | 4.48 | 1.46 | 1.01 | 3.41 | 0.46 | 0.93 | 1.18 |
| RUNX1 | 209360_s_at | GSE28317 | CD34+ cells | 41.99 | 0.43 | 13.04 | 0.5 | 1.43 | 0.41 | 0.44 |
| MKL1 | 212748_at | GSE13710 | HEL | 0.69 | 0.55 | 1.22 | 0.53 | 2.48 | 0.87 | 2.37 |
| PURA | 204020_at | GSE14464 | LNCAP | 1.59 | 1.23 | 1.01 | 1.75 | 3.41 | 0.92 | 1.88 |
| NOTCH1 | 218902_at | GSE12355 | EREB25 | 2.33 | 7.24 | 1.56 | 3.29 | 2.67 | 2.9 | 1.22 |
| CD99 | 201029_s_at | GSE61928 | SAOS2 | 1.42 | 1.1 | 0.89 | 1.16 | 2.51 | 1.03 | 0.86 |
| YAP1 | 213342_at | GSE61764 | HUCCT1 | 0.93 | 1.34 | 1.21 | 1.51 | 1.22 | 0.77 | 1.52 |
| TP53 | 201746_at | GSE64738 | MES7 | 1.99 | 1.83 | 0.7 | 1.44 | 0.38 | 0.49 | 0.96 |
| RELA | 201783_s_at | GSE32975 | HACAT | 2.52 | 1.58 | 0.71 | 6.15 | 4.61 | 0.46 | 1.46 |
| NUP98 | 203195_s_at | GSE57194 | CD34+ cells | 3.19 | 5.4 | 0.91 | 3.52 | 11.97 | 0.85 | 1.11 |
| RCAN1 | 208370_s_at | GSE48841 | endothelial ce | 2.83 | 1.32 | 0.98 | 3.42 | 1.35 | 0.68 | 0.99 |
| TP53 | 201746_at | GSE64738 | MES1 | 4 | 2.34 | 0.81 | 0.61 | 0.95 | 0.94 | 0.81 |
| NCOR2 | 207760_s_at | GSE8346 | HMT3522 | 4 | 2.29 | 1.05 | 1.56 | 1.5 | 0.89 | 1.74 |
| H2AFY | 207168_s_at | GSE53103 | HELA | 5.61 | 1.11 | 1.31 | 0.46 | 1.32 | 0.88 | 0.79 |
| CDH1 | 201131_s_at | GSE55529 | IPH926 | 1.09 | 1.09 | 0.73 | 0.91 | 0.41 | 1.4 | 1.05 |
| PNP | 201695_s_at | GSE53604 | HELA | 1.17 | 0.89 | 1.05 | 3.28 | 1.35 | 0.67 | 1.04 |
| KEAP1 | 202417_at | GSE66473 | CL15 | 0.52 | 0.31 | 1.02 | 0.75 | 1.17 | 0.93 | 0.85 |
| RUNX1 | 209360_s_at | GSE57194 | CD34+ cells | 1.77 | 1.72 | 1.04 | 9.83 | 4.08 | 0.72 | 0.72 |
| AR | 211110_s_at | GSE44924 | LNCAP | 3.33 | 1.48 | 1.14 | 2.92 | 1.17 | 1.01 | 2.39 |
| ARL2BP | 202092_s_at | GSE58224 | BL2 | 0.9 | 1.41 | 1.09 | 1.14 | 3.07 | 0.94 | 1.01 |

| | | | | | | | | | | |
|---|---|---|---|---|---|---|---|---|---|---|
| MYB | 204798_at | GSE1318 | MCF7 | 0.67 | 0.26 | 1 | 0.67 | 1.42 | 0.81 | 0.52 |
| PML | 211012_s_at | GSE57194 | CD34+ cells | 4.49 | 1.66 | 1.07 | 4.4 | 2.68 | 0.89 | 0.89 |
| MYC | 202431_s_at | GSE34055 | HMEC | 0.88 | 0.83 | 0.89 | 0.57 | 2.57 | 0.96 | 1.02 |
| WHSC1 | 209054_s_at | GSE50072 | KMS11 | 1.94 | 1.98 | 0.45 | 1.77 | 1.39 | 0.77 | 1.25 |
| PA2G4 | 214794_at | GSE12200 | C81 | 0 | 7 | 0.75 | 0.28 | 0.89 | 0.68 | 3.66 |
| OLR1 | 210004_at | GSE13139 | HAECT | 1.58 | 1.15 | 1.04 | 1.17 | 1.81 | 0.92 | 0.99 |
| ZMYM2 | 202778_s_at | GSE15811 | CD34+ cells | 0.78 | 0.63 | 1.64 | 0.91 | 1.24 | 1.4 | 0.62 |
| DKK1 | 204602_at | GSE8066 | IMR32 | 0.73 | 1.52 | 0.96 | 0.57 | 0.67 | 2.13 | 1.17 |
| IKBKB | 209341_s_at | GSE18047 | NCIH929 | 0.94 | 1 | 0.79 | 1.52 | 1.11 | 0.71 | 1.1 |
| ABL1 | 202123_s_at | GSE15811 | CD34+ cells | 0.62 | 0.87 | 1.15 | 1.36 | 1.5 | 1.63 | 0.55 |
| DLC1 | 210762_s_at | GSE46214 | SCP2 | 0.08 | 0.5 | 0.86 | 11 | 0.19 | 0.9 | 1.31 |
| H2AFY | 207168_s_at | GSE53103 | NAMALWA | 1.53 | 1.78 | 0.84 | 5.1 | 0.17 | 0.93 | 0.89 |
| RBM14 | 204178_s_at | GSE70540 | A549 | 0.77 | 0.38 | 1 | 4.35 | 2.44 | 0.98 | 1.06 |
| MYC | 202431_s_at | GSE29959 | KOPTK1 | 1.77 | 7.3 | 3.4 | 1.51 | 2.38 | 0.92 | 0.95 |
| IKBKB | 209341_s_at | GSE18047 | SACHI | 0.58 | 1 | 1.22 | 5.4 | 4.69 | 0.97 | 1.06 |
| ELK1 | 203617_x_at | GSE48792 | CSES7 | 0.75 | 0.5 | 0.88 | 2.28 | 1.17 | 1.52 | 1.02 |
| CAT | 201432_at | GSE12355 | EREB25 | 0.82 | 1.85 | 1.54 | 1.07 | 2.82 | 0.86 | 0.99 |
| PEBP1 | 210825_s_at | GSE47378 | MDAMB231-1 | 0.51 | 0.34 | 0.92 | 6.9 | 0.47 | 0.97 | 0.91 |
| FGFR3 | 204379_s_at | GSE61352 | urothelial cell | 2.06 | 1.33 | 0.71 | 2.73 | 0.84 | 1.24 | 3.72 |
| IL6 | 205207_at | GSE11883 | MZCHA1 | 0.49 | 2.5 | 0.88 | 1.29 | 1.17 | 0.99 | 1.17 |
| REPIN1 | 219041_s_at | GSE83140 | PC3 | 0.66 | 3 | 1.07 | 0.68 | 1.05 | 1.01 | 0.82 |
| PRKAR1A | 200603_at | GSE12854 | OVCAR8 | 8.33 | 0.58 | 1.62 | 0.93 | 1.53 | 1.33 | 0.03 |
| NOTCH1 | 218902_at | GSE29959 | KOPTK1 | 0.97 | 2.1 | 4.1 | 0.7 | 2.45 | 1.34 | 0.96 |
| MYB | 204798_at | GSE2815 | MCF7 | 0.97 | 0.91 | 0.88 | 0.7 | 3.19 | 0.89 | 1.08 |
| RECK | 205407_at | GSE56898 | MDAMB231 | 2.28 | 1 | 1 | 0.53 | 1.4 | 1.27 | 1.05 |
| BRCA1 | 204531_s_at | GSE30296 | SKOV3 | 332.69 | 0.97 | 1.04 | 0.7 | 1.92 | 0.27 | 1.49 |
| IKBKB | 209341_s_at | GSE44619 | H23 | 2 | 1 | 1.05 | 0.84 | 0.63 | 0.84 | 0.96 |
| RAD21 | 200608_s_at | GSE47641 | KASUMI1 | 3 | 1.5 | 1.83 | 1.38 | 1.81 | 0.93 | 0.81 |
| ARL2BP | 202092_s_at | GSE58224 | BJAB | 2.27 | 1.46 | 1.07 | 0.97 | 0.8 | 0.93 | 1 |
| MYC | 202431_s_at | GSE29959 | HPBALL | 0.75 | 1.67 | 1.96 | 0.88 | 2.68 | 0.86 | 0.97 |
| XBP1 | 200670_at | GSE8562 | MCF7 | 2.91 | 1.07 | 1.71 | 2.17 | 1.15 | 1.49 | 10.63 |
| IKBKB | 209341_s_at | GSE44619 | PC9 | 0.38 | 1 | 1.03 | 0.18 | 0.25 | 0.88 | 1 |
| PPARGC1A | 219195_at | GSE19643 | MSC | 0.3 | 2.08 | 0.41 | 3.79 | 1.29 | 0.75 | 0.81 |

| | | | | | | | | | | |
|---|---|---|---|---|---|---|---|---|---|---|
| TP53 | 201746_at | GSE8660 | NCIH1299 | 0.7 | 3.5 | 1.12 | 2.08 | 0.8 | 0.74 | 0.76 |
| KDM8 | 220070_at | GSE56908 | LNCAP | 0.48 | 1 | 0.88 | 5.67 | 0.78 | 1.34 | 2.5 |
| EGFR | 201984_s_at | GSE32975 | HACAT | 0.76 | 1.41 | 0.88 | 2.81 | 1.99 | 0.78 | 1.05 |
| ZNF217 | 203739_at | GSE35511 | MDAMB231 | 8.5 | 0.98 | 0.98 | 1.26 | 0.69 | 0.87 | 0.98 |
| NOTCH2 | 212377_s_at | GSE12355 | EREB25 | 0.95 | 9.26 | 2.87 | 1.13 | 1.25 | 0.93 | 1.33 |
| RBM14 | 204178_s_at | GSE70540 | LOVO | 1.02 | 1.33 | 0.99 | 1.06 | 3.67 | 0.95 | 0.98 |
| YAP1 | 213342_at | GSE10196 | MCF10A | 2.06 | 1.33 | 1.54 | 0.3 | 3.31 | 0.8 | 3.6 |
| MYC | 202431_s_at | GSE29959 | DND41 | 1.75 | 0.52 | 1.14 | 1.59 | 0.98 | 0.78 | 0.85 |
| MYBL1 | 213906_at | GSE1318 | MCF7 | 0.71 | 0.46 | 0.82 | 0.99 | 0.91 | 0.83 | 0.21 |
| RASSF1 | 204346_s_at | GSE24473 | T47D | 0.96 | 3.06 | 0.69 | 0.77 | 2.75 | 0.41 | 0.83 |
| IKBKB | 209341_s_at | GSE44619 | A549 | 1.1 | 1 | 1.34 | 0.3 | 2.86 | 1.1 | 0.78 |
| NFKBIA | 201502_s_at | GSE44619 | H23 | 1.5 | 0.5 | 1.16 | 0.88 | 0.71 | 0.89 | 0.89 |
| IKBKB | 209341_s_at | GSE44619 | H358 | 0.28 | 1 | 0.46 | 1.12 | 1 | 1.17 | 3.98 |
| MYC | 202431_s_at | GSE29959 | ALLSIL | 0.55 | 4.75 | 0.86 | 0.81 | 1.28 | 0.75 | 0.87 |
| RB1 | 203132_at | GSE29783 | H9 | 1.12 | 2.62 | 0.99 | 5.97 | 1.07 | 0.81 | 1.49 |
| AKT1 | 207163_s_at | GSE45276 | IMR90 | 0.97 | 0.63 | 0.97 | 2.41 | 0.91 | 0.44 | 1.25 |
| RECK | 205407_at | GSE56898 | LM24175 | 0.91 | 0.5 | 0.71 | 0.39 | 1.15 | 0.98 | 1.23 |
| MYC | 202431_s_at | GSE29959 | TALL1 | 0.78 | 2 | 2.02 | 1.29 | 0.85 | 0.68 | 0.8 |
| MSX1 | 205932_s_at | GSE9339 | SJNB8 | 2.25 | 1.42 | 0.93 | 4.33 | 2.76 | 42.28 | 0.49 |
| YWHAZ | 200639_s_at | GSE52032 | MCF10A | 0.31 | 0.7 | 0.36 | 2.31 | 1.02 | 0.35 | 24.5 |
| NDRG1 | 200632_s_at | GSE25086 | MIAPACA2 | 2.09 | 2.1 | 0.83 | 0.6 | 1.67 | 1.71 | 1.69 |
| BCR | 202315_s_at | GSE15811 | CD34+ cells | 1.13 | 1.69 | 0.97 | 0.96 | 1.04 | 1.49 | 0.68 |
| ERBB2 | 216836_s_at | GSE14987 | MCF10A | 1.83 | 1.85 | 0.47 | 1.79 | 5.29 | 1.32 | 0.52 |
| PIK3CA | 204369_at | GSE17785 | MCF10A | 0.7 | 1.19 | 0.48 | 1.34 | 0.96 | 1.93 | 2.84 |
| GRHL2 | 219388_at | GSE44808 | human broncl | 1.02 | 1.84 | 0.84 | 1.09 | 1.26 | 0.8 | 1.82 |
| SDCBP | 200958_s_at | GSE57760 | FADU | 0.7 | 1.03 | 1.1 | 2.21 | 0.6 | 0.72 | 1.38 |
| RUNX1 | 209360_s_at | GSE10520 | U937 | 0.49 | 0.5 | 1.27 | 0.56 | 0.8 | 1.1 | 1.08 |
| SNAI2 | 213139_at | GSE55269 | keratinocytes | 1.74 | 2.5 | 0.54 | 4.64 | 0.35 | 0.68 | 11.15 |
| TP63 | 209863_s_at | GSE20286 | MCF10A | 6.11 | 0.89 | 2.21 | 3.24 | 1.41 | 0.72 | 0.95 |
| TP53 | 201746_at | GSE21105 | DLD1 | 0.99 | 0.18 | 0.57 | 1.33 | 6.83 | 0.27 | 6.25 |
| BCL11B | 219528_s_at | GSE21382 | JURKAT | 0.4 | 1 | 1.24 | 0.06 | 4.6 | 0.64 | 0.93 |
| ZBTB16 | 205883_at | GSE18476 | U937 | 1.01 | 0.83 | 4.72 | 0.51 | 1.49 | 0.11 | 0.97 |
| TGFBR2 | 208944_at | GSE10847 | NCIH522 | 46.24 | 0.44 | 1.66 | 0.27 | 6.83 | 1.44 | 0.73 |

| | | | | | | | | | | |
|---|---|---|---|---|---|---|---|---|---|---|
| HSF1 | 202344_at | GSE19797 | HELA | 0.64 | 1 | 1.16 | 1.11 | 2.73 | 0.86 | 1.04 |
| PRSS2 | 205402_x_at | GSE32056 | HSC3 | 0.78 | 1.3 | 1.05 | 0.5 | 2.19 | 1.17 | 3.21 |
| SOX2 | 213721_at | GSE11330 | MCF7 | 1.81 | 0.83 | 0.45 | 2.5 | 0.36 | 1.02 | 0.77 |
| FOXO1 | 202724_s_at | GSE40543 | human skelet | 0.9 | 1.15 | 3.39 | 6.25 | 0.62 | 0.45 | 0.58 |
| GATA3 | 209602_s_at | GSE24249 | MDAMB231 | 0.31 | 0.28 | 1.48 | 4.49 | 1.31 | 0.82 | 0.33 |
| HLX | 214438_at | GSE13054 | HUVEC | 0.74 | 8.64 | 1.04 | 1.44 | 0.65 | 1.32 | 1.11 |
| XIAP | 206536_s_at | GSE11618 | HCT116 | 1.98 | 1.39 | 1.07 | 3.23 | 2.66 | 0.89 | 2.17 |
| SMAD7 | 204790_at | GSE18070 | MCF10A | 4 | 1.36 | 1.28 | 3.66 | 2.25 | 1.09 | 0.52 |
| GRHL2 | 219388_at | GSE44807 | human broncl | 1.2 | 1.25 | 0.74 | 0.85 | 0.58 | 0.78 | 4.49 |
| PPARGC1A | 219195_at | GSE36879 | A375P | 1.31 | 0.77 | 1.12 | 1.9 | 0.33 | 0.57 | 2.31 |
| BHLHE41 | 221530_s_at | GSE33950 | MDAMB231 | 0.13 | 1.09 | 1.07 | 1.57 | 0.73 | 0.87 | 1.1 |
| MEX3D | 91816_f_at | GSE12239 | HEK293 | 1.04 | 0.15 | 35.37 | 2.12 | 0.53 | 14.57 | 29.82 |
| BCL3 | 204908_s_at | GSE60551 | HACAT | 2.77 | 0.81 | 0.68 | 0.51 | 3.25 | 0.89 | 3.24 |
| EHMT2 | 202326_at | GSE22810 | CL15 | 161.25 | 0.03 | 1.07 | 1.35 | 1.75 | 0.76 | 0.64 |

| 221884_at | 220792_at | 211110_s_at | 220043_s_at | 203256_at | 38691_s_at | 220186_s_at | 202018_s_at | 208570_at | 205883_at | 208570_at |
|---|---|---|---|---|---|---|---|---|---|---|
| 7.74 | 0.86 | 0.89 | 1.15 | 2.81 | 1.58 | 1.31 | 1.3 | 2.86 | 5.05 | 2.86 |
| 1.47 | 0.88 | 0.76 | 0.89 | 1.09 | 1.62 | 1.09 | 0.9 | 0.88 | 0.92 | 0.88 |
| 3.73 | 1.23 | 1.68 | 1.27 | 1.37 | 0.94 | 0.73 | 0.81 | 3.33 | 1.08 | 3.33 |
| 1.17 | 1.77 | 1.02 | 2.63 | 1.34 | 2.08 | 1.08 | 2.61 | 1.71 | 2.06 | 1.71 |
| 0.94 | 1.52 | 1.54 | 1.17 | 1.64 | 1.57 | 0.88 | 1.09 | 1.1 | 3.76 | 1.1 |
| 4.63 | 1.39 | 1 | 8.08 | 1.24 | 1.33 | 3.43 | 1.4 | 3.19 | 1.39 | 3.19 |
| 0.4 | 0.92 | 1 | 0.5 | 0.36 | 0.47 | 1.38 | 1 | 2.33 | 1.47 | 2.33 |
| 521.37 | 0.75 | 0.96 | 5.74 | 0.37 | 1.16 | 0.61 | 0.62 | 0.79 | 0.72 | 0.79 |
| 0.92 | 293.65 | 0.66 | 0.91 | 1.61 | 1.48 | 0.89 | 1.97 | 2.79 | 0.66 | 2.79 |
| 0.65 | 0.99 | 268.62 | 0.87 | 1.13 | 2.32 | 0.67 | 1.33 | 16.15 | 7.57 | 16.15 |
| 0.55 | 0.71 | 0.87 | 235 | 6.67 | 0.88 | 0.73 | 0.43 | 0.29 | 0.48 | 0.29 |
| 1.2 | 0.79 | 0.22 | 0.59 | 205.96 | 1.55 | 0.82 | 0.57 | 2.67 | 0.67 | 2.67 |
| 1.04 | 0.79 | 1.02 | 1.08 | 0.14 | 173.51 | 1.09 | 1.16 | 3.26 | 1.22 | 3.26 |
| 30.24 | 7.09 | 10.4 | 9.17 | 0.01 | 32.84 | 169.81 | 8.3 | 67.31 | 59.78 | 67.31 |
| 1.52 | 1.4 | 0.7 | 1.43 | 0.6 | 1.51 | 0.86 | 169 | 3.38 | 2.4 | 3.38 |
| 1.24 | 1.99 | 1.12 | 0.53 | 3.82 | 0.98 | 1.8 | 0.45 | 156.88 | 0.94 | 156.88 |
| 2.18 | 0.74 | 1.14 | 2.85 | 5.2 | 2.85 | 0.63 | 11.56 | 1.67 | 153.83 | 1.67 |
| 0.61 | 3.05 | 1.52 | 0.61 | 2.47 | 0.65 | 1.1 | 1.42 | 138.69 | 7.42 | 138.69 |
| 7.8 | 0.97 | 0.82 | 1.31 | 3 | 1.29 | 0.44 | 1 | 3.17 | 0.55 | 3.17 |
| 2.62 | 3.45 | 1.2 | 1.66 | 1.42 | 2.55 | 1.72 | 3.13 | 1.33 | 1.19 | 1.33 |
| 4.33 | 0.19 | 1.67 | 2.17 | 1.28 | 1.62 | 1.86 | 0.95 | 0.51 | 1.62 | 0.51 |
| 1.72 | 13.53 | 10.97 | 20.39 | 2.57 | 6.84 | 5.08 | 11.44 | 7.07 | 8.55 | 7.07 |
| 1.24 | 2.74 | 1.5 | 8 | 24.24 | 0.71 | 0.47 | 1.58 | 3.25 | 0.28 | 3.25 |
| 0.14 | 0.91 | 1.42 | 0.98 | 0 | 0.69 | 1.77 | 0.69 | 3.25 | 1.95 | 3.25 |
| 0.16 | 0.69 | 0.42 | 6.94 | 0.04 | 1.56 | 0.8 | 0.73 | 1.67 | 0.17 | 1.67 |
| 0.53 | 0.92 | 0.87 | 1.72 | 0.94 | 1.14 | 0.9 | 0.56 | 1.4 | 1.43 | 1.4 |
| 1.09 | 1.09 | 1.14 | 1.18 | 0.62 | 1.58 | 0.46 | 1 | 0.23 | 0.25 | 0.23 |
| 0.88 | 2.44 | 3.7 | 1.47 | 5.63 | 2.31 | 2.77 | 2.52 | 3.32 | 18.34 | 3.32 |
| 90.42 | 1.41 | 1.32 | 3.89 | 13.38 | 1.04 | 1.12 | 0.82 | 0.91 | 1.13 | 0.91 |
| 1.23 | 1.28 | 1.11 | 1.4 | 1.4 | 1.39 | 0.98 | 1.07 | 1.04 | 1.45 | 1.04 |

| | | | | | | | | | | |
|---|---|---|---|---|---|---|---|---|---|---|
| 0.63 | 2.61 | 2 | 1.39 | 0.74 | 0.86 | 1.68 | 10.2 | 4.67 | 0.73 | 4.67 |
| 0.7 | 1.21 | 3.1 | 0.79 | 0.04 | 2.88 | 1.7 | 0.4 | 1.62 | 0.56 | 1.62 |
| 0.96 | 1.29 | 1.18 | 1.02 | 0.93 | 1.4 | 0.88 | 3.36 | 3.68 | 1.19 | 3.68 |
| 0.55 | 1.78 | 1.42 | 1.09 | 0.39 | 2.42 | 3.22 | 1.23 | 9.76 | 0.33 | 9.76 |
| 2.84 | 1.07 | 2.38 | 0.75 | 0.73 | 2.66 | 1.56 | 5.04 | 1 | 2.12 | 1 |
| 16.92 | 3.58 | 5.49 | 1.47 | 0.52 | 0.85 | 1.97 | 33.08 | 10.82 | 2.38 | 10.82 |
| 0.08 | 0.83 | 0.41 | 0.97 | 17.73 | 5.79 | 1.43 | 3.73 | 1.32 | 1.15 | 1.32 |
| 1.01 | 1.42 | 1.35 | 1 | 1.88 | 1.96 | 1.46 | 1.5 | 2 | 0.79 | 2 |
| 0.24 | 2.7 | 2.33 | 1.43 | 0.66 | 0.9 | 0.34 | 2.57 | 4.28 | 1.14 | 4.28 |
| 1.17 | 0.16 | 0.69 | 2.1 | 0.06 | 0.85 | 1.36 | 1.5 | 0.22 | 0.4 | 0.22 |
| 2.4 | 1.13 | 0.42 | 0.7 | 1.4 | 6.82 | 0.75 | 1.33 | 2.6 | 3.34 | 2.6 |
| 0.24 | 0.78 | 3.39 | 3.54 | 0.45 | 0.71 | 0.73 | 1.31 | 0.95 | 2.55 | 0.95 |
| 0.67 | 0.66 | 0.42 | 0.37 | 0.97 | 0.86 | 1.29 | 3 | 1.16 | 2.41 | 1.16 |
| 1.96 | 0.76 | 4.21 | 1.17 | 2.5 | 2.37 | 0.77 | 1.35 | 2.14 | 0.92 | 2.14 |
| 1.13 | 0.76 | 0.58 | 0.32 | 0.96 | 0.95 | 1.25 | 2.92 | 0.96 | 1.68 | 0.96 |
| 1.61 | 0.99 | 0.93 | 1.22 | 36.66 | 0.85 | 1.64 | 1.28 | 0.89 | 1.04 | 0.89 |
| 1.14 | 1.21 | 1.11 | 1 | 0.8 | 0.64 | 0.31 | 1 | 0.5 | 0.13 | 0.5 |
| 1.6 | 1.18 | 3.42 | 7.27 | 1.94 | 1.98 | 1.18 | 3.26 | 1.22 | 0.59 | 1.22 |
| 1 | 0.88 | 0.68 | 0.79 | 0.87 | 1.06 | 1.45 | 1.17 | 0.81 | 0.83 | 0.81 |
| 0.92 | 1.18 | 2.52 | 1.97 | 3.64 | 2.09 | 1.25 | 0.76 | 1.73 | 4.48 | 1.73 |
| 0.97 | 0.54 | 0.55 | 0.52 | 0.88 | 0.8 | 1.82 | 1 | 0.13 | 0.37 | 0.13 |
| 0.76 | 4.63 | 0.96 | 1.89 | 0.79 | 1.64 | 0.91 | 0.33 | 1.05 | 2.78 | 1.05 |
| 0.81 | 0.88 | 1.59 | 0.72 | 1.02 | 1.15 | 0.87 | 1.39 | 1.95 | 0.71 | 1.95 |
| 5.79 | 1.02 | 1.04 | 2.94 | 1.65 | 1.46 | 1.22 | 1.06 | 2.99 | 1.79 | 2.99 |
| 0.56 | 0.22 | 0.7 | 1.08 | 2.92 | 1.15 | 1.74 | 1.62 | 2.26 | 1.52 | 2.26 |
| 2.37 | 1.48 | 0.48 | 0.13 | 0.67 | 1.02 | 0.59 | 0.67 | 0.23 | 1.09 | 0.23 |
| 0.63 | 0.67 | 3.34 | 0.52 | 0.4 | 0.98 | 3.26 | 24.17 | 1.22 | 0.54 | 1.22 |
| 0.79 | 1.53 | 1.82 | 2.63 | 18.15 | 0.84 | 0.68 | 0.86 | 1.07 | 2.36 | 1.07 |
| 0.8 | 1.1 | 0.89 | 0.11 | 1.19 | 2.35 | 0.68 | 1.5 | 3 | 1.47 | 3 |
| 0.42 | 0.8 | 1.45 | 0.29 | 0.74 | 0.91 | 1.41 | 7.25 | 0.91 | 2 | 0.91 |
| 0.78 | 0.68 | 0.92 | 3.82 | 0.79 | 1.41 | 2.63 | 1.12 | 1.42 | 7.13 | 1.42 |
| 0.98 | 2.48 | 1.46 | 0.91 | 1.78 | 1.93 | 1.43 | 0.63 | 3.75 | 10 | 3.75 |
| 7.61 | 1.36 | 0.84 | 2.75 | 3.19 | 1.21 | 0.98 | 4.13 | 0.56 | 1.35 | 0.56 |

| | | | | | | | | | | |
|---|---|---|---|---|---|---|---|---|---|---|
| 0.93 | 0.85 | 1.46 | 0.85 | 1.44 | 2.02 | 1.03 | 0.83 | 7 | 0.36 | 7 |
| 1.97 | 0.74 | 0.88 | 0.6 | 1.35 | 1.57 | 1.51 | 2.5 | 2.05 | 8.62 | 2.05 |
| 1.09 | 0.75 | 5.07 | 3.12 | 9.93 | 1.05 | 1.11 | 1.25 | 0.3 | 1.77 | 0.3 |
| 5.49 | 1.53 | 1.33 | 0.97 | 5.56 | 1.9 | 1.29 | 1.51 | 2.65 | 1.45 | 2.65 |
| 1.26 | 1.14 | 1.49 | 1.47 | 0.43 | 2.02 | 1.83 | 1.71 | 1.68 | 3.79 | 1.68 |
| 1.07 | 0.69 | 1.41 | 1.58 | 1.12 | 1.62 | 1.62 | 0.85 | 0.06 | 1.22 | 0.06 |
| 0.02 | 1.42 | 2.01 | 1.88 | 2.17 | 2.83 | 0.9 | 0.96 | 1.9 | 4.62 | 1.9 |
| 0.85 | 0.91 | 1.22 | 3.43 | 0.84 | 1.44 | 1.74 | 14.06 | 4.12 | 1.39 | 4.12 |
| 1.02 | 1.35 | 0.97 | 2.16 | 0.86 | 0.85 | 0.9 | 1.16 | 2.06 | 0.27 | 2.06 |
| 1.83 | 1.61 | 0.9 | 0.44 | 1.13 | 1.78 | 0.86 | 1.96 | 0.6 | 0.82 | 0.6 |
| 8.17 | 1.17 | 3.59 | 3.29 | 1.87 | 0.95 | 1.7 | 2.64 | 1.87 | 2.01 | 1.87 |
| 1.1 | 0.56 | 12.2 | 2.71 | 0.71 | 0.34 | 1.26 | 1 | 1 | 0.79 | 1 |
| 0.94 | 0.88 | 1.44 | 1.58 | 5.5 | 1.18 | 0.76 | 22.5 | 1.5 | 0.88 | 1.5 |
| 1.05 | 1.1 | 2.24 | 0.74 | 1.74 | 1.7 | 1 | 1.05 | 2.19 | 2.66 | 2.19 |
| 1.41 | 0.51 | 4.8 | 1 | 4 | 3.48 | 0.46 | 0.5 | 2.5 | 1.1 | 2.5 |
| 0.62 | 0.07 | 0.02 | 0.2 | 0.05 | 0.06 | 0.05 | 0.04 | 0.36 | 0.3 | 0.36 |
| 1.55 | 0.89 | 1.8 | 2.17 | 1.4 | 1.35 | 1.23 | 1.16 | 0.35 | 0.92 | 0.35 |
| 9.53 | 0.68 | 0.24 | 0.02 | 1 | 1.02 | 0.46 | 1.67 | 0.12 | 0.2 | 0.12 |
| 3.87 | 4.7 | 1.59 | 0.9 | 0.6 | 0.9 | 0.88 | 1.09 | 4.94 | 1.05 | 4.94 |
| 7.09 | 1.65 | 12.73 | 1.82 | 1.93 | 1.5 | 0.79 | 2.12 | 2.1 | 2.01 | 2.1 |
| 0.19 | 2.6 | 1.41 | 8.34 | 614.06 | 1.78 | 1.13 | 0.78 | 0.3 | 4.91 | 0.3 |
| 0.92 | 1.12 | 0.58 | 1.05 | 1.21 | 0.94 | 1.31 | 75.6 | 1.03 | 0.88 | 1.03 |
| 0.5 | 0.82 | 1.02 | 1.65 | 0.56 | 0.43 | 2.02 | 1.25 | 1.83 | 0.96 | 1.83 |
| 0.63 | 0.96 | 2.08 | 1.79 | 0.81 | 1.58 | 1.02 | 1.3 | 2.5 | 11.36 | 2.5 |
| 0.8 | 2.74 | 2.03 | 1.93 | 1.14 | 0.84 | 0.9 | 2.77 | 1.1 | 1.44 | 1.1 |
| 0.59 | 0.7 | 0.94 | 1.28 | 0.83 | 0.8 | 0.78 | 1.5 | 1.54 | 0.91 | 1.54 |
| 0.84 | 0.62 | 0.78 | 3.7 | 1.61 | 1.15 | 0.95 | 2.62 | 2.88 | 0.92 | 2.88 |
| 2.53 | 1.16 | 3.32 | 1.14 | 1.04 | 0.87 | 1.05 | 1.5 | 1.29 | 1.63 | 1.29 |
| 0.94 | 1.33 | 0.96 | 1.28 | 0.96 | 1.43 | 1.06 | 1.5 | 1.91 | 0.8 | 1.91 |
| 4.03 | 1.13 | 3.24 | 2.18 | 0.6 | 0.94 | 0.8 | 0.78 | 0.83 | 1.12 | 0.83 |
| 1.01 | 1.01 | 0.91 | 1.02 | 3.02 | 0.6 | 3.19 | 0.89 | 1.07 | 0.83 | 1.07 |
| 2.22 | 1.29 | 1.46 | 1.25 | 1.63 | 0.89 | 1.26 | 2.29 | 2.82 | 0.55 | 2.82 |
| 1.2 | 3 | 5.3 | 0.23 | 0.83 | 1.88 | 2.55 | 8.62 | 0.34 | 3.72 | 0.34 |

| | | | | | | | | | | |
|---|---|---|---|---|---|---|---|---|---|---|
| 0.67 | 1.28 | 1.59 | 1.41 | 1.3 | 2.56 | 1.39 | 1.58 | 1.61 | 3.91 | 1.61 |
| 4.04 | 0.55 | 6.06 | 14.06 | 1.17 | 0.73 | 1.09 | 1.5 | 0.92 | 1.75 | 0.92 |
| 0.97 | 4.36 | 2.48 | 2.63 | 0.82 | 0.78 | 5.52 | 1.25 | 14.82 | 2.62 | 14.82 |
| 1.88 | 0.49 | 1.09 | 2.29 | 1.17 | 1.32 | 0.95 | 1.01 | 2.23 | 1.11 | 2.23 |
| 0.21 | 1.04 | 1.13 | 0.86 | 2.16 | 2.99 | 0.94 | 0.73 | 0.89 | 0.99 | 0.89 |
| 22.13 | 1.38 | 2.22 | 0.34 | 0.97 | 1.4 | 1.45 | 1.33 | 1.6 | 1.86 | 1.6 |
| 1.17 | 1.19 | 1.09 | 0.9 | 1.9 | 0.87 | 0.83 | 10.33 | 5.21 | 1 | 5.21 |
| 0.61 | 0.88 | 1.74 | 0.35 | 0.37 | 0.97 | 0.92 | 0.52 | 0.89 | 3.9 | 0.89 |
| 0.74 | 1.85 | 0.97 | 0.96 | 0.9 | 1.21 | 1.52 | 1.5 | 3.02 | 3.65 | 3.02 |
| 1.29 | 6.96 | 0.77 | 0.89 | 2 | 0.73 | 1.42 | 1.48 | 4.24 | 1.21 | 4.24 |
| 1.03 | 4.03 | 1.24 | 2.55 | 1.15 | 2.46 | 1.32 | 3.41 | 4 | 1.63 | 4 |
| 2.6 | 1.2 | 1.3 | 1.6 | 1.34 | 1.39 | 1.32 | 1.12 | 0.81 | 1.75 | 0.81 |
| 0.99 | 1.27 | 0.66 | 1.81 | 0.83 | 1.57 | 0.91 | 0.95 | 1.75 | 1.54 | 1.75 |
| 1 | 0.8 | 1.23 | 0.58 | 0.64 | 1.6 | 1.52 | 1.17 | 6.05 | 0.56 | 6.05 |
| 1.91 | 1.13 | 0.72 | 1.19 | 2.03 | 1.08 | 0.89 | 0.97 | 0.44 | 0.9 | 0.44 |
| 3.92 | 4.24 | 1.26 | 1.6 | 1.23 | 1.88 | 0.87 | 3.28 | 1.89 | 1.01 | 1.89 |
| 0.07 | 2.58 | 1.21 | 0.94 | 1.36 | 0.57 | 0.83 | 1.67 | 2.78 | 1.63 | 2.78 |
| 5.09 | 1.14 | 1.27 | 0.83 | 7.01 | 1.43 | 1.02 | 1.54 | 0.82 | 1.26 | 0.82 |
| 1.44 | 1.15 | 0.46 | 2.85 | 1.75 | 3.45 | 0.88 | 0.78 | 0.83 | 2.12 | 0.83 |
| 0.93 | 0.55 | 0.69 | 0.38 | 0.12 | 1.3 | 0.79 | 0.86 | 0.19 | 2.83 | 0.19 |
| 0.72 | 0.95 | 0.98 | 0.5 | 1.04 | 1.22 | 0.61 | 1.5 | 1 | 1.3 | 1 |
| 0.75 | 1.48 | 1.78 | 1.27 | 1.23 | 0.57 | 3.69 | 1.04 | 0.19 | 11.1 | 0.19 |
| 0.76 | 0.78 | 1.37 | 1.02 | 1.43 | 1 | 0.87 | 1.21 | 1.11 | 1.17 | 1.11 |
| 1.05 | 0.83 | 1.01 | 0.63 | 1.15 | 1.3 | 1.75 | 2 | 1.02 | 0.33 | 1.02 |
| 1.15 | 0.87 | 1.12 | 1.91 | 2.31 | 1.84 | 2.09 | 1.65 | 4.89 | 0.99 | 4.89 |
| 0.27 | 0.9 | 0.58 | 1.79 | 0.88 | 0.78 | 0.92 | 10.16 | 1.33 | 0.46 | 1.33 |
| 1.23 | 1.45 | 1.71 | 1.25 | 1.11 | 0.22 | 9.4 | 0.67 | 1.8 | 0.08 | 1.8 |
| 0.9 | 1.83 | 0.68 | 2.89 | 7.57 | 0.79 | 1.18 | 1.12 | 0.52 | 2.54 | 0.52 |
| 0.99 | 0.93 | 0.91 | 3.3 | 1.11 | 0.65 | 1.72 | 1.5 | 7.1 | 0.66 | 7.1 |
| 0.81 | 1.29 | 0.92 | 3.45 | 1.06 | 1.3 | 1.29 | 4.74 | 3.5 | 0.92 | 3.5 |
| 1.05 | 6.76 | 0.68 | 0.95 | 0.75 | 4.05 | 1.38 | 0.78 | 0.77 | 0.68 | 0.77 |
| 1.97 | 0.74 | 0.88 | 0.6 | 1.35 | 1.57 | 1.51 | 2.5 | 2.05 | 8.62 | 2.05 |
| 0.88 | 1.12 | 0.82 | 1.2 | 1 | 0.15 | 0.88 | 0.5 | 3 | 1.27 | 3 |

| | | | | | | | | | | |
|---|---|---|---|---|---|---|---|---|---|---|
| 0.92 | 1.14 | 0.85 | 1.89 | 0.95 | 1 | 1.52 | 0.54 | 0.99 | 1.73 | 0.99 |
| 1.41 | 1.54 | 0.95 | 1.15 | 1 | 0.95 | 4.06 | 1.21 | 2.49 | 0.6 | 2.49 |
| 1.8 | 6.24 | 1.03 | 0.86 | 2.68 | 1.38 | 1.34 | 0.96 | 6 | 0.95 | 6 |
| 1.49 | 0.78 | 2.86 | 0.66 | 1.02 | 1.05 | 0.81 | 0.4 | 2.19 | 0.72 | 2.19 |
| 1 | 1.02 | 1.03 | Inf | 66.3 | 1.79 | 0.94 | 1.7 | 0.8 | 1.33 | 0.8 |
| 1.18 | 1.02 | 0.43 | 2.75 | 1.25 | 2 | 0.65 | 1.31 | 1 | 0.12 | 1 |
| 5.06 | 1.09 | 0.95 | 0.88 | 1.71 | 1.65 | 0.97 | 0.97 | 0.36 | 0.79 | 0.36 |
| 0.81 | 0.81 | 1.01 | 1.11 | 0.5 | 2.65 | 0.78 | 1 | 3.73 | 0.93 | 3.73 |
| 1.03 | 0.73 | 1.31 | 2.12 | 1.78 | 0.82 | 0.6 | 1.22 | 0.67 | 13.98 | 0.67 |
| 1.35 | 1.24 | 3.27 | 0.64 | 0.73 | 1.54 | 1.23 | 0.55 | 0.47 | 0.75 | 0.47 |
| 0.77 | 2.39 | 0.38 | 1.04 | 2.87 | 1.44 | 1.52 | 1.14 | 3.09 | 0.94 | 3.09 |
| 0.52 | 0.57 | 0.57 | 0.4 | 0.9 | 1.46 | 3.63 | 1.24 | 0.84 | 3.01 | 0.84 |
| 5.22 | 1.22 | 1.05 | 0.96 | 5.67 | 2.6 | 1.38 | 2.43 | 1.49 | 1.77 | 1.49 |
| 1.96 | 1.17 | 1.42 | 0.8 | 0.33 | 0.6 | 0.77 | 1.38 | 1.17 | 1.25 | 1.17 |
| 0.64 | 1.25 | 1 | 1.01 | 0.51 | 0.79 | 1.43 | 35.42 | 1.42 | 0.2 | 1.42 |
| 1.07 | 1.38 | 1.08 | 1.12 | 0.98 | 1.49 | 2.26 | 1.62 | 6.16 | 1.19 | 6.16 |
| 4.35 | 0.71 | 1.03 | 0.87 | 1.07 | 1.72 | 1.13 | 1.52 | 1.88 | 1.13 | 1.88 |
| 2.67 | 1.89 | 1.48 | 2 | 0.74 | 1.71 | 1.16 | 0.83 | 1.78 | 0.89 | 1.78 |
| 0.77 | 1.37 | 1.16 | 1.64 | 0.66 | 0.77 | 0.95 | 3.46 | 1.14 | 0.8 | 1.14 |
| 1.17 | 2.04 | 2.13 | 3.75 | 1.17 | 1.44 | 2.74 | 3.4 | 3.33 | 1.11 | 3.33 |
| 0.53 | 0.75 | 3.33 | 0.45 | 2.87 | 1.08 | 1.29 | 0.78 | 3.25 | 0.83 | 3.25 |
| 0.92 | 10.74 | 1.77 | 2.59 | 1.14 | 0.87 | 1.92 | 2.51 | Inf | 1.16 | Inf |
| 2.46 | 1 | 1.84 | 1.46 | 1.45 | 1.01 | 1.13 | 3.79 | 2.64 | 0.98 | 2.64 |
| 1.46 | 1.16 | 0.81 | 2.97 | 1.08 | 0.84 | 0.73 | 0.65 | 1.3 | 1.1 | 1.3 |
| 1.01 | 1.01 | 1.01 | 0.53 | 6.08 | 3.1 | 1.58 | 28 | 1.22 | 2.43 | 1.22 |
| 1.4 | 1.26 | 1.99 | 1.37 | 1 | 1.51 | 2.21 | 1.47 | 1.43 | 0.79 | 1.43 |
| 1.11 | 0.77 | 0.92 | 0.53 | 3.58 | 0.99 | 0.95 | 0.89 | 2.59 | 0.85 | 2.59 |
| 0.98 | 1.48 | 1.24 | 1.41 | 0.79 | 1.14 | 1.9 | 0.87 | 2.67 | 1.29 | 2.67 |
| 1.01 | 1.01 | 1.25 | 1.99 | 1 | 1.16 | 0.68 | 1.56 | 3.67 | 1.38 | 3.67 |
| 0.53 | 1.11 | 2.04 | 0.84 | 8 | 1.26 | 1.29 | 1 | 0.75 | 1 | 0.75 |
| 1.26 | 1.53 | 1.35 | 2.06 | 0.92 | 1.16 | 1.77 | 2.42 | 2.09 | 1.07 | 2.09 |
| 0.48 | 2 | 0.98 | 0.71 | 0.94 | 1.23 | 1.41 | 0.7 | 1.54 | 2.25 | 1.54 |
| 2.17 | 0.55 | 0.82 | 0.16 | 0.81 | 0.83 | 0.7 | 0.76 | 0.92 | 1.03 | 0.92 |

| | | | | | | | | | | |
|---|---|---|---|---|---|---|---|---|---|---|
| 1.62 | 0.42 | 0.6 | 1.11 | 1.88 | 0.61 | 0.61 | 0.56 | 1.93 | 0.79 | 1.93 |
| 1.23 | 1.02 | 2.08 | 0.76 | 1.57 | 0.82 | 0.92 | 3.83 | 2.12 | 1.15 | 2.12 |
| 1.11 | 1 | 0.81 | 0.84 | 0.95 | 1 | 0.76 | 1.02 | 1.17 | 0.87 | 1.17 |
| 0.87 | 1.6 | 1.24 | 1.81 | 1.48 | 1.16 | 0.55 | 1.36 | 0.9 | 1.81 | 0.9 |
| 1.07 | 1.59 | 0.6 | 3.33 | 0.61 | 0.85 | 1.47 | 0.82 | 0.18 | 0.51 | 0.18 |
| 0.86 | 1.01 | 1.39 | 0.96 | 1.45 | 2.02 | 0.93 | 1.05 | 1.95 | 0.94 | 1.95 |
| 0.63 | 2.76 | 1.82 | 1.89 | 0.92 | 1.04 | 1.79 | 8.88 | 6 | 0.78 | 6 |
| 1.93 | 1.4 | 0.61 | 0.8 | 1.85 | 1.03 | 0.88 | 0.67 | 0.19 | 0.83 | 0.19 |
| 1.41 | 1 | 5.77 | 5.22 | 0.96 | 1.15 | 0.89 | 0.78 | 0.4 | 2.1 | 0.4 |
| 0.83 | 2.58 | 1.53 | 0.94 | 1.53 | 0.85 | 2.51 | 6.99 | 1.33 | 0.97 | 1.33 |
| 1.99 | 1.56 | 0.11 | 1.55 | 0.22 | 1.66 | 0.91 | 1 | 1 | 0.91 | 1 |
| 6.72 | 0.69 | 0.67 | 5.63 | 1.12 | 0.67 | 0.57 | 0.92 | 2.78 | 3.64 | 2.78 |
| 1 | 0.9 | 0.68 | 1.28 | 1.78 | 0.78 | 0.95 | 1.17 | 2.08 | 2.1 | 2.08 |
| 3.11 | 0.75 | 0.56 | 0.22 | 1.38 | 2.7 | 0.48 | 1 | 4.01 | 1.37 | 4.01 |
| 0.88 | 0.97 | 4.2 | 0.8 | 2.48 | 1.23 | 0.73 | 1.6 | 0.71 | 0.99 | 0.71 |
| 0.87 | 1.04 | 1.22 | 3 | 1.08 | 0.73 | 1.02 | 2.5 | 1.75 | 1.1 | 1.75 |
| 1.63 | 1.73 | 1.04 | 0.94 | 1.53 | 1.07 | 2.72 | 1.55 | 1.4 | 1.22 | 1.4 |
| 0.89 | 1.11 | 1.03 | 0.63 | 2.41 | 0.9 | 1.22 | 0.62 | 0.79 | 0.93 | 0.79 |
| 1.28 | 1.99 | 0.71 | 0.93 | 0.63 | 0.61 | 0.21 | 1.05 | 3.33 | 0.59 | 3.33 |
| 2.6 | 1.78 | 1.36 | 1.52 | 0.92 | 2.27 | 0.88 | 1.18 | 0.88 | 1.38 | 0.88 |
| 0.47 | 5.73 | 1.02 | 1.47 | 0.91 | 0.57 | 1.38 | 1.5 | 1.67 | 0.54 | 1.67 |
| 14.98 | 1.14 | 0.99 | 1.23 | 16.1 | 0.57 | 0.81 | 0.87 | 0.5 | 0.79 | 0.5 |
| 2.91 | 1.88 | 1.48 | 0.83 | 0.75 | 0.63 | 0.5 | 0.5 | 1.73 | 0.58 | 1.73 |
| 3.98 | 0.69 | 1.05 | 1.81 | 0.74 | 0.91 | 0.74 | 1.21 | 0.59 | 0.5 | 0.59 |
| 0.64 | 1.23 | 0.48 | 0.26 | 0.75 | 0.41 | 0.55 | 2 | 1.78 | 0.89 | 1.78 |
| 98.24 | 0.95 | 1.35 | 0.44 | 24.33 | 0.8 | 0.77 | 1.22 | 1.04 | 0.78 | 1.04 |
| 0.98 | 1.39 | 3.6 | 1.06 | 1.67 | 1.79 | 0.72 | 1.5 | 0.62 | 2.83 | 0.62 |
| 0.34 | 0.57 | 0.96 | 2.6 | 1.76 | 0.95 | 0.88 | 1.3 | 1.7 | 1.3 | 1.7 |
| 1.1 | 3.84 | 3.33 | 3.62 | 0.91 | 1.7 | 1.27 | 3.06 | 0.61 | 1.11 | 0.61 |
| 0.7 | 5.56 | 0.47 | 0.55 | 0.82 | 0.87 | 0.45 | 1.17 | 2.56 | 4.45 | 2.56 |
| 2.32 | 1.08 | 1.47 | 1.08 | 0.86 | 1.74 | 1.03 | 0.89 | 1.06 | 4.17 | 1.06 |
| 1 | 0.93 | 0.89 | 0.51 | 0.91 | 0.79 | 2.12 | 1 | 0.07 | 0.89 | 0.07 |
| 5.95 | 0.58 | 3.07 | 0.49 | 4.83 | 4.74 | 0.59 | 1.03 | 1.15 | 4.71 | 1.15 |

| | | | | | | | | | | |
|---|---|---|---|---|---|---|---|---|---|---|
| 1.02 | 0.94 | 0.92 | 4 | 2.33 | 0.69 | 1.64 | 2.17 | 1.86 | 1.21 | 1.86 |
| 1.23 | 1.55 | 1.61 | 0.33 | 4.5 | 0.58 | 1.38 | 1.26 | 3.33 | 0.62 | 3.33 |
| 1.3 | 4.67 | 1.24 | 1.84 | 0.97 | 1.11 | 0.98 | 2.22 | NA | 0.75 | NA |
| 0.95 | 0.53 | 0.44 | 0.2 | 0.42 | 1.14 | 0.97 | 0.59 | 2.64 | 0.69 | 2.64 |
| 1.97 | 0.76 | 1.26 | 2.55 | 1.2 | 2.15 | 1.1 | 1.17 | 1.38 | 1.21 | 1.38 |
| 0.89 | 1.02 | 0.84 | 1.06 | 1.44 | 1.25 | 2.1 | 0.67 | 4.53 | 0.68 | 4.53 |
| 0.56 | 0.81 | 1.54 | 1.05 | 0.8 | 1.16 | 0.94 | 0.68 | 3.23 | 0.7 | 3.23 |
| 1.02 | 1.86 | 1.99 | 1.61 | 1.18 | 0.56 | 3.55 | 3.62 | 0.19 | 7.58 | 0.19 |
| 1.16 | 0.7 | 0.88 | 2.91 | 1.78 | 0.82 | 0.83 | 0.84 | 0.62 | 1 | 0.62 |
| 1.07 | 0.95 | 0.41 | 1.93 | 1.93 | 1.43 | 1.38 | 0.92 | 1.49 | 1.14 | 1.49 |
| 2.71 | 0.62 | 0.78 | 1.1 | 1.33 | 1.29 | 0.84 | 1 | 1.31 | 0.56 | 1.31 |
| 0.96 | 1.15 | 3.7 | 1.28 | 0.24 | 1.07 | 1.14 | 1 | 0.12 | 2.75 | 0.12 |
| 1.29 | 0.62 | 0.69 | 0.32 | 0.62 | 2.67 | 1.28 | 1.5 | 2.5 | 0.76 | 2.5 |
| 1.98 | 4.79 | 1.53 | 0.56 | 0.62 | 0.72 | 1.01 | 0.78 | 0.48 | 0.97 | 0.48 |
| 0.84 | 1.06 | 0.97 | 7.31 | 1.27 | 2.28 | 0.61 | 1.88 | 1.12 | 0.09 | 1.12 |
| 1.05 | 1.1 | 1.73 | 1.64 | 2.11 | 1.76 | 1.44 | 0.97 | 1.01 | 1.1 | 1.01 |
| 1.23 | 1.22 | 1.52 | 4.5 | 0.51 | 0.79 | 0.99 | 1.12 | 0.55 | 1.52 | 0.55 |
| 1.45 | 0.48 | 1.59 | 0.41 | 1.17 | 2 | 1.79 | 1.62 | 0.58 | 0.93 | 0.58 |
| 7.5 | 2.84 | 1.91 | 10 | 1.83 | 1.25 | 1.77 | 1.33 | 1.2 | 16.67 | 1.2 |
| 1.44 | 1.56 | 0.91 | 1.72 | 0.04 | 1.24 | 1.5 | 4.5 | 1.3 | 5.25 | 1.3 |
| 8.67 | 1.17 | 1.67 | 0.79 | 1.98 | 1.57 | 1.93 | 1.69 | 13.1 | 1.84 | 13.1 |
| 0.73 | 2.52 | 1.86 | 0.91 | 1.04 | 0.76 | 2.04 | 9.26 | 2.33 | 0.82 | 2.33 |
| 0.72 | 0.97 | 5.75 | 8.01 | 1.03 | 1.84 | 1.04 | 2.12 | 1.85 | 0.27 | 1.85 |
| 1.08 | 1.04 | 1.23 | 2.01 | 0.05 | 0.46 | 1.27 | 0.99 | 2.13 | 0.45 | 2.13 |
| 1 | 1.13 | 0.77 | 4.35 | 1.19 | 1.29 | 1.57 | 4.42 | 1.26 | 1.65 | 1.26 |
| 0.81 | 1.22 | 0.78 | 3.75 | 1.71 | 1.69 | 1.49 | 0.88 | 4.48 | 2.82 | 4.48 |
| 0.3 | 1.05 | 1.4 | 0.48 | 0.58 | 0.96 | 0.77 | 0.73 | 0.73 | 0.71 | 0.73 |
| 1.8 | 0.94 | 0.6 | 1.98 | 1.01 | 0.81 | 0.69 | 0.75 | 0.5 | 0.55 | 0.5 |
| 0.89 | 0.7 | 1.4 | 0.98 | 2.11 | 1.01 | 0.92 | 1.3 | 1.17 | 0.56 | 1.17 |
| 1.15 | 0.88 | 0.42 | 5.21 | 0.84 | 12.81 | 3.04 | 1.12 | 2.2 | 0.58 | 2.2 |
| 1.29 | 0.4 | 2.25 | 4.88 | 3.67 | 0.91 | 0.64 | 1 | 2.64 | 0.41 | 2.64 |
| 1.78 | 1.03 | 1.08 | 0.86 | 1.4 | 0.79 | 0.99 | 2.16 | 1.9 | 0.48 | 1.9 |
| 0.05 | 2.46 | 0.88 | 3.8 | 3.89 | 1.97 | 1.27 | 1.11 | 1.1 | 8.39 | 1.1 |

| | | | | | | | | | | |
|---|---|---|---|---|---|---|---|---|---|---|
| 0.74 | 0.61 | 0.81 | 0.23 | 2 | 0.95 | 0.96 | 0.88 | 0.6 | 0.35 | 0.6 |
| 0.9 | 1.66 | 1.01 | 0.79 | 0.98 | 0.87 | 2.17 | 1 | 0.33 | 0.73 | 0.33 |
| 1.26 | 8.08 | 1.68 | 19.42 | 1 | 0.88 | 16.14 | 1 | 12.75 | 0.51 | 12.75 |
| 0.69 | 0.92 | 1.11 | 0.76 | 1.87 | 1.17 | 1.25 | 1.23 | 0.36 | 0.94 | 0.36 |
| 0.52 | 1.04 | 2.43 | 0.55 | 1.44 | 1.21 | 1.09 | 1.25 | 1.75 | 1.21 | 1.75 |
| 0.51 | 0.83 | 0.97 | 2.09 | 1.3 | 1.25 | 0.74 | 1.15 | 2.09 | 0.92 | 2.09 |
| 12.77 | 1.16 | 1.46 | 2.17 | 0.92 | 1.08 | 0.83 | 0.88 | 2.67 | 3.11 | 2.67 |
| 0.9 | 2.72 | 0.65 | 1.03 | 1.12 | 2.63 | 1.46 | 0.67 | 6.81 | 1.36 | 6.81 |
| 0.82 | 0.78 | 0.75 | 3.59 | 1.37 | 1 | 1.09 | 6.22 | 0.46 | 2.94 | 0.46 |
| 0.88 | 1.13 | 2.34 | 0.82 | 1.14 | 2.27 | 1.53 | 1.95 | 2.63 | 6.02 | 2.63 |
| 0.9 | 0.64 | 1.47 | 3.71 | 2.66 | 3.87 | 0.73 | 1 | 1.94 | 4.88 | 1.94 |
| 11.72 | 11.74 | 0.06 | 0.36 | 0.69 | 1.78 | 0.25 | 0.9 | 3.38 | 17.38 | 3.38 |
| 1.72 | 0.86 | 1.56 | 1.78 | 0.73 | 1.51 | 0.83 | 1.12 | 2.07 | 1.23 | 2.07 |
| 0.14 | 0.61 | 0.8 | 0.16 | 2 | 0.71 | 1.51 | 2 | 0.25 | 1.5 | 0.25 |

| 220804_s_at | 206858_s_at | 217192_s_at | 203543_s_at | 216248_s_at | 214520_at | 212063_at | 220308_at | 218182_s_at | 211120_x_at | 203680_at |
|---|---|---|---|---|---|---|---|---|---|---|
| 2.87 | 0.64 | 2.91 | 1 | 1.22 | 4.4 | 1.29 | 1.33 | 0.66 | 1.9 | 1.58 |
| 3.24 | 1.25 | 1.38 | 0.8 | 1.02 | 0.96 | 0.63 | 1 | 2.23 | 2.78 | 1.62 |
| 1.14 | 4.84 | 1.15 | 0.53 | 1.33 | 0.27 | 0.45 | 1.06 | 1.42 | 1.92 | 1.74 |
| 3.82 | 1.61 | 1.47 | 2.14 | 2.4 | 2.45 | 1.18 | 1.64 | 2.88 | 966.77 | 0.98 |
| 1.03 | 0.54 | 1.22 | 1.48 | 1.58 | 1.64 | 1.39 | 0.83 | 4.44 | 0.83 | 0.93 |
| 2.18 | 1.11 | 1.18 | 1.31 | 3.75 | 1.6 | 1.01 | 1.61 | 1.27 | 0.93 | 1.78 |
| 4 | 2.22 | 0.51 | 1.76 | 1 | 4.5 | 1.19 | 1.08 | 0.2 | 0.93 | 0.21 |
| 0.47 | 0.79 | 1.39 | 1.05 | 1.08 | 1.33 | 0.79 | 1.65 | 0.53 | 0.62 | 0.87 |
| 0.64 | 0.76 | 0.84 | 0.57 | 1.64 | 2.84 | 0.85 | 1.04 | 2.22 | 1.49 | 1.27 |
| 7.5 | 1.01 | 4.42 | 1.75 | 4.14 | 2.6 | 0.67 | 3.48 | 1.61 | 6.09 | 1.2 |
| 0.14 | 1.31 | 4.35 | 1.27 | 2.69 | 1.44 | 1.86 | 0.2 | 0.77 | 0.76 | 0.95 |
| 1.57 | 1.22 | 0.6 | 0.87 | 0.56 | 1.51 | 1 | 0.92 | 1.12 | 1.4 | 0.91 |
| 2.46 | 1.57 | 1.05 | 0.73 | 1.02 | 3.55 | 0.26 | 1.34 | 0.46 | 1.19 | 1.54 |
| 16.64 | 1.32 | 8.85 | 3.95 | 0.53 | 18.54 | 0.01 | 7.3 | 11.93 | 58.07 | 0.09 |
| 0.75 | 0.71 | 2.23 | 2.21 | 0.93 | 1.6 | 0.89 | 0.43 | 1.54 | 0.38 | 2.36 |
| 2.39 | 1.32 | 2.81 | 1.16 | 0.8 | 0.75 | 0.8 | 1.52 | 0.53 | 10.1 | 1.3 |
| 0.8 | 1.01 | 2.44 | 0.49 | 0.88 | 1.26 | 1.01 | 0.67 | 6.34 | 2.05 | 1.27 |
| 8.85 | 1.31 | 7.84 | 1.36 | 1.56 | 1.28 | 0.71 | 1.16 | 2.97 | 3.36 | 1.39 |
| 136 | 0.83 | 0.99 | 0.36 | 1.31 | 4.33 | 0.66 | 1.05 | 3.44 | 1 | 0.36 |
| 1.7 | 120.41 | 2.67 | 0.93 | 4.02 | 3.59 | 4.75 | 4.01 | 1.66 | 1.58 | 1.01 |
| 1.78 | 0.9 | 94.18 | 0.39 | 1.27 | 0.99 | 0.51 | 3.33 | 0.79 | 9.56 | 0.28 |
| 13.62 | 1.69 | 3.12 | 91.59 | 1.67 | 13.75 | 2.21 | 7.25 | 2.69 | 50.44 | 1.33 |
| 1.17 | 0.85 | 0.96 | 1.98 | 76.84 | 1.43 | 1.09 | 5.08 | 1.07 | 0.85 | 1.43 |
| 0.92 | 2.31 | 0.75 | 2.48 | 5.24 | 62.74 | 1.67 | 2.7 | 1.36 | 1.27 | 5.33 |
| 0.37 | 0.37 | 0.89 | 0.04 | 0.17 | 3.75 | 52.06 | 0.94 | 3.42 | 1.23 | 0.31 |
| 0.73 | 0.86 | 1.02 | 1.25 | 0.84 | 5.21 | 1.21 | 43.71 | 1.1 | 0.72 | 0.15 |
| 4.15 | 1.06 | 4.73 | 1.66 | 2.32 | 0.66 | 0.52 | 0.41 | 37.13 | 2.05 | 1 |
| 1.42 | 0.6 | 1.16 | 1.59 | 15.09 | 3.95 | 0.6 | 1.27 | 6.77 | 33.54 | 0.46 |
| 0.75 | 0.06 | 1.56 | 0.48 | 1.62 | 0.75 | 0.49 | 5.68 | 0.46 | 1.73 | 32.61 |
| 1.37 | 0.96 | 0.72 | 0.95 | 2 | 2.02 | 1.27 | 1.72 | 1.1 | 2.41 | 1.29 |

| | | | | | | | | | | |
|---|---|---|---|---|---|---|---|---|---|---|
| 1.41 | 0.93 | 0.89 | 0.91 | 1.9 | 2.21 | 0.71 | 1.22 | 0.35 | 0.73 | 0.54 |
| 1.23 | 2.32 | 1.05 | 8.05 | 0.87 | 2.46 | 2.05 | 0.95 | 0.24 | 5.13 | 0.71 |
| 1.35 | 1.2 | 2.16 | 1.42 | 1.08 | 0.86 | 0.97 | 0.98 | 1.04 | 1.04 | 1.25 |
| 1.3 | 0.35 | 5.91 | 0.45 | 2.64 | 3.51 | 0.54 | 1.99 | 0.42 | 1.56 | 16.68 |
| 0.36 | 1.29 | 0.22 | 0.43 | 1.5 | 21 | 0.11 | 0.64 | 4.64 | 0.2 | 0.62 |
| 4.86 | 1.34 | 4.08 | 0.54 | 2.3 | 2.03 | 1.09 | 2.96 | 2.19 | 1.11 | 0.79 |
| 2.4 | 0.36 | 2.43 | 1.16 | 33.58 | 0.33 | 0.28 | 2.11 | 0.52 | 1.41 | 6.88 |
| 7.71 | 0.8 | 1.79 | 1.13 | 1.12 | 0.95 | 1.04 | 1.18 | 1.74 | 6.6 | 0.79 |
| 7.78 | 1.05 | 1.66 | 0.99 | 0.93 | 0.6 | 1.25 | 0.9 | 0.92 | 3.13 | 25.25 |
| 0.67 | 1.3 | 0.52 | 8.08 | 0.13 | 0.5 | 2.05 | 2.43 | 1.38 | 1.27 | 3.33 |
| 5.75 | 1.38 | 1.07 | 0.71 | 1.45 | 0.73 | 0.06 | 0.23 | 0.52 | 1.35 | 0.95 |
| 0.73 | 1.04 | 0.42 | 1.22 | 0.94 | 2.28 | 0.95 | 1.93 | 1.04 | 1.23 | 1.04 |
| 0.22 | 0.91 | 2.92 | 4.65 | 1.2 | 1.72 | 0.74 | 3.96 | 10.42 | 0.22 | 3.11 |
| 0.83 | 4.77 | 1.59 | 1.71 | 0.32 | 1.59 | 3.35 | 2.45 | 1.95 | 2.55 | 2.55 |
| 0.3 | 0.81 | 1.95 | 3.21 | 1.12 | 1.56 | 0.78 | 3.98 | 7.79 | 0.22 | 3.56 |
| 1.1 | 14.43 | 1.29 | 1.19 | 8.07 | 1.37 | 2.72 | 1.42 | 3.69 | 3.12 | 14.93 |
| 16 | 1.14 | 1.46 | 1.19 | 0.69 | 0.21 | 0.96 | 0.63 | 0.71 | 1.9 | 2.63 |
| 2.81 | 1.32 | 2.88 | 1.48 | 0.98 | 2.53 | 0.74 | 0.19 | 1.02 | 1.46 | 0.47 |
| 2.46 | 1.06 | 0.56 | 1.06 | 0.95 | 1.17 | 1.13 | 4.34 | 1.04 | 3.19 | 0.98 |
| 1.1 | 0.95 | 1.16 | 2.37 | 1.98 | 1.18 | 0.96 | 0.66 | 1.1 | 1.22 | 0.82 |
| 0.5 | 1.7 | 0.43 | 0.59 | 0.93 | 1 | 0.97 | 1.51 | 0.45 | 0.29 | 1.06 |
| 2.07 | 0.53 | 0.74 | 0.89 | 1.01 | 0.63 | 1.27 | 2.62 | 1.6 | 4.75 | 0.63 |
| 1.05 | 1.21 | 1.65 | 1.22 | 0.77 | 1.76 | 1.16 | 1.26 | 2.6 | 5.99 | 1.16 |
| 2.73 | 2.38 | 4.69 | 1.69 | 1.7 | 1.11 | 1.76 | 1.65 | 1.61 | 2.04 | 0.99 |
| 2.38 | 0.64 | 3.92 | 0.5 | 0.06 | 3.16 | 0.92 | 5.67 | 1.13 | 3.73 | 0.2 |
| 9.4 | 0.92 | 1.24 | 0.72 | 2.36 | 0.62 | 0.79 | 0.18 | 0.75 | 1 | 1.95 |
| 2.5 | 0.75 | 1.8 | 0.04 | 0.03 | 1.59 | 1.08 | 9.45 | 4.28 | 1.89 | 0.37 |
| 15.09 | 1.99 | 2.12 | 0.52 | 2.38 | 0.95 | 6.8 | 1.18 | 6.39 | 0.9 | 1.21 |
| 2 | 0.65 | 1.14 | 1.75 | 1.01 | 0.75 | 1.12 | 3.08 | 0.75 | 1.88 | 0.96 |
| 0.22 | 1.08 | 2.01 | 3.89 | 1.67 | 0.75 | 0.71 | 5 | 7.78 | 0.3 | 2.67 |
| 0.66 | 2.73 | 3.28 | 2.46 | 2.15 | 0.79 | 0.6 | 1.45 | 1.81 | 0.16 | 0.77 |
| 1.79 | 1.39 | 1.1 | 2.49 | 1.32 | 1.07 | 2.03 | 3.78 | 0.49 | 0.31 | 2.05 |
| 1.14 | 1.51 | 3 | 3.5 | 0.64 | 1.54 | 1.27 | 1.5 | 0.9 | 0.73 | 1.29 |

| | | | | | | | | | | |
|---|---|---|---|---|---|---|---|---|---|---|
| 0.5 | 1.05 | 0.87 | 2.23 | 0.47 | 6.88 | 1.29 | 1.43 | 2.18 | 0.75 | 0.65 |
| 3.67 | 1.26 | 0.98 | 1 | 1.19 | 1.2 | 0.73 | 1.85 | 46.18 | 2.1 | 2.74 |
| 2.25 | 1.83 | 5.94 | 0.5 | 1.57 | 1.95 | 1.22 | 1 | 0.86 | 0.83 | 1 |
| 1.4 | 2.15 | 0.73 | 6.78 | 12.86 | 3.33 | 19.01 | 2.17 | 3.14 | 3.25 | 3.03 |
| 1.4 | 1.65 | 3.53 | 1.18 | 2.21 | 0.75 | 1.19 | 0.8 | 1.16 | 1.91 | 0.96 |
| 1.57 | 0.95 | 0.95 | 1.28 | 1.04 | 5.68 | 0.94 | 1.05 | 1.1 | 4.6 | 1.69 |
| 3.38 | 0.8 | 1.12 | 0.69 | 0.22 | 1.97 | 0.24 | 2.63 | 0.8 | 1.64 | 0.25 |
| 0.3 | 0.97 | 2.44 | 1.37 | 1.78 | 2.81 | 1.79 | 1.1 | 0.69 | 1.94 | 1.14 |
| 0.44 | 1.57 | 0.56 | 1.82 | 1.31 | 1.38 | 1.97 | 1.67 | 0.38 | 1.16 | 3.76 |
| 0.52 | 0.77 | 1.22 | 1.6 | 2.02 | 1.11 | 1.68 | 0.77 | 1.72 | 1.49 | 1.08 |
| 6.88 | 2.63 | 1.22 | 0.5 | 0.85 | 5.01 | 0.83 | 1.01 | 1.79 | 5.33 | 2.46 |
| 0.18 | 0.8 | 1.54 | 0.89 | 1.32 | 1.25 | 10.85 | 0.89 | 0.14 | 1.21 | 1.17 |
| 1 | 0.65 | 0.62 | 2.1 | 1.19 | 1 | 2.45 | 0.59 | 2.94 | 1.3 | 0.7 |
| 1.72 | 1.39 | 1.86 | 1.18 | 1.08 | 2.28 | 1 | 2.2 | 3.84 | 0.78 | 0.94 |
| 9.5 | 1.07 | 5.83 | 0.72 | 1.46 | 1.33 | 2.14 | 4.89 | 0.19 | 0.14 | 1.27 |
| 0.03 | 1.97 | 0.7 | 0.31 | 0.21 | 0.01 | 21.52 | 0.1 | 0.97 | 0.05 | 13.1 |
| 0.8 | 1.58 | 2.55 | 0.86 | 1.02 | 0.72 | 0.4 | 1.68 | 12.89 | 0.63 | 0.3 |
| 0.4 | 0.54 | 1.41 | 1.34 | 0.8 | 0.25 | 0.91 | 0.35 | 0.74 | 0.29 | 3.62 |
| 1.16 | 1.47 | 1.83 | 1.48 | 4.75 | 2.26 | 1.39 | 0.36 | 0.83 | 1.78 | 0.82 |
| 2.61 | 2.55 | 1.15 | 1.62 | 1.14 | 1.71 | 0.85 | 7.86 | 3.94 | 3.14 | 0.76 |
| 0.06 | 1.71 | 0.77 | 2.85 | 0.64 | 0.96 | 309.1 | 2.01 | 0.97 | 0.95 | 0.72 |
| 1.9 | 0.49 | 1.11 | 1.65 | 0.38 | 0.57 | 4.15 | 1.8 | 2.51 | 1.85 | 1.1 |
| 3.5 | 1.46 | 0.77 | 1.46 | 1.21 | 3.75 | 1 | 2.29 | 0.36 | 0.7 | 0.4 |
| 10.64 | 1.43 | 1.29 | 1.36 | 2.45 | 2.07 | 0.84 | 0.69 | 1.08 | 1.19 | 4.9 |
| 6.01 | 0.79 | 1.04 | 0.63 | 2.12 | 0.53 | 0.93 | 0.77 | 1.17 | 0.71 | 3.85 |
| 1 | 0.99 | 2.47 | 0.84 | 1.39 | 0.59 | 1.07 | 0.44 | 1.04 | 0.35 | 1.14 |
| 4.28 | 0.91 | 2.89 | 0.59 | 0.89 | 0.36 | 0.83 | 1.15 | 1.98 | 3.03 | 0.64 |
| 0.6 | 0.88 | 0.98 | 0.36 | 2.29 | 0.32 | 0.82 | 1.69 | 1.02 | 2.89 | 1.85 |
| 0.58 | 1.22 | 1.61 | 1.05 | 0.81 | 1.4 | 0.86 | 0.85 | 1.07 | 2.05 | 0.79 |
| 0.91 | 0.71 | 0.83 | 0.89 | 2.01 | 0.81 | 0.9 | 1.49 | 1.19 | 0.38 | 0.84 |
| 1.27 | 1.14 | 2.29 | 0.79 | 1.28 | 3.69 | 0.79 | 0.8 | 0.87 | 0.77 | 0.92 |
| 1.5 | 0.66 | 0.49 | 1.8 | 0.67 | 2.04 | 0.88 | 1.05 | 0.91 | 1.21 | 0.33 |
| 0.62 | 18.58 | 2.21 | 8.86 | 3.48 | 0.38 | 2.41 | 0.48 | 0.52 | 0.9 | 0.79 |

| | | | | | | | | | | |
|---|---|---|---|---|---|---|---|---|---|---|
| 1.12 | 1.46 | 1.61 | 8.02 | 0.88 | 2.64 | 0.9 | 1.85 | 1.99 | 1.22 | 0.48 |
| 0.67 | 0.69 | 0.82 | 0.2 | 1.57 | 2.35 | 0.86 | 1.5 | 0.61 | 5.85 | 0.83 |
| 1.11 | 1.13 | 2.91 | 0.65 | 1.81 | 7.3 | 1.17 | 2.45 | 2.19 | 0.97 | 1.03 |
| 2.22 | 1.16 | 3.38 | 0.41 | 1.02 | 0.92 | 0.84 | 1.01 | 3.2 | 0.38 | 4.54 |
| 1.45 | 0.83 | 1.36 | 1.15 | 1.28 | 4.08 | 0.97 | 2.16 | 1.38 | 1.07 | 1.14 |
| 0.88 | 0.16 | 0.79 | 0.5 | 1 | 4.29 | 1.2 | 1.44 | 5.28 | 2.29 | 0.71 |
| 1.64 | 1.05 | 1.63 | 0.79 | 1.2 | 1.45 | 0.85 | 2.1 | 3.7 | 1.86 | 0.8 |
| 0.56 | 1.68 | 1.34 | 5.44 | 1.25 | 0.19 | 1.97 | 1.09 | 1.87 | 0.84 | 1.47 |
| 1.47 | 0.78 | 1.9 | 0.84 | 0.51 | 4.53 | 1.63 | 1.6 | 1.88 | 1.73 | 1.25 |
| 1.96 | 0.59 | 0.92 | 1.26 | 1.41 | 0.99 | 0.17 | 2.02 | 4.77 | 0.51 | 0.76 |
| 14.89 | 0.89 | 1.5 | 0.57 | 1.52 | 1.55 | 1.06 | 1.82 | 1.08 | 0.56 | 0.92 |
| 0.87 | 1.09 | 2.24 | 0.73 | 1.08 | 3.26 | 1.1 | 1.04 | 1.25 | 0.57 | 1.43 |
| 1.88 | 0.52 | 2.38 | 0.93 | 4.13 | 0.44 | 0.96 | 2.44 | 2.76 | 1.88 | 2.25 |
| 1.47 | 0.6 | 2.61 | 0.65 | 4.31 | 3.11 | 0.81 | 1.18 | 0.88 | 0.74 | 9.25 |
| 0.64 | 1.09 | 1.18 | 2.97 | 0.78 | 2.11 | 5 | 1 | 1.01 | 1 | 1.06 |
| 3.91 | 1.75 | 1.09 | 1.05 | 1.57 | 7.26 | 1.18 | 0.98 | 9.57 | 4.5 | 1.26 |
| 0.29 | 1.76 | 3.28 | Inf | 7.62 | 0.59 | 1.76 | 0.62 | 7.94 | 0.79 | 1.39 |
| 1.18 | 1.93 | 0.62 | 5.6 | 4.88 | 2.67 | 13.58 | 1.2 | 3.82 | 2.35 | 1.98 |
| 0.89 | 1.19 | 2.74 | 0.68 | 1.05 | 0.93 | 2.27 | 1.8 | 6.75 | 1.15 | 0.74 |
| 1.67 | 1.61 | 2.26 | 2.6 | 10 | 1.74 | 1.3 | 4.88 | 0.34 | 1.65 | 0.3 |
| 0.68 | 1.31 | 1.36 | 0.33 | 0.18 | 4.75 | 1.47 | 1.64 | 2.29 | 1.09 | 0.36 |
| 0.84 | 1.49 | 1.14 | 0.2 | 0.84 | 5.13 | 1.41 | 0.65 | 1.26 | 2.5 | 0.54 |
| 2.02 | 1.08 | 1.6 | 0.78 | 1.01 | 2.49 | 1.09 | 1.25 | 0.71 | 0.94 | 1.45 |
| 1.12 | 1.59 | 0.78 | 0.59 | 0.88 | 1.5 | 1.09 | 3.02 | 4.03 | 0.59 | 0.48 |
| 1.6 | 0.35 | 0.62 | 1.51 | 5.57 | 1.19 | 0.64 | 1.62 | 0.97 | 1.41 | 1.07 |
| 0.96 | 0.55 | 0.85 | 1.74 | 0.17 | 0.8 | 0.73 | 1.42 | 0.47 | 3.28 | 1.09 |
| 3.5 | 0.62 | 3.22 | 0.89 | 1.18 | 1.33 | 1.11 | 1 | 1.05 | 0.33 | 1.2 |
| 1.08 | 1.07 | 1.13 | 2.54 | 1.54 | 1.42 | 1.83 | 0.45 | 1.19 | 3.83 | 1.33 |
| 1.58 | 0.79 | 0.82 | 0.2 | 0.49 | 2.76 | 0.85 | 2.13 | 0.77 | 0.67 | 0.55 |
| 0.65 | 1 | 1.08 | 1.74 | 1.09 | 1.76 | 1.03 | 3.59 | 1.38 | 1.15 | 1.21 |
| 1.75 | 0.13 | 1.96 | 12.19 | 1.15 | 0.43 | 0.82 | 0.44 | 0.69 | 0.7 | 0.13 |
| 3.67 | 1.26 | 0.98 | 1 | 1.19 | 1.2 | 0.73 | 1.85 | 46.18 | 2.1 | 2.74 |
| 1 | 0.92 | 1.09 | 0.81 | 2.8 | 0.11 | 0.82 | 0.65 | 3.18 | 0.4 | 1.84 |

| | | | | | | | | | | |
|---|---|---|---|---|---|---|---|---|---|---|
| 0.6 | 0.79 | 1.89 | 0.85 | 0.69 | 0.23 | 2.59 | 0.95 | 1.15 | 1.88 | 1.16 |
| 1.34 | 0.98 | 1.54 | 0.86 | 0.81 | 1.24 | 1.64 | 4.31 | 3.6 | 2.78 | 1.21 |
| 1.32 | 0.95 | 0.96 | 1.03 | 0.82 | 2.38 | 1.4 | 2.7 | 0.9 | 2.87 | 1.01 |
| 1.39 | 1.96 | 0.95 | 0.9 | 0.83 | 1.61 | 2.43 | 0.62 | 1.66 | 6.7 | 1.39 |
| 1.67 | 0.34 | 1.15 | 1.45 | 0.62 | 0.75 | 0.83 | 0.89 | 1.23 | 3.78 | 0.68 |
| 0.29 | 0.98 | 0.67 | 0.77 | 0.85 | 1.06 | 0.96 | 0.93 | 1.09 | 0.5 | 1.05 |
| 0.46 | 0.96 | 5.77 | 0.9 | 0.71 | 0.7 | 2.62 | 1.16 | 0.17 | 1.38 | 1.02 |
| 4.38 | 1.57 | 1 | 1.32 | 1.68 | 2.6 | 0.82 | 0.91 | 0.78 | 0.51 | 0.92 |
| 0.92 | 0.96 | 1.14 | 17.85 | 14.12 | 0.88 | 0.97 | 3.76 | 1.52 | 7.61 | 1.38 |
| 2 | 0.76 | 1.31 | 0.39 | 2.02 | 7.2 | 2.48 | 1.75 | 0.52 | 0.42 | 1.04 |
| 6.81 | 0.83 | 2.2 | 0.6 | 2.14 | 0.96 | 1.44 | 0.31 | 3.33 | 4.41 | 3.18 |
| 2.84 | 1.27 | 1 | 0.08 | 0.96 | 0.92 | 1.16 | 2.61 | 1.28 | 0.89 | 1.23 |
| 0.77 | 1.66 | 0.73 | 6.23 | 9.13 | 3.58 | 13.34 | 1 | 3.71 | 2.37 | 2.78 |
| 1.46 | 1.2 | 1.3 | 1.38 | 7.53 | 1.29 | 0.85 | 1.4 | 0.47 | 3.41 | 1.23 |
| 0.52 | 0.98 | 0.64 | 0.82 | 1.07 | 1.93 | 2.02 | 0.52 | 0.59 | 0.5 | 1.21 |
| 1.1 | 1.09 | 3 | 0.95 | 1.31 | 2.87 | 0.94 | 0.75 | 1.19 | 0.53 | 1.28 |
| 1.73 | 1.06 | 1.43 | 1.17 | 0.95 | 1.01 | 3.55 | 1.11 | 1.02 | 1.75 | 0.94 |
| 2.24 | 1.17 | 1.46 | 0.44 | 2.02 | 1.61 | 0.71 | 2.44 | 1.27 | 3.29 | 0.98 |
| 0.81 | 0.88 | 0.79 | 0.74 | 0.94 | 0.85 | 0.87 | 0.92 | 0.47 | 1.16 | 1.29 |
| 1.87 | 1.07 | 1.09 | 0.68 | 0.47 | 5.46 | 0.75 | 1.1 | 1.04 | 1.51 | 3.55 |
| 0.35 | 0.58 | 0.89 | 1.47 | 2.98 | 8 | 1.5 | 1.34 | 1.06 | 1.44 | 1.27 |
| 3.37 | 1.53 | 3 | 0.16 | 2.09 | 5.56 | 1.36 | 1.28 | 1.54 | 6.15 | 2.1 |
| 0.58 | 2.24 | 1.18 | 0.79 | 1.09 | 2.84 | 0.86 | 1.82 | 2.25 | 3.52 | 1.52 |
| 0.83 | 4.29 | 1.05 | 0.88 | 4.15 | 1.17 | 0.33 | 0.75 | 0.14 | 3.42 | 0.93 |
| 0.67 | 0.75 | 0.96 | 1.09 | 0.2 | 0.58 | 1.2 | 0.76 | 1.18 | 0.61 | 0.56 |
| 1.14 | 1.14 | 3.74 | 1.81 | 1.42 | 1.46 | 0.85 | 1.61 | 1.09 | 1.56 | 1.98 |
| 1.39 | 1.47 | 1.45 | 0.98 | 1.46 | 0.69 | 1.56 | 0.53 | 1.04 | 0.46 | 2.75 |
| 0.37 | 0.92 | 9.2 | 0.93 | 1.76 | 3.47 | 1.14 | 0.22 | 0.5 | 0.91 | 1.51 |
| 0.76 | 1.27 | 1.34 | 0.87 | 0.63 | 0.66 | 1.16 | 2.59 | 1.1 | 3.28 | 0.97 |
| 0.8 | 0.89 | 1.02 | 0.71 | 0.57 | 0.61 | 0.81 | 1.52 | 0.36 | 0.75 | 0.8 |
| 1.14 | 1.51 | 1.19 | 0.63 | 1.02 | 2.25 | 0.72 | 1.97 | 2.65 | 9.83 | 1.22 |
| 0.75 | 0.92 | 3.31 | 0.98 | 1.48 | 2.83 | NA | 1.01 | 2.95 | 2.92 | 0.98 |
| 1.33 | 0.57 | 0.64 | 3.75 | 1.31 | 2.19 | 1.32 | 0.78 | 0.39 | 1.14 | 0.94 |

| | | | | | | | | | | |
|---|---|---|---|---|---|---|---|---|---|---|
| 0.25 | 1.07 | 1.16 | 1.29 | 1.09 | 3.19 | 0.86 | 1.96 | 0.25 | 0.67 | 7.1 |
| 1.59 | 0.82 | 1.47 | 0.88 | 1.58 | 1.85 | 0.93 | 1.57 | 2.19 | 4.4 | 1.61 |
| 0.83 | 0.92 | 1.33 | 0.97 | 0.99 | 0.75 | 0.98 | 1.02 | 1.13 | 0.57 | 1.69 |
| 1.22 | 2.04 | 0.65 | 1.41 | 1.04 | 1.27 | 0.63 | 0.93 | 2.31 | 1.77 | 0.99 |
| 2.5 | 0.9 | 0.62 | 0.87 | 2.16 | 1.6 | 2.35 | 6.92 | 1.84 | 0.28 | 1.69 |
| 2.37 | 1.17 | 1.01 | 1.04 | 1.05 | 0.87 | 1.02 | 1.23 | 1.11 | 1.17 | 0.96 |
| 0.89 | 0.93 | 1.1 | 1.04 | 2.62 | 1.64 | 0.72 | 1.12 | 0.28 | 0.91 | 0.63 |
| 1.85 | 1.4 | 1.51 | 0.73 | 1.09 | 1.67 | 0.42 | 1.71 | 0.44 | 0.57 | 1.06 |
| 0.52 | 0.95 | 0.8 | 1.34 | 3.25 | 0.98 | 0.98 | 0.53 | 1.04 | 1.52 | 1.07 |
| 1.04 | 1.05 | 0.96 | 0.92 | 1.28 | 1.83 | 0.55 | 0.96 | 0.21 | 1.36 | 0.78 |
| 2 | 1.17 | 1 | 0.42 | 0.31 | 2 | 1.16 | 1.61 | 0.48 | 11 | 2.53 |
| 0.74 | 1.3 | 0.82 | 0.84 | 1.67 | 1.66 | 1.25 | 1.24 | 0.8 | 5.1 | 1.84 |
| 1.89 | 0.92 | 1.71 | 1.08 | 1.02 | 1.88 | 0.93 | 1.31 | 0.96 | 4.35 | 1.33 |
| 1.4 | 3.94 | 1.21 | 0.21 | 6.3 | 2.16 | 1.06 | 0.89 | 2.83 | 1.51 | 1.04 |
| 1.12 | 1.68 | 0.87 | 0.83 | 0.77 | 1.67 | 1.19 | 1.76 | 1.72 | 5.4 | 2.14 |
| 0.75 | 1.5 | 0.99 | 1.45 | 4.58 | 0.9 | 1.69 | 0.88 | 0.38 | 2.28 | 1.22 |
| 1.67 | 1.1 | 0.86 | 2.32 | 1.01 | 2.57 | 0.87 | 1.27 | 4.03 | 1.07 | 1.12 |
| 1.5 | 0.79 | 0.72 | 1.08 | 0.7 | 1.79 | 1.12 | 2.16 | 2.28 | 6.9 | 0.72 |
| 0.72 | 0.73 | 0.76 | 0.83 | 1.4 | 1.54 | 1 | 2.33 | 1.09 | 2.73 | 0.4 |
| 4.73 | 0.84 | 1.22 | 0.87 | 1 | 1.15 | 1.51 | 5 | 2.16 | 1.29 | 1.05 |
| 3 | 0.87 | 5.27 | 2 | 0.96 | 1.5 | 0.83 | 0.94 | 1.07 | 0.68 | 1.17 |
| 0.28 | 0.77 | 1.09 | 2.83 | 1.82 | 0.68 | 0.02 | 2.77 | 0.35 | 0.93 | 2.07 |
| 1.1 | 3.49 | 0.96 | 1.52 | 2.9 | 0.94 | 1.16 | 0.62 | 1.28 | 0.7 | 1.01 |
| 0.5 | 1.32 | 0.91 | 0.76 | 1.41 | 1.43 | 1.04 | 1.55 | 2.15 | 0.7 | 8.88 |
| 3 | 1.17 | 1.16 | 1.52 | 1.25 | 0.5 | 1.06 | 0.93 | 1.25 | 0.53 | 0.92 |
| 0.74 | 2.36 | 2.52 | 1.13 | 4.54 | 1.81 | 0.83 | 2.39 | 0.02 | 0.7 | 1.13 |
| 0.67 | 1.45 | 6.73 | 0.79 | 0.75 | 0.2 | 1.73 | 1.17 | 1.57 | 0.84 | 0.77 |
| 6.67 | 3.18 | 1.86 | 0.53 | 1.24 | 1.53 | 1 | 1.76 | 1.24 | 1.38 | 1.39 |
| 1.39 | 1.07 | 1.03 | 1.06 | 5.66 | 0.97 | 5.14 | 0.87 | 3.24 | 0.97 | 0.94 |
| 0.59 | 1.81 | 0.28 | Inf | 2.31 | 0.56 | 1.44 | 0.81 | 6.31 | 0.88 | 0.97 |
| 0.89 | 0.84 | 2.46 | 1.02 | 1.6 | 1.87 | 1.4 | 0.84 | 1.27 | 2.17 | 0.34 |
| 0.5 | 0.76 | 0.61 | 0.6 | 0.57 | 2 | 1.18 | 0.66 | 1.4 | 0.18 | 0.77 |
| 1.33 | 1.32 | 2.98 | 0.8 | 1.32 | 2.91 | 18.14 | 1.09 | 1.2 | 3.79 | 0.35 |

| | | | | | | | | | | |
|---|---|---|---|---|---|---|---|---|---|---|
| 7065.75 | 0.83 | 0.7 | 0.91 | 0.85 | 1.29 | 0.85 | 1.41 | 0.62 | 2.08 | 0.72 |
| 2 | 0.93 | 0.71 | 0.99 | 2.25 | 0.26 | 0.36 | 0.67 | 4.3 | 5.67 | 1.16 |
| 2.28 | 1.5 | 2.45 | 0.26 | 1.54 | 4.78 | 1.21 | 0.81 | 1.53 | 2.81 | 1.07 |
| 0.52 | 2.27 | 1.56 | 3.38 | 0.67 | 0.47 | 0.96 | 2.05 | 1.06 | 1.26 | 1.26 |
| 3.01 | 1.41 | 1.09 | 2.41 | 2.01 | 2.01 | 0.79 | 0.84 | 2.16 | 1.13 | 1.88 |
| 0.71 | 0.96 | 0.88 | 1.33 | 1 | 0.55 | 3.89 | 0.45 | 0.85 | 1.06 | 0.99 |
| 1.15 | 1.25 | 0.48 | 0.96 | 0.4 | 0.58 | 0.99 | 0.77 | 1.38 | 0.3 | 1.34 |
| 0.46 | 1.55 | 3.14 | 0.2 | 0.57 | 7.86 | 1.26 | 0.65 | 1.37 | 1.59 | 1.08 |
| 0.64 | 0.69 | 1.03 | 0.97 | 1.16 | 2.61 | 1.59 | 2.04 | 0.25 | 0.99 | 5.73 |
| 1.31 | 1.48 | 0.49 | 1.37 | 1.53 | 1.39 | 0.65 | 2.26 | 1.23 | 0.77 | 1.46 |
| 0.33 | 1.03 | 1.33 | 1.08 | 1.19 | 0.14 | 1.13 | 0.38 | 2 | 0.3 | 1.13 |
| 0.33 | 1.1 | 5.27 | 1.39 | 1.37 | 0.6 | 0.71 | 0.57 | 0.4 | 0.88 | 1.28 |
| 1 | 0.29 | 0.43 | 0.46 | 0.58 | 0.5 | 1.73 | 1.5 | 0.34 | 1.12 | 0.86 |
| 1.08 | 0.53 | 0.43 | 4.06 | 3.99 | 0.5 | 1.46 | 0.96 | 0.61 | 0.81 | 0.11 |
| 1.25 | 1.91 | 0.67 | 4.96 | 2.25 | 4.81 | 1.57 | 0.77 | 0.77 | 5.97 | 0.83 |
| 1.43 | 0.71 | 2.96 | 0.96 | 0.9 | 0.65 | 0.91 | 1.08 | 0.75 | 2.41 | 1.38 |
| 0.67 | 0.9 | 1.15 | 1.08 | 0.75 | 26 | 1.02 | 0.88 | 1.54 | 0.39 | 0.95 |
| 0.6 | 1.3 | 1.23 | 4.86 | 3.86 | 1.51 | 1.02 | 1.34 | 0.7 | 1.29 | 4.4 |
| 1.29 | 0.96 | 6.79 | 1.18 | 2.62 | 16.58 | 8.51 | 3.65 | 2.14 | 4.33 | 0.61 |
| 0.44 | 3.35 | 0.53 | 4.42 | 5.5 | 1.36 | 0.79 | 1.7 | 0.01 | 2.31 | 101.77 |
| 0.77 | 0.97 | 2.23 | 1.48 | 7.44 | 4.35 | 1.55 | 2.04 | 2.36 | 0.6 | 1.34 |
| 1.48 | 0.99 | 0.81 | 0.85 | 1.23 | 2.31 | 0.62 | 1.15 | 0.31 | 0.96 | 0.62 |
| 0.93 | 0.3 | 1.3 | 0.12 | 1.22 | 1.39 | 0.79 | 4 | 1.56 | 1.79 | 0.31 |
| 1.05 | 0.78 | 1.37 | 1.09 | 0.25 | 2.36 | 0.95 | 1.83 | 0.35 | 1.34 | 0.39 |
| 3.29 | 0.62 | 0.71 | 1.19 | 0.57 | 0.92 | 1.07 | 1.64 | 0.84 | 1.09 | 1.03 |
| 2.61 | 1.06 | 2.16 | 0.79 | 0.63 | 1.85 | 1.5 | 1.78 | 0.88 | 2.21 | 0.57 |
| 0.68 | 1.79 | 1.1 | 0.76 | 0.65 | 1.54 | 0.99 | 2.52 | 2.65 | 0.56 | 0.85 |
| 0.75 | 1.75 | 1.13 | 1.62 | 0.98 | 1.29 | 1.26 | 3.01 | 0.26 | 4.64 | 3.5 |
| 26.91 | 1.12 | 4.43 | 0.59 | 1.55 | 0.82 | 1.54 | 2.38 | 2.01 | 3.24 | 0.54 |
| 0.14 | 1.34 | 0.2 | 1.97 | 5.36 | 0.33 | 3.73 | 0.93 | 0.55 | 1.33 | 1.37 |
| 2 | 0.99 | 2.69 | 1.15 | 0.78 | 1.14 | 0.33 | 0.26 | 3.67 | 0.06 | 0.86 |
| 1.62 | 3.84 | 2.05 | 0.42 | 1.5 | 1.49 | 1.95 | 0.74 | 0.78 | 0.51 | 1.09 |
| 1.26 | 1.58 | 6.11 | 3.11 | 0.3 | 2.83 | 2.06 | 1.1 | 0.71 | 0.27 | 1.03 |

| | | | | | | | | | | |
|---|---|---|---|---|---|---|---|---|---|---|
| 0.33 | 0.84 | 0.73 | 1.05 | 0.84 | 0.97 | 1 | 1.19 | 0.56 | 1.11 | 1.17 |
| 1.33 | 1.33 | 1.25 | 1.89 | 1.44 | 1.58 | 1.04 | 0.84 | 1.04 | 0.5 | 1.24 |
| 2 | 2.67 | 3.18 | 10.28 | 1.47 | 0.63 | 6.51 | 1.69 | 0.32 | 2.5 | 1.29 |
| 3.46 | 0.68 | 0.52 | 0.46 | 0.55 | 1.04 | 0.54 | 0.67 | 6.15 | 6.25 | 0.51 |
| 1.07 | 0.89 | 0.56 | 0.17 | 0.71 | 3.32 | 0.89 | 1 | 2.02 | 4.49 | 0.31 |
| 1.13 | 1.31 | 0.75 | 1.16 | 0.76 | 1.89 | 2.25 | 3.12 | 3.36 | 1.44 | 0.79 |
| 1.54 | 1.09 | 0.93 | 0.86 | 0.9 | 1.52 | 1.12 | 1.47 | 1.31 | 3.23 | 1.18 |
| 1.33 | 0.71 | 1.07 | 0.79 | 0.97 | 0.69 | 0.8 | 0.62 | 6.73 | 3.66 | 1.46 |
| 1.5 | 0.58 | 0.64 | 1.28 | 1.22 | 1.18 | 1.82 | 0.56 | 1.16 | 0.85 | 2.76 |
| 3.38 | 1.7 | 1.42 | 0.96 | 3.92 | 2.02 | 0.89 | 4.09 | 1.78 | 1.9 | 1.31 |
| 7 | 1.04 | 1.81 | 0.33 | 0.99 | 2.74 | 1.8 | 1.62 | 3.44 | 1.57 | 1.28 |
| 1.19 | 46.26 | 0.31 | 13.11 | 10.6 | 0.95 | 0.16 | 0.72 | 0.59 | 2.12 | 0.48 |
| 0.61 | 0.87 | 4.01 | 3.19 | 1.31 | 1.38 | 0.93 | 0.89 | 0.7 | 0.51 | 0.97 |
| 7 | 1.84 | 0.43 | 1.49 | 1.42 | 0.11 | 0.33 | 1.2 | 3 | 1.35 | 0.41 |

| 207397_s_at | 210973_s_at | 213844_at | 207965_at | 212983_at | 213721_at | 206044_s_at | 207233_s_at | 201839_s_at | 212983_at | 219480_at |
|---|---|---|---|---|---|---|---|---|---|---|
| 2.89 | 0.96 | 0.4 | 0.87 | 1.29 | 1.69 | 1.57 | 0.86 | 0.19 | 1.29 | 1.93 |
| 1.11 | 1.91 | 0.97 | 1.75 | 0.97 | 0.84 | 0.91 | 1.35 | 1.76 | 0.97 | 1.49 |
| 1.72 | 1.41 | 1.99 | 1.24 | 1.51 | 3.91 | 1.28 | 2.37 | 2.6 | 1.51 | 0.99 |
| 3.03 | 1.53 | 1.46 | 3.15 | 1.07 | 2.09 | 1.23 | 1.07 | 0.88 | 1.07 | 3.82 |
| 1.19 | 0.73 | 0.73 | 2.32 | 0.88 | 2.75 | 0.93 | 0.65 | 2.29 | 0.88 | 0.51 |
| 1.24 | 1.79 | 1.79 | 4.07 | 0.83 | 1.25 | 1.54 | 3.28 | 1.51 | 0.83 | 1.05 |
| 1 | 0.44 | 0.67 | 1.37 | 2.06 | 2.13 | 1.07 | 0.35 | 0.04 | 2.06 | 2.33 |
| 0.62 | 4.14 | 1.47 | 3.8 | 1.04 | 0.87 | 0.9 | 0.74 | 1.19 | 1.04 | 1.75 |
| 1.87 | 1.45 | 1.39 | 1.82 | 1.1 | 0.33 | 1.29 | 1.54 | 1.58 | 1.1 | 1.07 |
| 13.89 | 1.21 | 0.66 | 0.77 | 1 | 1.27 | 0.69 | 0.96 | 0.8 | 1 | 10.32 |
| 0.72 | 1.06 | 2.8 | 0.29 | 0.91 | 1.03 | 1.14 | 1.59 | 0.25 | 0.91 | 1.06 |
| 1.03 | 0.98 | 0.91 | 1.69 | 1.44 | 1.52 | 1.24 | 1.62 | 0.88 | 1.44 | 1.49 |
| 0.97 | 0.95 | 1.34 | 0.41 | 1.18 | 0.88 | 0.92 | 1.49 | 1 | 1.18 | 1.06 |
| 7.67 | 3.62 | 27.85 | 6.5 | 0.74 | 60.13 | 10.29 | 7.91 | 0.01 | 0.74 | 0.68 |
| 3.83 | 1.47 | 1.59 | 1.53 | 0.96 | 0.66 | 1.35 | 0.71 | 1.15 | 0.96 | 1.06 |
| 1.51 | 1.57 | 0.96 | 5.81 | 0.99 | 2.16 | 1.14 | 0.69 | 1.38 | 0.99 | 1.1 |
| 1.56 | 1.47 | 1.17 | 1.49 | 1.13 | 1.77 | 0.73 | 0.93 | 0.77 | 1.13 | 0.6 |
| 4.82 | 2.06 | 1.15 | 2.06 | 1.07 | 3.17 | 2.04 | 1.45 | 2.65 | 1.07 | 3.94 |
| 0.33 | 0.27 | 1.1 | 0.22 | 1.88 | 2.73 | 1.54 | 0.94 | 0.93 | 1.88 | 1.31 |
| 5.06 | 1.97 | 1.91 | 2.18 | 1.24 | 1.7 | 0.84 | 2.85 | 0.56 | 1.24 | 4.67 |
| 0.52 | 1.01 | 1.33 | 2.68 | 2.52 | 0.83 | 0.65 | 1.38 | 0.47 | 2.52 | 0.83 |
| Inf | 14.89 | 0.54 | 7.75 | 0.92 | 4.55 | 2.69 | 0.74 | 0.66 | 0.92 | 9.25 |
| 5.67 | 0.97 | 0.77 | 2.37 | 0.95 | 1.13 | 0.85 | 0.89 | 1.09 | 0.95 | 0.84 |
| 2.2 | 7.64 | 1.24 | 2 | 0.71 | 4.57 | 0.73 | 0.82 | 0.01 | 0.71 | 1.05 |
| 0.35 | 0.63 | 0.11 | 3.26 | 0.84 | 0.11 | 0.61 | 0.48 | 0.01 | 0.84 | 2.05 |
| 0.03 | 1.23 | 2.14 | 0.51 | 0.84 | 0.63 | 1.13 | 1.07 | 0.97 | 0.84 | 2.99 |
| 1.05 | 0.72 | 0.82 | 4.12 | 0.98 | 2.91 | 1.05 | 1.07 | 3.85 | 0.98 | 0.27 |
| 1.94 | 6.16 | 3.68 | 8.59 | 0.61 | 1.54 | 2.16 | 1.01 | 0.36 | 0.61 | 7.43 |
| 1.01 | 1.84 | 0.5 | 2.12 | 1.34 | 1.23 | 1.14 | 4.13 | 8.05 | 1.34 | 1.56 |
| 29.34 | 1.25 | 0.94 | 4.08 | 1.28 | 1.18 | 1.23 | 1.4 | 0.85 | 1.28 | 8.23 |

| | | | | | | | | | | |
|---|---|---|---|---|---|---|---|---|---|---|
| 2.17 | 25.57 | 0.34 | 3.75 | 1.54 | 3.01 | 1.35 | 0.84 | 0.89 | 1.54 | 3.57 |
| 10.77 | 0.39 | 23.15 | 0.33 | 1.69 | 0.43 | 2.16 | 4.22 | 1.6 | 1.69 | 1.3 |
| 1.15 | 1.93 | 1.55 | 22 | 1.11 | 5.77 | 1.67 | 0.95 | 0.94 | 1.11 | 1.31 |
| 2.43 | 0.91 | 0.51 | 5.84 | 21.77 | 1.45 | 1.27 | 0.54 | 9.39 | 21.77 | 4.38 |
| 0.57 | 0.79 | 2.03 | 1 | 1.9 | 16.17 | 1.84 | 0.77 | 1.18 | 1.9 | 6.76 |
| 3.62 | 5.09 | 1.24 | 19.92 | 2.45 | 7.59 | 12.81 | 0.43 | 1.75 | 2.45 | 6.79 |
| 1.23 | 1.21 | 3.87 | 5.81 | 0.47 | 1.75 | 36.97 | 10.83 | 3.68 | 0.47 | 1.99 |
| 0.67 | 0.9 | 0.94 | 3 | 1.01 | 0.78 | 1.12 | 1.5 | 10.58 | 1.01 | 1.22 |
| Inf | 1.95 | 0.67 | 0.86 | 10.21 | 3.18 | 1.1 | 1.23 | 0.93 | 10.21 | 2.9 |
| 2 | 5.66 | 0.5 | 0.75 | 0.74 | 0.17 | 0.59 | 2.44 | 0.42 | 0.74 | 9.96 |
| 11.4 | 1.49 | 0.85 | 2.55 | 1.72 | 1.1 | 0.9 | 0.84 | 2.38 | 1.72 | 0.88 |
| 6 | 1.34 | 0.31 | 8.16 | 1.04 | 0.58 | 1.23 | 0.82 | 0.9 | 1.04 | 1.37 |
| 0.83 | 0.97 | 0.76 | 0.35 | 8.05 | 0.93 | 1.17 | 1.24 | 2.27 | 8.05 | 1.19 |
| 9 | 0.28 | 0.59 | 0.95 | 1.74 | 5.62 | 1.87 | 1.16 | 2.09 | 1.74 | 1.3 |
| 1 | 0.93 | 0.78 | 0.42 | 5.64 | 1.44 | 1.16 | 1.11 | 2.17 | 5.64 | 0.89 |
| 2.04 | 1.8 | 1.05 | 3.44 | 1.01 | 1 | 1.02 | 0.89 | 11.21 | 1.01 | 2.6 |
| 0.5 | 0.55 | 1.14 | 2.25 | 1.14 | 1.4 | 0.42 | 1.37 | 1.03 | 1.14 | 0.43 |
| 5.11 | 1.39 | 0.79 | 3.15 | 0.96 | 5.15 | 1.1 | 1.01 | 3.32 | 0.96 | 1.45 |
| 0.76 | 1.15 | 1.05 | 0.66 | 1.06 | 0.83 | 1.06 | 1.26 | 0.96 | 1.06 | 2.69 |
| 3.13 | 1.29 | 0.54 | 2.47 | 1.13 | 0.83 | 0.6 | 0.91 | 3.38 | 1.13 | 6.3 |
| 1 | 0.54 | 1.12 | 1.75 | 0.85 | 0.48 | 0.69 | 0.86 | 1.08 | 0.85 | 0.28 |
| 0.87 | 0.92 | 1.37 | 2.76 | 1.07 | 5.55 | 0.9 | 0.61 | 0.96 | 1.07 | 0.69 |
| 2.23 | 0.93 | 2.78 | 1.64 | 0.97 | 0.75 | 0.76 | 1.4 | 0.96 | 0.97 | 1.51 |
| 0.85 | 1.32 | 2.83 | 2.23 | 1.25 | 0.91 | 1.07 | 2.08 | 0.97 | 1.25 | 2.19 |
| 1.38 | 0.36 | 0.19 | 0.97 | 4.5 | 1.15 | 0.98 | 0.51 | 1.04 | 4.5 | 1.05 |
| 3 | 1.34 | 1.42 | 0.35 | 1.26 | 1.21 | 1.6 | 0.77 | 4.02 | 1.26 | 1.79 |
| 0.14 | 2.99 | 0.43 | 5.19 | 1.73 | 0.23 | 0.81 | 0.05 | 3.75 | 1.73 | 1.26 |
| 0.25 | 1.05 | 1.46 | 1.61 | 0.73 | 1.59 | 1.5 | 1 | 74.56 | 0.73 | 0.85 |
| 1 | 0.9 | 0.85 | 1.05 | 0.8 | 0.95 | 1.34 | 1.12 | 1.01 | 0.8 | 1.7 |
| 1.5 | 0.85 | 1.21 | 0.37 | 4.35 | 1.25 | 1.42 | 1.29 | 2.38 | 4.35 | 3.39 |
| 2.66 | 1.57 | 1.67 | 1.64 | 1.27 | 0.34 | 0.89 | 2.29 | 1.31 | 1.27 | 2.7 |
| Inf | 0.52 | 0.82 | 0.76 | 0.73 | 2.01 | 0.64 | 0.86 | 1.2 | 0.73 | 1.31 |
| 0.93 | 0.76 | 1.23 | 0.74 | 0.83 | 0.53 | 1.22 | 0.98 | 7.57 | 0.83 | 0.92 |

| | | | | | | | | | | |
|---|---|---|---|---|---|---|---|---|---|---|
| 0.16 | 1.09 | 1.03 | 1.13 | 1.25 | 11.25 | 1.14 | 1.05 | 0.75 | 1.25 | 0.73 |
| 28.88 | 0.75 | 0.8 | 1.68 | 1.51 | 10.14 | 1.02 | 0.77 | 1.17 | 1.51 | 15.06 |
| 0.26 | 0.93 | 1.26 | 1.5 | 0.98 | 1.1 | 0.97 | 3.23 | 0.79 | 0.98 | 5.21 |
| 1.48 | 1.44 | 1.7 | 1.08 | 0.99 | 2.26 | 1.19 | 1.8 | 7.95 | 0.99 | 2.72 |
| 1.09 | 1.37 | 1 | 1.48 | 0.89 | 1.16 | 1.06 | 1.22 | 1.16 | 0.89 | 1.1 |
| 2.65 | 0.41 | 2.14 | 1.5 | 1.38 | 3.38 | 1.06 | 1.62 | 2.3 | 1.38 | 1.75 |
| 1.33 | 1.58 | 2.23 | 2.05 | 1.06 | 0.66 | 1.81 | 2.55 | 0.64 | 1.06 | 0.82 |
| 1.67 | 3.87 | 0.88 | 3.47 | 1.63 | 0.68 | 1.26 | 0.93 | 0.78 | 1.63 | 1.36 |
| 1.63 | 1.23 | 0.73 | 0.72 | 1.36 | 1.42 | 0.74 | 1.34 | 1.82 | 1.36 | 0.42 |
| 0.85 | 0.45 | 1.16 | 2.15 | 0.78 | 1.22 | 0.7 | 1.19 | 1.3 | 0.78 | 0.79 |
| 0.22 | 2.13 | 12.19 | 8.41 | 0.37 | 1.04 | 2.06 | 1.61 | 1.99 | 0.37 | 0.46 |
| 0.63 | 1.31 | 1.15 | 5 | 1.03 | 0.72 | 1.05 | 0.97 | 0.54 | 1.03 | 1.67 |
| 2.75 | 1.56 | 0.84 | 3 | 0.98 | 1.19 | 1.99 | 1.61 | 0.68 | 0.98 | 1.07 |
| 1.78 | 1.11 | 1.53 | 2.76 | 1.11 | 2.85 | 0.87 | 6.96 | 1.06 | 1.11 | 1.64 |
| 1 | 0.27 | 1.32 | 0.57 | 1.88 | 0.12 | 1.04 | 1.12 | 1.07 | 1.88 | 0.23 |
| 0.98 | 0.08 | 0.1 | 0.04 | 2.17 | 0.01 | 0.05 | 1.59 | 1.05 | 2.17 | 0.1 |
| 0.62 | 1.52 | 1.84 | 0.79 | 0.6 | 3.04 | 0.85 | 8.77 | 0.92 | 0.6 | 1.32 |
| 8 | 0.07 | 1.55 | 0.39 | 0.84 | 1.37 | 1.22 | 0.78 | 2.07 | 0.84 | 0.09 |
| 1.3 | 0.88 | 1.11 | 1.83 | 0.85 | 1.25 | 1.22 | 0.92 | 1.26 | 0.85 | 1.72 |
| 3.98 | 2.6 | 3.04 | 13.29 | 1.88 | 1.35 | 1.83 | 1.15 | 1.3 | 1.88 | 3.14 |
| 1.1 | 1.33 | 3.15 | 1.23 | 1.08 | 1.87 | 0.68 | 0.19 | 84.39 | 1.08 | 3.12 |
| 5.07 | 1.12 | 1.19 | 1.97 | 1.24 | 1.19 | 0.91 | 0.88 | 1 | 1.24 | 2.87 |
| 1 | 2.41 | 0.66 | 0.76 | 1.6 | 1.71 | 1.29 | 0.59 | 1.97 | 1.6 | 1.67 |
| 2.53 | 2.46 | 1.16 | 1.24 | 1.52 | 1.87 | 0.85 | 0.79 | 1.11 | 1.52 | 0.91 |
| NA | 1.26 | 4.49 | 1.31 | 1.14 | 1.25 | 1.3 | 0.7 | 1 | 1.14 | 0.33 |
| 1 | 1.05 | 1.23 | 0.35 | 0.9 | 0.41 | 1.08 | 0.88 | 0.74 | 0.9 | 2.8 |
| 1.34 | 0.98 | 1.01 | 2.17 | 1.17 | 0.94 | 1.1 | 0.76 | 0.82 | 1.17 | 2.58 |
| 2.5 | 0.76 | 0.12 | 4.74 | 1.34 | 0.4 | 0.94 | 0.9 | 0.82 | 1.34 | 3.05 |
| 1.33 | 0.86 | 0.62 | 2.84 | 0.89 | 0.76 | 1.08 | 1.26 | 0.91 | 0.89 | 2.41 |
| 0.78 | 0.6 | 0.82 | 0.8 | 1.07 | 1.2 | 0.8 | 1.03 | 1.05 | 1.07 | 0.84 |
| 1.08 | 1.21 | 1.21 | 1.79 | 1.25 | 1.21 | 1.02 | 1.02 | 1.25 | 1.25 | 8.58 |
| 0.41 | 0.49 | 0.55 | 0.91 | 1.85 | 1.36 | 0.75 | 0.96 | 0.7 | 1.85 | 3.55 |
| 0.75 | 0.98 | 0.91 | 0.25 | 0.91 | 1.74 | 1.14 | 4.02 | 0.83 | 0.91 | 0.47 |

| | | | | | | | | | | |
|---|---|---|---|---|---|---|---|---|---|---|
| 1.44 | 1.82 | 2.45 | 3.1 | 0.69 | 1.46 | 1.29 | 1.18 | 1.83 | 0.69 | 1.28 |
| 2.24 | 1.79 | 1.04 | 1.87 | 1.23 | 1.48 | 2.02 | 0.31 | 3.1 | 1.23 | 1.95 |
| 1.5 | 2.5 | 1.27 | 0.66 | 1.26 | 2.27 | 1.2 | 1.49 | 1.01 | 1.26 | 0.58 |
| 1.77 | 1.92 | 0.78 | 1.18 | 1.5 | 2.86 | 0.78 | 1.87 | 1.06 | 1.5 | 1.25 |
| 3.88 | 1.1 | 1.16 | 0.55 | 0.9 | 1.26 | 1.23 | 2.35 | 0.44 | 0.9 | 1.42 |
| 0.79 | 0.4 | 0.45 | 1.81 | 1.74 | 3.17 | 0.7 | 0.36 | 1.06 | 1.74 | 1.15 |
| 1.77 | 1.07 | 0.79 | 2.86 | 1.2 | 0.53 | 2.06 | 1.14 | 1.89 | 1.2 | 1.11 |
| 0.45 | 0.36 | 1.31 | 0.33 | 1.04 | 3.89 | 1.05 | 1.84 | 1.53 | 1.04 | 0.79 |
| 2.7 | 1.26 | 0.94 | 5.25 | 0.73 | 0.44 | 1.04 | 1.2 | 0.65 | 0.73 | 4.65 |
| 1.62 | 1.58 | 4.29 | 1.03 | 0.79 | 1.52 | 1.06 | 1.08 | 0.9 | 0.79 | 1.22 |
| 0.84 | 1.6 | 0.99 | 2.15 | 1.17 | 1.76 | 0.94 | 0.65 | 0.96 | 1.17 | 1.08 |
| 1.17 | 1.65 | 1.07 | 1.29 | 1.04 | 1.72 | 0.98 | 2.43 | 1.04 | 1.04 | 1.58 |
| 9.83 | 1.9 | 1.02 | 2.4 | 1.18 | 0.79 | 2.26 | 1.44 | 1.42 | 1.18 | 1.52 |
| 1.17 | 1.18 | 0.74 | 3.09 | 14.7 | 0.72 | 1.07 | 0.5 | 5.43 | 14.7 | 2.72 |
| 0.56 | 1.48 | 0.58 | 1.09 | 0.9 | 0.48 | 1.22 | 1.11 | 1.56 | 0.9 | 0.91 |
| 1.48 | 1.66 | 4.46 | 3.08 | 1.06 | 1.68 | 0.87 | 0.84 | 1.27 | 1.06 | 0.89 |
| 14 | 1.17 | 0.86 | 0.39 | 0.94 | 0.91 | 0.97 | 2.07 | 0.76 | 0.94 | 0.55 |
| 1.48 | 1.5 | 2.37 | 1.14 | 1.12 | 2.53 | 1.07 | 1.77 | 7.91 | 1.12 | 2.49 |
| 0.83 | 1.3 | 0.95 | 0.99 | 0.85 | 1.48 | 1.13 | 1.65 | 3.19 | 0.85 | 3.79 |
| 0.25 | 0.95 | 1.02 | 2.5 | 1.4 | 0.45 | 1.15 | 0.92 | 1.09 | 1.4 | 8.53 |
| 0.17 | 2.32 | 0.93 | 0.86 | 0.9 | 2.19 | 1.19 | 0.95 | 1.58 | 0.9 | 1.09 |
| 1.02 | 1.55 | 1.05 | 0.72 | 0.96 | 3.25 | 0.9 | 0.48 | 1.38 | 0.96 | 1.25 |
| 1.17 | 1.17 | 0.99 | 1.72 | 0.95 | 1.97 | 0.86 | 1.21 | 1.02 | 0.95 | 1.12 |
| 2 | 1.65 | 0.91 | 0.19 | 0.82 | 0.16 | 0.6 | 0.88 | 0.96 | 0.82 | 1.11 |
| 0.75 | 0.86 | 0.84 | 1.84 | 0.99 | 1.87 | 2.11 | 1.41 | 1.1 | 0.99 | 2.41 |
| 1.53 | 0.67 | 0.7 | 1.63 | 0.8 | 0.78 | 1.41 | 1.27 | 0.89 | 0.8 | 1.27 |
| 0.25 | 1.26 | 0.9 | 0.8 | 1.13 | 0.49 | 1.3 | 1.54 | 1.09 | 1.13 | 1 |
| 1.33 | 1.18 | 0.88 | 0.66 | 0.57 | 1.06 | 0.79 | 0.82 | 0.96 | 0.57 | 0.7 |
| 0.22 | 3.06 | 0.81 | 1.38 | 0.93 | 3.62 | 1.11 | 0.76 | 3.57 | 0.93 | 4.15 |
| 1.85 | 1.92 | 0.67 | 1.04 | 0.94 | 1.49 | 0.87 | 0.89 | 0.74 | 0.94 | 2.55 |
| 0.77 | 0.99 | 0.92 | 2.12 | 1.11 | 0.6 | 1.3 | 1.68 | 1.04 | 1.11 | 1.29 |
| 28.88 | 0.75 | 0.8 | 1.68 | 1.51 | 10.14 | 1.02 | 0.77 | 1.17 | 1.51 | 15.06 |
| 1.5 | 0.85 | 0.74 | 4.25 | 0.9 | 1.54 | 1.02 | 1.42 | 0.96 | 0.9 | 0.18 |

| | | | | | | | | | | |
|---|---|---|---|---|---|---|---|---|---|---|
| 1.27 | 1.1 | 0.46 | 2.22 | 1 | 0.98 | 0.78 | 1.15 | 0.85 | 1 | 2.31 |
| 3.11 | 1.22 | 1.12 | 3.5 | 0.92 | 0.74 | 1.17 | 2.19 | 0.95 | 0.92 | 0.53 |
| 5.83 | 1.04 | 0.63 | 4.02 | 1.13 | 4.66 | 1.1 | 1.45 | 0.93 | 1.13 | 1.48 |
| 0.78 | 1.05 | 1.5 | 3.4 | 1.01 | 0.68 | 1.05 | 1.29 | 1.53 | 1.01 | 0.82 |
| 1.13 | 0.87 | 0.81 | 1.3 | 1.04 | 1.23 | 1.22 | 1.34 | 0.67 | 1.04 | 3.58 |
| 1.31 | 0.54 | 1.17 | 9 | 0.92 | 0.44 | 1.14 | 0.78 | 1.25 | 0.92 | 0.86 |
| 0.27 | 0.81 | 0.82 | 4.48 | 0.9 | 4.08 | 0.56 | 0.85 | 1.08 | 0.9 | 1.95 |
| 0.7 | 0.29 | 1.05 | 1.04 | 0.75 | 3.47 | 1.41 | 0.77 | 1.14 | 0.75 | 0.25 |
| 1.97 | 0.72 | 1.96 | 3.57 | 1.01 | 1.2 | 1.71 | 1.81 | 0.55 | 1.01 | 2.44 |
| 0.23 | 1.14 | 1.11 | 1.71 | 1.28 | 0.71 | 0.91 | 3.93 | 1.58 | 1.28 | 5.64 |
| 0.67 | 0.87 | 1.23 | 2.1 | 1.1 | 1.51 | 0.93 | 1.05 | 0.6 | 1.1 | 0.89 |
| 2.66 | 0.87 | 1.13 | 1.71 | 1.26 | 0.62 | 1.06 | 1.35 | 0.89 | 1.26 | 1.99 |
| 1.62 | 1.59 | 1.58 | 1.54 | 1.05 | 1.59 | 1.76 | 1.79 | 8.2 | 1.05 | 1.64 |
| 0.56 | 0.75 | 1.65 | 1.52 | 0.89 | 0.71 | 1.8 | 0.94 | 0.58 | 0.89 | 7.78 |
| 1 | 0.28 | 0.94 | 1.94 | 0.84 | 2.04 | 1.08 | 2.95 | 0.65 | 0.84 | 0.7 |
| 1.03 | 1.3 | 1.05 | 0.93 | 1.05 | 0.66 | 0.97 | 0.89 | 2.07 | 1.05 | 3.95 |
| 2.4 | 0.9 | 0.85 | 1.75 | 1.1 | 5.77 | 1.04 | 1.17 | 0.98 | 1.1 | 1.09 |
| 1.71 | 0.86 | 2.16 | 2.37 | 1.19 | 1.31 | 1 | 1.47 | 2.02 | 1.19 | 3.78 |
| 0.95 | 0.95 | 0.94 | 1.92 | 0.83 | 2.44 | 1.07 | 0.83 | 0.69 | 0.83 | 0.62 |
| 2.44 | 1.17 | 2.12 | 1.11 | 1.07 | 0.73 | 1.08 | 1.77 | 0.96 | 1.07 | 1.16 |
| 2.98 | 1.05 | 1.1 | 1.42 | 1.12 | 0.63 | 0.71 | 0.83 | 0.92 | 1.12 | 4.86 |
| NA | 2.84 | 0.86 | 1.13 | 0.66 | 3.13 | 1.57 | 0.64 | 1.28 | 0.66 | 6.94 |
| 4.52 | 1.02 | 4.21 | 2.13 | 1.35 | 0.76 | 1.05 | 0.8 | 1.2 | 1.35 | 1.92 |
| 1.38 | 1.3 | 1.44 | 1.94 | 0.92 | 1.6 | 1.04 | 0.7 | 1.16 | 0.92 | 1.11 |
| 0.33 | 1.05 | 0.98 | 1.99 | 2.54 | 0.9 | 1 | 0.93 | 1.31 | 2.54 | 0.92 |
| 1.23 | 0.8 | 0.62 | 3.22 | 1.42 | 2.2 | 0.83 | 1.05 | 0.9 | 1.42 | 0.86 |
| 0.83 | 2.76 | 1.4 | 1.44 | 1.28 | 1.46 | 0.82 | 1.71 | 1.47 | 1.28 | 2.59 |
| 1.06 | 1 | 1.26 | 3.15 | 1.06 | 4.8 | 0.77 | 1.4 | 0.93 | 1.06 | 4.77 |
| 1.22 | 1.05 | 0.79 | 2.83 | 1.03 | 1.01 | 0.98 | 1.23 | 0.85 | 1.03 | 0.45 |
| 1.04 | 1.1 | 0.89 | 2 | 0.98 | 0.98 | 0.93 | 0.93 | 0.3 | 0.98 | 0.74 |
| 0.77 | 1.01 | 0.81 | 2.11 | 1.14 | 0.7 | 1.05 | 1.41 | 1.35 | 1.14 | 2.93 |
| NA | 1.16 | 0.87 | 2.17 | 1.01 | 2.52 | 0.91 | 1.11 | 1.01 | 1.01 | 0.85 |
| 1.5 | 1.21 | 0.97 | 1.06 | 1.12 | 1.07 | 0.92 | 0.86 | 2.56 | 1.12 | 1 |

| | | | | | | | | | | |
|---|---|---|---|---|---|---|---|---|---|---|
| 0.57 | 1.27 | 0.84 | 0.83 | 1.86 | 3.06 | 0.66 | 2.05 | 1.61 | 1.86 | 0.76 |
| 3.19 | 0.69 | 0.81 | 2.26 | 1.31 | 0.72 | 0.96 | 1.26 | 1.59 | 1.31 | 1.32 |
| 1 | 1.33 | 0.93 | 0.6 | 1 | 1.1 | 0.98 | 1.1 | 1.42 | 1 | 1.39 |
| 2.62 | 2.02 | 1 | 2.37 | 0.53 | 0.39 | 0.92 | 1.3 | 0.96 | 0.53 | 1.56 |
| 23 | 0.59 | 0.88 | 0.29 | 1.13 | 0.55 | 1.4 | 1.59 | 1.43 | 1.13 | 1.8 |
| 1.76 | 1.12 | 0.9 | 1.25 | 1.08 | 1.25 | 1.11 | 0.89 | 1.76 | 1.08 | 1.03 |
| 3 | 14.96 | 0.24 | 3.37 | 1.55 | 3.3 | 1.36 | 1.07 | 1.03 | 1.55 | 3.79 |
| 0.51 | 0.72 | 1.11 | 0.29 | 0.81 | 0.86 | 0.97 | 0.86 | 1.39 | 0.81 | 1.5 |
| 2 | 1.03 | 1.11 | 1.51 | 0.91 | 0.92 | 0.82 | 1.01 | 2.74 | 0.91 | 1.74 |
| 1.67 | 0.95 | 0.36 | 2.54 | 1.38 | 2.75 | 1.32 | 0.73 | 1.09 | 1.38 | 1.79 |
| 9 | 1.34 | 0.61 | 11 | 1.09 | 1.14 | 1.04 | 0.72 | 0.93 | 1.09 | 1.25 |
| 0.89 | 4.28 | 0.94 | 0.46 | 1.8 | 1.23 | 0.91 | 2.19 | 0.57 | 1.8 | 1.48 |
| 1.11 | 0.95 | 0.82 | 1.13 | 0.9 | 0.43 | 1 | 0.89 | 0.71 | 0.9 | 1.09 |
| 5.25 | 1.61 | 1.73 | 2.3 | 0.86 | 3.57 | 1.4 | 1.69 | 0.66 | 0.86 | 1.76 |
| 0.78 | 1.07 | 1.57 | 0.97 | 0.82 | 0.84 | 1.05 | 1.43 | 2.57 | 0.82 | 0.66 |
| 1.5 | 1.22 | 1.35 | 2.83 | 0.99 | 0.83 | 1.31 | 1.54 | 1.12 | 0.99 | 7.8 |
| 2.76 | 1.06 | 1.9 | 1.27 | 0.96 | 2.7 | 1.09 | 0.93 | 2.18 | 0.96 | 2.27 |
| 8 | 1.15 | 2.55 | 0.9 | 0.91 | 0.77 | 1.06 | 0.83 | 1.04 | 0.91 | 1.6 |
| 0.54 | 1.04 | 1.01 | 1.19 | 0.57 | 1.85 | 1.3 | 0.39 | 2.43 | 0.57 | 2.3 |
| 1.14 | 1.08 | 1.69 | 2.86 | 0.78 | 0.28 | 0.96 | 1.19 | 1.17 | 0.78 | 0.91 |
| 1.38 | 0.87 | 1.2 | 1.68 | 1.02 | 0.83 | 0.45 | 1.23 | 1.15 | 1.02 | 1.58 |
| 0.65 | 1.44 | 2.37 | 3.14 | 2.17 | 1.13 | 0.52 | 1.01 | 16.17 | 2.17 | 2.08 |
| 10 | 0.4 | 0.56 | 0.58 | 1.08 | 2.53 | 1.16 | 4.26 | 0.68 | 1.08 | 0.14 |
| 1.09 | 1.98 | 1.07 | 1.22 | 1.18 | 1.37 | 0.33 | 1.75 | 1.61 | 1.18 | 0.48 |
| 1.88 | 0.67 | 1.13 | 2.73 | 0.98 | 0.62 | 0.84 | 0.95 | 0.79 | 0.98 | 0.93 |
| 1.67 | 0.31 | 0.06 | 0.86 | 0.54 | 0.3 | 0.65 | 3.52 | 20.05 | 0.54 | 1.04 |
| 1.5 | 1.05 | 0.93 | 0.42 | 1 | 1.09 | 1.09 | 1.56 | 0.86 | 1 | 1.29 |
| 4.3 | 4.01 | 1.16 | 0.49 | 1.16 | 0.72 | 0.99 | 1.1 | 1.03 | 1.16 | 4.15 |
| 2.31 | 0.89 | 1.03 | 2.04 | 1.04 | 0.86 | 1 | 1.35 | 1.03 | 1.04 | 1.43 |
| 2.5 | 0.99 | 0.83 | 0.49 | 1.18 | 0.62 | 1.26 | 1.09 | 0.48 | 1.18 | 0.09 |
| 3.7 | 3.29 | 1.68 | 1.87 | 1.51 | 1.71 | 1.14 | 4.99 | 0.86 | 1.51 | 1 |
| 0.5 | 0.88 | 0.87 | 1.25 | 1.02 | 1.04 | 0.65 | 0.7 | 1.11 | 1.02 | 0.25 |
| 1.31 | 1.03 | 1.88 | 1.12 | 0.95 | 0.71 | 1.33 | 3.87 | 1.08 | 0.95 | 1.64 |

| | | | | | | | | | | |
|---|---|---|---|---|---|---|---|---|---|---|
| 9.25 | 0.92 | 1.18 | 0.43 | 2.12 | 3.33 | 1.06 | 0.99 | 0.23 | 2.12 | 2.42 |
| 1 | 1.03 | 1.42 | 2.5 | 0.85 | 1.92 | 1.76 | 1.03 | 1.01 | 0.85 | 2.43 |
| NA | 2.27 | 0.7 | 0.77 | 0.95 | 1.24 | 1.83 | 0.66 | 1.21 | 0.95 | 2.91 |
| 0.7 | 0.43 | 0.13 | 2.9 | 0.84 | 0.55 | 0.37 | 2.35 | 0.13 | 0.84 | 0.35 |
| 2.67 | 0.86 | 3 | 4.41 | 1.13 | 2.49 | 0.96 | 1.43 | 0.91 | 1.13 | 2.49 |
| 2.67 | 0.98 | 3.43 | 2.09 | 0.85 | 2.13 | 1.16 | 2.03 | 1.65 | 0.85 | 0.8 |
| 0.73 | 1.06 | 0.51 | 0.5 | 0.93 | 1.08 | 0.7 | 0.66 | 0.66 | 0.93 | 1.69 |
| 1.74 | 1.83 | 1.08 | 0.54 | 1.06 | 1.57 | 0.86 | 1.69 | 1 | 1.06 | 8.56 |
| 0.67 | 0.77 | 0.42 | 0.56 | 1.32 | 2.05 | 0.74 | 1.53 | 1.55 | 1.32 | 1.15 |
| 0.56 | 1.15 | 1.66 | 2.35 | 0.73 | 0.58 | 1.81 | 0.44 | 0.89 | 0.73 | 3.91 |
| 1 | 0.83 | 0.92 | 0.41 | 0.81 | 1.02 | 1.31 | 0.83 | 0.97 | 0.81 | 2.8 |
| 1 | 0.71 | 0.93 | 0.25 | 0.97 | 1.76 | 0.83 | 1.21 | 1.49 | 0.97 | 1 |
| 2 | 1.41 | 1.21 | 0.71 | 0.86 | 0.46 | 0.86 | 1.02 | 1.08 | 0.86 | 1.97 |
| 2.05 | 1.03 | 1.03 | 3.27 | 0.92 | 0.6 | 0.6 | 4.64 | 0.32 | 0.92 | 0.94 |
| 3.33 | 0.78 | 1.5 | 0.5 | 0.75 | 0.67 | 0.94 | 1.16 | 0.91 | 0.75 | 0.52 |
| 0.99 | 0.76 | 1.13 | 1.3 | 0.88 | 1.37 | 1.16 | 0.83 | 0.8 | 0.88 | 1.78 |
| 3 | 0.84 | 2.17 | 0.79 | 0.87 | 1.59 | 0.74 | 1.15 | 0.6 | 0.87 | 0.47 |
| 1.83 | 0.94 | 0.53 | 0.67 | 1.09 | 1.25 | 1.15 | 0.88 | 0.75 | 1.09 | 2.39 |
| 1.13 | 4.94 | 9.83 | 5.97 | 0.92 | 0.65 | 0.69 | 1.31 | 0.67 | 0.92 | 2.72 |
| 0.61 | 2.02 | 2.97 | 1.4 | 0.41 | 0.26 | 0.35 | 2.6 | 0.19 | 0.41 | 0.66 |
| 3.75 | 2.2 | 2.9 | 2.78 | 0.96 | 1.8 | 1.03 | 2.08 | 0.56 | 0.96 | 0.91 |
| 1.5 | 18.57 | 0.4 | 3.33 | 1.45 | 2.73 | 1.34 | 0.67 | 0.92 | 1.45 | 2.57 |
| 2.22 | 3.2 | 0.54 | 1.53 | 1.31 | 1.12 | 0.99 | 0.56 | 2.42 | 1.31 | 2.21 |
| 1.19 | 3.86 | 0.23 | 0.44 | 1.27 | 0.93 | 1.02 | 0.79 | 0.09 | 1.27 | 2.25 |
| 3.03 | 0.65 | 1.03 | 0.4 | 1.11 | 0.68 | 0.94 | 1.02 | 1.15 | 1.11 | 1.58 |
| 2.56 | 1.2 | 0.83 | 1.11 | 1.26 | 1.72 | 0.97 | 0.84 | 0.83 | 1.26 | 8.15 |
| 0.25 | 0.79 | 2.16 | 5.18 | 0.63 | 0.57 | 0.68 | 1.1 | 1.15 | 0.63 | 1.37 |
| 2 | 1.27 | 1.15 | 3.33 | 0.99 | 2.04 | 1.19 | 1.12 | 0.08 | 0.99 | 0.45 |
| 2.13 | 1.03 | 0.91 | 3.16 | 1.47 | 1.02 | 0.9 | 0.68 | 0.53 | 1.47 | 1.32 |
| 12.33 | 0.64 | 1.6 | 0.07 | 1.83 | 1.16 | 0.99 | 0.45 | 1.55 | 1.83 | 0.3 |
| 0.79 | 0.73 | 0.82 | 3.22 | 1.19 | 2.7 | 1.25 | 0.3 | 1.27 | 1.19 | 0.23 |
| 1.11 | 0.69 | 1.21 | 2.53 | 1.48 | 1.17 | 1.01 | 0.73 | 4.53 | 1.48 | 2.57 |
| 2.2 | 6.51 | 2.13 | 0.36 | 1.41 | 4.52 | 1.86 | 0.13 | 50.9 | 1.41 | 5.06 |

| | | | | | | | | | | |
|---|---|---|---|---|---|---|---|---|---|---|
| 0.5 | 1.22 | 0.75 | 2.75 | 1.16 | 1.52 | 0.9 | 1 | 1.7 | 1.16 | 1.12 |
| 0.83 | 4.36 | 1.26 | 0.31 | 1.17 | 0.69 | 0.89 | 0.89 | 0.76 | 1.17 | 2.17 |
| 0.8 | 1.48 | 0.59 | 0.8 | 0.52 | 0.37 | 1.34 | 1.49 | 9.6 | 0.52 | 4.73 |
| 4.41 | 0.66 | 0.51 | 1.74 | 0.85 | 0.62 | 0.79 | 0.95 | 0.93 | 0.85 | 0.25 |
| 1.59 | 0.37 | 1.61 | 2.15 | 0.78 | 1.02 | 1.21 | 1.23 | 3.81 | 0.78 | 1.11 |
| 2.94 | 1.06 | 0.69 | 1.49 | 1.27 | 1.03 | 0.99 | 0.95 | 1.16 | 1.27 | 1.02 |
| 2.76 | 1.3 | 0.96 | 2.12 | 1.1 | 1.15 | 0.79 | 1.03 | 1.01 | 1.1 | 0.92 |
| 1.67 | 0.89 | 1.08 | 0.58 | 1.06 | 1.02 | 0.94 | 1.03 | 2.48 | 1.06 | 0.3 |
| 0.61 | 1.16 | 1.06 | 0.52 | 1.42 | 0.86 | 0.7 | 0.91 | 0.96 | 1.42 | 1.05 |
| 0.67 | 1.88 | 0.89 | 0.79 | 1.07 | 0.83 | 0.98 | 0.61 | 1.31 | 1.07 | 1.26 |
| Inf | 0.72 | 0.34 | 1.51 | 1.04 | 0.73 | 0.77 | 1.23 | 0.61 | 1.04 | 1.42 |
| 0.9 | 0.11 | 10.97 | 1.09 | 0.44 | 0.8 | 2.35 | 2.87 | 0.63 | 0.44 | 2.47 |
| 2.04 | 0.9 | 1.2 | 2.74 | 1.46 | 1.54 | 0.87 | 1.31 | 0.58 | 1.46 | 4.37 |
| 0.49 | 0.72 | 1.67 | 3.17 | 1.14 | 0.65 | 1.07 | 2.64 | 2.33 | 1.14 | 0.36 |

| 205199_at | 218567_x_at | 212983_at | 219159_s_at | 209952_s_at | 207213_s_at | 201502_s_at | 205547_s_at | 209702_at | 202431_s_at | 201502_s_at |
|---|---|---|---|---|---|---|---|---|---|---|
| 0.85 | 0.86 | 1.29 | 2.31 | 5.19 | 2.55 | 1.09 | 0.67 | 1.07 | 1.04 | 1.09 |
| 0.8 | 1.03 | 0.97 | 3.32 | 1.3 | 1.05 | 2.23 | 1.27 | 0.72 | 0.41 | 2.23 |
| 1.18 | 1.06 | 1.51 | 0.79 | 2.65 | 0.88 | 0.67 | 1.38 | 1.08 | 1.13 | 0.67 |
| 1.25 | 0.95 | 1.07 | 2.77 | 2.27 | 1.69 | 1.11 | 3.26 | 0.85 | 1.17 | 1.11 |
| 0.72 | 0.76 | 0.88 | 1.74 | 0.94 | 0.65 | 2.56 | 0.45 | 1.02 | 1.22 | 2.56 |
| 1.96 | 0.91 | 0.83 | 0.92 | 1.97 | 1.51 | 0.98 | 1.05 | 1.31 | 735.49 | 0.98 |
| 1.64 | 0.7 | 2.06 | 0.38 | 0.38 | 2 | 1.29 | 1.24 | 1.09 | 0.85 | 1.29 |
| 0.73 | 1.07 | 1.04 | 6 | 1.37 | 0.64 | 1.6 | 0.72 | 1.11 | 1.08 | 1.6 |
| 0.8 | 1.07 | 1.1 | 2.15 | 0.75 | 1.12 | 1.32 | 0.32 | 1.1 | 0.82 | 1.32 |
| 0.86 | 0.62 | 1 | 1.03 | 1.9 | 0.55 | 2.58 | 1.56 | 0.56 | 1.95 | 2.58 |
| 0.72 | 1.01 | 0.91 | 0.71 | 0.37 | 5.5 | 1.16 | 0.65 | 0.9 | 0.89 | 1.16 |
| 1.56 | 1.19 | 1.44 | 8.33 | 0.51 | 2 | 0.58 | 0.13 | 0.95 | 0.97 | 0.58 |
| 0.91 | 1.7 | 1.18 | 0.86 | 1.09 | 1.17 | 0.85 | 1.41 | 1.04 | 1.03 | 0.85 |
| 24.85 | 0.49 | 0.74 | 16.55 | 3.31 | 39.16 | 0.43 | 2.66 | 0.09 | 0.15 | 0.43 |
| 4.31 | 1.11 | 0.96 | 1.91 | 4.07 | 1.88 | 1.02 | 0.72 | 0.97 | 1.71 | 1.02 |
| 0.57 | 0.93 | 0.99 | 2.16 | 0.44 | 0.95 | 1.15 | 2.65 | 0.94 | 0.92 | 1.15 |
| 2.5 | 1.38 | 1.13 | 2.04 | 1.08 | 1.71 | 0.7 | 0.96 | 1.07 | 0.83 | 0.7 |
| 3.24 | 1 | 1.07 | 3.57 | 4.56 | 2.64 | 2.03 | 1.29 | 0.85 | 0.74 | 2.03 |
| 2.12 | 0.91 | 1.88 | 1.4 | 0.08 | 0.5 | 1.4 | 2 | 1.36 | 0.22 | 1.4 |
| 2.95 | 0.69 | 1.24 | 1.99 | 4.46 | 21.99 | 0.54 | 1.73 | 0.83 | 0.74 | 0.54 |
| 0.29 | 0.97 | 2.52 | 0.98 | 7.94 | 1 | 1.22 | 0.69 | 0.81 | 0.49 | 1.22 |
| 10.86 | 1.08 | 0.92 | 11.74 | 2.3 | 18.81 | 3.45 | 0.45 | 0.86 | 0.88 | 3.45 |
| 1.23 | 0.97 | 0.95 | 0.57 | 1.81 | 1.5 | 1.18 | 2 | 1.04 | 1.41 | 1.18 |
| 1.41 | 1.13 | 0.71 | Inf | 2.5 | 0.89 | 0.35 | 0.31 | 0.99 | 0.75 | 0.35 |
| 1.62 | 0.61 | 0.84 | 3.4 | 2.03 | 0.74 | 2.23 | 8.04 | 0.44 | 6.79 | 2.23 |
| 0.26 | 1.31 | 0.84 | 1.92 | 4.22 | 1.88 | 0.78 | 1.13 | 0.84 | 0.76 | 0.78 |
| 1.31 | 0.78 | 0.98 | 0.74 | 0.97 | 5.5 | 0.42 | 1.97 | 1.02 | 0.76 | 0.42 |
| 2.48 | 1.66 | 0.61 | 77.45 | 2.76 | 15.09 | 4.18 | 0.29 | 1.22 | 1.03 | 4.18 |
| 2 | 0.62 | 1.34 | 1.33 | 2 | 4.07 | 0.54 | 0.46 | 1 | 3.43 | 0.54 |
| 1.52 | 0.96 | 1.28 | 4.2 | 1.45 | 1.6 | 1.47 | 1.03 | 1.2 | 3.16 | 1.47 |

| | | | | | | | | | | |
|---|---|---|---|---|---|---|---|---|---|---|
| 0.94 | 1.01 | 1.54 | 0.22 | 1.61 | 1.13 | 0.66 | 0.4 | 0.77 | 1.37 | 0.66 |
| 0.97 | 2.3 | 1.69 | 0.65 | 2.36 | 9.99 | 0.93 | 0.61 | 1.94 | 4.73 | 0.93 |
| 0.76 | 0.99 | 1.11 | 1.26 | 1.43 | 1.93 | 0.84 | 1.32 | 1.1 | 0.71 | 0.84 |
| 2.1 | 0.66 | 21.77 | 1.32 | 0.65 | 1.75 | 0.35 | 1.34 | 1.02 | 0.55 | 0.35 |
| 0.92 | 0.61 | 1.9 | 0.26 | 1.43 | 0.7 | 2.61 | 0.16 | 0.45 | 1.69 | 2.61 |
| 2.39 | 1.1 | 2.45 | 2.96 | 4.45 | 4.25 | 0.81 | 8.48 | 0.99 | 1.53 | 0.81 |
| 1.35 | 0.8 | 0.47 | 52.03 | 1.72 | 15.16 | 2.35 | 0.01 | 0.95 | 0.97 | 2.35 |
| 0.87 | 1.08 | 1.01 | 3.5 | 0.68 | 1.17 | 0.71 | 0.97 | 1.03 | 1.17 | 0.71 |
| 1.01 | 0.55 | 10.21 | 0.79 | 1.56 | 1.42 | 3.57 | 0.66 | 0.8 | 0.55 | 3.57 |
| 0.52 | 0.64 | 0.74 | 3 | 0.15 | 1.67 | 1.29 | 5.73 | 1.7 | 0.48 | 1.29 |
| 8.1 | 0.9 | 1.72 | 2.28 | 1.83 | 1.55 | 0.91 | 1.76 | 1.08 | 0.71 | 0.91 |
| 0.51 | 8.09 | 1.04 | 2.02 | 0.71 | 1.64 | 1.24 | 6.33 | 1.16 | 0.47 | 1.24 |
| 0.64 | 1.11 | 8.05 | 15.27 | 4.94 | 0.73 | 3.43 | 6.15 | 0.54 | 0.44 | 3.43 |
| 2.31 | 0.97 | 1.74 | 7.92 | 0.3 | 0.6 | 0.54 | 1.75 | 1.01 | 2.84 | 0.54 |
| 0.94 | 1.18 | 5.64 | 11.81 | 7.75 | 0.69 | 2.85 | 5.3 | 0.65 | 0.6 | 2.85 |
| 3.29 | 1.47 | 1.01 | 1.42 | 0.52 | 7.52 | 2.51 | 0.99 | 0.95 | 1.23 | 2.51 |
| 1.06 | 1.03 | 1.14 | 1.2 | 0.61 | 0.77 | 7.24 | 0.82 | 1.02 | 0.86 | 7.24 |
| 1.45 | 0.89 | 0.96 | 2.2 | 6.38 | 3.54 | 1.45 | 6.89 | 1.34 | 1.38 | 1.45 |
| 2.32 | 0.97 | 1.06 | 0.66 | 5.75 | 1.51 | 0.94 | 0.79 | 6.35 | 0.9 | 0.94 |
| 7.38 | 0.82 | 1.13 | 1.05 | 1.41 | 11.52 | 0.6 | 0.98 | 1.16 | 6.01 | 0.6 |
| 8.8 | 0.81 | 0.85 | 0.25 | 1.08 | 0.27 | 5.73 | 1.43 | 0.96 | 0.74 | 5.73 |
| 1.78 | 0.87 | 1.07 | 1.42 | 2.73 | 0.68 | 0.72 | 0.85 | 1.25 | 5.45 | 0.72 |
| 0.66 | 1.39 | 0.97 | 1.87 | 1.23 | 1.15 | 0.85 | 15.26 | 1.09 | 1.2 | 0.85 |
| 0.96 | 0.85 | 1.25 | 2.53 | 2.6 | 0.85 | 1.21 | 0.87 | 1.04 | 0.64 | 1.21 |
| 1.63 | 1.01 | 4.5 | 29.32 | 1.26 | 0.65 | 1.24 | 0.22 | 0.67 | 1.96 | 1.24 |
| 1.2 | 1.03 | 1.26 | 1.33 | 1.28 | 1.29 | 1.54 | 0.91 | 0.97 | 0.62 | 1.54 |
| 1.38 | 4.1 | 1.73 | 1.9 | 3.66 | 3.04 | 6.08 | 1.31 | 0.76 | 0.59 | 6.08 |
| 1.26 | 1.23 | 0.73 | 1.3 | 4.22 | 1.91 | 1.78 | 1.31 | 1.67 | 0.5 | 1.78 |
| 0.91 | 0.97 | 0.8 | 0.67 | 1.34 | 0.5 | 4.1 | 1.5 | 1.15 | 0.82 | 4.1 |
| 1.27 | 1.08 | 4.35 | 10.95 | 4.37 | 1 | 4.97 | 5.84 | 0.59 | 0.46 | 4.97 |
| 1.19 | 1.04 | 1.27 | 2.14 | 3.2 | 1.3 | 2.33 | 2.21 | 0.85 | 0.67 | 2.33 |
| 1.67 | 0.88 | 0.73 | 0.59 | 0.79 | 0.58 | 0.6 | 0.7 | 1.05 | 1.04 | 0.6 |
| 0.75 | 0.92 | 0.83 | 9.86 | 1.49 | 3.75 | 1.04 | 67.82 | 1.14 | 142.34 | 1.04 |

| | | | | | | | | | | |
|---|---|---|---|---|---|---|---|---|---|---|
| 4.99 | 1.09 | 1.25 | 1.4 | 0.94 | 0.83 | 0.81 | 0.93 | 1.01 | 0.91 | 0.81 |
| 0.75 | 1.4 | 1.51 | 0.42 | 1.15 | 0.79 | 1.08 | 4.44 | 0.91 | 1.85 | 1.08 |
| 3.82 | 1.04 | 0.98 | 10.12 | 0.65 | 2.25 | 1.68 | 0.71 | 1.11 | 1 | 1.68 |
| 0.97 | 1.19 | 0.99 | 1.51 | 2.49 | 1.3 | 1.41 | 1.13 | 0.91 | 1.37 | 1.41 |
| 1.09 | 0.84 | 0.89 | 5.6 | 1.07 | 1.8 | 0.94 | 0.89 | 0.88 | 1.04 | 0.94 |
| 1.04 | 0.77 | 1.38 | 0.97 | 2.38 | 0.51 | 1.07 | 1.81 | 2.02 | 1.04 | 1.07 |
| 1.67 | 0.87 | 1.06 | 2.13 | 1.17 | 0.85 | 0.34 | 2.82 | 1.49 | 1.83 | 0.34 |
| 0.91 | 1.24 | 1.63 | 0.79 | 0.5 | 0.5 | 1.55 | 2.32 | 1.11 | 1.1 | 1.55 |
| 0.76 | 1.42 | 1.36 | 1.15 | 4.29 | 4.38 | 1.33 | 0.67 | 0.73 | 1.08 | 1.33 |
| 0.69 | 1.43 | 0.78 | 2.21 | 1.62 | 1.25 | 1.05 | 0.27 | 0.71 | 0.8 | 1.05 |
| 1.9 | 0.33 | 0.37 | 0.59 | 4.07 | 2.32 | 0.71 | 1.15 | 1.42 | 2.8 | 0.71 |
| 2.44 | 1.15 | 1.03 | 2.67 | 4 | 0.12 | 1.14 | 0.81 | 0.88 | 0.74 | 1.14 |
| 1.05 | 1.25 | 0.98 | 4.42 | 1.56 | 1.5 | 1.14 | 2.24 | 1.12 | 1.33 | 1.14 |
| 1.72 | 0.77 | 1.11 | 57.97 | 1.52 | 0.25 | 2.69 | 0.18 | 0.85 | 2 | 2.69 |
| 0.29 | 1.18 | 1.88 | 4.4 | 2.4 | 0.5 | 0.93 | 0.95 | 1.13 | 1 | 0.93 |
| 0.04 | 6.15 | 2.17 | 0.01 | 0.39 | 0.01 | 2.52 | 0.11 | 30.68 | 52.03 | 2.52 |
| 2.85 | 0.86 | 0.6 | 0.82 | 1.44 | 2.28 | 2.22 | 1.51 | 0.81 | 0.53 | 2.22 |
| 0.62 | 1.18 | 0.84 | 0.17 | 0.5 | 0.71 | 0.98 | 1.14 | 0.53 | 0.13 | 0.98 |
| 0.86 | 1.03 | 0.85 | 1.42 | 1.7 | 1.69 | 0.74 | 0.76 | 1.14 | 1.22 | 0.74 |
| 4.9 | 1.14 | 1.88 | 1.94 | 2.78 | 2.19 | 1.34 | 1.75 | 1.24 | 1.02 | 1.34 |
| 1.3 | 1.55 | 1.08 | 2.32 | 1.45 | 2 | 1.62 | 5.22 | 0.96 | 0.33 | 1.62 |
| 2.31 | 1.2 | 1.24 | 2.21 | 1.42 | 0.7 | 1.35 | 2.87 | 0.99 | 1.17 | 1.35 |
| 0.99 | 0.77 | 1.6 | 0.38 | 0.38 | 2 | 1.12 | 0.93 | 0.95 | 0.87 | 1.12 |
| 2.4 | 0.89 | 1.52 | 0.9 | 0.9 | 2.64 | 0.94 | 0.95 | 1.36 | 0.51 | 0.94 |
| 0.35 | 1.17 | 1.14 | 0.55 | 1.44 | 1.07 | 0.79 | 0.88 | 1.02 | 1.04 | 0.79 |
| 1.4 | 1.04 | 0.9 | 1 | 0.39 | 0.71 | 1.55 | 1.06 | 1.09 | 1.07 | 1.55 |
| 2.86 | 1.17 | 1.17 | 0.86 | 2.05 | 1.38 | 1.22 | 1.83 | 1.13 | 1.28 | 1.22 |
| 0.75 | 1.16 | 1.34 | 0.72 | 1.35 | 1.32 | 0.93 | 0.89 | 1.03 | 1.31 | 0.93 |
| 1.9 | 0.78 | 0.89 | 1.66 | 1.75 | 1.68 | 0.99 | 2.32 | 1.05 | 1.07 | 0.99 |
| 0.83 | 0.98 | 1.07 | 0.81 | 1.47 | 1.75 | 1.32 | 1.11 | 1.05 | 1.03 | 1.32 |
| 9.75 | 1.27 | 1.25 | 5.03 | 1.5 | 1.38 | 1.48 | 2.66 | 0.9 | 0.83 | 1.48 |
| 1.26 | 1.97 | 1.85 | 0.55 | 1.05 | 9.82 | 0.41 | 3 | 0.99 | 0.62 | 0.41 |
| 0.56 | 1.13 | 0.91 | 11.43 | 0.43 | 0.75 | 2.25 | 1.67 | 1.11 | 0.77 | 2.25 |

| | | | | | | | | | | |
|---|---|---|---|---|---|---|---|---|---|---|
| 1.04 | 0.93 | 0.69 | 1.57 | 2.08 | 1.64 | 0.75 | 1 | 1.02 | 2.26 | 0.75 |
| 0.99 | 1.13 | 1.23 | 1.82 | 1.33 | 2.07 | 1 | 0.63 | 0.93 | 0.92 | 1 |
| 1.38 | 0.99 | 1.26 | 9.25 | 1.51 | 0.33 | 1.15 | 1.05 | 0.96 | 1.59 | 1.15 |
| 0.77 | 1.21 | 1.5 | 1.81 | 2.86 | 3.73 | 1.18 | 0.7 | 1.72 | 0.6 | 1.18 |
| 1.07 | 0.63 | 0.9 | 1.68 | 0.43 | 1.07 | 0.85 | 0.95 | 1.92 | 1.17 | 0.85 |
| 0.49 | 3.14 | 1.74 | 0.58 | 3.88 | 9.41 | 3.58 | 0.78 | 0.38 | 0.62 | 3.58 |
| 0.87 | 0.93 | 1.2 | 1.47 | 1.07 | 13.5 | 1.89 | 1.7 | 0.92 | 1.52 | 1.89 |
| 1.29 | 1.1 | 1.04 | 0.52 | 1.75 | 0.58 | 1.02 | 1.27 | 1.19 | 0.71 | 1.02 |
| 2.34 | 0.54 | 0.73 | 3.06 | 1.13 | 1.28 | 0.64 | 2.8 | 1.05 | 1.65 | 0.64 |
| 2.42 | 0.74 | 0.79 | 1.08 | 3.44 | 16.07 | 1.71 | 1.19 | 0.95 | 1.39 | 1.71 |
| 1.35 | 1.14 | 1.17 | 3.56 | 1.62 | 3.41 | 0.88 | 0.9 | 1.17 | 0.65 | 0.88 |
| 1.42 | 1.16 | 1.04 | 0.8 | 0.79 | 0.9 | 0.89 | 1.19 | 1.26 | 1.26 | 0.89 |
| 2.89 | 0.89 | 1.18 | 1.81 | 0.83 | 2.52 | 1.41 | 0.43 | 0.97 | 1.03 | 1.41 |
| 1.44 | 0.91 | 14.7 | 0.78 | 0.49 | 0.81 | 0.68 | 1.3 | 1.03 | 0.84 | 0.68 |
| 0.93 | 0.85 | 0.9 | 1.26 | 0.88 | 1.26 | 1.04 | 1.95 | 0.93 | 2.56 | 1.04 |
| 1.02 | 0.93 | 1.06 | 0.7 | 1.87 | 2.38 | 1.31 | 2.63 | 1.01 | 1.01 | 1.31 |
| 1.3 | 1.05 | 0.94 | 2.07 | 2.71 | 0.53 | 0.83 | 1.98 | 0.97 | 1.32 | 0.83 |
| 0.76 | 1.28 | 1.12 | 1.11 | 1.66 | 1.13 | 2.39 | 0.96 | 0.95 | 1.75 | 2.39 |
| 1.81 | 1.19 | 0.85 | 0.62 | 1.72 | 0.87 | 4.17 | 1.28 | 1.01 | 0.97 | 4.17 |
| 0.95 | 0.82 | 1.4 | 2.5 | 1.42 | 0.8 | 0.66 | 1.2 | 0.8 | 0.81 | 0.66 |
| 0.5 | 1.15 | 0.9 | 0.83 | 1.7 | 1 | 0.73 | 0.77 | 0.81 | 0.85 | 0.73 |
| 1.27 | 0.94 | 0.96 | 0.7 | 3.83 | 0.69 | 1 | 0.97 | 0.99 | 1.09 | 1 |
| 0.87 | 0.96 | 0.95 | 1.18 | 1.5 | 0.71 | 1.05 | 0.48 | 1 | 1.21 | 1.05 |
| 1.09 | 1.5 | 0.82 | 1.36 | 0.77 | 1.25 | 0.52 | 0.27 | 1.43 | 1.73 | 0.52 |
| 1.97 | 1.02 | 0.99 | 0.7 | 4 | 0.33 | 0.48 | 0.62 | 1.13 | 0.87 | 0.48 |
| 1.78 | 1.2 | 0.8 | 0.57 | 0.62 | 0.37 | 0.88 | 0.59 | 0.85 | 1.76 | 0.88 |
| 0.69 | 1.14 | 1.13 | 0.5 | 4.22 | 2.5 | 1.14 | 1.33 | 0.92 | 1.23 | 1.14 |
| 1.17 | 0.89 | 0.57 | 0.94 | 1.29 | 0.75 | 1.19 | 0.2 | 1.34 | 2.06 | 1.19 |
| 0.4 | 0.9 | 0.93 | 0.37 | 0.32 | 0.8 | 0.58 | 4.58 | 1.32 | 1.32 | 0.58 |
| 1.38 | 1.02 | 0.94 | 0.85 | 2.22 | 1.04 | 0.98 | 1.36 | 0.94 | 0.9 | 0.98 |
| 0.62 | 1.02 | 1.11 | 0.67 | 0.84 | 0.85 | 1.24 | 1.3 | 0.95 | 1.04 | 1.24 |
| 0.75 | 1.4 | 1.51 | 0.42 | 1.15 | 0.79 | 1.08 | 4.44 | 0.91 | 1.85 | 1.08 |
| 1.08 | 1.11 | 0.9 | 0.88 | 0.82 | 0.54 | 3.55 | 0.65 | 0.97 | 1.59 | 3.55 |

| | | | | | | | | | | |
|---|---|---|---|---|---|---|---|---|---|---|
| 0.74 | 1.08 | 1 | 1.18 | 0.59 | 0.66 | 0.72 | 0.91 | 1.29 | 1.3 | 0.72 |
| 2.19 | 0.99 | 0.92 | 2.65 | 0.97 | 0.91 | 1.75 | 0.86 | 0.65 | 1.04 | 1.75 |
| 0.91 | 1.07 | 1.13 | 1.11 | 4.29 | 0.92 | 0.78 | 2.26 | 1.57 | 1.12 | 0.78 |
| 2.04 | 1.17 | 1.01 | 1.07 | 0.57 | 1.42 | 3 | 1 | 1.26 | 1.08 | 3 |
| 6.42 | 0.87 | 1.04 | 5.83 | 1.69 | 4.21 | 0.53 | 1.38 | 0.89 | 0.97 | 0.53 |
| 1 | 1.11 | 0.92 | 7.67 | 5.33 | 0.33 | 0.93 | 0.5 | 0.99 | 1.1 | 0.93 |
| 1.12 | 1.03 | 0.9 | 1.07 | 1.69 | 0.88 | 0.96 | 1.03 | 1 | 1.19 | 0.96 |
| 2.68 | 0.87 | 0.75 | 1.16 | 3 | 1.17 | 0.67 | 1.15 | 1.11 | 0.84 | 0.67 |
| 5.1 | 0.77 | 1.01 | 10.41 | 1.77 | 0.86 | 1.02 | 3.02 | 0.95 | 0.65 | 1.02 |
| 2.27 | 1.2 | 1.28 | 1.42 | 0.39 | 1.75 | 1.89 | 1.13 | 1.1 | 0.84 | 1.89 |
| 1.09 | 0.84 | 1.1 | 2.51 | 1.31 | 0.59 | 1.21 | 0.2 | 0.94 | 1.09 | 1.21 |
| 0.87 | 0.88 | 1.26 | 0.37 | 1.74 | 0.73 | 1.52 | 0.81 | 0.95 | 1.15 | 1.52 |
| 0.83 | 1.07 | 1.05 | 0.85 | 1.8 | 1.17 | 1.46 | 0.92 | 1 | 1.6 | 1.46 |
| 1.04 | 1.15 | 0.89 | 4.29 | 1.53 | 0.86 | 1.73 | 0.23 | 1.09 | 0.93 | 1.73 |
| 0.31 | 0.58 | 0.84 | 0.87 | 1.69 | 1.21 | 1.3 | 0.8 | 0.78 | 0.41 | 1.3 |
| 1.02 | 1.17 | 1.05 | 1.16 | 1.17 | 1.1 | 0.68 | 1.52 | 1.08 | 0.87 | 0.68 |
| 0.75 | 0.87 | 1.1 | 1.1 | 2.27 | 2.88 | 0.87 | 1.66 | 1.01 | 0.92 | 0.87 |
| 0.84 | 1.34 | 1.19 | 0.86 | 1.72 | 0.87 | 1.29 | 0.9 | 1.34 | 2.9 | 1.29 |
| 0.96 | 0.96 | 0.83 | 0.89 | 1.36 | 1.19 | 1.01 | 1.28 | 1.03 | 1.03 | 1.01 |
| 1.07 | 1.18 | 1.07 | 1.71 | 0.96 | 1.2 | 0.57 | 1.46 | 1.42 | 0.77 | 0.57 |
| 2.06 | 0.71 | 1.12 | 1.67 | 0.91 | 1.9 | 1.31 | 1.52 | 0.89 | 0.49 | 1.31 |
| 1.69 | 0.83 | 0.66 | 5.5 | 0.82 | 3.86 | 1.19 | 0.39 | 1.18 | 0.46 | 1.19 |
| 1.53 | 1.12 | 1.35 | 1.47 | 2.89 | 0.87 | 0.86 | 2.04 | 0.99 | 0.85 | 0.86 |
| 1.17 | 0.78 | 0.92 | 3.96 | 1.21 | 0.94 | 0.69 | 1.08 | 0.88 | 0.68 | 0.69 |
| 0.94 | 1.08 | 2.54 | 0.44 | 1.54 | 1.33 | 1.04 | 1.16 | 0.95 | 0.94 | 1.04 |
| 1.1 | 1.14 | 1.42 | 1.17 | 1.82 | 1.18 | 1.19 | 0.43 | 0.82 | 0.89 | 1.19 |
| 0.74 | 0.79 | 1.28 | 1.18 | 1.45 | 0.65 | 3.06 | 1.43 | 1 | 0.88 | 3.06 |
| 1.99 | 0.74 | 1.06 | 1.49 | 1.77 | 2.25 | 0.94 | 1.09 | 0.83 | 1.4 | 0.94 |
| 0.84 | 1.19 | 1.03 | 2.57 | 1.46 | 1.81 | 0.81 | 1.33 | 1.01 | 0.67 | 0.81 |
| 1.19 | 0.84 | 0.98 | 0.83 | 0.17 | 0.31 | 2 | 1.15 | 1.05 | 0.93 | 2 |
| 0.95 | 1.18 | 1.14 | 1.19 | 1.66 | 2.61 | 0.78 | 2.25 | 1.12 | 0.72 | 0.78 |
| 0.92 | 1.09 | 1.01 | 0.83 | 1.09 | 0.93 | 1.66 | 1.05 | 0.9 | 1.01 | 1.66 |
| 2.21 | 1.08 | 1.12 | 1.97 | 1.33 | 2.83 | 1.06 | 0.83 | 0.94 | 0.94 | 1.06 |

| | | | | | | | | | | |
|---|---|---|---|---|---|---|---|---|---|---|
| 0.56 | 1.39 | 1.86 | 1.12 | 0.47 | 1.01 | 1.15 | 0.38 | 0.74 | 0.81 | 1.15 |
| 1.28 | 1.18 | 1.31 | 1.33 | 1.48 | 0.95 | 0.74 | 1.78 | 1.11 | 0.89 | 0.74 |
| 1.18 | 1.06 | 1 | 0.89 | 1.91 | 1.33 | 1.04 | 1.11 | 0.97 | 0.96 | 1.04 |
| 1.93 | 0.49 | 0.53 | 0.9 | 2.18 | 0.74 | 1.07 | 1.19 | 0.68 | 0.77 | 1.07 |
| 0.61 | 0.71 | 1.13 | 0.69 | 0.35 | 13.33 | 1.3 | 1.62 | 0.57 | 0.68 | 1.3 |
| 0.99 | 1.01 | 1.08 | 1.24 | 0.93 | 1.13 | 1.19 | 1.16 | 0.95 | 0.92 | 1.19 |
| 1.12 | 1.03 | 1.55 | 0.22 | 2.59 | 0.97 | 0.66 | 0.48 | 0.79 | 1.4 | 0.66 |
| 0.69 | 1.05 | 0.81 | 1.14 | 0.38 | 0.17 | 0.87 | 0.67 | 1.11 | 2.13 | 0.87 |
| 1.84 | 1 | 0.91 | 0.66 | 1.25 | 2.28 | 1.45 | 1.18 | 1.12 | 0.71 | 1.45 |
| 0.47 | 0.91 | 1.38 | 0.34 | 2.33 | 2.26 | 0.62 | 0.7 | 0.77 | 1.63 | 0.62 |
| 0.59 | 0.92 | 1.09 | 0.8 | 0.68 | 1 | 1.18 | 1.14 | 0.97 | 0.9 | 1.18 |
| 1.14 | 1.11 | 1.8 | 0.98 | 2.78 | 2.26 | 0.9 | 1.44 | 0.98 | 0.93 | 0.9 |
| 0.76 | 0.68 | 0.9 | Inf | 0.77 | 0.59 | 0.87 | 0.7 | 0.83 | 0.98 | 0.87 |
| 0.93 | 0.8 | 0.86 | 2.92 | 5.09 | 1 | 0.92 | 1.51 | 1.06 | 0.92 | 0.92 |
| 1.42 | 1.15 | 0.82 | 1.13 | 0.6 | 1.25 | 3.13 | 0.87 | 1.23 | 0.97 | 3.13 |
| 1.54 | 0.99 | 0.99 | 1.5 | 1.79 | 0.71 | 1.35 | 1.38 | 1.01 | 1.52 | 1.35 |
| 1.27 | 1.17 | 0.96 | 1.23 | 0.65 | 2.59 | 0.96 | 1.13 | 1.11 | 0.86 | 0.96 |
| 1.07 | 0.91 | 0.91 | 0.69 | 0.76 | 1.37 | 1.89 | 0.56 | 0.96 | 0.97 | 1.89 |
| 0.91 | 1.16 | 0.57 | 1.1 | 0.47 | 0.07 | 0.83 | 1.29 | 1.08 | 1.24 | 0.83 |
| 1.62 | 0.96 | 0.78 | 2 | 1.88 | 0.77 | 0.77 | 1.07 | 0.78 | 0.99 | 0.77 |
| 0.91 | 0.89 | 1.02 | 0.78 | 1.08 | 1.33 | 1.13 | 0.85 | 1.03 | 1.01 | 1.13 |
| 1.07 | 1.17 | 2.17 | 1.83 | 2.33 | 0.84 | 3 | 0.57 | 0.61 | 1.33 | 3 |
| 1.13 | 0.98 | 1.08 | 2.52 | 2.27 | 1.25 | 0.91 | 1.04 | 0.95 | 1.34 | 0.91 |
| 0.92 | 1.07 | 1.18 | 0.94 | 2.31 | 1.33 | 1 | 1.14 | 1.1 | 0.89 | 1 |
| 0.83 | 0.97 | 0.98 | 0.71 | 0.81 | 2.02 | 0.98 | 1.24 | 0.99 | 1.27 | 0.98 |
| 1.31 | 0.8 | 0.54 | 52.71 | 0.69 | 0.46 | 0.21 | 1.38 | 0.97 | 0.27 | 0.21 |
| 2.3 | 1.03 | 1 | 2.8 | 1.5 | 0.73 | 1.11 | 1.93 | 0.95 | 0.84 | 1.11 |
| 1.18 | 1.11 | 1.16 | 1.26 | 1.21 | 1.2 | 1.06 | 0.9 | 0.94 | 0.93 | 1.06 |
| 4.55 | 0.99 | 1.04 | 1.46 | 1.23 | 1.19 | 0.97 | 1.08 | 0.91 | 0.93 | 0.97 |
| 0.89 | 1.15 | 1.18 | 1.62 | 0.53 | 0.42 | 0.92 | 1.82 | 1.03 | 0.86 | 0.92 |
| 0.43 | 0.95 | 1.51 | 1.26 | 0.47 | 0.18 | 0.86 | 1.45 | 1.44 | 1.49 | 0.86 |
| 6.6 | 0.9 | 1.02 | 0.25 | 1.12 | 1.06 | 1.07 | 1.02 | 0.91 | 0.88 | 1.07 |
| 0.74 | 1.21 | 0.95 | 4.76 | 0.17 | 1.3 | 1.1 | 1.29 | 0.78 | 0.75 | 1.1 |

| | | | | | | | | | | |
|---|---|---|---|---|---|---|---|---|---|---|
| 0.81 | 1.19 | 2.12 | 1.44 | 4.38 | 10.38 | 1.53 | 0.95 | 1.13 | 0.74 | 1.53 |
| 0.67 | 1.07 | 0.85 | 2.25 | 0.54 | 0.5 | 0.52 | 0.54 | 1.3 | 1.34 | 0.52 |
| 2.28 | 0.55 | 0.95 | 2.76 | 0.71 | 1.94 | 0.98 | 0.19 | 1.08 | 0.78 | 0.98 |
| 0.29 | 0.84 | 0.84 | 19.92 | 0.61 | 0.36 | 0.54 | 2.44 | 1.25 | 0.87 | 0.54 |
| 0.98 | 1.24 | 1.13 | 1.02 | 0.91 | 1.44 | 0.73 | 1.37 | 1.24 | 0.93 | 0.73 |
| 0.56 | 0.91 | 0.85 | 3.61 | 0.58 | 0.46 | 1.08 | 0.67 | 0.93 | 0.95 | 1.08 |
| 0.97 | 0.84 | 0.93 | 0.78 | 3.46 | 1.33 | 0.48 | 4.74 | 1 | 0.8 | 0.48 |
| 1.14 | 0.92 | 1.06 | 0.73 | 4 | 0.35 | 0.85 | 2.02 | 1.03 | 0.78 | 0.85 |
| 0.72 | 1.38 | 1.32 | 0.41 | 2.92 | 4.76 | 1.46 | 0.58 | 0.75 | 0.83 | 1.46 |
| 3.9 | 1.04 | 0.73 | 1.65 | 0.81 | 1.67 | 1.23 | 7.08 | 1.24 | 0.41 | 1.23 |
| 0.71 | 1.02 | 0.81 | 0.2 | 1 | 0.57 | 1.13 | 0.8 | 0.96 | 1.1 | 1.13 |
| 1.95 | 0.85 | 0.97 | 0.4 | 1.28 | 0.67 | 0.77 | 0.66 | 1.01 | 0.89 | 0.77 |
| 0.73 | 0.95 | 0.86 | 1.22 | 0.53 | 2.5 | 3.55 | 1.38 | 0.66 | 1.17 | 3.55 |
| 1.97 | 1.04 | 0.92 | 0.77 | 3.21 | 1.15 | 1.11 | 0.72 | 0.99 | 0.75 | 1.11 |
| 1.68 | 0.98 | 0.75 | 1.38 | 2.64 | 1.28 | 1.2 | 1.35 | 0.91 | 0.81 | 1.2 |
| 1.86 | 1.03 | 0.88 | 1.88 | 0.69 | 0.69 | 0.56 | 0.75 | 1.13 | 0.44 | 0.56 |
| 0.35 | 1.15 | 0.87 | 0.5 | 3.4 | 2.33 | 0.82 | 0.79 | 1.02 | 0.98 | 0.82 |
| 0.97 | 0.96 | 1.09 | 1.74 | 0.66 | 1.1 | 0.78 | 0.71 | 0.97 | 0.68 | 0.78 |
| 1.19 | 1.29 | 0.92 | 5.33 | 5.04 | 0.83 | 1.21 | 14.16 | 1.18 | 42.28 | 1.21 |
| 0.76 | 0.88 | 0.41 | 0.28 | 0.53 | 0.2 | 1.07 | 18.8 | 2.35 | 0.35 | 1.07 |
| 0.73 | 1.11 | 0.96 | 4.47 | 1.33 | 1 | 0.83 | 4.75 | 1.09 | 1.71 | 0.83 |
| 0.62 | 0.95 | 1.45 | 0.28 | 1.48 | 1.77 | 0.64 | 0.52 | 0.76 | 1.49 | 0.64 |
| 1.18 | 1.09 | 1.31 | 1.9 | 2.4 | 0.07 | 0.37 | 2.76 | 0.79 | 1.32 | 0.37 |
| 8.14 | 0.83 | 1.27 | 0.56 | 0.97 | 1.82 | 0.35 | 30.45 | 1.15 | 1.93 | 0.35 |
| 0.68 | 1.22 | 1.11 | 0.87 | 1.14 | 2.79 | 1.23 | 0.8 | 1.1 | 0.8 | 1.23 |
| 0.86 | 1.08 | 1.26 | 0.79 | 0.5 | 6.93 | 1.15 | 0.89 | 0.98 | 0.72 | 1.15 |
| 0.94 | 1.22 | 0.63 | 0.55 | 1.14 | 0.9 | 0.36 | 0.93 | 0.92 | 1.1 | 0.36 |
| 1.04 | 0.89 | 0.99 | 0.36 | 0.46 | 1.33 | 1.34 | 1.4 | 1.64 | 0.68 | 1.34 |
| 0.96 | 1.58 | 1.47 | 3.62 | 1.36 | 0.75 | 2.01 | 12.86 | 0.82 | 0.72 | 2.01 |
| 1.19 | 0.72 | 1.83 | 0.81 | 1.62 | 1.4 | 0.26 | 2.02 | 0.61 | 0.27 | 0.26 |
| 1.09 | 1.12 | 1.19 | 4 | 0.15 | 0.68 | 0.87 | 6.17 | 1.01 | 0.64 | 0.87 |
| 0.83 | 0.94 | 1.48 | 1.46 | 1.67 | 0.58 | 1.68 | 1.01 | 0.97 | 0.11 | 1.68 |
| 0.56 | 1.25 | 1.41 | 1.81 | 2.49 | 1.33 | 1.19 | 0.58 | 0.89 | 1.44 | 1.19 |

| | | | | | | | | | | |
|---|---|---|---|---|---|---|---|---|---|---|
| 0.68 | 1.19 | 1.16 | 1.57 | 3 | 0.89 | 0.88 | 1.64 | 1.01 | 0.86 | 0.88 |
| 0.82 | 0.8 | 1.17 | 0.58 | 0.77 | 0.73 | 1.21 | 3.17 | 0.98 | 1.17 | 1.21 |
| 1.03 | 0.56 | 0.52 | 1.52 | 16.38 | 0.95 | 0.73 | 0.78 | 2.32 | 1.02 | 0.73 |
| 0.46 | 0.86 | 0.85 | 0.95 | 0.88 | 1.78 | 0.92 | 0.28 | 0.93 | 0.45 | 0.92 |
| 0.75 | 1.29 | 0.78 | 0.25 | 0.71 | 1.91 | 0.84 | 2.06 | 1.02 | 0.82 | 0.84 |
| 0.9 | 0.83 | 1.27 | 1.07 | 1.85 | 1.11 | 1.05 | 1.01 | 0.95 | 1.32 | 1.05 |
| 1.33 | 0.95 | 1.1 | 2.38 | 1.48 | 2.97 | 1.14 | 1.16 | 0.91 | 0.89 | 1.14 |
| 1.24 | 0.99 | 1.06 | 0.8 | 1.34 | 3.47 | 0.8 | 0.3 | 0.87 | 1.09 | 0.8 |
| 1.65 | 1.24 | 1.42 | 0.68 | 1.31 | 1.12 | 1.33 | 1.39 | 1.1 | 0.78 | 1.33 |
| 2.84 | 0.81 | 1.07 | 0.4 | 0.61 | 0.97 | 1.19 | 0.56 | 1.16 | 0.57 | 1.19 |
| 0.68 | 1.01 | 1.04 | 0.14 | 1.14 | 0.96 | 1.14 | 3.91 | 1.15 | 0.87 | 1.14 |
| 6.25 | 0.26 | 0.44 | 0.15 | 0.28 | 0.63 | 2.33 | 2.53 | 7.17 | 14.57 | 2.33 |
| 0.81 | 0.74 | 1.46 | 2 | 0.53 | 0.55 | 0.29 | 2.31 | 0.95 | 0.89 | 0.29 |
| 0.55 | 1.1 | 1.14 | 0.5 | 1.67 | 8.67 | 0.76 | 0.44 | 1.21 | 0.76 | 0.76 |

| 202431_s_at | 217028_at | 217192_s_at | 212983_at | 208606_s_at | 214639_s_at | 201531_at | 201502_s_at | 201466_s_at | 219993_at | 207826_s_at |
|---|---|---|---|---|---|---|---|---|---|---|
| 1.04 | 1.24 | 2.91 | 1.29 | 0.51 | 0.98 | 1.23 | 1.09 | 1.15 | 2.06 | 1.34 |
| 0.41 | 1.78 | 1.38 | 0.97 | 1.21 | 1.56 | 3.36 | 2.23 | 0.66 | 0.91 | 1.81 |
| 1.13 | 0.83 | 1.15 | 1.51 | 1.4 | 1.18 | 0.93 | 0.67 | 7.25 | 2.21 | 1.14 |
| 1.17 | 3.57 | 1.47 | 1.07 | 6.01 | 2.35 | 1.27 | 1.11 | 1.42 | 2.61 | 1.04 |
| 1.22 | 1.07 | 1.22 | 0.88 | 1.94 | 0.77 | 0.65 | 2.56 | 0.88 | 0.96 | 1.19 |
| 735.49 | 1.12 | 1.18 | 0.83 | 3.69 | 117.83 | 0.99 | 0.98 | 3.02 | 1.9 | 0.81 |
| 0.85 | 0.17 | 0.51 | 2.06 | 0.02 | 0.48 | 0.58 | 1.29 | 1.1 | 3.3 | 0.63 |
| 1.08 | 0.9 | 1.39 | 1.04 | 0.95 | 1.21 | 1.19 | 1.6 | 1.89 | 2.55 | 2.75 |
| 0.82 | 2.41 | 0.84 | 1.1 | 7.47 | 1.83 | 1.11 | 1.32 | 0.49 | 0.84 | 1.02 |
| 1.95 | 4.84 | 4.42 | 1 | 3.75 | 0.72 | 6.21 | 2.58 | 4.29 | 2.81 | 12.93 |
| 0.89 | 2.6 | 4.35 | 0.91 | 0.83 | 0.4 | 1.56 | 1.16 | 0.92 | 0.35 | 1.15 |
| 0.97 | 2.78 | 0.6 | 1.44 | 2.5 | 1.27 | 1.66 | 0.58 | 1.22 | 2.41 | 1.55 |
| 1.03 | 1.56 | 1.05 | 1.18 | 1.49 | 1.47 | 1.43 | 0.85 | 0.95 | 0.87 | 1.01 |
| 0.15 | 0.45 | 8.85 | 0.74 | 13.08 | 3.63 | 0.84 | 0.43 | 0.39 | 8.31 | 0.03 |
| 1.71 | 0.7 | 2.23 | 0.96 | 3.5 | 1.56 | 1.55 | 1.02 | 0.87 | 1.33 | 0.88 |
| 0.92 | 7.08 | 2.81 | 0.99 | 1.8 | 1.26 | 0.59 | 1.15 | 1.06 | 5.07 | 2.69 |
| 0.83 | 2.64 | 2.44 | 1.13 | 0.94 | 0.69 | 0.8 | 0.7 | 0.88 | 3.07 | 0.58 |
| 0.74 | Inf | 7.84 | 1.07 | 2.85 | 1.07 | 1.27 | 2.03 | 0.97 | 0.6 | 2.48 |
| 0.22 | 0.4 | 0.99 | 1.88 | 30.5 | 1.24 | 0.94 | 1.4 | 0.83 | 2.06 | 1.02 |
| 0.74 | 1.96 | 2.67 | 1.24 | 3.33 | 5.27 | 1.17 | 0.54 | 0.91 | 0.86 | 0.84 |
| 0.49 | 1.21 | 94.18 | 2.52 | 0.14 | 0.51 | 0.91 | 1.22 | 1.74 | 0.58 | 1.73 |
| 0.88 | 0.97 | 3.12 | 0.92 | 10 | 0.99 | 2.24 | 3.45 | 0.97 | 0.15 | 0.25 |
| 1.41 | 4.86 | 0.96 | 0.95 | 6.52 | 0.36 | 1.01 | 1.18 | 1.08 | 3.28 | 0.77 |
| 0.75 | 4.44 | 0.75 | 0.71 | 1.94 | 0.45 | 0.88 | 0.35 | 1.95 | 6.65 | 0.11 |
| 6.79 | 0.15 | 0.89 | 0.84 | 1.45 | 0.01 | 2.39 | 2.23 | 0.95 | 0.82 | 0.14 |
| 0.76 | 0.19 | 1.02 | 0.84 | 1.62 | 2.42 | 1.19 | 0.78 | 3.33 | 0.84 | 1.79 |
| 0.76 | 0.74 | 4.73 | 0.98 | 1.12 | 1.53 | 0.97 | 0.42 | 0.53 | 1.38 | 0.26 |
| 1.03 | 3.78 | 1.16 | 0.61 | 2.68 | 2.79 | 7.44 | 4.18 | 0.78 | 2.24 | 0.52 |
| 3.43 | 0.21 | 1.56 | 1.34 | 1.33 | 0.11 | 1.29 | 0.54 | 0.31 | 7.27 | 2.78 |
| 3.16 | 2.13 | 0.72 | 1.28 | 1.76 | 1.3 | 1.82 | 1.47 | 1.02 | 5.28 | 1.72 |

| | | | | | | | | | | |
|---|---|---|---|---|---|---|---|---|---|---|
| 1.37 | 0.43 | 0.89 | 1.54 | 4.11 | 1.09 | 0.58 | 0.66 | 0.3 | 6.09 | 0.81 |
| 4.73 | 366.66 | 1.05 | 1.69 | 0.32 | 6.39 | 0.38 | 0.93 | 0.07 | 0.15 | 8.38 |
| 0.71 | 0.89 | 2.16 | 1.11 | 1.22 | 0.96 | 0.76 | 0.84 | 1.08 | 1.07 | 1.22 |
| 0.55 | 0.8 | 5.91 | 21.77 | 0.11 | 0.4 | 0.43 | 0.35 | 0.28 | 2.05 | 0.17 |
| 1.69 | 0.67 | 0.22 | 1.9 | 0.56 | 0.3 | 1.26 | 2.61 | 0.68 | 0.4 | 0.64 |
| 1.53 | 6.82 | 4.08 | 2.45 | 16.1 | 1.16 | 0.81 | 0.81 | 0.72 | 1.36 | 14.95 |
| 0.97 | 12.34 | 2.43 | 0.47 | 1.01 | 2.6 | 1.52 | 2.35 | 0.55 | 6.18 | 1.16 |
| 1.17 | 0.35 | 1.79 | 1.01 | 2 | 1.05 | 0.73 | 0.71 | 0.91 | 0.36 | 0.92 |
| 0.55 | 27.81 | 1.66 | 10.21 | 0.77 | 0.54 | 1.33 | 3.57 | 1.96 | 0.74 | 0.47 |
| 0.48 | 2.67 | 0.52 | 0.74 | 8.5 | 3.67 | 0.64 | 1.29 | 0.77 | 1.68 | 1.91 |
| 0.71 | 1.29 | 1.07 | 1.72 | 17.4 | 0.85 | 5.25 | 0.91 | 0.75 | 0.3 | 0.89 |
| 0.47 | 1.29 | 0.42 | 1.04 | 0.56 | 8.96 | 0.86 | 1.24 | 1.05 | 3.4 | 1.25 |
| 0.44 | 7.25 | 2.92 | 8.05 | 0.5 | 1.23 | 2.37 | 3.43 | 7.15 | 1.26 | 0.89 |
| 2.84 | 0.12 | 1.59 | 1.74 | 1.21 | 1.39 | 1.23 | 0.54 | 7.37 | 1.8 | 0.07 |
| 0.6 | 2.67 | 1.95 | 5.64 | 0.44 | 1.35 | 1.77 | 2.85 | 4.89 | 0.85 | 0.95 |
| 1.23 | 2.99 | 1.29 | 1.01 | 1.18 | 2.82 | 1.14 | 2.51 | 1.77 | 3.18 | 6.83 |
| 0.86 | 1.04 | 1.46 | 1.14 | 0.67 | 0.85 | 0.81 | 7.24 | 1.23 | 1 | 1.27 |
| 1.38 | 1.5 | 2.88 | 0.96 | 3.22 | 0.77 | 1.11 | 1.45 | 1.01 | 2.63 | 1.44 |
| 0.9 | 0.89 | 0.56 | 1.06 | 0.67 | 1.06 | 1.23 | 0.94 | 0.99 | 1.35 | 1.14 |
| 6.01 | 19.75 | 1.16 | 1.13 | 7.74 | 0.6 | 1.57 | 0.6 | 1.17 | 0.66 | 1.07 |
| 0.74 | 0.33 | 0.43 | 0.85 | 0.07 | 1.42 | 0.77 | 5.73 | 0.99 | 2.58 | 1.06 |
| 5.45 | 0.89 | 0.74 | 1.07 | 0.8 | 1.7 | 0.67 | 0.72 | 0.91 | 3.69 | 0.7 |
| 1.2 | 4.8 | 1.65 | 0.97 | 0.94 | 9.19 | 0.55 | 0.85 | 0.89 | 0.71 | 0.28 |
| 0.64 | 0.79 | 4.69 | 1.25 | 2.48 | 63.33 | 1.39 | 1.21 | 1.24 | 4.24 | 0.75 |
| 1.96 | 1357.58 | 3.92 | 4.5 | 2.85 | 0.76 | 0.44 | 1.24 | 1.52 | 12.32 | 0.4 |
| 0.62 | 18.33 | 1.24 | 1.26 | 4.39 | 0.7 | 1.33 | 1.54 | 1.16 | 0.81 | 0.52 |
| 0.59 | 14.5 | 1.8 | 1.73 | 0.2 | 4.27 | 1.89 | 6.08 | 3.73 | 2.97 | 0.62 |
| 0.5 | 0.84 | 2.12 | 0.73 | 3.8 | 1.18 | 4.16 | 1.78 | 0.6 | 3.8 | 2.1 |
| 0.82 | 0.59 | 1.14 | 0.8 | 0.67 | 0.5 | 1.53 | 4.1 | 0.57 | 0.76 | 0.94 |
| 0.46 | 1.75 | 2.01 | 4.35 | 0.58 | 1.36 | 2.55 | 4.97 | 4.1 | 1.48 | 1.64 |
| 0.67 | 10.49 | 3.28 | 1.27 | 2.51 | 0.83 | 1.38 | 2.33 | 1.06 | 3.45 | 1.02 |
| 1.04 | 1.75 | 1.1 | 0.73 | 0.91 | 0.66 | 0.6 | 0.6 | 0.55 | 1.08 | 3.43 |
| 142.34 | 14 | 3 | 0.83 | 1.37 | 4.08 | 1.42 | 1.04 | 1.08 | 1.39 | 4.43 |

| | | | | | | | | | | |
|---|---|---|---|---|---|---|---|---|---|---|
| 0.91 | 4.85 | 0.87 | 1.25 | 2.44 | 0.93 | 0.72 | 0.81 | 1.1 | 0.47 | 1.18 |
| 1.85 | 33.32 | 0.98 | 1.51 | 15.99 | 0.95 | 1.01 | 1.08 | 0.98 | 6.46 | 2.13 |
| 1 | 1.05 | 5.94 | 0.98 | 4.18 | 1.11 | 0.99 | 1.68 | 1.24 | 0.4 | 0.98 |
| 1.37 | 1.33 | 0.73 | 0.99 | 1.15 | 10.4 | 1.4 | 1.41 | 1.16 | 1.32 | 1.57 |
| 1.04 | 13.48 | 3.53 | 0.89 | 7.59 | 0.94 | 0.8 | 0.94 | 1.47 | 3.48 | 0.7 |
| 1.04 | 1.43 | 0.95 | 1.38 | 2.03 | 12.57 | 1.06 | 1.07 | 2.08 | 0.91 | 0.78 |
| 1.83 | 5.14 | 1.12 | 1.06 | 0.66 | 0.83 | 0.33 | 0.34 | 1.53 | 2.2 | 1.29 |
| 1.1 | 0.51 | 2.44 | 1.63 | 3 | 0.93 | 1.35 | 1.55 | 0.83 | 0.6 | 1.4 |
| 1.08 | 7.88 | 0.56 | 1.36 | 4.57 | 1.76 | 1.14 | 1.33 | 1.6 | 4.97 | 0.21 |
| 0.8 | 1.6 | 1.22 | 0.78 | 0.23 | 4.09 | 0.66 | 1.05 | 0.91 | 0.9 | 1.23 |
| 2.8 | 2.17 | 1.22 | 0.37 | 1.06 | 0.82 | 0.67 | 0.71 | 1.54 | 4.49 | 1.54 |
| 0.74 | 2.57 | 1.54 | 1.03 | 0.67 | 1.14 | 0.86 | 1.14 | 0.77 | 0.25 | 1.29 |
| 1.33 | 4.5 | 0.62 | 0.98 | 2.14 | 1.05 | 1.08 | 1.14 | 1.03 | 1.04 | 0.6 |
| 2 | 1.88 | 1.86 | 1.11 | 1.53 | 1.06 | 1.49 | 2.69 | 1.43 | 2.46 | 1.41 |
| 1 | 4 | 5.83 | 1.88 | 5.33 | 1.48 | 0.49 | 0.93 | 0.59 | 0.5 | 0.51 |
| 52.03 | 259.49 | 0.7 | 2.17 | 0.02 | 0.17 | 0.56 | 2.52 | 2.17 | 0.81 | 12.87 |
| 0.53 | 3.76 | 2.55 | 0.6 | 5.5 | 1.96 | 1.75 | 2.22 | 2.18 | 0.79 | 4.02 |
| 0.13 | 582.67 | 1.41 | 0.84 | 0.9 | 0.43 | 0.75 | 0.98 | 1.4 | 0.81 | 7.75 |
| 1.22 | 17.25 | 1.83 | 0.85 | 1.39 | 1.55 | 1.68 | 0.74 | 1.57 | 1.01 | 0.8 |
| 1.02 | 0.59 | 1.15 | 1.88 | 3.88 | 3.6 | 3.55 | 1.34 | 1.34 | 1.89 | 5.4 |
| 0.33 | 141.02 | 0.77 | 1.08 | 26.13 | 13.2 | 2.36 | 1.62 | 3.51 | 1.03 | 1.17 |
| 1.17 | 0.23 | 1.11 | 1.24 | 2.61 | 1.8 | 0.93 | 1.35 | 2.22 | 0.53 | 0.92 |
| 0.87 | 0.17 | 0.77 | 1.6 | 0.08 | 0.68 | 0.68 | 1.12 | 0.93 | 1.92 | 0.9 |
| 0.51 | 4.13 | 1.29 | 1.52 | 6.31 | 0.83 | 0.71 | 0.94 | 0.53 | 0.98 | 1.17 |
| 1.04 | 0.88 | 1.04 | 1.14 | 1.57 | 0.76 | 1.18 | 0.79 | 0.65 | 2 | 0.43 |
| 1.07 | 1.63 | 2.47 | 0.9 | 0.81 | 0.93 | 1.49 | 1.55 | 0.62 | 1.32 | 1.02 |
| 1.28 | 1.13 | 2.89 | 1.17 | 7 | 0.91 | 1.06 | 1.22 | 0.83 | 0.74 | 1.22 |
| 1.31 | 0.9 | 0.98 | 1.34 | 0.8 | 1.67 | 1.25 | 0.93 | 1.3 | 1.7 | 1.03 |
| 1.07 | 1.44 | 1.61 | 0.89 | 0.57 | 1.53 | 1.22 | 0.99 | 2.14 | 0.38 | 1.23 |
| 1.03 | 1.78 | 0.83 | 1.07 | 1.08 | 0.87 | 0.86 | 1.32 | 1.29 | 1.62 | 1.11 |
| 0.83 | 1.7 | 2.29 | 1.25 | 8.66 | 1.15 | 1.67 | 1.48 | 0.76 | 2.13 | 1.18 |
| 0.62 | 0.44 | 0.49 | 1.85 | 17.04 | 0.97 | 0.7 | 0.41 | 1.27 | 1.44 | 0.46 |
| 0.77 | 1.23 | 2.21 | 0.91 | 7.54 | 1.67 | 1.87 | 2.25 | 7.1 | 0.83 | 0.69 |

| | | | | | | | | | | |
|---|---|---|---|---|---|---|---|---|---|---|
| 2.26 | 3.8 | 1.61 | 0.69 | 2.44 | 1.43 | 0.63 | 0.75 | 1.78 | 1.83 | 0.74 |
| 0.92 | 0.95 | 0.82 | 1.23 | 1.83 | 0.75 | 1.23 | 1 | 0.7 | 0.67 | 1.17 |
| 1.59 | 3.15 | 2.91 | 1.26 | 2.65 | 1.8 | 1.59 | 1.15 | 1.17 | 1.37 | 1.23 |
| 0.6 | 2.92 | 3.38 | 1.5 | 2.18 | 0.83 | 0.8 | 1.18 | 1.45 | 2.11 | 0.36 |
| 1.17 | 1.96 | 1.36 | 0.9 | 0.9 | 8.89 | 0.54 | 0.85 | 0.76 | 0.71 | 1.05 |
| 0.62 | 0.19 | 0.79 | 1.74 | 1.82 | 0.7 | 0.31 | 3.58 | 0.44 | 1.2 | 5.61 |
| 1.52 | 5.79 | 1.63 | 1.2 | 2.07 | 1.05 | 1.32 | 1.89 | 1.03 | 1.9 | 1.13 |
| 0.71 | 5.04 | 1.34 | 1.04 | 1.22 | 1.11 | 0.87 | 1.02 | 1.43 | 1.09 | 0.9 |
| 1.65 | 0.67 | 1.9 | 0.73 | 0.68 | 3.2 | 1.13 | 0.64 | 1.14 | 2.66 | 0.6 |
| 1.39 | 1.29 | 0.92 | 0.79 | 6.6 | 5.15 | 2.04 | 1.71 | 3.71 | 6.73 | 1.2 |
| 0.65 | 0.93 | 1.5 | 1.17 | 2.2 | 0.95 | 1.38 | 0.88 | 0.93 | 5.44 | 0.84 |
| 1.26 | 1.51 | 2.24 | 1.04 | 1.12 | 1.17 | 0.88 | 0.89 | 0.84 | 1.31 | 1.77 |
| 1.03 | 0.42 | 2.38 | 1.18 | 1.5 | 1.44 | 2.12 | 1.41 | 0.9 | 5.96 | 0.67 |
| 0.84 | 0.93 | 2.61 | 14.7 | 0.24 | 0.69 | 0.82 | 0.68 | 0.52 | 1.88 | 0.65 |
| 2.56 | 0.34 | 1.18 | 0.9 | 0.72 | 0.73 | 2.1 | 1.04 | 1.23 | 0.86 | 0.87 |
| 1.01 | 1.08 | 1.09 | 1.06 | 1.71 | 1.51 | 0.74 | 1.31 | 2.01 | 0.87 | 2 |
| 1.32 | 0.99 | 3.28 | 0.94 | 0.39 | 0.23 | 0.87 | 0.83 | 0.49 | 2.21 | 2.37 |
| 1.75 | 1.34 | 0.62 | 1.12 | 0.87 | 9.73 | 1.39 | 2.39 | 1.74 | 1.1 | 1.81 |
| 0.97 | 0.64 | 2.74 | 0.85 | 9.85 | 0.92 | 1.45 | 4.17 | 0.8 | 2.29 | 0.45 |
| 0.81 | 0.38 | 2.26 | 1.4 | 0.71 | 3.21 | 1.77 | 0.66 | 1.36 | 2.12 | 1.67 |
| 0.85 | 0.29 | 1.36 | 0.9 | 0.57 | 1.03 | 0.29 | 0.73 | 0.57 | 1.1 | 1.21 |
| 1.09 | 0.97 | 1.14 | 0.96 | 0.66 | 2.17 | 0.77 | 1 | 1.14 | 0.69 | 1.37 |
| 1.21 | 1.85 | 1.6 | 0.95 | 1.09 | 1.23 | 1.4 | 1.05 | 0.75 | 1.22 | 1.23 |
| 1.73 | 0.18 | 0.78 | 0.82 | 0.68 | 1.47 | 0.98 | 0.52 | 4.37 | 1.15 | 0.76 |
| 0.87 | 1.79 | 0.62 | 0.99 | 3.83 | 0.75 | 1.34 | 0.48 | 0.39 | 1.25 | 2.7 |
| 1.76 | 0.93 | 0.85 | 0.8 | 2.1 | 1.13 | 0.9 | 0.88 | 1.26 | 0.45 | 1.99 |
| 1.23 | 1.31 | 3.22 | 1.13 | 9.62 | 0.62 | 1.06 | 1.14 | 1.03 | 5 | 1.07 |
| 2.06 | 1.33 | 1.13 | 0.57 | 11.53 | 0.82 | 0.91 | 1.19 | 1.57 | 3.88 | 0.14 |
| 1.32 | 0.67 | 0.82 | 0.93 | 1.92 | 2.3 | 1.03 | 0.58 | 0.67 | 0.89 | 1.07 |
| 0.9 | 0.8 | 1.08 | 0.94 | 3.08 | 0.93 | 1.01 | 0.98 | 1.01 | 1 | 1.59 |
| 1.04 | 0.93 | 1.96 | 1.11 | 1 | 0.82 | 1.18 | 1.24 | 1.06 | 0.95 | 0.58 |
| 1.85 | 33.32 | 0.98 | 1.51 | 15.99 | 0.95 | 1.01 | 1.08 | 0.98 | 6.46 | 2.13 |
| 1.59 | 1.78 | 1.09 | 0.9 | 12.67 | 1.21 | 0.91 | 3.55 | 0.84 | 0.67 | 1.39 |

| | | | | | | | | | | |
|---|---|---|---|---|---|---|---|---|---|---|
| 1.3 | 2.01 | 1.89 | 1 | 0.47 | 0.43 | 1.01 | 0.72 | 1.16 | 0.48 | 1.09 |
| 1.04 | 0.19 | 1.54 | 0.92 | 4.9 | 4.42 | 1.51 | 1.75 | 0.81 | 4.25 | 1.21 |
| 1.12 | 0.93 | 0.96 | 1.13 | 1.28 | 0.63 | 0.87 | 0.78 | 1.3 | 1.34 | 1.48 |
| 1.08 | 1.12 | 0.95 | 1.01 | 1.75 | 6.11 | 0.66 | 3 | 0.93 | 3.09 | 4.39 |
| 0.97 | 1.12 | 1.15 | 1.04 | 3.28 | 1.02 | 0.96 | 0.53 | 0.79 | 0.58 | 3.07 |
| 1.1 | 0.86 | 0.67 | 0.92 | 5 | 0.94 | 0.88 | 0.93 | 0.99 | 0.33 | 1.07 |
| 1.19 | 1.86 | 5.77 | 0.9 | 2.7 | 0.91 | 1.11 | 0.96 | 0.85 | 1.37 | 1.09 |
| 0.84 | 1.26 | 1 | 0.75 | 1.63 | 1.12 | 1.32 | 0.67 | 1.16 | 0.95 | 1.26 |
| 0.65 | 0.21 | 1.14 | 1.01 | 1.85 | 1.25 | 1.3 | 1.02 | 1.08 | 0.64 | 1.12 |
| 0.84 | 1.09 | 1.31 | 1.28 | 5.89 | 1.09 | 0.9 | 1.89 | 0.54 | 1.63 | 1.07 |
| 1.09 | 6.75 | 2.2 | 1.1 | 0.59 | 4.83 | 1.17 | 1.21 | 1.48 | 1.36 | 0.72 |
| 1.15 | 7.92 | 1 | 1.26 | 2.35 | 1.06 | 1.06 | 1.52 | 2.06 | 1.18 | 4.89 |
| 1.6 | 1.88 | 0.73 | 1.05 | 1.25 | 6.21 | 1.21 | 1.46 | 1.24 | 1.29 | 2.77 |
| 0.93 | 1.56 | 1.3 | 0.89 | 3.62 | 1.8 | 1.39 | 1.73 | 0.69 | 1.21 | 1.3 |
| 0.41 | 0.59 | 0.64 | 0.84 | 1.33 | 1.57 | 1.56 | 1.3 | 22.54 | 3.82 | 0.09 |
| 0.87 | 3.02 | 3 | 1.05 | 0.82 | 0.94 | 0.98 | 0.68 | 0.88 | 1.45 | 0.74 |
| 0.92 | 4.55 | 1.43 | 1.1 | 4.4 | 1.48 | 1.16 | 0.87 | 1.22 | 7.15 | 1.01 |
| 2.9 | 1.75 | 1.46 | 1.19 | 1.71 | 0.81 | 1.29 | 1.29 | 1.17 | 1.47 | 0.65 |
| 1.03 | 2.87 | 0.79 | 0.83 | 1.24 | 1.09 | 1.09 | 1.01 | 0.98 | 5.39 | 0.97 |
| 0.77 | 1.16 | 1.09 | 1.07 | 0.42 | 0.86 | 0.76 | 0.57 | 0.78 | 0.81 | 0.41 |
| 0.49 | 2.1 | 0.89 | 1.12 | 0.96 | 0.87 | 1.35 | 1.31 | 1.17 | 1.3 | 1.24 |
| 0.46 | 2.96 | 3 | 0.66 | 0.57 | 1.21 | 0.88 | 1.19 | 0.84 | 1.62 | 0.57 |
| 0.85 | 0.94 | 1.18 | 1.35 | 1.49 | 1.84 | 0.73 | 0.86 | 0.93 | 1.31 | 0.89 |
| 0.68 | 1.25 | 1.05 | 0.92 | 1.38 | 0.76 | 1.01 | 0.69 | 0.49 | 1.16 | 1.74 |
| 0.94 | 0.76 | 0.96 | 2.54 | 2.26 | 2.28 | 0.89 | 1.04 | 0.96 | 1.93 | 1.34 |
| 0.89 | 1.69 | 3.74 | 1.42 | 1.64 | 0.85 | 1.01 | 1.19 | 0.6 | 1.03 | 1.23 |
| 0.88 | 1.15 | 1.45 | 1.28 | 2.68 | 7.83 | 1.04 | 3.06 | 0.29 | 1.02 | 1.03 |
| 1.4 | 2.29 | 9.2 | 1.06 | 0.67 | 1.9 | 0.98 | 0.94 | 1.09 | 3.04 | 1.68 |
| 0.67 | 0.78 | 1.34 | 1.03 | 3.19 | 1.02 | 0.93 | 0.81 | 1 | 1.78 | 1 |
| 0.93 | 1.56 | 1.02 | 0.98 | 0.11 | 0.96 | 0.96 | 2 | 0.89 | 11 | 1.2 |
| 0.72 | 0.91 | 1.19 | 1.14 | 1.72 | 1.23 | 0.87 | 0.78 | 1.27 | 3 | 1.05 |
| 1.01 | 3.12 | 3.31 | 1.01 | 1.79 | 1.1 | 1.6 | 1.66 | 1.34 | 3.07 | 0.98 |
| 0.94 | 0.9 | 0.64 | 1.12 | 0.58 | 1 | 0.99 | 1.06 | 1.35 | 0.65 | 1.04 |

| | | | | | | | | | | |
|---|---|---|---|---|---|---|---|---|---|---|
| 0.81 | 2.87 | 1.16 | 1.86 | 2.44 | 2.97 | 1.15 | 1.15 | 2.41 | 5.39 | 0.46 |
| 0.89 | 1.21 | 1.47 | 1.31 | 1.85 | 2.29 | 0.9 | 0.74 | 1.46 | 0.85 | 1.08 |
| 0.96 | 2.17 | 1.33 | 1 | 0.7 | 0.85 | 0.86 | 1.04 | 1.09 | 1.17 | 1.2 |
| 0.77 | 2 | 0.65 | 0.53 | 1.53 | 0.77 | 1.2 | 1.07 | 1.03 | 3.73 | 1.41 |
| 0.68 | 0.32 | 0.62 | 1.13 | 3.67 | 1.66 | 1.07 | 1.3 | 1.95 | 2.71 | 71.03 |
| 0.92 | 1.21 | 1.01 | 1.08 | 1.65 | 0.84 | 1.02 | 1.19 | 1.13 | 1.02 | 1.55 |
| 1.4 | 0.41 | 1.1 | 1.55 | 3.33 | 1.35 | 0.6 | 0.66 | 0.17 | 7.05 | 0.86 |
| 2.13 | 1.49 | 1.51 | 0.81 | 1.18 | 0.93 | 3.29 | 0.87 | 1.1 | 1.61 | 1.49 |
| 0.71 | 4.22 | 0.8 | 0.91 | 0.38 | 1.02 | 1.25 | 1.45 | 1.2 | 2.07 | 4.89 |
| 1.63 | 0.52 | 0.96 | 1.38 | 2.22 | 1.29 | 0.49 | 0.62 | 0.59 | 4.85 | 0.36 |
| 0.9 | 1.62 | 1 | 1.09 | 0.07 | 4 | 1.11 | 1.18 | 2.43 | 3.67 | 0.37 |
| 0.93 | 0.96 | 0.82 | 1.8 | 1.29 | 1.04 | 0.72 | 0.9 | 0.72 | 1.08 | 0.89 |
| 0.98 | 0.89 | 1.71 | 0.9 | 0.24 | 0.93 | 0.68 | 0.87 | 1.04 | 1.12 | 0.97 |
| 0.92 | 0.9 | 1.21 | 0.86 | 2 | 1.05 | 0.93 | 0.92 | 1.46 | 1.75 | 1.42 |
| 0.97 | 1.17 | 0.87 | 0.82 | 2.85 | 3.72 | 0.53 | 3.13 | 0.98 | 0.88 | 4.63 |
| 1.52 | 1.72 | 0.99 | 0.99 | 0.06 | 0.48 | 1.24 | 1.35 | 1.45 | 1.16 | 1.21 |
| 0.86 | 1.18 | 0.86 | 0.96 | 1.56 | 0.81 | 0.84 | 0.96 | 1.42 | 1.87 | 0.83 |
| 0.97 | 0.5 | 0.72 | 0.91 | 0.83 | 1.58 | 0.83 | 1.89 | 0.94 | 3.61 | 1.86 |
| 1.24 | 4.57 | 0.76 | 0.57 | 0.77 | 1.07 | 0.91 | 0.83 | 1.22 | 0.41 | 0.77 |
| 0.99 | 0.56 | 1.22 | 0.78 | 0.34 | 1.18 | 0.49 | 0.77 | 0.91 | 1.49 | 0.56 |
| 1.01 | 1.69 | 5.27 | 1.02 | 0.85 | 1.02 | 1.02 | 1.13 | 1.16 | 4.12 | 0.76 |
| 1.33 | 0.01 | 1.09 | 2.17 | 0.89 | 0.54 | 10.48 | 3 | 0.17 | 2.63 | 5 |
| 1.34 | 0.91 | 0.96 | 1.08 | 1.08 | 1.3 | 0.88 | 0.91 | 0.57 | 2.33 | 1.57 |
| 0.89 | 6.63 | 0.91 | 1.18 | 2.15 | 3.15 | 0.72 | 1 | 1.05 | 5.37 | 0.21 |
| 1.27 | 1.56 | 1.16 | 0.98 | 0.9 | 0.75 | 0.97 | 0.98 | 0.92 | 0.83 | 0.63 |
| 0.27 | 1.4 | 2.52 | 0.54 | 0.79 | 0.02 | 0.8 | 0.21 | 1.7 | 0.11 | 1.81 |
| 0.84 | 0.75 | 6.73 | 1 | 3.57 | 1.04 | 0.97 | 1.11 | 1.29 | 1.02 | 0.98 |
| 0.93 | 1.96 | 1.86 | 1.16 | 1.11 | 0.99 | 1.07 | 1.06 | 0.64 | 1.26 | 0.95 |
| 0.93 | 6.34 | 1.03 | 1.04 | 1.57 | 0.98 | 0.94 | 0.97 | 1.28 | 0.69 | 0.98 |
| 0.86 | 1.01 | 0.28 | 1.18 | 0.64 | 0.42 | 1.03 | 0.92 | 0.55 | 4.39 | 1.43 |
| 1.49 | 1.46 | 2.46 | 1.51 | 5.95 | 1.55 | 1.12 | 0.86 | 0.93 | 0.85 | 1.18 |
| 0.88 | 2 | 0.61 | 1.02 | 2 | 1.39 | 0.91 | 1.07 | 0.98 | 0.08 | 0.99 |
| 0.75 | 0.59 | 2.98 | 0.95 | 1.97 | 2 | 0.66 | 1.1 | 1.22 | 2.41 | 0.83 |

| | | | | | | | | | | |
|---|---|---|---|---|---|---|---|---|---|---|
| 0.74 | 4.83 | 0.7 | 2.12 | 1 | 0.89 | 2.89 | 1.53 | 0.86 | 1.12 | 0.89 |
| 1.34 | 0.87 | 0.71 | 0.85 | 1 | 1.02 | 1.04 | 0.52 | 0.76 | 1.62 | 2.93 |
| 0.78 | 4.53 | 2.45 | 0.95 | 1.28 | 1.2 | 1.1 | 0.98 | 1 | 1.33 | 0.58 |
| 0.87 | 0.05 | 1.56 | 0.84 | 1.06 | 0.73 | 0.52 | 0.54 | 1.55 | 0.48 | 2.04 |
| 0.93 | 1.48 | 1.09 | 1.13 | 1.56 | 0.92 | 1.07 | 0.73 | 2.32 | 1.01 | 0.53 |
| 0.95 | 1.2 | 0.88 | 0.85 | 1.49 | 1.78 | 0.82 | 1.08 | 0.7 | 1.26 | 1 |
| 0.8 | 0.49 | 0.48 | 0.93 | 0.55 | 1.1 | 0.82 | 0.48 | 1.21 | 0.58 | 1.2 |
| 0.78 | 0.97 | 3.14 | 1.06 | 0.46 | 1.33 | 0.96 | 0.85 | 1.29 | 0.58 | 1.12 |
| 0.83 | 3.88 | 1.03 | 1.32 | 2.43 | 0.72 | 1.05 | 1.46 | 1.33 | 2.42 | 0.47 |
| 0.41 | 5.04 | 0.49 | 0.73 | 0.93 | 4.82 | 0.69 | 1.23 | 0.32 | 1.79 | 1.26 |
| 1.1 | 0.93 | 1.33 | 0.81 | 1.34 | 0.87 | 1.1 | 1.13 | 0.98 | 1.12 | 1.17 |
| 0.89 | 1.5 | 5.27 | 0.97 | 0.29 | 0.89 | 1.28 | 0.77 | 0.7 | 1.05 | 0.97 |
| 1.17 | 4.08 | 0.43 | 0.86 | 1 | 0.5 | 0.7 | 3.55 | 1.6 | 0.32 | 0.67 |
| 0.75 | 0.91 | 0.43 | 0.92 | 0.83 | 0.68 | 1.44 | 1.11 | 1.12 | 0.71 | 0.71 |
| 0.81 | 1.74 | 0.67 | 0.75 | 1.38 | 1.47 | 1.3 | 1.2 | 1.68 | 2.04 | 0.96 |
| 0.44 | 3.19 | 2.96 | 0.88 | 5.94 | 0.97 | 0.91 | 0.56 | 0.6 | 2.39 | 1.29 |
| 0.98 | 0.24 | 1.15 | 0.87 | 0.18 | 0.75 | 1.16 | 0.82 | 1.09 | 1.27 | 0.4 |
| 0.68 | 0.94 | 1.23 | 1.09 | 0.9 | 0.47 | 1.1 | 0.78 | 0.81 | 1.02 | 1.57 |
| 42.28 | 0.58 | 6.79 | 0.92 | 0.92 | 2.83 | 1.59 | 1.21 | 1.77 | 8.75 | 1.58 |
| 0.35 | 12.38 | 0.53 | 0.41 | 0.35 | 0.93 | 1.31 | 1.07 | 0.27 | 3 | 1.06 |
| 1.71 | 15.33 | 2.23 | 0.96 | 3.21 | 0.76 | 6.07 | 0.83 | 2.27 | 0.8 | 1.12 |
| 1.49 | 0.48 | 0.81 | 1.45 | 3.56 | 1.06 | 0.52 | 0.64 | 0.5 | 4.99 | 0.56 |
| 1.32 | 0.06 | 1.3 | 1.31 | 0.38 | 1.68 | 0.69 | 0.37 | 1.08 | 0.75 | 0.45 |
| 1.93 | 0.03 | 1.37 | 1.27 | 1.15 | 0.43 | 0.76 | 0.35 | 2.78 | 1.43 | 0.71 |
| 0.8 | 1.08 | 0.71 | 1.11 | 1.19 | 1.09 | 1.06 | 1.23 | 1.52 | 1.32 | 3.24 |
| 0.72 | 7.56 | 2.16 | 1.26 | 0.91 | 1.32 | 1.32 | 1.15 | 0.69 | 0.82 | 1.48 |
| 1.1 | 0.25 | 1.1 | 0.63 | 0.79 | 1.45 | 1.94 | 0.36 | 0.44 | 0.75 | 2.75 |
| 0.68 | 0.29 | 1.13 | 0.99 | 6 | 0.86 | 0.7 | 1.34 | 0.85 | 3.55 | 0.56 |
| 0.72 | 1.55 | 4.43 | 1.47 | 51.05 | 1.47 | 1.06 | 2.01 | 1.49 | 1.23 | 2.42 |
| 0.27 | 2 | 0.2 | 1.83 | 98.75 | 0.31 | 2.38 | 0.26 | 1.73 | 1.32 | 1.62 |
| 0.64 | 0.65 | 2.69 | 1.19 | 0.75 | 1.11 | 1.25 | 0.87 | 2.09 | 1.33 | 1.63 |
| 0.11 | 1.06 | 2.05 | 1.48 | 0.88 | 1.48 | 2.62 | 1.68 | 2.45 | 1.1 | 7.44 |
| 1.44 | 2.41 | 6.11 | 1.41 | 1.17 | 9.78 | 4.14 | 1.19 | 0.35 | 1.03 | 2.12 |

| | | | | | | | | | | |
|---|---|---|---|---|---|---|---|---|---|---|
| 0.86 | 0.85 | 0.73 | 1.16 | 9.5 | 4 | 2.8 | 0.88 | 1.14 | 0.87 | 0.98 |
| 1.17 | 1.41 | 1.25 | 1.17 | 0.66 | 1.2 | 1.47 | 1.21 | 1.2 | 2.53 | 0.9 |
| 1.02 | 1.89 | 3.18 | 0.52 | 0.21 | 9.83 | 1.76 | 0.73 | 4.07 | 1.12 | 0.4 |
| 0.45 | 65.11 | 0.52 | 0.85 | 1.06 | 2.05 | 0.65 | 0.92 | 2.28 | 0.16 | 1.04 |
| 0.82 | 0.3 | 0.56 | 0.78 | 0.96 | 1.83 | 0.68 | 0.84 | 0.62 | 2.07 | 1.21 |
| 1.32 | 1.9 | 0.75 | 1.27 | 3.05 | 1.3 | 1.23 | 1.05 | 2.54 | 1.17 | 0.74 |
| 0.89 | 2.62 | 0.93 | 1.1 | 1.8 | 0.92 | 1.41 | 1.14 | 1.92 | 0.69 | 1.1 |
| 1.09 | 23.33 | 1.07 | 1.06 | 1.63 | 1.28 | 1.06 | 0.8 | 0.88 | 0.92 | 0.04 |
| 0.78 | 0.7 | 0.64 | 1.42 | 1.35 | 0.89 | 0.99 | 1.33 | 2.04 | 1.02 | 4 |
| 0.57 | 0.83 | 1.42 | 1.07 | 5.84 | 1.01 | 1.12 | 1.19 | 1.26 | 1.53 | 0.33 |
| 0.87 | 1.13 | 1.81 | 1.04 | 0.48 | 0.69 | 0.93 | 1.14 | 0.89 | 2.83 | 4.98 |
| 14.57 | 4.06 | 0.31 | 0.44 | 0.54 | 1.85 | 1.37 | 2.33 | 107.71 | 0.83 | 0.43 |
| 0.89 | 1.54 | 4.01 | 1.46 | 0.33 | 1.3 | 0.96 | 0.29 | 1.96 | 0.69 | 1.14 |
| 0.76 | 2.5 | 0.43 | 1.14 | 1 | 2.12 | 1.36 | 0.76 | 0.34 | 2.29 | 1.48 |

| 203238_s_at | 201288_at | 204236_at | 205469_s_at | 207059_at | 214435_x_at | 204990_s_at | 206943_at | 211370_s_at | 206645_s_at | 201417_at |
|---|---|---|---|---|---|---|---|---|---|---|
| 1.1 | 2.81 | 2.9 | 1.32 | 1.45 | 1.11 | 16.02 | 4.01 | 0.67 | 2 | 0.8 |
| 1.07 | 3.56 | 1.39 | 0.97 | 1.17 | 1.49 | 1.6 | 1.08 | 0.94 | 0.66 | 1.52 |
| 2.05 | 0.82 | 1.13 | 0.79 | 1.43 | 1.28 | 1.45 | 0.57 | 9.06 | 0.39 | 0.65 |
| 1.06 | 1.13 | 3.27 | 0.92 | 1.36 | 1.13 | 1.16 | 0.99 | 2.8 | 2.28 | 0.9 |
| 1.07 | 3.08 | 2.24 | 1.02 | 1.03 | 0.96 | 6.01 | 1.08 | 1.13 | 1.5 | 0.86 |
| 1.13 | 0.98 | 1.08 | 1.37 | 3.92 | 0.89 | 6.11 | 4.08 | 23.33 | 2.74 | 0.48 |
| 0.78 | 1.53 | 4.08 | 0.31 | 0.58 | 1.54 | 0.88 | 4.56 | 0.73 | 0.24 | 0.69 |
| 1.08 | 0.89 | 0.81 | 0.99 | 0.76 | 0.81 | 3.73 | 0.57 | 1.16 | 0.33 | 0.91 |
| 1.05 | 1.47 | Inf | 0.97 | 1.57 | 1.2 | 1.19 | 0.92 | 0.96 | 1.27 | 0.56 |
| 0.15 | 0.49 | 0.88 | 1.64 | 0.75 | 1.35 | 0.3 | 1.11 | 0.44 | 1.53 | 1.3 |
| 1.34 | 0.74 | 1.04 | 0.93 | 0.84 | 0.84 | 3.59 | 1.88 | 1.04 | 1.17 | 1.15 |
| 2.3 | 0.85 | 0.93 | 0.7 | 1.26 | 0.93 | 0.81 | 0.85 | 1.14 | 0.61 | 0.79 |
| 1.08 | 1.12 | 0.95 | 0.96 | 0.75 | 0.66 | 0.32 | 0.79 | 1.1 | 3.58 | 1.18 |
| 0.47 | 0.42 | 22.5 | 2.03 | 3.36 | 0.55 | 0.66 | 6.21 | 7.52 | 10.82 | 26.24 |
| 2.58 | 0.81 | 0.88 | 0.54 | 1.51 | 0.54 | 1.81 | 1.75 | 1.65 | 1.07 | 0.84 |
| 1.15 | 0.12 | 0.43 | 0.65 | 0.91 | 0.87 | 1.14 | 0.72 | 0.9 | 0.31 | 3.45 |
| 0.98 | 2.52 | 1.29 | 0.63 | 0.73 | 1.22 | 0.88 | 0.39 | 1.59 | 0.62 | 0.42 |
| 3.44 | 0.87 | 1.26 | 1.36 | 1.45 | 1.14 | 0.86 | 1.08 | 1.06 | 2.42 | 2.66 |
| 0.4 | 2.6 | 0.78 | 1.45 | 1.43 | 1.04 | 3.63 | 1.31 | 1.25 | 0.67 | 0.42 |
| 1.74 | 1.89 | 0.23 | 0.45 | 2.52 | 1.03 | 6.39 | 0.56 | 1.77 | 3.42 | 0.59 |
| 2.57 | 1.08 | 0.63 | 1.4 | 0.94 | 0.71 | 3.31 | 0.79 | 0.43 | 1.48 | 2.37 |
| 9.08 | 0.68 | 7.01 | 17.64 | 5.3 | 0.59 | 1.65 | 6.92 | 1.64 | 2.79 | 0.68 |
| 1.21 | 1.38 | 0.85 | 2.24 | 1.42 | 0.87 | 4.61 | 0.78 | 1.17 | 0.38 | 1.69 |
| 4.9 | 0.4 | 1.65 | 0.72 | 0.76 | 1.02 | 0.07 | 0.45 | 1.11 | 0.47 | 0.26 |
| 0.3 | 170.23 | 0.99 | 0.57 | 0.59 | 0.93 | 10.1 | 0.11 | 0.48 | 1.38 | 0.06 |
| 2.75 | 1.23 | 2.16 | 0.79 | 0.92 | 1.29 | 0.67 | 10.2 | 1.41 | 1.88 | 1.06 |
| 1.06 | 0.41 | 0.12 | 0.55 | 0.97 | 0.8 | 0.95 | 1.1 | 1.01 | 7.28 | 2.53 |
| 2.31 | 0.47 | 11.75 | 7.36 | 6.8 | 1 | 0.96 | 4.26 | 1.29 | 1.97 | 1.28 |
| 0.42 | 1.74 | 5.62 | 1.33 | 1.34 | 0.72 | 11.44 | 3.81 | 1.92 | 0.13 | 3.83 |
| 0.7 | 2.67 | 0.94 | 1.68 | 1.25 | 0.87 | 3.81 | 0.43 | 0.68 | 1.18 | 1.27 |

| | | | | | | | | | | |
|---|---|---|---|---|---|---|---|---|---|---|
| 2.42 | 0.79 | 0.58 | 0.47 | 0.84 | 1.19 | 2.11 | 2.18 | 0.63 | 3.11 | 0.3 |
| 0.19 | 1.95 | 0.16 | 0.22 | 1.36 | 1.87 | 0.17 | 2.22 | 1.85 | 21.47 | 0.67 |
| 0.98 | 0.77 | 4.27 | 1.15 | 1.12 | 1.13 | 0.87 | 1.54 | 1.02 | 1.7 | 1.1 |
| 0.61 | 0.87 | 1.23 | 0.96 | 1.02 | 1.39 | 0.4 | 0.98 | 1.5 | 4.4 | 0.57 |
| 0.35 | 0.09 | 1.14 | 1.14 | 1.63 | 0.85 | 5.61 | 2.57 | 1.17 | 25.67 | 2 |
| 0.66 | 3.02 | 6.95 | 1.38 | 6.24 | 1.36 | 16.97 | 1.23 | 1.13 | 1.16 | 0.53 |
| 1.83 | 0.31 | 0.21 | 1.28 | 1.44 | 1.03 | 1.01 | 0.84 | 1.35 | 1.75 | 3.29 |
| 1.06 | 0.75 | 0.71 | 1.09 | 0.75 | 0.97 | 1.86 | 0.82 | 0.6 | 2.19 | 1.25 |
| 1.71 | 1.43 | 7.45 | 0.51 | 0.86 | 0.88 | 1.46 | 0.57 | 0.94 | 1.01 | 1.32 |
| 0.43 | 0.17 | 0.31 | 1.04 | 0.58 | 2.25 | 2.26 | 2.56 | 1.04 | 1.59 | 1.1 |
| 1.14 | 1.41 | 1 | 3.73 | 1.18 | 1.28 | 3.03 | 0.21 | 1.37 | 6.7 | 0.97 |
| 1.21 | 1.1 | 1.36 | 0.44 | 1.16 | 1.01 | 0.41 | 1.04 | 0.96 | 1.56 | 1.05 |
| 1.83 | 0.17 | 0.24 | 1.2 | 0.96 | 2.9 | 0.7 | 1.89 | 1.06 | 13.89 | 2.51 |
| 3.78 | 1.91 | 1.27 | 0.81 | 0.84 | 0.59 | 3.06 | 8.62 | 1.25 | 11.48 | 6.35 |
| 1.37 | 0.42 | 0.16 | 1.09 | 0.9 | 2.3 | 0.76 | 1.95 | 1.03 | 12.89 | 2.3 |
| 0.99 | 8.63 | 1.64 | 1.14 | 1.06 | 1.57 | 1.1 | 1.07 | 1.04 | 0.88 | 1.37 |
| 1.81 | 1.03 | 0 | 0.71 | 0.66 | 0.78 | 1.14 | 0.59 | 0.92 | 0.15 | 1.11 |
| 1.49 | Inf | Inf | 1.12 | 0.61 | 1.15 | 1.14 | 0.7 | 1.12 | 4.41 | 1.11 |
| 1.2 | 2.79 | 1.31 | 1.09 | 0.7 | 1.02 | 0.52 | 1.3 | 0.98 | 1.03 | 0.98 |
| 4.31 | 0.23 | 1.99 | 0.93 | 1.69 | 1.27 | 0.86 | 1.72 | 0.89 | 0.47 | 0.49 |
| 1.12 | 0.9 | 1 | 0.73 | 0.98 | 0.93 | 0.83 | 1.38 | 0.83 | 1.92 | 1.24 |
| 0.82 | 0.56 | 2.17 | 1.25 | 0.83 | 0.91 | 1.07 | 1.27 | 1.12 | 2.07 | 0.84 |
| 0.96 | 0.73 | 4.32 | 1.07 | 0.94 | 1.72 | 0.28 | 1.09 | 0.99 | 2.05 | 1.32 |
| 1.05 | 48.04 | 1.09 | 0.84 | 0.79 | 0.82 | 16 | 0.91 | 1.12 | 2.63 | 0.99 |
| 0.68 | 9.03 | 0.78 | 0.54 | 1.94 | 2.47 | 2.68 | 0.4 | 0.65 | 1.37 | 0.62 |
| 1.26 | 0.89 | 0.89 | 1.41 | 0.09 | 1.11 | 0.94 | 0.52 | 0.91 | 1.73 | 0.85 |
| 1.24 | 16.3 | 6.78 | 3.61 | 3.61 | 1.03 | 3.88 | 1.5 | 0.59 | 0.11 | 0.23 |
| 0.44 | 1.63 | 1.72 | 4.3 | 1 | 0.69 | 1.71 | 0.45 | 3.08 | 0.32 | 0.9 |
| 1.25 | 1.19 | 1 | 0.88 | 1.6 | 0.93 | 1.66 | 0.7 | 1.14 | 5.5 | 0.92 |
| 3.99 | 0.27 | 0.43 | 1.2 | 0.96 | 1.97 | 0.56 | 1.2 | 1.03 | 8.5 | 1.95 |
| 0.94 | 0.91 | 0.8 | 0.96 | 0.91 | 1.03 | 1.75 | 1.41 | 0.95 | 1.79 | 0.99 |
| 1.23 | 0.16 | 0.5 | 1.25 | 1.01 | 1.03 | 2.52 | 1.16 | 1.01 | 2.23 | 2.78 |
| 3.33 | 14 | 1.37 | 1.26 | 0.94 | 0.92 | 0.6 | 0.84 | 1.15 | 0.86 | 1.13 |

| | | | | | | | | | | |
|---|---|---|---|---|---|---|---|---|---|---|
| 0.37 | 3.28 | 4.27 | 1.09 | 1.33 | 1.03 | 1.73 | 1.72 | 0.95 | 3.71 | 1.04 |
| 3.69 | 3.76 | 2.95 | 3.91 | 7.55 | 1.22 | 5.65 | 1.72 | 1.03 | 61.5 | 3.69 |
| 1.44 | 1.01 | 0.89 | 2.8 | 0.23 | 1.62 | 1.71 | 0.72 | 1.18 | 3.45 | 0.95 |
| 6.01 | 5.85 | 8.05 | 1.05 | 2.77 | 1.12 | 1 | 2.43 | 0.95 | 5.89 | 2.15 |
| 0.81 | 1.35 | 1.58 | 1.54 | 1.07 | 2.55 | 0.68 | 1.01 | 0.87 | 1.1 | 1.06 |
| 3.17 | 1.34 | 3.96 | 0.82 | 1.29 | 1.13 | 2.52 | 2.37 | 0.97 | 1.06 | 13.66 |
| 0.86 | 0.07 | 3.55 | 1.32 | 0.96 | 1.05 | 0.9 | 2.49 | 1.53 | 5.04 | 0.91 |
| 0.82 | 0.96 | 1.15 | 0.98 | 0.76 | 0.61 | 14.72 | 1.73 | 2.43 | 0.69 | 0.7 |
| 0.59 | 3.34 | 2.26 | 0.77 | 0.97 | 1.87 | 1.65 | 1.9 | 0.67 | 2.42 | 1.83 |
| 1.53 | 1.07 | 0.28 | 0.62 | 1.42 | 1.73 | 0.55 | 0.72 | 1.85 | 0.85 | 2.26 |
| 7.59 | 2.14 | 6.74 | 0.95 | 8.66 | 0.43 | 2.34 | 0.34 | 3.01 | 21.26 | 2.54 |
| 0.72 | 0.24 | 3 | 0.46 | 1.05 | 1.01 | 1.01 | 0.62 | 0.77 | 1.73 | 1.29 |
| 0.86 | 2.05 | 5.5 | 0.45 | 1.14 | 1.04 | 0.52 | 0.95 | 1.12 | 1.04 | 0.88 |
| 1.26 | 1.25 | 1.58 | 1.51 | 1.81 | 0.9 | 1.35 | 0.59 | 0.69 | 1.78 | 8.99 |
| 1.45 | 1.3 | 0.46 | 1.06 | 0.61 | 0.59 | 0.78 | 1.55 | 0.81 | 0.58 | 0.95 |
| 0.68 | 0.32 | 0.59 | 0.12 | 0.04 | 1.27 | 0.79 | 0.59 | 0.21 | 0.06 | 0.04 |
| 1.42 | 5.21 | 3.16 | 1.07 | 1.26 | 0.78 | 3.63 | 1.38 | 0.92 | 0.8 | 0.73 |
| 0.68 | 0.65 | 1.71 | 0.15 | 0.38 | 1.29 | 0.74 | 0.44 | 0.64 | 0.87 | 3.04 |
| 1.59 | 1.04 | 1.18 | 2.15 | 1.09 | 1.23 | 1.19 | 1.25 | 1 | 0.9 | 0.8 |
| 4.05 | 0.93 | 0.79 | 1.13 | 1.45 | 0.81 | 2.27 | 1.44 | 1.69 | 12.6 | 4.25 |
| 0.42 | 15.3 | 0.79 | 3.67 | 2.32 | 0.91 | 2.91 | 1.52 | 1.22 | 0.59 | 0.55 |
| 0.77 | 3.37 | 0.92 | 2 | 3.91 | 1.14 | 4.09 | 3.02 | 1.71 | 1.97 | 0.83 |
| 0.98 | 1.49 | 3.91 | 0.74 | 0.68 | 1.44 | 0.89 | 3.89 | 0.85 | 0.43 | 0.74 |
| 1.37 | 0.93 | 0.44 | 0.67 | 1.13 | 0.84 | 1.11 | 0.96 | 1.09 | 0.96 | 0.79 |
| 1.11 | 0.91 | 2.56 | 2.4 | 2.2 | 0.76 | 2.01 | 0.57 | 1.34 | 2.34 | 0.85 |
| 1.62 | 0.74 | 3 | 0.86 | 1.38 | 0.97 | 1.03 | 1.28 | 0.79 | 1.35 | 0.78 |
| 1.05 | 1.42 | 0.93 | 0.63 | 0.77 | 0.92 | 1.34 | 4.91 | 0.99 | 1.08 | 0.89 |
| 1.61 | 0.98 | 1.07 | 1.67 | 2.55 | 1.06 | 7.42 | 1.27 | 1.03 | 0.4 | 0.88 |
| 0.84 | 0.53 | 7.24 | 0.79 | 0.83 | 1.11 | 0.54 | 1.17 | 0.58 | 1.67 | 0.94 |
| 3.18 | 0.63 | 0.91 | 1.04 | 0.86 | 0.95 | 1.52 | 0.8 | 1 | 5.36 | 0.97 |
| 1.3 | 1.3 | 0.69 | 0.73 | 0.96 | 1.06 | 3.59 | 1.19 | 1.25 | 6.77 | 0.87 |
| 0.34 | 0.74 | 0.5 | 1.05 | 1.08 | 1.03 | 0.65 | 1.16 | 1.09 | 1.13 | 0.22 |
| 0.6 | 1.08 | 0.75 | 1.26 | 3.16 | 1.48 | 3.17 | 1.38 | 1.19 | 9 | 1.15 |

| | | | | | | | | | | |
|---|---|---|---|---|---|---|---|---|---|---|
| 1.18 | 1.94 | 5.58 | 1.37 | 1.2 | 0.53 | 1.62 | 1.6 | 0.6 | 2.46 | 1.36 |
| 0.86 | 1.12 | 1.06 | 0.49 | 0.77 | 1.11 | 0.91 | 1 | 0.82 | 0.85 | 0.94 |
| 0.91 | 1.39 | 1.82 | 1.17 | 1.03 | 1.21 | 0.78 | 2.03 | 0.86 | 1.49 | 1.14 |
| 0.46 | 6.19 | 3.14 | 0.68 | 0.78 | 1.09 | 1.01 | 2.11 | 0.46 | 1.3 | 1.12 |
| 1.65 | 3.32 | 0.39 | 0.72 | 1.05 | 1.06 | 0.62 | 1.77 | 1.43 | 0.75 | 10.71 |
| 0.63 | 3.38 | 1.48 | 0.77 | 1.21 | 0.47 | 2.15 | 0.6 | 0.53 | 1.24 | 1.06 |
| 3.07 | 0.8 | 1.98 | 3.97 | 1.13 | 1.16 | 0.92 | 0.88 | 1.15 | 1.72 | 0.79 |
| 1.22 | 1.32 | 1.27 | 1.15 | 0.7 | 1.69 | 0.38 | 0.76 | 0.95 | 10.49 | 1.69 |
| 0.73 | 1.87 | 8.69 | 0.91 | 1.74 | 1.41 | 0.38 | 1.33 | 0.8 | 2.55 | 0.85 |
| 1.19 | 1.88 | 2.59 | 0.97 | 0.84 | 1.12 | 0.78 | 0.88 | 1.37 | 0.94 | 1.67 |
| 0.97 | 3.33 | 0.79 | 9 | 0.93 | 0.92 | 3 | 2.8 | 1.08 | 1.09 | 0.71 |
| 0.98 | 1.47 | 5.62 | 1.1 | 1.16 | 0.86 | 1.11 | 1.03 | 1.43 | 3.48 | 0.97 |
| 1.14 | 3.47 | 1.22 | 0.89 | 1.46 | 1.33 | 1.07 | 1.4 | 0.73 | 1.84 | 1.25 |
| 0.53 | 1.24 | 1.31 | 1.03 | 1.08 | 1.22 | 0.77 | 0.98 | 1.26 | 1.93 | 0.79 |
| 0.94 | 1.27 | 1.13 | 1 | 1.06 | 0.94 | 1.52 | 1 | 1.25 | 0.82 | 0.99 |
| 1.67 | 0.71 | 1.21 | 1.29 | 0.56 | 1.18 | 3 | 0.85 | 0.99 | 1.14 | 2.05 |
| 1.34 | 1.08 | 1.49 | 0.4 | 4.36 | 0.91 | 1.1 | 1.62 | 0.69 | 2.2 | 1.19 |
| 6.46 | 4.91 | 6.19 | 1.39 | 1.9 | 1.15 | 1.12 | 2.08 | 1.08 | 5.7 | 1.37 |
| 1.14 | 1.02 | 0.91 | 6.79 | 1.34 | 1.15 | 1.77 | 1.14 | 0.97 | 9.11 | 0.85 |
| 0.36 | 3.97 | 2.04 | 1.17 | 0.85 | 1.13 | 0.99 | 1.44 | 0.8 | 1 | 0.98 |
| 3.33 | 1.23 | 1.62 | 1.11 | 0.89 | 1.19 | 0.69 | 0.88 | 0.67 | 0.48 | 0.6 |
| 0.66 | 0.97 | 0.95 | 0.97 | 2.7 | 1.14 | 2.02 | 2.6 | 0.99 | 0.93 | 0.97 |
| 1.01 | 1.13 | 1.68 | 0.81 | 1.08 | 1.1 | 1.02 | 0.93 | 1.21 | 1 | 0.99 |
| 0.67 | 0.77 | 0.7 | 0.38 | 2.12 | 0.43 | 0.2 | 1.17 | 1.98 | 0.35 | 1.37 |
| 1.18 | 0.8 | 0.58 | 1.1 | 1.29 | 0.7 | 0.77 | 1.03 | 0.84 | 0.69 | 1 |
| 1.13 | 1.26 | 2.53 | 0.99 | 1.38 | 0.87 | 1.19 | 1.45 | 0.96 | 1.11 | 0.72 |
| 1.34 | 0.34 | 0.81 | 0.58 | 2.73 | 0.95 | 1.35 | 0.45 | 1.05 | 0.22 | 1.14 |
| 4.88 | 0.53 | 2.49 | 1.96 | 0.91 | 0.67 | 2.77 | 0.9 | 1.52 | 5.42 | 1.83 |
| 0.45 | 1.14 | 1.59 | 0.7 | 0.98 | 1.05 | 0.95 | 0.83 | 0.62 | 0.3 | 0.32 |
| 1.6 | 1 | 1.17 | 1.17 | 0.94 | 1.01 | 0.64 | 1.19 | 0.9 | 4.36 | 1.1 |
| 0.9 | 0.91 | 1.13 | 1.39 | 0.81 | 1.05 | 1.39 | 1.91 | 0.71 | 0.3 | 1.01 |
| 3.69 | 3.76 | 2.95 | 3.91 | 7.55 | 1.22 | 5.65 | 1.72 | 1.03 | 61.5 | 3.69 |
| 0.64 | 0.95 | 0.92 | 1.15 | 0.83 | 1.21 | 0.56 | 1.04 | 0.96 | 0.15 | 0.81 |

| | | | | | | | | | | |
|---|---|---|---|---|---|---|---|---|---|---|
| 1.52 | 2.72 | 0.78 | 0.67 | 1.79 | 0.84 | 1.14 | 0.68 | 0.99 | 0.03 | 3.56 |
| 1.34 | 1.58 | 8.15 | 2.22 | 1.69 | 0.83 | 0.95 | 1.36 | 1.05 | 1.07 | 1.86 |
| 1.12 | 0.57 | 23 | 1.67 | 2.05 | 1.06 | 0.46 | 1.69 | 0.95 | 0.87 | 1.05 |
| 2.61 | 0.72 | 0.62 | 0.94 | 1.46 | 1.02 | 1.63 | 1.14 | 1.04 | 3.25 | 0.98 |
| 0.82 | 4.24 | Inf | 3.98 | 2.96 | 1.26 | 3.83 | 1.12 | 0.91 | 1.23 | 362.78 |
| 0.16 | 1.5 | 1 | 0.12 | 1.94 | 1.01 | 0.87 | 0.71 | 0.78 | 1.71 | 1.27 |
| 1.01 | 2.92 | 0.83 | 1.1 | 0.58 | 0.97 | 1.95 | 1.89 | 0.84 | 1.4 | 0.89 |
| 1.9 | 1.17 | 1.26 | 2.13 | 1.11 | 1.14 | 1.88 | 0.6 | 1.41 | 0.64 | 1.15 |
| 0.13 | 0.94 | 7.62 | 1.56 | 0.92 | 0.65 | 0.04 | 0.58 | 1.18 | 0.02 | 0.98 |
| 1.97 | 1.21 | 0.81 | 1.23 | 0.61 | 1.24 | 0.49 | 1.32 | 1.06 | 4.93 | 1.02 |
| 0.43 | 1.06 | 1.53 | 0.38 | 3.17 | 1.85 | 0.82 | 1.13 | 0.68 | 0.84 | 1.16 |
| 2.67 | 3.14 | 3.04 | 0.69 | 0.83 | 1.46 | 0.7 | 0.69 | 3.09 | 1.08 | 1 |
| 3.82 | 6.4 | 7.88 | 1.2 | 1.4 | 1.11 | 0.92 | 2.58 | 1.38 | 4.33 | 1.6 |
| 4.77 | 0.65 | 0.2 | 1.03 | 1.66 | 0.88 | 0.95 | 0.69 | 0.84 | 4.44 | 0.95 |
| 3.01 | 0.78 | 1.41 | 0.53 | 0.49 | 2.1 | 3.1 | 0.54 | 1.38 | 2.54 | 2.16 |
| 0.85 | 1.02 | 1.16 | 1.24 | 0.89 | 1.27 | 2.41 | 0.65 | 1.11 | 1.92 | 1.19 |
| 1.26 | 2.85 | 3.33 | 1.16 | 1.07 | 0.94 | 2.18 | 0.78 | 0.99 | 3.81 | 0.98 |
| 0.76 | 0.82 | 0.52 | 1.02 | 0.74 | 1.32 | 1.65 | 1.23 | 1.08 | 1.13 | 0.8 |
| 0.49 | 1.76 | 1.47 | 1.22 | 1.2 | 0.91 | 0.44 | 0.71 | 0.93 | 1.52 | 0.92 |
| 0.5 | 1.08 | 1.37 | 1.34 | 1.99 | 1.01 | 1.1 | 0.73 | 0.81 | 1.54 | 0.58 |
| 1.01 | 23.83 | 1.11 | 1.49 | 1.09 | 0.95 | 0.75 | 1.37 | 1.04 | 0.98 | 0.8 |
| 2.1 | 0.71 | 0.54 | 1.07 | 0.67 | 3.15 | 0.98 | 1.42 | 1.8 | 0.46 | 0.62 |
| 0.84 | 0.95 | 0.88 | 1.01 | 1.24 | 1.09 | 1.15 | 0.92 | 1.34 | 2.88 | 1.77 |
| 1.62 | 0.83 | 1.74 | 2.08 | 0.31 | 0.64 | 1.83 | 2.89 | 0.84 | 0.32 | 1.33 |
| 1.24 | 0.22 | 0.93 | 1.3 | 2.45 | 1.4 | 1.08 | 4 | 1.01 | 1.1 | 0.93 |
| 1.25 | 3.31 | Inf | 1.26 | 1.17 | 0.96 | 1.4 | 2.84 | 1.1 | 2.36 | 0.56 |
| 0.55 | 1.25 | 0.5 | 0.99 | 1.05 | 1.05 | 1.34 | 0.88 | 1.37 | 0.36 | 1.1 |
| 0.92 | 0.56 | 4.33 | 1.02 | 0.72 | 1.17 | 0.59 | 1.31 | 0.75 | 0.85 | 1.21 |
| 1.46 | 3.63 | 1.11 | 1.39 | 1.49 | 1.09 | 1.2 | 1.36 | 1.07 | 3.03 | 1.11 |
| 0.27 | 0.48 | 0.83 | 4.67 | 0.92 | 1.07 | 2.35 | 1.26 | 1.21 | 0.13 | 0.52 |
| 0.92 | 1.03 | 0.65 | 0.75 | 1.36 | 1.07 | 1.06 | 0.58 | 0.93 | 1.34 | 1.19 |
| 0.91 | 6.48 | 3.38 | 1.01 | 1.11 | 0.98 | 2.3 | 4.42 | 0.92 | 0.94 | 0.92 |
| 0.33 | 1.02 | 0.85 | 1.26 | 1.74 | 1 | 0.29 | 3.06 | 0.99 | 1 | 1.02 |

| | | | | | | | | | | |
|---|---|---|---|---|---|---|---|---|---|---|
| 0.73 | 5.12 | 1.34 | 0.68 | 0.92 | 2.03 | 1.62 | 1.6 | 0.65 | 0.53 | 1.91 |
| 1.15 | 1.04 | 0.7 | 0.77 | 0.85 | 1.39 | 0.58 | 1.33 | 1.04 | 0.85 | 1.17 |
| 0.67 | 2.33 | 1.13 | 1.23 | 0.92 | 0.96 | 0.9 | 1.29 | 1.07 | 2.03 | 0.98 |
| 1.63 | 0.21 | 2.6 | 0.88 | 3.46 | 1.16 | 2.23 | 1.01 | 1.02 | 1.14 | 0.7 |
| 0.5 | 1.25 | 9.92 | 1.97 | 0.73 | 1.9 | 1.29 | 4.48 | 0.73 | 0.15 | 1.65 |
| 1.11 | 1 | 0.96 | 0.99 | 0.83 | 1.07 | 1.44 | 1.01 | 0.93 | 1.57 | 1.26 |
| 2.83 | 0.78 | 0.58 | 0.46 | 0.68 | 1.19 | 3.15 | 2.35 | 0.66 | 2.64 | 0.26 |
| 0.71 | 0.5 | 0.82 | 0.61 | 3.74 | 0.9 | 1.86 | 0.93 | 0.92 | 2.68 | 1 |
| 3.81 | 3.77 | 1.12 | 1.05 | 0.83 | 1.23 | 1.54 | 0.94 | 0.9 | 3.42 | 1.18 |
| 1.73 | 0.73 | 0.51 | 0.39 | 1.52 | 1.09 | 4.72 | 2.06 | 0.58 | 1.15 | 0.32 |
| 0.39 | 1.57 | 2.38 | 2 | 10 | 0.95 | 1.06 | 0.68 | 1.04 | 0.19 | 0.32 |
| 1.23 | 1.09 | 0.86 | 0.71 | 1.25 | 1.62 | 1.73 | 0.45 | 1.05 | 0.77 | 1.69 |
| 0.98 | 0.63 | 3.19 | 1.02 | 1.85 | 1.2 | 0.53 | 1.08 | 0.72 | 1.16 | 0.77 |
| 0.5 | 1.03 | 1.09 | 0.76 | 0.88 | 0.94 | 1.41 | 1.04 | 1.15 | 3.36 | 1.07 |
| 1.74 | 0.72 | 0.67 | 1.04 | 1.23 | 0.97 | 1.46 | 1.29 | 1.11 | 5.94 | 0.75 |
| 0.96 | 2.74 | 1.23 | 0.53 | 1.08 | 1.14 | 0.62 | 1.42 | 1.37 | 0.79 | 1.16 |
| 0.75 | 0.98 | 0.73 | 1.19 | 1.04 | 1.06 | 2.27 | 0.64 | 1.06 | 1.95 | 1.13 |
| 1.32 | 0.98 | 0.23 | 1.85 | 1.23 | 1.01 | 0.77 | 1.09 | 0.9 | 2.3 | 1.05 |
| 0.37 | 2.16 | 2.23 | 0.71 | 0.81 | 0.7 | 1.05 | 0.6 | 0.61 | 2.18 | 1.21 |
| 1.1 | 0.62 | 0.5 | 0.7 | 0.81 | 1.04 | 1.58 | 1.02 | 0.83 | 1.38 | 0.73 |
| 2.27 | 1.47 | 0.41 | 1.06 | 1.37 | 1.09 | 0.66 | 1.06 | 1.25 | 2 | 1.14 |
| 1.47 | 0.02 | 3.62 | 2.64 | 0.82 | 0.8 | 6.62 | 2.17 | 4.17 | 0.12 | 1.82 |
| 1.15 | 1.02 | 1.14 | 1.64 | 1.1 | 0.97 | 0.73 | 1.26 | 1.13 | 2.66 | 1.02 |
| 0.67 | 2.63 | 1.3 | 0.69 | 1.2 | 2.44 | 0.88 | 2.41 | 0.52 | 2.08 | 1.69 |
| 0.97 | 0.74 | NA | 1.03 | 0.84 | 0.87 | 0.74 | 0.94 | 1.18 | 3.44 | 0.86 |
| 0.78 | 853.2 | 0.62 | 2.47 | 1.09 | 1.11 | 20.89 | 0.9 | 0.99 | 0.66 | 2.35 |
| 0.89 | 0.23 | 5.5 | 0.65 | 1.11 | 1.21 | 0.57 | 1.34 | 1.06 | 0.84 | 0.85 |
| 0.67 | 1.05 | 0.88 | 1.26 | 2.88 | 0.96 | 2.75 | 0.7 | 0.93 | 0.5 | 1.12 |
| 1.23 | 1.03 | 0.95 | 1.05 | 0.74 | 1.07 | 1.02 | 1.1 | 1.03 | 3.3 | 1.25 |
| 0.92 | 1.1 | 1.14 | 0.7 | 4.94 | 1.09 | 1.21 | 2.87 | 0.67 | 2.25 | 1.18 |
| 0.82 | 0.85 | 9.02 | 1.04 | 1.03 | 0.64 | 1.33 | 6.31 | 1.18 | 2.46 | 0.74 |
| 0.73 | 1.15 | 2 | 0.74 | 0.83 | 1.06 | 0.88 | 0.97 | 0.83 | 1.36 | 0.83 |
| 1.91 | 1.84 | 2.81 | 5.18 | 0.75 | 1.4 | 0.84 | 2.32 | 1.44 | 0.43 | 0.25 |

| | | | | | | | | | | |
|---|---|---|---|---|---|---|---|---|---|---|
| 0.94 | 0.75 | 0.66 | 1.99 | 0.77 | 1.09 | 2.88 | 0.53 | 2.36 | 1.18 | 0.92 |
| 1.55 | 2 | 0.08 | 0.85 | 1.26 | 1.33 | 0.27 | 4.88 | 0.97 | 0.04 | 1.5 |
| 29.3 | 0.75 | 2.3 | 0.99 | 0.54 | 1.36 | 1.17 | 1.28 | 1.07 | 1.54 | 0.7 |
| 1.53 | 3.96 | 1.32 | 0.62 | 0.57 | 1.13 | 0.14 | 0.64 | 0.65 | 0.25 | 0.99 |
| 0.68 | 0.89 | 0.64 | 0.9 | 1.26 | 1.14 | 1.72 | 2.2 | 1.04 | 2.36 | 0.6 |
| 0.85 | 1.81 | 1.75 | 1.03 | 0.81 | 1.07 | 1.05 | 2.48 | 0.92 | 0.56 | 1.1 |
| 0.6 | 0.97 | 0.91 | 1 | 0.77 | 0.92 | 0.97 | 0.75 | 1.09 | 0.79 | 0.64 |
| 0.32 | 0.98 | 0.95 | 0.85 | 2.48 | 1.26 | 1.54 | 1.94 | 1.19 | 0.51 | 0.89 |
| 0.79 | 5.05 | 0.46 | 0.57 | 1.19 | 1.55 | 1.42 | 0.53 | 0.45 | 0.95 | 1.99 |
| 4.03 | 1.76 | 5.02 | 1.5 | 1.5 | 1.05 | 1.43 | 1.49 | 0.98 | 2.53 | 0.27 |
| 0.67 | 1.5 | 3 | 1.2 | 1.22 | 0.95 | 1.06 | 1.81 | 0.81 | 0.92 | 1.06 |
| 0.95 | 0.12 | 1 | 0.98 | 1.46 | 1.08 | 0.97 | 1 | 1.08 | 0.82 | 1.25 |
| 0.36 | 0.8 | 1 | 0.16 | 0.59 | 0.93 | 0.4 | 1.21 | 0.81 | 0.25 | 0.95 |
| 0.59 | 0.94 | 1.06 | 0.88 | 0.24 | 1.14 | 2.54 | 3.17 | 0.97 | 0.15 | 0.99 |
| 0.78 | 1.01 | 0.76 | 1.89 | 0.27 | 0.99 | 0.24 | 0.77 | 1.27 | 1.53 | 1.23 |
| 1.54 | 1.26 | 0.28 | 0.81 | 0.48 | 1.02 | 2.96 | 0.97 | 0.95 | 1.42 | 2.06 |
| 0.8 | 0.81 | 1.88 | 0.27 | 1.15 | 1.19 | 0.85 | 1.47 | 0.81 | 0.52 | 0.87 |
| 0.87 | 1.06 | 0.96 | 2.17 | 2.07 | 1.03 | 11.71 | 4.09 | 0.93 | 2.78 | 0.81 |
| 0.51 | 5 | 0.35 | 1.1 | 1.05 | 0.67 | 2.85 | 27.86 | 0.52 | 1.63 | 1.13 |
| 1.1 | 0.62 | 7.67 | 0.32 | 1.58 | 0.29 | 0.58 | 0.52 | 0.82 | 0.21 | 0.69 |
| 0.85 | 3.18 | 0.38 | 0.91 | 3.47 | 0.5 | 0.63 | 1.57 | 2.32 | 2 | 0.89 |
| 1.87 | 0.77 | 0.55 | 0.44 | 1.26 | 1.14 | 2.9 | 2.03 | 0.59 | 2.37 | 0.33 |
| 0.79 | 7.54 | 4.12 | 0.63 | 0.67 | 1.13 | 0.6 | 9.67 | 0.56 | 10.63 | 1.31 |
| 0.12 | 0.68 | 0.13 | 1.08 | 1.03 | 0.39 | 0.61 | 1.5 | 0.75 | 1.43 | 0.73 |
| 0.86 | 0.96 | 1.03 | 0.92 | 1.62 | 1 | 1.19 | 0.72 | 0.97 | 0.89 | 0.92 |
| 1.25 | 0.86 | 31.81 | 1.37 | 0.88 | 1.21 | 1.21 | 1.25 | 1.19 | 1 | 0.8 |
| 1.24 | 0.99 | 1.25 | 0.66 | 1.23 | 1.12 | 1.55 | 0.72 | 0.8 | 0.65 | 2.39 |
| 0.19 | 2.11 | 1.58 | 0.31 | 0.45 | 0.67 | 0.54 | 0.72 | 1.27 | 1.88 | 0.76 |
| 5.07 | 8.32 | 2.44 | 1.64 | 1.02 | 1.16 | 0.79 | 1.53 | 0.52 | 2.49 | 1.49 |
| 9.43 | 0.3 | 3 | 5.27 | 1.64 | 3.55 | 0.79 | 16 | 0.11 | 0.44 | 0.49 |
| 1.27 | 1.04 | 1.16 | 3 | 1.05 | 0.69 | 0.75 | 1.06 | 1.24 | 0.81 | 1.12 |
| 0.83 | 0.89 | 0.88 | 1.37 | 0.84 | 1.04 | 4.06 | 1.31 | 0.75 | 1.53 | 0.64 |
| 0.98 | 0.52 | 0.47 | 3.45 | 0.94 | 1.29 | 1.17 | 1.88 | 1.32 | 0.59 | 0.61 |

| | | | | | | | | | | |
|---|---|---|---|---|---|---|---|---|---|---|
| 0.62 | 0.6 | 0.72 | 0.95 | 0.9 | 0.79 | 0.54 | 0.86 | 1.18 | 11.5 | 1 |
| 0.89 | 1.08 | 3.28 | 0.74 | 1.19 | 0.9 | 1.13 | 1.02 | 1.15 | 1.11 | 1.64 |
| 0.44 | 0.11 | 0.5 | 0.6 | 1.01 | 14.67 | 0.96 | 1.61 | 0.74 | 0.4 | 1.77 |
| 0.09 | 0.22 | 0.12 | 1.31 | 0.7 | 0.71 | 2.64 | 2.95 | 0.94 | 1.05 | 1.6 |
| 2.14 | 0.22 | 1.95 | 2.97 | 1.06 | 1.21 | 1.53 | 0.52 | 0.84 | 1.98 | 0.13 |
| 0.7 | 0.85 | 1.36 | 1.49 | 0.7 | 1.01 | 1.17 | 0.84 | 0.99 | 1.78 | 0.59 |
| 1.07 | 1.86 | 3.04 | 1.1 | 1.09 | 1.09 | 1.33 | 0.94 | 0.87 | 1.41 | 0.98 |
| 1.32 | 1.45 | 0.39 | 1.11 | 1.47 | 0.94 | 1.03 | 1.16 | 1.04 | 6.72 | 0.65 |
| 1.13 | 0.51 | 1.5 | 0.97 | 0.88 | 1.27 | 1.85 | 1.08 | 1.12 | 1.42 | 0.89 |
| 1.82 | 1.3 | 2.32 | 1.15 | 1.25 | 1.06 | 3.85 | 2.37 | 0.89 | 0.58 | 1.16 |
| 3.87 | 0.72 | 6.81 | 1.72 | 0.88 | 1.24 | 0.64 | 0.67 | 1.03 | 1.46 | 0.72 |
| 2.75 | 1.01 | 0.19 | 0.1 | 0.31 | 23.1 | 0.57 | 0.39 | 0.3 | 1.02 | 6.25 |
| 1.28 | 1.61 | 1.34 | 0.99 | 0.69 | 0.94 | 1.42 | 0.86 | 1.42 | 1.21 | 1.47 |
| 1 | 0 | 0 | 4.4 | 0.86 | 1.09 | 1.82 | 0.45 | 1.67 | 130 | 1.49 |

| 204798_at | 202326_at | 220200_s_at | 207545_s_at | 211012_s_at | 220104_at | 221645_s_at | 205289_at | 207347_at | 204086_at | 213720_s_at |
|---|---|---|---|---|---|---|---|---|---|---|
| 1.93 | 1.03 | 0.99 | 0.95 | 1.39 | 1.65 | 0.92 | 2.27 | 1.13 | 1.16 | 0.97 |
| 1.54 | 1.05 | 1.14 | 1.8 | 0.76 | 1.18 | 1.55 | 1.57 | 1.06 | 1.25 | 1.06 |
| 1.06 | 1.23 | 0.81 | 1.16 | 0.65 | 1.34 | 3.21 | 3.6 | 1.52 | 1.27 | 0.84 |
| 1.04 | 1.18 | 0.97 | 1.59 | 1.88 | 1.21 | 1.42 | 4.64 | 1.29 | 2.22 | 1.01 |
| 1.16 | 0.83 | 1.06 | 1.09 | 1.41 | 1.75 | 0.93 | 0.67 | 1.09 | 1.84 | 0.88 |
| 0.7 | 1.59 | 3.27 | 1.21 | 0.81 | 1.12 | 0.87 | 3.04 | 1.93 | 1.95 | 1 |
| 0.07 | 4 | 1.43 | 1.17 | 1.25 | 0.47 | 0.67 | 4.2 | 1.91 | 0.72 | 0.96 |
| 0.9 | 0.92 | 0.99 | 0.63 | 0.99 | 1.09 | 1.19 | 0.97 | 1.07 | 0.88 | 1.12 |
| 0.6 | 0.74 | 0.92 | 1.04 | 1.09 | 1.24 | 0.68 | 0.98 | 1.05 | 1.56 | 0.83 |
| 0.27 | 0.45 | 1.76 | 1.13 | 7.93 | 36.31 | 0.81 | 7.91 | 0.76 | 0.62 | 0.69 |
| 1.17 | 1.01 | 1.07 | 1 | 0.45 | 1.33 | 1.16 | 1.41 | 1.01 | 2.69 | 1.18 |
| 1.25 | 1.14 | 1.27 | 0.95 | 0.68 | 0.78 | 0.79 | 1.28 | 0.92 | 1.09 | 1.02 |
| 0.85 | 0.88 | 1.19 | 1.08 | 0.93 | 1.04 | 0.85 | 1.52 | 0.9 | 2.16 | 0.98 |
| 2.15 | 0.11 | 1.98 | 0.16 | 0.35 | 1.2 | 0.04 | 581.77 | 1.05 | 0.17 | 0.24 |
| 0.85 | 1.98 | 2.01 | 1.15 | 0.66 | 0.26 | 2.13 | 1.45 | 1.27 | 4.17 | 0.84 |
| 1.76 | 0.99 | 0.96 | 1.26 | 0.68 | 1.33 | 1.32 | 0.86 | 1.06 | 1.29 | 1 |
| 2.98 | 1.32 | 1 | 1.33 | 1.9 | 0.62 | 1.18 | 1.35 | 0.86 | 3.58 | 1.53 |
| 4.28 | 1.29 | 1.3 | 1.06 | 1.29 | 1.25 | 1.04 | 1.39 | 1.11 | 1.51 | 1.28 |
| 0.53 | 1.97 | 0.74 | 1.38 | 0.15 | 1.69 | 1.22 | 0.8 | 1.25 | 0.78 | 0.78 |
| 1.27 | 0.26 | 1.08 | 2.17 | 3.92 | 1.31 | 1.53 | 8.54 | 1.29 | 1.44 | 1.12 |
| 1.26 | 7.62 | 0.28 | 0.4 | 2.3 | 1.59 | 0.53 | 2.35 | 1.06 | 2.58 | 1.22 |
| 2.98 | 1.77 | 0.95 | 0.58 | 3.24 | 2.62 | 0.91 | 0.78 | 2.61 | 1.09 | 1.53 |
| 1.57 | 1.14 | 0.52 | 0.74 | 0.94 | 0.79 | 0.95 | 0.84 | 0.9 | 11.95 | 0.94 |
| 1.07 | 1.42 | 0.95 | 1.97 | 1.57 | 1.1 | 0.81 | 3.85 | 2.29 | 1.23 | 0.88 |
| 0.04 | 1.25 | 0.47 | 1.15 | 7.42 | 1.06 | 0.06 | 0.06 | 1.21 | 16.08 | 0.04 |
| 0.44 | 1.06 | 1 | 1.39 | 0.95 | 0.56 | 1.37 | 0.9 | 1.52 | 1.45 | 0.81 |
| 10.86 | 1.05 | 1.04 | 0.39 | 1.22 | 1.78 | 1.34 | 3.94 | 1.05 | 1.18 | 0.74 |
| 1.54 | 1.25 | 1.01 | 1.18 | 24.79 | 1.24 | 1 | 5.82 | 2.03 | 0.61 | 0.75 |
| 3.77 | 0.82 | 1.15 | 0.77 | 1.81 | 1.3 | 0.81 | 2.36 | 1.5 | 0.39 | 1.51 |
| 0.91 | 0.79 | 1.43 | 0.98 | 1.18 | 1.35 | 1.38 | 1.91 | 0.91 | 1.24 | 0.91 |

| | | | | | | | | | | |
|---|---|---|---|---|---|---|---|---|---|---|
| 0.75 | 0.71 | 1.33 | 0.71 | 1.05 | 1.2 | 0.76 | 2.96 | 0.66 | 1.42 | 1.04 |
| 1.25 | 0.85 | 1.08 | 1.15 | 0.96 | 3.97 | 0.79 | 926.27 | 0.9 | 2.93 | 5.81 |
| 0.95 | 0.96 | 0.85 | 0.99 | 2 | 1.06 | 1.12 | 0.89 | 1.2 | 1.5 | 1.1 |
| 6.78 | 0.73 | 1.73 | 0.6 | 0.27 | 1.55 | 0.64 | 0.39 | 0.91 | 0.96 | 0.77 |
| 0.86 | 1.04 | 1.63 | 3.2 | 1.67 | 0.98 | 4.78 | 0.62 | 1.21 | 1.58 | 0.2 |
| 1.55 | 1.18 | 0.27 | 1.23 | 0.44 | 4.42 | 1.1 | 23.53 | 1.14 | 2 | 1.06 |
| 1.17 | 1.72 | 0.54 | 0.46 | 0.8 | 1.73 | 0.31 | 30.85 | 0.93 | 27.49 | 1.41 |
| 0.8 | 1.17 | 0.99 | 0.83 | 0.5 | 2.32 | 0.76 | 0.98 | 1.22 | 0.58 | 0.88 |
| 0.37 | 1 | 0.67 | 0.93 | 1.65 | 0.66 | 0.19 | 0.6 | 0.57 | 1.47 | 0.85 |
| 0.03 | 0.86 | 0.72 | 1.25 | 3.12 | 0.91 | 1.7 | 5.44 | 2.9 | 4.43 | 0.95 |
| 1.11 | 1.22 | 0.98 | 0.96 | 1.44 | 0.87 | 1.65 | 0.8 | 0.8 | 1.71 | 1.07 |
| 0.99 | 1.14 | 0.91 | 0.87 | 0.98 | 0.96 | 0.94 | 0.47 | 1.18 | 1.38 | 1.13 |
| 1.06 | 0.35 | 0.57 | 4.99 | 0.69 | 1.37 | 4.87 | 3.23 | 1.35 | 0.18 | 0.9 |
| 1.51 | 0.23 | 1.24 | 1.75 | 0.75 | 0.62 | 1.19 | 3.66 | 1 | 2.18 | 0.53 |
| 0.92 | 0.5 | 0.62 | 3.31 | 0.67 | 1.45 | 3.01 | 2.07 | 1.32 | 0.13 | 0.93 |
| 1.35 | 0.94 | 0.76 | 1.58 | 1.8 | 1.08 | 2.03 | 2.13 | 1.24 | 0.95 | 0.81 |
| 1.04 | 1.27 | 0.98 | 1.28 | 0.59 | 0.42 | 1.12 | 1.13 | 1.03 | 1.35 | 0.99 |
| 1.25 | 1.93 | 1.97 | 1.09 | 2.39 | 0.87 | 1.35 | 1.12 | 0.82 | 0.88 | 0.88 |
| 0.85 | 0.88 | 1.06 | 1.04 | 0.78 | 0.91 | 1.08 | 0.98 | 1.02 | 0.91 | 0.97 |
| 2.38 | 0.73 | 1.21 | 2.14 | 1.19 | 1.46 | 1.18 | 2.19 | 0.95 | 1.26 | 1.53 |
| 0.86 | 0.91 | 0.65 | 0.92 | 0.68 | 0.86 | 1.11 | 0.75 | 0.73 | 1.14 | 0.84 |
| 1.71 | 1.13 | 1.06 | 0.75 | 2.01 | 0.9 | 0.86 | 1.18 | 1.06 | 2.7 | 1.02 |
| 1.15 | 1.13 | 1.08 | 1.07 | 0.53 | 0.46 | 0.89 | 1.29 | 1.17 | 2.55 | 1 |
| 1.05 | 1.35 | 1.68 | 1.05 | 0.93 | 1.09 | 1.47 | 0.71 | 1.05 | 1.72 | 1.15 |
| 0.79 | 0.53 | 0.65 | 1.93 | 1.24 | 0.86 | 0.69 | 5.35 | 1.81 | 2 | 1.05 |
| 23.86 | 1.03 | 1.41 | 0.58 | 1.14 | 4.15 | 1 | 1.34 | 1.1 | 2.03 | 0.86 |
| 0.68 | 0.98 | 1.28 | 2.69 | 1.53 | 1.52 | 0.57 | 43.86 | 0.8 | 0.43 | 1.24 |
| 1.75 | 1.33 | 1.03 | 0.85 | 1.63 | 1.62 | 35.53 | 0.38 | 1.88 | 1.03 | 1.18 |
| 0.54 | 0.86 | 0.79 | 1.08 | 0.74 | 1.14 | 2 | 0.93 | 0.99 | 4.5 | 1.2 |
| 1.23 | 0.33 | 0.49 | 4.6 | 0.8 | 2.04 | 2.74 | 1.63 | 1.09 | 0.17 | 1.06 |
| 0.8 | 0.72 | 1.5 | 1.07 | 4.06 | 1.41 | 1 | 8.1 | 1.03 | 0.99 | 1.1 |
| 1.05 | 0.88 | 0.88 | 0.91 | 1.49 | 2.23 | 1.22 | 0.35 | 1.67 | 0.68 | 1.28 |
| 0.6 | 1.01 | 1.04 | 0.94 | 3.67 | 1.48 | 0.94 | 0.61 | 1.22 | 4.51 | 0.95 |

| | | | | | | | | | | |
|---|---|---|---|---|---|---|---|---|---|---|
| 2 | 0.73 | 0.98 | 1.2 | 0.84 | 1.34 | 1.22 | 1.14 | 0.99 | 0.31 | 1.01 |
| 2.41 | 1.16 | 1.33 | 0.98 | 1.38 | 2.47 | 1.37 | 1.71 | 1.31 | 1.41 | 1.31 |
| 0.96 | 1.26 | 1.14 | 1.02 | 0.97 | 0.7 | 1.36 | 1.05 | 1.02 | 1.5 | 0.95 |
| 1.64 | 1.27 | 1.03 | 1.34 | 2.25 | 0.96 | 2.46 | 2.15 | 1.13 | 4.05 | 2.17 |
| 0.88 | 0.69 | 1.13 | 1.11 | 1 | 0.8 | 1.35 | 0.9 | 1.07 | 1.05 | 0.85 |
| 0.99 | 1.3 | 1.4 | 1.7 | 0.59 | 0.8 | 0.55 | 2.06 | 0.92 | 0.79 | 1.21 |
| 0.43 | 1.82 | 1.75 | 0.95 | 0.87 | 1.53 | 2.21 | 1.71 | 1.3 | 0.92 | 0.84 |
| 0.72 | 1.96 | 1.64 | 0.9 | 1.47 | 0.87 | 0.92 | 0.66 | 1.17 | 2.12 | 0.91 |
| 0.73 | 1.37 | 1.61 | 1.05 | 1.17 | 0.39 | 0.82 | 1.09 | 0.54 | 1.46 | 0.75 |
| 1.46 | 0.62 | 0.57 | 1.37 | 1.37 | 0.6 | 1.46 | 1.92 | 0.88 | 1.18 | 1.91 |
| 2.23 | 1.36 | 3.66 | 0.91 | 0.75 | 1.34 | 1.14 | 7.31 | 1.38 | 2.99 | 0.95 |
| 1.47 | 2.18 | 1.18 | 1.1 | 1.77 | 1.45 | 1.05 | 1 | 1.47 | 0.92 | 0.81 |
| 0.83 | 0.61 | 2.16 | 0.98 | 1.26 | 2.58 | 1.39 | 3 | 0.98 | 0.98 | 0.98 |
| 1.41 | 1.41 | 1.39 | 2.07 | 1.43 | 1.14 | 1.16 | 16.57 | 0.89 | 2.1 | 1.16 |
| 1.29 | 1.38 | 0.87 | 0.75 | 2.02 | 1.06 | 0.67 | 1.47 | 0.93 | 0.79 | 1.24 |
| 0.14 | 0.09 | 0.2 | 1.17 | 4.34 | 2 | 39.78 | 3.38 | 0.51 | 0.88 | 15.25 |
| 0.33 | 0.74 | 1.06 | 1.33 | 0.38 | 0.76 | 1.93 | 1.04 | 1.03 | 1.65 | 0.73 |
| 2.86 | 1.02 | 0.34 | 0.88 | 0.62 | 2.17 | 1.1 | 1.93 | 1.17 | 0.67 | 1.58 |
| 0.85 | 0.84 | 1.02 | 1.89 | 1.49 | 1.49 | 1.21 | 1.31 | 1.86 | 1.39 | 0.93 |
| 0.8 | 5 | 0.8 | 1.16 | 6.78 | 0.89 | 0.76 | 3.88 | 1.78 | 1.8 | 1.02 |
| 0.73 | 0.45 | 0.51 | 1.96 | 6.47 | 5.65 | 0.69 | 4.09 | 1.11 | 0.45 | 1.75 |
| 1.35 | 1.02 | 1 | 0.72 | 1.18 | 1.07 | 1.32 | 0.72 | 0.88 | 3.44 | 1.16 |
| 0.21 | 5.38 | 1.06 | 1.12 | 0.98 | 0.51 | 0.86 | 5.77 | 1.66 | 0.47 | 0.89 |
| 39.08 | 2.27 | 2.37 | 0.69 | 0.64 | 1.5 | 0.75 | 2.49 | 1.17 | 1.83 | 1.14 |
| 0.87 | 0.95 | 0.98 | 1.06 | 0.95 | 1.81 | 1.31 | 1.69 | 1.02 | 2.31 | 0.87 |
| 0.65 | 0.76 | 1.16 | 0.92 | 0.81 | 0.77 | NA | 0.98 | 0.79 | 0.9 | 0.77 |
| 0.9 | 1.37 | 0.94 | 0.78 | 1.37 | 1.15 | 0.79 | 0.89 | 0.81 | 1.05 | 1.16 |
| 0.95 | 0.93 | 0.97 | 1.12 | 0.82 | 1.19 | 1.1 | 0.49 | 0.98 | 1.43 | 1.05 |
| 0.91 | 0.83 | 0.93 | 1.29 | 1.85 | 2.06 | 1.2 | 2.35 | 0.94 | 1.25 | 0.94 |
| 1.2 | 1.06 | 0.94 | 0.94 | 1.36 | 0.9 | 0.97 | 6.95 | 0.78 | 0.91 | 0.96 |
| 0.92 | 1.06 | 1.08 | 1.23 | 1.17 | 1.12 | 0.96 | 1.52 | 0.75 | 1.03 | 0.99 |
| 0.78 | 0.62 | 0.76 | 0.74 | 0.97 | 0.91 | 0.67 | 1.73 | 1.24 | 0.8 | 1.06 |
| 0.95 | 0.73 | 0.99 | 1.64 | 1.22 | 1.86 | 1.37 | 1.67 | 1.54 | 6.19 | 0.91 |

| | | | | | | | | | | |
|---|---|---|---|---|---|---|---|---|---|---|
| 1.19 | 1.53 | 1.09 | 0.71 | 1.72 | 0.29 | 1.03 | 2.06 | 0.93 | 1.31 | 1.05 |
| 0.82 | 1.02 | 0.8 | 1.03 | 2.25 | 0.94 | 0.63 | 0.53 | 0.6 | 0.71 | 0.88 |
| 1.09 | 1.03 | 1.16 | 0.72 | 0.95 | 0.83 | 0.96 | 0.83 | 1.08 | 0.54 | 1.03 |
| 0.89 | 1.27 | 1.11 | 0.93 | 0.86 | 1.29 | 0.71 | 2.11 | 0.56 | 1.32 | 0.63 |
| 1.67 | 0.89 | 1.37 | 1.4 | 0.84 | 0.82 | 0.28 | 4.65 | 0.9 | 1.01 | 1.34 |
| 0.71 | 0.66 | 1.44 | 0.16 | 1.65 | 0.56 | 0.11 | 3.04 | 1.15 | 2.54 | 0.77 |
| 0.76 | 0.92 | 1.54 | 1.25 | 3.18 | 6.31 | 1.08 | 1.62 | 1.15 | 1.99 | 0.97 |
| 1.27 | 0.57 | 0.53 | 1.33 | 0.95 | 0.91 | 1.02 | 2.16 | 1.31 | 1.07 | 0.71 |
| 0.49 | 0.77 | 1.21 | 0.84 | 0.83 | 1.1 | 1.42 | 9.29 | 1.31 | 5.65 | 0.9 |
| 0.82 | 0.9 | 1.22 | 1.38 | 1.41 | 0.78 | 1.43 | 7.67 | 0.9 | 2.77 | 0.81 |
| 2.41 | 1.61 | 1.03 | 1.05 | 0.98 | 0.92 | 0.78 | 1.05 | 0.9 | 1.27 | 0.96 |
| 1.99 | 0.92 | 0.99 | 0.9 | 0.54 | 0.94 | 0.97 | 3.71 | 1.05 | 1.03 | 1.04 |
| 1.56 | 0.76 | 1.08 | 0.87 | 1.06 | 1.26 | 0.99 | 5.54 | 1.19 | 2.68 | 0.77 |
| 5.13 | 1.19 | 1.21 | 0.91 | 1.18 | 2.15 | 0.77 | 0.76 | 1.23 | 1.13 | 0.94 |
| 0.91 | 0.9 | 0.95 | 1.26 | 2.76 | 1.61 | 1.41 | 0.93 | 1.29 | 0.78 | 1.03 |
| 0.96 | 1.07 | 1.72 | 1.09 | 1.73 | 0.94 | 1.12 | 2.67 | 1.26 | 1.51 | 1.28 |
| 1 | 0.87 | 0.77 | 0.89 | 1.06 | 0.51 | 1.15 | 0.15 | 0.95 | 1.08 | 0.97 |
| 1.39 | 1.31 | 1.22 | 1.22 | 1.98 | 1.08 | 3.84 | 9.91 | 1.25 | 9.04 | 2.1 |
| 0.88 | 1.07 | 0.96 | 0.87 | 1.38 | 1.85 | 0.64 | 2.72 | 1.33 | 6.49 | 0.99 |
| 2.12 | 1.13 | 1.19 | 1.77 | 0.36 | 1.72 | 0.88 | 0.5 | 1.2 | 1.28 | 1.09 |
| 0.47 | 0.99 | 0.8 | 1.79 | 0.73 | 1.02 | 1 | 0.96 | 1.29 | 0.84 | 0.85 |
| 0.94 | 0.89 | 1.41 | 0.88 | 1.34 | 0.77 | 0.94 | 6.72 | 1.11 | 0.74 | 1.1 |
| 1.34 | 1.06 | 0.92 | 1.2 | 2.63 | 1.1 | 1.15 | 0.99 | 0.98 | 0.97 | 1.1 |
| 0.69 | 1.54 | 0.78 | 1.47 | 0.62 | 0.83 | 3.39 | 1.24 | 1.45 | 15.13 | 1.27 |
| 1.25 | 1.32 | 1.24 | 0.5 | 1.13 | 0.93 | 1.53 | 1.61 | 0.71 | 0.72 | 1.01 |
| 1.44 | 0.72 | 0.53 | 1.46 | 1.25 | 1.59 | 1.03 | 2 | 0.97 | 1.27 | 0.98 |
| 1.02 | 0.98 | 0.88 | 0.97 | 7 | 1.35 | 1.08 | 5.33 | 0.97 | 0.67 | 0.83 |
| 2.5 | 1.05 | 0.93 | 1.11 | 1.57 | 0.8 | 1.22 | 5.21 | 0.71 | 1.27 | 1.07 |
| 1.39 | 0.88 | 1.65 | 0.97 | 1.99 | 1.3 | 0.45 | 0.29 | 0.73 | 3.21 | 1.09 |
| 0.94 | 0.8 | 0.95 | 1.22 | 1.07 | 1.12 | 0.95 | 0.81 | 0.87 | 1.77 | 0.96 |
| 0.82 | 1.04 | 1.15 | 1 | 1.04 | 1.34 | 1.12 | 0.73 | 0.62 | 1.52 | 0.98 |
| 2.41 | 1.16 | 1.33 | 0.98 | 1.38 | 2.47 | 1.37 | 1.71 | 1.31 | 1.41 | 1.31 |
| 0.7 | 1.04 | 1.14 | 1.55 | 1.07 | 1.15 | 1.06 | 2.14 | 1.31 | 0.94 | 0.82 |

| | | | | | | | | | | |
|---|---|---|---|---|---|---|---|---|---|---|
| 1.47 | 1.04 | 0.92 | 1.05 | 1.12 | 0.82 | 1.09 | 2.81 | 0.87 | 1.04 | 0.84 |
| 0.67 | 1.12 | 1.2 | 1.09 | 1.88 | 1.13 | 0.86 | 3.71 | 1.62 | 2.71 | 1.07 |
| 0.75 | 0.71 | 0.96 | 1.09 | 0.94 | 2.81 | 0.93 | 1.23 | 0.83 | 1 | 0.93 |
| 0.99 | 0.89 | 1.04 | 0.89 | 0.84 | 1.11 | 1.34 | 1.32 | 1.47 | 2.18 | 0.95 |
| 0.4 | 0.88 | 1.1 | 0.72 | 0.82 | 1.07 | 0.61 | 0.69 | 1.08 | 2.05 | 0.9 |
| 0.52 | 1.35 | 1.02 | 0.91 | 0.78 | 0.88 | 1.04 | 0.61 | 1.17 | 0.97 | 1.17 |
| 0.84 | 0.76 | 0.75 | 1.13 | 1.07 | 1.45 | 1.09 | 2.44 | 0.96 | 1.03 | 1.08 |
| 0.99 | 0.68 | 0.9 | 1.5 | 0.86 | 1.27 | 0.88 | 1.15 | 1.05 | 1.02 | 0.89 |
| 1.4 | 1.07 | 1.16 | 1.57 | 1.23 | 3.22 | 0.98 | 0.35 | 0.41 | 1.29 | 1.01 |
| 0.87 | 1.36 | 0.87 | 1.08 | 0.97 | 1.29 | 1.21 | 0.66 | 1.09 | 0.61 | 0.97 |
| 0.42 | 0.86 | 1.37 | 1 | 2.88 | 2.14 | 0.68 | 1.35 | 0.61 | 2.11 | 1.02 |
| 1.04 | 0.85 | 1.8 | 0.85 | 5.61 | 1.51 | 0.84 | 0.86 | 0.63 | 0.84 | 0.79 |
| 1.5 | 1.26 | 1.1 | 1.23 | 3.17 | 1.47 | 3.36 | 2.4 | 1.27 | 5.96 | 2.24 |
| 0.85 | 0.98 | 0.81 | 1.32 | 3.76 | 1.74 | 4.96 | 1.35 | 1.01 | 1.15 | 0.89 |
| 1.22 | 0.2 | 0.42 | 1.51 | 0.95 | 1.3 | 0.53 | 1.11 | 0.76 | 1.07 | 0.78 |
| 1.09 | 1.04 | 1.09 | 1.3 | 1.17 | 1.77 | 0.98 | 1 | 1.22 | 1.1 | 0.92 |
| 0.83 | 0.79 | 0.79 | 1.27 | 0.7 | 0.95 | 1.1 | 5.64 | 1.16 | 0.98 | 0.96 |
| 1.33 | 1.25 | 0.95 | 0.75 | 1.19 | 0.88 | 1.05 | 1.61 | 0.97 | 1.11 | 1.48 |
| 0.95 | 1.06 | 1 | 0.93 | 0.94 | 1.16 | 0.74 | 1.22 | 0.92 | 1.06 | 1 |
| 0.35 | 0.88 | 0.87 | 0.87 | 0.97 | 1.06 | 1.43 | 0.26 | 1.18 | 0.96 | 0.64 |
| 0.24 | 0.63 | 1.1 | 0.93 | 0.94 | 0.63 | 0.87 | 5.47 | 1.12 | 2.25 | 1.07 |
| 0.19 | 1.04 | 0.67 | 0.97 | 4.39 | 0.98 | 1.7 | 0.94 | 0.79 | 3.06 | 1.43 |
| 1.29 | 1.19 | 1.11 | 1.11 | 1.27 | 1.19 | 1.09 | 1.55 | 1.07 | 1.84 | 1.07 |
| 1.41 | 0.9 | 0.89 | 0.91 | 1.41 | 0.85 | 1.11 | 0.44 | 0.78 | 3.78 | 1.13 |
| 0.53 | 0.56 | 0.75 | 0.72 | 0.99 | 0.8 | 1.16 | 0.7 | 0.97 | 0.87 | 0.8 |
| 4.45 | 0.87 | 0.79 | 0.97 | 1.07 | 0.88 | 2.29 | 0.74 | 0.85 | 1.32 | 1.15 |
| 0.86 | 1.28 | 1.02 | 1.32 | 1.5 | 1.88 | 1 | 0.9 | 0.85 | 1.14 | 1 |
| 0.67 | 0.48 | 0.86 | 1.13 | 0.77 | 1.36 | 1 | 2.28 | 0.97 | 1.33 | 0.82 |
| 0.87 | 0.81 | 0.97 | 1.23 | 1.66 | 1.3 | 1.19 | 0.97 | 1.26 | 1.09 | 0.9 |
| 0.48 | 0.6 | 0.82 | 1.11 | 1.09 | 2.75 | 0.85 | Inf | 1.01 | 1.06 | 0.82 |
| 0.85 | 1.28 | 0.88 | 1.17 | 0.89 | 1.12 | 1.17 | 4.4 | 1.02 | 1.5 | 1.11 |
| 0.96 | 0.97 | 0.95 | 1.02 | 1.07 | 1.09 | 1.07 | Inf | 1.09 | 0.9 | 0.9 |
| 0.99 | 1.15 | 0.96 | 1.09 | 1.35 | 0.96 | 1.06 | 3.17 | 0.89 | 0.86 | 1.06 |

| | | | | | | | | | | |
|---|---|---|---|---|---|---|---|---|---|---|
| 0.97 | 1.24 | 0.97 | 0.49 | 2.43 | 0.78 | 0.9 | 0.49 | 0.47 | 0.68 | 1.04 |
| 1.08 | 1.34 | 0.92 | 1.08 | 0.97 | 1 | 1.17 | 4.21 | 0.92 | 2.48 | 1.11 |
| 1.01 | 1.02 | 1.08 | 0.98 | 1.04 | 1.03 | 0.89 | 0.94 | 0.91 | 0.45 | 1.11 |
| 2.34 | 0.35 | 0.7 | 1.52 | 2.85 | 1.31 | 1.33 | 1.5 | 1.39 | 0.33 | 0.87 |
| 2.98 | 1 | 1.15 | 0.42 | 1.27 | 0.72 | 0.59 | 7.09 | 1.25 | 1.36 | 0.96 |
| 0.99 | 1 | 0.87 | 0.99 | 1.21 | 1.06 | 0.85 | 1.23 | 0.87 | 1.99 | 0.97 |
| 0.81 | 0.66 | 1.28 | 0.67 | 1.08 | 1.27 | 0.81 | 3.06 | 0.66 | 1 | 1.06 |
| 0.94 | 0.91 | 0.99 | 0.72 | 5.91 | 1.96 | 0.97 | 0.95 | 1.02 | 1.16 | 1.16 |
| 1.8 | 1.31 | 1.12 | 0.89 | 1.84 | 1.53 | 1.4 | 11.05 | 0.86 | 1.07 | 0.97 |
| 0.81 | 0.74 | 1.41 | 0.57 | 1.01 | 1.33 | 0.77 | 2.01 | 0.63 | 2.09 | 1.02 |
| 2.28 | 0.86 | 1.01 | 0.84 | 1.15 | 1.23 | 0.47 | 1.27 | 1.29 | 2.1 | 1.27 |
| 0.91 | 1.57 | 0.95 | 2 | 0.68 | 0.56 | 1.15 | 2.13 | 0.62 | 0.51 | 1.04 |
| 0.79 | 0.74 | 0.76 | 0.78 | 0.46 | 0.86 | 0.41 | 0.58 | 1.24 | 1.07 | 1.06 |
| 0.98 | 0.9 | 0.98 | 0.98 | 1.12 | 0.59 | 0.77 | 0.91 | 0.83 | 0.84 | 0.99 |
| 1.07 | 0.91 | 1.03 | 0.99 | 0.87 | 1.1 | 1.52 | 2.23 | 1.42 | 1.45 | 0.86 |
| 0.67 | 1.53 | 2.86 | 0.87 | 0.91 | 1.5 | 0.91 | 1.23 | 1.13 | 0.67 | 0.97 |
| 0.83 | 1.14 | 0.94 | 1.04 | 1.31 | 1.16 | 1.2 | 2.54 | 0.95 | 0.93 | 1.1 |
| 0.92 | 0.75 | 1.04 | 0.95 | 1 | 0.99 | 0.84 | 2.49 | 0.86 | 1.42 | 0.93 |
| 1.37 | 1.16 | 0.57 | 0.86 | 0.75 | 0.9 | 1.25 | 0.54 | 0.64 | 2.48 | 1.24 |
| 1.19 | 0.72 | 1.58 | 0.94 | 0.41 | 0.57 | 1.29 | 0.52 | 1.34 | 1.24 | 1.05 |
| 1.35 | 1 | 1.12 | 1.02 | 0.91 | 1.33 | 0.66 | 0.28 | 0.85 | 1.06 | 1.15 |
| 5.83 | 3.23 | 1.25 | 0.5 | 2.06 | 0.49 | 0.37 | 0.37 | 1.16 | 0.03 | 0.7 |
| 1.07 | 0.98 | 1.05 | 1.06 | 1.26 | 0.87 | 0.85 | 2.43 | 0.88 | 1.08 | 1.02 |
| 0.88 | 1.15 | 1.2 | 0.63 | 0.53 | 0.87 | 0.85 | 0.59 | 0.8 | 0.75 | 0.68 |
| 0.81 | 0.97 | 0.74 | 0.92 | 0.98 | 0.85 | 1.37 | 1.6 | 1.09 | 1.14 | 0.98 |
| 1.97 | 0.7 | 0.78 | 0.77 | 1.44 | 0.94 | 0.7 | 3.61 | 0.86 | 0.01 | 13.04 |
| 0.87 | 0.86 | 1.45 | 1.38 | 0.62 | 1.23 | 1.53 | 1.75 | 1.08 | 0.94 | 1.27 |
| 1.04 | 1.67 | 1 | 0.9 | 1.3 | 1.14 | 0.82 | 2.11 | 1.01 | 3.51 | 1.1 |
| 0.99 | 0.94 | 0.79 | 1.08 | 1.47 | 1.15 | 0.99 | 1.44 | 0.97 | 0.83 | 0.94 |
| 0.99 | 0.95 | 0.98 | 0.79 | 2.73 | 0.89 | 1.03 | 0.36 | 0.83 | 0.81 | 1.08 |
| 0.96 | 0.83 | 1.05 | 1.23 | 4.76 | 1.64 | 1.39 | 0.45 | 1.16 | 0.76 | 0.86 |
| 0.79 | 0.67 | 0.9 | 0.76 | 1.21 | 0.95 | 0.86 | 0.75 | 0.9 | 1 | 0.84 |
| 10.67 | 1 | 1.04 | 0.67 | 2.45 | 1.83 | 0.7 | 2.44 | 1.5 | 1.16 | 0.87 |

| | | | | | | | | | | |
|---|---|---|---|---|---|---|---|---|---|---|
| 0.46 | 1.05 | 0.87 | 1.53 | 1.5 | 1.62 | 0.78 | 1.09 | 1.25 | 1.11 | 0.84 |
| 1.35 | 2.1 | 1.24 | 0.97 | 1.57 | 0.89 | 0.99 | 0.4 | 1.81 | 1.36 | 0.98 |
| 0.62 | 1.05 | 0.86 | 0.94 | 2.09 | 0.87 | 6.85 | 0.77 | 0.93 | 2.41 | 1.33 |
| 0.54 | 0.62 | 1.19 | 0.77 | 0.45 | 1.03 | 0.65 | 3.36 | 0.39 | 0.87 | 0.7 |
| 0.98 | 1.15 | 1.17 | 0.9 | 1.06 | 0.84 | 1.49 | 1.39 | 1.05 | 0.89 | 1.16 |
| 0.85 | 0.63 | 0.96 | 1.07 | 0.83 | 1.06 | 1.21 | 0.85 | 0.77 | 1 | 0.99 |
| 0.45 | 0.68 | 0.74 | 1.41 | 1.04 | 0.8 | 2.22 | 1.2 | 1.16 | 0.65 | 0.73 |
| 0.77 | 0.89 | 1.2 | 1.28 | 0.32 | 0.97 | 0.91 | 8.67 | 0.97 | 0.49 | 0.92 |
| 0.86 | 1.35 | 1.32 | 0.47 | 1.05 | 0.26 | 0.74 | 0.56 | 0.59 | 1.08 | 0.86 |
| 0.69 | 0.74 | 0.76 | 1.49 | 0.81 | 1.21 | 1 | 1.19 | 1.08 | 1.59 | 0.57 |
| 0.93 | 0.71 | 1.36 | 1.07 | 0.73 | 0.86 | Inf | 3.05 | 1.02 | 1 | 0.68 |
| 1.03 | 0.95 | 1.06 | 1.26 | 0.7 | 0.75 | 1.31 | 0.46 | 0.9 | 1.06 | 0.94 |
| 0.71 | 1.3 | 0.81 | 0.84 | 1.39 | 0.6 | 0.8 | 1.39 | 0.81 | 3.5 | 1.01 |
| 0.81 | 1.16 | 1.07 | 1.34 | 1.28 | 1.4 | 1.1 | 1.97 | 0.84 | 1.43 | 1.07 |
| 0.46 | 0.81 | 1.23 | 1.03 | 1.01 | 1.74 | 0.96 | 1.47 | 1.01 | 1.5 | 0.81 |
| 0.87 | 0.84 | 1.04 | 0.63 | 1.05 | 0.84 | 0.71 | 3.21 | 1.24 | 0.71 | 1.22 |
| 0.79 | 1.37 | 1.02 | 0.95 | 0.88 | 0.65 | 0.93 | 2.28 | 1.32 | 1.32 | 1.15 |
| 0.67 | 0.72 | 0.89 | 0.93 | 3.59 | 1.34 | 1.26 | 0.96 | 0.8 | 0.63 | 0.94 |
| 0.48 | 0.57 | 2.87 | 0.82 | 0.95 | 1.44 | 0.49 | 7.67 | 1.5 | 0.87 | 0.83 |
| 0.42 | 0.47 | 0.66 | 0.71 | 1.73 | 2.42 | 2.67 | 0.68 | 2.6 | 1.14 | 0.82 |
| 1.57 | 0.92 | 0.95 | 0.78 | 1.2 | 0.64 | 1.4 | 2.1 | 0.84 | 1.22 | 1.01 |
| 0.76 | 0.75 | 1.39 | 0.66 | 1.02 | 1.22 | 0.74 | 2.43 | 0.65 | 1.97 | 1.02 |
| 11.22 | 0.86 | 1.16 | 1 | 1.14 | 1.27 | 0.88 | 0.09 | 1.13 | 0.66 | 1.53 |
| 1.18 | 0.84 | 1.19 | 1 | 1.1 | 0.99 | 0.65 | 0.3 | 1.15 | 0.89 | 0.85 |
| 0.67 | 1.07 | 1.08 | 1.03 | 0.85 | 1.33 | 1.13 | 1.52 | 0.88 | 1.04 | 0.76 |
| 0.68 | 0.85 | 1 | 0.89 | 1.88 | 2.36 | 0.57 | 2.45 | 1.3 | 1.04 | 0.92 |
| 1.23 | 0.99 | 1.08 | 1.06 | 0.96 | 1.5 | 0.98 | 0.7 | 0.91 | 2.59 | 1.43 |
| 0.6 | 2.37 | 1.13 | 1.47 | 1.04 | 0.46 | 0.92 | 2.72 | 0.96 | 0.66 | 0.94 |
| 1.54 | 0.81 | 0.9 | 1.46 | 8.89 | 3.61 | 0.78 | 1.13 | 1.15 | 3.28 | 1.06 |
| 2.9 | 0.28 | 1.03 | 0.22 | 2.67 | 1.42 | 0.89 | 1.64 | 0.83 | 0.83 | 0.5 |
| 1.41 | 1.26 | 0.85 | 1 | 0.6 | 0.8 | 0.82 | 3.57 | 0.87 | 1.47 | 1.4 |
| 0.44 | 0.89 | 1.23 | 2.35 | 0.93 | 1.32 | 1.3 | 1.12 | 1.28 | 5.81 | 1 |
| 1.41 | 0.48 | 1.11 | 1.95 | 1.51 | 2.36 | 0.75 | 0.86 | 0.74 | 0.63 | 3.49 |

| | | | | | | | | | | |
|---|---|---|---|---|---|---|---|---|---|---|
| 0.65 | 0.99 | 0.83 | 0.85 | 1.21 | 0.67 | 1.15 | 0.86 | 1.27 | 5.12 | 0.82 |
| 1.27 | 1.36 | 0.9 | 0.92 | 0.88 | 1.35 | 0.17 | 0.55 | 1.29 | 1.24 | 1.12 |
| 3.26 | 0.89 | 2.37 | 5.96 | 1.11 | 11.62 | 3.67 | 0.13 | 2.26 | 0.22 | 0.69 |
| 0.62 | 0.77 | 0.83 | 0.59 | 0.67 | 1.8 | 0.67 | 18.06 | 0.65 | 4.27 | 0.9 |
| 1.25 | 0.87 | 1.02 | 0.89 | 0.62 | 0.6 | 0.83 | 1.29 | 1.61 | 0.9 | 0.86 |
| 2.41 | 0.89 | 1.07 | 0.99 | 1.36 | 1.47 | 1.13 | 0.57 | 0.96 | 2 | 1.07 |
| 1.13 | 0.91 | 1.01 | 1.1 | 1.63 | 0.95 | 1.14 | 1.07 | 1.12 | 1.12 | 0.95 |
| 3.29 | 0.98 | 1.05 | 1.15 | 0.97 | 1.73 | 0.86 | 0.54 | 1.18 | 1.03 | 1.06 |
| 1.26 | 0.81 | 0.94 | 0.89 | 1.36 | 1.32 | 1.26 | 2.7 | 1.09 | 2.87 | 0.78 |
| 0.83 | 1.16 | 1.22 | 1.18 | 0.95 | 1.1 | 0.97 | 12.15 | 1.08 | 0.87 | 0.93 |
| 0.6 | 1.13 | 1.26 | 1.12 | 1.05 | 0.74 | 1.37 | 0.69 | 1.6 | 5.87 | 0.97 |
| 2.33 | 3.75 | 1.18 | 3.52 | 0.06 | 0.61 | 10 | 0.1 | 1.3 | 0.34 | 29.33 |
| 1.72 | 0.99 | 1.16 | 1.23 | 1.02 | 0.97 | 1.39 | 0.92 | 1.16 | 1.38 | 1.12 |
| 2.28 | 0.06 | 0.94 | 0.46 | 0.58 | 0.59 | 1.2 | 5 | 1.05 | 1.45 | 1.43 |

| 211370_s_at | 212983_at | 204947_at | 213342_at | 201502_s_at | 205604_at | 218902_at | 201131_s_at | 205192_at | 221820_s_at | 206657_s_at |
|---|---|---|---|---|---|---|---|---|---|---|
| 0.67 | 1.29 | 1.26 | 1.02 | 1.09 | 1.44 | 1.27 | 0.94 | 0.95 | 0.92 | 1.77 |
| 0.94 | 0.97 | 1.39 | 2.17 | 2.23 | 0.87 | 1.02 | 0.87 | 0.75 | 1.03 | 0.95 |
| 9.06 | 1.51 | 1.07 | 2.24 | 0.67 | 1.05 | 1.77 | 4.13 | 0.77 | 0.85 | 0.85 |
| 2.8 | 1.07 | 0.97 | 1.37 | 1.11 | 1.69 | 1.36 | 1.06 | 2.03 | 1.2 | 2.44 |
| 1.13 | 0.88 | 2.02 | 1.07 | 2.56 | 0.94 | 1.27 | 0.32 | 0.88 | 1.07 | 1.47 |
| 23.33 | 0.83 | 1.16 | 3.76 | 0.98 | 9.23 | 0.91 | 2.39 | 0.77 | 1.53 | 1.03 |
| 0.73 | 2.06 | 0.42 | 1.04 | 1.29 | 0.85 | 0.34 | 0.25 | 1.39 | 1.39 | 0.38 |
| 1.16 | 1.04 | 0.81 | 3.5 | 1.6 | 1.93 | 1.05 | 2.16 | 1.03 | 0.98 | 1.68 |
| 0.96 | 1.1 | 1.27 | 1.01 | 1.32 | 0.95 | 0.77 | 0.7 | 0.85 | 0.85 | 1.06 |
| 0.44 | 1 | 0.78 | 1.04 | 2.58 | 0.39 | 1.23 | 0.5 | 1.57 | 0.87 | 1.7 |
| 1.04 | 0.91 | 1.44 | 0.63 | 1.16 | 0.87 | 1.3 | 1.85 | 1.44 | 1.08 | 0.18 |
| 1.14 | 1.44 | 2.23 | 1.22 | 0.58 | 0.8 | 0.9 | 1.88 | 1.3 | 0.86 | 1.65 |
| 1.1 | 1.18 | 2.01 | 0.91 | 0.85 | 0.98 | 0.96 | 1.8 | 1.27 | 1.18 | 0.92 |
| 7.52 | 0.74 | 10.61 | 0.47 | 0.43 | 8.81 | 0.65 | 0.05 | 2.46 | 1.69 | 10.09 |
| 1.65 | 0.96 | 1.23 | 1.04 | 1.02 | 1.14 | 0.72 | 0.82 | 1.41 | 1.22 | 1.28 |
| 0.9 | 0.99 | 1.08 | 1.26 | 1.15 | 1.2 | 0.87 | 1.46 | 1.26 | 0.89 | 1.15 |
| 1.59 | 1.13 | 2.14 | 0.94 | 0.7 | 2.94 | 1.15 | 1.69 | 1.37 | 0.76 | 0.59 |
| 1.06 | 1.07 | 1.25 | 1.36 | 2.03 | 1.51 | 1.52 | 0.95 | 0.85 | 1.02 | 1.27 |
| 1.25 | 1.88 | 0.69 | 2.72 | 1.4 | 0.36 | 6.36 | 7 | 0.31 | 0.72 | 1.25 |
| 1.77 | 1.24 | 3.81 | 0.93 | 0.54 | 8.12 | 0.57 | 0.71 | 0.2 | 1.6 | 16.6 |
| 0.43 | 2.52 | 1.02 | 0.88 | 1.22 | 1.15 | 1.06 | 5.79 | 1.11 | 0.67 | 3.51 |
| 1.64 | 0.92 | 3.15 | 0.53 | 3.45 | 2.56 | 1.18 | 1.07 | 2.13 | 0.51 | 6.88 |
| 1.17 | 0.95 | 0.58 | 0.87 | 1.18 | 0.67 | 0.75 | 20.64 | 0.9 | 1.07 | 0.75 |
| 1.11 | 0.71 | 1 | 0.78 | 0.35 | 0.68 | 0.28 | 0 | 1.55 | 0.82 | 0.72 |
| 0.48 | 0.84 | 0.77 | 1.35 | 2.23 | 0.03 | 0.52 | 0.06 | 5.08 | 0.45 | 1.09 |
| 1.41 | 0.84 | 1.29 | 1.07 | 0.78 | 1.02 | 1.86 | 1.05 | 1.79 | 1.03 | 1.21 |
| 1.01 | 0.98 | 0.98 | 0.87 | 0.42 | 0.3 | 0.88 | 4.08 | 1.07 | 1.18 | 0.49 |
| 1.29 | 0.61 | 0.65 | 0.67 | 4.18 | 3.43 | 0.59 | 0.38 | 1.17 | 1.48 | 5.38 |
| 1.92 | 1.34 | 0.53 | 0.51 | 0.54 | 2.04 | 1.47 | 63.41 | 1.57 | 1.37 | 1.03 |
| 0.68 | 1.28 | 1.27 | 1.32 | 1.47 | 1.3 | 1.27 | 1.24 | 1.12 | 0.87 | 1.52 |

| | | | | | | | | | | |
|---|---|---|---|---|---|---|---|---|---|---|
| 0.63 | 1.54 | 0.84 | 5.19 | 0.66 | 4.9 | 0.62 | 0.49 | 0.88 | 0.81 | 1.17 |
| 1.85 | 1.69 | 2.01 | 1.16 | 0.93 | 11.01 | 0.83 | 0.18 | 1.48 | 1.61 | 1.82 |
| 1.02 | 1.11 | 1.18 | 0.96 | 0.84 | 1.71 | 1.12 | 1.04 | 1.35 | 1.06 | 1.06 |
| 1.5 | 21.77 | 2.28 | 0.78 | 0.35 | 0.96 | 0.4 | 0.26 | 0.39 | 0.64 | 1.24 |
| 1.17 | 1.9 | 1.28 | 40.4 | 2.61 | 0.77 | 0.47 | 4 | 1.15 | 0.81 | 0.8 |
| 1.13 | 2.45 | 1.31 | 1.2 | 0.81 | 9.07 | 5.39 | 0.66 | 0.76 | 0.81 | 3.77 |
| 1.35 | 0.47 | 1.47 | 0.57 | 2.35 | 0.74 | 2.09 | 11.14 | 1.31 | 1.05 | 0.44 |
| 0.6 | 1.01 | 1.1 | 0.88 | 0.71 | 0.33 | 1 | 2.5 | 0.89 | 0.74 | 1.05 |
| 0.94 | 10.21 | 1.34 | 0.63 | 3.57 | 2.57 | 0.71 | 0.98 | 1.21 | 0.82 | 1.17 |
| 1.04 | 0.74 | 1.1 | 1.02 | 1.29 | 0.56 | 0.4 | 0.12 | 1.43 | 0.9 | 6.8 |
| 1.37 | 1.72 | 0.67 | 1.08 | 0.91 | 0.89 | 0.99 | 1.83 | 1.04 | 1.52 | 1.38 |
| 0.96 | 1.04 | 0.99 | 0.9 | 1.24 | 1.1 | 1.66 | 0.35 | 0.88 | 0.81 | 1.02 |
| 1.06 | 8.05 | 0.41 | 1.36 | 3.43 | 4.72 | 0.76 | 1.35 | 0.59 | 0.76 | 1.08 |
| 1.25 | 1.74 | 1.18 | 1.62 | 0.54 | 1.24 | 1.64 | 2.6 | 1.42 | 0.6 | 0.83 |
| 1.03 | 5.64 | 0.71 | 1.24 | 2.85 | 3.49 | 0.86 | 1.32 | 0.76 | 1 | 0.97 |
| 1.04 | 1.01 | 1.44 | 1.29 | 2.51 | 1.75 | 1.06 | 9.73 | 0.95 | 1.1 | 1.16 |
| 0.92 | 1.14 | 1.2 | 0.84 | 7.24 | 1.67 | 0.86 | 1.06 | 1.81 | 0.99 | 2.82 |
| 1.12 | 0.96 | 1.02 | 1.23 | 1.45 | 0.81 | 0.85 | 0.49 | 1.47 | 0.9 | 3.06 |
| 0.98 | 1.06 | 0.79 | 0.95 | 0.94 | 1.25 | 1.2 | 0.79 | 1.03 | 0.96 | 1.14 |
| 0.89 | 1.13 | 1.31 | 0.83 | 0.6 | 0.92 | 1.29 | 0.17 | 0.81 | 1.11 | 15.27 |
| 0.83 | 0.85 | 0.77 | 0.81 | 5.73 | 0.43 | 0.69 | 0.87 | 1.45 | 0.94 | 0.62 |
| 1.12 | 1.07 | 1.07 | 0.73 | 0.72 | 3.54 | 0.86 | 0.98 | 1.53 | 0.91 | 0.78 |
| 0.99 | 0.97 | 0.84 | 1.18 | 0.85 | 1.31 | 0.82 | 1.68 | 1.04 | 0.91 | 0.85 |
| 1.12 | 1.25 | 1.03 | 2.26 | 1.21 | 2.55 | 1.02 | 1.08 | 1.11 | 0.96 | 1.2 |
| 0.65 | 4.5 | 0.94 | 1.23 | 1.24 | 2.79 | 1.67 | 4.12 | 0.52 | 0.69 | 4.95 |
| 0.91 | 1.26 | 11.03 | 1.26 | 1.54 | 0.93 | 1.01 | 0.2 | 1.15 | 1.1 | 1.09 |
| 0.59 | 1.73 | 1.37 | 1.54 | 6.08 | 0.5 | 1.08 | 4.83 | 1.04 | 0.79 | 13.89 |
| 3.08 | 0.73 | 1.16 | 1.18 | 1.78 | 1.3 | 0.88 | 1.43 | 1.38 | 2.02 | 0.51 |
| 1.14 | 0.8 | 0.72 | 0.95 | 4.1 | 0.67 | 1.52 | 1.01 | 1.09 | 1.38 | 1.17 |
| 1.03 | 4.35 | 0.92 | 1.5 | 4.97 | 4.62 | 0.86 | 1.33 | 0.87 | 0.84 | 1.42 |
| 0.95 | 1.27 | 1.01 | 1.09 | 2.33 | 2.13 | 0.61 | 0.75 | 0.68 | 0.88 | 2.05 |
| 1.01 | 0.73 | 1.35 | 0.98 | 0.6 | 0.91 | 1.39 | 1.09 | 1.54 | 0.68 | 2.17 |
| 1.15 | 0.83 | 0.59 | 1.27 | 1.04 | 1.16 | 1.53 | 3.78 | 1.3 | 1 | 1.07 |

| | | | | | | | | | | |
|---|---|---|---|---|---|---|---|---|---|---|
| 0.95 | 1.25 | 1.17 | 0.98 | 0.81 | 1.06 | 0.72 | 0.14 | 0.58 | 0.87 | 0.77 |
| 1.03 | 1.51 | 1.71 | 0.78 | 1.08 | 1.63 | 1.81 | 2.08 | 1.02 | 0.97 | 17.34 |
| 1.18 | 0.98 | 1.22 | 0.98 | 1.68 | 2.5 | 0.74 | 251.75 | 1.07 | 1.04 | 0.68 |
| 0.95 | 0.99 | 1.09 | 1.13 | 1.41 | 1.13 | 1.47 | 2.83 | 1.48 | 1.1 | 1.09 |
| 0.87 | 0.89 | 1.15 | 0.9 | 0.94 | 0.87 | 0.99 | 1.56 | 0.98 | 0.83 | 1.42 |
| 0.97 | 1.38 | 1.38 | 0.74 | 1.07 | 2.39 | 1.69 | 0.82 | 0.39 | 0.9 | 1.04 |
| 1.53 | 1.06 | 0.67 | 1.73 | 0.34 | 2.88 | 1.12 | 0.67 | 1.36 | 1.46 | 0.46 |
| 2.43 | 1.63 | 1.28 | 0.87 | 1.55 | 2.23 | 0.94 | 0.66 | 1.14 | 1.15 | 1.46 |
| 0.67 | 1.36 | 0.79 | 0.88 | 1.33 | 2.58 | 0.75 | 1.63 | 0.46 | 1.13 | 0.73 |
| 1.85 | 0.78 | 1.69 | 1.26 | 1.05 | 3.82 | 1.27 | 1.52 | 0.89 | 0.66 | 2.47 |
| 3.01 | 0.37 | 3.76 | 2.14 | 0.71 | 0.81 | 1.56 | 10.38 | 1.06 | 1.69 | 6.16 |
| 0.77 | 1.03 | 0.96 | 1.26 | 1.14 | 1.47 | 1.36 | 1.06 | 1.9 | 0.89 | 5.38 |
| 1.12 | 0.98 | 0.84 | 1.11 | 1.14 | 1.08 | 1.34 | 1.41 | 1.3 | 0.73 | 1 |
| 0.69 | 1.11 | 0.65 | 1.18 | 2.69 | 1.39 | 1.24 | 0.74 | 0.68 | 1.03 | 1.46 |
| 0.81 | 1.88 | 1.45 | 2 | 0.93 | 0.12 | 0.91 | 2.67 | 1.44 | 0.82 | 0.5 |
| 0.21 | 2.17 | 0.94 | 52.59 | 2.52 | 0.22 | 0.3 | 2.37 | 0.71 | 0.14 | 0.06 |
| 0.92 | 0.6 | 0.5 | 1.07 | 2.22 | 1.14 | 1.04 | 0.9 | 0.99 | 1.15 | 2.34 |
| 0.64 | 0.84 | 1 | 2.06 | 0.98 | 0.41 | 0.89 | 0.1 | 0.42 | 1.15 | 0.27 |
| 1 | 0.85 | 0.89 | 1.52 | 0.74 | 1.05 | 0.79 | 1.63 | 1.29 | 1.29 | 1.87 |
| 1.69 | 1.88 | 1.42 | 5.36 | 1.34 | 1.58 | 2.04 | 1.19 | 2.58 | 2.56 | 2.43 |
| 1.22 | 1.08 | 0.4 | 0.88 | 1.62 | 1.94 | 1.71 | 279 | 0.79 | 0.64 | 1.24 |
| 1.71 | 1.24 | 2.42 | 0.78 | 1.35 | 0.8 | 0.94 | 4.57 | 1.77 | 1.09 | 1.05 |
| 0.85 | 1.6 | 1.01 | 0.86 | 1.12 | 0.81 | 0.39 | 0.53 | 1.21 | 1.44 | 0.88 |
| 1.09 | 1.52 | 1.56 | 1.26 | 0.94 | 0.88 | 0.73 | 0.85 | 0.75 | 1.09 | 1.38 |
| 1.34 | 1.14 | 1.24 | 1.55 | 0.79 | 1.01 | 1.17 | 1.12 | 0.95 | 0.92 | 2.16 |
| 0.79 | 0.9 | 1.23 | 1.15 | 1.55 | 1.96 | 0.67 | 1.36 | 1.4 | 0.99 | 2.94 |
| 0.99 | 1.17 | 1.3 | 0.84 | 1.22 | 1.53 | 1.1 | 1.04 | 1.23 | 1.13 | 2.29 |
| 1.03 | 1.34 | 0.77 | 2.29 | 0.93 | 1.08 | 1.51 | 0.51 | 0.95 | 1.06 | 0.59 |
| 0.58 | 0.89 | 1.18 | 1.2 | 0.99 | 1.7 | 0.98 | 1.49 | 0.76 | 0.93 | 0.85 |
| 1 | 1.07 | 1.11 | 1.02 | 1.32 | 1.18 | 1.05 | 0.94 | 1.47 | 0.88 | 0.72 |
| 1.25 | 1.25 | 1.41 | 0.9 | 1.48 | 1.32 | 1.28 | 0.78 | 1.09 | 1.46 | 2.78 |
| 1.09 | 1.85 | 0.91 | 0.82 | 0.41 | 0.73 | 0.79 | 2.92 | 1.01 | 0.76 | 1.4 |
| 1.19 | 0.91 | 0.65 | 1.65 | 2.25 | 1.19 | 1.37 | 2 | 1.01 | 0.92 | 0.44 |

| | | | | | | | | | | |
|---|---|---|---|---|---|---|---|---|---|---|
| 0.6 | 0.69 | 1.19 | 0.8 | 0.75 | 1.06 | 1 | 1.34 | 0.7 | 1.07 | 1.91 |
| 0.82 | 1.23 | 1.53 | 0.63 | 1 | 1.15 | 1.17 | 1.67 | 0.99 | 0.91 | 1.12 |
| 0.86 | 1.26 | 1.74 | 0.99 | 1.15 | 8.78 | 1.18 | 0.98 | 0.77 | 0.88 | 6.19 |
| 0.46 | 1.5 | 1.35 | 1.11 | 1.18 | 3.77 | 1.3 | 1.31 | 0.73 | 0.99 | 1.02 |
| 1.43 | 0.9 | 1.17 | 0.73 | 0.85 | 2.58 | 0.86 | 1.47 | 1.84 | 1.27 | 0.66 |
| 0.53 | 1.74 | 0.73 | 0.6 | 3.58 | 1.78 | 0.65 | 0.85 | 1.65 | 0.39 | 2.48 |
| 1.15 | 1.2 | 0.76 | 1.16 | 1.89 | 0.94 | 0.81 | 1.73 | 2.22 | 0.87 | 1.48 |
| 0.95 | 1.04 | 1.22 | 1.26 | 1.02 | 1.58 | 0.87 | 4.3 | 0.69 | 1.07 | 1.06 |
| 0.8 | 0.73 | 0.72 | 1.23 | 0.64 | 1.94 | 0.86 | 0.73 | 1.72 | 0.95 | 1.4 |
| 1.37 | 0.79 | 0.99 | 1.19 | 1.71 | 1.06 | 0.97 | 0.67 | 1.38 | 1.29 | 0.89 |
| 1.08 | 1.17 | 0.99 | 1.2 | 0.88 | 0.93 | 1.06 | 3.03 | 1.43 | 1.33 | 2.57 |
| 1.43 | 1.04 | 1.28 | 0.91 | 0.89 | 0.85 | 1.31 | 0.98 | 1.4 | 1.07 | 1.4 |
| 0.73 | 1.18 | 1.13 | 0.91 | 1.41 | 1.42 | 1.78 | 1.5 | 0.55 | 0.82 | 1.85 |
| 1.26 | 14.7 | 2.51 | 0.98 | 0.68 | 1.07 | 0.57 | 0.52 | 0.7 | 0.81 | 1.13 |
| 1.25 | 0.9 | 0.77 | 1.11 | 1.04 | 1.22 | 0.99 | 1.09 | 1.3 | 1.11 | 1.02 |
| 0.99 | 1.06 | 1.15 | 3.63 | 1.31 | 1.19 | 1.79 | 13.72 | 3.06 | 1.12 | 1.04 |
| 0.69 | 0.94 | 0.95 | 0.56 | 0.83 | 1.27 | 1.24 | 0.7 | 1.59 | 1.11 | 0.77 |
| 1.08 | 1.12 | 1.03 | 1.29 | 2.39 | 1.03 | 1.76 | 3.63 | 1.18 | 1.08 | 0.81 |
| 0.97 | 0.85 | 0.89 | 1.48 | 4.17 | 2.73 | 1.13 | 1.39 | 1.31 | 0.78 | 0.46 |
| 0.8 | 1.4 | 1.24 | 0.96 | 0.66 | 1.3 | 1.47 | 0.07 | 0.87 | 1.12 | 1.09 |
| 0.67 | 0.9 | 1.09 | 1.34 | 0.73 | 0.8 | 1.03 | 1.2 | 0.7 | 1.06 | 3.33 |
| 0.99 | 0.96 | 0.84 | 1.03 | 1 | 1.37 | 1.18 | 0.25 | 1.32 | 1.01 | 0.66 |
| 1.21 | 0.95 | 0.76 | 0.76 | 1.05 | 1.41 | 1.13 | 1.04 | 0.97 | 0.88 | 0.67 |
| 1.98 | 0.82 | 0.41 | 0.67 | 0.52 | 3.45 | 2.95 | 0.85 | 1.73 | 1.28 | 1.28 |
| 0.84 | 0.99 | 0.9 | 1.2 | 0.48 | 0.85 | 1.7 | 7.96 | 0.93 | 1.25 | 1.63 |
| 0.96 | 0.8 | 0.88 | 1.39 | 0.88 | 1.52 | 0.54 | 0.92 | 0.85 | 1.13 | 1.36 |
| 1.05 | 1.13 | 0.25 | 0.99 | 1.14 | 1.15 | 1.01 | 0.97 | 0.96 | 0.89 | 0.11 |
| 1.52 | 0.57 | 1.09 | 0.87 | 1.19 | 0.88 | 1.5 | 2.79 | 0.76 | 1.56 | 1.1 |
| 0.62 | 0.93 | 1.68 | 1.12 | 0.58 | 0.98 | 0.51 | 1.24 | 1.2 | 0.78 | 0.94 |
| 0.9 | 0.94 | 0.91 | 0.86 | 0.98 | 2.08 | 1.12 | 1 | 0.82 | 1.1 | 1.89 |
| 0.71 | 1.11 | 0.92 | 0.2 | 1.24 | 1.75 | 1.12 | 1.61 | 1.08 | 1.03 | 0.82 |
| 1.03 | 1.51 | 1.71 | 0.78 | 1.08 | 1.63 | 1.81 | 2.08 | 1.02 | 0.97 | 17.34 |
| 0.96 | 0.9 | 1.09 | 1.15 | 3.55 | 1.92 | 0.89 | 0.93 | 0.8 | 1.07 | 0.82 |

| | | | | | | | | | | |
|---|---|---|---|---|---|---|---|---|---|---|
| 0.99 | 1 | 0.81 | 1.32 | 0.72 | 1.78 | 0.98 | 3.07 | 1.27 | 0.94 | 0.8 |
| 1.05 | 0.92 | 0.7 | 1.01 | 1.75 | 2.28 | 0.95 | 0.96 | 1.81 | 1 | 2.03 |
| 0.95 | 1.13 | 1.22 | 1.25 | 0.78 | 2.85 | 1.23 | 0.52 | 2.55 | 1.39 | 1.11 |
| 1.04 | 1.01 | 0.76 | 8.57 | 3 | 1.1 | 1.23 | 1.77 | 1.08 | 1.05 | 0.73 |
| 0.91 | 1.04 | 0.78 | 0.94 | 0.53 | 1.42 | 0.67 | 6.35 | 1.74 | 1.02 | 1.35 |
| 0.78 | 0.92 | 1.27 | 1.05 | 0.93 | 0.28 | 1.04 | 1.31 | 0.93 | 1.08 | 0.21 |
| 0.84 | 0.9 | 0.82 | 0.94 | 0.96 | 1.32 | 1.2 | 0.92 | 1.09 | 0.96 | 0.6 |
| 1.41 | 0.75 | 0.44 | 1.18 | 0.67 | 0.92 | 1.28 | 3.52 | 1.4 | 1.13 | 1.58 |
| 1.18 | 1.01 | 1.76 | 0.62 | 1.02 | 1.61 | 1.47 | 2.27 | 0.96 | 0.58 | 1.23 |
| 1.06 | 1.28 | 1.2 | 1.05 | 1.89 | 4.52 | 1.38 | 1.5 | 1.12 | 0.97 | 0.65 |
| 0.68 | 1.1 | 0.83 | 0.59 | 1.21 | 0.5 | 1.05 | 0.51 | 0.66 | 0.89 | 1 |
| 3.09 | 1.26 | 1.12 | 1.09 | 1.52 | 0.61 | 0.8 | 1.28 | 0.73 | 1.13 | 2.57 |
| 1.38 | 1.05 | 1.02 | 1.15 | 1.46 | 0.99 | 1.72 | 2.18 | 1.02 | 1.37 | 1.06 |
| 0.84 | 0.89 | 0.6 | 1.08 | 1.73 | 0.44 | 1.53 | 2.19 | 1.18 | 0.97 | 0.76 |
| 1.38 | 0.84 | 0.31 | 2.65 | 1.3 | 1.83 | 0.45 | 1.87 | 0.44 | 0.6 | 0.94 |
| 1.11 | 1.05 | 1.2 | 0.28 | 0.68 | 1.11 | 1.07 | 0.8 | 1.01 | 0.97 | 1.62 |
| 0.99 | 1.1 | 1.22 | 0.84 | 0.87 | 1.52 | 0.89 | 1.06 | 1.81 | 1.14 | 1.19 |
| 1.08 | 1.19 | 10.89 | 2.47 | 1.29 | 2.34 | 1.03 | 7.66 | 0.97 | 0.92 | 1.08 |
| 0.93 | 0.83 | 0.91 | 0.87 | 1.01 | 0.88 | 0.8 | 2.43 | 1.15 | 1.03 | 1.44 |
| 0.81 | 1.07 | 1.07 | 1.02 | 0.57 | 1.45 | 0.85 | 1.15 | 0.96 | 1.43 | 0.91 |
| 1.04 | 1.12 | 1.82 | 1.15 | 1.31 | 0.9 | 1.46 | 0.95 | 1.34 | 0.85 | 1.97 |
| 1.8 | 0.66 | 1.08 | 1.23 | 1.19 | 2.31 | 0.46 | 0.96 | 0.95 | 1.05 | 2.03 |
| 1.34 | 1.35 | 1.18 | 3.56 | 0.86 | 1.1 | 1.02 | 0.86 | 1.02 | 0.92 | 1.49 |
| 0.84 | 0.92 | 2.58 | 1.37 | 0.69 | 3.05 | 1.37 | 0.34 | 1.05 | 1.04 | 1.1 |
| 1.01 | 2.54 | 0.76 | 1.32 | 1.04 | 0.34 | 1.32 | 1.81 | 0.91 | 0.77 | 2.37 |
| 1.1 | 1.42 | 0.9 | 0.82 | 1.19 | 1.07 | 1.06 | 1.1 | 1.78 | 1.1 | 1.11 |
| 1.37 | 1.28 | 1.25 | 0.83 | 3.06 | 1.21 | 0.88 | 1.98 | 0.72 | 0.7 | 1 |
| 0.75 | 1.06 | 0.97 | 1.13 | 0.94 | 1.48 | 1.25 | 0.99 | 0.95 | 0.91 | 0.73 |
| 1.07 | 1.03 | 0.66 | 1.2 | 0.81 | 1.17 | 1.05 | 1.67 | 1.24 | 0.93 | 0.95 |
| 1.21 | 0.98 | 1.4 | 0.83 | 2 | 0.53 | 0.97 | 0.52 | 1.08 | 0.78 | 1 |
| 0.93 | 1.14 | 1.53 | 2.19 | 0.78 | 2.31 | 0.85 | 1.09 | 0.96 | 0.93 | 2.15 |
| 0.92 | 1.01 | 1.04 | 1.03 | 1.66 | 1.44 | 0.8 | 1 | 1.44 | 0.93 | 1 |
| 0.99 | 1.12 | 0.95 | 3.17 | 1.06 | 3.15 | 0.8 | 0.84 | 1.07 | 1.06 | 1.05 |

| | | | | | | | | | | |
|---|---|---|---|---|---|---|---|---|---|---|
| 0.65 | 1.86 | 1.62 | 1.31 | 1.15 | 2.07 | 0.69 | 1.65 | 0.92 | 1.02 | 1.13 |
| 1.04 | 1.31 | 1.09 | 1.12 | 0.74 | 1.3 | 0.85 | 1.73 | 0.9 | 0.93 | 1.16 |
| 1.07 | 1 | 0.93 | 1.04 | 1.04 | 1.08 | 0.93 | 0.84 | 1.08 | 1.01 | 0.94 |
| 1.02 | 0.53 | 1.03 | 0.5 | 1.07 | 2 | 1.34 | 0.55 | 0.91 | 1.03 | 0.73 |
| 0.73 | 1.13 | 0.71 | 0.75 | 1.3 | 3.52 | 0.68 | 1.04 | 0.85 | 0.42 | 1.28 |
| 0.93 | 1.08 | 0.99 | 0.95 | 1.19 | 0.84 | 1.2 | 1.46 | 0.97 | 0.96 | 1.05 |
| 0.66 | 1.55 | 0.76 | 4.07 | 0.66 | 5.61 | 0.66 | 0.57 | 0.76 | 0.8 | 1.34 |
| 0.92 | 0.81 | 1.05 | 0.77 | 0.87 | 0.85 | 1.4 | 2.47 | 1.34 | 1.2 | 1.44 |
| 0.9 | 0.91 | 1.28 | 1.62 | 1.45 | 0.89 | 1.18 | 0.53 | 1.28 | 1.12 | 0.82 |
| 0.58 | 1.38 | 1.03 | 2.85 | 0.62 | 2.79 | 0.6 | 2.12 | 0.68 | 0.75 | 1.14 |
| 1.04 | 1.09 | 0.52 | 0.93 | 1.18 | 0.38 | 1.18 | 0.81 | 0.84 | 0.9 | 0.86 |
| 1.05 | 1.8 | 1.37 | 1.27 | 0.9 | 0.67 | 0.52 | 2.12 | 0.9 | 1.09 | 0.7 |
| 0.72 | 0.9 | 0.66 | 0.85 | 0.87 | 1.55 | 0.84 | 1.02 | 1.05 | 0.93 | 1.32 |
| 1.15 | 0.86 | 0.76 | 4.53 | 0.92 | 1.24 | 0.93 | 5.69 | 1.08 | 0.82 | 1.1 |
| 1.11 | 0.82 | 0.94 | 3.14 | 3.13 | 1.29 | 1.12 | 2.01 | 0.94 | 1 | 1 |
| 1.37 | 0.99 | 0.51 | 1.18 | 1.35 | 1.83 | 0.71 | 0.93 | 0.81 | 1.29 | 1 |
| 1.06 | 0.96 | 1.03 | 5 | 0.96 | 1.15 | 1.14 | 1.75 | 1.17 | 0.97 | 1.49 |
| 0.9 | 0.91 | 0.82 | 1.01 | 1.89 | 2.74 | 1.17 | 1.13 | 0.81 | 0.78 | 1.74 |
| 0.61 | 0.57 | 1.29 | 1.38 | 0.83 | 0.65 | 0.93 | 0.92 | 0.84 | 1.35 | 0.87 |
| 0.83 | 0.78 | 1.08 | 1.48 | 0.77 | 3.71 | 0.8 | 1.08 | 0.55 | 0.69 | 2.25 |
| 1.25 | 1.02 | 1.11 | 0.9 | 1.13 | 1.58 | 2.1 | 0.9 | 1.11 | 0.94 | 1.25 |
| 4.17 | 2.17 | 0.52 | 0.94 | 3 | 0.89 | 1.09 | 709.31 | 1.71 | 1.96 | 0.81 |
| 1.13 | 1.08 | 1.22 | 1.3 | 0.91 | 0.8 | 0.89 | 6.58 | 1.43 | 0.99 | 0.83 |
| 0.52 | 1.18 | 1.13 | 1.58 | 1 | 1.96 | 0.98 | 1.43 | 0.62 | 1.22 | 1.22 |
| 1.18 | 0.98 | 0.72 | 1.04 | 0.98 | 0.48 | 0.87 | 2.31 | 0.81 | 0.93 | 4.16 |
| 0.99 | 0.54 | 0.72 | 0.84 | 0.21 | 0.22 | 1.75 | 0.22 | 0.94 | 0.7 | 0.87 |
| 1.06 | 1 | 1.12 | 1.39 | 1.11 | 1.01 | 1 | 1.04 | 0.97 | 0.98 | 0.6 |
| 0.93 | 1.16 | 1.85 | 2.72 | 1.06 | 0.78 | 1.14 | 0.68 | 1.1 | 1.04 | 1.4 |
| 1.03 | 1.04 | 0.9 | 3.84 | 0.97 | 1.51 | 0.97 | 0.83 | 1.09 | 1.13 | 1.27 |
| 0.67 | 1.18 | 1.07 | 0.97 | 0.92 | 0.95 | 1.02 | 1.17 | 1.06 | 1.01 | 0.83 |
| 1.18 | 1.51 | 1.54 | 1.14 | 0.86 | 2.22 | 0.89 | 1.16 | 1.6 | 1.43 | 1.11 |
| 0.83 | 1.02 | 0.53 | 0.9 | 1.07 | 0.86 | 0.88 | 1.08 | 0.99 | 0.83 | 0.38 |
| 1.44 | 0.95 | 0.96 | 0.66 | 1.1 | 0.97 | 1.23 | 5.57 | 1.6 | 1.23 | 0.96 |

| | | | | | | | | | | |
|---|---|---|---|---|---|---|---|---|---|---|
| 2.36 | 2.12 | 0.6 | 0.95 | 1.53 | 0.85 | 1.13 | 0.11 | 1.27 | 0.85 | 1.88 |
| 0.97 | 0.85 | 2.03 | 1.44 | 0.52 | 2.29 | 1.4 | 1.4 | 0.87 | 0.75 | 2.33 |
| 1.07 | 0.95 | 1.76 | 0.79 | 0.98 | 1.39 | 0.85 | 1.05 | 0.9 | 1 | 1.54 |
| 0.65 | 0.84 | 1.1 | 0.74 | 0.54 | 0.41 | 0.63 | 1.33 | 0.75 | 1.09 | 0.87 |
| 1.04 | 1.13 | 6.79 | 2.21 | 0.73 | 4.51 | 1.04 | 1.83 | 1.14 | 1.27 | 1.1 |
| 0.92 | 0.85 | 0.91 | 1.08 | 1.08 | 1.5 | 1.04 | 2.28 | 0.74 | 1.12 | 2.19 |
| 1.09 | 0.93 | 0.64 | 0.78 | 0.48 | 0.93 | 0.56 | 1.1 | 1.22 | 1.48 | 0.56 |
| 1.19 | 1.06 | 0.76 | 0.29 | 0.85 | 0.17 | 0.63 | 0.49 | 1.04 | 0.88 | 0.32 |
| 0.45 | 1.32 | 1.58 | 0.79 | 1.46 | 1.22 | 0.82 | 1.64 | 0.77 | 1.42 | 1.16 |
| 0.98 | 0.73 | 0.81 | 1.4 | 1.23 | 1.13 | 1.16 | 0.56 | 0.88 | 0.8 | 1.37 |
| 0.81 | 0.81 | 0.77 | 1.33 | 1.13 | 1.44 | 0.32 | 1.13 | 0.58 | 1 | 0.59 |
| 1.08 | 0.97 | 1.12 | 0.62 | 0.77 | 0.89 | 0.91 | 0.39 | 1.12 | 1.06 | 0.8 |
| 0.81 | 0.86 | 1.1 | 0.8 | 3.55 | 0.36 | 0.83 | 1.07 | 1.46 | 0.71 | 1 |
| 0.97 | 0.92 | 0.94 | 0.17 | 1.11 | 0.85 | 1.11 | 5.92 | 0.93 | 1.05 | 3.28 |
| 1.27 | 0.75 | 0.58 | 0.92 | 1.2 | 0.65 | 1 | 1.22 | 0.72 | 0.85 | 0.94 |
| 0.95 | 0.88 | 1.39 | 1.01 | 0.56 | 1.59 | 0.9 | 1.69 | 0.75 | 0.9 | 2.08 |
| 0.81 | 0.87 | 0.97 | 0.82 | 0.82 | 0.7 | 1.47 | 0.3 | 1.24 | 0.93 | 1 |
| 0.93 | 1.09 | 0.72 | 9.36 | 0.78 | 1.04 | 0.7 | 0.55 | 0.84 | 0.94 | 0.52 |
| 0.52 | 0.92 | 0.66 | 1.3 | 1.21 | 2.11 | 1.06 | 0.3 | 1.83 | 1.23 | 1.88 |
| 0.82 | 0.41 | 0.43 | 0.5 | 1.07 | 0.91 | 0.4 | 0.03 | 4.06 | 1.08 | 1.35 |
| 2.32 | 0.96 | 1 | 0.9 | 0.83 | 0.77 | 1.07 | 1.16 | 1.37 | 1.2 | 0.73 |
| 0.59 | 1.45 | 0.98 | 4.58 | 0.64 | 3.48 | 0.6 | 1.27 | 0.84 | 0.78 | 1.07 |
| 0.56 | 1.31 | 2.47 | 0.99 | 0.37 | 2.4 | 0.56 | 0.81 | 1.03 | 0.98 | 0.89 |
| 0.75 | 1.27 | 1.49 | 1.11 | 0.35 | 1.6 | 0.53 | 0 | 0.35 | 0.65 | 1.24 |
| 0.97 | 1.11 | 0.61 | 0.99 | 1.23 | 1.4 | 1.1 | 1.04 | 1.38 | 0.75 | 1.36 |
| 1.19 | 1.26 | 1.27 | 1.39 | 1.15 | 3.79 | 1.04 | 0.99 | 1.61 | 1.16 | 1.53 |
| 0.8 | 0.63 | 2.51 | 0.63 | 0.36 | 0.71 | 1.18 | 0.86 | 0.54 | 0.83 | 0.77 |
| 1.27 | 0.99 | 3.31 | 1.14 | 1.34 | 0.7 | 0.49 | 0.2 | 1.37 | 1.38 | 0.4 |
| 0.52 | 1.47 | 1.65 | 1.73 | 2.01 | 0.82 | 0.7 | 0.85 | 1.65 | 0.97 | 0.96 |
| 0.11 | 1.83 | 1.67 | 3.36 | 0.26 | 3.51 | 3 | 1.13 | 1.24 | 0.35 | 1.75 |
| 1.24 | 1.19 | 0.81 | 2.14 | 0.87 | 3.33 | 0.71 | 2 | 0.89 | 0.9 | 0.5 |
| 0.75 | 1.48 | 1.25 | 2.41 | 1.68 | 1.2 | 1.1 | 0.93 | 3.64 | 0.64 | 0.72 |
| 1.32 | 1.41 | 1.22 | 1.58 | 1.19 | 1.17 | 1.81 | 2.11 | 3.66 | 0.73 | 5.38 |

| | | | | | | | | | | |
|---|---|---|---|---|---|---|---|---|---|---|
| 1.18 | 1.16 | 1.21 | 0.88 | 0.88 | 1 | 1 | 4.12 | 0.92 | 1.05 | 0.96 |
| 1.15 | 1.17 | 1.08 | 0.55 | 1.21 | 0.96 | 0.79 | 0.75 | 0.94 | 0.91 | 1.38 |
| 0.74 | 0.52 | 18.77 | 8.79 | 0.73 | 1.53 | 0.96 | 2.87 | 0.43 | 1.06 | 0.65 |
| 0.94 | 0.85 | 5.07 | 0.7 | 0.92 | 2.93 | 0.96 | 0.23 | 0.71 | 1.18 | 6.22 |
| 0.84 | 0.78 | 0.76 | 1.01 | 0.84 | 1.24 | 0.97 | 56.41 | 1.34 | 1.08 | 1.43 |
| 0.99 | 1.27 | 1.28 | 0.73 | 1.05 | 1.04 | 1.07 | 2.31 | 0.81 | 1.18 | 1.49 |
| 0.87 | 1.1 | 1.47 | 0.91 | 1.14 | 1.3 | 1.11 | 0.89 | 1.13 | 1.11 | 1.14 |
| 1.04 | 1.06 | 1.47 | 1.04 | 0.8 | 1.73 | 1.32 | 2.26 | 1.16 | 1.28 | 2.08 |
| 1.12 | 1.42 | 0.61 | 1.06 | 1.33 | 1.38 | 1.38 | 1.11 | 1.83 | 1.09 | 1.34 |
| 0.89 | 1.07 | 0.75 | 1.45 | 1.19 | 0.74 | 0.9 | 1.06 | 1 | 0.81 | 0.76 |
| 1.03 | 1.04 | 1.02 | 0.98 | 1.14 | 0.62 | 1.05 | 2.73 | 0.62 | 1 | 0.9 |
| 0.3 | 0.44 | 0.97 | 0.28 | 2.33 | 1.88 | 3.33 | 0.29 | 5.63 | 7.4 | 4.13 |
| 1.42 | 1.46 | 1.21 | 1.07 | 0.29 | 1.06 | 0.87 | 0.87 | 1.55 | 0.89 | 0.84 |
| 1.67 | 1.14 | 1.31 | 1.02 | 0.76 | 0.23 | 0.76 | 26.12 | 2.34 | 1.25 | 1.38 |

| 209341_s_at | 205225_at | 221334_s_at | 203628_at | 204849_at | 201124_at | 201576_s_at | 201289_at | 213844_at | 213139_at | 209239_at |
|---|---|---|---|---|---|---|---|---|---|---|
| 1.02 | 5.35 | 3.92 | 0.95 | 0.87 | 0.88 | 0.93 | 0.74 | 0.4 | 0.6 | 0.94 |
| 1.16 | 0.79 | 0.99 | 0.77 | 1.15 | 0.89 | 0.93 | 0.74 | 0.97 | 0.48 | 1.35 |
| 1.85 | 0.96 | 1.64 | 1.18 | 0.85 | 0.91 | 1.2 | 1.31 | 1.99 | 2.23 | 1.09 |
| 1.14 | 0.83 | 1.96 | 0.94 | 1.05 | 2.09 | 1.11 | 1.49 | 1.46 | 4.27 | 0.93 |
| 1.51 | 1.12 | 2.61 | 0.76 | 0.86 | 1.51 | 0.91 | 0.8 | 0.73 | 0.63 | 1.05 |
| 0.71 | 10.33 | 2.61 | 6.09 | 1.12 | 1.9 | 1.73 | 2.75 | 1.79 | 2.82 | 1.2 |
| 1.34 | 0.22 | 11.5 | 0.99 | 1.11 | 1.72 | 0.79 | 0.79 | 0.67 | 1.12 | 0.76 |
| 0.95 | 2.62 | 0.87 | 0.78 | 0.85 | 0.59 | 1.53 | 4.42 | 1.47 | 0.67 | 1.15 |
| 1 | 0.48 | 1.37 | 0.91 | 0.4 | 1.22 | 1.29 | 0.82 | 1.39 | 0.45 | 1.24 |
| 0.75 | 1.31 | 0.16 | 0.62 | 0.34 | 0.24 | 0.81 | 3.22 | 0.66 | 3.08 | 1.01 |
| 1.27 | 1.64 | 0.72 | 1.19 | 1 | 1.28 | 0.99 | 1.8 | 2.8 | 1.35 | 0.99 |
| 1.1 | 2.84 | 1.37 | 0.82 | 1 | 0.74 | 0.87 | 0.95 | 0.91 | 0.54 | 0.81 |
| 1.16 | 1.29 | 1.62 | 0.87 | 0.83 | 1.21 | 0.82 | 0.95 | 1.34 | 0.59 | 0.91 |
| 0.26 | 7.93 | 17.6 | 17.58 | 0.04 | 0.82 | 0.21 | 5.92 | 27.85 | 3.74 | 1.48 |
| 1.47 | 3.07 | 0.7 | 1.4 | 1.21 | 1.16 | 1.96 | 1.42 | 1.59 | 1.71 | 1.5 |
| 1.21 | 6.17 | 2.12 | 0.59 | 0.75 | 1.3 | 0.93 | 0.46 | 0.96 | 5.13 | 0.91 |
| 1.25 | 2.15 | 6.37 | 0.91 | 1.05 | 0.99 | 0.81 | 0.89 | 1.17 | 0.79 | 1.12 |
| 1.05 | 1.24 | 3.33 | 5.25 | 0.85 | 1.8 | 0.96 | 0.49 | 1.15 | 1.47 | 0.97 |
| 1.32 | 10.5 | 1.38 | 2.3 | 1.27 | 1.16 | 1.09 | 0.29 | 1.1 | 0.79 | 1.11 |
| 1.39 | 2.7 | 6.63 | 0.52 | 0.78 | 0.93 | 1.01 | 4.78 | 1.91 | 0.41 | 1.96 |
| 0.49 | 0.78 | 2.26 | 4.07 | 0.44 | 2.64 | 0.81 | 7.77 | 1.33 | 3.75 | 0.6 |
| 0.93 | 12.38 | 9 | 2.67 | 0.83 | 2.52 | 0.89 | 0.52 | 0.54 | 1.43 | 1.33 |
| 1.07 | 1.94 | 0.55 | 0.95 | 0.99 | 1 | 1.06 | 0.9 | 0.77 | 1.05 | 0.89 |
| 1.21 | 0.22 | 3.13 | 0.56 | 1.41 | 1.57 | 1.06 | 2.69 | 1.24 | 0.3 | 0.47 |
| 1.79 | 0.48 | 0.85 | 0.12 | 1.05 | 1.4 | 0.73 | 15.27 | 0.11 | 24.58 | 1.92 |
| 0.85 | 1.56 | 1.34 | 1.09 | 1.39 | 0.77 | 1.03 | 1.19 | 2.14 | 0.53 | 1.07 |
| 1.14 | 0.87 | 0.5 | 1.2 | 0.95 | 0.91 | 0.54 | 2.52 | 0.82 | 0.82 | 0.85 |
| 0.97 | 8.45 | 3.45 | 3.96 | 0.78 | 3.48 | 1.4 | 0.59 | 3.68 | 2.22 | 1.91 |
| 0.5 | 3.59 | 1.47 | 0.26 | 1.32 | 1.29 | 0.51 | 0.03 | 0.5 | 3.69 | 1.02 |
| 0.83 | 3.33 | 0.59 | 1.13 | 0.89 | 0.92 | 0.99 | 1.88 | 0.94 | 1.6 | 0.9 |

| | | | | | | | | | | |
|---|---|---|---|---|---|---|---|---|---|---|
| 0.69 | 0.62 | 0.92 | 0.61 | 0.74 | 0.77 | 0.7 | 1.61 | 0.34 | 1.79 | 0.85 |
| 3.19 | 2.42 | 1.84 | 8.87 | 4.2 | 1.05 | 3.97 | 6.69 | 23.15 | 9.91 | 1.03 |
| 1.26 | 1.55 | 1.29 | 1.1 | 1.12 | 1.01 | 0.99 | 1.17 | 1.55 | 2.37 | 1.1 |
| 0.76 | 0.76 | 1.44 | 0.54 | 1.43 | 1.58 | 0.95 | 0.48 | 0.51 | 0.39 | 0.9 |
| 2.42 | 7.31 | 0.24 | 2.3 | 0.35 | 1.92 | 1.15 | 6.03 | 2.03 | 0.35 | 0.74 |
| 0.78 | 1.59 | 2.44 | 0.24 | 0.79 | 1.15 | 0.58 | 7.47 | 1.24 | 0.83 | 1.26 |
| 0.93 | 0.82 | 2.04 | 0.66 | 0.91 | 0.68 | 0.54 | 0.03 | 3.87 | 1.32 | 1.72 |
| 0.78 | 1.17 | 2.16 | 0.4 | 0.8 | 1.41 | 0.98 | 1.06 | 0.94 | 0.9 | 0.85 |
| 0.89 | 0.68 | 1.42 | 1.09 | 1.31 | 1.93 | 1.15 | 0.9 | 0.67 | 0.83 | 0.69 |
| 1.88 | 0.13 | 1.57 | 0.62 | 1.79 | 1.87 | 0.66 | 1.44 | 0.5 | 31.56 | 0.92 |
| 1.12 | 1.25 | 0.8 | 1.2 | 0.81 | 0.65 | 0.94 | 0.87 | 0.85 | 0.63 | 0.77 |
| 0.95 | 1.56 | 2.8 | 0.97 | 0.9 | 15.67 | 1.12 | 1.03 | 0.31 | 1.14 | 0.92 |
| 1.41 | 0.77 | 1.51 | 0.84 | 0.73 | 0.9 | 1.2 | 1.79 | 0.76 | 0.61 | 1.23 |
| 1.49 | 0.98 | 3.83 | 0.66 | 0.2 | 1.97 | 0.85 | 0.69 | 0.59 | 1.13 | 1.77 |
| 1.22 | 1 | 1.39 | 0.77 | 0.72 | 0.98 | 1.12 | 1.67 | 0.78 | 0.69 | 1.22 |
| 1.59 | 2.13 | 2.47 | 1.07 | 1.37 | 1.14 | 1.12 | 3.18 | 1.05 | 4.09 | 1.09 |
| 1.14 | 1.13 | 0.5 | 1.1 | 1.16 | 1.25 | 1.06 | 1.22 | 1.14 | 1.06 | 0.71 |
| 1.1 | 0.37 | 2.15 | 0.48 | 0.63 | 0.85 | 0.94 | 2.75 | 0.79 | 2.72 | 1.1 |
| 1.06 | 4.38 | 3.46 | 1.13 | 1.11 | 0.98 | 0.9 | 1.04 | 1.05 | 1.13 | 0.99 |
| 0.89 | 9.33 | 1.82 | 0.65 | 0.67 | 1.87 | 0.98 | 1.08 | 0.54 | 1.79 | 1.13 |
| 0.81 | 0.98 | 2.25 | 1.23 | 1.02 | 1.15 | 0.94 | 1.08 | 1.12 | 0.97 | 0.67 |
| 1.06 | 1.02 | 0.61 | 0.96 | 1.14 | 0.8 | 0.8 | 0.99 | 1.37 | 0.68 | 1.01 |
| 0.7 | 1.84 | 0.96 | 0.96 | 1.41 | 0.72 | 1.08 | 0.93 | 2.78 | 1.86 | 1.02 |
| 1.13 | 3.13 | 1.46 | 1.17 | 0.91 | 0.86 | 3.08 | 1.14 | 2.83 | 7.72 | 1.07 |
| 0.66 | 1.51 | 0.96 | 0.44 | 1.05 | 0.46 | 1.32 | 0.61 | 0.19 | 0.88 | 1.06 |
| 1.16 | 0.61 | 0.22 | 0.65 | 1.06 | 0.8 | 1.11 | 0.79 | 1.42 | 0.52 | 1.36 |
| 1.15 | 5.33 | 2.37 | 0.46 | 0.32 | 0.64 | 0.84 | 7.6 | 0.43 | 0.46 | 1.05 |
| 2.16 | 1 | 1.43 | 1.51 | 1.01 | 1.79 | 1.08 | 4.66 | 1.46 | 0.01 | 0.65 |
| 1.43 | 1.31 | 0.88 | 1.42 | 1.13 | 1.73 | 1.23 | 0.73 | 0.85 | 0.83 | 0.85 |
| 1.22 | 0.81 | 0.76 | 0.7 | 0.55 | 0.63 | 1.02 | 1.61 | 1.21 | 0.45 | 1.6 |
| 0.92 | 7.08 | 0.45 | 1.04 | 0.79 | 0.92 | 1 | 0.89 | 1.67 | 1.67 | 1.06 |
| 1.61 | 0.37 | 0.53 | 2.23 | 1.37 | 1.53 | 1.6 | 1 | 0.82 | 2.36 | 0.7 |
| 1.02 | 2.94 | 2.51 | 0.95 | 1.13 | 10.63 | 1.12 | 3.74 | 1.23 | 2.03 | 0.81 |

| | | | | | | | | | | |
|---|---|---|---|---|---|---|---|---|---|---|
| 1.04 | 15.88 | 0.88 | 1.08 | 1.55 | 0.82 | 1.09 | 1.18 | 1.03 | 24.09 | 0.78 |
| 0.99 | 0.58 | 0.8 | 1.65 | 0.92 | 1.33 | 0.91 | 1.22 | 0.8 | 0.91 | 0.85 |
| 1 | 1.03 | 1.34 | 7.94 | 1.14 | 0.45 | 1.04 | 1.08 | 1.26 | 3.25 | 1.88 |
| 0.88 | 1.16 | 1.86 | 2.39 | 1.37 | 1.94 | 0.92 | 1.71 | 1.7 | 9.22 | 0.97 |
| 1.21 | 2.03 | 1.17 | 0.95 | 0.86 | 1 | 1.03 | 1.19 | 1 | 1.19 | 1.1 |
| 1.65 | 0.64 | 1.87 | 1.36 | 2.45 | 1.17 | 1.31 | 0.17 | 2.14 | 1.13 | 1.16 |
| 0.6 | 0.58 | 2.55 | 0.17 | 1.02 | 1.42 | 0.91 | 1.1 | 2.23 | 0.12 | 0.93 |
| 1.26 | 0.92 | 0.6 | 1.03 | 1.13 | 1.37 | 1.3 | 0.81 | 0.88 | 2.54 | 1.23 |
| 6.85 | 1.11 | 1.01 | 0.72 | 1.59 | 0.59 | 0.82 | 1.4 | 0.73 | 0.99 | 1.11 |
| 1.61 | 1.2 | 1.53 | 1.21 | 1.09 | 0.84 | 1.32 | 6.59 | 1.16 | 0.86 | 1.13 |
| 0.5 | 0.88 | 2.99 | 1.21 | 0.76 | 1.23 | 0.98 | 2.81 | 12.19 | 2.33 | 0.69 |
| 1.07 | 2.5 | 1.62 | 1 | 0.77 | 0.99 | 1.04 | 0.65 | 1.15 | 5.46 | 1.22 |
| 1.35 | 16 | 1.77 | 1.42 | 0.75 | 1.08 | 0.97 | 1.69 | 0.84 | 3.56 | 1.38 |
| 0.99 | 1.12 | 1.6 | 0.68 | 0.63 | 0.78 | 1.6 | 1.19 | 1.53 | 0.42 | 0.92 |
| 0.87 | 1 | 0.97 | 0.7 | 0.97 | 0.56 | 0.87 | 1.78 | 1.32 | 0.5 | 1.66 |
| 9.99 | 0.05 | 0.07 | 7.48 | 11.64 | 0.87 | 8.4 | 3.73 | 0.1 | 42.98 | 0.42 |
| 1.44 | 1.04 | 1.77 | 0.24 | 1.63 | 0.67 | 1.4 | 2.44 | 1.84 | 1 | 1.45 |
| 1.29 | 0.82 | 0.03 | 0.28 | 0.83 | 0.68 | 1.04 | 0.55 | 1.55 | 1.36 | 0.26 |
| 1.21 | 0.82 | 2.58 | 0.94 | 0.83 | 1.5 | 1.04 | 1.1 | 1.11 | 1.27 | 0.92 |
| 1.04 | 0.99 | 10.9 | 15.91 | 5.35 | 2.63 | 1.04 | 1.2 | 3.04 | 5.05 | 0.91 |
| 1.25 | 7.41 | 0.77 | 6.22 | 0.98 | 2.25 | 1.91 | 1.14 | 3.15 | 0.51 | 1.21 |
| 1.2 | 1.73 | 1.1 | 0.9 | 1.18 | 1.18 | 0.99 | 0.69 | 1.19 | 1.03 | 1.02 |
| 0.93 | 0.24 | 9.75 | 0.89 | 1.06 | 2.41 | 0.83 | 1.11 | 0.66 | 0.87 | 0.71 |
| 0.8 | 0.93 | 0.73 | 1.37 | 1.66 | 1.07 | 1.09 | 0.92 | 1.16 | 0.54 | 1.23 |
| 1.17 | 1.26 | 0.91 | 1.22 | 1.32 | 1.72 | 1.08 | 0.92 | 4.49 | 0.73 | 0.82 |
| 0.9 | 0.52 | 5.33 | 0.66 | 0.93 | 1.05 | 1.06 | 0.94 | 1.23 | 0.82 | 0.76 |
| 0.99 | 3.87 | 2.6 | 0.87 | 0.87 | 1.06 | 1.05 | 1.22 | 1.01 | 0.77 | 1.07 |
| 1 | 0.86 | 0.99 | 2.05 | 0.98 | 2.08 | 1.15 | 2.44 | 0.12 | 1.13 | 1.06 |
| 0.79 | 0.9 | 4.22 | 0.81 | 0.86 | 1.15 | 1.02 | 1.15 | 0.62 | 0.34 | 1.23 |
| 0.89 | 3.59 | 2.07 | 0.77 | 1.03 | 1.05 | 1.04 | 3.75 | 0.82 | 1.36 | 1.07 |
| 1.07 | 4.61 | 0.5 | 1.28 | 0.95 | 0.77 | 1.07 | 3.23 | 1.21 | 1.13 | 1.01 |
| 0.73 | 3.67 | 2.62 | 0.48 | 0.52 | 1.16 | 0.87 | 0.99 | 0.55 | 1.31 | 0.82 |
| 1.39 | 2.6 | 0.94 | 0.28 | 1.06 | 2.96 | 0.97 | 5.49 | 0.91 | 2.88 | 1.93 |

| | | | | | | | | | | |
|---|---|---|---|---|---|---|---|---|---|---|
| 1.31 | 1.39 | 1.25 | 2.59 | 1.03 | 1.31 | 0.99 | 1.45 | 2.45 | 0.66 | 1.31 |
| 0.83 | 0.47 | 1.39 | 7.25 | 1.05 | 2.56 | 0.99 | 2.12 | 1.04 | 1.22 | 0.86 |
| 0.75 | 0.77 | 3.64 | 1.39 | 1.1 | 0.95 | 0.92 | 2.08 | 1.27 | 2.66 | 0.9 |
| 4.71 | 0.94 | 0.77 | 0.85 | 1.36 | 1.38 | 0.8 | 3.28 | 0.78 | 1.83 | 1.05 |
| 1.72 | 1.31 | 1.77 | 2.45 | 2.68 | 1.35 | 1.29 | 0.15 | 1.16 | 1.75 | 1.48 |
| 0.5 | 0.41 | 0.69 | 0.6 | 1.05 | 0.91 | 1.34 | 0.59 | 0.45 | 3.24 | 0.93 |
| 1.47 | 2.38 | 2.57 | 0.69 | 0.95 | 0.88 | 0.88 | 1.34 | 0.79 | 1.45 | 1.44 |
| 0.94 | 0.66 | 1.24 | 1.19 | 1.25 | 0.79 | 1.03 | 1.72 | 1.31 | 1.16 | 0.67 |
| 0.57 | 0.65 | 3.35 | 0.86 | 1.14 | 1.06 | 1.05 | 3.57 | 0.94 | 1.29 | 0.95 |
| 1.01 | 2.38 | 0.93 | 1.75 | 1.05 | 0.89 | 1.29 | 3.09 | 4.29 | 4.37 | 1.29 |
| 0.91 | 1.54 | 4.3 | 1.08 | 0.86 | 0.79 | 1.15 | 1.06 | 0.99 | 0.68 | 0.96 |
| 1.14 | 1.23 | 5.83 | 0.85 | 0.93 | 1.04 | 1.1 | 0.93 | 1.07 | 18.48 | 1.09 |
| 0.73 | 1 | 1.19 | 0.54 | 0.79 | 0.39 | 0.79 | 0.96 | 1.02 | 0.96 | 1.48 |
| 0.87 | 0.72 | 1.16 | 0.52 | 1.32 | 1.39 | 0.96 | 1.02 | 0.74 | 0.68 | 1 |
| 1.26 | 1.67 | 1.18 | 1.25 | 1.18 | 3.04 | 1.23 | 0.97 | 0.58 | 1.81 | 1.02 |
| 1.24 | 1.46 | 2.01 | 0.99 | 1.17 | 2.76 | 0.92 | 1.57 | 4.46 | 2.83 | 1.03 |
| 0.92 | 1.16 | 0.74 | 2.48 | 1.15 | 1.58 | 0.93 | 0.56 | 0.86 | 2.35 | 1.08 |
| 1.19 | 1.02 | 1.42 | 3.23 | 1.33 | 1.87 | 0.96 | 1.6 | 2.37 | 9.33 | 1.08 |
| 1.2 | 5.51 | 0.98 | 0.93 | 1.23 | 1 | 1.2 | 1.13 | 0.95 | 0.53 | 2.32 |
| 0.7 | 1.35 | 1.73 | 0.66 | 1.19 | 1.61 | 1.08 | 0.95 | 1.02 | 1.32 | 0.77 |
| 0.98 | 0.55 | 0.42 | 0.88 | 0.83 | 0.83 | 0.83 | 1.07 | 0.93 | 1.26 | 1.33 |
| 0.71 | 0.26 | 2.87 | 1.15 | 1.15 | 0.78 | 1.12 | 1.23 | 1.05 | 0.74 | 1.14 |
| 1.1 | 1.11 | 1.26 | 0.92 | 1.12 | 1.02 | 1.08 | 1.35 | 0.99 | 0.79 | 1.08 |
| 1.86 | 0.85 | 6.42 | 0.36 | 0.89 | 1.44 | 0.94 | 8.69 | 0.91 | 0.39 | 1 |
| 0.88 | 0.91 | 0.78 | 0.44 | 0.78 | 0.67 | 0.92 | 4.66 | 0.84 | 7.96 | 0.87 |
| 1.08 | 4.75 | 0.72 | 0.42 | 1.35 | 0.96 | 0.84 | 1.77 | 0.7 | 1.92 | 1.28 |
| 1.29 | 0.97 | 0.59 | 0.89 | 1 | 1.31 | 0.97 | 1.05 | 0.9 | 1.21 | 1.02 |
| 1.33 | 0.89 | 0.57 | 1.86 | 1.42 | 2.98 | 1.03 | 0.21 | 0.88 | 1 | 1.01 |
| 0.79 | 1.65 | 0.42 | 0.52 | 0.76 | 1.45 | 0.78 | 2.31 | 0.81 | 0.68 | 1.14 |
| 0.82 | 1.46 | 0.78 | 0.83 | 0.91 | 0.97 | 0.93 | 6.54 | 0.67 | 1.9 | 1.02 |
| 0.84 | 0.84 | 4.11 | 0.84 | 0.98 | 2.1 | 0.97 | 3.02 | 0.92 | 0.65 | 0.88 |
| 0.99 | 0.58 | 0.8 | 1.65 | 0.92 | 1.33 | 0.91 | 1.22 | 0.8 | 0.91 | 0.85 |
| 1.09 | 1.33 | 0.28 | 0.61 | 1.01 | 0.64 | 1.05 | 1.04 | 0.74 | 0.87 | 1.65 |

| | | | | | | | | | | |
|---|---|---|---|---|---|---|---|---|---|---|
| 0.89 | 3.97 | 0.47 | 0.61 | 1.24 | 0.72 | 1.21 | 1.28 | 0.46 | 2.46 | 1.37 |
| 0.82 | 1.05 | 1.11 | 1.14 | 0.93 | 0.65 | 1 | 2.37 | 1.12 | 2.9 | 1.26 |
| 1.09 | 1.92 | 1.39 | 0.84 | 0.9 | 0.99 | 1.13 | 0.97 | 0.63 | 2.18 | 0.84 |
| 0.91 | 1.02 | 1.38 | 0.76 | 1.26 | 0.86 | 0.99 | 1.88 | 1.5 | 1.16 | 1.31 |
| 0.98 | 4.74 | 2.67 | 0.94 | 0.71 | 1.07 | 0.86 | 1.38 | 0.81 | 0.9 | 0.87 |
| 0.9 | 4 | 0.33 | 0.67 | 0.99 | 0.82 | 0.95 | 1.03 | 1.17 | 1 | 1 |
| 1 | 15.75 | 1.36 | 0.93 | 0.99 | 1.03 | 0.95 | 0.92 | 0.82 | 0.67 | 1.14 |
| 0.92 | 4.44 | 2.83 | 1.02 | 1.11 | 1.12 | 1.24 | 1.09 | 1.05 | 1.4 | 0.83 |
| 1.23 | 4.13 | 1.05 | 0.63 | 0.48 | 1.23 | 0.76 | 2.86 | 1.96 | 1.15 | 1.27 |
| 1.06 | 1.47 | 0.54 | 5.78 | 1.04 | 1.42 | 0.89 | 0.67 | 1.11 | 0.17 | 1.93 |
| 1.14 | 2.64 | 0.52 | 0.79 | 1.67 | 2.15 | 1 | 0.74 | 1.23 | 0.05 | 1.14 |
| 1.06 | 0.66 | 2.85 | 0.68 | 1.11 | 1.07 | 1.07 | 1.01 | 1.13 | 1.65 | 1.18 |
| 1.07 | 1.29 | 1.52 | 2.8 | 1.53 | 2.7 | 0.95 | 1.84 | 1.58 | 9.81 | 1.2 |
| 1.09 | 1.81 | 0.96 | 0.88 | 0.86 | 1.46 | 1.03 | 0.4 | 1.65 | 0.43 | 0.81 |
| 1.38 | 0.62 | 1.37 | 0.54 | 0.95 | 0.77 | 1.42 | 2.02 | 0.94 | 4.55 | 0.59 |
| 1.1 | 1.55 | 0.62 | 0.7 | 1.3 | 1.08 | 1.18 | 1.13 | 1.05 | 4.35 | 1.14 |
| 0.91 | 0.51 | 0.67 | 1.16 | 0.99 | 1.08 | 1.01 | 0.8 | 0.85 | 0.72 | 0.87 |
| 0.9 | 1.32 | 1.06 | 3.26 | 1 | 0.79 | 0.82 | 1.69 | 2.16 | 0.91 | 1.24 |
| 0.96 | 2.92 | 1.9 | 0.81 | 1.25 | 0.84 | 0.96 | 1.17 | 0.94 | 0.85 | 0.96 |
| 0.84 | 0.66 | 1.03 | 0.8 | 0.83 | 1.33 | 0.88 | 1.31 | 2.12 | 1.2 | 0.9 |
| 1.04 | 2.63 | 1.24 | 1.02 | 1.08 | 0.81 | 0.91 | 0.91 | 1.1 | 0.53 | 1.06 |
| 1.21 | 1.65 | 2.51 | 0.71 | 1.23 | 1.74 | 0.89 | 1.11 | 0.86 | 1.33 | 0.73 |
| 1.06 | 1.05 | 4.28 | 1.12 | 1.09 | 2.9 | 0.8 | 4.75 | 4.21 | 2.49 | 0.94 |
| 0.94 | 0.85 | 2.39 | 0.93 | 0.82 | 0.94 | 0.99 | 0.7 | 1.44 | 1.04 | 0.81 |
| 1.38 | 0.72 | 0.98 | 0.63 | 1.21 | 0.96 | 0.86 | 1.18 | 0.98 | 0.99 | 0.92 |
| 1.15 | 3.32 | 0.97 | 0.61 | 0.83 | 1.08 | 0.95 | 1.06 | 0.62 | 0.58 | 0.93 |
| 1.29 | 0.98 | 1.95 | 2.28 | 1.06 | 0.93 | 1.18 | 0.6 | 1.4 | 0.74 | 1.17 |
| 0.82 | 0.88 | 1.28 | 2.59 | 0.88 | 0.96 | 0.83 | 0.99 | 1.26 | 1.45 | 1.24 |
| 1.07 | 0.96 | 1.79 | 0.8 | 0.84 | 1.17 | 1.15 | 0.94 | 0.79 | 1.1 | 1 |
| 1.19 | 0.5 | 1.11 | 1.02 | 0.93 | 1.06 | 0.79 | 0.91 | 0.89 | 0.67 | 1.42 |
| 0.81 | 1.14 | 4.5 | 0.94 | 1.04 | 2.78 | 0.76 | 4.44 | 0.81 | 2.76 | 0.74 |
| 1.01 | 2.62 | 1.35 | 8.45 | 0.9 | 1.14 | 1.01 | 2.11 | 0.87 | 6.27 | 0.94 |
| 1.1 | 2.72 | 0.82 | 1.03 | 0.98 | 1.69 | 1 | 1.7 | 0.97 | 1.28 | 1.03 |

| | | | | | | | | | | |
|---|---|---|---|---|---|---|---|---|---|---|
| 6.69 | 1.44 | 1.2 | 0.71 | 1.83 | 0.65 | 0.97 | 3.54 | 0.84 | 0.77 | 0.72 |
| 0.82 | 0.9 | 3.45 | 1 | 1.15 | 2.37 | 0.86 | 4.74 | 0.81 | 2.1 | 0.83 |
| 1.01 | 0.94 | 1.03 | 0.94 | 0.98 | 1.03 | 0.98 | 1.23 | 0.93 | 0.89 | 1.04 |
| 1.19 | 1.04 | 1.46 | 0.9 | 1.43 | 1.23 | 0.96 | 3.39 | 1 | 0.9 | 0.94 |
| 0.51 | 0.24 | 2.28 | 1.11 | 1.21 | 1.12 | 0.83 | 0.91 | 0.88 | 2.33 | 1.06 |
| 0.99 | 1.52 | 1.08 | 0.89 | 0.95 | 0.94 | 0.93 | 0.87 | 0.9 | 1.18 | 0.95 |
| 0.7 | 0.72 | 1.2 | 0.55 | 0.76 | 1.16 | 0.69 | 2.07 | 0.24 | 2.62 | 0.82 |
| 0.93 | 0.73 | 0.67 | 1.03 | 1 | 0.64 | 1.2 | 1.01 | 1.11 | 1.02 | 0.96 |
| 0.94 | 3.48 | 2.44 | 0.83 | 1.05 | 0.93 | 0.94 | 1.42 | 1.11 | 2.01 | 1.18 |
| 0.68 | 0.51 | 0.66 | 0.91 | 0.76 | 0.62 | 0.67 | 2.6 | 0.36 | 3.52 | 0.84 |
| 0.73 | 2.1 | 5 | 0.55 | 1.35 | 0.54 | 0.85 | 0.92 | 0.61 | 2.3 | 1.74 |
| 0.83 | 1.26 | 1.21 | 2.45 | 0.83 | 0.35 | 0.77 | 1.43 | 0.94 | 0.81 | 0.93 |
| 0.92 | 2.67 | 1.7 | 0.75 | 0.91 | 0.98 | 0.8 | 1.36 | 0.82 | 1.95 | 0.8 |
| 0.91 | 0.08 | 0.51 | 0.81 | 1.19 | 1.07 | 1.13 | 2.21 | 1.73 | 3.18 | 1.07 |
| 0.92 | 0.96 | 0.86 | 0.61 | 1.4 | 0.79 | 1.07 | 2.25 | 1.57 | 0.86 | 1.31 |
| 1.06 | 0.97 | 0.5 | 0.65 | 0.73 | 0.79 | 1.19 | 1.58 | 1.35 | 0.96 | 1.37 |
| 1.19 | 4.34 | 1.56 | 0.55 | 0.95 | 4.44 | 0.97 | 1.99 | 1.9 | 1.71 | 1.13 |
| 0.78 | 2.91 | 2.24 | 0.97 | 1.02 | 0.87 | 1 | 0.98 | 2.55 | 0.67 | 1.09 |
| 1.21 | 0.73 | 1.52 | 0.32 | 1.38 | 2.82 | 1.01 | 0.79 | 1.01 | 1.38 | 0.98 |
| 1.12 | 1.54 | 0.65 | 0.56 | 1.27 | 1.08 | 0.83 | 1.07 | 1.69 | 1.02 | 1.17 |
| 1 | 1.1 | 1.86 | 0.87 | 1.05 | 1.74 | 0.89 | 0.78 | 1.2 | 0.99 | 0.81 |
| 0.57 | 1.71 | 1.03 | 0.62 | 0.66 | 1.06 | 0.63 | 0.05 | 2.37 | 0.05 | 1.46 |
| 0.96 | 0.97 | 0.68 | 1.29 | 1.24 | 0.5 | 1 | 7.14 | 0.56 | 3.21 | 1.05 |
| 3.2 | 0.91 | 1.81 | 0.65 | 1.84 | 0.6 | 0.88 | 4.25 | 1.07 | 0.88 | 0.89 |
| 1.13 | 0.97 | 0.72 | 1.02 | 0.95 | 1.79 | 1.21 | 1.06 | 1.13 | 0.94 | 1.15 |
| 1.07 | 1.13 | 1.87 | 2.09 | 0.99 | 0.71 | 0.91 | 1.75 | 0.06 | 34.58 | 0.65 |
| 0.87 | 1.62 | 2.79 | 0.96 | 1.07 | 1.25 | 1.26 | 0.95 | 0.93 | 1.33 | 1.42 |
| 0.95 | 1.07 | 0.56 | 0.93 | 1.03 | 0.98 | 1.07 | 0.89 | 1.16 | 2.91 | 0.97 |
| 1.12 | 1.59 | 0.83 | 0.46 | 1.09 | 2.78 | 1.03 | 1.3 | 1.03 | 1.25 | 0.9 |
| 0.84 | 1.44 | 3.98 | 1.08 | 1.14 | 0.8 | 0.99 | 1.27 | 0.83 | 3.69 | 0.97 |
| 1.16 | 1.54 | 0.55 | 1.08 | 0.91 | 0.89 | 1.02 | 1 | 1.68 | 5.28 | 1.19 |
| 0.84 | 0.93 | 5 | 0.97 | 0.81 | 0.85 | 0.87 | 1.33 | 0.87 | 0.97 | 1.04 |
| 1 | 1.43 | 1.25 | 1.46 | 0.89 | 0.55 | 1.03 | 0.55 | 1.88 | 1.78 | 0.82 |

| | | | | | | | | | | |
|---|---|---|---|---|---|---|---|---|---|---|
| 1.18 | 1 | 1.21 | 1.46 | 1.05 | 1.37 | 1.06 | 0.68 | 1.18 | 1.32 | 1.38 |
| 0.94 | 1.25 | 1.17 | 0.49 | 1.07 | 0.96 | 0.97 | 0.94 | 1.42 | 0.31 | 1.61 |
| 0.83 | 2.5 | 0.77 | 0.77 | 0.85 | 0.91 | 1.15 | 0.93 | 0.7 | 1.42 | 0.92 |
| 0.62 | 0.19 | 1.11 | 0.64 | 0.87 | 0.98 | 0.75 | 1.05 | 0.13 | 0.89 | 0.88 |
| 0.93 | 2.41 | 1.37 | 2.58 | 0.73 | 1.92 | 0.69 | 1.3 | 3 | 1.29 | 1.13 |
| 0.94 | 1.97 | 0.77 | 1.02 | 1.08 | 1.02 | 0.92 | 1.05 | 3.43 | 1.15 | 0.94 |
| 0.99 | 1.45 | 1.07 | 0.32 | 1.03 | 0.91 | 1.02 | 5.9 | 0.51 | 0.88 | 0.85 |
| 0.8 | 1.12 | 4.43 | 0.55 | 0.96 | 1.15 | 1.16 | 0.74 | 1.08 | 1.1 | 1.06 |
| 3.39 | 1.34 | 1.91 | 0.88 | 2 | 0.61 | 0.7 | 2.61 | 0.42 | 1.8 | 1.06 |
| 0.94 | 7 | 4.76 | 0.76 | 0.71 | 2.01 | 1.03 | 0.84 | 1.66 | 0.77 | 1 |
| 0.77 | 0.33 | 6.33 | 0.69 | 0.95 | 0.81 | 1.21 | 0.89 | 0.92 | 0.79 | 1.17 |
| 0.83 | 2.69 | 3.21 | 0.91 | 1.04 | 1.21 | 1.31 | 0.42 | 0.93 | 0.79 | 0.59 |
| 0.76 | 1.19 | 3.5 | 1.02 | 0.75 | 0.6 | 1.08 | 1.54 | 1.21 | 1.55 | 1.25 |
| 0.96 | 0.5 | 3.33 | 0.79 | 1.08 | 3.42 | 0.95 | 0.51 | 1.03 | 1.78 | 1.12 |
| 0.91 | 5.17 | 2.64 | 1.11 | 0.69 | 1.01 | 1.13 | 1.22 | 1.5 | 0.99 | 1.38 |
| 0.67 | 1.59 | 1.18 | 1.03 | 0.71 | 1 | 1.19 | 0.66 | 1.13 | 0.94 | 0.73 |
| 0.74 | 0.75 | 0.59 | 0.85 | 0.95 | 0.85 | 0.85 | 1.03 | 2.17 | 1.27 | 0.85 |
| 0.84 | 3.4 | 1.15 | 1.24 | 0.88 | 1.26 | 0.92 | 2.94 | 0.53 | 0.33 | 0.85 |
| 2.21 | 1.05 | 1.29 | 2.33 | 1.09 | 2.4 | 0.97 | 2.77 | 9.83 | 2.77 | 0.97 |
| 0.76 | 0.79 | 0.81 | 0.82 | 1.17 | 0.74 | 0.78 | 8.69 | 2.97 | 1.08 | 0.99 |
| 1.33 | 6.9 | 1.48 | 1 | 1.08 | 1.28 | 0.9 | 1.38 | 2.9 | 3.85 | 1.02 |
| 0.68 | 0.52 | 0.65 | 0.79 | 0.74 | 0.5 | 0.69 | 1.88 | 0.4 | 2.24 | 0.86 |
| 0.93 | 0.63 | 1.64 | 0.53 | 1.26 | 11.48 | 0.89 | 5.14 | 0.54 | 0.26 | 1.13 |
| 0.84 | 1.07 | 1.21 | 1.2 | 1.13 | 1.21 | 0.8 | 1.73 | 0.23 | 2.03 | 1.17 |
| 1.24 | 0.78 | 1.23 | 1.16 | 1.12 | 0.77 | 1.07 | 0.85 | 1.03 | 0.98 | 0.97 |
| 1.33 | 0.73 | 1.08 | 0.53 | 0.99 | 0.78 | 0.68 | 1.28 | 0.83 | 1.86 | 0.86 |
| 0.82 | 0.64 | 0.65 | 0.81 | 0.71 | 0.44 | 0.96 | 1.22 | 2.16 | 5.62 | 1.03 |
| 1.14 | 0.79 | 0.43 | 0.31 | 1.28 | 1.86 | 1.32 | 1.24 | 1.15 | 0.53 | 1.56 |
| 0.75 | 1.47 | 2.4 | 1.19 | 0.71 | 0.77 | 1.34 | 3.46 | 0.91 | 1.26 | 1.18 |
| 1.99 | 0.37 | 1.12 | 5.73 | 2.45 | 0.69 | 1.21 | 0.43 | 1.6 | 3.93 | 0.77 |
| 1.27 | 9.33 | 1.18 | 0.74 | 0.84 | 1.18 | 1.13 | 2.73 | 0.82 | 1.4 | 0.84 |
| 0.94 | 0.88 | 1.2 | 0.87 | 0.91 | 1.84 | 0.88 | 1.11 | 1.21 | 2.48 | 0.8 |
| 1.4 | 5.79 | 0.64 | 5.22 | 1.14 | 1.07 | 0.99 | 1.96 | 2.13 | 0.65 | 1.51 |

| | | | | | | | | | | |
|---|---|---|---|---|---|---|---|---|---|---|
| 1.07 | 19.75 | 1.08 | 1.05 | 0.85 | 1.02 | 0.89 | 0.91 | 0.75 | 1.11 | 0.94 |
| 1.06 | 1.26 | 0.45 | 0.69 | 0.88 | 0.97 | 0.97 | 1.02 | 1.26 | 1.01 | 0.99 |
| 2.45 | 0.91 | 7.14 | 2.09 | 1.51 | 0.94 | 0.47 | 0.87 | 0.59 | 1.75 | 0.69 |
| 0.59 | 3.17 | 2.98 | 0.81 | 0.66 | 1.52 | 1.25 | 0.91 | 0.51 | 0.64 | 1.19 |
| 0.73 | 1.53 | 1.68 | 0.71 | 0.86 | 0.94 | 1.05 | 1.2 | 1.61 | 0.91 | 0.83 |
| 1.28 | 0.86 | 1.06 | 0.7 | 1.1 | 1.92 | 0.98 | 0.77 | 0.69 | 3.73 | 1.08 |
| 1.25 | 3.72 | 2.17 | 1.54 | 0.88 | 0.86 | 0.99 | 1.79 | 0.96 | 1.69 | 0.96 |
| 1.07 | 1.61 | 1.99 | 0.97 | 0.88 | 0.66 | 0.84 | 0.56 | 1.08 | 0.45 | 0.97 |
| 1.24 | 1.1 | 0.88 | 2.3 | 1.63 | 1.23 | 1.16 | 0.63 | 1.06 | 2.24 | 0.86 |
| 1.08 | 1 | 1.26 | 1.63 | 0.88 | 1.7 | 1.18 | 1.54 | 0.89 | 0.97 | 1.72 |
| 1.25 | 5.22 | 3.37 | 1.14 | 1.02 | 1.48 | 0.94 | 0.92 | 0.34 | 0.39 | 1.05 |
| 1.69 | 0.05 | 7.24 | 2.82 | 0.45 | 0.08 | 0.04 | 76.03 | 10.97 | 25.94 | 1.79 |
| 1.35 | 2.28 | 0.78 | 0.74 | 0.96 | 0.57 | 0.9 | 1.74 | 1.2 | 1.22 | 1.22 |
| 1.16 | 0.42 | 1.58 | 2.66 | 0.77 | 1.7 | 1.26 | 1.71 | 1.67 | 0.32 | 1.13 |

| 208815_x_at | 203451_at | 205215_at | 202670_at | 205932_s_at | 217555_at | 218902_at | 204772_s_at | 209341_s_at | 204020_at | 208986_at |
|---|---|---|---|---|---|---|---|---|---|---|
| 0.96 | 1.04 | 1.39 | 1.07 | 1.23 | 1.35 | 1.27 | 0.94 | 1.02 | 0.89 | 0.85 |
| 1 | 1.1 | 1.47 | 1.02 | 1.14 | 1.04 | 1.02 | 0.83 | 1.16 | 1.12 | 1.02 |
| 1.39 | 0.78 | 3.06 | 0.93 | 0.6 | 0.68 | 1.77 | 1.41 | 1.85 | 1.58 | 2.54 |
| 1.15 | 1.31 | 1.81 | 1.06 | 1.2 | 2.15 | 1.36 | 1.01 | 1.14 | 0.8 | 0.96 |
| 0.92 | 0.7 | 0.9 | 1.24 | 1.09 | 0.93 | 1.27 | 0.97 | 1.51 | 0.97 | 0.86 |
| 1.08 | 1.53 | 1.54 | 1.02 | 5.53 | 1.7 | 0.91 | 1.07 | 0.71 | 0.98 | 0.93 |
| 1.08 | 1.04 | 1.68 | 0.89 | 1.34 | 1.05 | 0.34 | 1.13 | 1.34 | 1.24 | 1.33 |
| 1.14 | 1.05 | 0.91 | 1.39 | 1.01 | 1.06 | 1.05 | 1.26 | 0.95 | 0.99 | 0.92 |
| 1.16 | 1.07 | 0.82 | 1.32 | 1.51 | 1.13 | 0.77 | 1.02 | 1 | 1.11 | 0.98 |
| 0.9 | 1.26 | 0.96 | 0.98 | 3.52 | 1.17 | 1.23 | 1.11 | 0.75 | 0.77 | 0.64 |
| 0.99 | 0.66 | 1.29 | 0.98 | 1.03 | 0.83 | 1.3 | 2.39 | 1.27 | 1.05 | 0.98 |
| 1.18 | 0.99 | 1.05 | 1.24 | 0.85 | 1 | 0.9 | 1.13 | 1.1 | 0.88 | 1.01 |
| 1.21 | 1.46 | 1.16 | 0.92 | 1.09 | 1.55 | 0.96 | 0.99 | 1.16 | 1.07 | 1.29 |
| 0.93 | 2.07 | 0.81 | 0.63 | 0.01 | 46.45 | 0.65 | 0.36 | 0.26 | 2.66 | 6 |
| 0.99 | 0.9 | 0.59 | 1.3 | 1.54 | 0.75 | 0.72 | 1.51 | 1.47 | 1.37 | 1.18 |
| 0.89 | 1.35 | 1.36 | 0.8 | 1.51 | 2.9 | 0.87 | 0.86 | 1.21 | 1 | 1.77 |
| 1.16 | 1.4 | 0.96 | 1.19 | 1.24 | 1.7 | 1.15 | 1.18 | 1.25 | 1.09 | 0.85 |
| 0.86 | 1.98 | 1.44 | 1.33 | 6 | 0.92 | 1.52 | 0.7 | 1.05 | 1.22 | 1.38 |
| 1.11 | 1.37 | 1.25 | 0.94 | 0.7 | 1.39 | 6.36 | 1.3 | 1.32 | 1.12 | 1.15 |
| 1.88 | 2.45 | 6.52 | 0.46 | 0.64 | 11.8 | 0.57 | 1.17 | 1.39 | 0.84 | 0.66 |
| 1.1 | 1.96 | 2.98 | 0.63 | 6.53 | 0.84 | 1.06 | 0.43 | 0.49 | 1.49 | 0.83 |
| 0.73 | 1.99 | 2.03 | 1.94 | 0.95 | 1.77 | 1.18 | 0.74 | 0.93 | 0.89 | 0.73 |
| 0.83 | 0.88 | 1.6 | 0.83 | 1.28 | 0.87 | 0.75 | 1.11 | 1.07 | 0.9 | 1.15 |
| 0.9 | 0.59 | 1.1 | 0.54 | 0.71 | 0.53 | 0.28 | 0.69 | 1.21 | 0.78 | 0.99 |
| 1.42 | 0.51 | 1.03 | 0.55 | 0.14 | 0.38 | 0.52 | 0.23 | 1.79 | 1.04 | 0.91 |
| 0.95 | 0.72 | 0.85 | 0.95 | 0.92 | 0.76 | 1.86 | 0.52 | 0.85 | 0.47 | 0.71 |
| 1.19 | 1.66 | 1.35 | 1.01 | 0.82 | 1.62 | 0.88 | 0.84 | 1.14 | 0.71 | 0.84 |
| 0.88 | 0.91 | 1.53 | 1.05 | 0.84 | 1.1 | 0.59 | 0.96 | 0.97 | 0.74 | 0.78 |
| 0.57 | 1.1 | 1.06 | 0.95 | 2.74 | 1.41 | 1.47 | 1.21 | 0.5 | 1.01 | 1.03 |
| 0.95 | 1.05 | 1.19 | 1.11 | 1.19 | 0.93 | 1.27 | 1.02 | 0.83 | 1.14 | 0.83 |

| | | | | | | | | | | |
|---|---|---|---|---|---|---|---|---|---|---|
| 1.65 | 1.29 | 0.8 | 1.29 | 3.69 | 0.39 | 0.62 | 0.87 | 0.69 | 1 | 0.94 |
| 1.3 | 2.79 | 1.02 | 1.66 | 8.14 | 1.82 | 0.83 | 2.68 | 3.19 | 2.51 | 1.66 |
| 0.95 | 1.58 | 1.71 | 1.01 | 1.18 | 1.11 | 1.12 | 1.18 | 1.26 | 1.11 | 1.07 |
| 1.19 | 0.7 | 1.58 | 0.96 | 0.72 | 1.18 | 0.4 | 0.9 | 0.76 | 0.56 | 0.79 |
| 1.43 | 1.82 | 0.23 | 0.94 | 0.73 | 1.16 | 0.47 | 6.48 | 2.42 | 1.88 | 1.42 |
| 0.86 | 3.9 | 3.18 | 1.06 | 3.14 | 0.82 | 5.39 | 0.88 | 0.78 | 0.58 | 1.21 |
| 2.11 | 2.09 | 1.46 | 1.42 | 1.98 | 4.28 | 2.09 | 1.14 | 0.93 | 0.57 | 0.62 |
| 1.01 | 0.76 | 0.9 | 0.85 | 1.29 | 1.19 | 1 | 0.8 | 0.78 | 0.77 | 0.95 |
| 1.08 | 0.42 | 1.4 | 0.89 | 3.54 | 1.51 | 0.71 | 0.45 | 0.89 | 1.88 | 1.25 |
| 0.82 | 1.49 | 1.15 | 0.95 | 2.54 | 0.66 | 0.4 | 1.14 | 1.88 | 1 | 1.14 |
| 1 | 1.23 | 1.46 | 1.01 | 1.34 | 1.27 | 0.99 | 1.55 | 1.12 | 1.03 | 0.95 |
| 1.08 | 1.19 | 1.01 | 1.18 | 1 | 4.65 | 1.66 | 1.35 | 0.95 | 0.89 | 0.9 |
| 1.1 | 1.15 | 1.18 | 1 | 1.91 | 0.58 | 0.76 | 1.08 | 1.41 | 1.08 | 1.36 |
| 1.03 | 0.77 | 0.29 | 0.52 | 1.61 | 0.66 | 1.64 | 0.55 | 1.49 | 1.39 | 1.43 |
| 1.1 | 1.11 | 1.03 | 0.96 | 1.42 | 0.64 | 0.86 | 1.29 | 1.22 | 0.97 | 1.29 |
| 1.11 | 0.82 | 1.21 | 1.19 | 1.13 | 2.23 | 1.06 | 0.97 | 1.59 | 1.06 | 1.29 |
| 0.97 | 1.02 | 1.09 | 1.02 | 0.88 | 1.78 | 0.86 | 1.09 | 1.14 | 1.09 | 1.11 |
| 0.92 | 0.76 | 0.91 | 1 | 0.85 | 1.18 | 0.85 | 0.91 | 1.1 | 0.64 | 1.21 |
| 1 | 1.01 | 0.72 | 0.95 | 0.94 | 1.02 | 1.2 | 1.09 | 1.06 | 1 | 1.05 |
| 0.99 | 1.61 | 0.72 | 1.53 | 1.41 | 0.8 | 1.29 | 0.69 | 0.89 | 1.2 | 1.3 |
| 0.98 | 0.87 | 0.79 | 0.74 | 0.95 | 1.07 | 0.69 | 0.88 | 0.81 | 0.97 | 1.07 |
| 1.69 | 1.29 | 0.82 | 1.04 | 0.78 | 0.81 | 0.86 | 0.98 | 1.06 | 1.24 | 0.82 |
| 1 | 0.82 | 1.32 | 0.91 | 0.55 | 0.46 | 0.82 | 0.79 | 0.7 | 0.8 | 1.06 |
| 0.78 | 1.3 | 1.13 | 0.94 | 2.01 | 1.14 | 1.02 | 0.93 | 1.13 | 1.25 | 1.09 |
| 1.4 | 0.92 | 1.02 | 1.41 | 2.46 | 0.68 | 1.67 | 0.73 | 0.66 | 0.79 | 0.57 |
| 1.36 | 0.73 | 2.09 | 1.23 | 1.35 | 2.76 | 1.01 | 1.49 | 1.16 | 0.85 | 0.94 |
| 0.84 | 0.92 | 0.58 | 2.39 | 4.78 | 0.86 | 1.08 | 0.42 | 1.15 | 0.7 | 0.31 |
| 0.95 | 1.24 | 1.05 | 0.78 | 0.59 | 1.73 | 0.88 | 1.04 | 2.16 | 1 | 1.23 |
| 0.84 | 0.9 | 0.98 | 1 | 1.21 | 0.85 | 1.52 | 0.96 | 1.43 | 0.91 | 1.02 |
| 1.21 | 1.39 | 1.15 | 1.02 | 2.16 | 0.68 | 0.86 | 1.2 | 1.22 | 0.99 | 1.25 |
| 0.81 | 1.03 | 0.96 | 1.23 | 3.18 | 2 | 0.61 | 0.73 | 0.92 | 1.49 | 0.91 |
| 0.7 | 2.11 | 1.03 | 0.72 | 0.63 | 1.39 | 1.39 | 1.21 | 1.61 | 1.32 | 0.93 |
| 0.89 | 1.28 | 1.01 | 0.92 | 2.35 | 0.76 | 1.53 | 0.87 | 1.02 | 0.94 | 0.95 |

| | | | | | | | | | | |
|---|---|---|---|---|---|---|---|---|---|---|
| 0.86 | 1.17 | 0.91 | 1.08 | 0.65 | 1.62 | 0.72 | 0.71 | 1.04 | 1.09 | 0.99 |
| 1.12 | 1.63 | 0.82 | 0.95 | 7.72 | 1.05 | 1.81 | 1.22 | 0.99 | 1.09 | 1.92 |
| 0.97 | 0.9 | 0.9 | 1.32 | 1.49 | 0.96 | 0.74 | 0.79 | 1 | 1.21 | 1.27 |
| 0.99 | 0.91 | 1.94 | 1.13 | 1.17 | 1.94 | 1.47 | 1.01 | 0.88 | 1.29 | 1.18 |
| 0.97 | 1.34 | 0.99 | 1.06 | 0.66 | 0.7 | 0.99 | 1.12 | 1.21 | 1.27 | 1.08 |
| 0.88 | 0.58 | 2.58 | 0.64 | 1.13 | 2.07 | 1.69 | 1.01 | 1.65 | 2.5 | 0.65 |
| 1.2 | 1.07 | 0.45 | 0.87 | 1.07 | 1.15 | 1.12 | 1.5 | 0.6 | 1.69 | 0.43 |
| 0.84 | 1.17 | 1.12 | 1.1 | 0.65 | 0.65 | 0.94 | 0.94 | 1.26 | 0.92 | 0.77 |
| 1.05 | 1.53 | 9.29 | 1.47 | 1.18 | 1.26 | 0.75 | 1.47 | 6.85 | 0.79 | 0.43 |
| 1.31 | 1.16 | 2.07 | 0.82 | 1.09 | 1.2 | 1.27 | 1.11 | 1.61 | 1.3 | 1.56 |
| 0.94 | 1.9 | 0.58 | 1.53 | 2.29 | 1.33 | 1.56 | 0.85 | 0.5 | 1.55 | 1.21 |
| 1.12 | 0.76 | 1.68 | 1.13 | 1.24 | 1.76 | 1.36 | 1.01 | 1.07 | 1.01 | 0.84 |
| 0.91 | 0.97 | 1.34 | 1.16 | 1.32 | 0.62 | 1.34 | 0.97 | 1.35 | 0.84 | 1.03 |
| 0.95 | 1.23 | 0.9 | 0.72 | 0.98 | 0.94 | 1.24 | 0.78 | 0.99 | 1.39 | 0.73 |
| 1.32 | 1.31 | 1.22 | 1.06 | 0.74 | 0.92 | 0.91 | 1.06 | 0.87 | 0.83 | 1 |
| 1.99 | 0.33 | 5.16 | 5.66 | 8.98 | 0.28 | 0.3 | 24.74 | 9.99 | 0.03 | 0.08 |
| 0.89 | 0.78 | 1.83 | 0.76 | 1.02 | 1.43 | 1.04 | 0.88 | 1.44 | 1.44 | 0.68 |
| 1.13 | 1.3 | 1.21 | 1.05 | 1.73 | 0.8 | 0.89 | 0.97 | 1.29 | 0.87 | 1.87 |
| 0.97 | 1.27 | 1.05 | 1.02 | 0.9 | 0.97 | 0.79 | 0.74 | 1.21 | 1.17 | 1.22 |
| 1.04 | 1.43 | 3.26 | 1.13 | 6.68 | 1.58 | 2.04 | 1.44 | 1.04 | 0.82 | 1.01 |
| 0.71 | 3.48 | 0.67 | 2.05 | 10.51 | 0.28 | 1.71 | 0.59 | 1.25 | 0.48 | 0.97 |
| 0.9 | 0.72 | 1.1 | 1 | 1.73 | 1.07 | 0.94 | 1.11 | 1.2 | 1.12 | 0.94 |
| 1.17 | 1.27 | 1.29 | 0.88 | 0.98 | 1.13 | 0.39 | 1.16 | 0.93 | 1.14 | 1.16 |
| 0.68 | 1.07 | 0.86 | 1.62 | 2.26 | 0.98 | 0.73 | 1.91 | 0.8 | 2.14 | 1.04 |
| 0.9 | 1.1 | 1.03 | 1.11 | 0.78 | 1.6 | 1.17 | 0.87 | 1.17 | 1.82 | 1 |
| 1.02 | 0.69 | 1.29 | 1.1 | 1.09 | 0.95 | 0.67 | 0.85 | 0.9 | 0.94 | 0.95 |
| 0.9 | 0.92 | 0.9 | 1.13 | 1.08 | 0.95 | 1.1 | 0.83 | 0.99 | 0.71 | 0.74 |
| 1.14 | 0.95 | 1 | 1.05 | 1.73 | 1.1 | 1.51 | 1.07 | 1 | 1.01 | 1.07 |
| 1.07 | 1.04 | 1.27 | 0.89 | 1.13 | 1.24 | 0.98 | 0.94 | 0.79 | 1.12 | 1.02 |
| 0.93 | 1.12 | 1.02 | 0.95 | 0.95 | 0.79 | 1.05 | 1.06 | 0.89 | 0.94 | 0.95 |
| 0.9 | 1.1 | 1.17 | 1.17 | 1.18 | 1.08 | 1.28 | 1.12 | 1.07 | 0.94 | 1.01 |
| 0.99 | 0.76 | 0.88 | 1.77 | 1.31 | 1.13 | 0.79 | 1 | 0.73 | 0.96 | 0.84 |
| 1.04 | 1.02 | 1.02 | 1.02 | 0.99 | 0.7 | 1.37 | 0.82 | 1.39 | 1.03 | 1.13 |

| | | | | | | | | | | |
|---|---|---|---|---|---|---|---|---|---|---|
| 1.2 | 1.18 | 1.41 | 0.97 | 1.6 | 2.04 | 1 | 0.82 | 1.31 | 1.16 | 0.88 |
| 1.18 | 1.16 | 1 | 1.01 | 0.85 | 0.68 | 1.17 | 0.8 | 0.83 | 0.96 | 1.26 |
| 0.81 | 1.05 | 1.22 | 1.16 | 1.23 | 0.55 | 1.18 | 0.95 | 0.75 | 1.21 | 1.01 |
| 0.86 | 2.25 | 1.33 | 1.03 | 0.52 | 1.62 | 1.3 | 1.37 | 4.71 | 0.67 | 1.01 |
| 1.11 | 0.41 | 2.07 | 1 | 1.05 | 2.28 | 0.86 | 1.03 | 1.72 | 1.49 | 1.04 |
| 1.54 | 1.24 | 1.78 | 0.62 | 0.71 | 0.3 | 0.65 | 0.74 | 0.5 | 0.68 | 0.89 |
| 1.05 | 1.26 | 1.22 | 1.27 | 1.64 | 0.94 | 0.81 | 1.07 | 1.47 | 0.94 | 0.93 |
| 1.31 | 1.05 | 1.35 | 0.96 | 1.15 | 0.95 | 0.87 | 0.86 | 0.94 | 0.85 | 1.21 |
| 1.56 | 1.18 | 1.34 | 0.97 | 1.83 | 1.44 | 0.86 | 0.94 | 0.57 | 0.64 | 1.33 |
| 1.19 | 1.24 | 1.51 | 1.01 | 1.24 | 0.79 | 0.97 | 0.99 | 1.01 | 1.11 | 0.93 |
| 1.28 | 0.8 | 1.71 | 1.25 | 1.22 | 1.92 | 1.06 | 1.02 | 0.91 | 0.87 | 0.98 |
| 0.99 | 1.28 | 1.24 | 1.14 | 0.94 | 2.07 | 1.31 | 0.87 | 1.14 | 1.1 | 1.19 |
| 1.32 | 0.67 | 1.28 | 1.39 | 1.46 | 1.26 | 1.78 | 1.39 | 0.73 | 0.93 | 0.75 |
| 1.25 | 0.83 | 1.03 | 1.26 | 0.78 | 0.93 | 0.57 | 1 | 0.87 | 0.92 | 0.98 |
| 0.96 | 1.15 | 0.76 | 0.94 | 1.26 | 1.05 | 0.99 | 1.05 | 1.26 | 1.06 | 1.25 |
| 1 | 2.37 | 1.54 | 1.07 | 3.23 | 1.26 | 1.79 | 0.99 | 1.24 | 1.22 | 1.15 |
| 0.92 | 0.94 | 0.71 | 1.07 | 0.8 | 0.9 | 1.24 | 0.76 | 0.92 | 1.05 | 0.98 |
| 1.15 | 1.08 | 1.43 | 1.29 | 1.25 | 1.18 | 1.76 | 1.21 | 1.19 | 1.11 | 1.06 |
| 1.08 | 1.1 | 0.94 | 1.07 | 0.97 | 0.62 | 1.13 | 0.95 | 1.2 | 0.78 | 0.71 |
| 0.89 | 0.93 | 1 | 1.24 | 1.08 | 1.71 | 1.47 | 1.6 | 0.7 | 1.19 | 0.84 |
| 1.02 | 1.53 | 0.97 | 0.84 | 1.68 | 1.39 | 1.03 | 1.03 | 0.98 | 1.66 | 1.19 |
| 1.02 | 1.03 | 1.32 | 1.16 | 1.28 | 0.91 | 1.18 | 1.07 | 0.71 | 1.18 | 1.02 |
| 0.93 | 0.99 | 0.46 | 0.91 | 0.92 | 1.65 | 1.13 | 0.94 | 1.1 | 0.97 | 1.12 |
| 0.76 | 1.17 | 0.62 | 0.79 | 0.58 | 0.99 | 2.95 | 1.05 | 1.86 | 1.63 | 1.25 |
| 1.15 | 0.76 | 0.47 | 0.71 | 0.8 | 1.25 | 1.7 | 1.05 | 0.88 | 0.95 | 1.14 |
| 1.25 | 0.46 | 0.57 | 1.12 | 0.87 | 1.24 | 0.54 | 0.91 | 1.08 | 0.47 | 0.97 |
| 0.93 | 0.87 | 1.16 | 0.93 | 0.82 | 1 | 1.01 | 0.92 | 1.29 | 0.91 | 1.07 |
| 0.6 | 0.81 | 0.76 | 0.85 | 1.23 | 1.99 | 1.5 | 0.86 | 1.33 | 0.88 | 1.49 |
| 1.3 | 0.81 | 0.93 | 1.7 | 1.42 | 0.6 | 0.51 | 0.89 | 0.79 | 0.82 | 0.76 |
| 1.04 | 0.76 | 1.22 | 0.86 | 1.2 | 1.39 | 1.12 | 1.03 | 0.82 | 0.93 | 0.97 |
| 0.79 | 0.91 | 0.76 | 0.97 | 4.24 | 0.77 | 1.12 | 0.78 | 0.84 | 0.92 | 0.67 |
| 1.12 | 1.63 | 0.82 | 0.95 | 7.72 | 1.05 | 1.81 | 1.22 | 0.99 | 1.09 | 1.92 |
| 1.02 | 1.35 | 1 | 1.19 | 1.12 | 1.41 | 0.89 | 1.27 | 1.09 | 1.1 | 1.14 |

| | | | | | | | | | | |
|---|---|---|---|---|---|---|---|---|---|---|
| 0.99 | 1.01 | 0.75 | 1.6 | 1.58 | 1.71 | 0.98 | 1.45 | 0.89 | 1.3 | 1.18 |
| 1.03 | 1.05 | 1.02 | 0.97 | 0.99 | 1.08 | 0.95 | 1.19 | 0.82 | 0.81 | 1.1 |
| 1.2 | 1.06 | 1.04 | 0.94 | 1.73 | 0.34 | 1.23 | 0.66 | 1.09 | 1.04 | 0.99 |
| 1.14 | 0.81 | 1.33 | 0.94 | 1.04 | 1.12 | 1.23 | 1 | 0.91 | 1 | 1.13 |
| 1.04 | 1.03 | 0.83 | 1.01 | 0.67 | 1.84 | 0.67 | 0.91 | 0.98 | 1.08 | 0.99 |
| 1.03 | 1.01 | 0.68 | 1.11 | 0.98 | 1.27 | 1.04 | 1.08 | 0.9 | 0.98 | 1 |
| 1.08 | 0.84 | 0.82 | 0.99 | 1.02 | 0.73 | 1.2 | 0.93 | 1 | 0.92 | 1.03 |
| 0.83 | 1.04 | 1.02 | 0.85 | 0.83 | 0.8 | 1.28 | 0.67 | 0.92 | 1.06 | 1.27 |
| 1.01 | 0.77 | 0.93 | 2.45 | 0.98 | 1.73 | 1.47 | 0.91 | 1.23 | 1.32 | 2.7 |
| 0.97 | 0.93 | 0.84 | 1.1 | 0.84 | 0.7 | 1.38 | 0.79 | 1.06 | 0.99 | 1.07 |
| 1.08 | 0.89 | 1.52 | 0.83 | 0.53 | 1.47 | 1.05 | 1.36 | 1.14 | 0.88 | 1.21 |
| 0.97 | 1.19 | 0.99 | 1.52 | 1.02 | 1.95 | 0.8 | 0.82 | 1.06 | 1 | 1.18 |
| 1.05 | 0.98 | 1.27 | 1.18 | 1.89 | 1.18 | 1.72 | 1.2 | 1.07 | 1.2 | 1.21 |
| 0.94 | 1.24 | 1.15 | 0.73 | 0.79 | 2.21 | 1.53 | 0.92 | 1.09 | 1.28 | 0.95 |
| 0.64 | 0.97 | 0.74 | 0.82 | 3.43 | 0.44 | 0.45 | 0.65 | 1.38 | 1.46 | 1.36 |
| 1 | 1.37 | 0.87 | 1 | 1.08 | 1.56 | 1.07 | 0.93 | 1.1 | 0.98 | 0.98 |
| 0.96 | 1.4 | 0.69 | 0.96 | 1.1 | 1.07 | 0.89 | 1 | 0.91 | 1.03 | 0.96 |
| 1.29 | 1.4 | 1.23 | 0.97 | 2.1 | 1.1 | 1.03 | 0.85 | 0.9 | 1.02 | 1.04 |
| 1 | 0.9 | 1.29 | 1.03 | 0.91 | 1.07 | 0.8 | 0.92 | 0.96 | 0.83 | 0.83 |
| 0.87 | 1.52 | 0.97 | 1.22 | 0.7 | 0.97 | 0.85 | 1.02 | 0.84 | 1.19 | 1.02 |
| 1.1 | 1.1 | 1.86 | 1.17 | 0.65 | 1.07 | 1.46 | 1.1 | 1.04 | 1.13 | 1.01 |
| 1.49 | 0.84 | 1.51 | 1.08 | 2.18 | 0.94 | 0.46 | 0.7 | 1.21 | 1.16 | 1.58 |
| 1.05 | 1.12 | 1.06 | 1.01 | 6.42 | 0.92 | 1.02 | 1.19 | 1.06 | 1.03 | 1.25 |
| 0.88 | 0.86 | 1.38 | 0.83 | 0.99 | 1.65 | 1.37 | 1.16 | 0.94 | 1.08 | 1.25 |
| 1.13 | 0.88 | 0.93 | 1.1 | 1.09 | 0.94 | 1.32 | 0.84 | 1.38 | 1.12 | 0.98 |
| 0.68 | 1.16 | 1.26 | 0.89 | 0.9 | 1.05 | 1.06 | 0.77 | 1.15 | 1.18 | 0.75 |
| 1.12 | 0.68 | 1.36 | 0.96 | 0.94 | 0.91 | 0.88 | 0.95 | 1.29 | 0.91 | 0.76 |
| 1.22 | 1.33 | 1.48 | 0.98 | 1.16 | 1.23 | 1.25 | 1.12 | 0.82 | 0.99 | 0.9 |
| 0.96 | 0.92 | 1.64 | 1.01 | 1.12 | 0.66 | 1.05 | 0.86 | 1.07 | 1.08 | 1.12 |
| 0.93 | 0.86 | 0.84 | 0.77 | 0.85 | 1.57 | 0.97 | 0.93 | 1.19 | 1.03 | 0.7 |
| 1.13 | 1.03 | 1.06 | 0.9 | 9.13 | 1.11 | 0.85 | 1.28 | 0.81 | 0.7 | 1.01 |
| 1.01 | 0.96 | 1.02 | 1 | 1.2 | 1.23 | 0.8 | 1.06 | 1.01 | 0.85 | 0.96 |
| 1.01 | 0.9 | 1.13 | 1.09 | 1.12 | 1.05 | 0.8 | 1.05 | 1.1 | 1.02 | 0.92 |

| | | | | | | | | | | |
|---|---|---|---|---|---|---|---|---|---|---|
| 1.33 | 0.95 | 3.08 | 2.38 | 0.92 | 0.87 | 0.69 | 1.27 | 6.69 | 0.66 | 0.6 |
| 1.08 | 1.18 | 1.27 | 1.18 | 3.32 | 0.97 | 0.85 | 1.25 | 0.82 | 1.01 | 1.15 |
| 1.08 | 1.08 | 0.82 | 0.96 | 1.01 | 1.01 | 0.93 | 1.16 | 1.01 | 1.03 | 0.91 |
| 0.97 | 0.64 | 0.97 | 1.06 | 0.67 | 1.11 | 1.34 | 0.93 | 1.19 | 1.18 | 1.1 |
| 1.49 | 1.53 | 2.87 | 0.52 | 1.11 | 0.28 | 0.68 | 1.04 | 0.51 | 0.61 | 1.2 |
| 0.98 | 1.23 | 0.95 | 0.71 | 1.05 | 1.31 | 1.2 | 1.21 | 0.99 | 0.88 | 1.01 |
| 1.62 | 1.37 | 0.83 | 1.3 | 5.23 | 0.28 | 0.66 | 0.88 | 0.7 | 1.02 | 0.92 |
| 1.01 | 0.86 | 0.69 | 1 | 1.07 | 1.11 | 1.4 | 0.85 | 0.93 | 1.01 | 1.19 |
| 0.92 | 1.3 | 0.92 | 1.09 | 1.32 | 0.99 | 1.18 | 1.15 | 0.94 | 1.03 | 1.1 |
| 1.62 | 1.65 | 0.77 | 1.04 | 3 | 0.59 | 0.6 | 0.87 | 0.68 | 1.02 | 0.99 |
| 1.33 | 1.37 | 0.9 | 1.09 | 1.58 | 1.52 | 1.18 | 0.97 | 0.73 | 1 | 1.38 |
| 1.28 | 1.32 | 1.25 | 1.17 | 0.87 | 0.98 | 0.52 | 1.34 | 0.83 | 0.93 | 0.92 |
| 1.31 | 0.72 | 0.88 | 0.95 | 0.91 | 0.57 | 0.84 | 0.99 | 0.92 | 0.81 | 0.99 |
| 0.95 | 0.8 | 1.17 | 0.95 | 2.86 | 0.56 | 0.93 | 0.9 | 0.91 | 0.97 | 1.01 |
| 1.05 | 0.77 | 1.2 | 0.91 | 1.02 | 0.97 | 1.12 | 1.01 | 0.92 | 1.06 | 1.25 |
| 0.98 | 1.05 | 0.93 | 1.11 | 0.76 | 0.87 | 0.71 | 1 | 1.06 | 1.05 | 1.13 |
| 1.16 | 1.19 | 1.54 | 0.98 | 1.18 | 1.44 | 1.14 | 0.99 | 1.19 | 1.03 | 0.93 |
| 0.9 | 0.95 | 0.78 | 1.07 | 0.64 | 0.51 | 1.17 | 0.92 | 0.78 | 0.93 | 0.92 |
| 1.15 | 0.71 | 0.99 | 0.92 | 3.68 | 2.09 | 0.93 | 1.27 | 1.21 | 1.3 | 1.36 |
| 1.08 | 0.82 | 1.72 | 1.1 | 1.32 | 2 | 0.8 | 1.18 | 1.12 | 1.31 | 1.45 |
| 0.94 | 0.95 | 1.11 | 0.94 | 0.93 | 1.69 | 2.1 | 1.12 | 1 | 0.78 | 0.59 |
| 1.45 | 1.02 | 1.12 | 1.03 | 1.94 | 1.78 | 1.09 | 1.44 | 0.57 | 1.99 | 0.89 |
| 1.05 | 1.01 | 0.95 | 1.11 | 2.21 | 1.09 | 0.89 | 1 | 0.96 | 0.99 | 0.97 |
| 1.18 | 1.36 | 1.61 | 1.26 | 0.95 | 1.09 | 0.98 | 1.22 | 3.2 | 1.18 | 0.86 |
| 0.8 | 1.05 | 1.22 | 1.01 | 3.03 | 1.48 | 0.87 | 0.83 | 1.13 | 0.91 | 0.97 |
| 0.83 | 0.81 | 0.72 | 1.89 | 0.08 | 1.23 | 1.75 | 1.43 | 1.07 | 1.61 | 1.25 |
| 0.98 | 0.94 | 1.19 | 1.1 | 1.16 | 0.71 | 1 | 1.23 | 0.87 | 1.19 | 0.95 |
| 1.07 | 1.07 | 0.96 | 1.04 | 1.09 | 1.2 | 1.14 | 1.05 | 0.95 | 0.84 | 1.13 |
| 0.99 | 1.03 | 0.8 | 0.95 | 0.98 | 1.03 | 0.97 | 0.93 | 1.12 | 1.01 | 1 |
| 0.86 | 1.08 | 0.73 | 1.05 | 0.97 | 1.19 | 1.02 | 0.83 | 0.84 | 1.1 | 1.03 |
| 0.82 | 0.95 | 1.41 | 1.5 | 0.85 | 0.6 | 0.89 | 0.87 | 1.16 | 1.03 | 0.74 |
| 1 | 0.99 | 0.53 | 0.91 | 0.8 | 0.89 | 0.88 | 0.73 | 0.84 | 0.95 | 1.13 |
| 0.98 | 0.75 | 0.53 | 0.87 | 2.32 | 3.42 | 1.23 | 0.98 | 1 | 0.85 | 0.91 |

| | | | | | | | | | | |
|---|---|---|---|---|---|---|---|---|---|---|
| 1.13 | 2.37 | 1.25 | 0.97 | 1.39 | 1.18 | 1.13 | 0.8 | 1.18 | 0.86 | 0.92 |
| 1.06 | 0.91 | 0.71 | 1.01 | 0.83 | 0.6 | 1.4 | 0.88 | 0.94 | 0.79 | 1.02 |
| 1.31 | 0.98 | 1.33 | 1.07 | 1.02 | 0.87 | 0.85 | 0.94 | 0.83 | 1.08 | 1.07 |
| 0.98 | 0.59 | 1.05 | 0.66 | 0.5 | 0.76 | 0.63 | 1.28 | 0.62 | 1.73 | 0.77 |
| 1.42 | 0.9 | 1.37 | 0.91 | 2.06 | 0.86 | 1.04 | 0.9 | 0.93 | 1.13 | 1.17 |
| 1.13 | 0.78 | 1.05 | 1.05 | 5.33 | 1.52 | 1.04 | 1.28 | 0.94 | 1.08 | 1.06 |
| 0.79 | 0.76 | 0.72 | 0.91 | 0.91 | 0.99 | 0.56 | 0.96 | 0.99 | 0.66 | 0.94 |
| 1.13 | 1.12 | 0.91 | 1.11 | 1.37 | 0.78 | 0.63 | 0.79 | 0.8 | 1.25 | 1.05 |
| 0.93 | 1.12 | 2.67 | 1.21 | 1.2 | 1.31 | 0.82 | 1.3 | 3.39 | 0.71 | 0.67 |
| 1.05 | 2.52 | 1.71 | 0.85 | 1.11 | 1.02 | 1.16 | 1.27 | 0.94 | 1.1 | 0.87 |
| 0.92 | 0.76 | 1.12 | 1.08 | 0.84 | 0.53 | 0.32 | 0.79 | 0.77 | 0.87 | 1.01 |
| 0.98 | 0.84 | 1.39 | 0.89 | 0.8 | 1.07 | 0.91 | 1.05 | 0.83 | 1 | 1.09 |
| 0.92 | 0.91 | 0.62 | 1.09 | 1.48 | 0.87 | 0.83 | 0.85 | 0.76 | 0.91 | 0.97 |
| 0.87 | 0.93 | 0.82 | 1.06 | 3.11 | 0.83 | 1.11 | 0.74 | 0.96 | 0.93 | 0.71 |
| 0.98 | 0.92 | 1.42 | 1.07 | 2.14 | 0.65 | 1 | 0.94 | 0.91 | 1.25 | 1.16 |
| 1.2 | 1 | 1.04 | 1.03 | 1.35 | 1.05 | 0.9 | 0.9 | 0.67 | 1.18 | 1.7 |
| 0.82 | 1.13 | 0.95 | 1.01 | 1.4 | 1.26 | 1.47 | 1.06 | 0.74 | 0.92 | 1.07 |
| 0.85 | 1.02 | 1.11 | 0.86 | 5.3 | 0.87 | 0.7 | 0.83 | 0.84 | 0.99 | 1.62 |
| 0.77 | 0.69 | 1.09 | 0.78 | 0.68 | 0.63 | 1.06 | 0.96 | 2.21 | 0.73 | 0.6 |
| 0.48 | 0.34 | 0.32 | 0.75 | 2.07 | 2.26 | 0.4 | 1.35 | 0.76 | 1.35 | 0.99 |
| 1.05 | 0.65 | 0.93 | 0.91 | 0.98 | 0.78 | 1.07 | 0.9 | 1.33 | 0.91 | 0.85 |
| 1.65 | 1.43 | 0.77 | 1.16 | 2.58 | 0.54 | 0.6 | 0.87 | 0.68 | 1 | 0.98 |
| 2.08 | 0.95 | 1.31 | 1.3 | 2.31 | 1.52 | 0.56 | 1.07 | 0.93 | 1.07 | 0.54 |
| 0.8 | 0.63 | 0.8 | 1.56 | 1.56 | 1.1 | 0.53 | 0.67 | 0.84 | 1.19 | 0.8 |
| 0.99 | 0.95 | 0.89 | 0.78 | 0.87 | 0.9 | 1.1 | 1.02 | 1.24 | 0.8 | 1.07 |
| 0.87 | 1.1 | 0.79 | 1.23 | 1.53 | 1.09 | 1.04 | 0.99 | 1.33 | 0.83 | 0.96 |
| 0.89 | 0.85 | 1.2 | 1.46 | 1.59 | 0.7 | 1.18 | 1.29 | 0.82 | 0.9 | 1.03 |
| 1.06 | 1.76 | 0.91 | 0.81 | 0.66 | 1.12 | 0.49 | 0.81 | 1.14 | 0.88 | 1 |
| 1.01 | 1.9 | 0.84 | 1.31 | 1.43 | 1.11 | 0.7 | 1.03 | 0.75 | 0.72 | 0.53 |
| 0.81 | 1.28 | 0.39 | 0.31 | 2.53 | 0.8 | 3 | 0.49 | 1.99 | 1.02 | 0.56 |
| 0.84 | 1.38 | 1.59 | 1.08 | 0.74 | 2.67 | 0.71 | 1.01 | 1.27 | 0.98 | 1.71 |
| 0.84 | 0.99 | 2.05 | 2.36 | 0.97 | 1.77 | 1.1 | 1.15 | 0.94 | 1.64 | 1.25 |
| 1.17 | 2.79 | 1.18 | 1.9 | 5.32 | 0.47 | 1.81 | 0.95 | 1.4 | 0.81 | 1.54 |

| | | | | | | | | | | |
|---|---|---|---|---|---|---|---|---|---|---|
| 1.11 | 0.87 | 0.77 | 0.84 | 1.22 | 9.25 | 1 | 0.8 | 1.07 | 1.04 | 0.99 |
| 1.16 | 1.54 | 1.11 | 0.93 | 1.06 | 1.29 | 0.79 | 1.1 | 1.06 | 1.23 | 0.91 |
| 1.91 | 0.96 | 1.04 | 1.02 | 1.23 | 4.73 | 0.96 | 1.97 | 2.45 | 1.72 | 5.42 |
| 0.98 | 0.72 | 1.6 | 0.61 | 1.03 | 0.9 | 0.96 | 0.95 | 0.59 | 0.61 | 1.86 |
| 1.01 | 0.87 | 0.73 | 0.98 | 4.08 | 0.75 | 0.97 | 0.6 | 0.73 | 0.77 | 0.86 |
| 1.08 | 0.93 | 1.34 | 1 | 1.6 | 0.97 | 1.07 | 1.25 | 1.28 | 1.13 | 0.99 |
| 1.08 | 1.21 | 1.13 | 1.05 | 1.18 | 0.83 | 1.11 | 1 | 1.25 | 1.19 | 1.03 |
| 1.05 | 1.09 | 1.26 | 0.91 | 0.87 | 1.33 | 1.32 | 0.98 | 1.07 | 1 | 0.78 |
| 1.04 | 0.82 | 1.02 | 0.81 | 0.72 | 0.64 | 1.38 | 1.14 | 1.24 | 0.99 | 1.24 |
| 0.97 | 0.83 | 0.84 | 1.41 | 0.64 | 2.13 | 0.9 | 1.06 | 1.08 | 0.86 | 0.98 |
| 0.8 | 0.89 | 0.98 | 1.06 | 0.94 | 0.83 | 1.05 | 0.94 | 1.25 | 1.14 | 1.2 |
| 7.23 | 0.1 | 8.12 | 8.19 | 35.96 | 0.39 | 3.33 | 3.23 | 1.69 | 23.3 | 6.88 |
| 0.88 | 1.21 | 0.77 | 0.89 | 1.64 | 0.91 | 0.87 | 1.16 | 1.35 | 1.16 | 1.04 |
| 1 | 0.75 | 1.35 | 0.73 | 1.02 | 1.74 | 0.76 | 0.85 | 1.16 | 0.95 | 1.09 |

| 218902_at | 209205_s_at | 201244_s_at | 206283_s_at | 202387_at | 212248_at | 201432_at | 213342_at | 218902_at | 218902_at | 209214_s_at |
|---|---|---|---|---|---|---|---|---|---|---|
| 1.27 | 0.48 | 0.97 | 0.67 | 1.16 | 0.84 | 1.13 | 1.02 | 1.27 | 1.27 | 0.96 |
| 1.02 | 0.86 | 1.01 | 0.68 | 0.89 | 0.86 | 1.11 | 2.17 | 1.02 | 1.02 | 1.17 |
| 1.77 | 0.76 | 1.05 | 1.16 | 1.12 | 2.16 | 1.05 | 2.24 | 1.77 | 1.77 | 0.93 |
| 1.36 | 1.05 | 0.95 | 1.58 | 1.33 | 1.02 | 1.16 | 1.37 | 1.36 | 1.36 | 0.97 |
| 1.27 | 0.97 | 0.92 | 1.37 | 0.97 | 1.07 | 0.81 | 1.07 | 1.27 | 1.27 | 0.97 |
| 0.91 | 1.76 | 1.14 | 1.09 | 0.88 | 0.79 | 1.81 | 3.76 | 0.91 | 0.91 | 1.22 |
| 0.34 | 0.86 | 0.77 | 0.9 | 1.32 | 1.39 | 1.05 | 1.04 | 0.34 | 0.34 | 0.93 |
| 1.05 | 1.04 | 1.09 | 0.72 | 1.12 | 1.05 | 1.03 | 3.5 | 1.05 | 1.05 | 1.11 |
| 0.77 | 0.71 | 1.09 | 2.13 | 0.68 | 1.28 | 0.81 | 1.01 | 0.77 | 0.77 | 0.91 |
| 1.23 | 0.42 | 1.24 | 0.93 | 1.45 | 0.26 | 0.55 | 1.04 | 1.23 | 1.23 | 1.53 |
| 1.3 | 0.57 | 0.82 | 1.35 | 1.63 | 1.17 | 1.04 | 0.63 | 1.3 | 1.3 | 0.88 |
| 0.9 | 1.01 | 0.94 | 1.05 | 1.02 | 1.04 | 1.3 | 1.22 | 0.9 | 0.9 | 1.11 |
| 0.96 | 1.17 | 1.24 | 0.59 | 0.88 | 1.17 | 1.21 | 0.91 | 0.96 | 0.96 | 0.91 |
| 0.65 | 3.23 | 0.04 | 10.38 | 2.27 | 0 | 0.16 | 0.47 | 0.65 | 0.65 | 3.69 |
| 0.72 | 0.52 | 1.75 | 1.19 | 1.21 | 1.09 | 1.54 | 1.04 | 0.72 | 0.72 | 1.51 |
| 0.87 | 2.06 | 1.21 | 1.11 | 0.88 | 1.1 | 1.22 | 1.26 | 0.87 | 0.87 | 0.8 |
| 1.15 | 0.87 | 1.09 | 5.35 | 0.86 | 1.07 | 1.11 | 0.94 | 1.15 | 1.15 | 1.32 |
| 1.52 | 0.97 | 1.13 | 1.27 | 1.03 | 0.87 | 0.83 | 1.36 | 1.52 | 1.52 | 0.95 |
| 6.36 | 1.04 | 0.72 | 0.75 | 0.79 | 0.94 | 1 | 2.72 | 6.36 | 6.36 | 0.9 |
| 0.57 | 0.97 | 0.68 | 1.7 | 0.82 | 0.81 | 0.78 | 0.93 | 0.57 | 0.57 | 0.93 |
| 1.06 | 0.46 | 1.4 | 1.93 | 0.88 | 1.56 | 1.12 | 0.88 | 1.06 | 1.06 | 1.29 |
| 1.18 | 0.28 | 0.98 | 10.17 | 1.29 | 0.41 | 1.01 | 0.53 | 1.18 | 1.18 | 0.57 |
| 0.75 | 0.7 | 0.98 | 1.6 | 1.06 | 1.05 | 1.43 | 0.87 | 0.75 | 0.75 | 0.86 |
| 0.28 | 0.77 | 0.96 | 1.12 | 1.23 | 1.17 | 1.26 | 0.78 | 0.28 | 0.28 | 0.98 |
| 0.52 | 0.54 | 0.51 | 0.33 | 0.56 | 1.06 | 1.56 | 1.35 | 0.52 | 0.52 | 0.79 |
| 1.86 | 0.68 | 0.99 | 0.68 | 1.28 | 0.76 | 0.76 | 1.07 | 1.86 | 1.86 | 1.07 |
| 0.88 | 1.16 | 0.78 | 0.36 | 0.91 | 0.69 | 1.58 | 0.87 | 0.88 | 0.88 | 1.08 |
| 0.59 | 1.33 | 0.91 | 2 | 1.37 | 0.57 | 1.34 | 0.67 | 0.59 | 0.59 | 0.81 |
| 1.47 | 0.69 | 1.44 | 2.23 | 1.43 | 0.46 | 3.64 | 0.51 | 1.47 | 1.47 | 1.13 |
| 1.27 | 0.86 | 1.18 | 1.35 | 1.34 | 0.99 | 0.83 | 1.32 | 1.27 | 1.27 | 0.91 |

| | | | | | | | | | | |
|---|---|---|---|---|---|---|---|---|---|---|
| 0.62 | 0.44 | 0.89 | 1.21 | 0.99 | 1.01 | 0.47 | 5.19 | 0.62 | 0.62 | 1.07 |
| 0.83 | 1.67 | 3.08 | 0.62 | 6.99 | 12.02 | 11.35 | 1.16 | 0.83 | 0.83 | 1.94 |
| 1.12 | 0.97 | 1.1 | 0.95 | 0.93 | 1.36 | 1 | 0.96 | 1.12 | 1.12 | 0.99 |
| 0.4 | 0.99 | 0.76 | 2.61 | 0.67 | 0.7 | 0.74 | 0.78 | 0.4 | 0.4 | 2.96 |
| 0.47 | 1.18 | 6.1 | 0.87 | 1.19 | 0.4 | 0.32 | 40.4 | 0.47 | 0.47 | 1.69 |
| 5.39 | 1 | 0.73 | 1.94 | 1.01 | 0.66 | 0.54 | 1.2 | 5.39 | 5.39 | 2.28 |
| 2.09 | 2.6 | 0.47 | 3.8 | 3.11 | 1.26 | 0.43 | 0.57 | 2.09 | 2.09 | 0.96 |
| 1 | 0.79 | 0.95 | 1.06 | 1.43 | 0.87 | 0.93 | 0.88 | 1 | 1 | 0.41 |
| 0.71 | 0.65 | 0.88 | 3.74 | 0.8 | 1.55 | 1.89 | 0.63 | 0.71 | 0.71 | 1.13 |
| 0.4 | 0.65 | 0.97 | 1.15 | 0.46 | 0.59 | 1.8 | 1.02 | 0.4 | 0.4 | 0.67 |
| 0.99 | 1.03 | 1.21 | 0.97 | 0.72 | 1.46 | 0.8 | 1.08 | 0.99 | 0.99 | 0.86 |
| 1.66 | 1.18 | 1.08 | 0.61 | 1.13 | 1 | 1.04 | 0.9 | 1.66 | 1.66 | 1.07 |
| 0.76 | 1.97 | 1.3 | 1.09 | 1.79 | 1.66 | 0.81 | 1.36 | 0.76 | 0.76 | 0.88 |
| 1.64 | 1.06 | 1.09 | 0.92 | 0.94 | 1 | 1.23 | 1.62 | 1.64 | 1.64 | 0.83 |
| 0.86 | 1.46 | 1.29 | 0.86 | 1.53 | 1.43 | 0.93 | 1.24 | 0.86 | 0.86 | 0.91 |
| 1.06 | 1.23 | 0.99 | 1.28 | 1.16 | 1 | 1.3 | 1.29 | 1.06 | 1.06 | 1.06 |
| 0.86 | 0.94 | 1.02 | 0.23 | 1.2 | 1.12 | 1.05 | 0.84 | 0.86 | 0.86 | 1.04 |
| 0.85 | 1.21 | 1.05 | 0.77 | 1.73 | 1.04 | 0.8 | 1.23 | 0.85 | 0.85 | 0.87 |
| 1.2 | 0.92 | 0.99 | 1.12 | 0.99 | 0.93 | 0.96 | 0.95 | 1.2 | 1.2 | 1 |
| 1.29 | 0.69 | 0.97 | 1.34 | 1.66 | 1.45 | 0.72 | 0.83 | 1.29 | 1.29 | 1.27 |
| 0.69 | 0.95 | 0.92 | 0.04 | 0.63 | 1.04 | 1.34 | 0.81 | 0.69 | 0.69 | 0.97 |
| 0.86 | 0.75 | 0.89 | 1.61 | 1.13 | 0.99 | 1.01 | 0.73 | 0.86 | 0.86 | 0.95 |
| 0.82 | 0.68 | 0.9 | 1.14 | 1 | 0.69 | 1.06 | 1.18 | 0.82 | 0.82 | 0.95 |
| 1.02 | 1.54 | 1.09 | 0.9 | 1.23 | 1.33 | 1.73 | 2.26 | 1.02 | 1.02 | 0.96 |
| 1.67 | 0.99 | 0.76 | 1.87 | 0.62 | 1.37 | 0.77 | 1.23 | 1.67 | 1.67 | 1 |
| 1.01 | 0.88 | 1.03 | 12.5 | 1.46 | 0.78 | 0.92 | 1.26 | 1.01 | 1.01 | 1.53 |
| 1.08 | 0.39 | 0.86 | 0.68 | 0.7 | 0.36 | 0.63 | 1.54 | 1.08 | 1.08 | 1.12 |
| 0.88 | 1.19 | 1.07 | 2.52 | 1.01 | 0.6 | 0.75 | 1.18 | 0.88 | 0.88 | 0.73 |
| 1.52 | 0.88 | 0.96 | 1.06 | 0.58 | 1.08 | 1.27 | 0.95 | 1.52 | 1.52 | 0.89 |
| 0.86 | 1.62 | 1.49 | 1.73 | 2.33 | 1.28 | 0.78 | 1.5 | 0.86 | 0.86 | 0.93 |
| 0.61 | 0.68 | 0.84 | 1.03 | 1.15 | 1.1 | 2.85 | 1.09 | 0.61 | 0.61 | 0.84 |
| 1.39 | 0.95 | 1.04 | 0.65 | 0.88 | 1.76 | 0.93 | 0.98 | 1.39 | 1.39 | 1.07 |
| 1.53 | 0.82 | 0.95 | 1.32 | 0.94 | 0.78 | 1.33 | 1.27 | 1.53 | 1.53 | 0.84 |

| | | | | | | | | | | |
|---|---|---|---|---|---|---|---|---|---|---|
| 0.72 | 2.82 | 0.99 | 8.44 | 0.86 | 1.33 | 1.66 | 0.98 | 0.72 | 0.72 | 1.02 |
| 1.81 | 3.07 | 1.15 | 2.81 | 1.46 | 1.23 | 0.81 | 0.78 | 1.81 | 1.81 | 1.1 |
| 0.74 | 1.26 | 0.98 | 1.96 | 0.96 | 0.79 | 1.03 | 0.98 | 0.74 | 0.74 | 0.92 |
| 1.47 | 1.13 | 1.12 | 1.19 | 0.88 | 1.38 | 1.1 | 1.13 | 1.47 | 1.47 | 1.18 |
| 0.99 | 1.15 | 0.92 | 10.21 | 1.04 | 1.4 | 0.85 | 0.9 | 0.99 | 0.99 | 1.01 |
| 1.69 | 2.13 | 1.14 | 1.3 | 1.34 | 0.84 | 1.59 | 0.74 | 1.69 | 1.69 | 1.08 |
| 1.12 | 1.81 | 1.14 | 0.88 | 1.07 | 1.29 | 2.16 | 1.73 | 1.12 | 1.12 | 0.76 |
| 0.94 | 0.9 | 1.51 | 0.91 | 0.92 | 0.85 | 1.69 | 0.87 | 0.94 | 0.94 | 1.1 |
| 0.75 | 0.96 | 0.9 | 1.47 | 1.13 | 1.16 | 0.88 | 0.88 | 0.75 | 0.75 | 1.09 |
| 1.27 | 0.71 | 0.73 | 0.97 | 1.19 | 1.41 | 1.21 | 1.26 | 1.27 | 1.27 | 1.08 |
| 1.56 | 1.34 | 0.76 | 3.06 | 1.2 | 0.93 | 1.79 | 2.14 | 1.56 | 1.56 | 1.15 |
| 1.36 | 0.71 | 0.96 | 1.44 | 0.76 | 1.42 | 0.69 | 1.26 | 1.36 | 1.36 | 0.9 |
| 1.34 | 1.01 | 0.9 | 0.93 | 0.99 | 0.88 | 0.97 | 1.11 | 1.34 | 1.34 | 0.9 |
| 1.24 | 0.51 | 1.36 | 0.79 | 0.84 | 1.68 | 0.88 | 1.18 | 1.24 | 1.24 | 0.91 |
| 0.91 | 0.81 | 1.24 | 0.7 | 1.1 | 1.4 | 1.01 | 2 | 0.91 | 0.91 | 1.04 |
| 0.3 | 3.1 | 4.3 | 0.14 | 7.09 | 61.72 | 147.17 | 52.59 | 0.3 | 0.3 | 0.2 |
| 1.04 | 1.14 | 1.23 | 2.29 | 0.98 | 1.19 | 1.41 | 1.07 | 1.04 | 1.04 | 0.87 |
| 0.89 | 1.26 | 0.81 | 5 | 2.15 | 1.46 | 0.82 | 2.06 | 0.89 | 0.89 | 1.08 |
| 0.79 | 1.7 | 0.83 | 1.18 | 0.77 | 1.12 | 0.91 | 1.52 | 0.79 | 0.79 | 1.14 |
| 2.04 | 0.82 | 0.99 | 0.9 | 1.35 | 0.93 | 0.85 | 5.36 | 2.04 | 2.04 | 1.03 |
| 1.71 | 0.59 | 0.89 | 1.11 | 7.74 | 0.54 | 0.83 | 0.88 | 1.71 | 1.71 | 0.77 |
| 0.94 | 0.91 | 1.17 | 0.92 | 0.94 | 0.95 | 1.28 | 0.78 | 0.94 | 0.94 | 1.08 |
| 0.39 | 0.95 | 0.85 | 0.5 | 0.9 | 1.16 | 0.74 | 0.86 | 0.39 | 0.39 | 0.93 |
| 0.73 | 0.81 | 0.9 | 1.03 | 1.3 | 1.18 | 0.83 | 1.26 | 0.73 | 0.73 | 0.87 |
| 1.17 | 0.66 | 1.25 | 1.43 | 0.69 | 1.19 | 1.5 | 1.55 | 1.17 | 1.17 | 0.79 |
| 0.67 | 0.91 | 0.88 | 0.82 | 1 | 0.9 | 1.22 | 1.15 | 0.67 | 0.67 | 0.95 |
| 1.1 | 0.94 | 1.02 | 1.48 | 1.19 | 0.67 | 0.92 | 0.84 | 1.1 | 1.1 | 1.06 |
| 1.51 | 0.87 | 1.05 | 0.82 | 1.24 | 0.95 | 0.95 | 2.29 | 1.51 | 1.51 | 1.03 |
| 0.98 | 0.89 | 0.97 | 0.86 | 0.88 | 1.02 | 1.05 | 1.2 | 0.98 | 0.98 | 1.03 |
| 1.05 | 1.08 | 1.03 | 1.03 | 0.96 | 1.02 | 0.96 | 1.02 | 1.05 | 1.05 | 0.99 |
| 1.28 | 1.18 | 1.18 | 0.98 | 1.28 | 1.14 | 0.88 | 0.9 | 1.28 | 1.28 | 1.17 |
| 0.79 | 0.4 | 1.25 | 0.77 | 1.25 | 1.01 | 0.74 | 0.82 | 0.79 | 0.79 | 0.75 |
| 1.37 | 1.07 | 1.03 | 0.65 | 0.79 | 1.04 | 0.91 | 1.65 | 1.37 | 1.37 | 1.12 |

| | | | | | | | | | | |
|---|---|---|---|---|---|---|---|---|---|---|
| 1 | 0.81 | 1.11 | 2.01 | 1.34 | 1.62 | 0.69 | 0.8 | 1 | 1 | 0.97 |
| 1.17 | 0.88 | 0.9 | 1.17 | 0.99 | 1.01 | 1.03 | 0.63 | 1.17 | 1.17 | 1.13 |
| 1.18 | 1.07 | 1.21 | 4.84 | 1.3 | 0.83 | 1.19 | 0.99 | 1.18 | 1.18 | 1.06 |
| 1.3 | 0.66 | 0.84 | 0.5 | 1.19 | 0.75 | 0.98 | 1.11 | 1.3 | 1.3 | 1.03 |
| 0.86 | 1.24 | 1.05 | 0.82 | 1.45 | 1.27 | 1.17 | 0.73 | 0.86 | 0.86 | 1.24 |
| 0.65 | 0.58 | 1.24 | 3.03 | 1.16 | 1.72 | 0.48 | 0.6 | 0.65 | 0.65 | 1.19 |
| 0.81 | 1.02 | 1.03 | 1.27 | 2.4 | 0.8 | 0.9 | 1.16 | 0.81 | 0.81 | 0.98 |
| 0.87 | 0.77 | 1.05 | 0.87 | 1.14 | 1.66 | 1.32 | 1.26 | 0.87 | 0.87 | 0.97 |
| 0.86 | 0.84 | 1.08 | 2.58 | 0.67 | 0.74 | 1.23 | 1.23 | 0.86 | 0.86 | 1.19 |
| 0.97 | 0.76 | 1.01 | 0.82 | 0.78 | 1.24 | 0.97 | 1.19 | 0.97 | 0.97 | 0.96 |
| 1.06 | 0.66 | 1.12 | 0.98 | 0.91 | 1.21 | 1.02 | 1.2 | 1.06 | 1.06 | 1.24 |
| 1.31 | 1.11 | 1.01 | 1.18 | 0.93 | 1.21 | 1.17 | 0.91 | 1.31 | 1.31 | 1.08 |
| 1.78 | 1.17 | 0.97 | 0.79 | 1.33 | 1.01 | 0.88 | 0.91 | 1.78 | 1.78 | 1.16 |
| 0.57 | 1.05 | 0.91 | 1.34 | 0.79 | 0.95 | 0.8 | 0.98 | 0.57 | 0.57 | 1.78 |
| 0.99 | 0.25 | 1.04 | 0.99 | 0.97 | 1.2 | 0.98 | 1.11 | 0.99 | 0.99 | 0.98 |
| 1.79 | 1.08 | 0.97 | 4.62 | 1.22 | 0.89 | 0.84 | 3.63 | 1.79 | 1.79 | 1.11 |
| 1.24 | 0.35 | 0.97 | 0.79 | 0.96 | 1.18 | 1.08 | 0.56 | 1.24 | 1.24 | 0.96 |
| 1.76 | 1.25 | 1.1 | 1.08 | 0.92 | 1.38 | 1.05 | 1.29 | 1.76 | 1.76 | 1.06 |
| 1.13 | 0.98 | 0.98 | 3.47 | 0.9 | 0.64 | 1.02 | 1.48 | 1.13 | 1.13 | 0.86 |
| 1.47 | 0.97 | 1.02 | 1.11 | 1.6 | 0.86 | 0.92 | 0.96 | 1.47 | 1.47 | 1.3 |
| 1.03 | 0.6 | 1.24 | 0.59 | 1.19 | 1.19 | 0.78 | 1.34 | 1.03 | 1.03 | 1.4 |
| 1.18 | 1.14 | 1.01 | 0.49 | 0.88 | 1.04 | 0.95 | 1.03 | 1.18 | 1.18 | 1.1 |
| 1.13 | 1.17 | 0.97 | 1.23 | 0.86 | 1 | 1.07 | 0.76 | 1.13 | 1.13 | 1.09 |
| 2.95 | 1.95 | 1.15 | 1.18 | 0.81 | 1.04 | 1.23 | 0.67 | 2.95 | 2.95 | 0.89 |
| 1.7 | 0.7 | 0.83 | 1.14 | 0.92 | 0.8 | 1.13 | 1.2 | 1.7 | 1.7 | 1.27 |
| 0.54 | 0.67 | 0.92 | 1.32 | 1.14 | 1.22 | 1 | 1.39 | 0.54 | 0.54 | 1.04 |
| 1.01 | 0.92 | 0.98 | 0.96 | 1.14 | 1.14 | 0.73 | 0.99 | 1.01 | 1.01 | 1.04 |
| 1.5 | 1.65 | 0.89 | 1.98 | 0.43 | 0.71 | 1.13 | 0.87 | 1.5 | 1.5 | 0.87 |
| 0.51 | 0.89 | 0.89 | 3.5 | 0.48 | 0.79 | 1.11 | 1.12 | 0.51 | 0.51 | 1.09 |
| 1.12 | 0.83 | 0.93 | 3.91 | 1.05 | 0.97 | 0.91 | 0.86 | 1.12 | 1.12 | 1.02 |
| 1.12 | 0.92 | 0.84 | 7.61 | 0.91 | 0.88 | 0.98 | 0.2 | 1.12 | 1.12 | 0.83 |
| 1.81 | 3.07 | 1.15 | 2.81 | 1.46 | 1.23 | 0.81 | 0.78 | 1.81 | 1.81 | 1.1 |
| 0.89 | 1.03 | 1.06 | 0.92 | 1.14 | 1.36 | 0.81 | 1.15 | 0.89 | 0.89 | 1.05 |

| | | | | | | | | | | |
|---|---|---|---|---|---|---|---|---|---|---|
| 0.98 | 0.47 | 1.21 | 0.7 | 0.91 | 1.2 | 1.47 | 1.32 | 0.98 | 0.98 | 1 |
| 0.95 | 0.77 | 0.96 | 9.19 | 0.56 | 0.89 | 1.05 | 1.01 | 0.95 | 0.95 | 1.02 |
| 1.23 | 1.02 | 0.97 | 0.52 | 1.09 | 1.03 | 0.91 | 1.25 | 1.23 | 1.23 | 0.86 |
| 1.23 | 1.06 | 1.01 | 0.98 | 1.05 | 1.02 | 1.02 | 8.57 | 1.23 | 1.23 | 0.96 |
| 0.67 | 1.04 | 0.97 | 0.91 | 1.13 | 0.89 | 1.1 | 0.94 | 0.67 | 0.67 | 0.98 |
| 1.04 | 1.34 | 1.05 | 3 | 1.21 | 1.02 | 1.03 | 1.05 | 1.04 | 1.04 | 1.07 |
| 1.2 | 0.6 | 1.02 | 1.09 | 0.97 | 1.11 | 0.9 | 0.94 | 1.2 | 1.2 | 1.16 |
| 1.28 | 0.98 | 0.89 | 1.19 | 0.78 | 1.01 | 0.83 | 1.18 | 1.28 | 1.28 | 0.98 |
| 1.47 | 0.96 | 0.62 | 1.23 | 1.3 | 0.83 | 0.56 | 0.62 | 1.47 | 1.47 | 1.02 |
| 1.38 | 1.03 | 0.95 | 0.82 | 1.03 | 0.95 | 1.05 | 1.05 | 1.38 | 1.38 | 1.05 |
| 1.05 | 1.02 | 0.92 | 0.78 | 1.06 | 1.01 | 0.92 | 0.59 | 1.05 | 1.05 | 1.11 |
| 0.8 | 1.22 | 1.09 | 2.6 | 1.04 | 0.94 | 0.95 | 1.09 | 0.8 | 0.8 | 1.22 |
| 1.72 | 1.05 | 1.17 | 0.94 | 1.05 | 1.58 | 1.34 | 1.15 | 1.72 | 1.72 | 1.08 |
| 1.53 | 0.88 | 0.91 | 1.3 | 1.16 | 0.96 | 0.92 | 1.08 | 1.53 | 1.53 | 0.84 |
| 0.45 | 8.73 | 1.06 | 2.19 | 0.98 | 1.14 | 0.58 | 2.65 | 0.45 | 0.45 | 0.69 |
| 1.07 | 1.15 | 1.06 | 0.95 | 0.95 | 0.91 | 1.23 | 0.28 | 1.07 | 1.07 | 1.01 |
| 0.89 | 0.93 | 0.94 | 1.21 | 1.55 | 0.87 | 0.96 | 0.84 | 0.89 | 0.89 | 0.97 |
| 1.03 | 0.5 | 1.09 | 1.3 | 1.75 | 0.65 | 0.73 | 2.47 | 1.03 | 1.03 | 1.32 |
| 0.8 | 0.77 | 1.03 | 1.44 | 0.84 | 1.06 | 1.02 | 0.87 | 0.8 | 0.8 | 1.01 |
| 0.85 | 1.32 | 0.73 | 1.09 | 0.76 | 0.77 | 0.45 | 1.02 | 0.85 | 0.85 | 1.05 |
| 1.46 | 1.7 | 1.15 | 1.7 | 0.81 | 1.03 | 1.27 | 1.15 | 1.46 | 1.46 | 0.9 |
| 0.46 | 1.22 | 1.12 | 2.91 | 0.99 | 1.16 | 0.5 | 1.23 | 0.46 | 0.46 | 0.89 |
| 1.02 | 0.93 | 0.99 | 1.34 | 1.09 | 0.85 | 1.24 | 3.56 | 1.02 | 1.02 | 1.17 |
| 1.37 | 1.15 | 0.98 | 1.71 | 1.77 | 0.99 | 1.36 | 1.37 | 1.37 | 1.37 | 0.74 |
| 1.32 | 1.42 | 0.98 | 0.88 | 0.97 | 0.97 | 0.88 | 1.32 | 1.32 | 1.32 | 0.94 |
| 1.06 | 0.8 | 0.78 | 2.83 | 1.33 | 0.78 | 0.74 | 0.82 | 1.06 | 1.06 | 0.96 |
| 0.88 | 1.18 | 1.62 | 0.65 | 1.19 | 0.71 | 1.16 | 0.83 | 0.88 | 0.88 | 0.8 |
| 1.25 | 0.96 | 1.1 | 0.92 | 1.29 | 0.97 | 0.9 | 1.13 | 1.25 | 1.25 | 1.09 |
| 1.05 | 1.27 | 1.02 | 0.75 | 0.83 | 0.92 | 1.01 | 1.2 | 1.05 | 1.05 | 0.94 |
| 0.97 | 0.7 | 0.85 | 1.38 | 0.85 | 0.72 | 1.08 | 0.83 | 0.97 | 0.97 | 1.03 |
| 0.85 | 1.13 | 1.07 | 1.44 | 0.91 | 0.82 | 0.88 | 2.19 | 0.85 | 0.85 | 1.13 |
| 0.8 | 0.98 | 1.04 | 0.7 | 0.97 | 0.93 | 0.96 | 1.03 | 0.8 | 0.8 | 0.97 |
| 0.8 | 1.08 | 0.96 | 1.38 | 1.03 | 0.99 | 1.06 | 3.17 | 0.8 | 0.8 | 1.09 |

| | | | | | | | | | | |
|---|---|---|---|---|---|---|---|---|---|---|
| 0.69 | 0.67 | 0.9 | 1.28 | 1.07 | 1.07 | 1.12 | 1.31 | 0.69 | 0.69 | 1.15 |
| 0.85 | 1.09 | 1.08 | 1.56 | 0.98 | 0.96 | 0.91 | 1.12 | 0.85 | 0.85 | 1.02 |
| 0.93 | 0.98 | 1.02 | 2.36 | 1.11 | 1.21 | 0.98 | 1.04 | 0.93 | 0.93 | 1.03 |
| 1.34 | 1.03 | 1.12 | 1.73 | 0.67 | 1.05 | 1.25 | 0.5 | 1.34 | 1.34 | 0.86 |
| 0.68 | 2.34 | 0.81 | 0.38 | 1.16 | 0.65 | 0.85 | 0.75 | 0.68 | 0.68 | 0.92 |
| 1.2 | 0.99 | 0.85 | 0.92 | 0.81 | 1.01 | 0.97 | 0.95 | 1.2 | 1.2 | 1.18 |
| 0.66 | 0.45 | 0.89 | 1.46 | 0.95 | 1 | 0.43 | 4.07 | 0.66 | 0.66 | 1.02 |
| 1.4 | 0.81 | 1.09 | 23.41 | 1.1 | 1.09 | 1.08 | 0.77 | 1.4 | 1.4 | 0.98 |
| 1.18 | 1.81 | 1 | 0.95 | 1.06 | 0.87 | 0.93 | 1.62 | 1.18 | 1.18 | 0.99 |
| 0.6 | 0.46 | 0.89 | 1.64 | 1.1 | 1.03 | 0.49 | 2.85 | 0.6 | 0.6 | 1.02 |
| 1.18 | 1.07 | 1.05 | 1.4 | 0.65 | 0.77 | 1.06 | 0.93 | 1.18 | 1.18 | 1.12 |
| 0.52 | 0.75 | 1.18 | 1.76 | 1.3 | 0.62 | 0.81 | 1.27 | 0.52 | 0.52 | 1 |
| 0.84 | 0.62 | 0.84 | 0.92 | 0.88 | 0.94 | 0.84 | 0.85 | 0.84 | 0.84 | 1.05 |
| 0.93 | 0.63 | 0.94 | 0.95 | 0.97 | 0.97 | 0.99 | 4.53 | 0.93 | 0.93 | 0.88 |
| 1.12 | 0.96 | 1.1 | 1.19 | 0.94 | 0.99 | 0.94 | 3.14 | 1.12 | 1.12 | 1 |
| 0.71 | 1.02 | 1.03 | 0.67 | 0.9 | 0.88 | 1.46 | 1.18 | 0.71 | 0.71 | 1.12 |
| 1.14 | 0.89 | 1.09 | 1.32 | 1.15 | 0.87 | 0.91 | 5 | 1.14 | 1.14 | 1.06 |
| 1.17 | 0.97 | 0.94 | 2.53 | 0.83 | 0.97 | 0.91 | 1.01 | 1.17 | 1.17 | 1.04 |
| 0.93 | 0.82 | 0.71 | 1.18 | 0.58 | 1.35 | 0.76 | 1.38 | 0.93 | 0.93 | 0.96 |
| 0.8 | 1.51 | 1.14 | 1.09 | 0.78 | 1.7 | 1.16 | 1.48 | 0.8 | 0.8 | 0.99 |
| 2.1 | 1.02 | 1.06 | 3.93 | 1.25 | 0.76 | 1.01 | 0.9 | 2.1 | 2.1 | 1.44 |
| 1.09 | 0.37 | 1.23 | 1.3 | 1.34 | 0.6 | 1.34 | 0.94 | 1.09 | 1.09 | 1.2 |
| 0.89 | 0.63 | 1.08 | 0.85 | 1.02 | 0.92 | 1.08 | 1.3 | 0.89 | 0.89 | 0.89 |
| 0.98 | 1.45 | 0.91 | 1.21 | 1.01 | 1.43 | 1.07 | 1.58 | 0.98 | 0.98 | 1.21 |
| 0.87 | 1.15 | 1.03 | 1.63 | 0.94 | 1.01 | 0.93 | 1.04 | 0.87 | 0.87 | 0.97 |
| 1.75 | 3.2 | 1 | 1.19 | 1 | 1.81 | 2.26 | 0.84 | 1.75 | 1.75 | 0.82 |
| 1 | 1.03 | 1.06 | 2.07 | 1.05 | 1.09 | 0.94 | 1.39 | 1 | 1 | 1.1 |
| 1.14 | 0.95 | 0.94 | 2.17 | 1.19 | 0.97 | 1.09 | 2.72 | 1.14 | 1.14 | 0.99 |
| 0.97 | 0.85 | 1.07 | 0.94 | 1.04 | 1.08 | 0.94 | 3.84 | 0.97 | 0.97 | 1.09 |
| 1.02 | 0.9 | 0.91 | 1.01 | 1.07 | 1.23 | 1.15 | 0.97 | 1.02 | 1.02 | 0.99 |
| 0.89 | 1.14 | 1.25 | 0.67 | 3.03 | 0.99 | 1.43 | 1.14 | 0.89 | 0.89 | 0.94 |
| 0.88 | 0.94 | 0.98 | 0.31 | 0.86 | 0.96 | 0.8 | 0.9 | 0.88 | 0.88 | 0.95 |
| 1.23 | 0.74 | 1.02 | 3.33 | 2.61 | 0.91 | 0.97 | 0.66 | 1.23 | 1.23 | 0.87 |

| | | | | | | | | | | |
|---|---|---|---|---|---|---|---|---|---|---|
| 1.13 | 0.74 | 1.06 | 4.75 | 1.22 | 0.75 | 0.95 | 0.95 | 1.13 | 1.13 | 0.98 |
| 1.4 | 1.39 | 0.91 | 0.91 | 1.17 | 1.41 | 0.97 | 1.44 | 1.4 | 1.4 | 1.23 |
| 0.85 | 0.92 | 0.91 | 2.28 | 0.94 | 1.33 | 0.79 | 0.79 | 0.85 | 0.85 | 1.43 |
| 0.63 | 1.12 | 0.74 | 0.59 | 0.55 | 1.46 | 0.87 | 0.74 | 0.63 | 0.63 | 1.1 |
| 1.04 | 0.72 | 1.2 | 1.48 | 1.59 | 0.62 | 0.75 | 2.21 | 1.04 | 1.04 | 1.26 |
| 1.04 | 0.66 | 0.97 | 2.8 | 0.88 | 1.04 | 1.12 | 1.08 | 1.04 | 1.04 | 1.07 |
| 0.56 | 0.71 | 0.96 | 0.63 | 0.9 | 1.07 | 0.94 | 0.78 | 0.56 | 0.56 | 0.98 |
| 0.63 | 1.03 | 0.98 | 1.12 | 1.18 | 1.15 | 0.96 | 0.29 | 0.63 | 0.63 | 1.01 |
| 0.82 | 1.05 | 1.01 | 0.93 | 1.19 | 0.94 | 0.61 | 0.79 | 0.82 | 0.82 | 1 |
| 1.16 | 0.99 | 1.01 | 3.71 | 0.52 | 1.47 | 1.34 | 1.4 | 1.16 | 1.16 | 0.85 |
| 0.32 | 0.74 | 0.96 | 0.68 | 0.98 | 0.96 | 1.04 | 1.33 | 0.32 | 0.32 | 1.02 |
| 0.91 | 1.03 | 0.94 | 1.93 | 1.12 | 1.24 | 0.92 | 0.62 | 0.91 | 0.91 | 0.96 |
| 0.83 | 0.76 | 0.97 | 1.19 | 1.4 | 0.81 | 0.8 | 0.8 | 0.83 | 0.83 | 0.86 |
| 1.11 | 0.93 | 1.04 | 11.93 | 0.89 | 0.89 | 0.97 | 0.17 | 1.11 | 1.11 | 0.91 |
| 1 | 1.18 | 0.93 | 1.36 | 1 | 1.05 | 1.43 | 0.92 | 1 | 1 | 0.85 |
| 0.9 | 1.56 | 0.74 | 0.8 | 0.97 | 1.23 | 0.98 | 1.01 | 0.9 | 0.9 | 0.82 |
| 1.47 | 1.38 | 0.95 | 1.34 | 0.83 | 1.01 | 0.95 | 0.82 | 1.47 | 1.47 | 0.87 |
| 0.7 | 0.31 | 0.83 | 0.8 | 1.22 | 0.96 | 1 | 9.36 | 0.7 | 0.7 | 0.98 |
| 1.06 | 0.86 | 0.6 | 1.96 | 1.09 | 0.7 | 0.74 | 1.3 | 1.06 | 1.06 | 0.76 |
| 0.4 | 1.21 | 0.66 | 1.04 | 3.67 | 1.24 | 1.15 | 0.5 | 0.4 | 0.4 | 0.91 |
| 1.07 | 0.6 | 0.94 | 1.81 | 1.49 | 1.06 | 1.27 | 0.9 | 1.07 | 1.07 | 0.85 |
| 0.6 | 0.45 | 0.88 | 1.3 | 1.07 | 1.03 | 0.5 | 4.58 | 0.6 | 0.6 | 1.07 |
| 0.56 | 1.06 | 0.71 | 1.8 | 0.68 | 1.08 | 0.64 | 0.99 | 0.56 | 0.56 | 1.88 |
| 0.53 | 1.07 | 0.95 | 2.25 | 0.36 | 0.87 | 0.84 | 1.11 | 0.53 | 0.53 | 0.81 |
| 1.1 | 1.15 | 1.05 | 0.99 | 1.19 | 1.14 | 1.02 | 0.99 | 1.1 | 1.1 | 0.96 |
| 1.04 | 1.1 | 0.77 | 0.7 | 1.77 | 0.4 | 1.02 | 1.39 | 1.04 | 1.04 | 1.18 |
| 1.18 | 2.39 | 1.24 | 0.48 | 1.04 | 1.15 | 0.8 | 0.63 | 1.18 | 1.18 | 1.1 |
| 0.49 | 2 | 1.18 | 0.55 | 0.69 | 1.06 | 1.99 | 1.14 | 0.49 | 0.49 | 1.25 |
| 0.7 | 0.7 | 1.05 | 0.72 | 1.03 | 0.67 | 1.05 | 1.73 | 0.7 | 0.7 | 0.93 |
| 3 | 0.58 | 1.01 | 1.37 | 0.87 | 0.58 | 0.44 | 3.36 | 3 | 3 | 0.7 |
| 0.71 | 0.92 | 1.22 | 1.05 | 0.76 | 1.03 | 0.97 | 2.14 | 0.71 | 0.71 | 0.98 |
| 1.1 | 0.83 | 1.2 | 2.91 | 0.57 | 0.85 | 0.77 | 2.41 | 1.1 | 1.1 | 1.14 |
| 1.81 | 0.42 | 1.53 | 1.5 | 3.95 | 0.63 | 0.73 | 1.58 | 1.81 | 1.81 | 1.38 |

| | | | | | | | | | | |
|---|---|---|---|---|---|---|---|---|---|---|
| 1 | 0.95 | 0.87 | 1.11 | 1.01 | 1.2 | 0.96 | 0.88 | 1 | 1 | 0.94 |
| 0.79 | 1.35 | 0.83 | 4.64 | 1.2 | 1.41 | 1.12 | 0.55 | 0.79 | 0.79 | 1.11 |
| 0.96 | 1.21 | 0.78 | 1.77 | 0.26 | 3.59 | 1.23 | 8.79 | 0.96 | 0.96 | 3.18 |
| 0.96 | 0.65 | 2 | 0.91 | 1.61 | 0.86 | 0.55 | 0.7 | 0.96 | 0.96 | 1.19 |
| 0.97 | 0.49 | 0.92 | 1.34 | 0.83 | 0.94 | 0.71 | 1.01 | 0.97 | 0.97 | 0.86 |
| 1.07 | 0.57 | 0.99 | 0.99 | 1.13 | 0.97 | 1 | 0.73 | 1.07 | 1.07 | 0.89 |
| 1.11 | 1.06 | 1.01 | 1.88 | 0.79 | 0.94 | 1 | 0.91 | 1.11 | 1.11 | 1.06 |
| 1.32 | 1.07 | 1.06 | 0.96 | 1.64 | 0.89 | 0.93 | 1.04 | 1.32 | 1.32 | 1.1 |
| 1.38 | 0.99 | 1.08 | 1.04 | 1.53 | 1.15 | 0.74 | 1.06 | 1.38 | 1.38 | 0.97 |
| 0.9 | 1.59 | 0.98 | 1.08 | 0.83 | 1.04 | 1.07 | 1.45 | 0.9 | 0.9 | 0.76 |
| 1.05 | 1.09 | 1.03 | 1.12 | 0.78 | 1.04 | 0.92 | 0.98 | 1.05 | 1.05 | 0.83 |
| 3.33 | 1.99 | 11.6 | 0.63 | 0.11 | 7.66 | 6.17 | 0.28 | 3.33 | 3.33 | 0.31 |
| 0.87 | 1.49 | 1.13 | 1.02 | 1.44 | 0.96 | 0.79 | 1.07 | 0.87 | 0.87 | 1.14 |
| 0.76 | 0.59 | 0.86 | 0.73 | 0.91 | 1.17 | 0.88 | 1.02 | 0.76 | 0.76 | 0.92 |

| 209341_s_at | 200639_s_at | 207545_s_at | 201533_at | 205192_at | 204726_at | 204372_s_at | 203497_at | 216268_s_at | 206929_s_at | 209341_s_at |
|---|---|---|---|---|---|---|---|---|---|---|
| 1.02 | 1 | 0.95 | 0.96 | 0.95 | 3.75 | 1.01 | 0.92 | 1.13 | 1.02 | 1.02 |
| 1.16 | 1.16 | 1.8 | 1.3 | 0.75 | 0.93 | 1.05 | 0.97 | 0.78 | 0.9 | 1.16 |
| 1.85 | 0.84 | 1.16 | 0.89 | 0.77 | 0.27 | 0.98 | 1.56 | 2.11 | 1.18 | 1.85 |
| 1.14 | 0.93 | 1.59 | 1 | 2.03 | 2.86 | 1.04 | 1.14 | 1.39 | 0.72 | 1.14 |
| 1.51 | 1.17 | 1.09 | 0.96 | 0.88 | 4.6 | 0.87 | 1.05 | 1.42 | 0.65 | 1.51 |
| 0.71 | 1 | 1.21 | 1.29 | 0.77 | 2.19 | 0.93 | 1.02 | 2.41 | 2.64 | 0.71 |
| 1.34 | 0.83 | 1.17 | 0.84 | 1.39 | 2.38 | 1.41 | 1.3 | 0.68 | 1.21 | 1.34 |
| 0.95 | 0.89 | 0.63 | 0.99 | 1.03 | 3.5 | 0.9 | 0.88 | 0.74 | 0.67 | 0.95 |
| 1 | 0.95 | 1.04 | 0.98 | 0.85 | 0.49 | 0.87 | 1.09 | 0.44 | 0.7 | 1 |
| 0.75 | 1.13 | 1.13 | 1.05 | 1.57 | 8.25 | 0.89 | 0.97 | 0.77 | 0.74 | 0.75 |
| 1.27 | 0.88 | 1 | 1.02 | 1.44 | 0.14 | 0.96 | 1.1 | 1.65 | 1.06 | 1.27 |
| 1.1 | 0.99 | 0.95 | 0.95 | 1.3 | 1.67 | 1.15 | 0.88 | 0.96 | 0.96 | 1.1 |
| 1.16 | 1.16 | 1.08 | 0.95 | 1.27 | 0.52 | 1.22 | 0.89 | 1.26 | 1.13 | 1.16 |
| 0.26 | 0.54 | 0.16 | 15.7 | 2.46 | 26.35 | 0.06 | 0.43 | 5.98 | 2.04 | 0.26 |
| 1.47 | 1.13 | 1.15 | 0.75 | 1.41 | 1.63 | 0.56 | 0.35 | 1.29 | 0.44 | 1.47 |
| 1.21 | 0.94 | 1.26 | 1.18 | 1.26 | 0.76 | 0.86 | 0.83 | 0.52 | 0.57 | 1.21 |
| 1.25 | 1.14 | 1.33 | 0.95 | 1.37 | 0.67 | 1.12 | 0.89 | 0.71 | 1.14 | 1.25 |
| 1.05 | 1.12 | 1.06 | 1.15 | 0.85 | 1.63 | 1.04 | 1.23 | 0.81 | 1.2 | 1.05 |
| 1.32 | 0.89 | 1.38 | 1.15 | 0.31 | 1.75 | 0.85 | 0.95 | 7.3 | 0.79 | 1.32 |
| 1.39 | 1.01 | 2.17 | 0.68 | 0.2 | 5.45 | 0.61 | 1 | 1.45 | 0.68 | 1.39 |
| 0.49 | 1.01 | 0.4 | 3.6 | 1.11 | 2.67 | 0.94 | 0.91 | 0.48 | 1.55 | 0.49 |
| 0.93 | 0.82 | 0.58 | 0.82 | 2.13 | 27.12 | 0.78 | 0.46 | 1.13 | 1.04 | 0.93 |
| 1.07 | 0.96 | 0.74 | 1 | 0.9 | 1.11 | 0.91 | 0.82 | 0.98 | 0.81 | 1.07 |
| 1.21 | 0.9 | 1.97 | 0.64 | 1.55 | 0.5 | 1.06 | 0.98 | 0.72 | 1.27 | 1.21 |
| 1.79 | 1.43 | 1.15 | 0.74 | 5.08 | 19.52 | 0.55 | 0.59 | 0.03 | 0.81 | 1.79 |
| 0.85 | 0.74 | 1.39 | 1.03 | 1.79 | 2.2 | 1.23 | 0.72 | 1.32 | 1.53 | 0.85 |
| 1.14 | 1.16 | 0.39 | 0.85 | 1.07 | 1.88 | 1.02 | 0.86 | 0.28 | 1.33 | 1.14 |
| 0.97 | 1.78 | 1.18 | 0.38 | 1.17 | 0.57 | 0.71 | 0.7 | 0.43 | 0.7 | 0.97 |
| 0.5 | 0.75 | 0.77 | 1 | 1.57 | 0.27 | 1.34 | 1 | 3.37 | 0.91 | 0.5 |
| 0.83 | 0.92 | 0.98 | 1.06 | 1.12 | 0.75 | 0.95 | 1.09 | 2.11 | 1.01 | 0.83 |

| | | | | | | | | | | |
|---|---|---|---|---|---|---|---|---|---|---|
| 0.69 | 1.09 | 0.71 | 0.98 | 0.88 | 1.22 | 1.13 | 1.18 | 1.49 | 1.09 | 0.69 |
| 3.19 | 3.68 | 1.15 | 1.31 | 1.48 | 8.17 | 7.18 | 0.94 | 0.09 | 2.75 | 3.19 |
| 1.26 | 0.98 | 0.99 | 1.12 | 1.35 | 2.83 | 1.01 | 1.29 | 1.32 | 1.02 | 1.26 |
| 0.76 | 0.64 | 0.6 | 1.14 | 0.39 | 0.48 | 1.1 | 0.59 | 0.53 | 0.92 | 0.76 |
| 2.42 | 0.96 | 3.2 | 2.17 | 1.15 | 0.93 | 0.07 | 6.02 | 5 | 0.96 | 2.42 |
| 0.78 | 0.8 | 1.23 | 1.14 | 0.76 | 1.05 | 1.45 | 0.69 | 1 | 4.58 | 0.78 |
| 0.93 | 1.16 | 0.46 | 0.97 | 1.31 | 0.02 | 0.81 | 0.76 | 0.86 | 0.44 | 0.93 |
| 0.78 | 1.12 | 0.83 | 1.03 | 0.89 | 0.97 | 0.95 | 0.89 | 1.14 | 0.67 | 0.78 |
| 0.89 | 1.02 | 0.93 | 1.13 | 1.21 | 0.74 | 0.91 | 0.99 | 1.33 | 0.56 | 0.89 |
| 1.88 | 1.08 | 1.25 | 0.91 | 1.43 | 1.5 | 0.83 | 1.36 | 3.11 | 1.12 | 1.88 |
| 1.12 | 1.12 | 0.96 | 0.9 | 1.04 | 3.5 | 1.08 | 1.1 | 0.6 | 1.3 | 1.12 |
| 0.95 | 1.07 | 0.87 | 0.97 | 0.88 | 0.56 | 0.98 | 0.98 | 0.67 | 1.07 | 0.95 |
| 1.41 | 0.92 | 4.99 | 1.06 | 0.59 | 1.32 | 0.48 | 0.87 | 0.91 | 0.4 | 1.41 |
| 1.49 | 1.17 | 1.75 | 1.04 | 1.42 | 1.41 | 0.68 | 0.92 | 3.44 | 0.21 | 1.49 |
| 1.22 | 0.91 | 3.31 | 1.13 | 0.76 | 1.26 | 0.48 | 0.89 | 1.03 | 0.45 | 1.22 |
| 1.59 | 1.37 | 1.58 | 1.28 | 0.95 | 2.57 | 0.97 | 1.19 | 1.32 | 1.2 | 1.59 |
| 1.14 | 0.94 | 1.28 | 0.94 | 1.81 | 0 | 1.02 | 0.93 | 1.07 | 0.92 | 1.14 |
| 1.1 | 0.97 | 1.09 | 1 | 1.47 | 3.11 | 1.09 | 0.99 | 0.86 | 1.05 | 1.1 |
| 1.06 | 0.94 | 1.04 | 0.95 | 1.03 | 4.5 | 1.01 | 1.01 | 1.07 | 1.06 | 1.06 |
| 0.89 | 1.02 | 2.14 | 0.83 | 0.81 | 1.33 | 1.42 | 1 | 0.53 | 1.45 | 0.89 |
| 0.81 | 1.07 | 0.92 | 0.93 | 1.45 | 1 | 1.1 | 0.9 | 0.57 | 1.05 | 0.81 |
| 1.06 | 0.83 | 0.75 | 0.94 | 1.53 | 0.75 | 1.02 | 1.08 | 0.86 | 1.09 | 1.06 |
| 0.7 | 0.99 | 1.07 | 1.08 | 1.04 | 6.34 | 1.11 | 0.98 | 0.83 | 0.66 | 0.7 |
| 1.13 | 1.19 | 1.05 | 0.93 | 1.11 | 0.75 | 1.04 | 1.05 | 1.2 | 1.43 | 1.13 |
| 0.66 | 1.06 | 1.93 | 1.03 | 0.52 | 1.42 | 0.99 | 1.44 | 1.84 | 0.69 | 0.66 |
| 1.16 | 0.55 | 0.58 | 1.16 | 1.15 | 0.59 | 1.42 | 1.03 | 0.82 | 1.21 | 1.16 |
| 1.15 | 0.76 | 2.69 | 0.71 | 1.04 | 1.56 | 0.93 | 0.79 | 4.86 | 1.07 | 1.15 |
| 2.16 | 0.56 | 0.85 | 0.59 | 1.38 | Inf | 1.42 | 1.75 | 5.96 | 2.39 | 2.16 |
| 1.43 | 1.02 | 1.08 | 1.08 | 1.09 | 1 | 0.91 | 0.91 | 0.94 | 0.81 | 1.43 |
| 1.22 | 0.82 | 4.6 | 1.13 | 0.87 | 1.07 | 0.52 | 0.98 | 0.69 | 0.32 | 1.22 |
| 0.92 | 1.22 | 1.07 | 1.28 | 0.68 | 5.55 | 1.04 | 0.96 | 1.2 | 1.81 | 0.92 |
| 1.61 | 0.88 | 0.91 | 1.62 | 1.54 | 24 | 1.07 | 1.17 | 1.93 | 1.52 | 1.61 |
| 1.02 | 0.89 | 0.94 | 1.03 | 1.3 | 1.87 | 0.91 | 0.86 | 103.29 | 0.89 | 1.02 |

| | | | | | | | | | | |
|---|---|---|---|---|---|---|---|---|---|---|
| 1.04 | 1.35 | 1.2 | 0.93 | 0.58 | 0.62 | 1.14 | 1.01 | 0.6 | 1.27 | 1.04 |
| 0.99 | 1.05 | 0.98 | 1.4 | 1.02 | 1.51 | 1.25 | 1.17 | 3.07 | 0.96 | 0.99 |
| 1 | 1.13 | 1.02 | 1.01 | 1.07 | 0.79 | 1.12 | 0.86 | 0.98 | 1.03 | 1 |
| 0.88 | 1.05 | 1.34 | 1.3 | 1.48 | 10.47 | 1.24 | 1.05 | 1.56 | 1.18 | 0.88 |
| 1.21 | 0.84 | 1.11 | 1.03 | 0.98 | 1.32 | 1.01 | 1.4 | 1.2 | 1.02 | 1.21 |
| 1.65 | 0.83 | 1.7 | 0.95 | 0.39 | 4.69 | 0.95 | 0.96 | 1.28 | 1.1 | 1.65 |
| 0.6 | 1.04 | 0.95 | 1.59 | 1.36 | 1.28 | 1.28 | 1.55 | 0.26 | 0.62 | 0.6 |
| 1.26 | 0.95 | 0.9 | 1.07 | 1.14 | 0.93 | 0.99 | 0.85 | 0.89 | 1.59 | 1.26 |
| 6.85 | 0.96 | 1.05 | 0.99 | 0.46 | 3.01 | 1.07 | 0.52 | 1.72 | 0.17 | 6.85 |
| 1.61 | 1.12 | 1.37 | 1.26 | 0.89 | 1.75 | 1.13 | 1.49 | 4.02 | 0.84 | 1.61 |
| 0.5 | 1.1 | 0.91 | 0.76 | 1.06 | 2.48 | 1.36 | 0.76 | 0.89 | 1.86 | 0.5 |
| 1.07 | 1.06 | 1.1 | 0.68 | 1.9 | 2 | 1.02 | 1.14 | 3.04 | 1.14 | 1.07 |
| 1.35 | 1.05 | 0.98 | 1.2 | 1.3 | 0.6 | 0.79 | 1.03 | 1.09 | 1.09 | 1.35 |
| 0.99 | 1 | 2.07 | 1.41 | 0.68 | 2.62 | 1 | 1.39 | 2.64 | 1.3 | 0.99 |
| 0.87 | 0.91 | 0.75 | 1.17 | 1.44 | 0.33 | 1.06 | 1.11 | 1.13 | 0.97 | 0.87 |
| 9.99 | 36.43 | 1.17 | 0.33 | 0.71 | 1.28 | 46.84 | 4.95 | 3.08 | 0.58 | 9.99 |
| 1.44 | 1.11 | 1.33 | 0.81 | 0.99 | 3.37 | 1.04 | 1.05 | 0.73 | 0.83 | 1.44 |
| 1.29 | 1.25 | 0.88 | 3.21 | 0.42 | 0.31 | 1.64 | 0.73 | 3.8 | 0.48 | 1.29 |
| 1.21 | 1.32 | 1.89 | 1.19 | 1.29 | 1.76 | 1.36 | 1.03 | 1.16 | 1.38 | 1.21 |
| 1.04 | 0.91 | 1.16 | 1.26 | 2.58 | 2.75 | 2.04 | 1.89 | 0.75 | 12.44 | 1.04 |
| 1.25 | 1.45 | 1.96 | 2.81 | 0.79 | 2.25 | 2.09 | 0.84 | 1.25 | 3.37 | 1.25 |
| 1.2 | 1.11 | 0.72 | 0.83 | 1.77 | 1.07 | 0.95 | 0.97 | 1.22 | 0.83 | 1.2 |
| 0.93 | 1.08 | 1.12 | 1.04 | 1.21 | 1.56 | 1.09 | 1.16 | 0.72 | 1.09 | 0.93 |
| 0.8 | 1.06 | 0.69 | 1 | 0.75 | 0.97 | 1.23 | 0.71 | 0.82 | 1.44 | 0.8 |
| 1.17 | 1.05 | 1.06 | 1.01 | 0.95 | 1.27 | 0.91 | 0.87 | 0.8 | 0.99 | 1.17 |
| 0.9 | 0.99 | 0.92 | 0.91 | 1.4 | 0.5 | 0.68 | 0.79 | 0.69 | 0.93 | 0.9 |
| 0.99 | 0.84 | 0.78 | 1 | 1.23 | NA | 1.02 | 0.84 | 0.93 | 1.05 | 0.99 |
| 1 | 0.97 | 1.12 | 1.12 | 0.95 | 8.53 | 1.01 | 1 | 0.86 | 1.19 | 1 |
| 0.79 | 1.08 | 1.29 | 1.09 | 0.76 | 7.12 | 1.12 | 1.04 | 1.54 | 0.96 | 0.79 |
| 0.89 | 0.96 | 0.94 | 0.84 | 1.47 | 2.11 | 1.1 | 1.08 | 0.85 | 0.81 | 0.89 |
| 1.07 | 0.97 | 1.23 | 1.01 | 1.09 | 2.22 | 1.04 | 1.12 | 1.29 | 0.96 | 1.07 |
| 0.73 | 1.14 | 0.74 | 0.99 | 1.01 | 1.45 | 0.6 | 0.86 | 0.46 | 0.81 | 0.73 |
| 1.39 | 1.15 | 1.64 | 1.23 | 1.01 | 2.25 | 1.01 | 1.14 | 4.81 | 1.53 | 1.39 |

| | | | | | | | | | | |
|---|---|---|---|---|---|---|---|---|---|---|
| 1.31 | 1.08 | 0.71 | 0.95 | 0.7 | 1.6 | 1.1 | 1.43 | 0.79 | 0.8 | 1.31 |
| 0.83 | 1.05 | 1.03 | 0.94 | 0.99 | 0.85 | 1.01 | 0.96 | 1.02 | 0.76 | 0.83 |
| 0.75 | 0.58 | 0.72 | 0.65 | 0.77 | 0.88 | 1.17 | 1.03 | 0.73 | 1.3 | 0.75 |
| 4.71 | 0.83 | 0.93 | 0.7 | 0.73 | 3.25 | 1.61 | 0.7 | 1.42 | 0.81 | 4.71 |
| 1.72 | 1.56 | 1.4 | 1.18 | 1.84 | 0.81 | 0.87 | 0.99 | 1.41 | 1 | 1.72 |
| 0.5 | 1.07 | 0.16 | 0.68 | 1.65 | 4.42 | 0.69 | 0.34 | 0.43 | 4.74 | 0.5 |
| 1.47 | 0.97 | 1.25 | 0.84 | 2.22 | 1.23 | 0.79 | 1.15 | 1.12 | 0.91 | 1.47 |
| 0.94 | 1.07 | 1.33 | 1.08 | 0.69 | 5.42 | 1.23 | 0.67 | 0.69 | 1.04 | 0.94 |
| 0.57 | 0.99 | 0.84 | 0.85 | 1.72 | 2.52 | 0.9 | 0.9 | 4.05 | 0.76 | 0.57 |
| 1.01 | 1.3 | 1.38 | 0.98 | 1.38 | 4.38 | 1.22 | 0.81 | 0.71 | 1.35 | 1.01 |
| 0.91 | 1.03 | 1.05 | 1.05 | 1.43 | 3.99 | 1.08 | 1.08 | 0.91 | 1.32 | 0.91 |
| 1.14 | 0.95 | 0.9 | 1.05 | 1.4 | 3.61 | 0.98 | 0.95 | 0.76 | 0.93 | 1.14 |
| 0.73 | 1.17 | 0.87 | 0.87 | 0.55 | 0.96 | 1.14 | 1.17 | 2.05 | 0.88 | 0.73 |
| 0.87 | 0.81 | 0.91 | 1.4 | 0.7 | 0.69 | 1.08 | 0.83 | 0.87 | 1.1 | 0.87 |
| 1.26 | 1.28 | 1.26 | 1.31 | 1.3 | 1.58 | 1.08 | 1.12 | 2.8 | 1.06 | 1.26 |
| 1.24 | 1 | 1.09 | 1.68 | 3.06 | 2.33 | 1.06 | 1.12 | 0.69 | 0.85 | 1.24 |
| 0.92 | 0.99 | 0.89 | 0.89 | 1.59 | 0.3 | 1 | 0.99 | 4.69 | 0.12 | 0.92 |
| 1.19 | 1.09 | 1.22 | 1.13 | 1.18 | 9.16 | 1.25 | 1.09 | 1.66 | 1.16 | 1.19 |
| 1.2 | 0.86 | 0.87 | 0.98 | 1.31 | 1.3 | 0.94 | 0.83 | 0.69 | 0.57 | 1.2 |
| 0.7 | 1.03 | 1.77 | 1.12 | 0.87 | 1 | 1.11 | 1.04 | 0.99 | 0.57 | 0.7 |
| 0.98 | 1.07 | 1.79 | 1.37 | 0.7 | 1.77 | 1.37 | 1.66 | 1.34 | 0.95 | 0.98 |
| 0.71 | 0.94 | 0.88 | 1 | 1.32 | 3.49 | 0.97 | 1.06 | 1.46 | 0.79 | 0.71 |
| 1.1 | 0.97 | 1.2 | 1.38 | 0.97 | 1.19 | 1.18 | 1.15 | 0.74 | 0.86 | 1.1 |
| 1.86 | 0.85 | 1.47 | 1.37 | 1.73 | 3.12 | 1.81 | 2.16 | 0.4 | 1.36 | 1.86 |
| 0.88 | 1.23 | 0.5 | 0.9 | 0.93 | 4.27 | 0.94 | 0.68 | 1.02 | 2.27 | 0.88 |
| 1.08 | 1.19 | 1.46 | 0.9 | 0.85 | 3.25 | 0.83 | 0.86 | 1.12 | 0.49 | 1.08 |
| 1.29 | 0.93 | 0.97 | 1.09 | 0.96 | 0.17 | 1.02 | 0.93 | 2.32 | 0.92 | 1.29 |
| 1.33 | 0.67 | 1.11 | 0.95 | 0.76 | 1.46 | 0.85 | 1.04 | 2.3 | 1.17 | 1.33 |
| 0.79 | 0.87 | 0.97 | 1.03 | 1.2 | 0.73 | 1.45 | 0.66 | 0.49 | 0.96 | 0.79 |
| 0.82 | 1.01 | 1.22 | 1.2 | 0.82 | 0.93 | 1.09 | 0.94 | 2.58 | 1.03 | 0.82 |
| 0.84 | 1.05 | 1 | 0.77 | 1.08 | 2.75 | 1 | 0.98 | 3.81 | 0.8 | 0.84 |
| 0.99 | 1.05 | 0.98 | 1.4 | 1.02 | 1.51 | 1.25 | 1.17 | 3.07 | 0.96 | 0.99 |
| 1.09 | 0.89 | 1.55 | 0.99 | 0.8 | 2 | 0.94 | 1.11 | 1.25 | 0.85 | 1.09 |

| | | | | | | | | | | |
|---|---|---|---|---|---|---|---|---|---|---|
| 0.89 | 1.09 | 1.05 | 1.05 | 1.27 | 0.25 | 0.96 | 0.92 | 0.51 | 0.78 | 0.89 |
| 0.82 | 0.77 | 1.09 | 1.05 | 1.81 | 3.37 | 0.86 | 1.18 | 1.02 | 1.22 | 0.82 |
| 1.09 | 0.72 | 1.09 | 1.08 | 2.55 | 1.08 | 0.86 | 0.93 | 0.94 | 1.08 | 1.09 |
| 0.91 | 1.1 | 0.89 | 0.85 | 1.08 | 2.92 | 0.88 | 1.14 | 0.99 | 0.79 | 0.91 |
| 0.98 | 0.92 | 0.72 | 0.83 | 1.74 | 1.08 | 0.89 | 0.9 | 1.37 | 1.06 | 0.98 |
| 0.9 | 0.87 | 0.91 | 0.89 | 0.93 | 1.15 | 1.08 | 0.94 | 0.95 | 1.16 | 0.9 |
| 1 | 0.85 | 1.13 | 1.07 | 1.09 | 1.11 | 1.04 | 1.07 | 0.93 | 0.87 | 1 |
| 0.92 | 1.24 | 1.5 | 0.76 | 1.4 | 1.58 | 1.92 | 1.05 | 1.06 | 0.72 | 0.92 |
| 1.23 | 0.84 | 1.57 | 0.77 | 0.96 | 0.87 | 1.24 | 1.27 | 0.38 | 1.06 | 1.23 |
| 1.06 | 1.38 | 1.08 | 1.24 | 1.12 | 0.25 | 1.38 | 0.95 | 1.88 | 0.99 | 1.06 |
| 1.14 | 1.03 | 1 | 1.01 | 0.66 | 0.53 | 1.05 | 0.94 | 0.3 | 0.94 | 1.14 |
| 1.06 | 1.17 | 0.85 | 1.11 | 0.73 | 0.71 | 0.99 | 1.12 | 1.37 | 1.17 | 1.06 |
| 1.07 | 1.01 | 1.23 | 1.14 | 1.02 | 8.5 | 1.19 | 1 | 1.63 | 1.24 | 1.07 |
| 1.09 | 0.71 | 1.32 | 0.89 | 1.18 | 3.33 | 0.81 | 0.99 | 0.86 | 0.96 | 1.09 |
| 1.38 | 1.33 | 1.51 | 2.37 | 0.44 | 1.56 | 1.52 | 0.77 | 2.11 | 1.21 | 1.38 |
| 1.1 | 1.22 | 1.3 | 0.89 | 1.01 | 1.62 | 1.03 | 0.91 | 0.88 | 1.27 | 1.1 |
| 0.91 | 0.89 | 1.27 | 1.14 | 1.81 | 1.36 | 0.95 | 0.97 | 0.98 | 1.06 | 0.91 |
| 0.9 | 1.12 | 0.75 | 0.86 | 0.97 | 1.8 | 1.36 | 1.29 | 2.5 | 1.45 | 0.9 |
| 0.96 | 1.24 | 0.93 | 0.94 | 1.15 | 5.33 | 1.02 | 0.93 | 0.68 | 1.05 | 0.96 |
| 0.84 | 0.9 | 0.87 | 1 | 0.96 | 2.56 | 0.96 | 0.69 | 0.78 | 0.82 | 0.84 |
| 1.04 | 1.03 | 0.93 | 1.17 | 1.34 | 6 | 1 | 1.37 | 0.95 | 1.13 | 1.04 |
| 1.21 | 1.38 | 0.97 | 0.89 | 0.95 | 0.12 | 1.31 | 0.91 | 1.29 | 0.58 | 1.21 |
| 1.06 | 1.02 | 1.11 | 0.97 | 1.02 | 4.8 | 0.99 | 1.18 | 1.15 | 0.94 | 1.06 |
| 0.94 | 0.76 | 0.91 | 1.38 | 1.05 | 0.69 | 0.85 | 1 | 1.01 | 0.98 | 0.94 |
| 1.38 | 1.14 | 0.72 | 0.91 | 0.91 | 0.98 | 0.73 | 0.82 | 0.68 | 0.82 | 1.38 |
| 1.15 | 0.61 | 0.97 | 1.05 | 1.78 | 0.88 | 1.14 | 1.07 | 0.71 | 1.38 | 1.15 |
| 1.29 | 1.15 | 1.32 | 0.78 | 0.72 | 0.53 | 1.24 | 1.29 | 0.59 | 0.46 | 1.29 |
| 0.82 | 1.09 | 1.13 | 1.04 | 0.95 | 0.61 | 0.88 | 1.29 | 0.87 | 1.09 | 0.82 |
| 1.07 | 0.94 | 1.23 | 1.04 | 1.24 | Inf | 0.97 | 1 | 0.97 | 1.09 | 1.07 |
| 1.19 | 1.06 | 1.11 | 0.95 | 1.08 | 0 | 0.88 | 1.08 | 0.88 | 0.81 | 1.19 |
| 0.81 | 0.98 | 1.17 | 1.01 | 0.96 | 6.8 | 1.31 | 1.34 | 0.75 | 1.05 | 0.81 |
| 1.01 | 1.05 | 1.02 | 0.97 | 1.44 | 1.19 | 1.03 | 0.91 | 1.07 | 1.01 | 1.01 |
| 1.1 | 1.05 | 1.09 | 0.97 | 1.07 | 1.07 | 1.03 | 0.93 | 0.87 | 0.91 | 1.1 |

| | | | | | | | | | | |
|---|---|---|---|---|---|---|---|---|---|---|
| 6.69 | 1.05 | 0.49 | 1.19 | 0.92 | 0.48 | 1.47 | 0.48 | 1.73 | 0.56 | 6.69 |
| 0.82 | 1.05 | 1.08 | 0.91 | 0.9 | 2.86 | 1.13 | 1.17 | 1.33 | 1 | 0.82 |
| 1.01 | 0.92 | 0.98 | 0.92 | 1.08 | 0.88 | 1.09 | 1.01 | 0.84 | 0.94 | 1.01 |
| 1.19 | 1.48 | 1.52 | 1.3 | 0.91 | 2.85 | 0.86 | 1.07 | 0.92 | 0.64 | 1.19 |
| 0.51 | 0.89 | 0.42 | 0.82 | 0.85 | 6.83 | 1.02 | 0.99 | 0.68 | 0.77 | 0.51 |
| 0.99 | 0.95 | 0.99 | 1.18 | 0.97 | 1.19 | 0.99 | 0.95 | 1.07 | 0.98 | 0.99 |
| 0.7 | 1.02 | 0.67 | 0.97 | 0.76 | 0.89 | 1.1 | 1.16 | 1.44 | 1.05 | 0.7 |
| 0.93 | 0.87 | 0.72 | 0.94 | 1.34 | 1.17 | 0.94 | 1.02 | 0.74 | 0.86 | 0.93 |
| 0.94 | 1.15 | 0.89 | 1.2 | 1.28 | 0.67 | 1.11 | 1.06 | 1.04 | 0.96 | 0.94 |
| 0.68 | 1.11 | 0.57 | 1.12 | 0.68 | 3.56 | 1.18 | 1.32 | 1.22 | 1.22 | 0.68 |
| 0.73 | 1.06 | 0.84 | 1.01 | 0.84 | 2.5 | 1.34 | 1.05 | 1.61 | 1.07 | 0.73 |
| 0.83 | 1.27 | 2 | 1.1 | 0.9 | 2.07 | 1.75 | 1.28 | 0.52 | 0.76 | 0.83 |
| 0.92 | 0.98 | 0.78 | 0.96 | 1.05 | Inf | 0.92 | 0.96 | 1.54 | 0.83 | 0.92 |
| 0.91 | 1.01 | 0.98 | 0.92 | 1.08 | 2.83 | 0.93 | 0.87 | 0.44 | 1.18 | 0.91 |
| 0.92 | 0.9 | 0.99 | 0.82 | 0.94 | 1.11 | 0.92 | 1.15 | 0.95 | 0.84 | 0.92 |
| 1.06 | 0.94 | 0.87 | 1.16 | 0.81 | 1.37 | 0.93 | 0.92 | 0.87 | 3.77 | 1.06 |
| 1.19 | 1.07 | 1.04 | 0.87 | 1.17 | 2.55 | 0.96 | 0.98 | 1.4 | 1.26 | 1.19 |
| 0.78 | 0.95 | 0.95 | 0.98 | 0.81 | 2.64 | 0.95 | 0.99 | 0.98 | 0.88 | 0.78 |
| 1.21 | 0.8 | 0.86 | 0.99 | 0.84 | 1.71 | 0.92 | 1.11 | 1.34 | 0.61 | 1.21 |
| 1.12 | 0.82 | 0.94 | 0.96 | 0.55 | 1.36 | 0.73 | 1.13 | 1.25 | 0.84 | 1.12 |
| 1 | 1.12 | 1.02 | 1.32 | 1.11 | 7.67 | 1.26 | 0.88 | 0.97 | 1.11 | 1 |
| 0.57 | 0.71 | 0.5 | 2.54 | 1.71 | 0.13 | 0.8 | 0.82 | 4.82 | 0.73 | 0.57 |
| 0.96 | 0.95 | 1.06 | 0.87 | 1.43 | 0.92 | 0.93 | 1 | 0.81 | 0.7 | 0.96 |
| 3.2 | 0.87 | 0.63 | 0.98 | 0.62 | 1.08 | 1.1 | 0.66 | 1.98 | 0.41 | 3.2 |
| 1.13 | 0.95 | 0.92 | 1.07 | 0.81 | 1.12 | 0.8 | 0.88 | 1 | 0.83 | 1.13 |
| 1.07 | 0.86 | 0.77 | 0.47 | 0.94 | 1.83 | 0.9 | 1.17 | 63.43 | 1.25 | 1.07 |
| 0.87 | 1.1 | 1.38 | 1.03 | 0.97 | 1 | 1.08 | 1.28 | 1.24 | 0.96 | 0.87 |
| 0.95 | 1.07 | 0.9 | 0.95 | 1.1 | 2.65 | 1 | 0.95 | 1.16 | 0.75 | 0.95 |
| 1.12 | 0.93 | 1.08 | 0.99 | 1.09 | Inf | 1.12 | 1.05 | 0.84 | 1.48 | 1.12 |
| 0.84 | 1.05 | 0.79 | 1.06 | 1.06 | 0.35 | 0.96 | 1.05 | 3.7 | 1.2 | 0.84 |
| 1.16 | 0.75 | 1.23 | 0.82 | 1.6 | 0.71 | 0.88 | 0.86 | 0.77 | 0.91 | 1.16 |
| 0.84 | 1.08 | 0.76 | 0.99 | 0.99 | 1 | 1.01 | 0.89 | 1.09 | 0.84 | 0.84 |
| 1 | 0.93 | 0.67 | 1.05 | 1.6 | 0.88 | 1.01 | 1.32 | 0.53 | 0.94 | 1 |

| | | | | | | | | | | |
|---|---|---|---|---|---|---|---|---|---|---|
| 1.18 | 0.99 | 1.53 | 1.07 | 1.27 | 3.5 | 1.08 | 0.94 | 1.45 | 0.98 | 1.18 |
| 0.94 | 0.81 | 0.97 | 1.02 | 0.87 | 0.5 | 0.93 | 1.51 | 0.8 | 1.24 | 0.94 |
| 0.83 | 1.25 | 0.94 | 0.94 | 0.9 | 0.8 | 1.06 | 1.07 | 1.2 | 0.76 | 0.83 |
| 0.62 | 0.85 | 0.77 | 1.55 | 0.75 | 1.12 | 0.86 | 1.65 | 1.12 | 0.64 | 0.62 |
| 0.93 | 0.91 | 0.9 | 1 | 1.14 | 1.91 | 1.14 | 1.14 | 1.1 | 1.33 | 0.93 |
| 0.94 | 0.99 | 1.07 | 1.08 | 0.74 | 0.7 | 1.01 | 0.97 | 0.89 | 0.77 | 0.94 |
| 0.99 | 1.27 | 1.41 | 1.52 | 1.22 | 1.8 | 0.7 | 0.56 | 0.56 | 0.59 | 0.99 |
| 0.8 | 0.99 | 1.28 | 1.05 | 1.04 | 2.85 | 0.99 | 1.08 | 0.26 | 0.56 | 0.8 |
| 3.39 | 1.06 | 0.47 | 0.9 | 0.77 | 0.46 | 0.97 | 0.55 | 1.04 | 0.68 | 3.39 |
| 0.94 | 1.29 | 1.49 | 1.13 | 0.88 | 1.34 | 0.73 | 1.09 | 0.57 | 0.75 | 0.94 |
| 0.77 | 1.03 | 1.07 | 0.99 | 0.58 | 1 | 0.84 | 0.92 | 0.92 | 0.84 | 0.77 |
| 0.83 | 1.05 | 1.26 | 0.99 | 1.12 | 2 | 1.01 | 1.11 | 0.67 | 0.85 | 0.83 |
| 0.76 | 1.16 | 0.84 | 1.15 | 1.46 | 2 | 1.2 | 0.9 | 1.07 | 0.9 | 0.76 |
| 0.96 | 1.06 | 1.34 | 0.8 | 0.93 | 1.58 | 1.03 | 0.92 | 2.08 | 0.94 | 0.96 |
| 0.91 | 1.08 | 1.03 | 1.23 | 0.72 | 9.69 | 0.8 | 0.79 | 1.1 | 0.89 | 0.91 |
| 0.67 | 0.83 | 0.63 | 0.96 | 0.75 | 0.42 | 1.01 | 1.17 | 2.29 | 0.91 | 0.67 |
| 0.74 | 1.06 | 0.95 | 0.96 | 1.24 | 0.46 | 1.34 | 1.04 | 1.26 | 0.95 | 0.74 |
| 0.84 | 0.97 | 0.93 | 1.07 | 0.84 | 3.7 | 0.95 | 0.9 | 0.52 | 1.15 | 0.84 |
| 2.21 | 0.62 | 0.82 | 1.2 | 1.83 | 12 | 0.87 | 1.25 | 0.73 | 2.12 | 2.21 |
| 0.76 | 0.67 | 0.71 | 0.96 | 4.06 | 0.52 | 0.59 | 0.98 | 0.14 | 1.9 | 0.76 |
| 1.33 | 0.75 | 0.78 | 0.88 | 1.37 | 2.93 | 1.28 | 0.72 | 1.77 | 1.26 | 1.33 |
| 0.68 | 1.14 | 0.66 | 1.05 | 0.84 | 2.56 | 1.17 | 1.26 | 1.38 | 1.18 | 0.68 |
| 0.93 | 0.49 | 1 | 0.95 | 1.03 | 0.47 | 1.33 | 1.22 | 0.64 | 0.64 | 0.93 |
| 0.84 | 1.02 | 1 | 0.83 | 0.35 | 0.37 | 0.78 | 0.66 | 6.33 | 0.76 | 0.84 |
| 1.24 | 1.07 | 1.03 | 0.95 | 1.38 | 2.25 | 0.92 | 1.01 | 0.75 | 1.04 | 1.24 |
| 1.33 | 0.93 | 0.89 | 1.09 | 1.61 | 5.67 | 1.19 | 0.74 | 0.92 | 1.3 | 1.33 |
| 0.82 | 0.95 | 1.06 | 0.86 | 0.54 | 0.71 | 1.28 | 1.34 | 2.33 | 1.44 | 0.82 |
| 1.14 | 0.82 | 1.47 | 0.72 | 1.37 | 2.81 | 1 | 0.96 | 0.92 | 2.23 | 1.14 |
| 0.75 | 1.05 | 1.46 | 1.75 | 1.65 | 0.54 | 0.8 | 0.78 | 1.15 | 0.83 | 0.75 |
| 1.99 | 0.85 | 0.22 | 1.02 | 1.24 | 2.22 | 0.5 | 0.56 | 2.11 | 0.19 | 1.99 |
| 1.27 | 0.74 | 1 | 1.1 | 0.89 | 1.5 | 0.97 | 0.93 | 1.34 | 1.42 | 1.27 |
| 0.94 | 1.33 | 2.35 | 1.34 | 3.64 | 1.01 | 0.8 | 0.97 | 2.76 | 1.52 | 0.94 |
| 1.4 | 1.06 | 1.95 | 2.26 | 3.66 | 2.5 | 2.34 | 1.15 | 2.71 | 1.81 | 1.4 |

| | | | | | | | | | | |
|---|---|---|---|---|---|---|---|---|---|---|
| 1.07 | 1.05 | 0.85 | 0.87 | 0.92 | 1.39 | 0.96 | 0.9 | 0.83 | 1.15 | 1.07 |
| 1.06 | 1.1 | 0.92 | 0.93 | 0.94 | 0.94 | 0.94 | 1.01 | 0.72 | 0.88 | 1.06 |
| 2.45 | 6.31 | 5.96 | 3.93 | 0.43 | 0.92 | 5.63 | 2.47 | 123.5 | 0.82 | 2.45 |
| 0.59 | 0.75 | 0.59 | 1.2 | 0.71 | 0.97 | 1.23 | 1.04 | 0.61 | 0.66 | 0.59 |
| 0.73 | 1.07 | 0.89 | 0.74 | 1.34 | 1.6 | 0.85 | 0.76 | 0.96 | 0.8 | 0.73 |
| 1.28 | 1.1 | 0.99 | 1.12 | 0.81 | 1.04 | 1.14 | 1.09 | 0.83 | 1.02 | 1.28 |
| 1.25 | 1.03 | 1.1 | 1.07 | 1.13 | 1.42 | 1.01 | 0.97 | 1.37 | 1.17 | 1.25 |
| 1.07 | 0.96 | 1.15 | 0.84 | 1.16 | 0.31 | 1.11 | 1.01 | 0.67 | 0.79 | 1.07 |
| 1.24 | 0.92 | 0.89 | 0.77 | 1.83 | 7.91 | 0.94 | 1.15 | 1.06 | 1.09 | 1.24 |
| 1.08 | 0.79 | 1.18 | 1.27 | 1 | 5.2 | 1.15 | 1.02 | 1.11 | 0.95 | 1.08 |
| 1.25 | 1.07 | 1.12 | 0.97 | 0.62 | 2.82 | 1.03 | 1.01 | 0.51 | 0.94 | 1.25 |
| 1.69 | 79.4 | 3.52 | 15.01 | 5.63 | 0.44 | 67.85 | 1.37 | 0.68 | 2.03 | 1.69 |
| 1.35 | 1 | 1.23 | 0.84 | 1.55 | 0.81 | 1.01 | 0.99 | 0.75 | 0.76 | 1.35 |
| 1.16 | 0.84 | 0.46 | 0.92 | 2.34 | 0.75 | 0.79 | 1 | 1.14 | 1.08 | 1.16 |

| 218902_at | 213330_s_at | 201131_s_at | 207451_at | 203035_s_at | 209360_s_at | 212748_at | 204020_at | 218902_at | 201029_s_at | 213342_at |
|---|---|---|---|---|---|---|---|---|---|---|
| 1.27 | 1 | 0.94 | 0.56 | 1.14 | 0.89 | 1.17 | 0.89 | 1.27 | 0.99 | 1.02 |
| 1.02 | 1.12 | 0.87 | 0.71 | 0.92 | 0.51 | 1.05 | 1.12 | 1.02 | 1.24 | 2.17 |
| 1.77 | 0.97 | 4.13 | 0.72 | 0.91 | 1.03 | 1.36 | 1.58 | 1.77 | 1.23 | 2.24 |
| 1.36 | 1.06 | 1.06 | 4.05 | 0.97 | 1.19 | 1.15 | 0.8 | 1.36 | 0.95 | 1.37 |
| 1.27 | 0.84 | 0.32 | 0.87 | 0.9 | 1.29 | 0.8 | 0.97 | 1.27 | 0.99 | 1.07 |
| 0.91 | 1.05 | 2.39 | 6.29 | 0.91 | 0.8 | 0.84 | 0.98 | 0.91 | 1.13 | 3.76 |
| 0.34 | 0.88 | 0.25 | 0.7 | 1 | 0.63 | 0.83 | 1.24 | 0.34 | 1.88 | 1.04 |
| 1.05 | 1.21 | 2.16 | 0.56 | 1.05 | 0.87 | 1.04 | 0.99 | 1.05 | 0.78 | 3.5 |
| 0.77 | 1.02 | 0.7 | 0.53 | 0.86 | 0.72 | 1.04 | 1.11 | 0.77 | 0.93 | 1.01 |
| 1.23 | 1.45 | 0.5 | 1.24 | 0.71 | 0.68 | 1.06 | 0.77 | 1.23 | 1.06 | 1.04 |
| 1.3 | 0.86 | 1.85 | 0.81 | 1.06 | 1.09 | 1.11 | 1.05 | 1.3 | 1.03 | 0.63 |
| 0.9 | 1.49 | 1.88 | 3 | 0.98 | 0.82 | 1.08 | 0.88 | 0.9 | 1.06 | 1.22 |
| 0.96 | 1.37 | 1.8 | 0.86 | 0.76 | 0.77 | 1.24 | 1.07 | 0.96 | 1.6 | 0.91 |
| 0.65 | 0.22 | 0.05 | 1.7 | 0.08 | 0.71 | 2.94 | 2.66 | 0.65 | 0.09 | 0.47 |
| 0.72 | 2.07 | 0.82 | 2.82 | 1 | 1.26 | 1.5 | 1.37 | 0.72 | 1.12 | 1.04 |
| 0.87 | 1.11 | 1.46 | 1.09 | 1.33 | 0.8 | 1.44 | 1 | 0.87 | 1.78 | 1.26 |
| 1.15 | 1.28 | 1.69 | 0.75 | 1.24 | 0.88 | 1.04 | 1.09 | 1.15 | 0.91 | 0.94 |
| 1.52 | 1.11 | 0.95 | 4.38 | 1.14 | 1.39 | 1.47 | 1.22 | 1.52 | 1.53 | 1.36 |
| 6.36 | 0.81 | 7 | 2.33 | 1.62 | 1.2 | 0.97 | 1.12 | 6.36 | 1.36 | 2.72 |
| 0.57 | 0.95 | 0.71 | 24.53 | 0.85 | 8.37 | 3.37 | 0.84 | 0.57 | 0.82 | 0.93 |
| 1.06 | 0.92 | 5.79 | 1.17 | 1.41 | 0.8 | 0.9 | 1.49 | 1.06 | 1.39 | 0.88 |
| 1.18 | 0.71 | 1.07 | 2.92 | 2.3 | 1.45 | 2.21 | 0.89 | 1.18 | 2.13 | 0.53 |
| 0.75 | 0.88 | 20.64 | 3.07 | 0.87 | 1.1 | 1.16 | 0.9 | 0.75 | 1.06 | 0.87 |
| 0.28 | 1.07 | 0 | 1.2 | 1.53 | 1.03 | 0.94 | 0.78 | 0.28 | 1.01 | 0.78 |
| 0.52 | 1.14 | 0.06 | 0.6 | 2.37 | 1.89 | 0.68 | 1.04 | 0.52 | 0.67 | 1.35 |
| 1.86 | 1.05 | 1.05 | 1.56 | 0.93 | 0.94 | 0.99 | 0.47 | 1.86 | 0.71 | 1.07 |
| 0.88 | 0.91 | 4.08 | 2.18 | 1.26 | 0.9 | 1.08 | 0.71 | 0.88 | 0.52 | 0.87 |
| 0.59 | 0.93 | 0.38 | 1.98 | 1.54 | 1.03 | 2.15 | 0.74 | 0.59 | 0.77 | 0.67 |
| 1.47 | 2.03 | 63.41 | 0.95 | 1.47 | 0.4 | 1.03 | 1.01 | 1.47 | 1.25 | 0.51 |
| 1.27 | 0.92 | 1.24 | 4.22 | 1.2 | 1.03 | 1.29 | 1.14 | 1.27 | 0.99 | 1.32 |

| | | | | | | | | | | |
|---|---|---|---|---|---|---|---|---|---|---|
| 0.62 | 1.75 | 0.49 | 0.76 | 0.81 | 0.79 | 0.63 | 1 | 0.62 | 0.56 | 5.19 |
| 0.83 | 2.7 | 0.18 | 2.66 | 1.98 | 0.53 | 1.96 | 2.51 | 0.83 | 3.69 | 1.16 |
| 1.12 | 0.99 | 1.04 | 1.62 | 1.01 | 1 | 1.15 | 1.11 | 1.12 | 1.02 | 0.96 |
| 0.4 | 1.2 | 0.26 | 3.17 | 0.93 | 0.31 | 0.41 | 0.56 | 0.4 | 1.03 | 0.78 |
| 0.47 | 0.43 | 4 | 0.9 | 0.38 | 0.71 | 5.07 | 1.88 | 0.47 | 2.13 | 40.4 |
| 5.39 | 0.69 | 0.66 | 3.03 | 0.64 | 2.58 | 1.08 | 0.58 | 5.39 | 0.52 | 1.2 |
| 2.09 | 1.17 | 11.14 | 0.52 | 0.6 | 0.32 | 0.77 | 0.57 | 2.09 | 0.5 | 0.57 |
| 1 | 0.9 | 2.5 | 0.33 | 1.08 | 1.05 | 1.06 | 0.77 | 1 | 0.97 | 0.88 |
| 0.71 | 0.94 | 0.98 | 0.76 | 0.96 | 0.82 | 0.6 | 1.88 | 0.71 | 1.65 | 0.63 |
| 0.4 | 0.89 | 0.12 | 0.42 | 0.84 | 2.14 | 2.32 | 1 | 0.4 | 0.89 | 1.02 |
| 0.99 | 1.25 | 1.83 | 0.81 | 1.23 | 2.8 | 0.78 | 1.03 | 0.99 | 1.01 | 1.08 |
| 1.66 | 0.93 | 0.35 | 3.1 | 1.02 | 0.78 | 0.76 | 0.89 | 1.66 | 1.01 | 0.9 |
| 0.76 | 0.98 | 1.35 | 0.43 | 0.91 | 1.59 | 1.62 | 1.08 | 0.76 | 0.82 | 1.36 |
| 1.64 | 0.96 | 2.6 | 1.89 | 1.21 | 2.86 | 1.06 | 1.39 | 1.64 | 2.25 | 1.62 |
| 0.86 | 1.06 | 1.32 | 0.43 | 0.76 | 1.53 | 1.34 | 0.97 | 0.86 | 0.81 | 1.24 |
| 1.06 | 1.07 | 9.73 | 0.59 | 0.96 | 2.83 | 1.28 | 1.06 | 1.06 | 0.78 | 1.29 |
| 0.86 | 1.01 | 1.06 | 1.55 | 0.99 | 1.08 | 1.04 | 1.09 | 0.86 | 1.22 | 0.84 |
| 0.85 | 0.82 | 0.49 | 0.95 | 0.88 | 1.36 | 1.31 | 0.64 | 0.85 | 0.87 | 1.23 |
| 1.2 | 0.99 | 0.79 | 2.15 | 1.04 | 1.05 | 0.99 | 1 | 1.2 | 0.93 | 0.95 |
| 1.29 | 1.23 | 0.17 | 0.85 | 1.09 | 0.77 | 0.71 | 1.2 | 1.29 | 1.5 | 0.83 |
| 0.69 | 1.08 | 0.87 | 0.94 | 0.9 | 0.74 | 0.95 | 0.97 | 0.69 | 0.97 | 0.81 |
| 0.86 | 0.91 | 0.98 | 2.14 | 0.77 | 1.21 | 1.15 | 1.24 | 0.86 | 0.83 | 0.73 |
| 0.82 | 0.87 | 1.68 | 1.03 | 0.74 | 1.09 | 1.33 | 0.8 | 0.82 | 0.91 | 1.18 |
| 1.02 | 0.88 | 1.08 | 1.76 | 0.93 | 4.23 | 1.32 | 1.25 | 1.02 | 1.84 | 2.26 |
| 1.67 | 1.4 | 4.12 | 1 | 1.02 | 0.58 | 0.73 | 0.79 | 1.67 | 0.83 | 1.23 |
| 1.01 | 1.4 | 0.2 | 0.49 | 0.99 | 0.57 | 0.81 | 0.85 | 1.01 | 1.01 | 1.26 |
| 1.08 | 1.91 | 4.83 | 2.98 | 0.91 | 1.32 | 1.29 | 0.7 | 1.08 | 1.36 | 1.54 |
| 0.88 | 0.67 | 1.43 | 1.72 | 0.99 | 2.78 | 1.41 | 1 | 0.88 | 0.71 | 1.18 |
| 1.52 | 0.76 | 1.01 | 1.26 | 1.35 | 1.1 | 1.07 | 0.91 | 1.52 | 1.23 | 0.95 |
| 0.86 | 1.03 | 1.33 | 0.52 | 0.78 | 0.91 | 1.27 | 0.99 | 0.86 | 0.59 | 1.5 |
| 0.61 | 0.68 | 0.75 | 2.18 | 1.01 | 3.68 | 0.99 | 1.49 | 0.61 | 6.39 | 1.09 |
| 1.39 | 0.61 | 1.09 | 1.04 | 1.05 | 1.32 | 1.47 | 1.32 | 1.39 | 1.18 | 0.98 |
| 1.53 | 0.87 | 3.78 | 1.28 | 1.45 | 2.64 | 1.63 | 0.94 | 1.53 | 1.12 | 1.27 |

| | | | | | | | | | | |
|---|---|---|---|---|---|---|---|---|---|---|
| 0.72 | 1.14 | 0.14 | 0.69 | 1.26 | 1.05 | 1.04 | 1.09 | 0.72 | 1.81 | 0.98 |
| 1.81 | 1.19 | 2.08 | 1.85 | 1.08 | 0.94 | 1.14 | 1.09 | 1.81 | 0.96 | 0.78 |
| 0.74 | 1.11 | 251.75 | 0.73 | 1.09 | 0.89 | 1.11 | 1.21 | 0.74 | 1.18 | 0.98 |
| 1.47 | 0.97 | 2.83 | 1.24 | 1.19 | 2.76 | 1.28 | 1.29 | 1.47 | 1.14 | 1.13 |
| 0.99 | 0.85 | 1.56 | 1.94 | 0.84 | 1.17 | 0.97 | 1.27 | 0.99 | 0.9 | 0.9 |
| 1.69 | 0.92 | 0.82 | 0.61 | 0.94 | 0.9 | 0.53 | 2.5 | 1.69 | 1.36 | 0.74 |
| 1.12 | 1.2 | 0.67 | 4.14 | 0.59 | 0.65 | 1.17 | 1.69 | 1.12 | 0.76 | 1.73 |
| 0.94 | 1.64 | 0.66 | 4.24 | 1.02 | 0.92 | 1.73 | 0.92 | 0.94 | 1.06 | 0.87 |
| 0.75 | 0.88 | 1.63 | 1.48 | 1.31 | 3.41 | 2.83 | 0.79 | 0.75 | 1.49 | 0.88 |
| 1.27 | 0.81 | 1.52 | 1.54 | 1.15 | 0.92 | 1.24 | 1.3 | 1.27 | 1.03 | 1.26 |
| 1.56 | 1.15 | 10.38 | 2.38 | 1.24 | 0.91 | 0.99 | 1.55 | 1.56 | 0.82 | 2.14 |
| 1.36 | 1.58 | 1.06 | 1.37 | 0.87 | 11.73 | 0.93 | 1.01 | 1.36 | 0.84 | 1.26 |
| 1.34 | 0.72 | 1.41 | 5.25 | 0.95 | 1.23 | 1.08 | 0.84 | 1.34 | 1.21 | 1.11 |
| 1.24 | 1.02 | 0.74 | 5.36 | 1.26 | 1.45 | 1.2 | 1.39 | 1.24 | 0.51 | 1.18 |
| 0.91 | 1.75 | 2.67 | 1.33 | 1.09 | 1.03 | 1.37 | 0.83 | 0.91 | 1.16 | 2 |
| 0.3 | 0.49 | 2.37 | 0.22 | 14.79 | 2.12 | 2.87 | 0.03 | 0.3 | 9.65 | 52.59 |
| 1.04 | 0.95 | 0.9 | 1.62 | 1.46 | 1.45 | 2.09 | 1.44 | 1.04 | 0.67 | 1.07 |
| 0.89 | 0.64 | 0.1 | 0.27 | 0.62 | 1.2 | 1.11 | 0.87 | 0.89 | 0.82 | 2.06 |
| 0.79 | 1.11 | 1.63 | 4.4 | 0.95 | 0.78 | 0.78 | 1.17 | 0.79 | 0.87 | 1.52 |
| 2.04 | 1.42 | 1.19 | 2.36 | 1.22 | 0.65 | 2.38 | 0.82 | 2.04 | 0.93 | 5.36 |
| 1.71 | 0.42 | 279 | 0.47 | 1.45 | 8.44 | 2.29 | 0.48 | 1.71 | 2.97 | 0.88 |
| 0.94 | 1.38 | 4.57 | 1.01 | 1.08 | 6.51 | 1.09 | 1.12 | 0.94 | 1.02 | 0.78 |
| 0.39 | 1.37 | 0.53 | 2 | 0.97 | 0.57 | 1.06 | 1.14 | 0.39 | 1.62 | 0.86 |
| 0.73 | 0.43 | 0.85 | 0.52 | 2.16 | 0.46 | 0.89 | 2.14 | 0.73 | 1.04 | 1.26 |
| 1.17 | 0.95 | 1.12 | 1.28 | 0.94 | 1.02 | 0.88 | 1.82 | 1.17 | 1.3 | 1.55 |
| 0.67 | 0.94 | 1.36 | 1.38 | 1 | 1 | 0.76 | 0.94 | 0.67 | 1.08 | 1.15 |
| 1.1 | 1.39 | 1.04 | 1.24 | 0.98 | 0.78 | 1.05 | 0.71 | 1.1 | 1.19 | 0.84 |
| 1.51 | 1.17 | 0.51 | 0.66 | 0.98 | 0.84 | 0.99 | 1.01 | 1.51 | 1.19 | 2.29 |
| 0.98 | 1.32 | 1.49 | 1.58 | 1.13 | 0.93 | 1.08 | 1.12 | 0.98 | 1.19 | 1.2 |
| 1.05 | 0.99 | 0.94 | 0.99 | 0.89 | 1 | 1.23 | 0.94 | 1.05 | 0.8 | 1.02 |
| 1.28 | 1.03 | 0.78 | 2.7 | 0.86 | 1.38 | 1.19 | 0.94 | 1.28 | 1.02 | 0.9 |
| 0.79 | 0.95 | 2.92 | 3.25 | 1.03 | 2.6 | 1.04 | 0.96 | 0.79 | 0.95 | 0.82 |
| 1.37 | 0.87 | 2 | 3 | 1.34 | 0.79 | 1.19 | 1.03 | 1.37 | 1.31 | 1.65 |

| | | | | | | | | | | |
|---|---|---|---|---|---|---|---|---|---|---|
| 1 | 1.65 | 1.34 | 1.96 | 1.3 | 1.03 | 1.07 | 1.16 | 1 | 0.64 | 0.8 |
| 1.17 | 1.07 | 1.67 | 1.39 | 1.04 | 0.74 | 1.15 | 0.96 | 1.17 | 0.82 | 0.63 |
| 1.18 | 1.04 | 0.98 | 0.71 | 1.17 | 1.1 | 0.96 | 1.21 | 1.18 | 0.92 | 0.99 |
| 1.3 | 1.1 | 1.31 | 3.85 | 0.65 | 3.53 | 2.84 | 0.67 | 1.3 | 0.84 | 1.11 |
| 0.86 | 0.86 | 1.47 | 0.63 | 1.02 | 1.28 | 0.85 | 1.49 | 0.86 | 1.18 | 0.73 |
| 0.65 | 2 | 0.85 | 0.95 | 0.85 | 0.34 | 0.98 | 0.68 | 0.65 | 0.55 | 0.6 |
| 0.81 | 0.9 | 1.73 | 3.04 | 0.97 | 0.86 | 1.42 | 0.94 | 0.81 | 0.74 | 1.16 |
| 0.87 | 0.75 | 4.3 | 1.07 | 1.62 | 1.23 | 0.92 | 0.85 | 0.87 | 0.77 | 1.26 |
| 0.86 | 1.08 | 0.73 | 0.95 | 1.01 | 0.79 | 1.26 | 0.64 | 0.86 | 1.16 | 1.23 |
| 0.97 | 1.06 | 0.67 | 2.75 | 1.06 | 1.04 | 1.25 | 1.11 | 0.97 | 0.88 | 1.19 |
| 1.06 | 1.64 | 3.03 | 3.41 | 1.28 | 1.18 | 1.77 | 0.87 | 1.06 | 1.06 | 1.2 |
| 1.31 | 0.82 | 0.98 | 1.35 | 1.26 | 1.25 | 1.15 | 1.1 | 1.31 | 1.09 | 0.91 |
| 1.78 | 1.68 | 1.5 | 1.46 | 0.83 | 0.71 | 1 | 0.93 | 1.78 | 0.78 | 0.91 |
| 0.57 | 1.44 | 0.52 | 1.36 | 1.16 | 0.53 | 0.67 | 0.92 | 0.57 | 0.97 | 0.98 |
| 0.99 | 0.88 | 1.09 | 1.31 | 0.96 | 0.55 | 1.21 | 1.06 | 0.99 | 1 | 1.11 |
| 1.79 | 1.26 | 13.72 | 2.62 | 1.15 | 1.3 | 1.02 | 1.22 | 1.79 | 1.18 | 3.63 |
| 1.24 | 0.85 | 0.7 | 0.73 | 0.81 | 1.1 | 1 | 1.05 | 1.24 | 0.85 | 0.56 |
| 1.76 | 1.12 | 3.63 | 1.41 | 1.12 | 3.21 | 1.22 | 1.11 | 1.76 | 1.32 | 1.29 |
| 1.13 | 0.75 | 1.39 | 2.74 | 1.59 | 0.85 | 1.43 | 0.78 | 1.13 | 1.35 | 1.48 |
| 1.47 | 1.9 | 0.07 | 1.82 | 0.9 | 1.06 | 1.01 | 1.19 | 1.47 | 1.09 | 0.96 |
| 1.03 | 1.36 | 1.2 | 0.64 | 0.84 | 0.84 | 0.79 | 1.66 | 1.03 | 1.41 | 1.34 |
| 1.18 | 1.26 | 0.25 | 1.96 | 0.73 | 1.13 | 1.04 | 1.18 | 1.18 | 1 | 1.03 |
| 1.13 | 0.81 | 1.04 | 1.3 | 1.2 | 1.39 | 1.03 | 0.97 | 1.13 | 1.11 | 0.76 |
| 2.95 | 0.49 | 0.85 | 0.58 | 0.61 | 0.87 | 1.58 | 1.63 | 2.95 | 0.44 | 0.67 |
| 1.7 | 1.18 | 7.96 | 3.3 | 0.6 | 1.04 | 1.17 | 0.95 | 1.7 | 0.81 | 1.2 |
| 0.54 | 0.73 | 0.92 | 1.76 | 1 | 1.49 | 1.05 | 0.47 | 0.54 | 0.93 | 1.39 |
| 1.01 | 1.21 | 0.97 | 0.82 | 0.86 | 1.2 | 0.81 | 0.91 | 1.01 | 0.93 | 0.99 |
| 1.5 | 0.59 | 2.79 | 0.85 | 1.07 | 0.85 | 1 | 0.88 | 1.5 | 1.82 | 0.87 |
| 0.51 | 0.92 | 1.24 | 0.24 | 1.03 | 1.07 | 0.96 | 0.82 | 0.51 | 1.01 | 1.12 |
| 1.12 | 0.96 | 1 | 1.63 | 1.13 | 0.92 | 1.06 | 0.93 | 1.12 | 0.99 | 0.86 |
| 1.12 | 0.96 | 1.61 | 1.46 | 0.98 | 0.95 | 1.23 | 0.92 | 1.12 | 0.66 | 0.2 |
| 1.81 | 1.19 | 2.08 | 1.85 | 1.08 | 0.94 | 1.14 | 1.09 | 1.81 | 0.96 | 0.78 |
| 0.89 | 0.96 | 0.93 | 1.3 | 0.94 | 0.81 | 0.77 | 1.1 | 0.89 | 0.9 | 1.15 |

| | | | | | | | | | | |
|---|---|---|---|---|---|---|---|---|---|---|
| 0.98 | 1.75 | 3.07 | 1.56 | 1.54 | 0.36 | 0.95 | 1.3 | 0.98 | 0.86 | 1.32 |
| 0.95 | 1.1 | 0.96 | 1.3 | 1.12 | 0.84 | 1.34 | 0.81 | 0.95 | 1.21 | 1.01 |
| 1.23 | 0.85 | 0.52 | 1.09 | 1.19 | 0.7 | 0.88 | 1.04 | 1.23 | 1.02 | 1.25 |
| 1.23 | 1.15 | 1.77 | 0.98 | 1.02 | 3.24 | 1.1 | 1 | 1.23 | 0.56 | 8.57 |
| 0.67 | 0.89 | 6.35 | 1.58 | 0.89 | 0.82 | 0.86 | 1.08 | 0.67 | 0.98 | 0.94 |
| 1.04 | 1.17 | 1.31 | 2.4 | 1.15 | 1.01 | 0.97 | 0.98 | 1.04 | 0.96 | 1.05 |
| 1.2 | 1.14 | 0.92 | 1.92 | 0.96 | 0.86 | 1 | 0.92 | 1.2 | 0.96 | 0.94 |
| 1.28 | 0.94 | 3.52 | 1.12 | 1.04 | 0.8 | 1.01 | 1.06 | 1.28 | 0.84 | 1.18 |
| 1.47 | 0.89 | 2.27 | 1.12 | 1.26 | 1.33 | 0.66 | 1.32 | 1.47 | 0.98 | 0.62 |
| 1.38 | 1.25 | 1.5 | 1.29 | 1 | 0.84 | 1.14 | 0.99 | 1.38 | 1.34 | 1.05 |
| 1.05 | 1.2 | 0.51 | 3.33 | 1.02 | 0.47 | 1.05 | 0.88 | 1.05 | 0.94 | 0.59 |
| 0.8 | 1.05 | 1.28 | 1.12 | 1.14 | 0.92 | 0.71 | 1 | 0.8 | 0.9 | 1.09 |
| 1.72 | 1.04 | 2.18 | 1.04 | 1.3 | 2.59 | 1.04 | 1.2 | 1.72 | 1.22 | 1.15 |
| 1.53 | 0.71 | 2.19 | 0.91 | 1.04 | 0.67 | 0.94 | 1.28 | 1.53 | 0.94 | 1.08 |
| 0.45 | 0.27 | 1.87 | 0.79 | 1.14 | 1.03 | 0.57 | 1.46 | 0.45 | 1.79 | 2.65 |
| 1.07 | 1.08 | 0.8 | 1.13 | 1.11 | 1 | 1.03 | 0.98 | 1.07 | 1.34 | 0.28 |
| 0.89 | 0.99 | 1.06 | 1.46 | 0.85 | 1.58 | 0.84 | 1.03 | 0.89 | 1 | 0.84 |
| 1.03 | 1.4 | 7.66 | 3.14 | 1.46 | 0.72 | 0.87 | 1.02 | 1.03 | 0.76 | 2.47 |
| 0.8 | 1.04 | 2.43 | 2.29 | 1.05 | 0.74 | 0.95 | 0.83 | 0.8 | 1.03 | 0.87 |
| 0.85 | 0.89 | 1.15 | 1.27 | 0.84 | 0.97 | 1.2 | 1.19 | 0.85 | 1.16 | 1.02 |
| 1.46 | 1.24 | 0.95 | 0.51 | 1.85 | 1.35 | 1.07 | 1.13 | 1.46 | 0.87 | 1.15 |
| 0.46 | 0.65 | 0.96 | 1.09 | 0.93 | 1.23 | 0.8 | 1.16 | 0.46 | 1.07 | 1.23 |
| 1.02 | 0.98 | 0.86 | 1.76 | 0.92 | 1.16 | 1.13 | 1.03 | 1.02 | 1.14 | 3.56 |
| 1.37 | 0.63 | 0.34 | 1.97 | 0.86 | 0.27 | 1.06 | 1.08 | 1.37 | 0.9 | 1.37 |
| 1.32 | 0.86 | 1.81 | 1.67 | 1.41 | 0.93 | 1.28 | 1.12 | 1.32 | 1.02 | 1.32 |
| 1.06 | 0.91 | 1.1 | 0.89 | 0.88 | 0.78 | 0.87 | 1.18 | 1.06 | 1.25 | 0.82 |
| 0.88 | 0.88 | 1.98 | 3.51 | 0.87 | 0.66 | 1.01 | 0.91 | 0.88 | 1.09 | 0.83 |
| 1.25 | 0.92 | 0.99 | 2.47 | 1.09 | 1.37 | 1.03 | 0.99 | 1.25 | 0.9 | 1.13 |
| 1.05 | 0.63 | 1.67 | 1.01 | 1.08 | 3.08 | 1.23 | 1.08 | 1.05 | 1.04 | 1.2 |
| 0.97 | 0.93 | 0.52 | 1.53 | 0.87 | 1.12 | 0.85 | 1.03 | 0.97 | 0.99 | 0.83 |
| 0.85 | 1.12 | 1.09 | 3.63 | 0.87 | 0.98 | 0.83 | 0.7 | 0.85 | 0.93 | 2.19 |
| 0.8 | 1.03 | 1 | 0.99 | 0.98 | 13.94 | 1.08 | 0.85 | 0.8 | 1.08 | 1.03 |
| 0.8 | 1.07 | 0.84 | 0.79 | 1.06 | 0.98 | 1.09 | 1.02 | 0.8 | 1.02 | 3.17 |

| | | | | | | | | | | |
|---|---|---|---|---|---|---|---|---|---|---|
| 0.69 | 0.85 | 1.65 | 0.69 | 0.93 | 3.9 | 2.45 | 0.66 | 0.69 | 1.4 | 1.31 |
| 0.85 | 1.15 | 1.73 | 0.83 | 1.03 | 1.05 | 1.13 | 1.01 | 0.85 | 0.99 | 1.12 |
| 0.93 | 1.13 | 0.84 | 1.36 | 0.98 | 0.97 | 1.02 | 1.03 | 0.93 | 0.85 | 1.04 |
| 1.34 | 0.43 | 0.55 | 0.53 | 0.67 | 1 | 0.92 | 1.18 | 1.34 | 0.64 | 0.5 |
| 0.68 | 1.31 | 1.04 | 6 | 1.44 | 0.78 | 1.26 | 0.61 | 0.68 | 0.44 | 0.75 |
| 1.2 | 0.89 | 1.46 | 2.12 | 1.02 | 1.01 | 1.03 | 0.88 | 1.2 | 1.06 | 0.95 |
| 0.66 | 1.64 | 0.57 | 0.84 | 0.86 | 0.78 | 0.65 | 1.02 | 0.66 | 0.55 | 4.07 |
| 1.4 | 1.09 | 2.47 | 0.72 | 0.81 | 1.22 | 0.78 | 1.01 | 1.4 | 0.94 | 0.77 |
| 1.18 | 0.92 | 0.53 | 0.66 | 1.29 | 0.66 | 1.22 | 1.03 | 1.18 | 1.29 | 1.62 |
| 0.6 | 1.62 | 2.12 | 0.74 | 0.77 | 1.02 | 0.61 | 1.02 | 0.6 | 0.57 | 2.85 |
| 1.18 | 1.94 | 0.81 | 1.23 | 1.62 | 1.33 | 0.81 | 1 | 1.18 | 0.88 | 0.93 |
| 0.52 | 0.89 | 2.12 | 3.4 | 0.94 | 0.85 | 1.17 | 0.93 | 0.52 | 1.63 | 1.27 |
| 0.84 | 0.88 | 1.02 | 0.82 | 0.96 | 1 | 0.88 | 0.81 | 0.84 | 0.69 | 0.85 |
| 0.93 | 0.93 | 5.69 | 1.35 | 1.54 | 0.82 | 0.98 | 0.97 | 0.93 | 0.95 | 4.53 |
| 1.12 | 1.2 | 2.01 | 2.7 | 0.9 | 2.1 | 1.18 | 1.06 | 1.12 | 0.66 | 3.14 |
| 0.71 | 1.14 | 0.93 | 0.75 | 0.86 | 0.5 | 0.98 | 1.05 | 0.71 | 1.12 | 1.18 |
| 1.14 | 1.23 | 1.75 | 2.06 | 1.29 | 1.19 | 1.09 | 1.03 | 1.14 | 0.89 | 5 |
| 1.17 | 0.93 | 1.13 | 1.59 | 0.72 | 0.91 | 0.8 | 0.93 | 1.17 | 0.89 | 1.01 |
| 0.93 | 1.13 | 0.92 | 1.22 | 0.63 | 2.32 | 0.71 | 1.3 | 0.93 | 1.4 | 1.38 |
| 0.8 | 0.73 | 1.08 | 0.3 | 1.29 | 0.89 | 0.94 | 1.31 | 0.8 | 1.08 | 1.48 |
| 2.1 | 1.33 | 0.9 | 3 | 1.01 | 0.73 | 2.46 | 0.78 | 2.1 | 1.12 | 0.9 |
| 1.09 | 7.28 | 709.31 | 0.52 | 0.79 | 0.16 | 1.05 | 1.99 | 1.09 | 2.5 | 0.94 |
| 0.89 | 1.13 | 6.58 | 0.84 | 1.43 | 0.75 | 1.24 | 0.99 | 0.89 | 0.98 | 1.3 |
| 0.98 | 1.14 | 1.43 | 1.88 | 0.8 | 4.07 | 1.73 | 1.18 | 0.98 | 1.19 | 1.58 |
| 0.87 | 0.8 | 2.31 | 0.79 | 1.03 | 1.35 | 0.84 | 0.91 | 0.87 | 0.94 | 1.04 |
| 1.75 | 0.67 | 0.22 | 3.04 | 0.74 | 8.09 | 1.01 | 1.61 | 1.75 | 1.44 | 0.84 |
| 1 | 0.91 | 1.04 | 1.18 | 1.04 | 1.46 | 1.13 | 1.19 | 1 | 1.08 | 1.39 |
| 1.14 | 1.02 | 0.68 | 1.37 | 1.02 | 1.02 | 1.04 | 0.84 | 1.14 | 1.1 | 2.72 |
| 0.97 | 1.06 | 0.83 | 2.85 | 1.03 | 1.03 | 0.99 | 1.01 | 0.97 | 0.95 | 3.84 |
| 1.02 | 0.91 | 1.17 | 0.22 | 0.98 | 1.1 | 1.07 | 1.1 | 1.02 | 1 | 0.97 |
| 0.89 | 1.55 | 1.16 | 1.68 | 1.08 | 1.12 | 0.71 | 1.03 | 0.89 | 1.11 | 1.14 |
| 0.88 | 1.05 | 1.08 | 0.8 | 0.94 | 0.88 | 0.9 | 0.95 | 0.88 | 0.96 | 0.9 |
| 1.23 | 0.88 | 5.57 | 5.97 | 0.67 | 1.03 | 0.88 | 0.85 | 1.23 | 1.14 | 0.66 |

| | | | | | | | | | | |
|---|---|---|---|---|---|---|---|---|---|---|
| 1.13 | 1.06 | 0.11 | 4.22 | 1.17 | 1.09 | 1.84 | 0.86 | 1.13 | 1.26 | 0.95 |
| 1.4 | 1.09 | 1.4 | 0.14 | 0.76 | 1.5 | 0.87 | 0.79 | 1.4 | 1.3 | 1.44 |
| 0.85 | 0.64 | 1.05 | 0.84 | 0.89 | 1.08 | 0.53 | 1.08 | 0.85 | 1.03 | 0.79 |
| 0.63 | 1.47 | 1.33 | 0.92 | 0.86 | 0.73 | 0.8 | 1.73 | 0.63 | 0.85 | 0.74 |
| 1.04 | 1.44 | 1.83 | 2.16 | 1.22 | 0.85 | 1.01 | 1.13 | 1.04 | 0.79 | 2.21 |
| 1.04 | 1.21 | 2.28 | 1.99 | 0.82 | 1.98 | 0.82 | 1.08 | 1.04 | 0.68 | 1.08 |
| 0.56 | 0.74 | 1.1 | 1.51 | 1.04 | 0.97 | 0.7 | 0.66 | 0.56 | 1.15 | 0.78 |
| 0.63 | 1.33 | 0.49 | 2.31 | 0.69 | 0.72 | 1.05 | 1.25 | 0.63 | 0.83 | 0.29 |
| 0.82 | 1.3 | 1.64 | 1.41 | 0.89 | 3.12 | 1.64 | 0.71 | 0.82 | 1.15 | 0.79 |
| 1.16 | 0.89 | 0.56 | 0.86 | 0.91 | 0.85 | 1.39 | 1.1 | 1.16 | 0.45 | 1.4 |
| 0.32 | 0.96 | 1.13 | 1.04 | 0.98 | 0.74 | 0.78 | 0.87 | 0.32 | 1.09 | 1.33 |
| 0.91 | 0.89 | 0.39 | 1.01 | 0.94 | 0.62 | 1.08 | 1 | 0.91 | 1.11 | 0.62 |
| 0.83 | 0.81 | 1.07 | 0.94 | 0.59 | 0.93 | 1.25 | 0.91 | 0.83 | 1.31 | 0.8 |
| 1.11 | 0.93 | 5.92 | 1.33 | 0.98 | 0.86 | 1.4 | 0.93 | 1.11 | 0.65 | 0.17 |
| 1 | 0.97 | 1.22 | 2.39 | 0.93 | 0.97 | 1.13 | 1.25 | 1 | 1.04 | 0.92 |
| 0.9 | 1 | 1.69 | 1.09 | 1.05 | 1.2 | 1.17 | 1.18 | 0.9 | 0.7 | 1.01 |
| 1.47 | 0.86 | 0.3 | 1.07 | 0.84 | 0.74 | 1.16 | 0.92 | 1.47 | 0.97 | 0.82 |
| 0.7 | 1.04 | 0.55 | 1.12 | 1.15 | 1.01 | 0.92 | 0.99 | 0.7 | 0.98 | 9.36 |
| 1.06 | 0.92 | 0.3 | 2.59 | 0.77 | 2.72 | 1.8 | 0.73 | 1.06 | 2.03 | 1.3 |
| 0.4 | 0.55 | 0.03 | 1.58 | 0.41 | 0.9 | 1.1 | 1.35 | 0.4 | 1.47 | 0.5 |
| 1.07 | 0.71 | 1.16 | 0.65 | 0.93 | 1.78 | 1.34 | 0.91 | 1.07 | 1.05 | 0.9 |
| 0.6 | 1.74 | 1.27 | 0.7 | 0.76 | 0.91 | 0.61 | 1 | 0.6 | 0.57 | 4.58 |
| 0.56 | 2.95 | 0.81 | 0.68 | 0.89 | 1.05 | 0.8 | 1.07 | 0.56 | 0.85 | 0.99 |
| 0.53 | 1.15 | 0 | 1.37 | 0.99 | 1.42 | 0.9 | 1.19 | 0.53 | 1.05 | 1.11 |
| 1.1 | 0.81 | 1.04 | 0.54 | 0.79 | 1 | 1.01 | 0.8 | 1.1 | 1.13 | 0.99 |
| 1.04 | 0.92 | 0.99 | 1.29 | 1.49 | 0.83 | 0.93 | 0.83 | 1.04 | 1.87 | 1.39 |
| 1.18 | 1.1 | 0.86 | 0.73 | 0.74 | 0.59 | 0.72 | 0.9 | 1.18 | 1.31 | 0.63 |
| 0.49 | 1.78 | 0.2 | 0.92 | 1.62 | 0.94 | 0.78 | 0.88 | 0.49 | 1.41 | 1.14 |
| 0.7 | 1.16 | 0.85 | 1.19 | 1.8 | 0.79 | 1.18 | 0.72 | 0.7 | 1.1 | 1.73 |
| 3 | 0.23 | 1.13 | 0.22 | 0.74 | 1.66 | 0.91 | 1.02 | 3 | 0.59 | 3.36 |
| 0.71 | 0.96 | 2 | 5 | 1.89 | 1.83 | 1.51 | 0.98 | 0.71 | 0.75 | 2.14 |
| 1.1 | 0.69 | 0.93 | 0.83 | 1.97 | 0.93 | 1.4 | 1.64 | 1.1 | 2.03 | 2.41 |
| 1.81 | 0.68 | 2.11 | 0.25 | 1.66 | 1.13 | 1.74 | 0.81 | 1.81 | 2.51 | 1.58 |

| | | | | | | | | | | |
|---|---|---|---|---|---|---|---|---|---|---|
| 1 | 1.34 | 4.12 | 4.5 | 1.01 | 1.34 | 0.96 | 1.04 | 1 | 1.13 | 0.88 |
| 0.79 | 0.91 | 0.75 | 3.93 | 1.03 | 0.97 | 1.02 | 1.23 | 0.79 | 0.92 | 0.55 |
| 0.96 | 6.75 | 2.87 | 15.4 | 0.51 | 0.94 | 3.28 | 1.72 | 0.96 | 1.28 | 8.79 |
| 0.96 | 0.85 | 0.23 | 1.16 | 0.8 | 1.05 | 1.66 | 0.61 | 0.96 | 0.73 | 0.7 |
| 0.97 | 0.9 | 56.41 | 0.95 | 0.94 | 0.92 | 1.12 | 0.77 | 0.97 | 1.01 | 1.01 |
| 1.07 | 1.09 | 2.31 | 0.95 | 1.16 | 1.1 | 0.88 | 1.13 | 1.07 | 1.08 | 0.73 |
| 1.11 | 1.23 | 0.89 | 1.16 | 0.99 | 1.1 | 1.06 | 1.19 | 1.11 | 0.98 | 0.91 |
| 1.32 | 1.08 | 2.26 | 1.84 | 0.91 | 0.95 | 0.59 | 1 | 1.32 | 0.77 | 1.04 |
| 1.38 | 0.92 | 1.11 | 4.62 | 0.72 | 1.03 | 1.17 | 0.99 | 1.38 | 1.64 | 1.06 |
| 0.9 | 1.01 | 1.06 | 1.51 | 1.62 | 0.66 | 0.97 | 0.86 | 0.9 | 0.93 | 1.45 |
| 1.05 | 0.97 | 2.73 | 0.97 | 1.04 | 1.52 | 1.31 | 1.14 | 1.05 | 1.11 | 0.98 |
| 3.33 | 1.14 | 0.29 | 0.2 | 0.09 | 0.51 | 0.63 | 23.3 | 3.33 | 0.62 | 0.28 |
| 0.87 | 0.93 | 0.87 | 1.35 | 1.15 | 0.97 | 1.61 | 1.16 | 0.87 | 0.54 | 1.07 |
| 0.76 | 0.82 | 26.12 | 1.35 | 1.07 | 0.69 | 1.09 | 0.95 | 0.76 | 0.78 | 1.02 |

| 201746_at | 201783_s_at | 203195_s_at | 208370_s_at | 201746_at | 207760_s_at | 207168_s_at | 201131_s_at | 201695_s_at | 202417_at | 209360_s_at |
|---|---|---|---|---|---|---|---|---|---|---|
| 0.92 | 1.07 | 1.04 | 1.8 | 0.92 | 1.08 | 0.97 | 0.94 | 1.03 | 1.07 | 0.89 |
| 0.88 | 0.94 | 1.14 | 1.06 | 0.88 | 0.73 | 0.92 | 0.87 | 0.69 | 1.29 | 0.51 |
| 0.94 | 1.28 | 0.97 | 1.49 | 0.94 | 0.99 | 0.97 | 4.13 | 1 | 1 | 1.03 |
| 1.02 | 0.95 | 1.45 | 1.16 | 1.02 | 1.06 | 0.93 | 1.06 | 1.07 | 0.89 | 1.19 |
| 0.89 | 0.99 | 1.17 | 0.79 | 0.89 | 0.81 | 1.02 | 0.32 | 1.34 | 0.8 | 1.29 |
| 0.95 | 1.1 | 0.94 | 1.55 | 0.95 | 0.95 | 0.97 | 2.39 | 1.18 | 0.97 | 0.8 |
| 0.14 | 0.64 | 1.12 | 0.47 | 0.14 | 1.02 | 0.96 | 0.25 | 1.49 | 0.71 | 0.63 |
| 1.36 | 1.46 | 1.29 | 1.14 | 1.36 | 1.02 | 0.94 | 2.16 | 1.14 | 1.26 | 0.87 |
| 0.82 | 0.98 | 1.11 | 0.89 | 0.82 | 0.71 | 1.04 | 0.7 | 1.25 | 0.88 | 0.72 |
| 1.42 | 1.53 | 1.06 | 2.52 | 1.42 | 0.76 | 0.85 | 0.5 | 2.37 | 0.77 | 0.68 |
| 0.68 | 1.05 | 0.93 | 0.78 | 0.68 | 1.02 | 1.02 | 1.85 | 1.08 | 1.02 | 1.09 |
| 0.93 | 0.89 | 1.02 | 1.04 | 0.93 | 0.91 | 1.31 | 1.88 | 1.45 | 1.05 | 0.82 |
| 1.26 | 1.13 | 1.17 | 1.31 | 1.26 | 0.85 | 1.13 | 1.8 | 0.82 | 1.26 | 0.77 |
| 0.19 | 0.45 | 0.47 | 0.07 | 0.19 | 0.35 | 0.15 | 0.05 | 0.26 | 0.37 | 0.71 |
| 1.11 | 0.99 | 1.17 | 1.88 | 1.11 | 1.02 | 1.1 | 0.82 | 0.96 | 1.75 | 1.26 |
| 1.33 | 0.94 | 0.77 | 1.25 | 1.33 | 0.99 | 1.01 | 1.46 | 0.87 | 0.93 | 0.8 |
| 0.99 | 1.12 | 1.11 | 0.63 | 0.99 | 1.35 | 1.03 | 1.69 | 1.43 | 1.07 | 0.88 |
| 1.13 | 1.24 | 1.24 | 0.79 | 1.13 | 1.06 | 0.98 | 0.95 | 0.66 | 1.04 | 1.39 |
| 0.1 | 0.61 | 0.75 | 0.86 | 0.1 | 1.53 | 1.31 | 7 | 0.43 | 1.17 | 1.2 |
| 0.96 | 1.2 | 0.55 | 1.29 | 0.96 | 1.13 | 0.62 | 0.71 | 1.19 | 1.07 | 8.37 |
| 1.87 | 1.03 | 1.17 | 0.51 | 1.87 | 1.36 | 0.74 | 5.79 | 1.02 | 1.1 | 0.8 |
| 3.15 | 2.02 | 1.55 | 0.64 | 3.15 | 0.77 | 0.86 | 1.07 | 1.38 | 0.98 | 1.45 |
| 1.32 | 0.94 | 0.71 | 0.99 | 1.32 | 1.04 | 0.95 | 20.64 | 0.9 | 0.9 | 1.1 |
| 0.95 | 0.95 | 1.05 | 0.93 | 0.95 | 1.28 | 0.71 | 0 | 1.27 | 1.72 | 1.03 |
| 0.63 | 1.15 | 1.22 | 1.03 | 0.63 | 2.72 | 0.79 | 0.06 | 0.98 | 0.55 | 1.89 |
| 1.11 | 0.9 | 0.89 | 0.9 | 1.11 | 0.96 | 1.08 | 1.05 | 1.07 | 1.22 | 0.94 |
| 1.93 | 0.7 | 1.12 | 1.31 | 1.93 | 0.78 | 1.05 | 4.08 | 0.56 | 1.2 | 0.9 |
| 6.62 | 2.18 | 1.67 | 2.1 | 6.62 | 0.72 | 0.62 | 0.38 | 2.55 | 0.88 | 1.03 |
| 0.15 | 0.7 | 1.78 | 1.5 | 0.15 | 2.16 | 0.71 | 63.41 | 1.02 | 1.16 | 0.4 |
| 0.73 | 1.31 | 1.1 | 0.82 | 0.73 | 0.88 | 0.82 | 1.24 | 2.2 | 1.27 | 1.03 |

| | | | | | | | | | | |
|---|---|---|---|---|---|---|---|---|---|---|
| 0.56 | 0.99 | 0.94 | 0.94 | 0.56 | 0.89 | 0.78 | 0.49 | 1.68 | 1.16 | 0.79 |
| 1.38 | 1.54 | 1.12 | 3.63 | 1.38 | 3.92 | 3.49 | 0.18 | 3.13 | 2.89 | 0.53 |
| 1.07 | 0.89 | 1.33 | 1.08 | 1.07 | 0.96 | 1.08 | 1.04 | 1.07 | 1.06 | 1 |
| 1.27 | 0.98 | 0.89 | 1.05 | 1.27 | 0.55 | 1.52 | 0.26 | 2.48 | 0.83 | 0.31 |
| 16 | 2.04 | 0.85 | 0.82 | 16 | 1.32 | 1.38 | 4 | 21 | 0.78 | 0.71 |
| 1.65 | 1.08 | 0.54 | 1.48 | 1.65 | 0.39 | 0.87 | 0.66 | 1.44 | 0.83 | 2.58 |
| 0.57 | 0.69 | 0.66 | 0.96 | 0.57 | 1.3 | 0.89 | 11.14 | 1.83 | 1.01 | 0.32 |
| 0.88 | 0.84 | 0.84 | 0.81 | 0.88 | 0.94 | 1.18 | 2.5 | 0.91 | 1.08 | 1.05 |
| 1.24 | 0.78 | 1.14 | 1.07 | 1.24 | 1.26 | 1.06 | 0.98 | 0.86 | 0.73 | 0.82 |
| 1.01 | 1.55 | 0.79 | 1.71 | 1.01 | 0.99 | 0.65 | 0.12 | 0.35 | 0.8 | 2.14 |
| 2.01 | 1.28 | 1.09 | 1.04 | 2.01 | 1.19 | 0.92 | 1.83 | 0.92 | 0.76 | 2.8 |
| 0.95 | 1.15 | 1.29 | 0.85 | 0.95 | 1.08 | 1.15 | 0.35 | 1.1 | 1.24 | 0.78 |
| 0.26 | 1.62 | 1.06 | 1.5 | 0.26 | 1.21 | 0.99 | 1.35 | 1.49 | 1.01 | 1.59 |
| 2.55 | 1.09 | 0.87 | 1.86 | 2.55 | 1.83 | 0.88 | 2.6 | 0.56 | 0.76 | 2.86 |
| 0.41 | 1.25 | 0.95 | 1.49 | 0.41 | 1 | 1.02 | 1.32 | 1.35 | 1.1 | 1.53 |
| 1.09 | 1.2 | 1.09 | 1.11 | 1.09 | 1.16 | 0.91 | 9.73 | 0.95 | 0.93 | 2.83 |
| 0.97 | 1.01 | 1.02 | 1.05 | 0.97 | 1.14 | 1.06 | 1.06 | 0.96 | 1.02 | 1.08 |
| 0.81 | 1 | 1.13 | 0.78 | 0.81 | 1.41 | 1.05 | 0.49 | 0.8 | 1.07 | 1.36 |
| 0.86 | 1 | 1.04 | 0.98 | 0.86 | 1.06 | 1.05 | 0.79 | 1.02 | 0.93 | 1.05 |
| 0.69 | 1.14 | 0.98 | 1.36 | 0.69 | 0.88 | 0.77 | 0.17 | 1.63 | 0.88 | 0.77 |
| 0.96 | 0.78 | 0.82 | 0.87 | 0.96 | 0.99 | 1.11 | 0.87 | 0.76 | 0.97 | 0.74 |
| 1.05 | 0.88 | 1.22 | 0.53 | 1.05 | 0.91 | 1.19 | 0.98 | 1.69 | 0.94 | 1.21 |
| 1.09 | 0.88 | 1.05 | 1.42 | 1.09 | 1.12 | 0.83 | 1.68 | 1.12 | 1.08 | 1.09 |
| 0.81 | 1.37 | 1.12 | 1.64 | 0.81 | 1.33 | 1.76 | 1.08 | 0.96 | 0.84 | 4.23 |
| 0.35 | 0.81 | 1.08 | 0.64 | 0.35 | 1.53 | 1.1 | 4.12 | 8.76 | 1.24 | 0.58 |
| 1.48 | 1.27 | 1.42 | 0.91 | 1.48 | 0.97 | 1.28 | 0.2 | 12.57 | 1.14 | 0.57 |
| 0.93 | 1.93 | 0.96 | 1.06 | 0.93 | 1.59 | 1.38 | 4.83 | 5.55 | 1.52 | 1.32 |
| 1.99 | 1.04 | 0.83 | 1.35 | 1.99 | 1.38 | 1.19 | 1.43 | 0.8 | 1.34 | 2.78 |
| 0.25 | 1.06 | 1.16 | 0.72 | 0.25 | 1.07 | 0.95 | 1.01 | 0.95 | 0.9 | 1.1 |
| 0.41 | 1.31 | 1.13 | 2.15 | 0.41 | 1.01 | 0.97 | 1.33 | 1.39 | 1.15 | 0.91 |
| 0.73 | 1.04 | 0.84 | 1.37 | 0.73 | 0.73 | 2.74 | 0.75 | 0.62 | 0.69 | 3.68 |
| 0.79 | 0.85 | 1.17 | 1.39 | 0.79 | 0.69 | 0.94 | 1.09 | 0.98 | 0.98 | 1.32 |
| 1.14 | 0.97 | 1.03 | 0.99 | 1.14 | 0.87 | 0.91 | 3.78 | 0.93 | 1 | 2.64 |

| | | | | | | | | | | |
|---|---|---|---|---|---|---|---|---|---|---|
| 0.91 | 1.12 | 0.97 | 1.67 | 0.91 | 1 | 1.11 | 0.14 | 0.75 | 1 | 1.05 |
| 1.49 | 1.37 | 1.19 | 1.41 | 1.49 | 1.18 | 0.86 | 2.08 | 2.38 | 1 | 0.94 |
| 0.89 | 1.08 | 0.93 | 1.25 | 0.89 | 1.07 | 0.98 | 251.75 | 1.08 | 0.94 | 0.89 |
| 0.87 | 1.11 | 0.95 | 0.95 | 0.87 | 1.19 | 1 | 2.83 | 1.24 | 1.57 | 2.76 |
| 0.85 | 1 | 0.94 | 1.27 | 0.85 | 1.04 | 0.96 | 1.56 | 1.09 | 0.86 | 1.17 |
| 1.35 | 0.71 | 1.37 | 1.46 | 1.35 | 0.97 | 1.28 | 0.82 | 0.83 | 1.22 | 0.9 |
| 1.65 | 1.16 | 1.27 | 2.9 | 1.65 | 1.55 | 0.85 | 0.67 | 1.22 | 1.35 | 0.65 |
| 0.98 | 1.38 | 1.66 | 0.95 | 0.98 | 1 | 1.05 | 0.66 | 1.43 | 1.35 | 0.92 |
| 0.92 | 1.06 | 1.91 | 2.06 | 0.92 | 1.5 | 0.88 | 1.63 | 2.51 | 1.06 | 3.41 |
| 1.12 | 1 | 1.27 | 1.23 | 1.12 | 0.64 | 0.81 | 1.52 | 1.15 | 0.9 | 0.92 |
| 1.34 | 0.85 | 1.01 | 0.84 | 1.34 | 0.95 | 1.04 | 10.38 | 1.48 | 0.93 | 0.91 |
| 0.9 | 1.11 | 1.1 | 1.07 | 0.9 | 1.07 | 1.08 | 1.06 | 0.93 | 1.06 | 11.73 |
| 1.19 | 1.3 | 1.19 | 1.28 | 1.19 | 1.03 | 0.83 | 1.41 | 1.33 | 0.8 | 1.23 |
| 1.01 | 0.9 | 1.49 | 0.69 | 1.01 | 1.38 | 0.81 | 0.74 | 1.61 | 0.99 | 1.45 |
| 0.42 | 1.02 | 1.03 | 1.1 | 0.42 | 1.04 | 1.31 | 2.67 | 1.34 | 1.38 | 1.03 |
| 4.36 | 1.04 | 2.34 | 8 | 4.36 | 9.01 | 12.5 | 2.37 | 22.14 | 0.05 | 2.12 |
| 1.36 | 1.3 | 1.05 | 9.07 | 1.36 | 1.2 | 1.07 | 0.9 | 0.64 | 1.06 | 1.45 |
| 0.32 | 0.87 | 0.73 | 0.18 | 0.32 | 0.87 | 1.95 | 0.1 | 0.89 | 0.78 | 1.2 |
| 0.9 | 1.04 | 0.95 | 1 | 0.9 | 0.7 | 0.93 | 1.63 | 1.01 | 0.88 | 0.78 |
| 0.9 | 0.88 | 0.99 | 1.43 | 0.9 | 1.22 | 0.99 | 1.19 | 0.98 | 0.81 | 0.65 |
| 0.72 | 2.58 | 0.75 | 2.05 | 0.72 | 3.02 | 0.95 | 279 | 1 | 1.3 | 8.44 |
| 0.91 | 1.1 | 1.26 | 1.22 | 0.91 | 1.32 | 1.11 | 4.57 | 0.95 | 1.09 | 6.51 |
| 0.11 | 0.82 | 1.05 | 0.44 | 0.11 | 0.93 | 1.01 | 0.53 | 1.47 | 0.67 | 0.57 |
| 0.97 | 0.88 | 1.28 | 1.22 | 0.97 | 0.98 | 0.92 | 0.85 | 1.17 | 1.47 | 0.46 |
| 1.37 | 1.19 | 0.87 | 1 | 1.37 | 0.93 | 1.02 | 1.12 | 0.86 | 1.08 | 1.02 |
| 0.91 | 0.97 | 0.85 | 1.28 | 0.91 | 0.74 | 0.97 | 1.36 | 1.07 | 1 | 1 |
| 1.03 | 1.13 | 1.12 | 0.93 | 1.03 | 0.99 | 1.02 | 1.04 | 1.29 | 1.17 | 0.78 |
| 1.09 | 1.31 | 1.13 | 1.37 | 1.09 | 1.38 | 0.99 | 0.51 | 0.96 | 1.22 | 0.84 |
| 1.05 | 1.11 | 0.95 | 0.9 | 1.05 | 1.06 | 0.95 | 1.49 | 1.33 | 0.82 | 0.93 |
| 0.94 | 0.95 | 1 | 0.99 | 0.94 | 1.01 | 1.02 | 0.94 | 0.95 | 1.03 | 1 |
| 1.22 | 1.29 | 1.19 | 0.95 | 1.22 | 1.05 | 1 | 0.78 | 1.05 | 1.21 | 1.38 |
| 0.56 | 1.05 | 0.74 | 3.26 | 0.56 | 0.86 | 0.77 | 2.92 | 0.5 | 0.84 | 2.6 |
| 1.08 | 1.01 | 1.18 | 1.29 | 1.08 | 1.08 | 0.81 | 2 | 0.95 | 0.94 | 0.79 |

| | | | | | | | | | | |
|---|---|---|---|---|---|---|---|---|---|---|
| 0.94 | 1.17 | 1.01 | 0.82 | 0.94 | 0.61 | 0.99 | 1.34 | 0.89 | 1.05 | 1.03 |
| 0.86 | 0.86 | 1.33 | 1.1 | 0.86 | 1.01 | 1.07 | 1.67 | 1.14 | 1.08 | 0.74 |
| 1.42 | 0.98 | 1.22 | 1.27 | 1.42 | 0.92 | 0.89 | 0.98 | 1.09 | 1.23 | 1.1 |
| 0.96 | 0.96 | 1.48 | 2.33 | 0.96 | 1.12 | 0.75 | 1.31 | 2.44 | 1.07 | 3.53 |
| 1 | 0.84 | 1.27 | 1.38 | 1 | 0.62 | 1.55 | 1.47 | 0.83 | 1.38 | 1.28 |
| 0.73 | 0.84 | 0.78 | 0.32 | 0.73 | 1.2 | 0.58 | 0.85 | 2.11 | 1.7 | 0.34 |
| 1.13 | 1.46 | 1.13 | 0.99 | 1.13 | 0.87 | 0.83 | 1.73 | 1.12 | 1.03 | 0.86 |
| 1.12 | 1.03 | 0.75 | 1.63 | 1.12 | 1.08 | 1.13 | 4.3 | 0.61 | 0.97 | 1.23 |
| 0.75 | 1 | 0.98 | 1.15 | 0.75 | 0.8 | 0.68 | 0.73 | 0.84 | 0.78 | 0.79 |
| 1.33 | 1.19 | 1 | 2.67 | 1.33 | 1.48 | 1.08 | 0.67 | 0.91 | 0.74 | 1.04 |
| 1.43 | 0.94 | 1.12 | 0.97 | 1.43 | 0.98 | 1.43 | 3.03 | 1.03 | 1.01 | 1.18 |
| 1.05 | 0.87 | 0.87 | 0.75 | 1.05 | 1.17 | 1.02 | 0.98 | 1.08 | 0.92 | 1.25 |
| 0.79 | 1.05 | 1.27 | 0.89 | 0.79 | 1.21 | 1.08 | 1.5 | 2.18 | 0.71 | 0.71 |
| 1.16 | 1.04 | 1.41 | 1.18 | 1.16 | 0.64 | 1.15 | 0.52 | 2.67 | 1.1 | 0.53 |
| 1.08 | 1.03 | 1.03 | 0.96 | 1.08 | 1.09 | 1 | 1.09 | 0.84 | 0.93 | 0.55 |
| 1.14 | 1.08 | 1.19 | 0.8 | 1.14 | 1.08 | 1.06 | 13.72 | 0.8 | 1.27 | 1.3 |
| 1.26 | 0.89 | 1.03 | 0.93 | 1.26 | 0.93 | 0.94 | 0.7 | 1.27 | 0.89 | 1.1 |
| 0.91 | 1.18 | 1.02 | 0.95 | 0.91 | 1.4 | 1.02 | 3.63 | 1.53 | 2.27 | 3.21 |
| 1.14 | 1.01 | 1.05 | 1 | 1.14 | 1.1 | 0.9 | 1.39 | 0.93 | 1.07 | 0.85 |
| 0.7 | 0.99 | 1.89 | 1.26 | 0.7 | 0.92 | 1.23 | 0.07 | 0.94 | 1.51 | 1.06 |
| 0.7 | 0.95 | 0.92 | 1.11 | 0.7 | 0.86 | 0.99 | 1.2 | 1.28 | 1.16 | 0.84 |
| 1.09 | 0.87 | 0.85 | 0.88 | 1.09 | 0.62 | 0.91 | 0.25 | 1.08 | 0.99 | 1.13 |
| 1.03 | 0.89 | 0.96 | 1.15 | 1.03 | 1.13 | 0.93 | 1.04 | 0.97 | 1.08 | 1.39 |
| 1.63 | 1.14 | 1.42 | 1.24 | 1.63 | 0.81 | 0.95 | 0.85 | 0.4 | 1.35 | 0.87 |
| 1.31 | 0.82 | 1.06 | 1.05 | 1.31 | 0.84 | 1.03 | 7.96 | 1.14 | 1.27 | 1.04 |
| 1.21 | 0.96 | 0.91 | 1.26 | 1.21 | 0.98 | 0.84 | 0.92 | 1.37 | 0.94 | 1.49 |
| 1.03 | 1.13 | 0.69 | 0.98 | 1.03 | 1.07 | 1.12 | 0.97 | 0.91 | 1.12 | 1.2 |
| 1.32 | 0.73 | 1.09 | 1 | 1.32 | 1.46 | 0.87 | 2.79 | 0.8 | 0.68 | 0.85 |
| 0.52 | 0.97 | 1.07 | 0.69 | 0.52 | 1.29 | 1.18 | 1.24 | 1.76 | 1.03 | 1.07 |
| 0.94 | 1.02 | 1 | 1.21 | 0.94 | 1.71 | 0.96 | 1 | 0.93 | 0.89 | 0.92 |
| 5.78 | 1.19 | 0.98 | 0.45 | 5.78 | 0.87 | 1.02 | 1.61 | 1.08 | 0.95 | 0.95 |
| 1.49 | 1.37 | 1.19 | 1.41 | 1.49 | 1.18 | 0.86 | 2.08 | 2.38 | 1 | 0.94 |
| 0.97 | 1.24 | 0.99 | 1.59 | 0.97 | 0.85 | 1 | 0.93 | 1.35 | 1.11 | 0.81 |

| | | | | | | | | | | |
|---|---|---|---|---|---|---|---|---|---|---|
| 1.58 | 0.96 | 0.98 | 1.09 | 1.58 | 0.8 | 1.11 | 3.07 | 1.08 | 1.05 | 0.36 |
| 0.93 | 1.22 | 1.43 | 1.45 | 0.93 | 0.92 | 0.97 | 0.96 | 1.1 | 0.99 | 0.84 |
| 1.09 | 1.04 | 0.83 | 1.28 | 1.09 | 0.84 | 1.2 | 0.52 | 0.75 | 1.28 | 0.7 |
| 1.28 | 0.87 | 0.89 | 1.48 | 1.28 | 0.87 | 0.98 | 1.77 | 1.02 | 0.84 | 3.24 |
| 1.35 | 0.87 | 1.01 | 0.87 | 1.35 | 1.1 | 1.06 | 6.35 | 0.91 | 0.93 | 0.82 |
| 0.75 | 0.88 | 1.06 | 1.15 | 0.75 | 0.85 | 0.99 | 1.31 | 1.01 | 1.14 | 1.01 |
| 0.89 | 0.91 | 0.89 | 1.03 | 0.89 | 1 | 1.02 | 0.92 | 1.22 | 0.93 | 0.86 |
| 0.98 | 0.88 | 0.73 | 0.89 | 0.98 | 0.84 | 1.02 | 3.52 | 0.94 | 0.78 | 0.8 |
| 1.3 | 1.08 | 1.11 | 1.27 | 1.3 | 1.3 | 1.17 | 2.27 | 1.08 | 1.12 | 1.33 |
| 1.24 | 0.96 | 0.86 | 1.48 | 1.24 | 1.1 | 0.86 | 1.5 | 1 | 0.93 | 0.84 |
| 0.96 | 0.97 | 1.31 | 1.1 | 0.96 | 0.73 | 1.13 | 0.51 | 1.56 | 1.09 | 0.47 |
| 1.17 | 0.87 | 0.85 | 1.32 | 1.17 | 0.91 | 1.09 | 1.28 | 1.06 | 0.92 | 0.92 |
| 0.88 | 1.17 | 1.15 | 1.11 | 0.88 | 1.17 | 1.03 | 2.18 | 1.08 | 2.36 | 2.59 |
| 0.88 | 0.9 | 0.84 | 0.79 | 0.88 | 1.22 | 0.9 | 2.19 | 0.79 | 1.13 | 0.67 |
| 0.29 | 1.32 | 0.74 | 0.86 | 0.29 | 1.11 | 0.8 | 1.87 | 0.4 | 0.8 | 1.03 |
| 1.01 | 0.96 | 1.08 | 1.11 | 1.01 | 1.12 | 0.94 | 0.8 | 0.96 | 0.98 | 1 |
| 0.95 | 0.93 | 1.05 | 1.15 | 0.95 | 1.08 | 1.02 | 1.06 | 0.85 | 1.14 | 1.58 |
| 1.12 | 1.28 | 1.31 | 1.42 | 1.12 | 1.32 | 1.22 | 7.66 | 1.87 | 1.18 | 0.72 |
| 3.3 | 1.03 | 0.96 | 0.79 | 3.3 | 1.03 | 1.03 | 2.43 | 1.09 | 1.06 | 0.74 |
| 1.5 | 1.1 | 0.81 | 0.84 | 1.5 | 1.05 | 0.98 | 1.15 | 0.93 | 1.06 | 0.97 |
| 1.02 | 0.97 | 1.1 | 1.26 | 1.02 | 1.14 | 1.01 | 0.95 | 1.44 | 0.73 | 1.35 |
| 1.41 | 1.02 | 0.89 | 0.63 | 1.41 | 1.33 | 1.17 | 0.96 | 0.78 | 0.61 | 1.23 |
| 1.13 | 0.94 | 1.02 | 1.17 | 1.13 | 1.06 | 1.06 | 0.86 | 0.89 | 1.07 | 1.16 |
| 1.01 | 1.03 | 0.9 | 1.01 | 1.01 | 1.18 | 0.81 | 0.34 | 0.73 | 1.19 | 0.27 |
| 1 | 0.91 | 0.91 | 1.1 | 1 | 0.89 | 0.98 | 1.81 | 0.77 | 0.97 | 0.93 |
| 1.09 | 1.1 | 0.78 | 0.92 | 1.09 | 1 | 1.04 | 1.1 | 0.99 | 1.04 | 0.78 |
| 1.31 | 1.21 | 1.53 | 1.43 | 1.31 | 1.16 | 1 | 1.98 | 0.82 | 1.43 | 0.66 |
| 0.9 | 1.14 | 1.21 | 0.87 | 0.9 | 1.08 | 0.88 | 0.99 | 1.33 | 0.98 | 1.37 |
| 1.01 | 0.99 | 0.84 | 1.16 | 1.01 | 1.05 | 1 | 1.67 | 0.99 | 1.03 | 3.08 |
| 0.83 | 0.98 | 0.81 | 0.83 | 0.83 | 1.02 | 1.07 | 0.52 | 0.9 | 0.99 | 1.12 |
| 1.24 | 0.91 | 0.94 | 1.08 | 1.24 | 1.03 | 1.11 | 1.09 | 1.04 | 0.76 | 0.98 |
| 0.95 | 0.99 | 1.09 | 1.42 | 0.95 | 0.91 | 1 | 1 | 1.07 | 0.99 | 13.94 |
| 1.06 | 1.02 | 1.09 | 1.14 | 1.06 | 1.06 | 1.01 | 0.84 | 1.06 | 0.98 | 0.98 |

| | | | | | | | | | | |
|---|---|---|---|---|---|---|---|---|---|---|
| 0.81 | 1.14 | 1.67 | 1.63 | 0.81 | 2.06 | 0.97 | 1.65 | 2.1 | 1.16 | 3.9 |
| 1.22 | 1.02 | 1.08 | 1.24 | 1.22 | 1.08 | 1.04 | 1.73 | 1.09 | 0.77 | 1.05 |
| 0.97 | 0.97 | 0.98 | 0.96 | 0.97 | 1.04 | 0.96 | 0.84 | 1.03 | 1.25 | 0.97 |
| 3.63 | 1.04 | 0.84 | 1.57 | 3.63 | 0.94 | 1.04 | 0.55 | 0.51 | 1.04 | 1 |
| 0.93 | 1.08 | 0.75 | 0.52 | 0.93 | 0.71 | 1.23 | 1.04 | 0.86 | 1.09 | 0.78 |
| 1.62 | 0.99 | 0.96 | 0.9 | 1.62 | 0.96 | 0.96 | 1.46 | 0.99 | 0.94 | 1.01 |
| 0.54 | 1.03 | 0.96 | 1.02 | 0.54 | 0.93 | 0.76 | 0.57 | 1.68 | 1.17 | 0.78 |
| 0.9 | 1.07 | 1.05 | 1.2 | 0.9 | 0.93 | 1.03 | 2.47 | 1.01 | 1 | 1.22 |
| 0.91 | 1.09 | 1 | 0.88 | 0.91 | 1.04 | 1.06 | 0.53 | 1.1 | 1.05 | 0.66 |
| 0.61 | 1.06 | 1.04 | 0.81 | 0.61 | 0.92 | 0.76 | 2.12 | 1.41 | 1.14 | 1.02 |
| 1.3 | 0.93 | 1.39 | 0.84 | 1.3 | 1.41 | 0.96 | 0.81 | 1.13 | 1.21 | 1.33 |
| 1.82 | 0.99 | 1.4 | 1.5 | 1.82 | 1.7 | 0.93 | 2.12 | 0.73 | 1.45 | 0.85 |
| 0.81 | 0.75 | 1.12 | 1.02 | 0.81 | 0.86 | 0.9 | 1.02 | 1.07 | 0.79 | 1 |
| 0.95 | 1.11 | 0.96 | 0.95 | 0.95 | 0.91 | 1.06 | 5.69 | 0.92 | 0.87 | 0.82 |
| 1.4 | 0.96 | 0.85 | 1.36 | 1.4 | 0.94 | 1.05 | 2.01 | 0.94 | 0.91 | 2.1 |
| 1.39 | 1.06 | 0.89 | 1.35 | 1.39 | 0.99 | 0.87 | 0.93 | 1.05 | 1.01 | 0.5 |
| 0.91 | 1.05 | 1.3 | 1.07 | 0.91 | 1.01 | 0.99 | 1.75 | 1.01 | 1.1 | 1.19 |
| 0.97 | 1.01 | 0.86 | 0.77 | 0.97 | 1 | 1 | 1.13 | 1.13 | 0.9 | 0.91 |
| 0.69 | 0.75 | 1.05 | 0.91 | 0.69 | 0.99 | 1.24 | 0.92 | 1.56 | 0.86 | 2.32 |
| 0.88 | 1.03 | 1.12 | 0.84 | 0.88 | 0.79 | 0.9 | 1.08 | 0.73 | 0.94 | 0.89 |
| 0.53 | 1.06 | 1.28 | 0.97 | 0.53 | 0.93 | 1.01 | 0.9 | 1.03 | 1.12 | 0.73 |
| 0.72 | 0.71 | 0.82 | 0.79 | 0.72 | 0.69 | 1.94 | 709.31 | 1.27 | 1.6 | 0.16 |
| 0.89 | 1.08 | 0.99 | 0.96 | 0.89 | 1.11 | 0.95 | 6.58 | 0.93 | 1.09 | 0.75 |
| 0.87 | 1.02 | 3.13 | 1.44 | 0.87 | 0.91 | 1.01 | 1.43 | 1.69 | 1.11 | 4.07 |
| 1.03 | 1.21 | 0.92 | 0.98 | 1.03 | 1.34 | 0.93 | 2.31 | 0.9 | 0.9 | 1.35 |
| 2.78 | 0.54 | 0.48 | 2.5 | 2.78 | 2.64 | 0.82 | 0.22 | 1.04 | 1.36 | 8.09 |
| 1.17 | 1.04 | 0.93 | 1.36 | 1.17 | 0.97 | 0.99 | 1.04 | 1.06 | 1.1 | 1.46 |
| 1.11 | 0.94 | 1.1 | 1.09 | 1.11 | 1.03 | 1.04 | 0.68 | 1.12 | 1.05 | 1.02 |
| 0.95 | 0.89 | 0.99 | 1.07 | 0.95 | 0.93 | 1.01 | 0.83 | 0.94 | 0.92 | 1.03 |
| 1.06 | 0.82 | 1 | 0.98 | 1.06 | 0.93 | 1.06 | 1.17 | 1.2 | 0.81 | 1.1 |
| 0.84 | 0.9 | 1.2 | 1.38 | 0.84 | 0.8 | 1.18 | 1.16 | 0.99 | 1.25 | 1.12 |
| 0.89 | 0.94 | 0.87 | 0.9 | 0.89 | 0.91 | 1.14 | 1.08 | 0.97 | 0.97 | 0.88 |
| 0.86 | 0.86 | 0.78 | 1.58 | 0.86 | 1.31 | 1.08 | 5.57 | 0.69 | 1.03 | 1.03 |

| | | | | | | | | | | |
|---|---|---|---|---|---|---|---|---|---|---|
| 0.83 | 1.06 | 0.88 | 1.23 | 0.83 | 2.03 | 0.96 | 0.11 | 0.96 | 1.12 | 1.09 |
| 1.12 | 0.95 | 1.05 | 0.82 | 1.12 | 1.07 | 1.05 | 1.4 | 1.14 | 0.74 | 1.5 |
| 1.06 | 0.75 | 1.02 | 1.02 | 1.06 | 1 | 1.16 | 1.05 | 1.02 | 0.85 | 1.08 |
| 0.66 | 0.75 | 1.06 | 0.88 | 0.66 | 1.05 | 0.74 | 1.33 | 2.21 | 0.93 | 0.73 |
| 0.85 | 1.04 | 1.62 | 1.32 | 0.85 | 1.13 | 1.11 | 1.83 | 1.5 | 0.99 | 0.85 |
| 0.64 | 0.74 | 0.88 | 0.77 | 0.64 | 0.93 | 1.04 | 2.28 | 0.88 | 0.95 | 1.98 |
| 0.83 | 0.87 | 0.95 | 0.86 | 0.83 | 1.29 | 0.85 | 1.1 | 0.76 | 0.94 | 0.97 |
| 1.13 | 1.05 | 0.85 | 1.07 | 1.13 | 0.99 | 0.84 | 0.49 | 1.21 | 1.1 | 0.72 |
| 0.99 | 0.97 | 2.14 | 2.57 | 0.99 | 1.18 | 0.94 | 1.64 | 1.8 | 1.14 | 3.12 |
| 0.98 | 1.26 | 0.79 | 2.53 | 0.98 | 0.68 | 0.86 | 0.56 | 0.83 | 0.94 | 0.85 |
| 1.26 | 1.52 | 0.82 | 0.85 | 1.26 | 0.82 | 0.95 | 1.13 | 1.02 | 0.98 | 0.74 |
| 0.82 | 0.95 | 0.89 | 1.03 | 0.82 | 1.04 | 1.03 | 0.39 | 0.9 | 0.81 | 0.62 |
| 1 | 1.18 | 0.81 | 1.35 | 1 | 1.07 | 1.01 | 1.07 | 0.66 | 1.36 | 0.93 |
| 4.58 | 1.18 | 1.06 | 0.4 | 4.58 | 0.91 | 1.04 | 5.92 | 1.11 | 0.96 | 0.86 |
| 1.06 | 0.69 | 0.83 | 0.9 | 1.06 | 0.89 | 0.92 | 1.22 | 1.1 | 0.92 | 0.97 |
| 0.81 | 0.62 | 1.03 | 0.59 | 0.81 | 1.28 | 1.02 | 1.69 | 1.02 | 1.3 | 1.2 |
| 0.6 | 1.07 | 0.95 | 1.09 | 0.6 | 0.79 | 1.07 | 0.3 | 1.05 | 1.34 | 0.74 |
| 1.14 | 0.94 | 0.81 | 1.17 | 1.14 | 0.95 | 0.92 | 0.55 | 1.05 | 1.13 | 1.01 |
| 1.4 | 1.14 | 0.97 | 1.2 | 1.4 | 0.93 | 0.79 | 0.3 | 0.63 | 1.02 | 2.72 |
| 1.14 | 0.79 | 1.28 | 0.23 | 1.14 | 1.23 | 0.74 | 0.03 | 0.25 | 1.4 | 0.9 |
| 0.75 | 0.91 | 0.91 | 1.07 | 0.75 | 1.15 | 0.91 | 1.16 | 1.22 | 1.14 | 1.78 |
| 0.6 | 1.01 | 0.99 | 0.83 | 0.6 | 0.89 | 0.78 | 1.27 | 1.54 | 1.15 | 0.91 |
| 1.07 | 1.01 | 1.71 | 0.83 | 1.07 | 0.73 | 1.63 | 0.81 | 3.02 | 0.84 | 1.05 |
| 0.59 | 1.35 | 0.91 | 3.08 | 0.59 | 1.02 | 0.96 | 0 | 1.05 | 0.76 | 1.42 |
| 0.89 | 0.96 | 0.93 | 1.15 | 0.89 | 1.18 | 0.99 | 1.04 | 0.81 | 1.15 | 1 |
| 1.11 | 1.05 | 0.78 | 1.32 | 1.11 | 1.04 | 1.1 | 0.99 | 1.09 | 1.14 | 0.83 |
| 1.19 | 1 | 0.9 | 1.04 | 1.19 | 0.56 | 0.93 | 0.86 | 0.8 | 1.46 | 0.59 |
| 1.19 | 1.2 | 0.99 | 1.25 | 1.19 | 1.11 | 0.87 | 0.2 | 0.75 | 1.11 | 0.94 |
| 0.61 | 1.52 | 0.98 | 1.02 | 0.61 | 0.8 | 1.13 | 0.85 | 2.05 | 1.19 | 0.79 |
| 0.5 | 0.47 | 0.42 | 0.79 | 0.5 | 2.61 | 1.16 | 1.13 | 1.62 | 0.14 | 1.66 |
| 1.41 | 0.87 | 1.47 | 0.68 | 1.41 | 0.97 | 0.92 | 2 | 0.87 | 0.73 | 1.83 |
| 0.98 | 0.94 | 1.18 | 0.82 | 0.98 | 0.57 | 0.99 | 0.93 | 0.3 | 0.63 | 0.93 |
| 0.64 | 3.12 | 1.61 | 1.67 | 0.64 | 1.85 | 0.98 | 2.11 | 1.2 | 1.84 | 1.13 |

| | | | | | | | | | | |
|---|---|---|---|---|---|---|---|---|---|---|
| 0.91 | 1.03 | 0.99 | 1.1 | 0.91 | 0.86 | 0.96 | 4.12 | 0.99 | 0.26 | 1.34 |
| 0.66 | 0.94 | 1.16 | 1.27 | 0.66 | 0.94 | 1.01 | 0.75 | 1.13 | 1.16 | 0.97 |
| 5.4 | 1.34 | 1.06 | 2.16 | 5.4 | 0.52 | 2.13 | 2.87 | 0.8 | 0.71 | 0.94 |
| 0.71 | 0.82 | 0.74 | 1.54 | 0.71 | 0.65 | 0.93 | 0.23 | 0.28 | 1.35 | 1.05 |
| 0.52 | 0.99 | 0.96 | 1.08 | 0.52 | 0.92 | 0.98 | 56.41 | 1.57 | 0.84 | 0.92 |
| 1.08 | 0.98 | 1.23 | 1.03 | 1.08 | 1.72 | 1.07 | 2.31 | 1.08 | 1.08 | 1.1 |
| 0.94 | 1.02 | 1.03 | 1.12 | 0.94 | 1.27 | 1.05 | 0.89 | 1 | 1.03 | 1.1 |
| 1.01 | 1.05 | 0.92 | 0.98 | 1.01 | 0.79 | 1.04 | 2.26 | 0.91 | 1.01 | 0.95 |
| 0.94 | 0.88 | 0.92 | 1.43 | 0.94 | 1.28 | 1.01 | 1.11 | 0.83 | 1.11 | 1.03 |
| 1.2 | 1.08 | 1.11 | 1.01 | 1.2 | 1.4 | 1.03 | 1.06 | 1.65 | 1.15 | 0.66 |
| 1.26 | 1.08 | 1.17 | 1.15 | 1.26 | 1.31 | 1 | 2.73 | 0.99 | 1.04 | 1.52 |
| 6.6 | 13.57 | 5.27 | 6.89 | 6.6 | 1.6 | 0.77 | 0.29 | 3.78 | 0.09 | 0.51 |
| 1.01 | 1.14 | 1.05 | 1.21 | 1.01 | 1.37 | 1.07 | 0.87 | 0.96 | 1.23 | 0.97 |
| 0.52 | 0.87 | 1.21 | 0.66 | 0.52 | 1.05 | 1.18 | 26.12 | 0.33 | 1.17 | 0.69 |

| 211110_s_at | 202092_s_at | 204798_at | 211012_s_at | 202431_s_at | 209054_s_at | 214794_at | 210004_at | 202778_s_at | 204602_at | 209341_s_at |
|---|---|---|---|---|---|---|---|---|---|---|
| 0.89 | 1.16 | 1.93 | 1.39 | 1.04 | 0.88 | 1.01 | 0.64 | 0.86 | 1.4 | 1.02 |
| 0.76 | 0.97 | 1.54 | 0.76 | 0.41 | 0.71 | 0.81 | 1.17 | 1.01 | 0.91 | 1.16 |
| 1.68 | 1.04 | 1.06 | 0.65 | 1.13 | 0.88 | 0.96 | 1.59 | 4.32 | 1.41 | 1.85 |
| 1.02 | 0.99 | 1.04 | 1.88 | 1.17 | 0.82 | 1.02 | 1.81 | 0.96 | 0.74 | 1.14 |
| 1.54 | 1.21 | 1.16 | 1.41 | 1.22 | 1.7 | 1.2 | 1.14 | 0.69 | 1.08 | 1.51 |
| 1 | 1.23 | 0.7 | 0.81 | 735.49 | 0.84 | 1.35 | 2.05 | 1.2 | 0.57 | 0.71 |
| 1 | 1.48 | 0.07 | 1.25 | 0.85 | 0.75 | 1.82 | 0.44 | 1.06 | 1.61 | 1.34 |
| 0.96 | 1.16 | 0.9 | 0.99 | 1.08 | 0.94 | 1.02 | 0.58 | 0.92 | 1.43 | 0.95 |
| 0.66 | 1.06 | 0.6 | 1.09 | 0.82 | 0.92 | 0.94 | 0.67 | 0.99 | 4.07 | 1 |
| 268.62 | 1.08 | 0.27 | 7.93 | 1.95 | 0.75 | 1.27 | 5.43 | 0.63 | 1.43 | 0.75 |
| 0.87 | 0.81 | 1.17 | 0.45 | 0.89 | 0.96 | 0.93 | 0.5 | 1.23 | 0.68 | 1.27 |
| 0.22 | 1.22 | 1.25 | 0.68 | 0.97 | 1.37 | 1.03 | 0.26 | 0.87 | 0.91 | 1.1 |
| 1.02 | 0.98 | 0.85 | 0.93 | 1.03 | 0.93 | 1.11 | 0.9 | 0.95 | 0.53 | 1.16 |
| 10.4 | 0.24 | 2.15 | 0.35 | 0.15 | 0.48 | 2.33 | 17.12 | 11.05 | 0.06 | 0.26 |
| 0.7 | 1.98 | 0.85 | 0.66 | 1.71 | 1.31 | 1 | 1.74 | 1.06 | 1.11 | 1.47 |
| 1.12 | 0.99 | 1.76 | 0.68 | 0.92 | 0.92 | 0.81 | 1.16 | 1.13 | 0.49 | 1.21 |
| 1.14 | 1.07 | 2.98 | 1.9 | 0.83 | 1.2 | 0.84 | 1.3 | 0.99 | 1.03 | 1.25 |
| 1.52 | 1.17 | 4.28 | 1.29 | 0.74 | 1 | 0.99 | 11.86 | 1.63 | 0.4 | 1.05 |
| 0.82 | 1.06 | 0.53 | 0.15 | 0.22 | 0.84 | 0.71 | 0.54 | 0.72 | 0.25 | 1.32 |
| 1.2 | 0.98 | 1.27 | 3.92 | 0.74 | 0.59 | 1 | 2.35 | 1.15 | 2.69 | 1.39 |
| 1.67 | 2.49 | 1.26 | 2.3 | 0.49 | 0.79 | 0.65 | 2.07 | 1.21 | 0.81 | 0.49 |
| 10.97 | 1.47 | 2.98 | 3.24 | 0.88 | 0.91 | 0.57 | 9.3 | 0.8 | 2.05 | 0.93 |
| 1.5 | 0.84 | 1.57 | 0.94 | 1.41 | 0.85 | 0.85 | 2.97 | 1.01 | 3.05 | 1.07 |
| 1.42 | 0.75 | 1.07 | 1.57 | 0.75 | 0.69 | 0.81 | 0.52 | 0.85 | 1.89 | 1.21 |
| 0.42 | 1.14 | 0.04 | 7.42 | 6.79 | 1.33 | 0.77 | 8.67 | 0.01 | 94.97 | 1.79 |
| 0.87 | 0.97 | 0.44 | 0.95 | 0.76 | 1.26 | 1.67 | 0.56 | 0.64 | 2.77 | 0.85 |
| 1.14 | 1.12 | 10.86 | 1.22 | 0.76 | 1.04 | 0.97 | 0.69 | 1.4 | 1.01 | 1.14 |
| 3.7 | 2.34 | 1.54 | 24.79 | 1.03 | 0.78 | 1.1 | 1.24 | 0.8 | 4.7 | 0.97 |
| 1.32 | 0.83 | 3.77 | 1.81 | 3.43 | 1.41 | 0.81 | 3.74 | 1.72 | 3.61 | 0.5 |
| 1.11 | 0.9 | 0.91 | 1.18 | 3.16 | 0.88 | 0.87 | Inf | 1.06 | 2.06 | 0.83 |

| | | | | | | | | | | |
|---|---|---|---|---|---|---|---|---|---|---|
| 2 | 0.98 | 0.75 | 1.05 | 1.37 | 0.92 | 1.53 | 0.29 | 0.93 | 0.77 | 0.69 |
| 3.1 | 0.97 | 1.25 | 0.96 | 4.73 | 1.54 | 10.02 | 89.53 | 1.52 | 0.95 | 3.19 |
| 1.18 | 0.96 | 0.95 | 2 | 0.71 | 0.99 | 0.77 | 1.79 | 1.18 | 0.78 | 1.26 |
| 1.42 | 0.76 | 6.78 | 0.27 | 0.55 | 2.05 | 1.84 | 0.4 | 0.68 | 0.99 | 0.76 |
| 2.38 | 0.49 | 0.86 | 1.67 | 1.69 | 0.09 | 1.15 | 0.56 | 0.3 | 0.38 | 2.42 |
| 5.49 | 0.78 | 1.55 | 0.44 | 1.53 | 1.27 | 0.92 | 23.91 | 0.62 | 11.11 | 0.78 |
| 0.41 | 0.58 | 1.17 | 0.8 | 0.97 | 0.65 | 0.91 | 0.6 | 0.93 | 0.01 | 0.93 |
| 1.35 | 1.1 | 0.8 | 0.5 | 1.17 | 0.91 | 0.99 | 0.92 | 0.72 | 1.04 | 0.78 |
| 2.33 | 1.39 | 0.37 | 1.65 | 0.55 | 1.27 | 0.88 | 0.16 | 0.9 | 0.78 | 0.89 |
| 0.69 | 1.05 | 0.03 | 3.12 | 0.48 | 0.7 | 0.55 | 9.5 | 1.15 | 0.54 | 1.88 |
| 0.42 | 1.11 | 1.11 | 1.44 | 0.71 | 1.16 | 1.36 | 0.47 | 0.87 | 14.66 | 1.12 |
| 3.39 | 1.02 | 0.99 | 0.98 | 0.47 | 0.97 | 1.11 | 0.83 | 1.07 | 0.98 | 0.95 |
| 0.42 | 0.86 | 1.06 | 0.69 | 0.44 | 0.36 | 0.47 | 0.63 | 2.59 | 0.48 | 1.41 |
| 4.21 | 1.13 | 1.51 | 0.75 | 2.84 | 1.19 | 0.8 | 1.84 | 1.78 | 2.66 | 1.49 |
| 0.58 | 0.89 | 0.92 | 0.67 | 0.6 | 0.53 | 0.63 | 0.53 | 2 | 0.52 | 1.22 |
| 0.93 | 0.81 | 1.35 | 1.8 | 1.23 | 1.04 | 1 | 1.39 | 1.22 | 4.94 | 1.59 |
| 1.11 | 1.14 | 1.04 | 0.59 | 0.86 | 1.25 | 0.94 | 1.12 | 0.97 | 1.17 | 1.14 |
| 3.42 | 1.04 | 1.25 | 2.39 | 1.38 | 1.23 | 1.06 | 1.65 | 1.28 | 1.69 | 1.1 |
| 0.68 | 0.84 | 0.85 | 0.78 | 0.9 | 0.96 | 0.95 | 1.01 | 1.04 | 0.87 | 1.06 |
| 2.52 | 0.82 | 2.38 | 1.19 | 6.01 | 1.58 | 1.05 | 0.52 | 0.81 | 0.54 | 0.89 |
| 0.55 | 0.92 | 0.86 | 0.68 | 0.74 | 1.03 | 0.88 | 0.2 | 1.07 | 0.82 | 0.81 |
| 0.96 | 0.93 | 1.71 | 2.01 | 5.45 | 0.98 | 1.63 | 1.64 | 0.85 | 0.56 | 1.06 |
| 1.59 | 0.93 | 1.15 | 0.53 | 1.2 | 0.79 | 0.73 | 12.88 | 1.14 | 1.69 | 0.7 |
| 1.04 | 1.24 | 1.05 | 0.93 | 0.64 | 1.15 | 0.84 | 6.07 | 0.97 | 2.14 | 1.13 |
| 0.7 | 1.03 | 0.79 | 1.24 | 1.96 | 0.82 | 0.95 | 0.08 | 0.95 | 0.33 | 0.66 |
| 0.48 | 0.82 | 23.86 | 1.14 | 0.62 | 1.81 | 1.95 | 0.52 | 0.76 | 0.78 | 1.16 |
| 3.34 | 1.51 | 0.68 | 1.53 | 0.59 | 1.17 | 1.08 | 3.96 | 0.47 | 1.01 | 1.15 |
| 1.82 | 1.31 | 1.75 | 1.63 | 0.5 | 1.25 | 1.39 | 0.97 | 1.98 | 2.22 | 2.16 |
| 0.89 | 1.02 | 0.54 | 0.74 | 0.82 | 0.88 | 0.65 | 2.12 | 1.06 | 0.69 | 1.43 |
| 1.45 | 0.76 | 1.23 | 0.8 | 0.46 | 0.47 | 0.58 | 0.73 | 2.51 | 0.26 | 1.22 |
| 0.92 | 1.45 | 0.8 | 4.06 | 0.67 | 1.15 | 0.61 | 8.8 | 0.79 | 3.22 | 0.92 |
| 1.46 | 0.96 | 1.05 | 1.49 | 1.04 | 0.99 | 0.69 | 2.53 | 1.32 | 1.14 | 1.61 |
| 0.84 | 1.07 | 0.6 | 3.67 | 142.34 | 0.8 | 0.76 | 0.9 | 1.06 | 1.76 | 1.02 |

| | | | | | | | | | | |
|---|---|---|---|---|---|---|---|---|---|---|
| 1.46 | 1.15 | 2 | 0.84 | 0.91 | 0.93 | 0.8 | 0.59 | 1.26 | 0.44 | 1.04 |
| 0.88 | 1 | 2.41 | 1.38 | 1.85 | 0.9 | 1.57 | 7.21 | 1.34 | 6 | 0.99 |
| 5.07 | 0.99 | 0.96 | 0.97 | 1 | 1.06 | 0.89 | 0.96 | 0.97 | 10.33 | 1 |
| 1.33 | 0.94 | 1.64 | 2.25 | 1.37 | 1.15 | 0.99 | 1.41 | 1.25 | 6.41 | 0.88 |
| 1.49 | 0.94 | 0.88 | 1 | 1.04 | 0.95 | 0.99 | 2.09 | 1.04 | 1.02 | 1.21 |
| 1.41 | 1.7 | 0.99 | 0.59 | 1.04 | 1.09 | 1.15 | 0.62 | 1.61 | 0.21 | 1.65 |
| 2.01 | 2.13 | 0.43 | 0.87 | 1.83 | 1.82 | 0.44 | 2.5 | 0.6 | 0.79 | 0.6 |
| 1.22 | 1.15 | 0.72 | 1.47 | 1.1 | 0.99 | 1.16 | 1.11 | 0.74 | 1.2 | 1.26 |
| 0.97 | 1.5 | 0.73 | 1.17 | 1.08 | 0.72 | 0.96 | 0.64 | 1.13 | 0.54 | 6.85 |
| 0.9 | 0.95 | 1.46 | 1.37 | 0.8 | 1.1 | 1.02 | 0.74 | 1.2 | 1.11 | 1.61 |
| 3.59 | 1.34 | 2.23 | 0.75 | 2.8 | 1.15 | 0.92 | 0.8 | 1.95 | 2.12 | 0.5 |
| 12.2 | 0.89 | 1.47 | 1.77 | 0.74 | 0.8 | 0.88 | 0.34 | 0.75 | 4.32 | 1.07 |
| 1.44 | 0.9 | 0.83 | 1.26 | 1.33 | 0.7 | 1.2 | 1.31 | 1.07 | 2.19 | 1.35 |
| 2.24 | 0.77 | 1.41 | 1.43 | 2 | 0.97 | 0.96 | 1.03 | 1.15 | 1.18 | 0.99 |
| 4.8 | 1.17 | 1.29 | 2.02 | 1 | 0.94 | 1.18 | 0.5 | 0.76 | 0.56 | 0.87 |
| 0.02 | 0.64 | 0.14 | 4.34 | 52.03 | 1.29 | 0.94 | 20.38 | 5.5 | 30.85 | 9.99 |
| 1.8 | 1.09 | 0.33 | 0.38 | 0.53 | 1.09 | 0.99 | 7.18 | 0.84 | 11.04 | 1.44 |
| 0.24 | 0.32 | 2.86 | 0.62 | 0.13 | 0.85 | 0.62 | 0.71 | 2.41 | 5.05 | 1.29 |
| 1.59 | 0.91 | 0.85 | 1.49 | 1.22 | 0.87 | 1.14 | 0.48 | 1.3 | 1.05 | 1.21 |
| 12.73 | 1.1 | 0.8 | 6.78 | 1.02 | 0.86 | 0.88 | 1.43 | 0.91 | 6.42 | 1.04 |
| 1.41 | 1.4 | 0.73 | 6.47 | 0.33 | 0.45 | 1.17 | 1.39 | 0.74 | 1.75 | 1.25 |
| 0.58 | 1.17 | 1.35 | 1.18 | 1.17 | 1.12 | 1.18 | 3.64 | 0.85 | 2.02 | 1.2 |
| 1.02 | 1.53 | 0.21 | 0.98 | 0.87 | 0.85 | 1.48 | 0.31 | 1.03 | 1.57 | 0.93 |
| 2.08 | 1.08 | 39.08 | 0.64 | 0.51 | 3.22 | 1.87 | 3.02 | 0.56 | 0.5 | 0.8 |
| 2.03 | 1.1 | 0.87 | 0.95 | 1.04 | 1.16 | 0.87 | 0.19 | 1.15 | 0.83 | 1.17 |
| 0.94 | 1.17 | 0.65 | 0.81 | 1.07 | 0.89 | 0.81 | 1.32 | 0.78 | 0.97 | 0.9 |
| 0.78 | 0.94 | 0.9 | 1.37 | 1.28 | 0.92 | 0.97 | 2.99 | 0.69 | 0.95 | 0.99 |
| 3.32 | 0.87 | 0.95 | 0.82 | 1.31 | 1.11 | 1.09 | 1.41 | 1.01 | 14.97 | 1 |
| 0.96 | 1.21 | 0.91 | 1.85 | 1.07 | 0.93 | 1.18 | 1.16 | 1.2 | 1.67 | 0.79 |
| 3.24 | 1.02 | 1.2 | 1.36 | 1.03 | 0.87 | 1.27 | 0.84 | 0.9 | 1.43 | 0.89 |
| 0.91 | 1.11 | 0.92 | 1.17 | 0.83 | 0.99 | 1.17 | 3.13 | 1.05 | 0.91 | 1.07 |
| 1.46 | 1.17 | 0.78 | 0.97 | 0.62 | 0.56 | 0.99 | 0.81 | 1.23 | 0.95 | 0.73 |
| 5.3 | 1.12 | 0.95 | 1.22 | 0.77 | 0.79 | 1.05 | 1.51 | 1.42 | 1.67 | 1.39 |

| | | | | | | | | | | |
|---|---|---|---|---|---|---|---|---|---|---|
| 1.59 | 0.92 | 1.19 | 1.72 | 2.26 | 1.2 | 1.49 | 1.51 | 12.62 | 2.2 | 1.31 |
| 6.06 | 0.88 | 0.82 | 2.25 | 0.92 | 0.96 | 1.1 | 1.79 | 0.76 | 0.57 | 0.83 |
| 2.48 | 1.64 | 1.09 | 0.95 | 1.59 | 1.07 | 0.96 | 0.86 | 0.73 | 1.98 | 0.75 |
| 1.09 | 1.08 | 0.89 | 0.86 | 0.6 | 1.28 | 0.87 | 0.72 | 0.79 | 0.68 | 4.71 |
| 1.13 | 1.67 | 1.67 | 0.84 | 1.17 | 0.67 | 1.26 | 0.45 | 2.02 | 0.21 | 1.72 |
| 2.22 | 1.18 | 0.71 | 1.65 | 0.62 | 0.53 | 1.29 | 1.74 | 0.53 | 6.83 | 0.5 |
| 1.09 | 0.93 | 0.76 | 3.18 | 1.52 | 0.93 | 0.87 | 2.81 | 1.18 | 0.87 | 1.47 |
| 1.74 | 0.62 | 1.27 | 0.95 | 0.71 | 0.98 | 0.76 | 0.81 | 1.31 | 6.71 | 0.94 |
| 0.97 | 0.65 | 0.49 | 0.83 | 1.65 | 0.78 | 1.17 | 0.86 | 1.37 | 0.96 | 0.57 |
| 0.77 | 1 | 0.82 | 1.41 | 1.39 | 0.9 | 1.03 | 19.53 | 1.08 | 1.49 | 1.01 |
| 1.24 | 0.97 | 2.41 | 0.98 | 0.65 | 1.07 | 1.64 | 2.69 | 0.91 | 0.83 | 0.91 |
| 1.3 | 1.12 | 1.99 | 0.54 | 1.26 | 1.16 | 1.06 | 0.9 | 0.94 | 1.14 | 1.14 |
| 0.66 | 0.69 | 1.56 | 1.06 | 1.03 | 1.58 | 1.05 | 0.92 | 1 | 0.98 | 0.73 |
| 1.23 | 0.95 | 5.13 | 1.18 | 0.84 | 1.76 | 1.57 | 0.44 | 0.76 | 0.94 | 0.87 |
| 0.72 | 0.92 | 0.91 | 2.76 | 2.56 | 0.92 | 0.84 | 0.83 | 1.41 | 1.65 | 1.26 |
| 1.26 | 0.82 | 0.96 | 1.73 | 1.01 | 1.06 | 1.18 | 1.68 | 1.35 | 1.71 | 1.24 |
| 1.21 | 0.48 | 1 | 1.06 | 1.32 | 1.1 | 0.9 | 1.7 | 0.98 | 1.02 | 0.92 |
| 1.27 | 0.98 | 1.39 | 1.98 | 1.75 | 1.35 | 1.16 | 1.44 | 1.22 | 5.89 | 1.19 |
| 0.46 | 1.04 | 0.88 | 1.38 | 0.97 | 0.63 | 0.86 | 1.62 | 0.74 | 1.15 | 1.2 |
| 0.69 | 0.78 | 2.12 | 0.36 | 0.81 | 1.33 | 0.99 | 0.71 | 1.2 | 0.79 | 0.7 |
| 0.98 | 0.93 | 0.47 | 0.73 | 0.85 | 1.16 | 1.51 | 1.24 | 0.79 | 1.13 | 0.98 |
| 1.78 | 1.46 | 0.94 | 1.34 | 1.09 | 0.98 | 1.08 | 0.3 | 0.95 | 0.66 | 0.71 |
| 1.37 | 1.15 | 1.34 | 2.63 | 1.21 | 1.29 | 1.13 | 0.93 | 0.92 | 1.21 | 1.1 |
| 1.01 | 0.88 | 0.69 | 0.62 | 1.73 | 1.71 | 1.41 | 8 | 1.43 | 1.1 | 1.86 |
| 1.12 | 0.99 | 1.25 | 1.13 | 0.87 | 1.18 | 0.46 | 1.83 | 1.29 | 0.33 | 0.88 |
| 0.58 | 0.86 | 1.44 | 1.25 | 1.76 | 0.97 | 0.87 | 3.67 | 1.27 | 1.7 | 1.08 |
| 1.71 | 1.04 | 1.02 | 7 | 1.23 | 1.12 | 1.15 | 0.54 | 1.09 | 0.81 | 1.29 |
| 0.68 | 1.02 | 2.5 | 1.57 | 2.06 | 1.19 | 0.68 | 1.3 | 1.22 | 0.6 | 1.33 |
| 0.91 | 1.26 | 1.39 | 1.99 | 1.32 | 2.88 | 1.5 | 13.15 | 0.41 | 0.62 | 0.79 |
| 0.92 | 0.86 | 0.94 | 1.07 | 0.9 | 0.97 | 1.03 | 0.97 | 1.01 | 0.76 | 0.82 |
| 0.68 | 1.04 | 0.82 | 1.04 | 1.04 | 1.06 | 1.16 | 1.05 | 0.9 | 10.5 | 0.84 |
| 0.88 | 1 | 2.41 | 1.38 | 1.85 | 0.9 | 1.57 | 7.21 | 1.34 | 6 | 0.99 |
| 0.82 | 1.41 | 0.7 | 1.07 | 1.59 | 0.96 | 1.14 | 1.38 | 1.01 | 0.79 | 1.09 |

| | | | | | | | | | | |
|---|---|---|---|---|---|---|---|---|---|---|
| 0.85 | 1.28 | 1.47 | 1.12 | 1.3 | 0.93 | 1.27 | 0.91 | 1.08 | 0.83 | 0.89 |
| 0.95 | 0.88 | 0.67 | 1.88 | 1.04 | 0.95 | 1.02 | 0.73 | 0.98 | 0.92 | 0.82 |
| 1.03 | 1.35 | 0.75 | 0.94 | 1.12 | 0.77 | 0.77 | 0.43 | 1.28 | 2.13 | 1.09 |
| 2.86 | 1.17 | 0.99 | 0.84 | 1.08 | 0.79 | 0.95 | 0.93 | 0.98 | 0.59 | 0.91 |
| 1.03 | 1.1 | 0.4 | 0.82 | 0.97 | 1.04 | 0.9 | 1.61 | 1.01 | 1.64 | 0.98 |
| 0.43 | 0.96 | 0.52 | 0.78 | 1.1 | 0.97 | 1.13 | 0.95 | 1.15 | 0.64 | 0.9 |
| 0.95 | 0.88 | 0.84 | 1.07 | 1.19 | 0.86 | 0.95 | 0.43 | 1.12 | 0.56 | 1 |
| 1.01 | 0.75 | 0.99 | 0.86 | 0.84 | 1.05 | 0.78 | 1.62 | 1.37 | 0.64 | 0.92 |
| 1.31 | 1.01 | 1.4 | 1.23 | 0.65 | 1.36 | 0.84 | 2.75 | 1.15 | 0.22 | 1.23 |
| 3.27 | 0.97 | 0.87 | 0.97 | 0.84 | 0.91 | 0.94 | 0.88 | 1.13 | 1.26 | 1.06 |
| 0.38 | 1.22 | 0.42 | 2.88 | 1.09 | 1.22 | 1.36 | 0.93 | 0.95 | 1.12 | 1.14 |
| 0.57 | 1.1 | 1.04 | 5.61 | 1.15 | 0.82 | 0.83 | 1.16 | 1.11 | 1.25 | 1.06 |
| 1.05 | 1.03 | 1.5 | 3.17 | 1.6 | 1.22 | 1.07 | 1.03 | 1.31 | 5.8 | 1.07 |
| 1.42 | 0.76 | 0.85 | 3.76 | 0.93 | 0.9 | 0.68 | 1.02 | 1.11 | 2.46 | 1.09 |
| 1 | 0.8 | 1.22 | 0.95 | 0.41 | 0.79 | 0.4 | 1.32 | 1.57 | 0.92 | 1.38 |
| 1.08 | 1.07 | 1.09 | 1.17 | 0.87 | 0.97 | 0.92 | 0.77 | 1.01 | 0.71 | 1.1 |
| 1.03 | 0.88 | 0.83 | 0.7 | 0.92 | 1 | 0.98 | 2.27 | 1.04 | 0.66 | 0.91 |
| 1.48 | 0.68 | 1.33 | 1.19 | 2.9 | 1.19 | 1.84 | 0.91 | 1.05 | 1.94 | 0.9 |
| 1.16 | 1.08 | 0.95 | 0.94 | 1.03 | 0.83 | 0.92 | 0.96 | 0.78 | 1.34 | 0.96 |
| 2.13 | 1.73 | 0.35 | 0.97 | 0.77 | 1.02 | 0.92 | 2.29 | 1.16 | 1.28 | 0.84 |
| 3.33 | 0.78 | 0.24 | 0.94 | 0.49 | 0.84 | 1.21 | 0.99 | 1.59 | 0.51 | 1.04 |
| 1.77 | 0.71 | 0.19 | 4.39 | 0.46 | 0.9 | 0.75 | 0.79 | 1.17 | 1.85 | 1.21 |
| 1.84 | 1.1 | 1.29 | 1.27 | 0.85 | 1.13 | 1 | 1.07 | 0.94 | 1.87 | 1.06 |
| 0.81 | 0.88 | 1.41 | 1.41 | 0.68 | 0.93 | 0.69 | 1.71 | 0.95 | 0.23 | 0.94 |
| 1.01 | 1.21 | 0.53 | 0.99 | 0.94 | 0.61 | 1.84 | 1.04 | 1 | 0.87 | 1.38 |
| 1.99 | 0.95 | 4.45 | 1.07 | 0.89 | 1.1 | 0.68 | 0.86 | 1.12 | 2.22 | 1.15 |
| 0.92 | 0.89 | 0.86 | 1.5 | 0.88 | 0.65 | 1.98 | 0.75 | 1.1 | 0.87 | 1.29 |
| 1.24 | 0.82 | 0.67 | 0.77 | 1.4 | 0.92 | 1.07 | 1.28 | 0.9 | 0.46 | 0.82 |
| 1.25 | 1.1 | 0.87 | 1.66 | 0.67 | 0.77 | 0.85 | 0.87 | 1.07 | 0.91 | 1.07 |
| 2.04 | 1.12 | 0.48 | 1.09 | 0.93 | 0.94 | 0.83 | 0.7 | 0.82 | 0.34 | 1.19 |
| 1.35 | 1 | 0.85 | 0.89 | 0.72 | 0.91 | 1.19 | 1.61 | 0.97 | 2.51 | 0.81 |
| 0.98 | 1 | 0.96 | 1.07 | 1.01 | 0.95 | 1.15 | 1.03 | 0.95 | 1.91 | 1.01 |
| 0.82 | 0.98 | 0.99 | 1.35 | 0.94 | 0.99 | 0.99 | 0.84 | 0.97 | 0.94 | 1.1 |

| | | | | | | | | | | |
|---|---|---|---|---|---|---|---|---|---|---|
| 0.6 | 1.06 | 0.97 | 2.43 | 0.81 | 1.05 | 1.35 | 0.33 | 1.21 | 0.6 | 6.69 |
| 2.08 | 0.91 | 1.08 | 0.97 | 0.89 | 1.01 | 1.1 | 1.25 | 0.88 | 2.75 | 0.82 |
| 0.81 | 0.9 | 1.01 | 1.04 | 0.96 | 1.18 | 0.95 | 7.52 | 0.92 | 1 | 1.01 |
| 1.24 | 0.78 | 2.34 | 2.85 | 0.77 | 0.96 | 0.66 | 0.97 | 1.65 | 0.69 | 1.19 |
| 0.6 | 0.91 | 2.98 | 1.27 | 0.68 | 1.48 | 0.96 | 4.39 | 0.83 | 1.68 | 0.51 |
| 1.39 | 1.04 | 0.99 | 1.21 | 0.92 | 0.89 | 1.01 | 0.96 | 0.94 | 0.92 | 0.99 |
| 1.82 | 1.06 | 0.81 | 1.08 | 1.4 | 0.92 | 1.42 | 0.34 | 0.95 | 0.76 | 0.7 |
| 0.61 | 0.97 | 0.94 | 5.91 | 2.13 | 1.18 | 1 | 0.75 | 1.19 | 0.95 | 0.93 |
| 5.77 | 0.98 | 1.8 | 1.84 | 0.71 | 0.96 | 1.01 | 0.96 | 1.1 | 1.2 | 0.94 |
| 1.53 | 1.08 | 0.81 | 1.01 | 1.63 | 0.97 | 1.55 | 0.24 | 1.01 | 1.05 | 0.68 |
| 0.11 | 1.02 | 2.28 | 1.15 | 0.9 | 1.59 | 1.89 | 0.36 | 1.39 | 1.18 | 0.73 |
| 0.67 | 0.8 | 0.91 | 0.68 | 0.93 | 1.09 | 2.48 | 1.54 | 0.99 | 2.1 | 0.83 |
| 0.68 | 1.04 | 0.79 | 0.46 | 0.98 | 1.09 | 0.81 | 0.64 | 1.04 | 0.99 | 0.92 |
| 0.56 | 0.87 | 0.98 | 1.12 | 0.92 | 0.89 | 0.93 | 14.55 | 0.95 | 5.54 | 0.91 |
| 4.2 | 1.12 | 1.07 | 0.87 | 0.97 | 0.89 | 0.96 | 1.02 | 1.04 | 0.59 | 0.92 |
| 1.22 | 1.07 | 0.67 | 0.91 | 1.52 | 0.79 | 1.02 | 2.76 | 1.08 | 0.86 | 1.06 |
| 1.04 | 1.2 | 0.83 | 1.31 | 0.86 | 1.19 | 0.87 | 1.85 | 1.12 | 1.14 | 1.19 |
| 1.03 | 0.88 | 0.92 | 1 | 0.97 | 0.96 | 1.05 | 1.19 | 0.93 | 0.83 | 0.78 |
| 0.71 | 0.85 | 1.37 | 0.75 | 1.24 | 1.35 | 0.55 | 0.7 | 1.12 | 0.58 | 1.21 |
| 1.36 | 1.08 | 1.19 | 0.41 | 0.99 | 1 | 0.92 | 1.52 | 1.49 | 1.23 | 1.12 |
| 1.02 | 0.94 | 1.35 | 0.91 | 1.01 | 1.05 | 1.27 | 3.12 | 0.52 | 0.95 | 1 |
| 0.99 | 1.2 | 5.83 | 2.06 | 1.33 | 1.21 | 1.07 | 269.44 | 1.27 | 0.62 | 0.57 |
| 1.48 | 0.88 | 1.07 | 1.26 | 1.34 | 0.85 | 1.01 | 16.45 | 1 | 2.85 | 0.96 |
| 1.05 | 0.84 | 0.88 | 0.53 | 0.89 | 0.84 | 1.16 | 0.6 | 1.03 | 0.81 | 3.2 |
| 0.48 | 0.97 | 0.81 | 0.98 | 1.27 | 0.96 | 0.82 | 9.75 | 1.01 | 1.01 | 1.13 |
| 1.35 | 0.71 | 1.97 | 1.44 | 0.27 | 1.5 | 0.68 | 1.8 | 0.7 | 56.44 | 1.07 |
| 3.6 | 1.02 | 0.87 | 0.62 | 0.84 | 0.92 | 1.05 | 2.14 | 1.05 | 0.95 | 0.87 |
| 0.96 | 1.06 | 1.04 | 1.3 | 0.93 | 0.99 | 1.08 | 0.77 | 0.92 | 1.02 | 0.95 |
| 3.33 | 0.87 | 0.99 | 1.47 | 0.93 | 0.99 | 1.01 | 1.31 | 1.09 | 0.87 | 1.12 |
| 0.47 | 0.63 | 0.99 | 2.73 | 0.86 | 1.04 | 0.91 | 0.96 | 0.88 | 0.8 | 0.84 |
| 1.47 | 1.16 | 0.96 | 4.76 | 1.49 | 0.91 | 0.89 | 0.94 | 0.75 | 1.14 | 1.16 |
| 0.89 | 0.93 | 0.79 | 1.21 | 0.88 | 1.01 | 0.92 | 3.3 | 0.82 | 1.01 | 0.84 |
| 3.07 | 1.67 | 10.67 | 2.45 | 0.75 | 0.75 | 0.77 | 3.6 | 0.96 | 0.49 | 1 |

| | | | | | | | | | | |
|---|---|---|---|---|---|---|---|---|---|---|
| 0.92 | 1.02 | 0.46 | 1.5 | 0.74 | 0.97 | 0.86 | 0.29 | 1.06 | 0.65 | 1.18 |
| 1.61 | 1.35 | 1.35 | 1.57 | 1.34 | 1.23 | 1.1 | 0.78 | 0.74 | 0.49 | 0.94 |
| 1.24 | 0.78 | 0.62 | 2.09 | 0.78 | 0.84 | 0.62 | 0.37 | 0.94 | 1.75 | 0.83 |
| 0.44 | 0.91 | 0.54 | 0.45 | 0.87 | 1.38 | 1.43 | 0.45 | 0.6 | 1.08 | 0.62 |
| 1.26 | 0.73 | 0.98 | 1.06 | 0.93 | 1.33 | 1.35 | 1.07 | 1.06 | 1.2 | 0.93 |
| 0.84 | 1.03 | 0.85 | 0.83 | 0.95 | 1.04 | 0.99 | 2.82 | 1.17 | 2.31 | 0.94 |
| 1.54 | 0.99 | 0.45 | 1.04 | 0.8 | 1.09 | 0.95 | 3.3 | 1.09 | 1.88 | 0.99 |
| 1.99 | 1.37 | 0.77 | 0.32 | 0.78 | 0.94 | 1.28 | 1.2 | 0.92 | 0.39 | 0.8 |
| 0.88 | 0.82 | 0.86 | 1.05 | 0.83 | 1.11 | 1.31 | 0.57 | 1.08 | 1.11 | 3.39 |
| 0.41 | 1.13 | 0.69 | 0.81 | 0.41 | 0.61 | 0.66 | 1.64 | 1.46 | 1.16 | 0.94 |
| 0.78 | 1.23 | 0.93 | 0.73 | 1.1 | 0.81 | 0.91 | 1.53 | 0.82 | 1.07 | 0.77 |
| 3.7 | 1.1 | 1.03 | 0.7 | 0.89 | 0.93 | 0.92 | 0.41 | 1.06 | 0.93 | 0.83 |
| 0.69 | 0.71 | 0.71 | 1.39 | 1.17 | 0.77 | 0.81 | 1.75 | 0.69 | 0.95 | 0.76 |
| 1.53 | 0.94 | 0.81 | 1.28 | 0.75 | 1.02 | 1.04 | 2.43 | 0.97 | 5.92 | 0.96 |
| 0.97 | 1.19 | 0.46 | 1.01 | 0.81 | 0.53 | 0.72 | 1.84 | 0.92 | 2.24 | 0.91 |
| 1.73 | 0.78 | 0.87 | 1.05 | 0.44 | 0.95 | 0.77 | 0.4 | 1.15 | 0.72 | 0.67 |
| 1.52 | 0.99 | 0.79 | 0.88 | 0.98 | 1.22 | 1.06 | 0.56 | 0.83 | 0.92 | 0.74 |
| 1.59 | 0.95 | 0.67 | 3.59 | 0.68 | 0.91 | 1.11 | 1.34 | 1.08 | 4.78 | 0.84 |
| 1.91 | 1.5 | 0.48 | 0.95 | 42.28 | 0.49 | 1.06 | 5.94 | 0.88 | 2.86 | 2.21 |
| 0.91 | 1.46 | 0.42 | 1.73 | 0.35 | 0.6 | 0.91 | 0.14 | 1.12 | 23.97 | 0.76 |
| 1.67 | 0.68 | 1.57 | 1.2 | 1.71 | 0.83 | 1.1 | 1.28 | 0.78 | 1.32 | 1.33 |
| 1.86 | 0.99 | 0.76 | 1.02 | 1.49 | 0.94 | 1.59 | 0.24 | 0.96 | 0.91 | 0.68 |
| 5.75 | 1.79 | 11.22 | 1.14 | 1.32 | 2.06 | 2 | 0.47 | 0.64 | 0.15 | 0.93 |
| 1.23 | 1.29 | 1.18 | 1.1 | 1.93 | 1.09 | 1.04 | 0.61 | 0.73 | 0.77 | 0.84 |
| 0.77 | 1.05 | 0.67 | 0.85 | 0.8 | 0.86 | 0.86 | 1.26 | 1.05 | 0.78 | 1.24 |
| 0.78 | 0.9 | 0.68 | 1.88 | 0.72 | 0.88 | 0.84 | 0.5 | 0.72 | 0.91 | 1.33 |
| 1.4 | 1.05 | 1.23 | 0.96 | 1.1 | 0.91 | 1.12 | 1.33 | 1.04 | 0.66 | 0.82 |
| 0.6 | 1.19 | 0.6 | 1.04 | 0.68 | 1.4 | 0.95 | 3.27 | 1.11 | 1.71 | 1.14 |
| 1.4 | 0.76 | 1.54 | 8.89 | 0.72 | 0.73 | 1.01 | 2.59 | 0.63 | 2.29 | 0.75 |
| 0.42 | 1.35 | 2.9 | 2.67 | 0.27 | 1.32 | 0.48 | 0.35 | 0.73 | 0.4 | 1.99 |
| 2.25 | 0.95 | 1.41 | 0.6 | 0.64 | 1.09 | 0.73 | 1.5 | 1.1 | 0.29 | 1.27 |
| 1.08 | 0.92 | 0.44 | 0.93 | 0.11 | 1.18 | 0.45 | 1.38 | 1.36 | 1.03 | 0.94 |
| 0.88 | 1.16 | 1.41 | 1.51 | 1.44 | 1.07 | 2.46 | 1.11 | 1.07 | 0.21 | 1.4 |

| | | | | | | | | | | |
|---|---|---|---|---|---|---|---|---|---|---|
| 0.81 | 0.92 | 0.65 | 1.21 | 0.86 | 1.07 | 1.05 | 0.46 | 0.83 | 0.84 | 1.07 |
| 1.01 | 1.05 | 1.27 | 0.88 | 1.17 | 1.02 | 0.97 | 0.56 | 0.98 | 1.22 | 1.06 |
| 1.68 | 0.83 | 3.26 | 1.11 | 1.02 | 1.03 | 1.52 | 0.29 | 9 | 3.21 | 2.45 |
| 1.11 | 0.6 | 0.62 | 0.67 | 0.45 | 1.3 | 1.2 | 0.67 | 0.82 | 0.99 | 0.59 |
| 2.43 | 1.22 | 1.25 | 0.62 | 0.82 | 0.83 | 0.83 | 4.27 | 1.01 | 1.18 | 0.73 |
| 0.97 | 0.97 | 2.41 | 1.36 | 1.32 | 1 | 1.28 | 0.63 | 1.05 | 0.28 | 1.28 |
| 1.46 | 0.99 | 1.13 | 1.63 | 0.89 | 1.32 | 1 | 3.29 | 0.99 | 2.48 | 1.25 |
| 0.65 | 1.17 | 3.29 | 0.97 | 1.09 | 1.14 | 1.18 | 2.24 | 0.94 | 1.01 | 1.07 |
| 0.75 | 1.2 | 1.26 | 1.36 | 0.78 | 0.88 | 0.86 | 0.84 | 1.18 | 0.6 | 1.24 |
| 2.34 | 0.98 | 0.83 | 0.95 | 0.57 | 0.95 | 0.82 | 6.69 | 1.13 | 0.18 | 1.08 |
| 1.47 | 0.94 | 0.6 | 1.05 | 0.87 | 0.87 | 1.04 | 2.24 | 0.98 | 1.04 | 1.25 |
| 0.06 | 11.77 | 2.33 | 0.06 | 14.57 | 5.25 | 0.33 | 0.5 | 48.59 | 1.28 | 1.69 |
| 1.56 | 1.01 | 1.72 | 1.02 | 0.89 | 1 | 1.04 | 1.84 | 1.09 | 1.8 | 1.35 |
| 0.8 | 0.65 | 2.28 | 0.58 | 0.76 | 1.8 | 1.12 | 1.11 | 1.04 | 0.82 | 1.16 |

| 202123_s_at | 210762_s_at | 207168_s_at | 204178_s_at | 202431_s_at | 209341_s_at | 203617_x_at | 201432_at | 210825_s_at | 204379_s_at | 205207_at |
|---|---|---|---|---|---|---|---|---|---|---|
| 0.99 | 9.17 | 0.97 | 1.08 | 1.04 | 1.02 | 0.97 | 1.13 | 0.93 | 1.3 | 0.3 |
| 0.95 | 0.75 | 0.92 | 1.26 | 0.41 | 1.16 | 1.01 | 1.11 | 1.17 | 2.74 | 1.16 |
| 0.98 | 4.16 | 0.97 | 0.86 | 1.13 | 1.85 | 1.01 | 1.05 | 0.79 | 0.67 | 1.38 |
| 1.14 | 0.93 | 0.93 | 1.59 | 1.17 | 1.14 | 1.1 | 1.16 | 0.87 | 0.87 | 1.43 |
| 0.99 | 1.02 | 1.02 | 1 | 1.22 | 1.51 | 0.99 | 0.81 | 0.92 | 0.54 | 9.4 |
| 1.01 | 3.79 | 0.97 | 1.82 | 735.49 | 0.71 | 1.46 | 1.81 | 1.03 | 1.21 | 0.93 |
| 1.43 | 3.68 | 0.96 | 1.17 | 0.85 | 1.34 | 1.23 | 1.05 | 1.24 | 0.13 | 8.21 |
| 1.18 | 0.88 | 0.94 | 1.33 | 1.08 | 0.95 | 1.1 | 1.03 | 1.09 | 1.06 | 1.03 |
| 0.79 | 0.88 | 1.04 | 0.75 | 0.82 | 1 | 0.92 | 0.81 | 0.93 | 1.75 | 1.73 |
| 1 | 1.93 | 0.85 | 1.27 | 1.95 | 0.75 | 0.9 | 0.55 | 0.92 | 3.79 | 68.55 |
| 1.47 | 1.25 | 1.02 | 0.86 | 0.89 | 1.27 | 0.79 | 1.04 | 1 | 1.07 | 0.32 |
| 0.95 | 0.98 | 1.31 | 1.41 | 0.97 | 1.1 | 0.94 | 1.3 | 1.38 | 0.56 | 0.42 |
| 0.79 | 0.97 | 1.13 | 1.13 | 1.03 | 1.16 | 0.98 | 1.21 | 1.1 | 1.72 | 0.87 |
| 0.37 | 9.96 | 0.15 | 3.52 | 0.15 | 0.26 | 1.2 | 0.16 | 0.02 | 0.14 | 22.95 |
| 1.41 | 1.6 | 1.1 | 1.23 | 1.71 | 1.47 | 1.5 | 1.54 | 0.83 | 0.81 | 0.38 |
| 1.2 | 0.85 | 1.01 | 1.01 | 0.92 | 1.21 | 0.83 | 1.22 | 0.99 | 1.46 | 0.42 |
| 0.95 | 1.16 | 1.03 | 1.1 | 0.83 | 1.25 | 1.02 | 1.11 | 1.16 | 2.17 | 0.49 |
| 1.13 | 0.56 | 0.98 | 1.01 | 0.74 | 1.05 | 1 | 0.83 | 0.77 | 2.93 | 1.22 |
| 0.99 | 1.57 | 1.31 | 0.97 | 0.22 | 1.32 | 1.2 | 1 | 1.04 | 6.49 | 2.3 |
| 1.08 | 3.4 | 0.62 | 0.82 | 0.74 | 1.39 | 0.72 | 0.78 | 0.56 | 1.12 | 0.62 |
| 0.71 | 1.25 | 0.74 | 1.83 | 0.49 | 0.49 | 1.08 | 1.12 | 1.6 | 1.58 | 0.56 |
| 1.43 | 1.5 | 0.86 | 1.02 | 0.88 | 0.93 | 1.51 | 1.01 | 1.19 | 0.62 | 27.02 |
| 0.9 | 0.73 | 0.95 | 0.89 | 1.41 | 1.07 | 0.91 | 1.43 | 1.03 | 0.38 | 1 |
| 1 | 3.88 | 0.71 | 1.21 | 0.75 | 1.21 | 1.2 | 1.26 | 1.09 | 0.01 | 1.45 |
| 0.41 | 3.04 | 0.79 | 0.82 | 6.79 | 1.79 | 0.83 | 1.56 | 0.78 | 0.03 | 10.3 |
| 1.05 | 2.58 | 1.08 | 1.26 | 0.76 | 0.85 | 1 | 0.76 | 1.08 | 1.58 | 3.47 |
| 1.01 | 2.45 | 1.05 | 0.77 | 0.76 | 1.14 | 0.9 | 1.58 | 0.76 | 0.63 | 0.5 |
| 2.5 | 1.37 | 0.62 | 0.85 | 1.03 | 0.97 | 2.21 | 1.34 | 0.66 | 0.58 | 16.43 |
| 1.65 | 0.15 | 0.71 | 1.04 | 3.43 | 0.5 | 1.97 | 3.64 | 1.62 | 0.04 | 0.39 |
| 1 | 1.45 | 0.82 | 1.01 | 3.16 | 0.83 | 1.02 | 0.83 | 1.04 | 2.25 | 1.78 |

| | | | | | | | | | | |
|---|---|---|---|---|---|---|---|---|---|---|
| 0.84 | 0.89 | 0.78 | 1.21 | 1.37 | 0.69 | 1.22 | 0.47 | 0.89 | 0.8 | 8.62 |
| 2.9 | 2.3 | 3.49 | 6 | 4.73 | 3.19 | 2.78 | 11.35 | 2.9 | 1.88 | 9.55 |
| 1.04 | 1.04 | 1.08 | 1.01 | 0.71 | 1.26 | 0.98 | 1 | 0.91 | 1.03 | 0.83 |
| 0.84 | 1.06 | 1.52 | 1.02 | 0.55 | 0.76 | 1.1 | 0.74 | 1.17 | 0.12 | 1.72 |
| 1.18 | 0.73 | 1.38 | 0.13 | 1.69 | 2.42 | 1.17 | 0.32 | 0.16 | 0.55 | 2.46 |
| 2.32 | 0.61 | 0.87 | 0.71 | 1.53 | 0.78 | 1.2 | 0.54 | 0.93 | 29.75 | 4.34 |
| 0.49 | 0.22 | 0.89 | 1 | 0.97 | 0.93 | 0.9 | 0.43 | 1.44 | 3.89 | 0.1 |
| 0.91 | 0.87 | 1.18 | 0.72 | 1.17 | 0.78 | 0.8 | 0.93 | 1.01 | 1.01 | 1.17 |
| 0.74 | 7.28 | 1.06 | 0.76 | 0.55 | 0.89 | 0.95 | 1.89 | 0.87 | 1.38 | 0.94 |
| 1.84 | 0.28 | 0.65 | 0.53 | 0.48 | 1.88 | 0.93 | 1.8 | 1.53 | 1.38 | 1.77 |
| 0.88 | 1.25 | 0.92 | 1.25 | 0.71 | 1.12 | 1.26 | 0.8 | 1.05 | 1.1 | 0.2 |
| 1.06 | 0.99 | 1.15 | 0.91 | 0.47 | 0.95 | 0.99 | 1.04 | 1.1 | 2.37 | 0.77 |
| 0.91 | 0.77 | 0.99 | 0.44 | 0.44 | 1.41 | 1.03 | 0.81 | 0.84 | 0.26 | 3.01 |
| 0.77 | 0.73 | 0.88 | 0.64 | 2.84 | 1.49 | 0.78 | 1.23 | 0.47 | 0.82 | 0.67 |
| 0.87 | 0.76 | 1.02 | 0.55 | 0.6 | 1.22 | 0.83 | 0.93 | 0.92 | 0.7 | 2.59 |
| 1.04 | 2.77 | 0.91 | 1.02 | 1.23 | 1.59 | 1.14 | 1.3 | 0.93 | 5.33 | 10.83 |
| 0.96 | 1.04 | 1.06 | 1.05 | 0.86 | 1.14 | 0.92 | 1.05 | 1.1 | 2.08 | 0.21 |
| 0.86 | 1.09 | 1.05 | 0.95 | 1.38 | 1.1 | 1.11 | 0.8 | 0.91 | 0.87 | 2.13 |
| 0.99 | 1.01 | 1.05 | 0.92 | 0.9 | 1.06 | 1.21 | 0.96 | 0.94 | 1.02 | 1.16 |
| 1.3 | 1.43 | 0.77 | 1.39 | 6.01 | 0.89 | 0.92 | 0.72 | 1.44 | 5.28 | 4.22 |
| 1.12 | 0.56 | 1.11 | 0.96 | 0.74 | 0.81 | 0.89 | 1.34 | 1.16 | 2.19 | 0.25 |
| 0.99 | 1.04 | 1.19 | 0.96 | 5.45 | 1.06 | 0.81 | 1.01 | 1.13 | 0.86 | 1.29 |
| 0.76 | 0.48 | 0.83 | 0.94 | 1.2 | 0.7 | 1.01 | 1.06 | 1.03 | 0.67 | 0.85 |
| 1.17 | 5.46 | 1.76 | 1.18 | 0.64 | 1.13 | 1.38 | 1.73 | 0.94 | 1.74 | 3.13 |
| 0.9 | 0.42 | 1.1 | 1.17 | 1.96 | 0.66 | 1.07 | 0.77 | 0.87 | 0.59 | 4.27 |
| 1.12 | 0.64 | 1.28 | 1.66 | 0.62 | 1.16 | 2.71 | 0.92 | 1.14 | 2.56 | 0.87 |
| 0.67 | 0.57 | 1.38 | 2.1 | 0.59 | 1.15 | 1.16 | 0.63 | 0.74 | 4.9 | 62.08 |
| 2.34 | 1.44 | 1.19 | 0.61 | 0.5 | 2.16 | 1.04 | 0.75 | 1.17 | 0.66 | 0.85 |
| 1 | 1.21 | 0.95 | 0.79 | 0.82 | 1.43 | 1.04 | 1.27 | 0.9 | 1.62 | 0.89 |
| 0.8 | 0.91 | 0.97 | 0.47 | 0.46 | 1.22 | 0.88 | 0.78 | 0.94 | 0.49 | 5.01 |
| 0.78 | 4.7 | 2.74 | 0.92 | 0.67 | 0.92 | 1.17 | 2.85 | 0.89 | 3.73 | 1.33 |
| 1.24 | 0.76 | 0.94 | 0.85 | 1.04 | 1.61 | 0.91 | 0.93 | 0.5 | 0.07 | 0.97 |
| 1.03 | 1.31 | 0.91 | 0.83 | 142.34 | 1.02 | 1.29 | 1.33 | 1.04 | 1.35 | 1.32 |

| | | | | | | | | | | |
|---|---|---|---|---|---|---|---|---|---|---|
| 1.12 | 0.78 | 1.11 | 0.9 | 0.91 | 1.04 | 0.78 | 1.66 | 0.98 | 1.75 | 1.06 |
| 0.9 | 1.55 | 0.86 | 1.2 | 1.85 | 0.99 | 1.52 | 0.81 | 0.96 | 10.91 | 4.79 |
| 0.93 | 4.62 | 0.98 | 1.03 | 1 | 1 | 1.03 | 1.03 | 1.07 | 5.5 | 2.39 |
| 0.98 | 7.92 | 1 | 1.03 | 1.37 | 0.88 | 0.97 | 1.1 | 1.14 | 4.15 | 1.45 |
| 1.04 | 1.13 | 0.96 | 0.81 | 1.04 | 1.21 | 1.11 | 0.85 | 1.07 | 1.33 | 1.2 |
| 1.04 | 0.64 | 1.28 | 0.9 | 1.04 | 1.65 | 0.62 | 1.59 | 1.18 | 1 | 2.02 |
| 1.84 | 0.67 | 0.85 | 1.41 | 1.83 | 0.6 | 1.67 | 2.16 | 0.52 | 0.79 | 1.21 |
| 0.96 | 0.7 | 1.05 | 1.56 | 1.1 | 1.26 | 1.5 | 1.69 | 0.95 | 2.14 | 1.15 |
| 1.26 | 1.67 | 0.88 | 1.17 | 1.08 | 6.85 | 1.11 | 0.88 | 2.35 | 3.72 | 0.84 |
| 0.61 | 0.55 | 0.81 | 0.76 | 0.8 | 1.61 | 1.44 | 1.21 | 0.98 | 0.51 | 0.74 |
| 6.73 | 1.98 | 1.04 | 1.44 | 2.8 | 0.5 | 1.51 | 1.79 | 2.26 | 0.68 | 2.45 |
| 0.71 | 2.49 | 1.08 | 1.01 | 0.74 | 1.07 | 0.73 | 0.69 | 1.01 | 0.79 | 3 |
| 1.11 | 1.3 | 0.83 | 0.76 | 1.33 | 1.35 | 1.49 | 0.97 | 1.19 | 0.66 | 2.96 |
| 0.63 | 2.14 | 0.81 | 0.73 | 2 | 0.99 | 0.83 | 0.88 | 1.03 | 0.65 | 7.4 |
| 1.04 | 2.93 | 1.31 | 1.25 | 1 | 0.87 | 0.95 | 1.01 | 1.3 | 0.97 | 0.47 |
| 1.47 | 0.22 | 12.5 | 0.74 | 52.03 | 9.99 | 1.24 | 147.17 | 20.36 | 2.44 | 0.27 |
| 0.99 | 0.4 | 1.07 | 0.77 | 0.53 | 1.44 | 1.02 | 1.41 | 0.7 | 4.44 | 1.41 |
| 0.91 | 0.35 | 1.95 | 0.73 | 0.13 | 1.29 | 1.2 | 0.82 | 2.63 | 7.33 | 0.26 |
| 1.11 | 0.98 | 0.93 | 0.95 | 1.22 | 1.21 | 0.92 | 0.91 | 0.87 | 0.78 | 0.91 |
| 0.9 | 0.9 | 0.99 | 1.22 | 1.02 | 1.04 | 2 | 0.85 | 0.88 | 1.77 | 0.87 |
| 1.27 | 1.12 | 0.95 | 0.9 | 0.33 | 1.25 | 1.33 | 0.83 | 1.57 | 1.44 | 7.44 |
| 1.19 | 1.3 | 1.11 | 1.15 | 1.17 | 1.2 | 1.02 | 1.28 | 0.94 | 11.68 | 2.16 |
| 1.13 | 2.13 | 1.01 | 1.12 | 0.87 | 0.93 | 1.28 | 0.74 | 1.04 | 0.13 | 5.29 |
| 1.91 | 0.42 | 0.92 | 3.02 | 0.51 | 0.8 | 0.73 | 0.83 | 0.93 | 22.71 | 0.6 |
| 0.92 | 0.82 | 1.02 | 0.86 | 1.04 | 1.17 | 0.97 | 1.5 | 0.99 | 0.26 | 0.64 |
| 0.93 | 2.07 | 0.97 | 1.02 | 1.07 | 0.9 | 0.67 | 1.22 | 0.89 | 0.87 | 0.9 |
| 1.11 | 1 | 1.02 | 1.28 | 1.28 | 0.99 | 0.99 | 0.92 | 1.07 | 1.26 | 0.8 |
| 1.14 | 3.61 | 0.99 | 1 | 1.31 | 1 | 1.24 | 0.95 | 1.18 | 3.83 | 3.89 |
| 0.86 | 2.14 | 0.95 | 1.07 | 1.07 | 0.79 | 1.3 | 1.05 | 1.08 | 1.28 | 2.03 |
| 1.15 | 0.92 | 1.02 | 1.22 | 1.03 | 0.89 | 1.18 | 0.96 | 0.92 | 1.2 | 1.09 |
| 0.94 | 0.97 | 1 | 1.12 | 0.83 | 1.07 | 0.84 | 0.88 | 0.94 | 1.34 | 0.84 |
| 0.8 | 0.64 | 0.77 | 0.76 | 0.62 | 0.73 | 0.87 | 0.74 | 1.14 | 3.78 | 0.36 |
| 1.02 | 7.25 | 0.81 | 0.9 | 0.77 | 1.39 | 1.09 | 0.91 | 0.8 | 0.98 | 7.33 |

| | | | | | | | | | | |
|---|---|---|---|---|---|---|---|---|---|---|
| 1.18 | 1.34 | 0.99 | 1.08 | 2.26 | 1.31 | 1.05 | 0.69 | 1.06 | 1.09 | 1.3 |
| 0.99 | 0.31 | 1.07 | 0.95 | 0.92 | 0.83 | 1.11 | 1.03 | 1.06 | 1.27 | 2 |
| 1.09 | 1.51 | 0.89 | 1.08 | 1.59 | 0.75 | 1.47 | 1.19 | 0.94 | 1.12 | 3.26 |
| 1.17 | 0.78 | 0.75 | 1.29 | 0.6 | 4.71 | 1.3 | 0.98 | 1.41 | 1.64 | 1.09 |
| 0.84 | 0.97 | 1.55 | 1.21 | 1.17 | 1.72 | 0.71 | 1.17 | 1.08 | 0.61 | 1.78 |
| 0.47 | 1.57 | 0.58 | 0.71 | 0.62 | 0.5 | 0.63 | 0.48 | 0.78 | 1.2 | 1.98 |
| 1.21 | 1.26 | 0.83 | 0.78 | 1.52 | 1.47 | 0.87 | 0.9 | 0.76 | 0.78 | 5.14 |
| 1.02 | 1.43 | 1.13 | 0.82 | 0.71 | 0.94 | 1.04 | 1.32 | 1.76 | 1.74 | 0.76 |
| 0.95 | 0.84 | 0.68 | 1.03 | 1.65 | 0.57 | 0.93 | 1.23 | 0.64 | 0.94 | 1.52 |
| 1.18 | 1.86 | 1.08 | 0.91 | 1.39 | 1.01 | 1.11 | 0.97 | 0.95 | 0.74 | 1.95 |
| 0.86 | 0.75 | 1.43 | 1.03 | 0.65 | 0.91 | 1.09 | 1.02 | 0.94 | 1.01 | 1.19 |
| 0.93 | 0.97 | 1.02 | 1.22 | 1.26 | 1.14 | 1.2 | 1.17 | 1.02 | 0.82 | 0.8 |
| 0.97 | 0.96 | 1.08 | 1.68 | 1.03 | 0.73 | 0.9 | 0.88 | 1.04 | 1.36 | 8.24 |
| 1.12 | 1.15 | 1.15 | 1.17 | 0.84 | 0.87 | 1.36 | 0.8 | 1.28 | 0.31 | 1.09 |
| 1.09 | 0.69 | 1 | 0.83 | 2.56 | 1.26 | 0.98 | 0.98 | 0.99 | 0.88 | 1.35 |
| 1.29 | 1.33 | 1.06 | 0.98 | 1.01 | 1.24 | 0.97 | 0.84 | 1.09 | 2.95 | 0.99 |
| 0.75 | 0.39 | 0.94 | 1.06 | 1.32 | 0.92 | 0.9 | 1.08 | 1 | 2.77 | 1.8 |
| 1.15 | 8.55 | 1.02 | 1.2 | 1.75 | 1.19 | 0.97 | 1.05 | 1.06 | 3.43 | 2.44 |
| 0.9 | 1.4 | 0.9 | 0.95 | 0.97 | 1.2 | 0.94 | 1.02 | 1.1 | 1.47 | 1.68 |
| 1.1 | 4.53 | 1.23 | 0.98 | 0.81 | 0.7 | 1 | 0.92 | 0.89 | 1.82 | 1.75 |
| 0.82 | 1.14 | 0.99 | 1.59 | 0.85 | 0.98 | 1.09 | 0.78 | 0.77 | 0.66 | 1.15 |
| 1.09 | 0.36 | 0.91 | 0.92 | 1.09 | 0.71 | 1.04 | 0.95 | 0.96 | 0.9 | 2.54 |
| 1.03 | 1.01 | 0.93 | 1.05 | 1.21 | 1.1 | 1 | 1.07 | 1.14 | 2.61 | 0.91 |
| 0.94 | 1 | 0.95 | 0.86 | 1.73 | 1.86 | 0.76 | 1.23 | 0.94 | 1.22 | 1.15 |
| 1.35 | 0.77 | 1.03 | 0.98 | 0.87 | 0.88 | 0.95 | 1.13 | 1.36 | 0.7 | 0.58 |
| 0.75 | 1.54 | 0.84 | 0.78 | 1.76 | 1.08 | 0.92 | 1 | 0.96 | 1.16 | 1.86 |
| 1.02 | 0.76 | 1.12 | 0.95 | 1.23 | 1.29 | 1.18 | 0.73 | 0.93 | 1.02 | 1 |
| 1.28 | 1.36 | 0.87 | 0.61 | 2.06 | 1.33 | 0.76 | 1.13 | 0.82 | 0.94 | 0.83 |
| 0.79 | 2.68 | 1.18 | 1.39 | 1.32 | 0.79 | 0.98 | 1.11 | 0.77 | 0.23 | 0.88 |
| 0.9 | 1.02 | 0.96 | 1.19 | 0.9 | 0.82 | 1.22 | 0.91 | 1.01 | 0.75 | 10.34 |
| 1.08 | 0.26 | 1.02 | 0.93 | 1.04 | 0.84 | 0.9 | 0.98 | 1.08 | 1.24 | 1.23 |
| 0.9 | 1.55 | 0.86 | 1.2 | 1.85 | 0.99 | 1.52 | 0.81 | 0.96 | 10.91 | 4.79 |
| 1.13 | 0.81 | 1 | 1.01 | 1.59 | 1.09 | 0.89 | 0.81 | 0.95 | 0.75 | 8.18 |

| | | | | | | | | | | |
|---|---|---|---|---|---|---|---|---|---|---|
| 1.15 | 0.24 | 1.11 | 1.02 | 1.3 | 0.89 | 1.22 | 1.47 | 0.97 | 1.15 | 0.39 |
| 1.08 | 1.57 | 0.97 | 1.04 | 1.04 | 0.82 | 0.86 | 1.05 | 0.81 | 0.85 | 2.72 |
| 1.28 | 1.08 | 1.2 | 0.74 | 1.12 | 1.09 | 2.05 | 0.91 | 0.81 | 1.15 | 0.52 |
| 1.06 | 1.21 | 0.98 | 0.95 | 1.08 | 0.91 | 0.92 | 1.02 | 1 | 0.88 | 6.17 |
| 0.93 | 0.81 | 1.06 | 0.78 | 0.97 | 0.98 | 1.08 | 1.1 | 0.91 | 3.72 | 0.45 |
| 0.95 | 0.82 | 0.99 | 1.12 | 1.1 | 0.9 | 0.8 | 1.03 | 0.99 | 1.13 | 0.96 |
| 0.82 | 0.18 | 1.02 | 1 | 1.19 | 1 | 1.51 | 0.9 | 0.95 | 0.95 | 0.71 |
| 1.09 | 0.97 | 1.02 | 0.81 | 0.84 | 0.92 | 1.11 | 0.83 | 0.97 | 0.84 | 1.07 |
| 1.17 | 3.66 | 1.17 | 0.99 | 0.65 | 1.23 | 1.31 | 0.56 | 2.19 | 0.09 | 4.09 |
| 0.95 | 19.75 | 0.86 | 1.05 | 0.84 | 1.06 | 1.58 | 1.05 | 1.06 | 7.02 | 2.01 |
| 0.76 | 0.87 | 1.13 | 1.01 | 1.09 | 1.14 | 1.43 | 0.92 | 1.1 | 0.41 | 1.33 |
| 0.97 | 0.77 | 1.09 | 0.99 | 1.15 | 1.06 | 0.83 | 0.95 | 0.9 | 2.22 | 3.13 |
| 1.3 | 7.45 | 1.03 | 1.21 | 1.6 | 1.07 | 0.98 | 1.34 | 1.08 | 2.78 | 1.97 |
| 0.97 | 0.83 | 0.9 | 0.62 | 0.93 | 1.09 | 1.08 | 0.92 | 0.91 | 1.01 | 0.47 |
| 0.86 | 2.09 | 0.8 | 0.81 | 0.41 | 1.38 | 0.88 | 0.58 | 1.53 | 0.66 | 14.38 |
| 0.97 | 0.76 | 0.94 | 1.13 | 0.87 | 1.1 | 0.94 | 1.23 | 1 | 3.11 | 0.63 |
| 0.91 | 0.97 | 1.02 | 0.94 | 0.92 | 0.91 | 0.84 | 0.96 | 1.21 | 0.72 | 1.29 |
| 1.38 | 5.44 | 1.22 | 1.82 | 2.9 | 0.9 | 1.8 | 0.73 | 1.21 | 1.09 | 2.67 |
| 0.93 | 1.17 | 1.03 | 1.04 | 1.03 | 0.96 | 0.93 | 1.02 | 1.17 | 0.56 | 1.04 |
| 1.08 | 1.16 | 0.98 | 0.91 | 0.77 | 0.84 | 1.07 | 0.45 | 1.13 | 0.94 | 0.26 |
| 0.73 | 0.85 | 1.01 | 0.78 | 0.49 | 1.04 | 1.05 | 1.27 | 0.68 | 0.96 | 1.63 |
| 0.75 | 3.09 | 1.17 | 1.16 | 0.46 | 1.21 | 0.91 | 0.5 | 1.09 | 0.57 | 3.06 |
| 1.01 | 1.42 | 1.06 | 0.91 | 0.85 | 1.06 | 1.13 | 1.24 | 1.1 | 0.82 | 1.31 |
| 0.87 | 1.13 | 0.81 | 0.63 | 0.68 | 0.94 | 0.87 | 1.36 | 1.06 | 1.81 | 0.6 |
| 0.73 | 1.31 | 0.98 | 1.19 | 0.94 | 1.38 | 1.36 | 0.88 | 1.09 | 3.31 | 1.27 |
| 1 | 0.97 | 1.04 | 1.06 | 0.89 | 1.15 | 1.15 | 0.74 | 1.07 | 1.12 | 0.32 |
| 1.12 | 8.54 | 1 | 1.15 | 0.88 | 1.29 | 0.72 | 1.16 | 1.47 | 0.87 | 1.25 |
| 0.86 | 3.08 | 0.88 | 1.25 | 1.4 | 0.82 | 0.93 | 0.9 | 1.03 | 1.16 | 1.68 |
| 0.97 | 0.86 | 1 | 0.83 | 0.67 | 1.07 | 1.04 | 1.01 | 0.89 | 1.06 | 1.15 |
| 0.98 | 0.56 | 1.07 | 0.81 | 0.93 | 1.19 | 1.32 | 1.08 | 0.94 | 1.13 | 1.84 |
| 1.07 | 2.12 | 1.11 | 1.19 | 0.72 | 0.81 | 0.8 | 0.88 | 1.04 | 0.89 | 1.31 |
| 0.93 | 1.86 | 1 | 1.07 | 1.01 | 1.01 | 0.97 | 0.96 | 0.99 | 1.8 | 2.97 |
| 0.98 | 0.75 | 1.01 | 1.07 | 0.94 | 1.1 | 0.96 | 1.06 | 1.17 | 1.33 | 0.78 |

| | | | | | | | | | | |
|---|---|---|---|---|---|---|---|---|---|---|
| 1.49 | 1.46 | 0.97 | 1.58 | 0.81 | 6.69 | 1.09 | 1.12 | 2.47 | 5.19 | 0.71 |
| 1.07 | 1.88 | 1.04 | 1.31 | 0.89 | 0.82 | 0.98 | 0.91 | 1.16 | 1.38 | 1.25 |
| 0.99 | 0.99 | 0.96 | 1.09 | 0.96 | 1.01 | 1.01 | 0.98 | 1.15 | 0.57 | 0.67 |
| 0.52 | 1.1 | 1.04 | 0.74 | 0.77 | 1.19 | 0.85 | 1.25 | 0.94 | 0.9 | 0.94 |
| 1.07 | 1.66 | 1.23 | 2.1 | 0.68 | 0.51 | 0.98 | 0.85 | 1.31 | 1.53 | 1.79 |
| 0.97 | 1.21 | 0.96 | 1.29 | 0.92 | 0.99 | 1 | 0.97 | 0.87 | 0.99 | 1.45 |
| 0.83 | 1.08 | 0.76 | 1.17 | 1.4 | 0.7 | 1.39 | 0.43 | 0.8 | 1.07 | 10.29 |
| 0.92 | 1.1 | 1.03 | 0.96 | 2.13 | 0.93 | 0.84 | 1.08 | 1.03 | 1.2 | 1.08 |
| 1.06 | 0.93 | 1.06 | 1.13 | 0.71 | 0.94 | 1.29 | 0.93 | 0.98 | 1.26 | 0.65 |
| 0.93 | 1.02 | 0.76 | 1.22 | 1.63 | 0.68 | 0.99 | 0.49 | 0.86 | 2.16 | 6.44 |
| 1.21 | 0.93 | 0.96 | 0.78 | 0.9 | 0.73 | 0.85 | 1.06 | 0.87 | 1.62 | 1.73 |
| 1.11 | 0.62 | 0.93 | 1.11 | 0.93 | 0.83 | 1.08 | 0.81 | 1.59 | 0.9 | 1.13 |
| 0.77 | 1.62 | 0.9 | 0.93 | 0.98 | 0.92 | 0.73 | 0.84 | 0.78 | 0.7 | 1.38 |
| 1.02 | 0.99 | 1.06 | 0.98 | 0.92 | 0.91 | 0.83 | 0.99 | 1 | 0.77 | 1.65 |
| 0.97 | 0.7 | 1.05 | 1 | 0.97 | 0.92 | 1.12 | 0.94 | 1.06 | 0.93 | 2.89 |
| 1.06 | 1.37 | 0.87 | 1.14 | 1.52 | 1.06 | 0.92 | 1.46 | 1 | 1.09 | 1.23 |
| 1.06 | 1.17 | 0.99 | 1.44 | 0.86 | 1.19 | 1.4 | 0.91 | 1.02 | 1.59 | 1.04 |
| 0.94 | 0.9 | 1 | 0.98 | 0.97 | 0.78 | 0.76 | 0.91 | 0.91 | 2.04 | 3.19 |
| 1.29 | 1.07 | 1.24 | 0.7 | 1.24 | 1.21 | 0.97 | 0.76 | 0.61 | 0.91 | 0.34 |
| 0.92 | 1.07 | 0.9 | 0.92 | 0.99 | 1.12 | 0.82 | 1.16 | 1.26 | 1.09 | 0.9 |
| 1.32 | 0.52 | 1.01 | 0.97 | 1.01 | 1 | 1.05 | 1.01 | 1.23 | 0.57 | 1.13 |
| 0.97 | 0.34 | 1.94 | 1.26 | 1.33 | 0.57 | 1.37 | 1.34 | 1.14 | 0.13 | 0.22 |
| 1.03 | 0.59 | 0.95 | 1.17 | 1.34 | 0.96 | 0.85 | 1.08 | 1.06 | 0.97 | 1 |
| 1.34 | 1.21 | 1.01 | 1.09 | 0.89 | 3.2 | 1.18 | 1.07 | 1.28 | 5.22 | 0.88 |
| 1.03 | 0.97 | 0.93 | 0.89 | 1.27 | 1.13 | 1.08 | 0.93 | 1.02 | 0.66 | 1.31 |
| 1.45 | 1.74 | 0.82 | 0.59 | 0.27 | 1.07 | 0.83 | 2.26 | 1.05 | 2.29 | 0.18 |
| 1.27 | 1.08 | 0.99 | 1.23 | 0.84 | 0.87 | 1.39 | 0.94 | 1 | 0.88 | 1.41 |
| 0.98 | 1.96 | 1.04 | 1.01 | 0.93 | 0.95 | 0.88 | 1.09 | 1 | 1.86 | 0.77 |
| 0.92 | 1.18 | 1.01 | 1.13 | 0.93 | 1.12 | 1.01 | 0.94 | 1.02 | 2 | 4.94 |
| 0.87 | 0.5 | 1.06 | 0.96 | 0.86 | 0.84 | 0.53 | 1.15 | 1.05 | 0.93 | 0.88 |
| 0.88 | 0.84 | 1.18 | 1.06 | 1.49 | 1.16 | 1.25 | 1.43 | 0.9 | 0.75 | 0.66 |
| 1.18 | 0.7 | 1.14 | 0.83 | 0.88 | 0.84 | 1.23 | 0.8 | 1.05 | 1.19 | 1.2 |
| 1.03 | 0.92 | 1.08 | 1.02 | 0.75 | 1 | 2.43 | 0.97 | 1.17 | 0.9 | 0.25 |

| | | | | | | | | | | |
|---|---|---|---|---|---|---|---|---|---|---|
| 1.24 | 0.67 | 0.96 | 0.92 | 0.74 | 1.18 | 1.77 | 0.95 | 1.16 | 15.26 | 2.96 |
| 1.11 | 1.1 | 1.05 | 0.91 | 1.34 | 0.94 | 1.1 | 0.97 | 0.96 | 0.66 | 0.34 |
| 1.16 | 6.91 | 1.16 | 1.16 | 0.78 | 0.83 | 0.99 | 0.79 | 0.99 | 0.71 | 1.4 |
| 0.72 | 1.02 | 0.74 | 1.08 | 0.87 | 0.62 | 0.74 | 0.87 | 0.67 | 3.66 | 1.07 |
| 1.34 | 7.53 | 1.11 | 1.63 | 0.93 | 0.93 | 1.44 | 0.75 | 0.78 | 0.87 | 1.08 |
| 0.89 | 2.5 | 1.04 | 0.82 | 0.95 | 0.94 | 0.78 | 1.12 | 1.07 | 1.33 | 2.28 |
| 0.94 | 1.58 | 0.85 | 0.9 | 0.8 | 0.99 | 0.84 | 0.94 | 1.15 | 0.57 | 1.38 |
| 0.91 | 0.3 | 0.84 | 0.94 | 0.78 | 0.8 | 1.01 | 0.96 | 1.18 | 0.61 | 2.08 |
| 1.19 | 1.82 | 0.94 | 1.42 | 0.83 | 3.39 | 1.44 | 0.61 | 1.97 | 3.99 | 0.64 |
| 1.02 | 1.27 | 0.86 | 0.88 | 0.41 | 0.94 | 1.48 | 1.34 | 0.59 | 4.39 | 2 |
| 0.82 | 0.62 | 0.95 | 0.9 | 1.1 | 0.77 | 0.82 | 1.04 | 1.05 | 0.77 | 2.45 |
| 0.55 | 1.03 | 1.03 | 1.09 | 0.89 | 0.83 | 1.06 | 0.92 | 1.11 | 1.16 | 0.45 |
| 0.96 | 0.18 | 1.01 | 0.93 | 1.17 | 0.76 | 0.91 | 0.8 | 0.93 | 0.41 | 1.33 |
| 1.14 | 1.31 | 1.04 | 1.08 | 0.75 | 0.96 | 1.02 | 0.97 | 1.09 | 1.13 | 1.64 |
| 0.9 | 1.4 | 0.92 | 0.76 | 0.81 | 0.91 | 1.01 | 1.43 | 0.87 | 0.77 | 1.28 |
| 1.28 | 1.08 | 1.02 | 1.07 | 0.44 | 0.67 | 0.6 | 0.98 | 0.91 | 4.83 | 0.36 |
| 0.87 | 1.06 | 1.07 | 0.91 | 0.98 | 0.74 | 1.5 | 0.95 | 0.95 | 0.72 | 1 |
| 0.93 | 1.34 | 0.92 | 0.87 | 0.68 | 0.84 | 0.96 | 1 | 1.05 | 1.21 | 2.79 |
| 1.22 | 1.17 | 0.79 | 1.35 | 42.28 | 2.21 | 1.15 | 0.74 | 1.15 | 5.22 | 1.95 |
| 2.17 | 6.91 | 0.74 | 1.21 | 0.35 | 0.76 | 1.15 | 1.15 | 1.87 | 0.24 | 2.45 |
| 1.12 | 1.21 | 0.91 | 0.92 | 1.71 | 1.33 | 1.28 | 1.27 | 0.87 | 0.76 | 1.6 |
| 0.89 | 0.87 | 0.78 | 1.24 | 1.49 | 0.68 | 1.02 | 0.5 | 0.91 | 1.34 | 6.7 |
| 0.91 | 1.1 | 1.63 | 1.28 | 1.32 | 0.93 | 1.15 | 0.64 | 1.08 | 0.08 | 3.01 |
| 1.04 | 2.04 | 0.96 | 0.9 | 1.93 | 0.84 | 1.13 | 0.84 | 0.98 | 1.03 | 1.36 |
| 1.12 | 1.48 | 0.99 | 1.03 | 0.8 | 1.24 | 0.99 | 1.02 | 1.17 | 0.97 | 1.27 |
| 0.94 | 2.54 | 1.1 | 1.05 | 0.72 | 1.33 | 1.19 | 1.02 | 0.85 | 1.49 | 4.44 |
| 0.76 | 1.12 | 0.93 | 1.16 | 1.1 | 0.82 | 1.17 | 0.8 | 1.2 | 0.69 | 0.63 |
| 1.75 | 2.43 | 0.87 | 0.78 | 0.68 | 1.14 | 1.31 | 1.99 | 1.09 | 0.55 | 2.09 |
| 0.79 | 0.68 | 1.13 | 1.6 | 0.72 | 0.75 | 1.24 | 1.05 | 1.27 | 3.21 | 2.11 |
| 0.54 | 0.57 | 1.16 | 0.32 | 0.27 | 1.99 | 0.44 | 0.44 | 1.32 | 14.29 | 5.62 |
| 0.92 | 1.3 | 0.92 | 0.98 | 0.64 | 1.27 | 0.66 | 0.97 | 0.88 | 1.18 | 0.85 |
| 0.92 | 1.32 | 0.99 | 0.81 | 0.11 | 0.94 | 0.95 | 0.77 | 0.78 | 7.74 | 1.19 |
| 1.88 | 2.02 | 0.98 | 2.48 | 1.44 | 1.4 | 2 | 0.73 | 1.57 | 1.82 | 3.68 |

| | | | | | | | | | | |
|---|---|---|---|---|---|---|---|---|---|---|
| 0.83 | 0.78 | 0.96 | 0.76 | 0.86 | 1.07 | 0.98 | 0.96 | 0.93 | 0.79 | 0.68 |
| 0.93 | 1.04 | 1.01 | 0.89 | 1.17 | 1.06 | 1.26 | 1.12 | 0.87 | 1.34 | 1.02 |
| 1.39 | 0.86 | 2.13 | 1.01 | 1.02 | 2.45 | 1.47 | 1.23 | 3.14 | 0.67 | 0.92 |
| 0.97 | 0.31 | 0.93 | 1.19 | 0.45 | 0.59 | 0.84 | 0.55 | 0.75 | 1.74 | 0.13 |
| 0.49 | 1.04 | 0.98 | 0.96 | 0.82 | 0.73 | 0.82 | 0.71 | 0.75 | 1.65 | 0.98 |
| 0.85 | 1.06 | 1.07 | 1.08 | 1.32 | 1.28 | 1.06 | 1 | 1.09 | 1.12 | 1.68 |
| 0.91 | 3.51 | 1.05 | 1.1 | 0.89 | 1.25 | 1.1 | 1 | 0.91 | 1.17 | 1.18 |
| 0.85 | 0.76 | 1.04 | 1.12 | 1.09 | 1.07 | 0.84 | 0.93 | 1.01 | 1.46 | 0.87 |
| 1.2 | 3.35 | 1.01 | 1.05 | 0.78 | 1.24 | 1.58 | 0.74 | 0.93 | 2.02 | 1.56 |
| 1.3 | 1.05 | 1.03 | 0.85 | 0.57 | 1.08 | 1.14 | 1.07 | 0.65 | 2.32 | 0.51 |
| 0.89 | 1.09 | 1 | 0.89 | 0.87 | 1.25 | 1.04 | 0.92 | 1.06 | 2.04 | 1.67 |
| 0.35 | 6.2 | 0.77 | 0.76 | 14.57 | 1.69 | 0.31 | 6.17 | 4.6 | 0.75 | 2.13 |
| 1.14 | 1.38 | 1.07 | 1.21 | 0.89 | 1.35 | 1.12 | 0.79 | 1.45 | 1.43 | 1.53 |
| 1.05 | 2.41 | 1.18 | 0.87 | 0.76 | 1.16 | 1.94 | 0.88 | 1.41 | 1.82 | 0.44 |

| 219041_s_at | 200603_at | 218902_at | 204798_at | 205407_at | 204531_s_at | 209341_s_at | 200608_s_at | 202092_s_at | 202431_s_at | 200670_at |
|---|---|---|---|---|---|---|---|---|---|---|
| 1.18 | 1.01 | 1.27 | 1.93 | 8.89 | 1 | 1.02 | 0.86 | 1.16 | 1.04 | 1.09 |
| 0.65 | 1.24 | 1.02 | 1.54 | 0.98 | 0.98 | 1.16 | 1 | 0.97 | 0.41 | 1.21 |
| 1.16 | 1.78 | 1.77 | 1.06 | 0.47 | 1.79 | 1.85 | 1.57 | 1.04 | 1.13 | 1.07 |
| 1.06 | 0.93 | 1.36 | 1.04 | 9.11 | 1.01 | 1.14 | 0.76 | 0.99 | 1.17 | 1 |
| 0.65 | 1.19 | 1.27 | 1.16 | 0.72 | 1.22 | 1.51 | 1.21 | 1.21 | 1.22 | 1.13 |
| 0.84 | 1 | 0.91 | 0.7 | 2.54 | 1.04 | 0.71 | 1.14 | 1.23 | 735.49 | 1.23 |
| 1.59 | 0.9 | 0.34 | 0.07 | 3.48 | 0.85 | 1.34 | 0.83 | 1.48 | 0.85 | 1.39 |
| 1.01 | 0.97 | 1.05 | 0.9 | 0.6 | 1.02 | 0.95 | 0.88 | 1.16 | 1.08 | 0.88 |
| 0.62 | 0.9 | 0.77 | 0.6 | 1.1 | 1.03 | 1 | 0.86 | 1.06 | 0.82 | 0.95 |
| 0.57 | 0.71 | 1.23 | 0.27 | 0.58 | 0.69 | 0.75 | 1.2 | 1.08 | 1.95 | 1.44 |
| 1.53 | 0.87 | 1.3 | 1.17 | 1.63 | 1.26 | 1.27 | 0.86 | 0.81 | 0.89 | 0.67 |
| 0.87 | 0.99 | 0.9 | 1.25 | 0.98 | 0.9 | 1.1 | 1.03 | 1.22 | 0.97 | 0.9 |
| 0.98 | 1.09 | 0.96 | 0.85 | 0.52 | 0.91 | 1.16 | 1.14 | 0.98 | 1.03 | 1.42 |
| 0.1 | 0.01 | 0.65 | 2.15 | 0.44 | 0.13 | 0.26 | 0.98 | 0.24 | 0.15 | 0.1 |
| 1.05 | 1.38 | 0.72 | 0.85 | 1.48 | 0.66 | 1.47 | 1.1 | 1.98 | 1.71 | 1.48 |
| 0.76 | 1.18 | 0.87 | 1.76 | 1.13 | 0.72 | 1.21 | 0.86 | 0.99 | 0.92 | 1.3 |
| 0.87 | 0.98 | 1.15 | 2.98 | 1.03 | 1.02 | 1.25 | 0.86 | 1.07 | 0.83 | 0.74 |
| 1.23 | 1.16 | 1.52 | 4.28 | 0.93 | 0.83 | 1.05 | 1.03 | 1.17 | 0.74 | 1.13 |
| 0.65 | 1.24 | 6.36 | 0.53 | 1.35 | 0.91 | 1.32 | 1.16 | 1.06 | 0.22 | 0.72 |
| 0.71 | 0.5 | 0.57 | 1.27 | 10.58 | 0.5 | 1.39 | 0.54 | 0.98 | 0.74 | 1.43 |
| 1.52 | 0.82 | 1.06 | 1.26 | 1.6 | 0.51 | 0.49 | 0.8 | 2.49 | 0.49 | 1.44 |
| 1.04 | 0.81 | 1.18 | 2.98 | 0.82 | 0.53 | 0.93 | 0.56 | 1.47 | 0.88 | 0.95 |
| 0.88 | 0.98 | 0.75 | 1.57 | 1.17 | 0.67 | 1.07 | 0.94 | 0.84 | 1.41 | 0.76 |
| 2.21 | 1.24 | 0.28 | 1.07 | 4.89 | 1.11 | 1.21 | 0.98 | 0.75 | 0.75 | 1.18 |
| 0.35 | 0.78 | 0.52 | 0.04 | 0.09 | 0.41 | 1.79 | 1 | 1.14 | 6.79 | 0.89 |
| 1.26 | 0.94 | 1.86 | 0.44 | 1.54 | 0.55 | 0.85 | 0.83 | 0.97 | 0.76 | 0.9 |
| 0.86 | 1 | 0.88 | 10.86 | 1.03 | 1.19 | 1.14 | 0.76 | 1.12 | 0.76 | 1.01 |
| 0.88 | 0.87 | 0.59 | 1.54 | 0.74 | 0.38 | 0.97 | 0.55 | 2.34 | 1.03 | 1.65 |
| 2 | 1.5 | 1.47 | 3.77 | 0.84 | 1.41 | 0.5 | 0.9 | 0.83 | 3.43 | 0.93 |
| 1.26 | 0.82 | 1.27 | 0.91 | 0.79 | 0.86 | 0.83 | 0.92 | 0.9 | 3.16 | 0.77 |

| | | | | | | | | | | |
|---|---|---|---|---|---|---|---|---|---|---|
| 0.96 | 0.89 | 0.62 | 0.75 | 0.63 | 0.99 | 0.69 | 0.94 | 0.98 | 1.37 | 1.1 |
| 2.24 | 21.99 | 0.83 | 1.25 | 2.21 | 3.42 | 3.19 | 1.65 | 0.97 | 4.73 | 2.09 |
| 1.14 | 1.27 | 1.12 | 0.95 | 1.33 | 1.83 | 1.26 | 1.06 | 0.96 | 0.71 | 0.94 |
| 1.19 | 0.85 | 0.4 | 6.78 | 0.38 | 1.79 | 0.76 | 1.22 | 0.76 | 0.55 | 0.95 |
| 2.52 | 2.8 | 0.47 | 0.86 | 0.03 | 4.46 | 2.42 | 4.16 | 0.49 | 1.69 | 0.81 |
| 1.26 | 1.2 | 5.39 | 1.55 | 0.86 | 0.74 | 0.78 | 0.6 | 0.78 | 1.53 | 0.96 |
| 0.92 | 0.83 | 2.09 | 1.17 | 0.2 | 1.8 | 0.93 | 0.96 | 0.58 | 0.97 | 0.76 |
| 1.22 | 0.99 | 1 | 0.8 | 0.85 | 0.82 | 0.78 | 0.98 | 1.1 | 1.17 | 1 |
| 0.89 | 1.12 | 0.71 | 0.37 | 7.07 | 1.08 | 0.89 | 1.1 | 1.39 | 0.55 | 1.24 |
| 0.8 | 0.71 | 0.4 | 0.03 | 2.47 | 1.07 | 1.88 | 0.71 | 1.05 | 0.48 | 0.48 |
| 0.87 | 0.94 | 0.99 | 1.11 | 1.03 | 0.98 | 1.12 | 0.98 | 1.11 | 0.71 | 0.84 |
| 1.08 | 0.97 | 1.66 | 0.99 | 1.27 | 1.04 | 0.95 | 1.02 | 1.02 | 0.47 | 0.88 |
| 1.78 | 1.23 | 0.76 | 1.06 | 1.57 | 0.14 | 1.41 | 1.13 | 0.86 | 0.44 | 1.9 |
| 1.73 | 1.5 | 1.64 | 1.51 | 0.56 | 1.53 | 1.49 | 0.76 | 1.13 | 2.84 | 1.36 |
| 1.8 | 1.12 | 0.86 | 0.92 | 1.64 | 0.49 | 1.22 | 1.08 | 0.89 | 0.6 | 1.66 |
| 1 | 1.1 | 1.06 | 1.35 | 16.03 | 1.21 | 1.59 | 0.92 | 0.81 | 1.23 | 1.05 |
| 1.02 | 0.96 | 0.86 | 1.04 | 1.58 | 1.18 | 1.14 | 1.05 | 1.14 | 0.86 | 1.09 |
| 1.01 | 0.79 | 0.85 | 1.25 | 4.3 | 1.01 | 1.1 | 1.2 | 1.04 | 1.38 | 1.33 |
| 0.99 | 0.97 | 1.2 | 0.85 | 1.03 | 1.02 | 1.06 | 1.08 | 0.84 | 0.9 | 0.98 |
| 1.17 | 0.85 | 1.29 | 2.38 | 2.71 | 0.83 | 0.89 | 1.06 | 0.82 | 6.01 | 1.97 |
| 0.9 | 1.08 | 0.69 | 0.86 | 0.91 | 1.09 | 0.81 | 1.17 | 0.92 | 0.74 | 0.84 |
| 0.97 | 0.89 | 0.86 | 1.71 | Inf | 0.87 | 1.06 | 0.95 | 0.93 | 5.45 | 0.88 |
| 0.87 | 0.97 | 0.82 | 1.15 | 1.8 | 0.91 | 0.7 | 1.02 | 0.93 | 1.2 | 0.89 |
| 1.07 | 1.14 | 1.02 | 1.05 | 0.95 | 0.82 | 1.13 | 0.91 | 1.24 | 0.64 | 0.89 |
| 0.79 | 0.91 | 1.67 | 0.79 | 0.11 | 0.92 | 0.66 | 0.97 | 1.03 | 1.96 | 1.01 |
| 1.32 | 0.49 | 1.01 | 23.86 | 0.56 | 5.8 | 1.16 | 1.35 | 0.82 | 0.62 | 0.78 |
| 0.8 | 0.55 | 1.08 | 0.68 | 0.66 | 0.52 | 1.15 | 0.46 | 1.51 | 0.59 | 1.13 |
| 1.13 | 0.65 | 0.88 | 1.75 | 0.83 | 2.25 | 2.16 | 0.95 | 1.31 | 0.5 | 1.45 |
| 0.87 | 1.04 | 1.52 | 0.54 | 0.91 | 0.72 | 1.43 | 0.86 | 1.02 | 0.82 | 1.2 |
| 2.82 | 1.25 | 0.86 | 1.23 | 2.19 | 0.4 | 1.22 | 1.23 | 0.76 | 0.46 | 2.01 |
| 0.72 | 1.09 | 0.61 | 0.8 | 1.05 | 0.93 | 0.92 | 0.86 | 1.45 | 0.67 | 1.08 |
| 0.89 | 0.9 | 1.39 | 1.05 | 1.71 | 1.19 | 1.61 | 1.15 | 0.96 | 1.04 | 1.66 |
| 0.98 | 0.95 | 1.53 | 0.6 | 1.12 | 0.79 | 1.02 | 0.89 | 1.07 | 142.34 | 0.93 |

| | | | | | | | | | | |
|---|---|---|---|---|---|---|---|---|---|---|
| 0.99 | 1.22 | 0.72 | 2 | 1.38 | 0.89 | 1.04 | 1.03 | 1.15 | 0.91 | 0.92 |
| 2.16 | 0.86 | 1.81 | 2.41 | 0.77 | 1.33 | 0.99 | 0.91 | 1 | 1.85 | 1.83 |
| 0.97 | 1.14 | 0.74 | 0.96 | 1.43 | 1.03 | 1 | 1.04 | 0.99 | 1 | 0.84 |
| 1.22 | 1.33 | 1.47 | 1.64 | 7.15 | 1.46 | 0.88 | 1.26 | 0.94 | 1.37 | 1.27 |
| 0.94 | 0.96 | 0.99 | 0.88 | 0.98 | 1.17 | 1.21 | 1 | 0.94 | 1.04 | 1.03 |
| 1.37 | 1.24 | 1.69 | 0.99 | 0.37 | 1.04 | 1.65 | 1.18 | 1.7 | 1.04 | 0.54 |
| 1.36 | 0.53 | 1.12 | 0.43 | 3.12 | 0.93 | 0.6 | 0.71 | 2.13 | 1.83 | 0.34 |
| 0.78 | 0.82 | 0.94 | 0.72 | 1.32 | 0.97 | 1.26 | 0.89 | 1.15 | 1.1 | 0.81 |
| 1.31 | 0.7 | 0.75 | 0.73 | 7.64 | 1.04 | 6.85 | 1.19 | 1.5 | 1.08 | 0.95 |
| 1.01 | 1.15 | 1.27 | 1.46 | 7.76 | 0.72 | 1.61 | 1.39 | 0.95 | 0.8 | 1.64 |
| 0.91 | 0.96 | 1.56 | 2.23 | 9.94 | 2.12 | 0.5 | 0.75 | 1.34 | 2.8 | 0.95 |
| 1.22 | 1 | 1.36 | 1.47 | 0.91 | 1.11 | 1.07 | 0.81 | 0.89 | 0.74 | 0.77 |
| 0.98 | 1.2 | 1.34 | 0.83 | 0.74 | 0.6 | 1.35 | 0.68 | 0.9 | 1.33 | 1.27 |
| 1.42 | 1.26 | 1.24 | 1.41 | 1.15 | 1.27 | 0.99 | 1.13 | 0.77 | 2 | 1.36 |
| 1.19 | 0.98 | 0.91 | 1.29 | 1.43 | 0.93 | 0.87 | 0.97 | 1.17 | 1 | 0.57 |
| 10.69 | 476.22 | 0.3 | 0.14 | 24.89 | 50.09 | 9.99 | 18.84 | 0.64 | 52.03 | 1.97 |
| 0.85 | 1.18 | 1.04 | 0.33 | 4.65 | 0.58 | 1.44 | 0.83 | 1.09 | 0.53 | 0.73 |
| 2.47 | 1.26 | 0.89 | 2.86 | 0.79 | 1 | 1.29 | 1.27 | 0.32 | 0.13 | 0.25 |
| 1.38 | 0.98 | 0.79 | 0.85 | 1.24 | 1.29 | 1.21 | 1.11 | 0.91 | 1.22 | 1.31 |
| 1.1 | 0.98 | 2.04 | 0.8 | 4.47 | 1.12 | 1.04 | 0.93 | 1.1 | 1.02 | 0.66 |
| 1.55 | 0.95 | 1.71 | 0.73 | 1.56 | 0.42 | 1.25 | 0.62 | 1.4 | 0.33 | 1.02 |
| 0.88 | 0.81 | 0.94 | 1.35 | 1.04 | 2.16 | 1.2 | 0.93 | 1.17 | 1.17 | 0.78 |
| 1.99 | 0.89 | 0.39 | 0.21 | 2.42 | 0.73 | 0.93 | 1.08 | 1.53 | 0.87 | 1.26 |
| 0.59 | 0.86 | 0.73 | 39.08 | 0.43 | 2.14 | 0.8 | 1.58 | 1.08 | 0.51 | 1.05 |
| 0.97 | 1.04 | 1.17 | 0.87 | 1.56 | 1.2 | 1.17 | 1.06 | 1.1 | 1.04 | 0.83 |
| 1.06 | 1.09 | 0.67 | 0.65 | 1.01 | 0.8 | 0.9 | 1.02 | 1.17 | 1.07 | 1.04 |
| 1.09 | 0.63 | 1.1 | 0.9 | 0.73 | 0.71 | 0.99 | 0.65 | 0.94 | 1.28 | 1.01 |
| 0.94 | 0.93 | 1.51 | 0.95 | 0.81 | 1.04 | 1 | 0.91 | 0.87 | 1.31 | 0.66 |
| 0.86 | 1.01 | 0.98 | 0.91 | 0.91 | 0.82 | 0.79 | 1 | 1.21 | 1.07 | 0.81 |
| 1.07 | 0.92 | 1.05 | 1.2 | 0.97 | 1.35 | 0.89 | 1.01 | 1.02 | 1.03 | 0.99 |
| 1.06 | 1.05 | 1.28 | 0.92 | 0.69 | 0.88 | 1.07 | 0.95 | 1.11 | 0.83 | 1.12 |
| 1.33 | 1.62 | 0.79 | 0.78 | 0.42 | 0.66 | 0.73 | 1.18 | 1.17 | 0.62 | 1.19 |
| 1.1 | 1.22 | 1.37 | 0.95 | 0.88 | 1.01 | 1.39 | 1.14 | 1.12 | 0.77 | 1.13 |

| | | | | | | | | | | |
|---|---|---|---|---|---|---|---|---|---|---|
| 1.08 | 0.86 | 1 | 1.19 | 2.43 | 0.99 | 1.31 | 1.24 | 0.92 | 2.26 | 1.11 |
| 1 | 1.04 | 1.17 | 0.82 | 0.49 | 0.95 | 0.83 | 0.94 | 0.88 | 0.92 | 0.88 |
| 0.77 | 1.09 | 1.18 | 1.09 | 1.62 | 1.12 | 0.75 | 1.09 | 1.64 | 1.59 | 1 |
| 1.18 | 0.78 | 1.3 | 0.89 | 5.38 | 0.65 | 4.71 | 1.21 | 1.08 | 0.6 | 0.75 |
| 1.42 | 1.61 | 0.86 | 1.67 | 0.66 | 0.99 | 1.72 | 1.68 | 1.67 | 1.17 | 0.62 |
| 0.63 | 0.8 | 0.65 | 0.71 | 1.02 | 0.47 | 0.5 | 0.2 | 1.18 | 0.62 | 0.4 |
| 1.75 | 0.86 | 0.81 | 0.76 | 0.54 | 0.8 | 1.47 | 0.89 | 0.93 | 1.52 | 2.08 |
| 1.11 | 2.44 | 0.87 | 1.27 | 1.38 | 0.99 | 0.94 | 1.61 | 0.62 | 0.71 | 0.89 |
| 0.49 | 1.14 | 0.86 | 0.49 | 2.83 | 0.95 | 0.57 | 1 | 0.65 | 1.65 | 0.57 |
| 1.4 | 0.93 | 0.97 | 0.82 | 0.7 | 0.86 | 1.01 | 0.92 | 1 | 1.39 | 1.16 |
| 1.64 | 1 | 1.06 | 2.41 | 0.82 | 0.92 | 0.91 | 1.12 | 0.97 | 0.65 | 0.98 |
| 1.12 | 0.95 | 1.31 | 1.99 | 2.26 | 1.18 | 1.14 | 1.09 | 1.12 | 1.26 | 1.07 |
| 0.93 | 0.98 | 1.78 | 1.56 | 0.81 | 1.32 | 0.73 | 1.09 | 0.69 | 1.03 | 0.98 |
| 1.04 | 0.86 | 0.57 | 5.13 | 0.54 | 1.99 | 0.87 | 1.27 | 0.95 | 0.84 | 1.28 |
| 1.13 | 1.23 | 0.99 | 0.91 | 1.19 | 0.86 | 1.26 | 1.16 | 0.92 | 2.56 | 1.07 |
| 0.95 | 0.94 | 1.79 | 0.96 | 1.22 | 1.09 | 1.24 | 1.27 | 0.82 | 1.01 | 1.03 |
| 1.13 | 1.01 | 1.24 | 1 | 1.05 | 0.76 | 0.92 | 1.03 | 0.48 | 1.32 | 2.07 |
| 1.32 | 1.04 | 1.76 | 1.39 | 4.76 | 1.46 | 1.19 | 1.01 | 0.98 | 1.75 | 1.47 |
| 0.93 | 0.8 | 1.13 | 0.88 | 0.71 | 0.75 | 1.2 | 0.84 | 1.04 | 0.97 | 1.18 |
| 0.82 | 0.91 | 1.47 | 2.12 | 2.39 | 1.38 | 0.7 | 1.92 | 0.78 | 0.81 | 1.62 |
| 1.09 | 1.23 | 1.03 | 0.47 | 1.46 | 1.16 | 0.98 | 1.4 | 0.93 | 0.85 | 1.23 |
| 1.13 | 0.95 | 1.18 | 0.94 | 0.75 | 0.91 | 0.71 | 0.92 | 1.46 | 1.09 | 0.96 |
| 1.13 | 1.26 | 1.13 | 1.34 | 0.42 | 0.81 | 1.1 | 1.1 | 1.15 | 1.21 | 1.17 |
| 1.22 | 0.72 | 2.95 | 0.69 | 0.29 | 1.02 | 1.86 | 1.13 | 0.88 | 1.73 | 1.01 |
| 1.27 | 0.77 | 1.7 | 1.25 | 0.75 | 0.98 | 0.88 | 0.81 | 0.99 | 0.87 | 0.81 |
| 0.92 | 0.81 | 0.54 | 1.44 | 0.96 | 0.95 | 1.08 | 1.08 | 0.86 | 1.76 | 0.6 |
| 1.05 | 1.04 | 1.01 | 1.02 | 2.57 | 0.77 | 1.29 | 0.98 | 1.04 | 1.23 | 0.83 |
| 0.96 | 1.2 | 1.5 | 2.5 | 1.98 | 1.26 | 1.33 | 1.18 | 1.02 | 2.06 | 1.77 |
| 0.61 | 0.7 | 0.51 | 1.39 | 0.72 | 3.85 | 0.79 | 0.89 | 1.26 | 1.32 | 1.37 |
| 0.84 | 1 | 1.12 | 0.94 | 0.92 | 1.11 | 0.82 | 1.09 | 0.86 | 0.9 | 1.03 |
| 0.97 | 0.99 | 1.12 | 0.82 | 0.61 | 0.91 | 0.84 | 1.09 | 1.04 | 1.04 | 0.79 |
| 2.16 | 0.86 | 1.81 | 2.41 | 0.77 | 1.33 | 0.99 | 0.91 | 1 | 1.85 | 1.83 |
| 1.54 | 0.93 | 0.89 | 0.7 | 2.24 | 1.03 | 1.09 | 0.97 | 1.41 | 1.59 | 1.56 |

| | | | | | | | | | | |
|---|---|---|---|---|---|---|---|---|---|---|
| 0.75 | 1.24 | 0.98 | 1.47 | 1.1 | 0.79 | 0.89 | 0.93 | 1.28 | 1.3 | 0.56 |
| 1.11 | 0.58 | 0.95 | 0.67 | 6.08 | 0.91 | 0.82 | 0.85 | 0.88 | 1.04 | 1.01 |
| 0.79 | 1.05 | 1.23 | 0.75 | 2.45 | 0.73 | 1.09 | 0.82 | 1.35 | 1.12 | 0.96 |
| 1.13 | 1.03 | 1.23 | 0.99 | 0.97 | 0.94 | 0.91 | 0.93 | 1.17 | 1.08 | 1.04 |
| 1.17 | 1.04 | 0.67 | 0.4 | 0.87 | 0.76 | 0.98 | 0.95 | 1.1 | 0.97 | 0.97 |
| 0.88 | 0.99 | 1.04 | 0.52 | 0.89 | 0.91 | 0.9 | 1.01 | 0.96 | 1.1 | 0.92 |
| 0.98 | 1.22 | 1.2 | 0.84 | 0.89 | 0.85 | 1 | 1.06 | 0.88 | 1.19 | 0.8 |
| 1.07 | 1.08 | 1.28 | 0.99 | 1.33 | 1.07 | 0.92 | 1.13 | 0.75 | 0.84 | 1.11 |
| 1.65 | 0.82 | 1.47 | 1.4 | 0.73 | 1.25 | 1.23 | 0.82 | 1.01 | 0.65 | 1.28 |
| 1.11 | 1.17 | 1.38 | 0.87 | 1 | 0.92 | 1.06 | 1.17 | 0.97 | 0.84 | 0.95 |
| 1.06 | 0.92 | 1.05 | 0.42 | 0.8 | 0.87 | 1.14 | 1.08 | 1.22 | 1.09 | 1.04 |
| 1.04 | 1.03 | 0.8 | 1.04 | 2.38 | 0.95 | 1.06 | 1.08 | 1.1 | 1.15 | 0.96 |
| 1.25 | 1.06 | 1.72 | 1.5 | 5.15 | 1.38 | 1.07 | 1.17 | 1.03 | 1.6 | 1.34 |
| 1.05 | 1.11 | 1.53 | 0.85 | 0.87 | 0.76 | 1.09 | 1.09 | 0.76 | 0.93 | 1.35 |
| 0.85 | 1.36 | 0.45 | 1.22 | 1.74 | 0.61 | 1.38 | 1.38 | 0.8 | 0.41 | 0.67 |
| 1.05 | 1.02 | 1.07 | 1.09 | 1.3 | 0.78 | 1.1 | 0.97 | 1.07 | 0.87 | 1.22 |
| 0.98 | 1.01 | 0.89 | 0.83 | 2.15 | 1.09 | 0.91 | 1.08 | 0.88 | 0.92 | 1.19 |
| 0.91 | 0.96 | 1.03 | 1.33 | 0.79 | 2.08 | 0.9 | 1.06 | 0.68 | 2.9 | 1.45 |
| 1.02 | 0.94 | 0.8 | 0.95 | 0.96 | 0.84 | 0.96 | 0.98 | 1.08 | 1.03 | 1.05 |
| 1.13 | 0.88 | 0.85 | 0.35 | 2.2 | 0.99 | 0.84 | 0.85 | 1.73 | 0.77 | 1.53 |
| 1.08 | 0.92 | 1.46 | 0.24 | 1.58 | 0.87 | 1.04 | 1.14 | 0.78 | 0.49 | 1.18 |
| 1.49 | 0.92 | 0.46 | 0.19 | Inf | 0.77 | 1.21 | 1.05 | 0.71 | 0.46 | 1.17 |
| 0.95 | 1.17 | 1.02 | 1.29 | 1.16 | 1.05 | 1.06 | 0.96 | 1.1 | 0.85 | 0.78 |
| 1.5 | 1.04 | 1.37 | 1.41 | 0.95 | 2.93 | 0.94 | 0.77 | 0.88 | 0.68 | 1.03 |
| 1.01 | 0.94 | 1.32 | 0.53 | 0.97 | 0.42 | 1.38 | 0.79 | 1.21 | 0.94 | 0.92 |
| 1.78 | 1.31 | 1.06 | 4.45 | 0.66 | 2.64 | 1.15 | 0.92 | 0.95 | 0.89 | 0.86 |
| 1.07 | 0.98 | 0.88 | 0.86 | 0.69 | 0.77 | 1.29 | 1.09 | 0.89 | 0.88 | 1.35 |
| 0.96 | 0.95 | 1.25 | 0.67 | 3.08 | 0.84 | 0.82 | 1.12 | 0.82 | 1.4 | 1.02 |
| 0.94 | 1.05 | 1.05 | 0.87 | 0.96 | 1 | 1.07 | 0.95 | 1.1 | 0.67 | 1.09 |
| 0.79 | 0.72 | 0.97 | 0.48 | 1.05 | 0.7 | 1.19 | 1.1 | 1.12 | 0.93 | 0.84 |
| 0.74 | 1.1 | 0.85 | 0.85 | 0.91 | 1.14 | 0.81 | 1.04 | 1 | 0.72 | 0.77 |
| 0.91 | 0.95 | 0.8 | 0.96 | 0.86 | 1.06 | 1.01 | 0.95 | 1 | 1.01 | 0.96 |
| 0.98 | 1 | 0.8 | 0.99 | 1.06 | 1.06 | 1.1 | 0.96 | 0.98 | 0.94 | 1.05 |

| | | | | | | | | | | |
|---|---|---|---|---|---|---|---|---|---|---|
| 0.88 | 0.89 | 0.69 | 0.97 | 2.89 | 0.79 | 6.69 | 2.07 | 1.06 | 0.81 | 1.59 |
| 0.8 | 1.18 | 0.85 | 1.08 | 0.99 | 1.08 | 0.82 | 1.07 | 0.91 | 0.89 | 0.84 |
| 1.24 | 0.94 | 0.93 | 1.01 | 0.85 | 1.59 | 1.01 | 1.11 | 0.9 | 0.96 | 0.95 |
| 0.86 | 1.96 | 1.34 | 2.34 | 1.04 | 1.49 | 1.19 | 1.56 | 0.78 | 0.77 | 0.99 |
| 0.86 | 0.75 | 0.68 | 2.98 | 1.69 | 1.15 | 0.51 | 1.25 | 0.91 | 0.68 | 1.19 |
| 0.94 | 0.97 | 1.2 | 0.99 | 0.82 | 1.07 | 0.99 | 0.92 | 1.04 | 0.92 | 1.19 |
| 0.97 | 0.98 | 0.66 | 0.81 | 0.7 | 0.94 | 0.7 | 0.91 | 1.06 | 1.4 | 0.98 |
| 1.2 | 0.99 | 1.4 | 0.94 | 1.29 | 1.27 | 0.93 | 1.01 | 0.97 | 2.13 | 1.03 |
| 1.11 | 1.1 | 1.18 | 1.8 | 0.91 | 1.44 | 0.94 | 1.24 | 0.98 | 0.71 | 0.97 |
| 1 | 0.81 | 0.6 | 0.81 | 0.52 | 1.09 | 0.68 | 0.99 | 1.08 | 1.63 | 1.01 |
| 1.07 | 0.96 | 1.18 | 2.28 | 0.95 | 1.6 | 0.73 | 1.21 | 1.02 | 0.9 | 0.45 |
| 0.61 | 1.02 | 0.52 | 0.91 | 0.65 | 1.46 | 0.83 | 1.38 | 0.8 | 0.93 | 0.96 |
| 0.91 | 0.89 | 0.84 | 0.79 | 0.91 | 1.07 | 0.92 | 1.04 | 1.04 | 0.98 | 0.7 |
| 0.78 | 1.04 | 0.93 | 0.98 | 0.93 | 1.03 | 0.91 | 0.98 | 0.87 | 0.92 | 1.27 |
| 1.14 | 0.97 | 1.12 | 1.07 | 1.07 | 0.98 | 0.92 | 0.94 | 1.12 | 0.97 | 1.04 |
| 1.03 | 1.25 | 0.71 | 0.67 | 0.92 | 1.06 | 1.06 | 1.17 | 1.07 | 1.52 | 1.21 |
| 1.03 | 0.96 | 1.14 | 0.83 | 0.92 | 0.97 | 1.19 | 1.01 | 1.2 | 0.86 | 1.01 |
| 0.96 | 1.03 | 1.17 | 0.92 | 0.91 | 1 | 0.78 | 0.94 | 0.88 | 0.97 | 0.96 |
| 1 | 1.27 | 0.93 | 1.37 | 1.12 | 1.92 | 1.21 | 1.19 | 0.85 | 1.24 | 0.99 |
| 0.62 | 1.2 | 0.8 | 1.19 | 1.18 | 1.17 | 1.12 | 1.14 | 1.08 | 0.99 | 0.6 |
| 0.9 | 0.91 | 2.1 | 1.35 | 1.64 | 0.79 | 1 | 0.93 | 0.94 | 1.01 | 0.79 |
| 1.46 | 0.89 | 1.09 | 5.83 | 0.35 | 2.03 | 0.57 | 0.74 | 1.2 | 1.33 | 1.9 |
| 0.79 | 1.01 | 0.89 | 1.07 | 0.76 | 0.91 | 0.96 | 0.95 | 0.88 | 1.34 | 1.04 |
| 0.85 | 1.08 | 0.98 | 0.88 | 0.52 | 1.28 | 3.2 | 1.54 | 0.84 | 0.89 | 1.12 |
| 0.91 | 0.85 | 0.87 | 0.81 | 0.88 | 1.01 | 1.13 | 0.9 | 0.97 | 1.27 | 1.62 |
| 1.37 | 1.8 | 1.75 | 1.97 | 2.52 | 0.88 | 1.07 | 0.96 | 0.71 | 0.27 | 1.65 |
| 1.4 | 0.92 | 1 | 0.87 | 1.21 | 1 | 0.87 | 1.12 | 1.02 | 0.84 | 1.1 |
| 0.91 | 0.95 | 1.14 | 1.04 | 0.88 | 0.94 | 0.95 | 0.87 | 1.06 | 0.93 | 1.01 |
| 1.03 | 0.96 | 0.97 | 0.99 | 1.01 | 1.16 | 1.12 | 1.13 | 0.87 | 0.93 | 1.11 |
| 1.17 | 0.94 | 1.02 | 0.99 | 0.93 | 0.82 | 0.84 | 0.96 | 0.63 | 0.86 | 0.93 |
| 0.83 | 0.87 | 0.89 | 0.96 | 17.28 | 0.56 | 1.16 | 0.99 | 1.16 | 1.49 | 0.86 |
| 0.86 | 1.1 | 0.88 | 0.79 | 1.58 | 0.9 | 0.84 | 1.05 | 0.93 | 0.88 | 0.91 |
| 1.23 | 1 | 1.23 | 10.67 | 2.91 | 0.6 | 1 | 0.86 | 1.67 | 0.75 | 0.79 |

| | | | | | | | | | | |
|---|---|---|---|---|---|---|---|---|---|---|
| 1.16 | 1.02 | 1.13 | 0.46 | 1.18 | 0.63 | 1.18 | 0.98 | 1.02 | 0.74 | 0.88 |
| 1.45 | 1.14 | 1.4 | 1.35 | 0.51 | 1.48 | 0.94 | 1.25 | 1.35 | 1.34 | 1.72 |
| 1.51 | 0.99 | 0.85 | 0.62 | Inf | 0.71 | 0.83 | 1 | 0.78 | 0.78 | 0.94 |
| 0.65 | 0.99 | 0.63 | 0.54 | 0.98 | 1.75 | 0.62 | 0.85 | 0.91 | 0.87 | 0.94 |
| 0.78 | 0.94 | 1.04 | 0.98 | 1.25 | 2.21 | 0.93 | 1.19 | 0.73 | 0.93 | 1.67 |
| 1.04 | 0.97 | 1.04 | 0.85 | 1.2 | 1.07 | 0.94 | 1.02 | 1.03 | 0.95 | 0.78 |
| 0.93 | 1.22 | 0.56 | 0.45 | 3.79 | 1.09 | 0.99 | 1.14 | 0.99 | 0.8 | 1.2 |
| 1.01 | 0.98 | 0.63 | 0.77 | 0.98 | 0.93 | 0.8 | 0.9 | 1.37 | 0.78 | 1.06 |
| 1.15 | 0.9 | 0.82 | 0.86 | 0.69 | 1.1 | 3.39 | 1.54 | 0.82 | 0.83 | 1.17 |
| 1.18 | 1.51 | 1.16 | 0.69 | 4.19 | 0.75 | 0.94 | 0.93 | 1.13 | 0.41 | 1.06 |
| 1.17 | 1.24 | 0.32 | 0.93 | 0.82 | 0.91 | 0.77 | 1.1 | 1.23 | 1.1 | 1.32 |
| 1.14 | 1.03 | 0.91 | 1.03 | 0.82 | 0.95 | 0.83 | 1.22 | 1.1 | 0.89 | 1.13 |
| 1 | 1.12 | 0.83 | 0.71 | 0.78 | 0.97 | 0.76 | 0.97 | 0.71 | 1.17 | 0.63 |
| 0.91 | 1.13 | 1.11 | 0.81 | 0.84 | 0.99 | 0.96 | 1.27 | 0.94 | 0.75 | 0.92 |
| 0.78 | 1.24 | 1 | 0.46 | 0.84 | 0.54 | 0.91 | 0.91 | 1.19 | 0.81 | 1 |
| 1.17 | 0.82 | 0.9 | 0.87 | 0.38 | 1.36 | 0.67 | 1.21 | 0.78 | 0.44 | 0.92 |
| 0.73 | 0.86 | 1.47 | 0.79 | 0.69 | 0.91 | 0.74 | 0.9 | 0.99 | 0.98 | 1.02 |
| 0.89 | 0.91 | 0.7 | 0.67 | 0.9 | 0.99 | 0.84 | 0.87 | 0.95 | 0.68 | 0.81 |
| 0.79 | 0.77 | 1.06 | 0.48 | 0.88 | 0.71 | 2.21 | 0.65 | 1.5 | 42.28 | 0.66 |
| 2.78 | 1.74 | 0.4 | 0.42 | 24.94 | 0.7 | 0.76 | 1.46 | 1.46 | 0.35 | 0.63 |
| 0.86 | 0.9 | 1.07 | 1.57 | 0.53 | 0.62 | 1.33 | 0.76 | 0.68 | 1.71 | 1.03 |
| 0.98 | 0.81 | 0.6 | 0.76 | 0.54 | 1.07 | 0.68 | 0.97 | 0.99 | 1.49 | 1.11 |
| 1.1 | 0.79 | 0.56 | 11.22 | 0.46 | 4.72 | 0.93 | 0.96 | 1.79 | 1.32 | 2.29 |
| 0.65 | 0.84 | 0.53 | 1.18 | 1.95 | 0.31 | 0.84 | 0.76 | 1.29 | 1.93 | 1.28 |
| 1.09 | 0.96 | 1.1 | 0.67 | 1.54 | 0.77 | 1.24 | 1 | 1.05 | 0.8 | 0.76 |
| 0.96 | 0.97 | 1.04 | 0.68 | 1.04 | 0.76 | 1.33 | 0.72 | 0.9 | 0.72 | 0.92 |
| 0.54 | 1.39 | 1.18 | 1.23 | 0.82 | 0.85 | 0.82 | 1.18 | 1.05 | 1.1 | 0.67 |
| 1.27 | 1.12 | 0.49 | 0.6 | 3.16 | 0.89 | 1.14 | 1.35 | 1.19 | 0.68 | 1.33 |
| 0.69 | 0.93 | 0.7 | 1.54 | 0.63 | 1.22 | 0.75 | 0.82 | 0.76 | 0.72 | 0.81 |
| 4.2 | 0.91 | 3 | 2.9 | 1.44 | 0.41 | 1.99 | 0.46 | 1.35 | 0.27 | 0.74 |
| 0.58 | 0.89 | 0.71 | 1.41 | 1.24 | 0.88 | 1.27 | 0.79 | 0.95 | 0.64 | 0.45 |
| 0.59 | 1.3 | 1.1 | 0.44 | 2.12 | 0.97 | 0.94 | 1.18 | 0.92 | 0.11 | 0.88 |
| 1.06 | 0.88 | 1.81 | 1.41 | 0.41 | 0.93 | 1.4 | 0.75 | 1.16 | 1.44 | 1.03 |

| | | | | | | | | | | |
|---|---|---|---|---|---|---|---|---|---|---|
| 0.92 | 1.13 | 1 | 0.65 | 0.94 | 0.97 | 1.07 | 1.17 | 0.92 | 0.86 | 0.97 |
| 1.01 | 1.03 | 0.79 | 1.27 | 1.15 | 1.02 | 1.06 | 0.91 | 1.05 | 1.17 | 1.37 |
| 0.99 | 15.8 | 0.96 | 3.26 | 0.08 | 2.61 | 2.45 | 23.95 | 0.83 | 1.02 | 3.32 |
| 2.83 | 1 | 0.96 | 0.62 | 0.56 | 1.68 | 0.59 | 0.98 | 0.6 | 0.45 | 1.03 |
| 1.4 | 0.93 | 0.97 | 1.25 | 2.53 | 1.05 | 0.73 | 1.09 | 1.22 | 0.82 | 1.05 |
| 1.17 | 1.11 | 1.07 | 2.41 | 1.27 | 1.47 | 1.28 | 0.96 | 0.97 | 1.32 | 0.97 |
| 0.88 | 1.22 | 1.11 | 1.13 | 0.84 | 1.19 | 1.25 | 1.07 | 0.99 | 0.89 | 0.91 |
| 1.2 | 0.92 | 1.32 | 3.29 | 0.58 | 1.06 | 1.07 | 1.04 | 1.17 | 1.09 | 1 |
| 0.84 | 1.09 | 1.38 | 1.26 | 2.63 | 0.81 | 1.24 | 1.1 | 1.2 | 0.78 | 0.82 |
| 0.68 | 0.99 | 0.9 | 0.83 | 1.13 | 1.06 | 1.08 | 1.11 | 0.98 | 0.57 | 1.05 |
| 0.97 | 1.01 | 1.05 | 0.6 | 1.53 | 1.32 | 1.25 | 0.92 | 0.94 | 0.87 | 1.43 |
| 0.04 | 114.69 | 3.33 | 2.33 | 6.27 | 4.96 | 1.69 | 181.72 | 11.77 | 14.57 | 44.84 |
| 0.92 | 0.94 | 0.87 | 1.72 | 0.86 | 0.94 | 1.35 | 1.08 | 1.01 | 0.89 | 0.84 |
| 1.7 | 1.17 | 0.76 | 2.28 | 1.3 | 0.86 | 1.16 | 1.13 | 0.65 | 0.76 | 0.75 |

| 209341_s_at | 219195_at | 201746_at | 220070_at | 201984_s_at | 203739_at | 212377_s_at | 204178_s_at | 213342_at | 202431_s_at | 213906_at |
|---|---|---|---|---|---|---|---|---|---|---|
| 1.02 | 5.28 | 0.92 | 1.09 | 1.36 | 0.98 | 1.04 | 1.08 | 1.02 | 1.04 | 1.16 |
| 1.16 | 1.39 | 0.88 | 0.94 | 1.16 | 0.82 | 0.65 | 1.26 | 2.17 | 0.41 | 0.88 |
| 1.85 | 1 | 0.94 | 1.05 | 1.96 | 1.27 | 0.68 | 0.86 | 2.24 | 1.13 | 6.87 |
| 1.14 | 4.86 | 1.02 | 2.13 | 2.77 | 0.8 | 0.96 | 1.59 | 1.37 | 1.17 | 1.48 |
| 1.51 | 1.92 | 0.89 | 5.09 | 1.11 | 1.07 | 1.33 | 1 | 1.07 | 1.22 | 1.13 |
| 0.71 | 2.15 | 0.95 | 3.33 | 3.33 | 1.5 | 1.15 | 1.82 | 3.76 | 735.49 | 1.05 |
| 1.34 | 0.29 | 0.14 | 1.48 | 0.55 | 1.34 | 1.08 | 1.17 | 1.04 | 0.85 | 2.09 |
| 0.95 | 2.36 | 1.36 | 1.49 | 0.53 | 0.81 | 1.24 | 1.33 | 3.5 | 1.08 | 1.24 |
| 1 | 0.93 | 0.82 | 1.73 | 0.75 | 0.96 | 1.06 | 0.75 | 1.01 | 0.82 | 1.04 |
| 0.75 | 8.72 | 1.42 | 1.25 | 1.39 | 1.24 | 0.61 | 1.27 | 1.04 | 1.95 | 1.61 |
| 1.27 | 1.52 | 0.68 | 0.8 | 0.91 | 0.74 | 0.88 | 0.86 | 0.63 | 0.89 | 1.06 |
| 1.1 | 1.26 | 0.93 | 2.39 | 0.87 | 0.92 | 0.79 | 1.41 | 1.22 | 0.97 | 1.63 |
| 1.16 | 1.5 | 1.26 | 1.83 | 0.78 | 1.23 | 1.08 | 1.13 | 0.91 | 1.03 | 0.93 |
| 0.26 | 30.01 | 0.19 | 1.27 | 0.34 | 0.34 | 0.28 | 3.52 | 0.47 | 0.15 | 4.15 |
| 1.47 | 2.91 | 1.11 | 1.06 | 0.89 | 1.39 | 0.9 | 1.23 | 1.04 | 1.71 | 0.67 |
| 1.21 | 3.93 | 1.33 | 0.96 | 0.48 | 0.73 | 0.79 | 1.01 | 1.26 | 0.92 | 0.99 |
| 1.25 | 0.12 | 0.99 | 0.6 | 1.6 | 0.97 | 0.94 | 1.1 | 0.94 | 0.83 | 2.25 |
| 1.05 | 0.54 | 1.13 | 1.02 | 0.8 | 1.13 | 1.48 | 1.01 | 1.36 | 0.74 | 1.07 |
| 1.32 | 0.67 | 0.1 | 1.15 | 0.48 | 0.98 | 0.96 | 0.97 | 2.72 | 0.22 | 0.64 |
| 1.39 | 1.19 | 0.96 | 0.86 | 1.49 | 0.67 | 1.12 | 0.82 | 0.93 | 0.74 | 0.52 |
| 0.49 | 0.36 | 1.87 | 3.24 | 2.99 | 1.18 | 0.41 | 1.83 | 0.88 | 0.49 | 0.99 |
| 0.93 | 15.33 | 3.15 | 0.62 | 2.25 | 0.6 | 1.14 | 1.02 | 0.53 | 0.88 | 0.84 |
| 1.07 | 5.78 | 1.32 | 0.97 | 0.8 | 1 | 0.99 | 0.89 | 0.87 | 1.41 | 0.77 |
| 1.21 | 0.5 | 0.95 | 1.1 | 0.59 | 1.26 | 1.38 | 1.21 | 0.78 | 0.75 | 1.82 |
| 1.79 | 0.06 | 0.63 | 0.67 | 3.23 | 1.96 | 0.93 | 0.82 | 1.35 | 6.79 | 8.39 |
| 0.85 | 0.19 | 1.11 | 1.23 | 0.94 | 0.79 | 1.3 | 1.26 | 1.07 | 0.76 | 0.99 |
| 1.14 | 0.57 | 1.93 | 1.62 | 1.16 | 0.84 | 0.78 | 0.77 | 0.87 | 0.76 | 0.98 |
| 0.97 | 1.51 | 6.62 | 0.82 | 2.11 | 0.71 | 1.07 | 0.85 | 0.67 | 1.03 | 0.49 |
| 0.5 | 1.08 | 0.15 | 1.25 | 0.8 | 1.88 | 0.45 | 1.04 | 0.51 | 3.43 | 0.46 |
| 0.83 | 0.97 | 0.73 | 0.91 | 0.67 | 0.95 | 1.28 | 1.01 | 1.32 | 3.16 | 1.5 |

| | | | | | | | | | | |
|---|---|---|---|---|---|---|---|---|---|---|
| 0.69 | 5.58 | 0.56 | 0.89 | 2.15 | 0.55 | 0.74 | 1.21 | 5.19 | 1.37 | 0.85 |
| 3.19 | 29.66 | 1.38 | 3.29 | 1.7 | 4.62 | 3.03 | 6 | 1.16 | 4.73 | 0.89 |
| 1.26 | 0.97 | 1.07 | 1.98 | 1.01 | 1.03 | 1.05 | 1.01 | 0.96 | 0.71 | 2.29 |
| 0.76 | 2.16 | 1.27 | 1.38 | 0.52 | 0.46 | 0.59 | 1.02 | 0.78 | 0.55 | 3.25 |
| 2.42 | 1.5 | 16 | 0.99 | 38.56 | 0.18 | 0.5 | 0.13 | 40.4 | 1.69 | 4.12 |
| 0.78 | 0.67 | 1.65 | 1.16 | 1.02 | 0.58 | 0.89 | 0.71 | 1.2 | 1.53 | 0.9 |
| 0.93 | 20.97 | 0.57 | 2.81 | 0.13 | 0.61 | 0.8 | 1 | 0.57 | 0.97 | 0.36 |
| 0.78 | 0.07 | 0.88 | 1.42 | 0.87 | 0.85 | 0.95 | 0.72 | 0.88 | 1.17 | 0.94 |
| 0.89 | 0.83 | 1.24 | 0.71 | 0.46 | 1.16 | 0.83 | 0.76 | 0.63 | 0.55 | 0.66 |
| 1.88 | 0.53 | 1.01 | 1.35 | 2.64 | 1.25 | 1.74 | 0.53 | 1.02 | 0.48 | 3.14 |
| 1.12 | 1.75 | 2.01 | 0.47 | 0.74 | 0.7 | 0.95 | 1.25 | 1.08 | 0.71 | 0.68 |
| 0.95 | 1.59 | 0.95 | 0.99 | 1.32 | 0.96 | 11.25 | 0.91 | 0.9 | 0.47 | 1.19 |
| 1.41 | 0.42 | 0.26 | 0.92 | 0.81 | 1.83 | 1.87 | 0.44 | 1.36 | 0.44 | 0.3 |
| 1.49 | 1.4 | 2.55 | 0.53 | 0.84 | 0.72 | 0.97 | 0.64 | 1.62 | 2.84 | 0.18 |
| 1.22 | 0.56 | 0.41 | 0.85 | 0.72 | 1.59 | 1.55 | 0.55 | 1.24 | 0.6 | 0.68 |
| 1.59 | 21.09 | 1.09 | 0.96 | 1.27 | 1.22 | 0.94 | 1.02 | 1.29 | 1.23 | 4.45 |
| 1.14 | 2.33 | 0.97 | 1.34 | 1.02 | 0.98 | 1.05 | 1.05 | 0.84 | 0.86 | 0.87 |
| 1.1 | 4.56 | 0.81 | 0.67 | 1.13 | 0.73 | 1.02 | 0.95 | 1.23 | 1.38 | 1.61 |
| 1.06 | 1.17 | 0.86 | 1.45 | 0.82 | 1.01 | 1.06 | 0.92 | 0.95 | 0.9 | 1.01 |
| 0.89 | 3.49 | 0.69 | 0.83 | 1.48 | 1.12 | 0.96 | 1.39 | 0.83 | 6.01 | 1.58 |
| 0.81 | 3.5 | 0.96 | 0.84 | 0.8 | 1.04 | 0.82 | 0.96 | 0.81 | 0.74 | 0.52 |
| 1.06 | 0.22 | 1.05 | 1.18 | 1.45 | 0.83 | 0.93 | 0.96 | 0.73 | 5.45 | 1.06 |
| 0.7 | 0.51 | 1.09 | 1.42 | 1.35 | 1.11 | 1.12 | 0.94 | 1.18 | 1.2 | 1.12 |
| 1.13 | 1.47 | 0.81 | 0.78 | 1.55 | 0.98 | 1.17 | 1.18 | 2.26 | 0.64 | 0.86 |
| 0.66 | 1.06 | 0.35 | 0.81 | 1.52 | 1.57 | 1.51 | 1.17 | 1.23 | 1.96 | 0.18 |
| 1.16 | 0.12 | 1.48 | 1.27 | 1.23 | 0.7 | 0.75 | 1.66 | 1.26 | 0.62 | 3.85 |
| 1.15 | 0.2 | 0.93 | 1.6 | 6.59 | 1.76 | 0.64 | 2.1 | 1.54 | 0.59 | 0.75 |
| 2.16 | 2.37 | 1.99 | 1.13 | 1.27 | 1.2 | 0.74 | 0.61 | 1.18 | 0.5 | 0.78 |
| 1.43 | 1.49 | 0.25 | 1.23 | 1.2 | 1.21 | 1.26 | 0.79 | 0.95 | 0.82 | 0.56 |
| 1.22 | 0.27 | 0.41 | 0.94 | 0.69 | 2.05 | 1.65 | 0.47 | 1.5 | 0.46 | 0.3 |
| 0.92 | 0.61 | 0.73 | 1.01 | 0.94 | 1.18 | 0.78 | 0.92 | 1.09 | 0.67 | 0.72 |
| 1.61 | 1.38 | 0.79 | 1.75 | 1.36 | 1.74 | 1.73 | 0.85 | 0.98 | 1.04 | 1.53 |
| 1.02 | 0.81 | 1.14 | 1.04 | 1.49 | 0.9 | 0.71 | 0.83 | 1.27 | 142.34 | 0.65 |

| | | | | | | | | | | |
|---|---|---|---|---|---|---|---|---|---|---|
| 1.04 | 0.25 | 0.91 | 0.62 | 0.9 | 1.16 | 1.17 | 0.9 | 0.98 | 0.91 | 3.26 |
| 0.99 | 4.71 | 1.49 | 2.72 | 0.58 | 1.14 | 0.73 | 1.2 | 0.78 | 1.85 | 2.96 |
| 1 | 2.8 | 0.89 | 1.22 | 1.29 | 1.25 | 1.61 | 1.03 | 0.98 | 1 | 1.59 |
| 0.88 | 1.42 | 0.87 | 1.41 | 1.22 | 1.06 | 1.27 | 1.03 | 1.13 | 1.37 | 2.46 |
| 1.21 | 4.7 | 0.85 | 0.98 | 1.08 | 0.9 | 1.14 | 0.81 | 0.9 | 1.04 | 1.05 |
| 1.65 | 0.65 | 1.35 | 0.64 | 1.28 | 1.09 | 1.19 | 0.9 | 0.74 | 1.04 | 2.06 |
| 0.6 | 2.83 | 1.65 | 1.84 | 3.19 | 0.9 | 1.95 | 1.41 | 1.73 | 1.83 | 0.23 |
| 1.26 | 0.64 | 0.98 | 1.31 | 1.28 | 0.78 | 0.99 | 1.56 | 0.87 | 1.1 | 1.14 |
| 6.85 | 1.19 | 0.92 | 0.93 | 1.71 | 1.55 | 1.62 | 1.17 | 0.88 | 1.08 | 0.79 |
| 1.61 | 0.52 | 1.12 | 1.23 | 1.26 | 2.02 | 1.3 | 0.76 | 1.26 | 0.8 | 1.08 |
| 0.5 | 5.73 | 1.34 | 1.89 | 1.57 | 1.36 | 0.84 | 1.44 | 2.14 | 2.8 | 2.71 |
| 1.07 | 0.1 | 0.9 | 1.44 | 1.49 | 1.15 | 0.97 | 1.01 | 1.26 | 0.74 | 1.72 |
| 1.35 | 0.97 | 1.19 | 0.74 | 1.15 | 1.21 | 0.99 | 0.76 | 1.11 | 1.33 | 0.57 |
| 0.99 | 1.23 | 1.01 | 1.54 | 0.82 | 1.05 | 1.07 | 0.73 | 1.18 | 2 | 0.77 |
| 0.87 | 0.17 | 0.42 | 1.87 | 0.5 | 0.97 | 1.05 | 1.25 | 2 | 1 | 1.5 |
| 9.99 | 1.4 | 4.36 | 0.16 | 4.6 | 102.5 | 4.79 | 0.74 | 52.59 | 52.03 | 1.1 |
| 1.44 | 1.89 | 1.36 | 0.85 | 1.55 | 2.06 | 1.14 | 0.77 | 1.07 | 0.53 | 0.37 |
| 1.29 | 4.88 | 0.32 | 1 | 0.52 | 1.79 | 1.58 | 0.73 | 2.06 | 0.13 | 1.3 |
| 1.21 | 1.54 | 0.9 | 0.88 | 1.26 | 0.98 | 1.34 | 0.95 | 1.52 | 1.22 | 0.92 |
| 1.04 | 3.51 | 0.9 | 2.93 | 2.74 | 0.87 | 0.87 | 1.22 | 5.36 | 1.02 | 5.14 |
| 1.25 | 2.21 | 0.72 | 2.59 | 2.74 | 0.55 | 1.47 | 0.9 | 0.88 | 0.33 | 0.45 |
| 1.2 | 3.34 | 0.91 | 1.01 | 2.36 | 1.28 | 1.48 | 1.15 | 0.78 | 1.17 | 7.54 |
| 0.93 | 0.17 | 0.11 | 1.55 | 1.13 | 1.37 | 0.95 | 1.12 | 0.86 | 0.87 | 2.05 |
| 0.8 | 1.15 | 0.97 | 0.87 | 0.85 | 0.78 | 0.67 | 3.02 | 1.26 | 0.51 | 17.12 |
| 1.17 | 6.37 | 1.37 | 1.57 | 1.06 | 1.22 | 0.98 | 0.86 | 1.55 | 1.04 | 1.05 |
| 0.9 | 1.4 | 0.91 | 0.88 | 1.21 | 0.88 | 0.84 | 1.02 | 1.15 | 1.07 | 0.64 |
| 0.99 | 0.76 | 1.03 | 0.71 | 1.1 | 0.82 | 0.95 | 1.28 | 0.84 | 1.28 | 0.62 |
| 1 | 16.98 | 1.09 | 1.14 | 1.09 | 0.86 | 1.04 | 1 | 2.29 | 1.31 | 0.79 |
| 0.79 | 1.16 | 1.05 | 0.88 | 2.52 | 0.91 | 1.1 | 1.07 | 1.2 | 1.07 | 0.99 |
| 0.89 | 3.73 | 0.94 | 0.99 | 1.69 | 0.9 | 1.05 | 1.22 | 1.02 | 1.03 | 1.21 |
| 1.07 | 0.8 | 1.22 | 0.83 | 0.64 | 1.09 | 0.99 | 1.12 | 0.9 | 0.83 | 1 |
| 0.73 | 3.11 | 0.56 | 2.66 | 0.81 | 0.57 | 0.68 | 0.76 | 0.82 | 0.62 | 1.06 |
| 1.39 | 7.15 | 1.08 | 1.13 | 0.68 | 1.35 | 1.82 | 0.9 | 1.65 | 0.77 | 0.8 |

| | | | | | | | | | | |
|---|---|---|---|---|---|---|---|---|---|---|
| 1.31 | 2.09 | 0.94 | 1.65 | 0.92 | 0.9 | 0.95 | 1.08 | 0.8 | 2.26 | 1.03 |
| 0.83 | 1.57 | 0.86 | 0.77 | 0.92 | 0.77 | 0.84 | 0.95 | 0.63 | 0.92 | 0.86 |
| 0.75 | 0.99 | 1.42 | 3.92 | 0.75 | 0.68 | 0.84 | 1.08 | 0.99 | 1.59 | 1.53 |
| 4.71 | 1.9 | 0.96 | 0.67 | 1.91 | 0.69 | 1.57 | 1.29 | 1.11 | 0.6 | 0.69 |
| 1.72 | 10.09 | 1 | 1.8 | 1.07 | 0.95 | 1.26 | 1.21 | 0.73 | 1.17 | 1.07 |
| 0.5 | 1.58 | 0.73 | 0.47 | 0.54 | 0.67 | 0.28 | 0.71 | 0.6 | 0.62 | 0.54 |
| 1.47 | 1.53 | 1.13 | 0.87 | 1.55 | 1.45 | 0.98 | 0.78 | 1.16 | 1.52 | 1.08 |
| 0.94 | 0.89 | 1.12 | 1.24 | 0.93 | 1.44 | 1.29 | 0.82 | 1.26 | 0.71 | 1.14 |
| 0.57 | 3.25 | 0.75 | 1.11 | 3.6 | 1.03 | 1.19 | 1.03 | 1.23 | 1.65 | 0.84 |
| 1.01 | 0.81 | 1.33 | 0.73 | 4.06 | 1.22 | 1.08 | 0.91 | 1.19 | 1.39 | 1.14 |
| 0.91 | 0.76 | 1.43 | 1.69 | 1.03 | 0.84 | 0.88 | 1.03 | 1.2 | 0.65 | 0.9 |
| 1.14 | 1.78 | 1.05 | 1.02 | 1.28 | 1.11 | 0.96 | 1.22 | 0.91 | 1.26 | 1.13 |
| 0.73 | 0.9 | 0.79 | 1.14 | 1.35 | 0.95 | 1.08 | 1.68 | 0.91 | 1.03 | 1.33 |
| 0.87 | 1.26 | 1.16 | 1.09 | 0.7 | 0.78 | 0.79 | 1.17 | 0.98 | 0.84 | 2.9 |
| 1.26 | 1.08 | 1.08 | 1.05 | 0.71 | 1.04 | 0.71 | 0.83 | 1.11 | 2.56 | 0.9 |
| 1.24 | 1.14 | 1.14 | 0.94 | 1.2 | 1.06 | 1.12 | 0.98 | 3.63 | 1.01 | 0.79 |
| 0.92 | 1.14 | 1.26 | 1.03 | 1.54 | 1.03 | 1.07 | 1.06 | 0.56 | 1.32 | 0.93 |
| 1.19 | 1.27 | 0.91 | 1.38 | 1.44 | 1.02 | 1.3 | 1.2 | 1.29 | 1.75 | 1.77 |
| 1.2 | 7.4 | 1.14 | 0.76 | 1.51 | 0.83 | 1.32 | 0.95 | 1.48 | 0.97 | 0.56 |
| 0.7 | 3.83 | 0.7 | 1.41 | 1.1 | 0.77 | 1.21 | 0.98 | 0.96 | 0.81 | 2.22 |
| 0.98 | 0.92 | 0.7 | 0.76 | 0.88 | 1.1 | 1.16 | 1.59 | 1.34 | 0.85 | 1.33 |
| 0.71 | 1.58 | 1.09 | 1.41 | 3.3 | 1.08 | 0.91 | 0.92 | 1.03 | 1.09 | 1.06 |
| 1.1 | 0.92 | 1.03 | 1.05 | 1.07 | 0.98 | 1.15 | 1.05 | 0.76 | 1.21 | 1.01 |
| 1.86 | 1.24 | 1.63 | 0.96 | 0.61 | 1.65 | 1.1 | 0.86 | 0.67 | 1.73 | 0.35 |
| 0.88 | 1.7 | 1.31 | 0.35 | 1.2 | 0.5 | 1.02 | 0.98 | 1.2 | 0.87 | 0.59 |
| 1.08 | 2.36 | 1.21 | 0.93 | 1.14 | 1.08 | 1.26 | 0.78 | 1.39 | 1.76 | 1.27 |
| 1.29 | 0.15 | 1.03 | 0.68 | 1.22 | 1.3 | 1.13 | 0.95 | 0.99 | 1.23 | 1.08 |
| 1.33 | 1.93 | 1.32 | 0.64 | 2.46 | 1.58 | 1.03 | 0.61 | 0.87 | 2.06 | 1.11 |
| 0.79 | 0.9 | 0.52 | 0.39 | 0.98 | 0.9 | 1.01 | 1.39 | 1.12 | 1.32 | 2.59 |
| 0.82 | 1.49 | 0.94 | 2.69 | 0.86 | 1.22 | 1.07 | 1.19 | 0.86 | 0.9 | 1.16 |
| 0.84 | 0.72 | 5.78 | 0.91 | 0.98 | 1.31 | 1.11 | 0.93 | 0.2 | 1.04 | 1.14 |
| 0.99 | 4.71 | 1.49 | 2.72 | 0.58 | 1.14 | 0.73 | 1.2 | 0.78 | 1.85 | 2.96 |
| 1.09 | 1.44 | 0.97 | 1.02 | 1.24 | 1.18 | 1.18 | 1.01 | 1.15 | 1.59 | 1.69 |

| | | | | | | | | | | |
|---|---|---|---|---|---|---|---|---|---|---|
| 0.89 | 4.25 | 1.58 | 1.48 | 1.38 | 1.22 | 1.08 | 1.02 | 1.32 | 1.3 | 0.95 |
| 0.82 | 0.98 | 0.93 | 1.04 | 1.66 | 1.2 | 1.32 | 1.04 | 1.01 | 1.04 | 0.7 |
| 1.09 | 1.58 | 1.09 | 0.51 | 0.94 | 0.85 | 0.96 | 0.74 | 1.25 | 1.12 | 0.64 |
| 0.91 | 1.52 | 1.28 | 2.18 | 1.55 | 0.87 | 1.19 | 0.95 | 8.57 | 1.08 | 0.6 |
| 0.98 | 1.25 | 1.35 | 1.04 | 1.06 | 0.96 | 1.06 | 0.78 | 0.94 | 0.97 | 0.76 |
| 0.9 | 2 | 0.75 | 1.08 | 0.98 | 0.99 | 0.9 | 1.12 | 1.05 | 1.1 | 0.9 |
| 1 | 0.98 | 0.89 | 0.51 | 0.86 | 1.09 | 1 | 1 | 0.94 | 1.19 | 0.79 |
| 0.92 | 4.47 | 0.98 | 0.79 | 0.88 | 0.89 | 0.92 | 0.81 | 1.18 | 0.84 | 0.82 |
| 1.23 | 1.46 | 1.3 | 0.5 | 0.63 | 1.23 | 0.74 | 0.99 | 0.62 | 0.65 | 0.6 |
| 1.06 | 21.73 | 1.24 | 1.26 | 0.61 | 1.08 | 1.85 | 1.05 | 1.05 | 0.84 | 1.41 |
| 1.14 | 1.07 | 0.96 | 1.94 | 0.84 | 0.93 | 0.6 | 1.01 | 0.59 | 1.09 | 0.63 |
| 1.06 | 2.07 | 1.17 | 3.43 | 1.25 | 1.54 | 0.89 | 0.99 | 1.09 | 1.15 | 1.12 |
| 1.07 | 1.59 | 0.88 | 1.53 | 1.38 | 1.02 | 1.35 | 1.21 | 1.15 | 1.6 | 1.74 |
| 1.09 | 3.68 | 0.88 | 2.59 | 0.96 | 0.85 | 0.74 | 0.62 | 1.08 | 0.93 | 0.45 |
| 1.38 | 3.14 | 0.29 | 0.97 | 2.04 | 1.67 | 1.45 | 0.81 | 2.65 | 0.41 | 0.51 |
| 1.1 | 0.84 | 1.01 | 0.85 | 0.95 | 0.93 | 1.17 | 1.13 | 0.28 | 0.87 | 1.02 |
| 0.91 | 1.23 | 0.95 | 0.63 | 1.22 | 1.11 | 0.9 | 0.94 | 0.84 | 0.92 | 1.83 |
| 0.9 | 0.48 | 1.12 | 1.03 | 1.13 | 0.87 | 1.03 | 1.82 | 2.47 | 2.9 | 0.62 |
| 0.96 | 0.89 | 3.3 | 1.47 | 0.81 | 0.88 | 1.1 | 1.04 | 0.87 | 1.03 | 0.96 |
| 0.84 | 1.04 | 1.5 | 0.9 | 1.09 | 0.92 | 1.01 | 0.91 | 1.02 | 0.77 | 0.79 |
| 1.04 | 0.75 | 1.02 | 0.92 | 0.82 | 1.38 | 1.18 | 0.78 | 1.15 | 0.49 | 0.85 |
| 1.21 | 1.19 | 1.41 | 1.1 | 1.26 | 0.98 | 1.26 | 1.16 | 1.23 | 0.46 | 1.34 |
| 1.06 | 1.48 | 1.13 | 1.31 | 6.22 | 1.32 | 1.16 | 0.91 | 3.56 | 0.85 | 1.18 |
| 0.94 | 0.72 | 1.01 | 2.15 | 0.91 | 0.76 | 0.64 | 0.63 | 1.37 | 0.68 | 0.77 |
| 1.38 | 0.64 | 1 | 0.98 | 1.34 | 1 | 0.89 | 1.19 | 1.32 | 0.94 | 0.46 |
| 1.15 | 2.14 | 1.09 | 0.96 | 0.6 | 0.85 | 0.77 | 1.06 | 0.82 | 0.89 | 0.58 |
| 1.29 | 2.35 | 1.31 | 0.74 | 1 | 0.94 | 1.03 | 1.15 | 0.83 | 0.88 | 1.11 |
| 0.82 | 1.21 | 0.9 | 2.39 | 1.03 | 0.76 | 0.92 | 1.25 | 1.13 | 1.4 | 0.66 |
| 1.07 | 0.95 | 1.01 | 0.58 | 1.04 | 1.19 | 1.06 | 0.83 | 1.2 | 0.67 | 1.09 |
| 1.19 | 3 | 0.83 | 0.77 | 0.8 | 0.86 | 1.02 | 0.81 | 0.83 | 0.93 | 0.76 |
| 0.81 | 3.78 | 1.24 | 1.41 | 4.74 | 1 | 0.8 | 1.19 | 2.19 | 0.72 | 1.27 |
| 1.01 | 0.99 | 0.95 | 1.01 | 1.15 | 1.03 | 1.09 | 1.07 | 1.03 | 1.01 | 1.18 |
| 1.1 | 2.66 | 1.06 | 1.47 | 0.7 | 1.04 | 0.95 | 1.07 | 3.17 | 0.94 | 0.98 |

| | | | | | | | | | | |
|---|---|---|---|---|---|---|---|---|---|---|
| 6.69 | 0.88 | 0.81 | 0.33 | 1.06 | 1.33 | 1.5 | 1.58 | 1.31 | 0.81 | 1.03 |
| 0.82 | 1.81 | 1.22 | 0.87 | 4.74 | 0.91 | 0.92 | 1.31 | 1.12 | 0.89 | 1.22 |
| 1.01 | 0.89 | 0.97 | 1.04 | 1.01 | 0.88 | 1.08 | 1.09 | 1.04 | 0.96 | 1.31 |
| 1.19 | 1.97 | 3.63 | 0.88 | 1.27 | 1.49 | 1.27 | 0.74 | 0.5 | 0.77 | 1.12 |
| 0.51 | 1.54 | 0.93 | 14.76 | 0.34 | 0.89 | 1.34 | 2.1 | 0.75 | 0.68 | 6.09 |
| 0.99 | 2.25 | 1.62 | 0.96 | 1.15 | 1 | 0.97 | 1.29 | 0.95 | 0.92 | 1.02 |
| 0.7 | 5.46 | 0.54 | 0.88 | 1.09 | 0.51 | 0.74 | 1.17 | 4.07 | 1.4 | 0.77 |
| 0.93 | 1.03 | 0.9 | 0.84 | 0.79 | 1.24 | 0.53 | 0.96 | 0.77 | 2.13 | 1 |
| 0.94 | 4.35 | 0.91 | 1.26 | 2.65 | 1.02 | 1.02 | 1.13 | 1.62 | 0.71 | 2.52 |
| 0.68 | 1.28 | 0.61 | 0.86 | 3.62 | 0.5 | 0.79 | 1.22 | 2.85 | 1.63 | 0.75 |
| 0.73 | 0.99 | 1.3 | 0.62 | 1.63 | 0.67 | 1.18 | 0.78 | 0.93 | 0.9 | 2.9 |
| 0.83 | 0.85 | 1.82 | 4.19 | 0.88 | 0.62 | 0.99 | 1.11 | 1.27 | 0.93 | 1.6 |
| 0.92 | 0.48 | 0.81 | 0.82 | 1.26 | 1.14 | 0.88 | 0.93 | 0.85 | 0.98 | 1.35 |
| 0.91 | 2.75 | 0.95 | 3.18 | 0.77 | 1.01 | 1.05 | 0.98 | 4.53 | 0.92 | 0.83 |
| 0.92 | 1.88 | 1.4 | 4.41 | 1.33 | 0.86 | 1.19 | 1 | 3.14 | 0.97 | 0.6 |
| 1.06 | 1.44 | 1.39 | 0.89 | 1.06 | 1.07 | 0.95 | 1.14 | 1.18 | 1.52 | 0.77 |
| 1.19 | 3.02 | 0.91 | 1.44 | 1.42 | 0.88 | 0.99 | 1.44 | 5 | 0.86 | 1.46 |
| 0.78 | 0.87 | 0.97 | 1.1 | 0.89 | 0.87 | 0.98 | 0.98 | 1.01 | 0.97 | 0.84 |
| 1.21 | 2.61 | 0.69 | 2.7 | 1.14 | 1.48 | 0.86 | 0.7 | 1.38 | 1.24 | 1.38 |
| 1.12 | 0.69 | 0.88 | 9.21 | 1.48 | 1.32 | 1.29 | 0.92 | 1.48 | 0.99 | 2.78 |
| 1 | 1.3 | 0.53 | 1.4 | 1.07 | 0.95 | 0.94 | 0.97 | 0.9 | 1.01 | 0.68 |
| 0.57 | 0.62 | 0.72 | 0.85 | 0.58 | 0.72 | 0.29 | 1.26 | 0.94 | 1.33 | 0.31 |
| 0.96 | 1.21 | 0.89 | 1.48 | 0.92 | 1.02 | 1.11 | 1.17 | 1.3 | 1.34 | 0.8 |
| 3.2 | 0.51 | 0.87 | 0.27 | 1.34 | 1.06 | 1.35 | 1.09 | 1.58 | 0.89 | 0.85 |
| 1.13 | 0.92 | 1.03 | 0.73 | 1.26 | 1 | 1.13 | 0.89 | 1.04 | 1.27 | 0.75 |
| 1.07 | 26.67 | 2.78 | 1.28 | 2.13 | 0.24 | 1.88 | 0.59 | 0.84 | 0.27 | 6.67 |
| 0.87 | 0.83 | 1.17 | 1.64 | 1.28 | 1.13 | 1.18 | 1.23 | 1.39 | 0.84 | 1.29 |
| 0.95 | 1.15 | 1.11 | 0.69 | 2.97 | 0.93 | 1.21 | 1.01 | 2.72 | 0.93 | 0.95 |
| 1.12 | 6.75 | 0.95 | 1.32 | 3.81 | 0.97 | 0.96 | 1.13 | 3.84 | 0.93 | 1.05 |
| 0.84 | 0.94 | 1.06 | 0.65 | 1.22 | 1.11 | 0.96 | 0.96 | 0.97 | 0.86 | 0.85 |
| 1.16 | 7.19 | 0.84 | 1.21 | 0.65 | 0.62 | 0.78 | 1.06 | 1.14 | 1.49 | 1.17 |
| 0.84 | 2 | 0.89 | 1.27 | 0.89 | 0.89 | 1.06 | 0.83 | 0.9 | 0.88 | 1.12 |
| 1 | 0.83 | 0.86 | 1.17 | 1.05 | 0.93 | 1.18 | 1.02 | 0.66 | 0.75 | 1.78 |

| | | | | | | | | | | |
|---|---|---|---|---|---|---|---|---|---|---|
| 1.18 | 1.19 | 0.83 | 1.48 | 0.92 | 1.02 | 1.1 | 0.92 | 0.95 | 0.74 | 0.8 |
| 0.94 | 0.33 | 1.12 | 0.83 | 1.18 | 1.02 | 1.02 | 0.91 | 1.44 | 1.34 | 1.19 |
| 0.83 | 0.48 | 1.06 | 1.02 | 0.82 | 0.57 | 1.21 | 1.16 | 0.79 | 0.78 | 1.57 |
| 0.62 | 0.55 | 0.66 | 0.83 | 0.65 | 0.82 | 0.87 | 1.08 | 0.74 | 0.87 | 0.97 |
| 0.93 | 2.78 | 0.85 | 0.94 | 0.65 | 0.77 | 0.82 | 1.63 | 2.21 | 0.93 | 0.74 |
| 0.94 | 4.25 | 0.64 | 0.94 | 0.71 | 0.99 | 1.23 | 0.82 | 1.08 | 0.95 | 1.09 |
| 0.99 | 0.28 | 0.83 | 0.48 | 1.04 | 1.36 | 1.16 | 0.9 | 0.78 | 0.8 | 1.69 |
| 0.8 | 2.23 | 1.13 | 1.31 | 1.98 | 0.89 | 1.13 | 0.94 | 0.29 | 0.78 | 1.07 |
| 3.39 | 1.19 | 0.99 | 0.4 | 1.04 | 1.27 | 1.09 | 1.42 | 0.79 | 0.83 | 0.78 |
| 0.94 | 1.83 | 0.98 | 0.82 | 1.13 | 1.29 | 1.32 | 0.88 | 1.4 | 0.41 | 0.89 |
| 0.77 | 0.93 | 1.26 | 0.61 | 0.97 | 0.94 | 0.9 | 0.9 | 1.33 | 1.1 | 0.76 |
| 0.83 | 0.5 | 0.82 | 2.5 | 1.15 | 1.13 | 0.98 | 1.09 | 0.62 | 0.89 | 0.64 |
| 0.76 | 1.04 | 1 | 1.47 | 1.17 | 0.58 | 1.14 | 0.93 | 0.8 | 1.17 | 1.27 |
| 0.96 | 0.66 | 4.58 | 0.71 | 1.33 | 1.44 | 1.06 | 1.08 | 0.17 | 0.75 | 1.22 |
| 0.91 | 1.85 | 1.06 | 1.55 | 0.89 | 0.89 | 0.94 | 0.76 | 0.92 | 0.81 | 0.8 |
| 0.67 | 3.31 | 0.81 | 1 | 0.83 | 0.77 | 1.03 | 1.07 | 1.01 | 0.44 | 0.43 |
| 0.74 | 1.48 | 0.6 | 0.99 | 0.78 | 0.97 | 1 | 0.91 | 0.82 | 0.98 | 1.04 |
| 0.84 | 0.62 | 1.14 | 0.77 | 1.17 | 0.92 | 1.15 | 0.87 | 9.36 | 0.68 | 0.82 |
| 2.21 | 0.61 | 1.4 | 2.18 | 0.66 | 0.57 | 1.28 | 1.35 | 1.3 | 42.28 | 0.36 |
| 0.76 | 2.04 | 1.14 | 1.55 | 0.44 | 0.39 | 0.75 | 1.21 | 0.5 | 0.35 | 1.81 |
| 1.33 | 1.5 | 0.75 | 0.6 | 0.9 | 0.94 | 0.94 | 0.92 | 0.9 | 1.71 | 0.91 |
| 0.68 | 3.48 | 0.6 | 0.88 | 3.42 | 0.54 | 0.77 | 1.24 | 4.58 | 1.49 | 0.85 |
| 0.93 | 0.18 | 1.07 | 0.96 | 0.47 | 0.87 | 0.7 | 1.28 | 0.99 | 1.32 | 7.21 |
| 0.84 | 1.16 | 0.59 | 0.49 | 0.36 | 0.93 | 1.92 | 0.9 | 1.11 | 1.93 | 3.1 |
| 1.24 | 1.94 | 0.89 | 1.11 | 1.14 | 1.01 | 1.15 | 1.03 | 0.99 | 0.8 | 0.84 |
| 1.33 | 3.48 | 1.11 | 1.05 | 0.95 | 0.81 | 0.85 | 1.05 | 1.39 | 0.72 | 0.8 |
| 0.82 | 0.87 | 1.19 | 1.09 | 0.98 | 1.53 | 0.61 | 1.16 | 0.63 | 1.1 | 2.34 |
| 1.14 | 3.1 | 1.19 | 0.95 | 0.96 | 1.08 | 1.1 | 0.78 | 1.14 | 0.68 | 1.54 |
| 0.75 | 1.03 | 0.61 | 0.8 | 0.72 | 1.05 | 1.36 | 1.6 | 1.73 | 0.72 | 0.76 |
| 1.99 | 0.27 | 0.5 | 0.62 | 1.78 | 0.99 | 0.9 | 0.32 | 3.36 | 0.27 | 1.79 |
| 1.27 | 0.69 | 1.41 | 1.16 | 1.85 | 1.02 | 0.7 | 0.98 | 2.14 | 0.64 | 1.64 |
| 0.94 | 1.06 | 0.98 | 1.04 | 2.11 | 4.2 | 0.8 | 0.81 | 2.41 | 0.11 | 1.76 |
| 1.4 | 2.52 | 0.64 | 2.13 | 0.45 | 0.62 | 1.27 | 2.48 | 1.58 | 1.44 | 0.76 |

| | | | | | | | | | | |
|---|---|---|---|---|---|---|---|---|---|---|
| 1.07 | 1.09 | 0.91 | 1.14 | 1.03 | 0.72 | 0.78 | 0.76 | 0.88 | 0.86 | 1.11 |
| 1.06 | 0.81 | 0.66 | 1.66 | 1.07 | 0.77 | 0.99 | 0.89 | 0.55 | 1.17 | 0.97 |
| 2.45 | 2.3 | 5.4 | 2.32 | 1.17 | 9.51 | 2.45 | 1.01 | 8.79 | 1.02 | 11.74 |
| 0.59 | 4.54 | 0.71 | 0.81 | 0.85 | 0.9 | 1.15 | 1.19 | 0.7 | 0.45 | 0.56 |
| 0.73 | 3.14 | 0.52 | 1.09 | 1.26 | 0.97 | 1.07 | 0.96 | 1.01 | 0.82 | 0.71 |
| 1.28 | 1.12 | 1.08 | 1.2 | 0.89 | 0.73 | 1.32 | 1.08 | 0.73 | 1.32 | 1.01 |
| 1.25 | 3.31 | 0.94 | 0.81 | 1.3 | 1.2 | 0.9 | 1.1 | 0.91 | 0.89 | 1.86 |
| 1.07 | 0.44 | 1.01 | 2.12 | 1.13 | 0.98 | 0.84 | 1.12 | 1.04 | 1.09 | 0.93 |
| 1.24 | 2.89 | 0.94 | 1.3 | 2.04 | 1.04 | 1.16 | 1.05 | 1.06 | 0.78 | 0.97 |
| 1.08 | 0.23 | 1.2 | 0.98 | 1.1 | 1.75 | 1.6 | 0.85 | 1.45 | 0.57 | 0.98 |
| 1.25 | 0.64 | 1.26 | 1.05 | 0.84 | 1.04 | 1.1 | 0.89 | 0.98 | 0.87 | 1.31 |
| 1.69 | 0.08 | 6.6 | 0.23 | 0.4 | 27.04 | 3.35 | 0.76 | 0.28 | 14.57 | 15.54 |
| 1.35 | 1.83 | 1.01 | 1.82 | 0.9 | 1.09 | 1.13 | 1.21 | 1.07 | 0.89 | 1.3 |
| 1.16 | 0.33 | 0.52 | 1.1 | 1.56 | 0.72 | 1.34 | 0.87 | 1.02 | 0.76 | 1.48 |

| 204346_s_at | 209341_s_at | 201502_s_at | 209341_s_at | 202431_s_at | 203132_at | 207163_s_at | 205407_at | 202431_s_at | 205932_s_at | 200639_s_at |
|---|---|---|---|---|---|---|---|---|---|---|
| 1.13 | 1.02 | 1.09 | 1.02 | 1.04 | 0.96 | 1.01 | 8.89 | 1.04 | 1.23 | 1 |
| 0.86 | 1.16 | 2.23 | 1.16 | 0.41 | 1.21 | 1.01 | 0.98 | 0.41 | 1.14 | 1.16 |
| 0.82 | 1.85 | 0.67 | 1.85 | 1.13 | 3.28 | 0.99 | 0.47 | 1.13 | 0.6 | 0.84 |
| 1.58 | 1.14 | 1.11 | 1.14 | 1.17 | 0.88 | 1.02 | 9.11 | 1.17 | 1.2 | 0.93 |
| 1.08 | 1.51 | 2.56 | 1.51 | 1.22 | 1 | 1 | 0.72 | 1.22 | 1.09 | 1.17 |
| 1.01 | 0.71 | 0.98 | 0.71 | 735.49 | 0.82 | 1.33 | 2.54 | 735.49 | 5.53 | 1 |
| 1.03 | 1.34 | 1.29 | 1.34 | 0.85 | 1.21 | 1.22 | 3.48 | 0.85 | 1.34 | 0.83 |
| 0.95 | 0.95 | 1.6 | 0.95 | 1.08 | 0.93 | 0.82 | 0.6 | 1.08 | 1.01 | 0.89 |
| 1.07 | 1 | 1.32 | 1 | 0.82 | 1.04 | 0.97 | 1.1 | 0.82 | 1.51 | 0.95 |
| 4.08 | 0.75 | 2.58 | 0.75 | 1.95 | 0.53 | 1.02 | 0.58 | 1.95 | 3.52 | 1.13 |
| 0.84 | 1.27 | 1.16 | 1.27 | 0.89 | 1.11 | 1.12 | 1.63 | 0.89 | 1.03 | 0.88 |
| 1.06 | 1.1 | 0.58 | 1.1 | 0.97 | 1 | 1.04 | 0.98 | 0.97 | 0.85 | 0.99 |
| 1.29 | 1.16 | 0.85 | 1.16 | 1.03 | 1.03 | 1.42 | 0.52 | 1.03 | 1.09 | 1.16 |
| 0.49 | 0.26 | 0.43 | 0.26 | 0.15 | 0.1 | 0.16 | 0.44 | 0.15 | 0.01 | 0.54 |
| 1.07 | 1.47 | 1.02 | 1.47 | 1.71 | 1.06 | 1.59 | 1.48 | 1.71 | 1.54 | 1.13 |
| 1.36 | 1.21 | 1.15 | 1.21 | 0.92 | 0.87 | 1.08 | 1.13 | 0.92 | 1.51 | 0.94 |
| 1.13 | 1.25 | 0.7 | 1.25 | 0.83 | 1.16 | 1.29 | 1.03 | 0.83 | 1.24 | 1.14 |
| 0.86 | 1.05 | 2.03 | 1.05 | 0.74 | 0.91 | 0.95 | 0.93 | 0.74 | 6 | 1.12 |
| 1.11 | 1.32 | 1.4 | 1.32 | 0.22 | 0.67 | 1.32 | 1.35 | 0.22 | 0.7 | 0.89 |
| 0.78 | 1.39 | 0.54 | 1.39 | 0.74 | 1.69 | 0.85 | 10.58 | 0.74 | 0.64 | 1.01 |
| 2.35 | 0.49 | 1.22 | 0.49 | 0.49 | 0.39 | 0.8 | 1.6 | 0.49 | 6.53 | 1.01 |
| 1.03 | 0.93 | 3.45 | 0.93 | 0.88 | 0.46 | 2.29 | 0.82 | 0.88 | 0.95 | 0.82 |
| 0.62 | 1.07 | 1.18 | 1.07 | 1.41 | 0.76 | 0.83 | 1.17 | 1.41 | 1.28 | 0.96 |
| 1.1 | 1.21 | 0.35 | 1.21 | 0.75 | 0.83 | 1.69 | 4.89 | 0.75 | 0.71 | 0.9 |
| 2.39 | 1.79 | 2.23 | 1.79 | 6.79 | 0.54 | 0.83 | 0.09 | 6.79 | 0.14 | 1.43 |
| 0.79 | 0.85 | 0.78 | 0.85 | 0.76 | 0.44 | 1.05 | 1.54 | 0.76 | 0.92 | 0.74 |
| 0.79 | 1.14 | 0.42 | 1.14 | 0.76 | 0.7 | 1.09 | 1.03 | 0.76 | 0.82 | 1.16 |
| 0.81 | 0.97 | 4.18 | 0.97 | 1.03 | 0.53 | 3.35 | 0.74 | 1.03 | 0.84 | 1.78 |
| 0.48 | 0.5 | 0.54 | 0.5 | 3.43 | 6.56 | 1.92 | 0.84 | 3.43 | 2.74 | 0.75 |
| 1.18 | 0.83 | 1.47 | 0.83 | 3.16 | 0.85 | 0.85 | 0.79 | 3.16 | 1.19 | 0.92 |

| | | | | | | | | | | |
|---|---|---|---|---|---|---|---|---|---|---|
| 0.92 | 0.69 | 0.66 | 0.69 | 1.37 | 0.8 | 0.96 | 0.63 | 1.37 | 3.69 | 1.09 |
| 5.76 | 3.19 | 0.93 | 3.19 | 4.73 | 10.55 | 4.92 | 2.21 | 4.73 | 8.14 | 3.68 |
| 0.94 | 1.26 | 0.84 | 1.26 | 0.71 | 1.18 | 1.02 | 1.33 | 0.71 | 1.18 | 0.98 |
| 1.39 | 0.76 | 0.35 | 0.76 | 0.55 | 1.34 | 1.49 | 0.38 | 0.55 | 0.72 | 0.64 |
| 0.73 | 2.42 | 2.61 | 2.42 | 1.69 | 1.64 | 1.34 | 0.03 | 1.69 | 0.73 | 0.96 |
| 4.76 | 0.78 | 0.81 | 0.78 | 1.53 | 1.08 | 1.44 | 0.86 | 1.53 | 3.14 | 0.8 |
| 0.68 | 0.93 | 2.35 | 0.93 | 0.97 | 0.86 | 0.49 | 0.2 | 0.97 | 1.98 | 1.16 |
| 0.94 | 0.78 | 0.71 | 0.78 | 1.17 | 0.74 | 0.98 | 0.85 | 1.17 | 1.29 | 1.12 |
| 0.67 | 0.89 | 3.57 | 0.89 | 0.55 | 0.9 | 1.29 | 7.07 | 0.55 | 3.54 | 1.02 |
| 0.69 | 1.88 | 1.29 | 1.88 | 0.48 | 1.07 | 0.91 | 2.47 | 0.48 | 2.54 | 1.08 |
| 1.62 | 1.12 | 0.91 | 1.12 | 0.71 | 1.42 | 1.48 | 1.03 | 0.71 | 1.34 | 1.12 |
| 0.92 | 0.95 | 1.24 | 0.95 | 0.47 | 0.85 | 0.91 | 1.27 | 0.47 | 1 | 1.07 |
| 2.16 | 1.41 | 3.43 | 1.41 | 0.44 | 1.17 | 0.52 | 1.57 | 0.44 | 1.91 | 0.92 |
| 24.61 | 1.49 | 0.54 | 1.49 | 2.84 | 6.84 | 0.73 | 0.56 | 2.84 | 1.61 | 1.17 |
| 1.76 | 1.22 | 2.85 | 1.22 | 0.6 | 1.05 | 0.55 | 1.64 | 0.6 | 1.42 | 0.91 |
| 1.05 | 1.59 | 2.51 | 1.59 | 1.23 | 1.04 | 1.14 | 16.03 | 1.23 | 1.13 | 1.37 |
| 1.2 | 1.14 | 7.24 | 1.14 | 0.86 | 1 | 1.01 | 1.58 | 0.86 | 0.88 | 0.94 |
| 1.28 | 1.1 | 1.45 | 1.1 | 1.38 | 0.97 | 0.86 | 4.3 | 1.38 | 0.85 | 0.97 |
| 0.85 | 1.06 | 0.94 | 1.06 | 0.9 | 1.16 | 0.85 | 1.03 | 0.9 | 0.94 | 0.94 |
| 1.1 | 0.89 | 0.6 | 0.89 | 6.01 | 0.77 | 0.93 | 2.71 | 6.01 | 1.41 | 1.02 |
| 1.09 | 0.81 | 5.73 | 0.81 | 0.74 | 0.94 | 1.11 | 0.91 | 0.74 | 0.95 | 1.07 |
| 1.09 | 1.06 | 0.72 | 1.06 | 5.45 | 0.87 | 0.93 | Inf | 5.45 | 0.78 | 0.83 |
| 0.73 | 0.7 | 0.85 | 0.7 | 1.2 | 1.08 | 0.82 | 1.8 | 1.2 | 0.55 | 0.99 |
| 1.25 | 1.13 | 1.21 | 1.13 | 0.64 | 1.03 | 1.46 | 0.95 | 0.64 | 2.01 | 1.19 |
| 1.05 | 0.66 | 1.24 | 0.66 | 1.96 | 0.93 | 0.69 | 0.11 | 1.96 | 2.46 | 1.06 |
| 1.75 | 1.16 | 1.54 | 1.16 | 0.62 | 1.02 | 1.46 | 0.56 | 0.62 | 1.35 | 0.55 |
| 1.13 | 1.15 | 6.08 | 1.15 | 0.59 | 0.38 | 1.56 | 0.66 | 0.59 | 4.78 | 0.76 |
| 0.77 | 2.16 | 1.78 | 2.16 | 0.5 | 1.38 | 0.82 | 0.83 | 0.5 | 0.59 | 0.56 |
| 0.68 | 1.43 | 4.1 | 1.43 | 0.82 | 0.97 | 1.07 | 0.91 | 0.82 | 1.21 | 1.02 |
| 1.58 | 1.22 | 4.97 | 1.22 | 0.46 | 1.21 | 0.31 | 2.19 | 0.46 | 2.16 | 0.82 |
| 1.55 | 0.92 | 2.33 | 0.92 | 0.67 | 0.66 | 1.72 | 1.05 | 0.67 | 3.18 | 1.22 |
| 1.47 | 1.61 | 0.6 | 1.61 | 1.04 | 0.87 | 0.84 | 1.71 | 1.04 | 0.63 | 0.88 |
| 0.98 | 1.02 | 1.04 | 1.02 | 142.34 | 0.78 | 0.89 | 1.12 | 142.34 | 2.35 | 0.89 |

| | | | | | | | | | | |
|---|---|---|---|---|---|---|---|---|---|---|
| 0.95 | 1.04 | 0.81 | 1.04 | 0.91 | 1.06 | 0.89 | 1.38 | 0.91 | 0.65 | 1.35 |
| 1.22 | 0.99 | 1.08 | 0.99 | 1.85 | 1.2 | 1.3 | 0.77 | 1.85 | 7.72 | 1.05 |
| 0.89 | 1 | 1.68 | 1 | 1 | 1.19 | 0.92 | 1.43 | 1 | 1.49 | 1.13 |
| 1.03 | 0.88 | 1.41 | 0.88 | 1.37 | 6.21 | 0.88 | 7.15 | 1.37 | 1.17 | 1.05 |
| 0.95 | 1.21 | 0.94 | 1.21 | 1.04 | 1.17 | 0.96 | 0.98 | 1.04 | 0.66 | 0.84 |
| 1.56 | 1.65 | 1.07 | 1.65 | 1.04 | 1.15 | 1.1 | 0.37 | 1.04 | 1.13 | 0.83 |
| 3.03 | 0.6 | 0.34 | 0.6 | 1.83 | 0.89 | 1.57 | 3.12 | 1.83 | 1.07 | 1.04 |
| 1.26 | 1.26 | 1.55 | 1.26 | 1.1 | 0.79 | 3.02 | 1.32 | 1.1 | 0.65 | 0.95 |
| 1.43 | 6.85 | 1.33 | 6.85 | 1.08 | 0.81 | 0.95 | 7.64 | 1.08 | 1.18 | 0.96 |
| 0.89 | 1.61 | 1.05 | 1.61 | 0.8 | 2.58 | 0.77 | 7.76 | 0.8 | 1.09 | 1.12 |
| 7 | 0.5 | 0.71 | 0.5 | 2.8 | 0.65 | 2.08 | 9.94 | 2.8 | 2.29 | 1.1 |
| 1.06 | 1.07 | 1.14 | 1.07 | 0.74 | 1.05 | 0.83 | 0.91 | 0.74 | 1.24 | 1.06 |
| 1.04 | 1.35 | 1.14 | 1.35 | 1.33 | 0.88 | 1.08 | 0.74 | 1.33 | 1.32 | 1.05 |
| 1.48 | 0.99 | 2.69 | 0.99 | 2 | 1.22 | 0.71 | 1.15 | 2 | 0.98 | 1 |
| 0.9 | 0.87 | 0.93 | 0.87 | 1 | 1.06 | 0.94 | 1.43 | 1 | 0.74 | 0.91 |
| 4.65 | 9.99 | 2.52 | 9.99 | 52.03 | 148.25 | 5.01 | 24.89 | 52.03 | 8.98 | 36.43 |
| 0.71 | 1.44 | 2.22 | 1.44 | 0.53 | 1.06 | 1.21 | 4.65 | 0.53 | 1.02 | 1.11 |
| 0.86 | 1.29 | 0.98 | 1.29 | 0.13 | 1.71 | 0.67 | 0.79 | 0.13 | 1.73 | 1.25 |
| 1.33 | 1.21 | 0.74 | 1.21 | 1.22 | 1.4 | 1.02 | 1.24 | 1.22 | 0.9 | 1.32 |
| 1.4 | 1.04 | 1.34 | 1.04 | 1.02 | 1.03 | 2.39 | 4.47 | 1.02 | 6.68 | 0.91 |
| 1.18 | 1.25 | 1.62 | 1.25 | 0.33 | 1.5 | 1.39 | 1.56 | 0.33 | 10.51 | 1.45 |
| 1.42 | 1.2 | 1.35 | 1.2 | 1.17 | 0.97 | 1.17 | 1.04 | 1.17 | 1.73 | 1.11 |
| 1.22 | 0.93 | 1.12 | 0.93 | 0.87 | 0.97 | 1.09 | 2.42 | 0.87 | 0.98 | 1.08 |
| 2.02 | 0.8 | 0.94 | 0.8 | 0.51 | 1.89 | 4.81 | 0.43 | 0.51 | 2.26 | 1.06 |
| 1.08 | 1.17 | 0.79 | 1.17 | 1.04 | 1.07 | 1.28 | 1.56 | 1.04 | 0.78 | 1.05 |
| 0.79 | 0.9 | 1.55 | 0.9 | 1.07 | 0.89 | 0.8 | 1.01 | 1.07 | 1.09 | 0.99 |
| 1.13 | 0.99 | 1.22 | 0.99 | 1.28 | 0.53 | 1.05 | 0.73 | 1.28 | 1.08 | 0.84 |
| 0.99 | 1 | 0.93 | 1 | 1.31 | 1.06 | 0.94 | 0.81 | 1.31 | 1.73 | 0.97 |
| 1.23 | 0.79 | 0.99 | 0.79 | 1.07 | 1.07 | 0.87 | 0.91 | 1.07 | 1.13 | 1.08 |
| 1.15 | 0.89 | 1.32 | 0.89 | 1.03 | 0.81 | 0.99 | 0.97 | 1.03 | 0.95 | 0.96 |
| 1.63 | 1.07 | 1.48 | 1.07 | 0.83 | 0.88 | 1.3 | 0.69 | 0.83 | 1.18 | 0.97 |
| 1.38 | 0.73 | 0.41 | 0.73 | 0.62 | 1.37 | 1.59 | 0.42 | 0.62 | 1.31 | 1.14 |
| 1.12 | 1.39 | 2.25 | 1.39 | 0.77 | 1.47 | 0.79 | 0.88 | 0.77 | 0.99 | 1.15 |

| | | | | | | | | | | |
|---|---|---|---|---|---|---|---|---|---|---|
| 1.64 | 1.31 | 0.75 | 1.31 | 2.26 | 0.92 | 0.87 | 2.43 | 2.26 | 1.6 | 1.08 |
| 1.04 | 0.83 | 1 | 0.83 | 0.92 | 0.82 | 0.87 | 0.49 | 0.92 | 0.85 | 1.05 |
| 1.93 | 0.75 | 1.15 | 0.75 | 1.59 | 0.75 | 1.44 | 1.62 | 1.59 | 1.23 | 0.58 |
| 0.83 | 4.71 | 1.18 | 4.71 | 0.6 | 1.32 | 1 | 5.38 | 0.6 | 0.52 | 0.83 |
| 1.34 | 1.72 | 0.85 | 1.72 | 1.17 | 1.15 | 0.85 | 0.66 | 1.17 | 1.05 | 1.56 |
| 0.55 | 0.5 | 3.58 | 0.5 | 0.62 | 0.26 | 1.01 | 1.02 | 0.62 | 0.71 | 1.07 |
| 1.88 | 1.47 | 1.89 | 1.47 | 1.52 | 0.94 | 0.96 | 0.54 | 1.52 | 1.64 | 0.97 |
| 0.83 | 0.94 | 1.02 | 0.94 | 0.71 | 1.8 | 1.02 | 1.38 | 0.71 | 1.15 | 1.07 |
| 1.01 | 0.57 | 0.64 | 0.57 | 1.65 | 0.94 | 0.91 | 2.83 | 1.65 | 1.83 | 0.99 |
| 0.9 | 1.01 | 1.71 | 1.01 | 1.39 | 0.81 | 1.07 | 0.7 | 1.39 | 1.24 | 1.3 |
| 1.11 | 0.91 | 0.88 | 0.91 | 0.65 | 1.01 | 1.25 | 0.82 | 0.65 | 1.22 | 1.03 |
| 0.85 | 1.14 | 0.89 | 1.14 | 1.26 | 0.97 | 1.18 | 2.26 | 1.26 | 0.94 | 0.95 |
| 0.95 | 0.73 | 1.41 | 0.73 | 1.03 | 1.22 | 0.94 | 0.81 | 1.03 | 1.46 | 1.17 |
| 1.44 | 0.87 | 0.68 | 0.87 | 0.84 | 1.25 | 1.26 | 0.54 | 0.84 | 0.78 | 0.81 |
| 0.96 | 1.26 | 1.04 | 1.26 | 2.56 | 1.37 | 0.9 | 1.19 | 2.56 | 1.26 | 1.28 |
| 1.19 | 1.24 | 1.31 | 1.24 | 1.01 | 1.31 | 1.02 | 1.22 | 1.01 | 3.23 | 1 |
| 0.42 | 0.92 | 0.83 | 0.92 | 1.32 | 0.93 | 0.99 | 1.05 | 1.32 | 0.8 | 0.99 |
| 1.3 | 1.19 | 2.39 | 1.19 | 1.75 | 5.6 | 0.98 | 4.76 | 1.75 | 1.25 | 1.09 |
| 0.82 | 1.2 | 4.17 | 1.2 | 0.97 | 0.82 | 1.01 | 0.71 | 0.97 | 0.97 | 0.86 |
| 3.84 | 0.7 | 0.66 | 0.7 | 0.81 | 1.3 | 0.88 | 2.39 | 0.81 | 1.08 | 1.03 |
| 0.68 | 0.98 | 0.73 | 0.98 | 0.85 | 1.49 | 0.96 | 1.46 | 0.85 | 1.68 | 1.07 |
| 1.36 | 0.71 | 1 | 0.71 | 1.09 | 1.12 | 0.86 | 0.75 | 1.09 | 1.28 | 0.94 |
| 1 | 1.1 | 1.05 | 1.1 | 1.21 | 1.02 | 1.4 | 0.42 | 1.21 | 0.92 | 0.97 |
| 1.07 | 1.86 | 0.52 | 1.86 | 1.73 | 0.79 | 0.85 | 0.29 | 1.73 | 0.58 | 0.85 |
| 1.06 | 0.88 | 0.48 | 0.88 | 0.87 | 0.87 | 0.98 | 0.75 | 0.87 | 0.8 | 1.23 |
| 1.08 | 1.08 | 0.88 | 1.08 | 1.76 | 1.34 | 0.86 | 0.96 | 1.76 | 0.87 | 1.19 |
| 0.71 | 1.29 | 1.14 | 1.29 | 1.23 | 1.17 | 1.14 | 2.57 | 1.23 | 0.82 | 0.93 |
| 1.24 | 1.33 | 1.19 | 1.33 | 2.06 | 0.9 | 1.12 | 1.98 | 2.06 | 1.23 | 0.67 |
| 1.83 | 0.79 | 0.58 | 0.79 | 1.32 | 0.77 | 1.29 | 0.72 | 1.32 | 1.42 | 0.87 |
| 0.89 | 0.82 | 0.98 | 0.82 | 0.9 | 1.04 | 0.91 | 0.92 | 0.9 | 1.2 | 1.01 |
| 1.16 | 0.84 | 1.24 | 0.84 | 1.04 | 2.17 | 1.04 | 0.61 | 1.04 | 4.24 | 1.05 |
| 1.22 | 0.99 | 1.08 | 0.99 | 1.85 | 1.2 | 1.3 | 0.77 | 1.85 | 7.72 | 1.05 |
| 1.11 | 1.09 | 3.55 | 1.09 | 1.59 | 1.09 | 0.93 | 2.24 | 1.59 | 1.12 | 0.89 |

| | | | | | | | | | | |
|---|---|---|---|---|---|---|---|---|---|---|
| 0.96 | 0.89 | 0.72 | 0.89 | 1.3 | 1.12 | 1.15 | 1.1 | 1.3 | 1.58 | 1.09 |
| 0.71 | 0.82 | 1.75 | 0.82 | 1.04 | 1.05 | 0.8 | 6.08 | 1.04 | 0.99 | 0.77 |
| 0.84 | 1.09 | 0.78 | 1.09 | 1.12 | 1.15 | 1.03 | 2.45 | 1.12 | 1.73 | 0.72 |
| 0.92 | 0.91 | 3 | 0.91 | 1.08 | 0.97 | 0.87 | 0.97 | 1.08 | 1.04 | 1.1 |
| 0.97 | 0.98 | 0.53 | 0.98 | 0.97 | 0.9 | 0.82 | 0.87 | 0.97 | 0.67 | 0.92 |
| 1.03 | 0.9 | 0.93 | 0.9 | 1.1 | 1.18 | 1.13 | 0.89 | 1.1 | 0.98 | 0.87 |
| 0.82 | 1 | 0.96 | 1 | 1.19 | 1.08 | 0.96 | 0.89 | 1.19 | 1.02 | 0.85 |
| 0.78 | 0.92 | 0.67 | 0.92 | 0.84 | 1.77 | 0.87 | 1.33 | 0.84 | 0.83 | 1.24 |
| 0.7 | 1.23 | 1.02 | 1.23 | 0.65 | 1.05 | 0.92 | 0.73 | 0.65 | 0.98 | 0.84 |
| 0.78 | 1.06 | 1.89 | 1.06 | 0.84 | 1.35 | 0.79 | 1 | 0.84 | 0.84 | 1.38 |
| 0.68 | 1.14 | 1.21 | 1.14 | 1.09 | 0.95 | 0.94 | 0.8 | 1.09 | 0.53 | 1.03 |
| 0.73 | 1.06 | 1.52 | 1.06 | 1.15 | 1.06 | 0.9 | 2.38 | 1.15 | 1.02 | 1.17 |
| 1.5 | 1.07 | 1.46 | 1.07 | 1.6 | 6.91 | 0.86 | 5.15 | 1.6 | 1.89 | 1.01 |
| 0.73 | 1.09 | 1.73 | 1.09 | 0.93 | 1.02 | 0.9 | 0.87 | 0.93 | 0.79 | 0.71 |
| 0.69 | 1.38 | 1.3 | 1.38 | 0.41 | 1.16 | 0.49 | 1.74 | 0.41 | 3.43 | 1.33 |
| 1.29 | 1.1 | 0.68 | 1.1 | 0.87 | 1.08 | 1.32 | 1.3 | 0.87 | 1.08 | 1.22 |
| 1.35 | 0.91 | 0.87 | 0.91 | 0.92 | 1.1 | 0.86 | 2.15 | 0.92 | 1.1 | 0.89 |
| 0.79 | 0.9 | 1.29 | 0.9 | 2.9 | 0.81 | 1.06 | 0.79 | 2.9 | 2.1 | 1.12 |
| 1.02 | 0.96 | 1.01 | 0.96 | 1.03 | 0.98 | 0.51 | 0.96 | 1.03 | 0.91 | 1.24 |
| 0.91 | 0.84 | 0.57 | 0.84 | 0.77 | 1.22 | 0.97 | 2.2 | 0.77 | 0.7 | 0.9 |
| 0.86 | 1.04 | 1.31 | 1.04 | 0.49 | 0.92 | 1.05 | 1.58 | 0.49 | 0.65 | 1.03 |
| 1.24 | 1.21 | 1.19 | 1.21 | 0.46 | 0.79 | 0.7 | Inf | 0.46 | 2.18 | 1.38 |
| 1.02 | 1.06 | 0.86 | 1.06 | 0.85 | 0.95 | 1.31 | 1.16 | 0.85 | 6.42 | 1.02 |
| 1.06 | 0.94 | 0.69 | 0.94 | 0.68 | 1.08 | 0.8 | 0.95 | 0.68 | 0.99 | 0.76 |
| 0.85 | 1.38 | 1.04 | 1.38 | 0.94 | 1.23 | 0.98 | 0.97 | 0.94 | 1.09 | 1.14 |
| 1.17 | 1.15 | 1.19 | 1.15 | 0.89 | 1.41 | 0.94 | 0.66 | 0.89 | 0.9 | 0.61 |
| 0.7 | 1.29 | 3.06 | 1.29 | 0.88 | 1.11 | 1.52 | 0.69 | 0.88 | 0.94 | 1.15 |
| 1.27 | 0.82 | 0.94 | 0.82 | 1.4 | 0.89 | 0.89 | 3.08 | 1.4 | 1.16 | 1.09 |
| 0.95 | 1.07 | 0.81 | 1.07 | 0.67 | 1.05 | 0.9 | 0.96 | 0.67 | 1.12 | 0.94 |
| 0.78 | 1.19 | 2 | 1.19 | 0.93 | 0.92 | 0.79 | 1.05 | 0.93 | 0.85 | 1.06 |
| 0.8 | 0.81 | 0.78 | 0.81 | 0.72 | 1.07 | 1.05 | 0.91 | 0.72 | 9.13 | 0.98 |
| 1.26 | 1.01 | 1.66 | 1.01 | 1.01 | 1.04 | 0.92 | 0.86 | 1.01 | 1.2 | 1.05 |
| 1.06 | 1.1 | 1.06 | 1.1 | 0.94 | 1.06 | 1.09 | 1.06 | 0.94 | 1.12 | 1.05 |

| | | | | | | | | | | |
|---|---|---|---|---|---|---|---|---|---|---|
| 1.74 | 6.69 | 1.15 | 6.69 | 0.81 | 0.7 | 0.95 | 2.89 | 0.81 | 0.92 | 1.05 |
| 1.04 | 0.82 | 0.74 | 0.82 | 0.89 | 1.13 | 1.17 | 0.99 | 0.89 | 3.32 | 1.05 |
| 1.15 | 1.01 | 1.04 | 1.01 | 0.96 | 1.01 | 0.81 | 0.85 | 0.96 | 1.01 | 0.92 |
| 0.82 | 1.19 | 1.07 | 1.19 | 0.77 | 1.76 | 0.69 | 1.04 | 0.77 | 0.67 | 1.48 |
| 0.86 | 0.51 | 1.3 | 0.51 | 0.68 | 1.11 | 0.62 | 1.69 | 0.68 | 1.11 | 0.89 |
| 1.01 | 0.99 | 1.19 | 0.99 | 0.92 | 1.1 | 1.08 | 0.82 | 0.92 | 1.05 | 0.95 |
| 0.95 | 0.7 | 0.66 | 0.7 | 1.4 | 0.75 | 1 | 0.7 | 1.4 | 5.23 | 1.02 |
| 1.22 | 0.93 | 0.87 | 0.93 | 2.13 | 1.09 | 0.78 | 1.29 | 2.13 | 1.07 | 0.87 |
| 1 | 0.94 | 1.45 | 0.94 | 0.71 | 1.1 | 1.17 | 0.91 | 0.71 | 1.32 | 1.15 |
| 0.95 | 0.68 | 0.62 | 0.68 | 1.63 | 0.76 | 0.93 | 0.52 | 1.63 | 3 | 1.11 |
| 0.55 | 0.73 | 1.18 | 0.73 | 0.9 | 1.96 | 0.87 | 0.95 | 0.9 | 1.58 | 1.06 |
| 0.64 | 0.83 | 0.9 | 0.83 | 0.93 | 0.96 | 1.69 | 0.65 | 0.93 | 0.87 | 1.27 |
| 1.05 | 0.92 | 0.87 | 0.92 | 0.98 | 1.11 | 0.6 | 0.91 | 0.98 | 0.91 | 0.98 |
| 0.89 | 0.91 | 0.92 | 0.91 | 0.92 | 1 | 0.9 | 0.93 | 0.92 | 2.86 | 1.01 |
| 0.99 | 0.92 | 3.13 | 0.92 | 0.97 | 0.91 | 0.91 | 1.07 | 0.97 | 1.02 | 0.9 |
| 1.08 | 1.06 | 1.35 | 1.06 | 1.52 | 1.14 | 1.18 | 0.92 | 1.52 | 0.76 | 0.94 |
| 1.08 | 1.19 | 0.96 | 1.19 | 0.86 | 0.92 | 1.07 | 0.92 | 0.86 | 1.18 | 1.07 |
| 0.88 | 0.78 | 1.89 | 0.78 | 0.97 | 1 | 0.98 | 0.91 | 0.97 | 0.64 | 0.95 |
| 0.76 | 1.21 | 0.83 | 1.21 | 1.24 | 1.31 | 0.72 | 1.12 | 1.24 | 3.68 | 0.8 |
| 0.63 | 1.12 | 0.77 | 1.12 | 0.99 | 1.22 | 0.87 | 1.18 | 0.99 | 1.32 | 0.82 |
| 1.27 | 1 | 1.13 | 1 | 1.01 | 0.57 | 1.16 | 1.64 | 1.01 | 0.93 | 1.12 |
| 0.67 | 0.57 | 3 | 0.57 | 1.33 | 8.36 | 3.22 | 0.35 | 1.33 | 1.94 | 0.71 |
| 0.65 | 0.96 | 0.91 | 0.96 | 1.34 | 0.99 | 0.99 | 0.76 | 1.34 | 2.21 | 0.95 |
| 1.84 | 3.2 | 1 | 3.2 | 0.89 | 0.77 | 1.27 | 0.52 | 0.89 | 0.95 | 0.87 |
| 1.04 | 1.13 | 0.98 | 1.13 | 1.27 | 0.83 | 1.05 | 0.88 | 1.27 | 3.03 | 0.95 |
| 0.85 | 1.07 | 0.21 | 1.07 | 0.27 | 1.62 | 0.88 | 2.52 | 0.27 | 0.08 | 0.86 |
| 1.05 | 0.87 | 1.11 | 0.87 | 0.84 | 1.23 | 1.07 | 1.21 | 0.84 | 1.16 | 1.1 |
| 1.06 | 0.95 | 1.06 | 0.95 | 0.93 | 0.88 | 1.07 | 0.88 | 0.93 | 1.09 | 1.07 |
| 1.02 | 1.12 | 0.97 | 1.12 | 0.93 | 1.1 | 0.88 | 1.01 | 0.93 | 0.98 | 0.93 |
| 1.05 | 0.84 | 0.92 | 0.84 | 0.86 | 0.92 | 0.89 | 0.93 | 0.86 | 0.97 | 1.05 |
| 1.27 | 1.16 | 0.86 | 1.16 | 1.49 | 0.82 | 1.41 | 17.28 | 1.49 | 0.85 | 0.75 |
| 1.28 | 0.84 | 1.07 | 0.84 | 0.88 | 0.92 | 1.08 | 1.58 | 0.88 | 0.8 | 1.08 |
| 0.89 | 1 | 1.1 | 1 | 0.75 | 0.87 | 1.26 | 2.91 | 0.75 | 2.32 | 0.93 |

| | | | | | | | | | | |
|---|---|---|---|---|---|---|---|---|---|---|
| 0.78 | 1.18 | 1.53 | 1.18 | 0.74 | 1.36 | 1.73 | 1.18 | 0.74 | 1.39 | 0.99 |
| 0.95 | 0.94 | 0.52 | 0.94 | 1.34 | 1.42 | 0.88 | 0.51 | 1.34 | 0.83 | 0.81 |
| 0.88 | 0.83 | 0.98 | 0.83 | 0.78 | 0.81 | 0.89 | Inf | 0.78 | 1.02 | 1.25 |
| 1.39 | 0.62 | 0.54 | 0.62 | 0.87 | 0.73 | 0.59 | 0.98 | 0.87 | 0.5 | 0.85 |
| 0.94 | 0.93 | 0.73 | 0.93 | 0.93 | 1.2 | 1.05 | 1.25 | 0.93 | 2.06 | 0.91 |
| 1.06 | 0.94 | 1.08 | 0.94 | 0.95 | 1.16 | 0.78 | 1.2 | 0.95 | 5.33 | 0.99 |
| 1.95 | 0.99 | 0.48 | 0.99 | 0.8 | 0.96 | 0.87 | 3.79 | 0.8 | 0.91 | 1.27 |
| 1.13 | 0.8 | 0.85 | 0.8 | 0.78 | 1.07 | 0.95 | 0.98 | 0.78 | 1.37 | 0.99 |
| 2.43 | 3.39 | 1.46 | 3.39 | 0.83 | 0.65 | 1 | 0.69 | 0.83 | 1.2 | 1.06 |
| 0.78 | 0.94 | 1.23 | 0.94 | 0.41 | 1.41 | 0.79 | 4.19 | 0.41 | 1.11 | 1.29 |
| 0.78 | 0.77 | 1.13 | 0.77 | 1.1 | 1.03 | 0.75 | 0.82 | 1.1 | 0.84 | 1.03 |
| 0.91 | 0.83 | 0.77 | 0.83 | 0.89 | 0.94 | 0.9 | 0.82 | 0.89 | 0.8 | 1.05 |
| 1.26 | 0.76 | 3.55 | 0.76 | 1.17 | 0.98 | 1.01 | 0.78 | 1.17 | 1.48 | 1.16 |
| 1.14 | 0.96 | 1.11 | 0.96 | 0.75 | 5.5 | 1.1 | 0.84 | 0.75 | 3.11 | 1.06 |
| 0.89 | 0.91 | 1.2 | 0.91 | 0.81 | 0.74 | 0.88 | 0.84 | 0.81 | 2.14 | 1.08 |
| 1.18 | 0.67 | 0.56 | 0.67 | 0.44 | 1.18 | 0.71 | 0.38 | 0.44 | 1.35 | 0.83 |
| 1.26 | 0.74 | 0.82 | 0.74 | 0.98 | 1.09 | 0.72 | 0.69 | 0.98 | 1.4 | 1.06 |
| 1.03 | 0.84 | 0.78 | 0.84 | 0.68 | 0.93 | 0.94 | 0.9 | 0.68 | 5.3 | 0.97 |
| 1.05 | 2.21 | 1.21 | 2.21 | 42.28 | 0.51 | 1.03 | 0.88 | 42.28 | 0.68 | 0.62 |
| 1.02 | 0.76 | 1.07 | 0.76 | 0.35 | 1.06 | 1.11 | 24.94 | 0.35 | 2.07 | 0.67 |
| 0.88 | 1.33 | 0.83 | 1.33 | 1.71 | 0.97 | 0.69 | 0.53 | 1.71 | 0.98 | 0.75 |
| 0.93 | 0.68 | 0.64 | 0.68 | 1.49 | 0.81 | 0.92 | 0.54 | 1.49 | 2.58 | 1.14 |
| 2.73 | 0.93 | 0.37 | 0.93 | 1.32 | 0.72 | 1.62 | 0.46 | 1.32 | 2.31 | 0.49 |
| 2.27 | 0.84 | 0.35 | 0.84 | 1.93 | 1.09 | 1.12 | 1.95 | 1.93 | 1.56 | 1.02 |
| 1.22 | 1.24 | 1.23 | 1.24 | 0.8 | 1 | 0.77 | 1.54 | 0.8 | 0.87 | 1.07 |
| 1.22 | 1.33 | 1.15 | 1.33 | 0.72 | 1.12 | 1.14 | 1.04 | 0.72 | 1.53 | 0.93 |
| 0.68 | 0.82 | 0.36 | 0.82 | 1.1 | 1.64 | 1.19 | 0.82 | 1.1 | 1.59 | 0.95 |
| 0.78 | 1.14 | 1.34 | 1.14 | 0.68 | 1.18 | 2.2 | 3.16 | 0.68 | 0.66 | 0.82 |
| 1.89 | 0.75 | 2.01 | 0.75 | 0.72 | 1.19 | 1.15 | 0.63 | 0.72 | 1.43 | 1.05 |
| 0.66 | 1.99 | 0.26 | 1.99 | 0.27 | 1.44 | 0.79 | 1.44 | 0.27 | 2.53 | 0.85 |
| 1.28 | 1.27 | 0.87 | 1.27 | 0.64 | 0.95 | 0.84 | 1.24 | 0.64 | 0.74 | 0.74 |
| 1.13 | 0.94 | 1.68 | 0.94 | 0.11 | 1.72 | 0.93 | 2.12 | 0.11 | 0.97 | 1.33 |
| 1.94 | 1.4 | 1.19 | 1.4 | 1.44 | 1.75 | 0.97 | 0.41 | 1.44 | 5.32 | 1.06 |

| | | | | | | | | | | |
|---|---|---|---|---|---|---|---|---|---|---|
| 0.96 | 1.07 | 0.88 | 1.07 | 0.86 | 1.01 | 0.93 | 0.94 | 0.86 | 1.22 | 1.05 |
| 1.14 | 1.06 | 1.21 | 1.06 | 1.17 | 0.98 | 1.17 | 1.15 | 1.17 | 1.06 | 1.1 |
| 0.39 | 2.45 | 0.73 | 2.45 | 1.02 | 7 | 0.49 | 0.08 | 1.02 | 1.23 | 6.31 |
| 1.04 | 0.59 | 0.92 | 0.59 | 0.45 | 1.18 | 0.94 | 0.56 | 0.45 | 1.03 | 0.75 |
| 1.01 | 0.73 | 0.84 | 0.73 | 0.82 | 0.87 | 0.89 | 2.53 | 0.82 | 4.08 | 1.07 |
| 1.3 | 1.28 | 1.05 | 1.28 | 1.32 | 0.97 | 1.05 | 1.27 | 1.32 | 1.6 | 1.1 |
| 1.27 | 1.25 | 1.14 | 1.25 | 0.89 | 1.19 | 1.13 | 0.84 | 0.89 | 1.18 | 1.03 |
| 0.95 | 1.07 | 0.8 | 1.07 | 1.09 | 1.05 | 0.96 | 0.58 | 1.09 | 0.87 | 0.96 |
| 1.36 | 1.24 | 1.33 | 1.24 | 0.78 | 1.37 | 0.69 | 2.63 | 0.78 | 0.72 | 0.92 |
| 1.06 | 1.08 | 1.19 | 1.08 | 0.57 | 1.74 | 0.87 | 1.13 | 0.57 | 0.64 | 0.79 |
| 0.85 | 1.25 | 1.14 | 1.25 | 0.87 | 1.08 | 1.07 | 1.53 | 0.87 | 0.94 | 1.07 |
| 3.65 | 1.69 | 2.33 | 1.69 | 14.57 | 30.87 | 0.57 | 6.27 | 14.57 | 35.96 | 79.4 |
| 1.29 | 1.35 | 0.29 | 1.35 | 0.89 | 1 | 1.4 | 0.86 | 0.89 | 1.64 | 1 |
| 1 | 1.16 | 0.76 | 1.16 | 0.76 | 0.73 | 0.72 | 1.3 | 0.76 | 1.02 | 0.84 |

| 200632_s_at | 202315_s_at | 216836_s_at | 204369_at | 219388_at | 200958_s_at | 209360_s_at | 213139_at | 209863_s_at | 201746_at | 219528_s_at |
|---|---|---|---|---|---|---|---|---|---|---|
| 3.09 | 1.06 | 1.12 | 1.05 | 1.78 | 0.92 | 0.89 | 0.6 | 1.16 | 0.92 | 0.86 |
| 1.76 | 1.38 | 0.93 | 1.48 | 1.05 | 1.51 | 0.51 | 0.48 | 0.85 | 0.88 | 0.54 |
| 0.87 | 1.01 | 1.06 | 1.85 | 0.97 | 1.03 | 1.03 | 2.23 | 0.55 | 0.94 | 0.92 |
| 1.07 | 1.36 | 0.95 | 0.99 | 1.07 | 1.1 | 1.19 | 4.27 | 1.12 | 1.02 | 1.17 |
| 1.22 | 0.75 | 0.7 | 1.19 | 0.58 | 1.07 | 1.29 | 0.63 | 4.7 | 0.89 | 2.28 |
| 0.83 | 6.11 | 1.19 | 1.44 | 1.65 | 1.09 | 0.8 | 2.82 | 6.25 | 0.95 | 1.22 |
| 0.69 | 0.88 | 0.48 | 0.96 | 0.11 | 1.76 | 0.63 | 1.12 | 0.67 | 0.14 | 0.53 |
| 0.7 | 1.03 | 1.01 | 1.09 | 0.7 | 1.16 | 0.87 | 0.67 | 0.6 | 1.36 | 0.54 |
| 1.03 | 0.5 | 0.79 | 1.35 | 1.43 | 1.39 | 0.72 | 0.45 | 2.39 | 0.82 | 0.88 |
| 0.52 | 0.71 | 0.88 | 0.64 | 0.69 | 1.05 | 0.68 | 3.08 | 1.81 | 1.42 | 0.46 |
| 0.91 | 0.8 | 0.96 | 1.03 | 0.16 | 0.91 | 1.09 | 1.35 | 0.99 | 0.68 | 0.98 |
| 1.24 | 1.24 | 0.76 | 0.98 | 1.79 | 0.97 | 0.82 | 0.54 | 1.08 | 0.93 | 1.4 |
| 0.46 | 1.31 | 0.95 | 1.09 | 0.95 | 0.99 | 0.77 | 0.59 | 0.91 | 1.26 | 1.48 |
| 0.12 | 0.71 | 1.11 | 2.73 | 1.02 | 0.46 | 0.71 | 3.74 | 8.89 | 0.19 | 2.68 |
| 2.16 | 1.08 | 0.97 | 1.42 | 0.8 | 1.25 | 1.26 | 1.71 | 0.85 | 1.11 | 1 |
| 0.65 | 0.69 | 0.93 | 0.75 | 0.99 | 1.07 | 0.8 | 5.13 | 1.42 | 1.33 | 1.51 |
| 1 | 1.36 | 1.03 | 0.96 | 3.63 | 1.06 | 0.88 | 0.79 | 1.69 | 0.99 | 1.28 |
| 5.32 | 1.18 | 0.76 | 1.03 | 0.88 | 1.16 | 1.39 | 1.47 | 1.43 | 1.13 | 1.13 |
| 2.7 | 1.5 | 0.64 | 1.19 | 0.97 | 0.9 | 1.2 | 0.79 | 3.85 | 0.1 | 2.01 |
| 0.82 | 1.97 | 2.25 | 0.16 | 0.73 | 0.87 | 8.37 | 0.41 | 3.05 | 0.96 | 1.94 |
| 0.97 | 1.19 | 1.08 | 0.47 | 4.39 | 0.93 | 0.8 | 3.75 | 1.63 | 1.87 | 1.29 |
| 8.3 | 1.9 | 1.39 | 1.16 | 0.94 | 0.87 | 1.45 | 1.43 | 33.05 | 3.15 | 5.07 |
| 1.38 | 1.03 | 1.17 | 0.83 | 0.92 | 1.03 | 1.1 | 1.05 | 0.16 | 1.32 | 1.75 |
| 0.07 | 0.57 | 1.23 | 1.08 | 0.06 | 1.2 | 1.03 | 0.3 | 0.01 | 0.95 | 0.03 |
| 1.13 | 0.5 | 1.2 | 0.69 | 4.07 | 0.73 | 1.89 | 24.58 | 2.92 | 0.63 | 0.13 |
| 0.63 | 1.22 | 1.05 | 0.56 | 0.85 | 0.7 | 0.94 | 0.53 | 1.1 | 1.11 | 0.63 |
| 0.67 | 1.17 | 0.43 | 1.22 | 1.3 | 0.97 | 0.9 | 0.82 | 0.73 | 1.93 | 1.07 |
| 1.47 | 4.53 | 0.97 | 1.54 | 5.84 | 2.33 | 1.03 | 2.22 | 19.68 | 6.62 | 2.73 |
| 9.12 | 1.8 | 3.04 | 1 | 1.77 | 1.24 | 0.4 | 3.69 | 1.43 | 0.15 | 0.73 |
| 1.2 | 1.02 | 0.95 | 1.12 | 2.4 | 1.08 | 1.03 | 1.6 | 4.47 | 0.73 | 1.32 |

| | | | | | | | | | | |
|---|---|---|---|---|---|---|---|---|---|---|
| 0.74 | 0.68 | 0.44 | 1.05 | 1.5 | 0.8 | 0.79 | 1.79 | 0.89 | 0.56 | 2.26 |
| 0.23 | 1.27 | 1.28 | 0.43 | 0.15 | 2.69 | 0.53 | 9.91 | 1.5 | 1.38 | 0.17 |
| 0.96 | 1.11 | 1.01 | 1.13 | 1.16 | 1.03 | 1 | 2.37 | 1.05 | 1.07 | 1.27 |
| 0.48 | 1.18 | 0.91 | 0.55 | 2.26 | 0.85 | 0.31 | 0.39 | 0.27 | 1.27 | 0.31 |
| 0.88 | 1.23 | 1.26 | 1.85 | 0.65 | 5.04 | 0.71 | 0.35 | 1.21 | 16 | 0.31 |
| 0.83 | 1.55 | 1.45 | 0.51 | 4.2 | 1.47 | 2.58 | 0.83 | 1.58 | 1.65 | 18.81 |
| 2.96 | 1.07 | 0.53 | 0.85 | 1.96 | 2.84 | 0.32 | 1.32 | 0.83 | 0.57 | 3.37 |
| 0.83 | 1.18 | 0.8 | 1 | 2.29 | 0.93 | 1.05 | 0.9 | 1.18 | 0.88 | 1.33 |
| 0.24 | 1.06 | 0.98 | 1.03 | 0.96 | 1.1 | 0.82 | 0.83 | 0.81 | 1.24 | 0.32 |
| 1.5 | 1.07 | 0.62 | 1.65 | 0.03 | 2.2 | 2.14 | 31.56 | 0.18 | 1.01 | 0.04 |
| 1.29 | 1.17 | 1.34 | 0.95 | 0.83 | 0.98 | 2.8 | 0.63 | 2.77 | 2.01 | 1.11 |
| 0.99 | 1.08 | 0.88 | 1 | 1.18 | 0.94 | 0.78 | 1.14 | 3.47 | 0.95 | 1.2 |
| 1.18 | 1.15 | 0.95 | 1.32 | 1 | 1.36 | 1.59 | 0.61 | 0.35 | 0.26 | 1.07 |
| 0.49 | 1.71 | 0.6 | 1.43 | 0.92 | 1.76 | 2.86 | 1.13 | 0.58 | 2.55 | 1.69 |
| 1.16 | 0.99 | 0.84 | 1.19 | 0.95 | 1.17 | 1.53 | 0.69 | 0.56 | 0.41 | 1.12 |
| 3.5 | 1.91 | 0.99 | 1.23 | 5.09 | 1.28 | 2.83 | 4.09 | 7.81 | 1.09 | 1.42 |
| 1.41 | 0.94 | 1.18 | 0.94 | 1.08 | 0.79 | 1.08 | 1.06 | 1.03 | 0.97 | 1.5 |
| 0.8 | 1.17 | 1.09 | 1.18 | 1.07 | 0.98 | 1.36 | 2.72 | 10.7 | 0.81 | 3.15 |
| 0.93 | 0.97 | 1.15 | 1.07 | 0.95 | 0.98 | 1.05 | 1.13 | 5.14 | 0.86 | 0.89 |
| 8.87 | 1.18 | 1.01 | 0.95 | 7.24 | 1.23 | 0.77 | 1.79 | 4.32 | 0.69 | 1.69 |
| 0.51 | 0.79 | 0.97 | 1.1 | 1.09 | 0.83 | 0.74 | 0.97 | 2.17 | 0.96 | 0.5 |
| 0.84 | 0.92 | 0.72 | 0.83 | 0.91 | 0.76 | 1.21 | 0.68 | 0.34 | 1.05 | 1.07 |
| 0.53 | 0.74 | 1.23 | 1.01 | 1.03 | 0.92 | 1.09 | 1.86 | 1.03 | 1.09 | 1.38 |
| 4.03 | 1.36 | 0.88 | 1.22 | 1.53 | 1.34 | 4.23 | 7.72 | 8.03 | 0.81 | 0.96 |
| 0.94 | 1.61 | 0.35 | 1.03 | 0.52 | 1.35 | 0.58 | 0.88 | 3.21 | 0.35 | 4.16 |
| 0.69 | 1.35 | 0.87 | 1.01 | 0.88 | 0.88 | 0.57 | 0.52 | 0.1 | 1.48 | 0.76 |
| 0.52 | 2.33 | 1.16 | 0.5 | 4.46 | 0.94 | 1.32 | 0.46 | 1.23 | 0.93 | 4.98 |
| 1.66 | 1.04 | 1.97 | 1.99 | 1.01 | 0.89 | 2.78 | 0.01 | 1.43 | 1.99 | 1.61 |
| 2.03 | 1.07 | 1.55 | 1.09 | 1.02 | 1.07 | 1.1 | 0.83 | 1.14 | 0.25 | 0.71 |
| 0.99 | 1.36 | 1.1 | 1.09 | 1.07 | 1.19 | 0.91 | 0.45 | 0.4 | 0.41 | 0.66 |
| 1.7 | 1.27 | 0.75 | 0.91 | 0.77 | 1.46 | 3.68 | 1.67 | 2.69 | 0.73 | 0.76 |
| 1.21 | 1.23 | 1.86 | 0.83 | 1.05 | 0.78 | 1.32 | 2.36 | 1.38 | 0.79 | 0.45 |
| 1.07 | 1.16 | 0.68 | 1.24 | 1.56 | 1.02 | 2.64 | 2.03 | 1.25 | 1.14 | 1 |

| | | | | | | | | | | |
|---|---|---|---|---|---|---|---|---|---|---|
| 2.1 | 0.74 | 1.26 | 0.89 | 1.77 | 1.33 | 1.05 | 24.09 | 0.71 | 0.91 | 0.74 |
| 0.88 | 1.17 | 1.43 | 1.02 | 0.79 | 0.8 | 0.94 | 0.91 | 7.16 | 1.49 | 21.22 |
| 2.37 | 1.03 | 0.95 | 1.37 | 2.72 | 1.08 | 0.89 | 3.25 | 3.92 | 0.89 | 0.65 |
| 2.16 | 1.19 | 5.95 | 1.16 | 3.78 | 1.27 | 2.76 | 9.22 | 1.7 | 0.87 | 1.23 |
| 1 | 1.01 | 1.04 | 1.15 | 9.16 | 0.98 | 1.17 | 1.19 | 2.75 | 0.85 | 0.78 |
| 1.24 | 0.81 | 0.85 | 0.97 | 0.78 | 1.11 | 0.9 | 1.13 | 1.07 | 1.35 | 0.78 |
| 0.38 | 1.67 | 2.02 | 1.15 | 1.08 | 0.19 | 0.65 | 0.12 | 3.44 | 1.65 | 2.59 |
| 4.27 | 1.87 | 1.41 | 0.99 | 0.56 | 1.1 | 0.92 | 2.54 | 0.21 | 0.98 | 0.82 |
| 2.31 | 1.91 | 0.82 | 0.77 | 1.05 | 1.08 | 3.41 | 0.99 | 0.69 | 0.92 | 2.66 |
| 0.7 | 0.89 | 0.84 | 1.67 | 1.67 | 1.3 | 0.92 | 0.86 | 0.8 | 1.12 | 1.04 |
| 1.29 | 9.29 | 8.81 | 1.55 | 1.38 | 0.97 | 0.91 | 2.33 | 2.12 | 1.34 | 0.98 |
| 3.42 | 0.86 | 0.71 | 1.11 | 1 | 1.22 | 11.73 | 5.46 | 0.07 | 0.9 | 1.32 |
| 0.8 | 0.33 | 1.15 | 0.97 | 2.58 | 1.27 | 1.23 | 3.56 | 0.8 | 1.19 | 1.21 |
| 0.94 | 1.76 | 0.9 | 1.57 | 3.32 | 1.02 | 1.45 | 0.42 | 3.79 | 1.01 | 2.54 |
| 0.68 | 1.08 | 0.67 | 1.58 | 0.6 | 1.05 | 1.03 | 0.5 | 0.44 | 0.42 | 0.2 |
| 1.51 | 0.33 | 0.22 | 6.5 | 0.09 | 27.08 | 2.12 | 42.98 | 0.13 | 4.36 | 0.07 |
| 2.62 | 0.85 | 1.99 | 1.11 | 0.96 | 1.17 | 1.45 | 1 | 0.73 | 1.36 | 2.74 |
| 0.63 | 1.02 | 0.68 | 0.76 | 0.76 | 1.83 | 1.2 | 1.36 | 0.45 | 0.32 | 0.52 |
| 1.41 | 0.88 | 1.2 | 1.13 | 1.49 | 1.56 | 0.78 | 1.27 | 1.22 | 0.9 | 1.17 |
| 1.01 | 1.11 | 2.1 | 1.31 | 1.8 | 1.03 | 0.65 | 5.05 | 1.67 | 0.9 | 6.91 |
| 11.62 | 2.15 | 1.82 | 0.69 | 1.89 | 1.01 | 8.44 | 0.51 | 0.91 | 0.72 | 0.54 |
| 2.62 | 1.01 | 1.7 | 1.08 | 1.27 | 1.11 | 6.51 | 1.03 | 1.23 | 0.91 | 0.98 |
| 0.86 | 0.96 | 0.61 | 0.96 | 0.56 | 1.75 | 0.57 | 0.87 | 0.71 | 0.11 | 0.39 |
| 1.25 | 1.42 | 0.85 | 0.69 | 0.94 | 0.98 | 0.46 | 0.54 | 0.63 | 0.97 | 1.54 |
| 3.59 | 0.84 | 1.08 | 1.16 | 1.06 | 1.01 | 1.02 | 0.73 | 1.02 | 1.37 | 1.86 |
| 1.47 | 0.91 | 0.87 | 0.75 | 0.64 | 1.06 | 1 | 0.82 | 0.54 | 0.91 | 0.77 |
| 1.04 | 1.07 | 1.04 | 0.63 | 3.6 | 0.76 | 0.78 | 0.77 | 0.35 | 1.03 | 0.92 |
| 0.92 | 1.25 | 1.17 | 1.02 | 1.02 | 0.9 | 0.84 | 1.13 | 1.31 | 1.09 | 0.98 |
| 0.9 | 0.79 | 2.16 | 1.11 | 1.01 | 1.21 | 0.93 | 0.34 | 0.94 | 1.05 | 1.67 |
| 0.92 | 0.99 | 1.04 | 0.93 | 0.68 | 0.9 | 1 | 1.36 | 4.3 | 0.94 | 1.38 |
| 1.08 | 0.98 | 0.92 | 0.96 | 3.08 | 0.95 | 1.38 | 1.13 | 1.33 | 1.22 | 1.41 |
| 0.82 | 3.06 | 1.17 | 0.92 | 1.02 | 1.42 | 2.6 | 1.31 | 5.53 | 0.56 | 0.51 |
| 1.8 | 0.98 | 1.52 | 1.26 | 0.1 | 1.15 | 0.79 | 2.88 | 20.43 | 1.08 | 0.95 |

| | | | | | | | | | | |
|---|---|---|---|---|---|---|---|---|---|---|
| 0.86 | 0.83 | 1.06 | 0.99 | 1.22 | 0.84 | 1.03 | 0.66 | 1.53 | 0.94 | 1.49 |
| 1.02 | 1.26 | 2.03 | 0.68 | 1.08 | 0.96 | 0.74 | 1.22 | 4.81 | 0.86 | 0.86 |
| 0.8 | 1.17 | 1.11 | 0.97 | 0.82 | 0.97 | 1.1 | 2.66 | 0.58 | 1.42 | 1.23 |
| 1.39 | 1.12 | 0.54 | 0.87 | 1.28 | 1.39 | 3.53 | 1.83 | 1.38 | 0.96 | 3.55 |
| 0.78 | 0.97 | 1.1 | 1.1 | 0.53 | 1.79 | 1.28 | 1.75 | 1.29 | 1 | 1.55 |
| 0.46 | 1.01 | 0.33 | 0.29 | 0.43 | 1.44 | 0.34 | 3.24 | 1.17 | 0.73 | 0.4 |
| 0.91 | 1.05 | 0.95 | 1.1 | 1.83 | 1.03 | 0.86 | 1.45 | 1.32 | 1.13 | 1.12 |
| 1.17 | 1.14 | 0.83 | 0.71 | 0.8 | 1.07 | 1.23 | 1.16 | 0.44 | 1.12 | 1.23 |
| 2.16 | 0.65 | 0.63 | 1.35 | 0.68 | 1.9 | 0.79 | 1.29 | 0.98 | 0.75 | 1 |
| 1.53 | 1.26 | 1.08 | 1.2 | 0.9 | 1.84 | 1.04 | 4.37 | 3.09 | 1.33 | 0.63 |
| 0.94 | 0.94 | 1.05 | 0.93 | 2.11 | 0.91 | 1.18 | 0.68 | 0.46 | 1.43 | 0.78 |
| 2.22 | 0.98 | 1.02 | 0.97 | 0.86 | 0.82 | 1.25 | 18.48 | 1.29 | 1.05 | 1.6 |
| 1.65 | 1.06 | 0.92 | 0.86 | 1.42 | 0.96 | 0.71 | 0.96 | 3.82 | 0.79 | 2.97 |
| 0.78 | 1.22 | 0.86 | 0.71 | 1.09 | 0.9 | 0.53 | 0.68 | 0.52 | 1.16 | 0.47 |
| 2.66 | 1.05 | 1.1 | 1.19 | 0.58 | 1.21 | 0.55 | 1.81 | 0.72 | 1.08 | 1.35 |
| 1.16 | 1.78 | 2.6 | 1.01 | 3.19 | 0.86 | 1.3 | 2.83 | 1.07 | 1.14 | 0.81 |
| 2.37 | 0.9 | 1.68 | 0.87 | 1.99 | 0.74 | 1.1 | 2.35 | 1.66 | 1.26 | 1.08 |
| 1.83 | 1.48 | 5.28 | 1.21 | 3.63 | 1.09 | 3.21 | 9.33 | 1.43 | 0.91 | 0.65 |
| 2.25 | 1 | 1.48 | 1.05 | 0.5 | 0.94 | 0.85 | 0.53 | 1.98 | 1.14 | 1.17 |
| 3.75 | 0.74 | 0.83 | 0.89 | 1.24 | 0.85 | 1.06 | 1.32 | 0.94 | 0.7 | 0.97 |
| 0.49 | 1.19 | 0.62 | 1.06 | 0.78 | 1.42 | 0.84 | 1.26 | 0.73 | 0.7 | 0.62 |
| 0.89 | 0.81 | 2.19 | 1.25 | 0.64 | 0.88 | 1.13 | 0.74 | 0.43 | 1.09 | 0.97 |
| 1.65 | 1.04 | 1.02 | 0.92 | 1.18 | 0.89 | 1.39 | 0.79 | 0.96 | 1.03 | 1.29 |
| 0.58 | 0.85 | 1.74 | 1.1 | 0.95 | 0.25 | 0.87 | 0.39 | 1.06 | 1.63 | 9.86 |
| 1.57 | 0.83 | 2.39 | 0.74 | 1.12 | 0.77 | 1.04 | 7.96 | 1.07 | 1.31 | 0.72 |
| 1.08 | 0.82 | 0.95 | 1.16 | 1.07 | 0.96 | 1.49 | 1.92 | 4.19 | 1.21 | 0.97 |
| 1.2 | 1.37 | 1.26 | 0.91 | 0.96 | 1.26 | 1.2 | 1.21 | 1.64 | 1.03 | 1.8 |
| 1.1 | 1.06 | 1.16 | 0.98 | 1.59 | 1.02 | 0.85 | 1 | 1.42 | 1.32 | 2.24 |
| 1.32 | 1.24 | 1.45 | 0.75 | 1.21 | 0.41 | 1.07 | 0.68 | 0.31 | 0.52 | 0.8 |
| 0.95 | 1.18 | 0.98 | 1 | 1.09 | 0.97 | 0.92 | 1.9 | 0.73 | 0.94 | 1.04 |
| 0.6 | 0.68 | 1.2 | 0.9 | 1.13 | 0.97 | 0.95 | 0.65 | 4.59 | 5.78 | 0.9 |
| 0.88 | 1.17 | 1.43 | 1.02 | 0.79 | 0.8 | 0.94 | 0.91 | 7.16 | 1.49 | 21.22 |
| 0.9 | 1.01 | 0.82 | 0.91 | 0.87 | 1.15 | 0.81 | 0.87 | 0.93 | 0.97 | 0.75 |

| | | | | | | | | | | |
|---|---|---|---|---|---|---|---|---|---|---|
| 0.89 | 0.86 | 1.02 | 1.29 | 0.77 | 1.35 | 0.36 | 2.46 | 0.26 | 1.58 | 1.12 |
| 2.03 | 0.99 | 1.16 | 1.21 | 1.26 | 0.76 | 0.84 | 2.9 | 1.48 | 0.93 | 1.4 |
| 0.7 | 1.05 | 0.91 | 1.29 | 2.26 | 1.16 | 0.7 | 2.18 | 1.62 | 1.09 | 2 |
| 0.77 | 1.05 | 1.52 | 0.99 | 0.95 | 1.04 | 3.24 | 1.16 | 1.02 | 1.28 | 10.61 |
| 1.38 | 0.93 | 0.98 | 0.82 | 1.95 | 1.07 | 0.82 | 0.9 | 0.68 | 1.35 | 3 |
| 1.06 | 0.89 | 0.97 | 0.94 | 1.11 | 0.92 | 1.01 | 1 | 0.09 | 0.75 | 0.44 |
| 1.11 | 0.97 | 0.8 | 0.98 | 0.92 | 1.05 | 0.86 | 0.67 | 0.73 | 0.89 | 0.45 |
| 1.11 | 0.99 | 2.37 | 0.76 | 0.95 | 1.13 | 0.8 | 1.4 | 0.98 | 0.98 | 0.64 |
| 1.07 | 1.01 | 0.81 | 0.88 | 1.95 | 0.93 | 1.33 | 1.15 | 0.09 | 1.3 | 0.37 |
| 0.92 | 0.8 | 1.26 | 1.4 | 2.08 | 1.04 | 0.84 | 0.17 | 10.54 | 1.24 | 1.7 |
| 0.97 | 0.86 | 0.82 | 0.95 | 2.27 | 1.08 | 0.47 | 0.05 | 8.74 | 0.96 | 1.65 |
| 1.21 | 0.93 | 0.84 | 0.8 | 0.87 | 1.3 | 0.92 | 1.65 | 3.13 | 1.17 | 0.97 |
| 1.42 | 1.27 | 3.87 | 1.17 | 3.38 | 1.23 | 2.59 | 9.81 | 1.38 | 0.88 | 1.09 |
| 1.52 | 0.74 | 0.84 | 0.89 | 0.76 | 0.94 | 0.67 | 0.43 | 0.97 | 0.88 | 1.03 |
| 1.2 | 0.58 | 1.24 | 2.13 | 0.78 | 2.77 | 1.03 | 4.55 | 4.09 | 0.29 | 1.23 |
| 1.05 | 1.03 | 1.59 | 1 | 0.78 | 0.61 | 1 | 4.35 | 3.22 | 1.01 | 3.33 |
| 1.22 | 0.87 | 1.11 | 0.7 | 1.02 | 1.09 | 1.58 | 0.72 | 0.45 | 0.95 | 1.49 |
| 0.9 | 1.06 | 0.93 | 0.83 | 0.79 | 1.1 | 0.72 | 0.91 | 0.75 | 1.12 | 0.97 |
| 1.38 | 1.15 | 0.86 | 0.67 | 1.44 | 1.24 | 0.74 | 0.85 | 3.96 | 3.3 | 1.46 |
| 0.59 | 1.29 | 1.01 | 0.97 | 1.07 | 0.86 | 0.97 | 1.2 | 0.68 | 1.5 | 0.3 |
| 1.33 | 0.59 | 0.77 | 1.03 | 1.1 | 0.82 | 1.35 | 0.53 | 1.3 | 1.02 | 1.24 |
| 0.94 | 0.78 | 0.64 | 1.16 | 1.04 | 0.79 | 1.23 | 1.33 | 1.06 | 1.41 | 0.41 |
| 0.94 | 1.23 | 1.29 | 1.14 | 1.47 | 0.97 | 1.16 | 2.49 | 1 | 1.13 | 1.26 |
| 1.26 | 1.31 | 0.9 | 0.98 | 0.37 | 0.96 | 0.27 | 1.04 | 0.77 | 1.01 | 0.74 |
| 0.62 | 1.04 | 0.97 | 0.74 | 2.85 | 0.99 | 0.93 | 0.99 | 4.51 | 1 | 0.94 |
| 0.93 | 1.07 | 1.1 | 1.12 | 1.19 | 0.93 | 0.78 | 0.58 | 0.68 | 1.09 | 1.07 |
| 0.89 | 1.19 | 1.12 | 1.16 | 1.83 | 1.24 | 0.66 | 0.74 | 0.98 | 1.31 | 0.62 |
| 1.26 | 0.98 | 0.83 | 1.04 | 0.86 | 0.64 | 1.37 | 1.45 | 0.92 | 0.9 | 1.67 |
| 0.85 | 0.97 | 1.05 | 1.1 | 0.72 | 1.09 | 3.08 | 1.1 | 1.4 | 1.01 | 1.88 |
| 1.04 | 1.08 | 0.98 | 0.74 | 4.5 | 0.98 | 1.12 | 0.67 | 6 | 0.83 | 2.86 |
| 0.89 | 0.83 | 1.36 | 1.11 | 1.25 | 1.03 | 0.98 | 2.76 | 1.2 | 1.24 | 1.58 |
| 1.66 | 0.92 | 0.96 | 1.03 | 1.08 | 1.05 | 13.94 | 6.27 | 0.6 | 0.95 | 0.78 |
| 0.97 | 1.03 | 1.23 | 0.96 | 1.26 | 0.94 | 0.98 | 1.28 | 1.04 | 1.06 | 2.84 |

| | | | | | | | | | | |
|---|---|---|---|---|---|---|---|---|---|---|
| 1.97 | 1.98 | 0.7 | 0.89 | 1.07 | 1.64 | 3.9 | 0.77 | 0.54 | 0.81 | 2 |
| 0.89 | 1.05 | 1.11 | 1.24 | 1.15 | 1.18 | 1.05 | 2.1 | 1.47 | 1.22 | 1.57 |
| 0.86 | 1.01 | 0.93 | 1.17 | 1.78 | 1.01 | 0.97 | 0.89 | 0.88 | 0.97 | 1.19 |
| 1.27 | 0.61 | 1.04 | 2.11 | 1.5 | 1.14 | 1 | 0.9 | 0.67 | 3.63 | 1.83 |
| 0.16 | 0.9 | 0.49 | 1.45 | 0.78 | 0.96 | 0.78 | 2.33 | 2.94 | 0.93 | 1.56 |
| 1.32 | 1.01 | 0.95 | 1.01 | 1.23 | 0.99 | 1.01 | 1.18 | 2.3 | 1.62 | 1.74 |
| 0.78 | 0.76 | 0.51 | 1.09 | 1.46 | 0.89 | 0.78 | 2.62 | 0.95 | 0.54 | 2.35 |
| 0.93 | 0.86 | 0.86 | 1.16 | 0.91 | 0.99 | 1.22 | 1.02 | 1.39 | 0.9 | 1.18 |
| 2.49 | 0.97 | 0.89 | 1.06 | 0.58 | 1.39 | 0.66 | 2.01 | 8.26 | 0.91 | 1.5 |
| 0.72 | 0.71 | 0.85 | 1.33 | 1.79 | 0.77 | 1.02 | 3.52 | 0.88 | 0.61 | 3.36 |
| 0.36 | 1 | 1.12 | 0.9 | 0.59 | 0.89 | 1.33 | 2.3 | 0.67 | 1.3 | 0.56 |
| 1.01 | 1.4 | 1.19 | 1.15 | 0.74 | 1.07 | 0.85 | 0.81 | 0.64 | 1.82 | 1.44 |
| 1.01 | 0.71 | 0.59 | 0.95 | 4.46 | 0.97 | 1 | 1.95 | 0.38 | 0.81 | 0.7 |
| 0.97 | 1.15 | 0.85 | 1.05 | 0.82 | 0.99 | 0.82 | 3.18 | 0.51 | 0.95 | 0.97 |
| 0.77 | 1.14 | 1.41 | 0.95 | 0.74 | 1.05 | 2.1 | 0.86 | 1.22 | 1.4 | 7.34 |
| 1.38 | 1.16 | 0.99 | 1.11 | 1.22 | 1.11 | 0.5 | 0.96 | 1.55 | 1.39 | 1.01 |
| 1.16 | 1.09 | 1.18 | 0.96 | 1.62 | 0.95 | 1.19 | 1.71 | 1.12 | 0.91 | 1.39 |
| 0.8 | 1.18 | 1.02 | 0.8 | 0.68 | 1.14 | 0.91 | 0.67 | 1.14 | 0.97 | 0.85 |
| 0.89 | 1.02 | 0.6 | 0.93 | 1.1 | 1.06 | 2.32 | 1.38 | 0.94 | 0.69 | 1 |
| 1.41 | 0.87 | 0.92 | 2.32 | 1.64 | 0.94 | 0.89 | 1.02 | 1.19 | 0.88 | 2.17 |
| 0.9 | 1.06 | 0.83 | 0.51 | 1.21 | 0.87 | 0.73 | 0.99 | 0.65 | 0.53 | 0.31 |
| 0.64 | 1.73 | 3.11 | 0.3 | 11.76 | 1.5 | 0.16 | 0.05 | 0.71 | 0.72 | 1.24 |
| 0.85 | 1.08 | 1.26 | 1.1 | 1.65 | 0.95 | 0.75 | 3.21 | 3.89 | 0.89 | 0.95 |
| 1.01 | 1.06 | 0.36 | 1.24 | 0.99 | 1.23 | 4.07 | 0.88 | 0.77 | 0.87 | 1.93 |
| 1.03 | 0.81 | 0.99 | 1 | 1.5 | 0.95 | 1.35 | 0.94 | 2.11 | 1.03 | 0.3 |
| 7.38 | 1.11 | 0.97 | 0.96 | 1.88 | 1.36 | 8.09 | 34.58 | 5.13 | 2.78 | 0.82 |
| 0.92 | 1.04 | 1.22 | 1.13 | 1.04 | 1.38 | 1.46 | 1.33 | 0.88 | 1.17 | 1.02 |
| 1.01 | 1 | 0.75 | 0.87 | 1.32 | 0.91 | 1.02 | 2.91 | 0.81 | 1.11 | 4.16 |
| 1.21 | 0.99 | 1.17 | 0.96 | 0.82 | 1 | 1.03 | 1.25 | 1.02 | 0.95 | 7.67 |
| 3.68 | 0.93 | 1.69 | 0.99 | 1.26 | 0.84 | 1.1 | 3.69 | 0.93 | 1.06 | 1.12 |
| 1.73 | 0.65 | 1.69 | 1.19 | 1.13 | 1.04 | 1.12 | 5.28 | 0.56 | 0.84 | 1.06 |
| 0.55 | 0.96 | 0.78 | 0.93 | 1 | 1.03 | 0.88 | 0.97 | 1.67 | 0.89 | 0.4 |
| 0.65 | 1.17 | 1.02 | 0.77 | 0.26 | 1.38 | 1.03 | 1.78 | 1.41 | 0.86 | 1.72 |

| | | | | | | | | | | |
|---|---|---|---|---|---|---|---|---|---|---|
| 1.86 | 1.45 | 1.45 | 0.93 | 1.05 | 1.01 | 1.09 | 1.32 | 1.61 | 0.83 | 0.61 |
| 0.33 | 1.2 | 0.9 | 1.25 | 0.72 | 0.87 | 1.5 | 0.31 | 0.69 | 1.12 | 1.68 |
| 0.91 | 1.06 | 0.91 | 0.84 | 0.96 | 1.15 | 1.08 | 1.42 | 1.05 | 1.06 | 0.31 |
| 0.22 | 0.54 | 0.64 | 0.87 | 0.58 | 1.13 | 0.73 | 0.89 | 0.46 | 0.66 | 0.91 |
| 1.32 | 0.91 | 1.06 | 0.89 | 0.97 | 0.94 | 0.85 | 1.29 | 0.77 | 0.85 | 1.11 |
| 0.64 | 0.89 | 0.84 | 1.36 | 1.24 | 1.08 | 1.98 | 1.15 | 0.81 | 0.64 | 3.54 |
| 1.33 | 0.77 | 1.2 | 0.74 | 0.61 | 1.08 | 0.97 | 0.88 | 0.21 | 0.83 | 0.63 |
| 0.76 | 0.87 | 0.79 | 1.14 | 0.65 | 0.97 | 0.72 | 1.1 | 0.51 | 1.13 | 0.78 |
| 1.43 | 1.81 | 0.47 | 0.77 | 1.25 | 1.34 | 3.12 | 1.8 | 0.74 | 0.99 | 2.32 |
| 0.61 | 1.12 | 1.84 | 1.36 | 3.01 | 1.65 | 0.85 | 0.77 | 0.79 | 0.98 | 1.22 |
| 1.02 | 0.86 | 0.91 | 0.75 | 0.82 | 1.12 | 0.74 | 0.79 | 1.22 | 1.26 | 0.96 |
| 1.09 | 1.1 | 1.11 | 1.26 | 0.54 | 0.94 | 0.62 | 0.79 | 1 | 0.82 | 1.7 |
| 0.67 | 1.1 | 0.82 | 0.83 | 0.81 | 1.35 | 0.93 | 1.55 | 0.16 | 1 | 1.07 |
| 0.46 | 0.94 | 1.96 | 0.88 | 1.11 | 1.06 | 0.86 | 1.78 | 2.34 | 4.58 | 0.87 |
| 1.44 | 0.95 | 0.82 | 1.23 | 1.19 | 1.38 | 0.97 | 0.99 | 1.31 | 1.06 | 1.12 |
| 0.65 | 0.82 | 0.51 | 1.26 | 0.76 | 0.95 | 1.2 | 0.94 | 3.36 | 0.81 | 3.16 |
| 1.58 | 0.79 | 0.74 | 0.98 | 0.44 | 1.09 | 0.74 | 1.27 | 1.91 | 0.6 | 0.8 |
| 0.66 | 0.96 | 1.07 | 0.9 | 1.12 | 0.93 | 1.01 | 0.33 | 1.1 | 1.14 | 0.98 |
| 1.2 | 0.89 | 3.64 | 1.07 | 0.57 | 1.38 | 2.72 | 2.77 | 3.43 | 1.4 | 0.8 |
| 0.2 | 0.5 | 0.46 | 1.1 | 0.08 | 2.09 | 0.9 | 1.08 | 0.04 | 1.14 | 0.04 |
| 0.67 | 0.87 | 1.26 | 0.9 | 1.33 | 0.87 | 1.78 | 3.85 | 1.32 | 0.75 | 3.26 |
| 0.71 | 0.66 | 0.61 | 1.17 | 1.66 | 0.73 | 0.91 | 2.24 | 0.86 | 0.6 | 2.77 |
| 0.17 | 0.99 | 0.65 | 0.63 | 1.99 | 0.6 | 1.05 | 0.26 | 0.21 | 1.07 | 0.64 |
| 1.6 | 1.18 | 0.58 | 0.65 | 2.3 | 0.9 | 1.42 | 2.03 | 0.12 | 0.59 | 0.67 |
| 0.78 | 1.14 | 0.83 | 1 | 0.63 | 1.07 | 1 | 0.98 | 1.1 | 0.89 | 1.15 |
| 0.89 | 1.24 | 1.29 | 0.37 | 0.81 | 0.61 | 0.83 | 1.86 | 0.83 | 1.11 | 7.21 |
| 0.43 | 0.57 | 1.09 | 0.92 | 0.85 | 0.84 | 0.59 | 5.62 | 1.15 | 1.19 | 1.97 |
| 1.13 | 1.19 | 1.2 | 1.01 | 0.13 | 1.24 | 0.94 | 0.53 | 0.71 | 1.19 | 0.4 |
| 3.92 | 0.86 | 0.95 | 0.95 | 1.4 | 0.97 | 0.79 | 1.26 | 0.52 | 0.61 | 1.9 |
| 40.94 | 0.41 | 0.48 | 1.54 | 1 | 0.89 | 1.66 | 3.93 | 0.15 | 0.5 | 16 |
| 1.54 | 1.02 | 0.71 | 0.91 | 0.1 | 1.59 | 1.83 | 1.4 | 0.91 | 1.41 | 0.49 |
| 0.86 | 1 | 1.13 | 1.16 | 0.92 | 1.01 | 0.93 | 2.48 | 1.02 | 0.98 | 1.92 |
| 0.8 | 2.45 | 1.36 | 0.46 | 1.67 | 0.41 | 1.13 | 0.65 | 0.56 | 0.64 | 0.52 |

| | | | | | | | | | | |
|---|---|---|---|---|---|---|---|---|---|---|
| 1.28 | 0.97 | 0.93 | 0.97 | 0.78 | 1.03 | 1.34 | 1.11 | 0.79 | 0.91 | 3.2 |
| 0.92 | 1.26 | 1.07 | 1.01 | 1.03 | 0.92 | 0.97 | 1.01 | 1.2 | 0.66 | 0.96 |
| 0.62 | 0.63 | 0.67 | 2.04 | 2.03 | 4.18 | 0.94 | 1.75 | 1.32 | 5.4 | 1.4 |
| 1.94 | 0.15 | 1.35 | 0.62 | 0.7 | 0.52 | 1.05 | 0.64 | 1.06 | 0.71 | 1.02 |
| 0.28 | 1.48 | 0.95 | 1.14 | 8.84 | 1.11 | 0.92 | 0.91 | 0.58 | 0.52 | 3.45 |
| 1.27 | 0.92 | 0.97 | 0.89 | 6.22 | 0.86 | 1.1 | 3.73 | 2.42 | 1.08 | 1.98 |
| 0.75 | 0.99 | 0.99 | 1 | 0.88 | 0.97 | 1.1 | 1.69 | 1.42 | 0.94 | 1.19 |
| 0.42 | 1.09 | 1.11 | 0.75 | 1.25 | 1.09 | 0.95 | 0.45 | 1.56 | 1.01 | 0.62 |
| 1.16 | 1.63 | 0.65 | 1.13 | 0.3 | 1.12 | 1.03 | 2.24 | 1.11 | 0.94 | 1.19 |
| 2.38 | 1.03 | 1.28 | 1.29 | 2.45 | 0.66 | 0.66 | 0.97 | 0.59 | 1.2 | 2.13 |
| 0.74 | 0.89 | 1.17 | 1.07 | 0.97 | 0.7 | 1.52 | 0.39 | 0.73 | 1.26 | 2.92 |
| 0.52 | 0.57 | 0.58 | 4 | 2.45 | 3.84 | 0.51 | 25.94 | 0.52 | 6.6 | 1.17 |
| 2.15 | 1.52 | 1.07 | 1.03 | 1.15 | 1.56 | 0.97 | 1.22 | 1.38 | 1.01 | 1.4 |
| 1.15 | 1.41 | 1.44 | 0.69 | 0.93 | 0.85 | 0.69 | 0.32 | 0.78 | 0.52 | 4.67 |

| 205883_at | 208944_at | 202344_at | 205402_x_at | 213721_at | 202724_s_at | 209602_s_at | 214438_at | 206536_s_at | 204790_at | 219388_at |
|---|---|---|---|---|---|---|---|---|---|---|
| 5.05 | 1.15 | 1.08 | 1.99 | 1.69 | 1.18 | 10.4 | 1.73 | 1.11 | 0.32 | 1.78 |
| 0.92 | 1.24 | 0.93 | 1.28 | 0.84 | 1.56 | 2.24 | 1.08 | 2.11 | 0.98 | 1.05 |
| 1.08 | 1.47 | 4.29 | 0.48 | 3.91 | 1.23 | 1.25 | 1.84 | 3.27 | 2.2 | 0.97 |
| 2.06 | 1.19 | 1.04 | 3.03 | 2.09 | 3.73 | 0.81 | 1.39 | 1.32 | 1.05 | 1.07 |
| 3.76 | 1.43 | 1.08 | 1.1 | 2.75 | 1.57 | 2.44 | 0.83 | 1.11 | 0.95 | 0.58 |
| 1.39 | 1.02 | 1.32 | 1.47 | 1.25 | 1.06 | 8.43 | 2.38 | 0.87 | 0.94 | 1.65 |
| 1.47 | 1.25 | 1.22 | 4.36 | 2.13 | 1.79 | 0.11 | 9.83 | 0.79 | 1.67 | 0.11 |
| 0.72 | 1.04 | 1.09 | 0.94 | 0.87 | 0.76 | 0.88 | 0.93 | 1.05 | 1.56 | 0.7 |
| 0.66 | 0.93 | 0.96 | 0.59 | 0.33 | 1.21 | 1.48 | 2.22 | 1.41 | 0.91 | 1.43 |
| 7.57 | 0.6 | 0.91 | 0.58 | 1.27 | 1.07 | 1.23 | 2.24 | 3.62 | 2.78 | 0.69 |
| 0.48 | 0.42 | 0.79 | 2.1 | 1.03 | 1.7 | 1 | 0.43 | 0.77 | 0.84 | 0.16 |
| 0.67 | 0.84 | 1.19 | 0.78 | 1.52 | 1.09 | 1.17 | 0.91 | 0.89 | 0.73 | 1.79 |
| 1.22 | 0.97 | 1.03 | 0.44 | 0.88 | 0.89 | 1.26 | 1.05 | 1.13 | 1.05 | 0.95 |
| 59.78 | 0.75 | 0.22 | 0.14 | 60.13 | 1.54 | 1.92 | 1.5 | 0.41 | 2.81 | 1.02 |
| 2.4 | 1.52 | 1.34 | 0.88 | 0.66 | 1.67 | 0.64 | 8.27 | 2.62 | 0.97 | 0.8 |
| 0.94 | 1.26 | 0.97 | 2.41 | 2.16 | 1.11 | 2.73 | 1.29 | 0.82 | 1.35 | 0.99 |
| 153.83 | 1.9 | 1.17 | 5.24 | 1.77 | 0.87 | 0.56 | 1.06 | 0.93 | 0.84 | 3.63 |
| 7.42 | 0.78 | 0.91 | 1.44 | 3.17 | 2.67 | 0.91 | 1.41 | 1.4 | 1.86 | 0.88 |
| 0.55 | 0.53 | 2.19 | 2.15 | 2.73 | 4.61 | 3 | 1.23 | 0.98 | 1 | 0.97 |
| 1.19 | 2.08 | 0.8 | 1.24 | 1.7 | 1.43 | 5.25 | 1.4 | 1.95 | 0.92 | 0.73 |
| 1.62 | 0.66 | 0.5 | 2.61 | 0.83 | 1.33 | 4.12 | 1.59 | 6.3 | 1.09 | 4.39 |
| 8.55 | 0.62 | 1.78 | 10.52 | 4.55 | 1.04 | 4.36 | 13.74 | 0.99 | 1.89 | 0.94 |
| 0.28 | 0.87 | 0.88 | 0.73 | 1.13 | 1.58 | 0.47 | 0.85 | 1.43 | 0.88 | 0.92 |
| 1.95 | 0.95 | 1.02 | 2.26 | 4.57 | 0.4 | 0.14 | 6.98 | 1.13 | 0.36 | 0.06 |
| 0.17 | 1.46 | 1.53 | 1.52 | 0.11 | 0.25 | 0.99 | 1.37 | 0.75 | 0.05 | 4.07 |
| 1.43 | 1.13 | 1.16 | 2.68 | 0.63 | 0.62 | 4.88 | 0.58 | 1.47 | 1.38 | 0.85 |
| 0.25 | 0.79 | 0.41 | 0.75 | 2.91 | 1.07 | 0.12 | 0.75 | 0.95 | 1.55 | 1.3 |
| 18.34 | 1 | 1.96 | 1.97 | 1.54 | 3.12 | 1.92 | 12.72 | 2.26 | 1.01 | 5.84 |
| 1.13 | 0.95 | 20.52 | 13.31 | 1.23 | 1.57 | 2.83 | 5.23 | 1.81 | 0.84 | 1.77 |
| 1.45 | 0.97 | 1.19 | 0.8 | 1.18 | 1.15 | 1.27 | 1.44 | 1.05 | 1.22 | 2.4 |

| | | | | | | | | | | |
|---|---|---|---|---|---|---|---|---|---|---|
| 0.73 | 0.97 | 1 | 1.39 | 3.01 | 0.7 | 1.2 | 0.71 | 1.8 | 0.65 | 1.5 |
| 0.56 | 0.94 | 3.2 | 0.59 | 0.43 | 2.09 | 0.1 | 11.65 | 0.94 | 3.05 | 0.15 |
| 1.19 | 0.92 | 0.96 | 0.94 | 5.77 | 1.28 | 2 | 0.73 | 1.18 | 1.13 | 1.16 |
| 0.33 | 0.38 | 0.62 | 0.95 | 1.45 | 0.43 | 0.29 | 0.75 | 0.72 | 0.76 | 2.26 |
| 2.12 | 0.9 | 4.27 | 0.72 | 16.17 | 0.04 | 0.42 | 0.5 | 8.69 | 1.02 | 0.65 |
| 2.38 | 0.53 | 13.96 | 36.8 | 7.59 | 1.26 | 1.83 | 4.89 | 3.44 | 0.67 | 4.2 |
| 1.15 | 0.37 | 1.58 | 1.41 | 1.75 | 2.28 | 0.91 | 0.34 | 1.13 | 0.77 | 1.96 |
| 0.79 | 0.59 | 1.01 | 0.41 | 0.78 | 1.23 | 0.37 | 0.8 | 1.12 | 0.72 | 2.29 |
| 1.14 | 1.41 | 0.79 | 5.02 | 3.18 | 1.6 | 1.16 | 1.36 | 2.37 | 0.44 | 0.96 |
| 0.4 | 3.89 | 1.24 | 0.04 | 0.17 | 1.76 | 37.5 | 6.44 | 1.98 | 1.23 | 0.03 |
| 3.34 | 0.71 | 1.38 | 0.32 | 1.1 | 0.58 | 1.5 | 0.23 | 1.3 | 3.08 | 0.83 |
| 2.55 | 4.87 | 1.13 | 1.47 | 0.58 | 0.93 | 0.93 | 0.95 | 0.92 | 1.05 | 1.18 |
| 2.41 | 0.77 | 0.97 | 0.97 | 0.93 | 1.25 | 2.29 | 1.03 | 1.51 | 1.79 | 1 |
| 0.92 | 1.67 | 1.89 | 1.24 | 5.62 | 1.94 | 0.62 | 0.88 | 0.84 | 2.59 | 0.92 |
| 1.68 | 0.85 | 0.72 | 0.9 | 1.44 | 1.26 | 1.93 | 0.7 | 1.33 | 1.37 | 0.95 |
| 1.04 | 1.45 | 0.99 | 0.75 | 1 | 1.37 | 12.97 | 1.75 | 1.92 | 2.45 | 5.09 |
| 0.13 | 0.99 | 1.07 | 1.46 | 1.4 | 0.99 | 1.09 | 0.75 | 0.77 | 1.21 | 1.08 |
| 0.59 | 1.44 | 0.9 | 0.91 | 5.15 | 1.1 | Inf | 1.33 | 0.85 | 1.14 | 1.07 |
| 0.83 | 0.95 | 1.18 | 1.29 | 0.83 | 1 | 1.06 | 0.96 | 0.93 | 0.99 | 0.95 |
| 4.48 | 1.18 | 1.5 | 1.98 | 0.83 | 1.06 | 0.96 | 1.38 | 1.42 | 1.24 | 7.24 |
| 0.37 | 0.97 | 0.82 | 0.52 | 0.48 | 1.02 | 0.6 | 1.31 | 0.93 | 1.08 | 1.09 |
| 2.78 | 0.95 | 1.54 | 0.82 | 5.55 | 1.14 | 0.83 | 1.12 | 0.9 | 0.93 | 0.91 |
| 0.71 | 0.99 | 0.83 | 0.78 | 0.75 | 0.97 | 1.37 | 1.23 | 1 | 2.33 | 1.03 |
| 1.79 | 1.72 | 1.23 | 0.59 | 0.91 | 0.99 | 25.46 | 1.35 | 1.7 | 1.16 | 1.53 |
| 1.52 | 1.33 | 1.52 | 10.09 | 1.15 | 1.03 | NA | 0.66 | 1.19 | 2.7 | 0.52 |
| 1.09 | 0.85 | 0.81 | 7.33 | 1.21 | 0.55 | 0 | 0.84 | 1.25 | 1.81 | 0.88 |
| 0.54 | 0.62 | 1.41 | 24.54 | 0.23 | 0.55 | 1.29 | 0.66 | 1.36 | 1.33 | 4.46 |
| 2.36 | 1.49 | 0.98 | 1.87 | 1.59 | 1.08 | 5.28 | 0.91 | 0.58 | 1.76 | 1.01 |
| 1.47 | 0.78 | 1.01 | 2.05 | 0.95 | 1.07 | 1 | 1.03 | 1.12 | 0.96 | 1.02 |
| 2 | 0.63 | 0.77 | 0.62 | 1.25 | 1.54 | 3.03 | 0.86 | 1.53 | 2.01 | 1.07 |
| 7.13 | 1.06 | 0.9 | 1.21 | 0.34 | 0.64 | 15.32 | 1.96 | 0.87 | 0.98 | 0.77 |
| 10 | 1.22 | 0.99 | 7.07 | 2.01 | 1.37 | 0.66 | 2.75 | 1 | 2.14 | 1.05 |
| 1.35 | 10.3 | 1.02 | 1.31 | 0.53 | 1.12 | 0.83 | 1.42 | 1.22 | 1.19 | 1.56 |

| | | | | | | | | | | |
|---|---|---|---|---|---|---|---|---|---|---|
| 0.36 | 1.19 | 1.45 | 3.17 | 11.25 | 0.6 | 3.5 | 2.41 | 0.65 | 0.9 | 1.77 |
| 8.62 | 0.6 | 3.7 | 0.64 | 10.14 | 1.99 | Inf | 1.08 | 1.29 | 1.88 | 0.79 |
| 1.77 | 0.97 | 1.03 | 1.42 | 1.1 | 0.9 | 4.89 | 1.27 | 0.77 | 1.47 | 2.72 |
| 1.45 | 1.8 | 1.25 | 1.82 | 2.26 | 1.09 | 8.18 | 2.26 | 2.64 | 10.11 | 3.78 |
| 3.79 | 1.06 | 0.93 | 1.22 | 1.16 | 1.05 | 2.78 | 1.12 | 0.99 | 0.86 | 9.16 |
| 1.22 | 1.17 | 1.04 | 0.82 | 3.38 | 0.5 | 2.6 | 2.63 | 1.92 | 0.72 | 0.78 |
| 4.62 | 0.45 | 0.75 | 1.34 | 0.66 | 0.65 | 0.71 | 1.21 | 1.35 | 1.73 | 1.08 |
| 1.39 | 0.81 | 5.81 | 3.12 | 0.68 | 0.78 | 0.6 | 1.05 | 0.68 | 1.6 | 0.56 |
| 0.27 | 0.78 | 0.78 | 0.86 | 1.42 | 0.38 | 1.21 | 1.03 | 1.05 | 0.52 | 1.05 |
| 0.82 | 1.29 | 0.52 | 1.29 | 1.22 | 0.88 | 1.05 | 1.88 | 1.23 | 0.86 | 1.67 |
| 2.01 | 2.3 | 2.37 | 3.74 | 1.04 | 0.68 | 12.12 | 1.25 | 0.97 | 1.38 | 1.38 |
| 0.79 | 2.62 | 1.49 | 4.5 | 0.72 | 1.06 | 0.38 | 0.64 | 1.64 | 1.23 | 1 |
| 0.88 | 1.33 | 1.17 | 11 | 1.19 | 0.86 | 1.28 | 2.2 | 0.83 | 1.11 | 2.58 |
| 2.66 | 3.14 | 1.19 | 2.53 | 2.85 | 1.88 | 0.9 | 1.53 | 1.78 | 1.08 | 3.32 |
| 1.1 | 1.17 | 1.03 | 1.57 | 0.12 | 0.79 | 1 | 0.46 | 1.43 | 0.6 | 0.6 |
| 0.3 | 1.62 | 0.53 | 0.03 | 0.01 | 1.7 | 0.61 | 0.75 | 0.88 | 2.64 | 0.09 |
| 0.92 | 0.62 | 0.85 | 7.15 | 3.04 | 1.84 | 0.5 | 0.91 | 0.92 | 1.27 | 0.96 |
| 0.2 | 0.77 | 0.9 | 0.67 | 1.37 | 4.16 | 15 | 0.36 | 1.89 | 11.08 | 0.76 |
| 1.05 | 1.01 | 1 | 0.64 | 1.25 | 0.56 | 1.45 | 0.9 | 1.01 | 0.85 | 1.49 |
| 2.01 | 0.91 | 2.12 | 2.48 | 1.35 | 1.78 | 30.56 | 3.69 | 3.15 | 4.47 | 1.8 |
| 4.91 | 1.21 | 5.79 | 0.85 | 1.87 | 4.28 | 0.83 | 2.94 | 0.93 | 8.72 | 1.89 |
| 0.88 | 1 | 0.94 | 2.89 | 1.19 | 0.78 | 0.62 | 0.28 | 1.21 | 1.31 | 1.27 |
| 0.96 | 1.17 | 1.23 | 3.44 | 1.71 | 1.18 | 0.12 | 5.42 | 0.89 | 1.55 | 0.56 |
| 11.36 | 0.59 | 1.3 | 0.79 | 1.87 | 1.83 | 0.55 | 0.53 | 2.26 | 1.16 | 0.94 |
| 1.44 | 1.02 | 1.09 | 0.51 | 1.25 | 2.25 | 0.6 | 1.19 | 1.18 | 1.14 | 1.06 |
| 0.91 | 1.08 | 0.9 | 1.13 | 0.41 | 0.95 | 0.7 | 1.05 | 1.74 | 0.69 | 0.64 |
| 0.92 | 0.87 | 1.24 | 0.9 | 0.94 | 0.79 | 1.06 | 1.34 | 1.06 | 0.97 | 3.6 |
| 1.63 | 1.22 | 1.82 | 3.65 | 0.4 | 0.86 | 1.12 | 0.7 | 0.76 | 0.28 | 1.02 |
| 0.8 | 1.39 | 0.99 | 3.06 | 0.76 | 1.04 | 0.82 | 1.27 | 1.24 | 1.18 | 1.01 |
| 1.12 | 0.83 | 0.71 | 1.98 | 1.2 | 0.98 | 2.94 | 0.84 | 0.84 | 1.03 | 0.68 |
| 0.83 | 0.84 | 1.31 | 0.73 | 1.21 | 1.1 | 0.63 | 0.99 | 1.05 | 1.21 | 3.08 |
| 0.55 | 0.96 | 1.32 | 1.23 | 1.36 | 1.2 | 2.15 | 0.58 | 1.09 | 0.51 | 1.02 |
| 3.72 | 1.03 | 1.11 | 2.07 | 1.74 | 1.26 | 1.97 | 2.62 | 1.39 | 2.39 | 0.1 |

| | | | | | | | | | | |
|---|---|---|---|---|---|---|---|---|---|---|
| 3.91 | 0.81 | 2.77 | 0.66 | 1.46 | 1.06 | 1.64 | 2.1 | 1.62 | 0.48 | 1.22 |
| 1.75 | 0.83 | 0.84 | 1.15 | 1.48 | 0.54 | 0.95 | 0.37 | 1.25 | 3.89 | 1.08 |
| 2.62 | 1.19 | 1.07 | 6.03 | 2.27 | 0.83 | 0.57 | 0.18 | 1.16 | 0.94 | 0.82 |
| 1.11 | 0.88 | 0.78 | 0.43 | 2.86 | 1.44 | 1.08 | 1.63 | 0.48 | 0.18 | 1.28 |
| 0.99 | 2.62 | 0.64 | 0.93 | 1.26 | 0.46 | 1.1 | 2.71 | 1.45 | 0.91 | 0.53 |
| 1.86 | 8.1 | 1.62 | 3.27 | 3.17 | 0.83 | 0.32 | 3.08 | 1 | 0.96 | 0.43 |
| 1 | 0.9 | 1.27 | 1.67 | 0.53 | 0.99 | 2.08 | 1.51 | 1.89 | 1.55 | 1.83 |
| 3.9 | 1.53 | 1.21 | 0.7 | 3.89 | 1.03 | 2.22 | 1.06 | 0.82 | 1.69 | 0.8 |
| 3.65 | 1.89 | 0.61 | 1.23 | 0.44 | 1.33 | 0.71 | 1.19 | 1.38 | 1.32 | 0.68 |
| 1.21 | 1.02 | 0.95 | 0.91 | 1.52 | 2.15 | 2.09 | 0.2 | 1.36 | 1.34 | 0.9 |
| 1.63 | 0.9 | 1.38 | 3.15 | 1.76 | 1.19 | 1.08 | 1.14 | 0.95 | 1.29 | 2.11 |
| 1.75 | 0.88 | 1 | 2.75 | 1.72 | 1.04 | 0.91 | 1.11 | 1.31 | 1.49 | 0.86 |
| 1.54 | 0.89 | 1.17 | 3.04 | 0.79 | 1.97 | Inf | 0.98 | 1.25 | 0.79 | 1.42 |
| 0.56 | 0.74 | 0.76 | 1.32 | 0.72 | 0.56 | 0.58 | 0.88 | 0.79 | 1.25 | 1.09 |
| 0.9 | 0.93 | 0.98 | 1.74 | 0.48 | 1.46 | 1.03 | 0.74 | 0.73 | 1.05 | 0.58 |
| 1.01 | 1.11 | 0.82 | 4.18 | 1.68 | 1.74 | 25.98 | 1.08 | 1.08 | 1.22 | 3.19 |
| 1.63 | 1.27 | 0.81 | 0.24 | 0.91 | 0.77 | 0.79 | 1.07 | 0.75 | 0.5 | 1.99 |
| 1.26 | 1.67 | 1.46 | 3.88 | 2.53 | 1.11 | 1.32 | 3.29 | 2.44 | 6.63 | 3.63 |
| 2.12 | 1.3 | 0.94 | 1.05 | 1.48 | 0.7 | 7.68 | 1.13 | 1.34 | 1.91 | 0.5 |
| 2.83 | 0.6 | 1.97 | 0.72 | 0.45 | 0.77 | 0.5 | 2.08 | 0.58 | 1.32 | 1.24 |
| 1.3 | 1.6 | 0.87 | 35.5 | 2.19 | 0.74 | 1.31 | 1.75 | 0.88 | 0.73 | 0.78 |
| 11.1 | 0.88 | 0.7 | 0.6 | 3.25 | 0.7 | 1.03 | 2.02 | 1.81 | 1.32 | 0.64 |
| 1.17 | 0.96 | 1 | 0.62 | 1.97 | 1.52 | 0.87 | 0.95 | 0.88 | 1.2 | 1.18 |
| 0.33 | 0.29 | 0.81 | 1.96 | 0.16 | 2.29 | 0.9 | 0.93 | 1.44 | 1.6 | 0.95 |
| 0.99 | 0.79 | 1.07 | 1.42 | 1.87 | 1.51 | 1.01 | 1.2 | 0.44 | 0.45 | 1.12 |
| 0.46 | 1.39 | 1.22 | 0.64 | 0.78 | 0.99 | NA | 1.84 | 0.91 | 1.1 | 1.07 |
| 0.08 | 1.1 | 1.28 | 1.03 | 0.49 | 1.17 | 0.97 | 1 | 0.91 | 1.08 | 0.96 |
| 2.54 | 0.74 | 1.22 | 1.93 | 1.06 | 1.04 | 1.94 | 1.01 | 1.21 | 0.33 | 1.59 |
| 0.66 | 1.38 | 1.42 | 2.45 | 3.62 | 0.47 | 1.24 | 0.58 | 0.72 | 0.42 | 1.21 |
| 0.92 | 0.85 | 0.92 | 3.33 | 1.49 | 0.96 | 0.91 | 2.21 | 0.98 | 0.98 | 1.09 |
| 0.68 | 1.04 | 1.05 | 3.01 | 0.6 | 0.56 | 1.15 | 3.11 | 0.91 | 0.45 | 1.13 |
| 8.62 | 0.6 | 3.7 | 0.64 | 10.14 | 1.99 | Inf | 1.08 | 1.29 | 1.88 | 0.79 |
| 1.27 | 1.43 | 1.15 | 1.44 | 1.54 | 1.68 | 0.91 | 1.24 | 1.34 | 0.71 | 0.87 |

| | | | | | | | | | | |
|---|---|---|---|---|---|---|---|---|---|---|
| 1.73 | 1.24 | 0.91 | 0.66 | 0.98 | 0.61 | 0.87 | 2.06 | 1.79 | 2.24 | 0.77 |
| 0.6 | 0.9 | 0.95 | 1.44 | 0.74 | 1.42 | 0.85 | 0.91 | 0.69 | 0.75 | 1.26 |
| 0.95 | 0.97 | 1.1 | 0.6 | 4.66 | 0.99 | 2.42 | 0.77 | 0.99 | 1.01 | 2.26 |
| 0.72 | 1.21 | 0.8 | 3.56 | 0.68 | 1.04 | 1.22 | 0.89 | 1.43 | 0.76 | 0.95 |
| 1.33 | 0.87 | 0.84 | 1.85 | 1.23 | 2.76 | Inf | 1.22 | 1.04 | 0.89 | 1.95 |
| 0.12 | 1.03 | 0.99 | 0.82 | 0.44 | 0.94 | 0.12 | 0.91 | 1.08 | 0.93 | 1.11 |
| 0.79 | 0.96 | 0.86 | 0.97 | 4.08 | 1.51 | 0.55 | 1.18 | 0.93 | 0.9 | 0.92 |
| 0.93 | 0.94 | 0.96 | 1.1 | 3.47 | 1.37 | 1.52 | 1.08 | 0.68 | 1.2 | 0.95 |
| 13.98 | 0.65 | 0.78 | 0.22 | 1.2 | 1 | 0.59 | 1.04 | 1.44 | 1.19 | 1.95 |
| 0.75 | 0.91 | 0.75 | 1.63 | 0.71 | 1.09 | 1.22 | 1.81 | 1.07 | 1.24 | 2.08 |
| 0.94 | 1.07 | 0.61 | 3.55 | 1.51 | 1.28 | 1.58 | 2.11 | 2.58 | 0.5 | 2.27 |
| 3.01 | 0.79 | 0.97 | 0.93 | 0.62 | 0.83 | 3.33 | 4.59 | 1.21 | 1.33 | 0.87 |
| 1.77 | 2.1 | 1.47 | 1.79 | 1.59 | 1.63 | 5.54 | 2.59 | 3.21 | 7.38 | 3.38 |
| 1.25 | 0.7 | 0.91 | 0.49 | 0.71 | 1.19 | 0.53 | 0.73 | 0.8 | 0.74 | 0.76 |
| 0.2 | 2.71 | 0.86 | 1.35 | 2.04 | 1.26 | 17.69 | 1.03 | 1.3 | 0.71 | 0.78 |
| 1.19 | 0.99 | 1.27 | 0.71 | 0.66 | 0.82 | 0.37 | 4.71 | 0.9 | 0.66 | 0.78 |
| 1.13 | 0.84 | 1.15 | 5.44 | 5.77 | 1.31 | 2.25 | 0.55 | 1.06 | 1.02 | 1.02 |
| 0.89 | 0.7 | 1.29 | 1.33 | 1.31 | 1.01 | 7.13 | 1.33 | 1.13 | 0.72 | 0.79 |
| 0.8 | 0.98 | 0.97 | 0.97 | 2.44 | 0.92 | Inf | 1.11 | 0.71 | 0.66 | 1.44 |
| 1.11 | 1.01 | 0.87 | 0.4 | 0.73 | 0.95 | 1.85 | 0.42 | 0.86 | 1.34 | 1.07 |
| 0.83 | 0.92 | 0.87 | 2.8 | 0.63 | 1.01 | 0.99 | 1.32 | 1.36 | 1.36 | 1.1 |
| 1.16 | 1.19 | 1.72 | 2.32 | 3.13 | 0.65 | 1.25 | 2.14 | 2.07 | 1.73 | 1.04 |
| 0.98 | 0.95 | 0.95 | 0.74 | 0.76 | 1.35 | 5.14 | 1.67 | 1.41 | 1.04 | 1.47 |
| 1.1 | 0.98 | 1.04 | 0.95 | 1.6 | 1.38 | 1.09 | 2.59 | 0.92 | 3.04 | 0.37 |
| 2.43 | 1.46 | 0.75 | 0.56 | 0.9 | 0.68 | 1.48 | 1 | 1.05 | 0.92 | 2.85 |
| 0.79 | 0.66 | 0.99 | 1.61 | 2.2 | 0.84 | 0.98 | 0.8 | 5.06 | 0.79 | 1.19 |
| 0.85 | 1.32 | 1.23 | 0.68 | 1.46 | 1.07 | 6.11 | 1.29 | 0.97 | 1.22 | 1.83 |
| 1.29 | 1.02 | 0.85 | 1.08 | 4.8 | 1.01 | 0.87 | 1.87 | 1.04 | 1.06 | 0.86 |
| 1.38 | 1.06 | 0.95 | 2.81 | 1.01 | 1.09 | 10.21 | 1.13 | 1.09 | 1.11 | 0.72 |
| 1 | 0.78 | 0.97 | 0.95 | 0.98 | 0.79 | 0.14 | 0.77 | 1.1 | 0.82 | 4.5 |
| 1.07 | 0.93 | 1.01 | 0.94 | 0.7 | 1.16 | 2.51 | 0.87 | 1.29 | 0.74 | 1.25 |
| 2.25 | 2.4 | 1.06 | 0.73 | 2.52 | 1.1 | NA | 2.05 | 1.05 | 0.89 | 1.08 |
| 1.03 | 0.96 | 1.12 | 1.54 | 1.07 | 0.97 | NA | 0.64 | 0.91 | 1.08 | 1.26 |

| | | | | | | | | | | |
|---|---|---|---|---|---|---|---|---|---|---|
| 0.79 | 1.21 | 1.34 | 0.25 | 3.06 | 0.68 | 1.37 | 0.36 | 0.7 | 0.6 | 1.07 |
| 1.15 | 1.22 | 1.11 | 0.77 | 0.72 | 1.1 | 0.83 | 1.01 | 1.13 | 1.07 | 1.15 |
| 0.87 | 1 | 1.09 | 0.84 | 1.1 | 0.98 | 1.43 | 2.83 | 1.03 | 1.15 | 1.78 |
| 1.81 | 1.71 | 0.74 | 1.92 | 0.39 | 1.32 | 3.29 | 0.61 | 1.23 | 0.95 | 1.5 |
| 0.51 | 1.09 | 0.67 | 0.66 | 0.55 | 9.53 | 0.95 | 0.89 | 1.17 | 1.59 | 0.78 |
| 0.94 | 0.98 | 1.01 | 1.29 | 1.25 | 1.01 | 1 | 1.28 | 1.06 | 0.89 | 1.23 |
| 0.78 | 1.07 | 1.01 | 1.14 | 3.3 | 0.7 | 1.69 | 0.71 | 1.71 | 0.68 | 1.46 |
| 0.83 | 0.61 | 0.69 | 1.55 | 0.86 | 0.98 | 0.89 | 1.02 | 1.4 | 0.98 | 0.91 |
| 2.1 | 1.47 | 0.93 | 1.93 | 0.92 | 1.15 | 5.43 | 0.92 | 0.74 | 1.68 | 0.58 |
| 0.97 | 1.04 | 0.97 | 1.62 | 2.75 | 1 | 0.48 | 0.57 | 1.71 | 0.68 | 1.79 |
| 0.91 | 1.36 | 0.97 | 0.64 | 1.14 | 0.88 | 1.63 | 1.27 | 0.61 | 1.13 | 0.59 |
| 3.64 | 1 | 1.31 | 1 | 1.23 | 0.82 | 5.67 | 1.34 | 0.7 | 1.18 | 0.74 |
| 2.1 | 0.67 | 0.69 | 0.65 | 0.43 | 0.97 | 0.88 | 0.81 | 1.15 | 1.1 | 4.46 |
| 1.37 | 0.95 | 0.97 | 2.47 | 3.57 | 0.23 | 0.9 | 0.97 | 1.34 | 5.25 | 0.82 |
| 0.99 | 1.16 | 0.86 | 4.94 | 0.84 | 0.94 | 0.98 | 0.75 | 1.37 | 0.67 | 0.74 |
| 1.1 | 1.17 | 1.24 | 1.13 | 0.83 | 1.02 | 0.95 | 0.97 | 2.16 | 1.27 | 1.22 |
| 1.22 | 1.21 | 1.15 | 1.51 | 2.7 | 0.99 | 0.89 | 2.31 | 0.89 | 1.28 | 1.62 |
| 0.93 | 1.1 | 0.81 | 1.15 | 0.77 | 1.24 | 0.91 | 0.95 | 0.93 | 0.68 | 0.68 |
| 0.59 | 1.22 | 0.64 | 1.09 | 1.85 | 0.98 | 0.57 | 1.17 | 0.98 | 0.89 | 1.1 |
| 1.38 | 1.38 | 0.82 | 0.98 | 0.28 | 0.79 | 0.41 | 1.91 | 1.23 | 1.45 | 1.64 |
| 0.54 | 1 | 0.87 | 1.05 | 0.83 | 0.66 | 0.71 | 1.55 | 0.87 | 0.52 | 1.21 |
| 0.79 | 0.57 | 1.31 | 3.18 | 1.13 | 1.85 | 0.32 | 0.63 | 1.1 | 2.03 | 11.76 |
| 0.58 | 1.13 | 1.04 | 0.75 | 2.53 | 0.56 | 0.84 | 2.74 | 1.36 | 1 | 1.65 |
| 0.5 | 0.84 | 0.77 | 0.73 | 1.37 | 1.45 | 1.26 | 0.56 | 0.64 | 0.48 | 0.99 |
| 0.89 | 1.1 | 1.04 | 0.66 | 0.62 | 1.18 | 0.95 | 1.23 | 0.49 | 1.34 | 1.5 |
| 0.78 | 1.32 | 0.83 | 4.42 | 0.3 | 1.76 | 34.44 | 0.17 | 0.82 | 1.85 | 1.88 |
| 2.83 | 0.89 | 0.87 | 2.12 | 1.09 | 1.38 | 1.1 | 1.94 | 1.25 | 1.09 | 1.04 |
| 1.3 | 0.98 | 1.18 | 0.96 | 0.72 | 0.85 | 2.02 | 0.92 | 1.74 | 1.39 | 1.32 |
| 1.11 | 1.01 | 1.02 | 0.74 | 0.86 | 1.12 | NA | 2.05 | 0.95 | 0.99 | 0.82 |
| 4.45 | 1.24 | 0.97 | 0.48 | 0.62 | 0.72 | 1.24 | 0.94 | 1.11 | 1.25 | 1.26 |
| 4.17 | 0.4 | 1.8 | 2.86 | 1.71 | 1.39 | 0.65 | 0.66 | 0.96 | 0.7 | 1.13 |
| 0.89 | 1.04 | 0.97 | 1.06 | 1.04 | 0.77 | 0.64 | 1.31 | 0.74 | 1.08 | 1 |
| 4.71 | 0.71 | 0.93 | 7.1 | 0.71 | 0.95 | 1.5 | 1.62 | 0.88 | 2.66 | 0.26 |

| | | | | | | | | | | |
|---|---|---|---|---|---|---|---|---|---|---|
| 1.21 | 1 | 1.53 | 0.14 | 3.33 | 1.15 | 2.5 | 0.89 | 0.68 | 1.23 | 1.05 |
| 0.62 | 0.6 | 1.15 | 2.16 | 1.92 | 0.53 | 1 | 3.21 | 1.18 | 1.03 | 0.72 |
| 0.75 | 1.48 | 1.01 | 0.6 | 1.24 | 0.28 | 1.07 | 1.72 | 1.63 | 1.24 | 0.96 |
| 0.69 | 1.09 | 0.43 | 0.87 | 0.55 | 1.12 | 0.9 | 0.6 | 0.83 | 0.51 | 0.58 |
| 1.21 | 0.71 | 0.93 | 0.92 | 2.49 | 0.83 | 5.7 | 1.37 | 1.34 | 0.8 | 0.97 |
| 0.68 | 1.46 | 0.77 | 1.06 | 2.13 | 1.57 | 0.11 | 0.79 | 1.02 | 1 | 1.24 |
| 0.7 | 1.27 | 0.81 | 0.47 | 1.08 | 1.18 | 1.77 | 1.69 | 0.91 | 0.84 | 0.61 |
| 7.58 | 1.14 | 1.28 | 0.2 | 1.57 | 0.88 | 1.13 | 0.75 | 2.12 | 0.62 | 0.65 |
| 1 | 1.06 | 0.89 | 0.76 | 2.05 | 1.17 | 1.5 | 0.54 | 0.68 | 0.92 | 1.25 |
| 1.14 | 1.86 | 0.67 | 0.95 | 0.58 | 1.49 | 2.29 | 0.79 | 1.09 | 1.46 | 3.01 |
| 0.56 | 1.13 | 0.99 | 1.48 | 1.02 | 1.56 | 0.9 | 0.77 | 1.46 | 0.8 | 0.82 |
| 2.75 | 1.17 | 0.85 | 1.31 | 1.76 | 1.33 | 0.42 | 1.91 | 0.93 | 0.9 | 0.54 |
| 0.76 | 1.2 | 1.32 | 1 | 0.46 | 1.38 | 1 | 1.47 | 0.78 | 1.21 | 0.81 |
| 0.97 | 1.05 | 0.75 | 2.3 | 0.6 | 0.69 | 1.68 | 0.99 | 1.19 | 1.38 | 1.11 |
| 0.09 | 1.18 | 0.84 | 2.87 | 0.67 | 0.83 | 0.86 | 4 | 1.33 | 1.17 | 1.19 |
| 1.1 | 1.06 | 0.62 | 0.7 | 1.37 | 0.64 | Inf | 0.3 | 1.08 | 3.41 | 0.76 |
| 1.52 | 1.07 | 0.79 | 2.32 | 1.59 | 0.95 | 1.45 | 1.38 | 1.39 | 1.07 | 0.44 |
| 0.93 | 0.76 | 0.95 | 6.44 | 1.25 | 1.33 | 0.5 | 2.03 | 0.92 | 1.21 | 1.12 |
| 16.67 | 2.77 | 1.32 | 0.9 | 0.65 | 0.72 | 0.64 | 0.54 | 3.01 | 1.36 | 0.57 |
| 5.25 | 3.01 | 0.56 | 0 | 0.26 | 5.47 | 1.44 | 4.93 | 1.22 | 0.44 | 0.08 |
| 1.84 | 1.62 | 1.24 | 1.21 | 1.8 | 1.37 | 2.36 | 1.35 | 1.54 | 1.09 | 1.33 |
| 0.82 | 0.95 | 0.98 | 1.63 | 2.73 | 0.85 | 0.6 | 0.64 | 1.8 | 0.65 | 1.66 |
| 0.27 | 1.01 | 1.04 | 0.48 | 1.12 | 0.29 | 0.64 | 9.48 | 1.72 | 1.61 | 1.99 |
| 0.45 | 0.87 | 0.88 | 1.53 | 0.93 | 0.75 | 0.47 | 8.38 | 1.08 | 0.99 | 2.3 |
| 1.65 | 1.22 | 0.97 | 1.74 | 0.68 | 0.95 | 1.23 | 1.24 | 1.16 | 1.86 | 0.63 |
| 2.82 | 0.92 | 0.98 | 1.93 | 1.72 | 0.8 | 0.53 | 2.58 | 0.75 | 0.65 | 0.81 |
| 0.71 | 0.94 | 1.22 | 1.39 | 0.57 | 1.62 | 1.23 | 0.6 | 1.58 | 0.44 | 0.85 |
| 0.55 | 1.38 | 1.26 | 0.39 | 2.04 | 0.42 | 1.09 | 1.34 | 1.29 | 0.72 | 0.13 |
| 0.56 | 1.02 | 0.76 | 1.97 | 1.02 | 0.87 | 0.84 | 0.8 | 0.62 | 0.95 | 1.4 |
| 0.58 | 0.16 | 0.57 | 0.53 | 1.16 | 3.41 | 2.5 | 0.5 | 0.61 | 1.09 | 1 |
| 0.41 | 1.13 | 1.15 | 0.56 | 2.7 | 1.75 | 0.97 | 1.4 | 0.81 | 9 | 0.1 |
| 0.48 | 5.55 | 0.79 | 4.32 | 1.17 | 1.73 | 1.22 | 2.69 | 1.09 | 1.18 | 0.92 |
| 8.39 | 0.44 | 6.97 | 0.53 | 4.52 | 2.74 | 0.83 | 1.37 | 1.21 | 4.51 | 1.67 |

| | | | | | | | | | | |
|---|---|---|---|---|---|---|---|---|---|---|
| 0.35 | 1.16 | 0.42 | 1.17 | 1.52 | 1.05 | 0.87 | 0.64 | 0.67 | 0.97 | 0.78 |
| 0.73 | 1.04 | 1.18 | 0.41 | 0.69 | 1.14 | 1.13 | 2.57 | 1.13 | 1.39 | 1.03 |
| 0.51 | 0.53 | 1.43 | 1.02 | 0.37 | 1.54 | 6.73 | 0.58 | 3.57 | 0.58 | 2.03 |
| 0.94 | 0.94 | 1.05 | 0.43 | 0.62 | 0.37 | 0.74 | 0.12 | 1.01 | 0.96 | 0.7 |
| 1.21 | 1.16 | 0.79 | 3.16 | 1.02 | 1.81 | 0.34 | 0.23 | 1.27 | 0.99 | 8.84 |
| 0.92 | 0.92 | 1.16 | 1.01 | 1.03 | 1.09 | 0.7 | 0.34 | 1.06 | 1.95 | 6.22 |
| 3.11 | 0.99 | 1.35 | 1.31 | 1.15 | 1.1 | 3.6 | 3.08 | 0.33 | 1.82 | 0.88 |
| 1.36 | 1.02 | 1.06 | 1.91 | 1.02 | 0.76 | 0.94 | 3.56 | 1.31 | 0.3 | 1.25 |
| 2.94 | 1.22 | 1.15 | 1.4 | 0.86 | 0.94 | 14.56 | 2.6 | 0.67 | 2.12 | 0.3 |
| 6.02 | 1.7 | 1.11 | 4.4 | 0.83 | 1.02 | Inf | 1.46 | 1.04 | 1.7 | 2.45 |
| 4.88 | 0.95 | 1.21 | 0.52 | 0.73 | 0.74 | 0.84 | 1.38 | 0.99 | 0.71 | 0.97 |
| 17.38 | 0.69 | 0.48 | 0.52 | 0.8 | 4.66 | 7.09 | 1 | 3.14 | 6.46 | 2.45 |
| 1.23 | 1 | 1.61 | 1.16 | 1.54 | 2 | 0.95 | 1.34 | 0.81 | 1.32 | 1.15 |
| 1.5 | 1.62 | 1 | 1.14 | 0.65 | 0.43 | 2.6 | 0.93 | 0.65 | 1.5 | 0.93 |

| 219195_at | 221530_s_at | 91816_f_at | 204908_s_at | 202326_at |
|---|---|---|---|---|
| 5.28 | 5.23 | 1.07 | 1.1 | 1.03 |
| 1.39 | 0.73 | 0.74 | 1.56 | 1.05 |
| 1 | 0.51 | 1.4 | 0.55 | 1.23 |
| 4.86 | 2.7 | 0.75 | 0.83 | 1.18 |
| 1.92 | 1.78 | 0.94 | 0.96 | 0.83 |
| 2.15 | 0.99 | 2.45 | 1.08 | 1.59 |
| 0.29 | 0.09 | 0.52 | 1.12 | 4 |
| 2.36 | 0.88 | 2.11 | 1.29 | 0.92 |
| 0.93 | 0.6 | 0.7 | 1.14 | 0.74 |
| 8.72 | 0.41 | 1.03 | 1.32 | 0.45 |
| 1.52 | 3.75 | 0.63 | 1.2 | 1.01 |
| 1.26 | 0.66 | 1.33 | 1.09 | 1.14 |
| 1.5 | 1.12 | 1.96 | 0.73 | 0.88 |
| 30.01 | 23.73 | 0.27 | 0.95 | 0.11 |
| 2.91 | 0.85 | 1.03 | 0.81 | 1.98 |
| 3.93 | 0.84 | 1.04 | 0.75 | 0.99 |
| 0.12 | 0.97 | 0.96 | 1.02 | 1.32 |
| 0.54 | 1.08 | 1.15 | 1.63 | 1.29 |
| 0.67 | 1.37 | 1.15 | 1.19 | 1.97 |
| 1.19 | 5.92 | 1.15 | 0.8 | 0.26 |
| 0.36 | 1.98 | 1.41 | 1.39 | 7.62 |
| 15.33 | 60.47 | 0.4 | 2.48 | 1.77 |
| 5.78 | 7.45 | 0.64 | 1.12 | 1.14 |
| 0.5 | 3.99 | 0.7 | 1.34 | 1.42 |
| 0.06 | 0.09 | 0.44 | 1.97 | 1.25 |
| 0.19 | 1.99 | 0.81 | 0.67 | 1.06 |
| 0.57 | 1.53 | 0.76 | 1.35 | 1.05 |
| 1.51 | 2.92 | 0.77 | 2.67 | 1.25 |
| 1.08 | 215.82 | 1.25 | 1.62 | 0.82 |
| 0.97 | 0.8 | 1.1 | 0.95 | 0.79 |

| | | | | |
|---|---|---|---|---|
| 5.58 | 0.75 | 0.81 | 0.91 | 0.71 |
| 29.66 | 0.6 | 2.3 | 2.56 | 0.85 |
| 0.97 | 1.37 | 1.08 | 0.89 | 0.96 |
| 2.16 | 0.53 | 1.39 | 0.54 | 0.73 |
| 1.5 | 2.83 | 9.64 | 0.56 | 1.04 |
| 0.67 | 0.65 | 0.79 | 5.08 | 1.18 |
| 20.97 | 15.87 | 0.48 | 0.91 | 1.72 |
| 0.07 | 0.58 | 1.01 | 1.05 | 1.17 |
| 0.83 | 0.31 | 0.96 | 2.82 | 1 |
| 0.53 | 17.29 | 1.34 | 2.02 | 0.86 |
| 1.75 | 2.8 | 0.77 | 2.05 | 1.22 |
| 1.59 | 0.36 | 1.17 | 0.58 | 1.14 |
| 0.42 | 3.14 | 0.79 | 0.77 | 0.35 |
| 1.4 | 1.55 | 1.4 | 1.39 | 0.23 |
| 0.56 | 2.67 | 0.73 | 0.78 | 0.5 |
| 21.09 | 2.75 | 1.14 | 1.45 | 0.94 |
| 2.33 | 1.23 | 1.31 | 0.48 | 1.27 |
| 4.56 | 1.19 | 1.01 | 1.06 | 1.93 |
| 1.17 | 1.05 | 0.99 | 1.13 | 0.88 |
| 3.49 | 0.58 | 1.28 | 1.78 | 0.73 |
| 3.5 | 1.17 | 0.98 | 0.97 | 0.91 |
| 0.22 | 0.86 | 0.7 | 0.64 | 1.13 |
| 0.51 | 0.39 | 1.02 | 0.46 | 1.13 |
| 1.47 | 1.14 | 1.25 | 1.02 | 1.35 |
| 1.06 | 5.03 | 0.91 | 0.79 | 0.53 |
| 0.12 | 0.8 | 1.28 | 0.92 | 1.03 |
| 0.2 | 0.33 | 1.45 | 2.88 | 0.98 |
| 2.37 | 2.82 | 0.56 | 1.44 | 1.33 |
| 1.49 | 1.61 | 0.81 | 1.08 | 0.86 |
| 0.27 | 5.79 | 0.54 | 1.18 | 0.33 |
| 0.61 | 0.86 | 0.95 | 2.08 | 0.72 |
| 1.38 | 1.85 | 2.28 | 0.68 | 0.88 |
| 0.81 | 2.34 | 0.69 | 2.17 | 1.01 |

| | | | | |
|---|---|---|---|---|
| 0.25 | 3.25 | 1.25 | 1.47 | 0.73 |
| 4.71 | 11.91 | 1.11 | 1.16 | 1.16 |
| 2.8 | 42.4 | 1.1 | 1.21 | 1.26 |
| 1.42 | 4.24 | 0.96 | 1.67 | 1.27 |
| 4.7 | 1.13 | 0.98 | 0.7 | 0.69 |
| 0.65 | 0.56 | 1.34 | 0.72 | 1.3 |
| 2.83 | 0.53 | 1.07 | 1.35 | 1.82 |
| 0.64 | 0.57 | 1.29 | 1.68 | 1.96 |
| 1.19 | 2.21 | 2.11 | 1.78 | 1.37 |
| 0.52 | 4.86 | 1.75 | 1.05 | 0.62 |
| 5.73 | 0.5 | 1.86 | 1.17 | 1.36 |
| 0.1 | 4.86 | 0.75 | 1.53 | 2.18 |
| 0.97 | 0.73 | 0.83 | 1.4 | 0.61 |
| 1.23 | 1.39 | 0.66 | 1.59 | 1.41 |
| 0.17 | 2.75 | 1.34 | 0.72 | 1.38 |
| 1.4 | 0.16 | 0.79 | 1.62 | 0.09 |
| 1.89 | 3.77 | 0.78 | 5.1 | 0.74 |
| 4.88 | 1.24 | 2.52 | 0.36 | 1.02 |
| 1.54 | 3.2 | 1.01 | 1.05 | 0.84 |
| 3.51 | 1.52 | 3.11 | 2.48 | 5 |
| 2.21 | 16.5 | 3.3 | 2.56 | 0.45 |
| 3.34 | 0.59 | 0.91 | 1.48 | 1.02 |
| 0.17 | 0.3 | 0.46 | 1.05 | 5.38 |
| 1.15 | 0.78 | 1.37 | 2.25 | 2.27 |
| 6.37 | 0.54 | 1.31 | 0.97 | 0.95 |
| 1.4 | 0.64 | 0.7 | 1.42 | 0.76 |
| 0.76 | 0.91 | 1.01 | 0.93 | 1.37 |
| 16.98 | 4.05 | 0.89 | 2.68 | 0.93 |
| 1.16 | 1.96 | 0.83 | 0.86 | 0.83 |
| 3.73 | 0.8 | 0.91 | 0.96 | 1.06 |
| 0.8 | 1.01 | 1.26 | 1.24 | 1.06 |
| 3.11 | 5.07 | 0.41 | 0.47 | 0.62 |
| 7.15 | 3.09 | 0.78 | 2.23 | 0.73 |

| | | | | |
|---|---|---|---|---|
| 2.09 | 0.7 | 0.76 | 0.24 | 1.53 |
| 1.57 | 3.06 | 0.83 | 0.89 | 1.02 |
| 0.99 | 0.89 | 1.18 | 1.2 | 1.03 |
| 1.9 | 8.79 | 1.87 | 1.06 | 1.27 |
| 10.09 | 0.94 | 1.11 | 0.68 | 0.89 |
| 1.58 | 0.23 | 0.35 | 0.59 | 0.66 |
| 1.53 | 3.65 | 0.77 | 1.74 | 0.92 |
| 0.89 | 6.05 | 0.69 | 1.38 | 0.57 |
| 3.25 | 1.58 | 0.77 | 0.91 | 0.77 |
| 0.81 | 0.58 | 1.17 | 1 | 0.9 |
| 0.76 | 0.85 | 0.96 | 1.18 | 1.61 |
| 1.78 | 1.98 | 0.99 | 1.32 | 0.92 |
| 0.9 | 1.32 | 0.87 | 1.09 | 0.76 |
| 1.26 | 0.56 | 1.34 | 0.95 | 1.19 |
| 1.08 | 2.59 | 0.94 | 2.18 | 0.9 |
| 1.14 | 17 | 1.42 | 0.84 | 1.07 |
| 1.14 | 2.75 | 1.08 | 1.68 | 0.87 |
| 1.27 | 2.96 | 1.06 | 2.19 | 1.31 |
| 7.4 | 0.81 | 1.04 | 2.18 | 1.07 |
| 3.83 | 1.35 | 0.93 | 0.57 | 1.13 |
| 0.92 | 0.98 | 1.06 | 0.68 | 0.99 |
| 1.58 | 1.23 | 0.94 | 0.45 | 0.89 |
| 0.92 | 0.74 | 1.08 | 1.09 | 1.06 |
| 1.24 | 0.04 | 1.04 | 1.29 | 1.54 |
| 1.7 | 0.86 | 1.08 | 1.85 | 1.32 |
| 2.36 | 2.79 | 0.83 | 0.92 | 0.72 |
| 0.15 | 0.51 | 1.04 | 0.98 | 0.98 |
| 1.93 | 1.31 | 1.15 | 2.09 | 1.05 |
| 0.9 | 0.23 | 0.93 | 0.45 | 0.88 |
| 1.49 | 1.11 | 0.98 | 1.08 | 0.8 |
| 0.72 | 2.57 | 0.95 | 3.64 | 1.04 |
| 4.71 | 11.91 | 1.11 | 1.16 | 1.16 |
| 1.44 | 0.9 | 0.96 | 1.27 | 1.04 |

| | | | | |
|---|---|---|---|---|
| 4.25 | 1.38 | 1.39 | 0.6 | 1.04 |
| 0.98 | 6.91 | 1.56 | 1.4 | 1.12 |
| 1.58 | 0.3 | 1.16 | 0.89 | 0.71 |
| 1.52 | 1.05 | 0.88 | 6.11 | 0.89 |
| 1.25 | 0.79 | 0.87 | 0.7 | 0.88 |
| 2 | 1.07 | 1.06 | 0.99 | 1.35 |
| 0.98 | 0.5 | 1.06 | 1.25 | 0.76 |
| 4.47 | 0.97 | 0.81 | 1.27 | 0.68 |
| 1.46 | 1.18 | 0.97 | 0.74 | 1.07 |
| 21.73 | 4.92 | 1.02 | 1.31 | 1.36 |
| 1.07 | 1.1 | 0.88 | 1.15 | 0.86 |
| 2.07 | 0.61 | 0.84 | 1.08 | 0.85 |
| 1.59 | 2.39 | 0.96 | 2.2 | 1.26 |
| 3.68 | 0.45 | 0.68 | 1.93 | 0.98 |
| 3.14 | 16.95 | 4.55 | 3.06 | 0.2 |
| 0.84 | 0.94 | 1.3 | 1.35 | 1.04 |
| 1.23 | 2.11 | 0.95 | 0.7 | 0.79 |
| 0.48 | 2.88 | 1.31 | 1.57 | 1.25 |
| 0.89 | 0.83 | 0.92 | 0.93 | 1.06 |
| 1.04 | 0.12 | 0.82 | 0.55 | 0.88 |
| 0.75 | 1.85 | 0.61 | 0.98 | 0.63 |
| 1.19 | 1.29 | 0.75 | 0.95 | 1.04 |
| 1.48 | 0.63 | 1.23 | 0.72 | 1.19 |
| 0.72 | 0.53 | 1.19 | 1.04 | 0.9 |
| 0.64 | 0.73 | 0.84 | 0.75 | 0.56 |
| 2.14 | 0.41 | 1.06 | 1.13 | 0.87 |
| 2.35 | 0.74 | 0.51 | 1.28 | 1.28 |
| 1.21 | 1.29 | 1.1 | 0.99 | 0.48 |
| 0.95 | 1.03 | 1 | 1.08 | 0.81 |
| 3 | 0.68 | 0.84 | 0.89 | 0.6 |
| 3.78 | 1.3 | 1.15 | 0.66 | 1.28 |
| 0.99 | 1.08 | 1.25 | 1.43 | 0.97 |
| 2.66 | 4.58 | 0.92 | 1 | 1.15 |

| | | | | |
|---|---|---|---|---|
| 0.88 | 2.64 | 1.66 | 2.04 | 1.24 |
| 1.81 | 1.1 | 1.46 | 0.72 | 1.34 |
| 0.89 | 1.21 | 1.1 | 0.94 | 1.02 |
| 1.97 | 0.38 | 1.28 | 1.31 | 0.35 |
| 1.54 | 7.12 | 1.26 | 1.09 | 1 |
| 2.25 | 1.8 | 1.07 | 1.87 | 1 |
| 5.46 | 1.06 | 0.78 | 0.87 | 0.66 |
| 1.03 | 3.41 | 0.72 | 0.42 | 0.91 |
| 4.35 | 1.12 | 0.9 | 2.04 | 1.31 |
| 1.28 | 0.86 | 0.71 | 0.59 | 0.74 |
| 0.99 | 0.87 | 1.02 | 1.01 | 0.86 |
| 0.85 | 2.58 | 0.86 | 0.63 | 1.57 |
| 0.48 | 1.74 | 0.6 | 0.82 | 0.74 |
| 2.75 | 2.41 | 0.94 | 0.95 | 0.9 |
| 1.88 | 1.06 | 0.84 | 8.15 | 0.91 |
| 1.44 | 1.01 | 0.88 | 1.52 | 1.53 |
| 3.02 | 2.37 | 0.92 | 0.94 | 1.14 |
| 0.87 | 1.17 | 0.87 | 1.61 | 0.75 |
| 2.61 | 1.61 | 0.58 | 1.14 | 1.16 |
| 0.69 | 1.05 | 1.38 | 1.02 | 0.72 |
| 1.3 | 0.57 | 1.32 | 1.02 | 1 |
| 0.62 | 6.08 | 1.18 | 1.68 | 3.23 |
| 1.21 | 1.23 | 1.08 | 0.89 | 0.98 |
| 0.51 | 2.51 | 1.46 | 0.49 | 1.15 |
| 0.92 | 1.09 | 0.89 | 0.99 | 0.97 |
| 26.67 | 0.24 | 0.75 | 1.88 | 0.7 |
| 0.83 | 0.83 | 1.21 | 0.98 | 0.86 |
| 1.15 | 2 | 1.02 | 1.03 | 1.67 |
| 6.75 | 2.51 | 1.11 | 1.11 | 0.94 |
| 0.94 | 3.5 | 0.96 | 1.62 | 0.95 |
| 7.19 | 5.56 | 1.31 | 1.13 | 0.83 |
| 2 | 0.82 | 0.8 | 1.44 | 0.67 |
| 0.83 | 0.93 | 0.73 | 0.88 | 1 |

| | | | | |
|---|---|---|---|---|
| 1.19 | 1.24 | 0.56 | 1.33 | 1.05 |
| 0.33 | 1.69 | 1.47 | 0.87 | 2.1 |
| 0.48 | 1.18 | 0.79 | 1.33 | 1.05 |
| 0.55 | 0.81 | 0.57 | 0.49 | 0.62 |
| 2.78 | 1.66 | 0.97 | 1.15 | 1.15 |
| 4.25 | 2.77 | 0.93 | 0.72 | 0.63 |
| 0.28 | 0.21 | 0.9 | 0.64 | 0.68 |
| 2.23 | 1.41 | 1.07 | 2.23 | 0.89 |
| 1.19 | 1.99 | 2.12 | 2.13 | 1.35 |
| 1.83 | 0.48 | 0.72 | 0.95 | 0.74 |
| 0.93 | 1.15 | 0.88 | 1.13 | 0.71 |
| 0.5 | 0.81 | 1 | 1.03 | 0.95 |
| 1.04 | 1.11 | 0.5 | 1.04 | 1.3 |
| 0.66 | 3.2 | 1.24 | 2.88 | 1.16 |
| 1.85 | 0.61 | 0.8 | 1.14 | 0.81 |
| 3.31 | 0.71 | 1.17 | 1.03 | 0.84 |
| 1.48 | 0.86 | 1.38 | 1.05 | 1.37 |
| 0.62 | 1.32 | 0.86 | 1.8 | 0.72 |
| 0.61 | 2.16 | 0.77 | 4.09 | 0.57 |
| 2.04 | 0.11 | 0.57 | 0.68 | 0.47 |
| 1.5 | 0.85 | 1.57 | 1.6 | 0.92 |
| 3.48 | 0.65 | 0.78 | 0.77 | 0.75 |
| 0.18 | 1.7 | 1.03 | 1.04 | 0.86 |
| 1.16 | 0.3 | 0.86 | 0.9 | 0.84 |
| 1.94 | 1.17 | 0.9 | 1.04 | 1.07 |
| 3.48 | 1.58 | 0.9 | 1.38 | 0.85 |
| 0.87 | 1.05 | 1.39 | 0.23 | 0.99 |
| 3.1 | 1.82 | 1.1 | 1.6 | 2.37 |
| 1.03 | 2.42 | 0.97 | 1.15 | 0.81 |
| 0.27 | 3.65 | 0.25 | 1.3 | 0.28 |
| 0.69 | 4 | 1.09 | 1.87 | 1.26 |
| 1.06 | 0.77 | 1.08 | 31.07 | 0.89 |
| 2.52 | 2.25 | 4.51 | 1.11 | 0.48 |

| | | | | |
|---|---|---|---|---|
| 1.09 | 0.76 | 1.17 | 1.13 | 0.99 |
| 0.81 | 0.93 | 1.19 | 0.9 | 1.36 |
| 2.3 | 5.5 | 1.59 | 1.49 | 0.89 |
| 4.54 | 0.12 | 0.51 | 0.21 | 0.77 |
| 3.14 | 0.64 | 0.71 | 1.05 | 0.87 |
| 1.12 | 3.68 | 0.76 | 1.78 | 0.89 |
| 3.31 | 1.97 | 1.08 | 0.85 | 0.91 |
| 0.44 | 0.7 | 0.98 | 1.21 | 0.98 |
| 2.89 | 2.04 | 1.04 | 1.69 | 0.81 |
| 0.23 | 1.6 | 1.47 | 1.02 | 1.16 |
| 0.64 | 0.2 | 1.17 | 0.86 | 1.13 |
| 0.08 | 4.72 | 0.13 | 0.19 | 3.75 |
| 1.83 | 0.59 | 1.05 | 0.13 | 0.99 |
| 0.33 | 5 | 1 | 1.63 | 0.06 |
